# The 2022 report of the *Lancet* Countdown on health and climate change: health at the mercy of fossil fuels

**DOI:** 10.1016/S0140-6736(22)01540-9

**Published:** 2022-10-25

**Authors:** Marina Romanello, Claudia Di Napoli, Paul Drummond, Carole Green, Harry Kennard, Pete Lampard, Daniel Scamman, Nigel Arnell, Sonja Ayeb-Karlsson, Lea Berrang Ford, Kristine Belesova, Kathryn Bowen, Wenjia Cai, Max Callaghan, Diarmid Campbell-Lendrum, Jonathan Chambers, Kim R van Daalen, Carole Dalin, Niheer Dasandi, Shouro Dasgupta, Michael Davies, Paula Dominguez-Salas, Robert Dubrow, Kristie L Ebi, Matthew Eckelman, Paul Ekins, Luis E Escobar, Lucien Georgeson, Hilary Graham, Samuel H Gunther, Ian Hamilton, Yun Hang, Risto Hänninen, Stella Hartinger, Kehan He, Jeremy J Hess, Shih-Che Hsu, Slava Jankin, Louis Jamart, Ollie Jay, Ilan Kelman, Gregor Kiesewetter, Patrick Kinney, Tord Kjellstrom, Dominic Kniveton, Jason KW Lee, Bruno Lemke, Yang Liu, Zhao Liu, Melissa Lott, Martin Lotto Batista, Rachel Lowe, Frances MacGuire, Maquins Odhiambo Sewe, Jaime Martinez-Urtaza, Mark Maslin, Lucy McAllister, Alice McGushin, Celia McMichael, Zhifu Mi, James Milner, Kelton Minor, Jan C Minx, Nahid Mohajeri, Maziar Moradi-Lakeh, Karyn Morrissey, Simon Munzert, Kris A Murray, Tara Neville, Maria Nilsson, Nick Obradovich, Megan B O’Hare, Tadj Oreszczyn, Matthias Otto, Fereidoon Owfi, Olivia Pearman, Mahnaz Rabbaniha, Elizabeth JZ Robinson, Joacim Rocklöv, Renee N Salas, Jan C Semenza, Jodi D Sherman, Liuhua Shi, Joy Shumake-Guillemot, Grant Silbert, Mikhail Sofiev, Marco Springmann, Jennifer Stowell, Meisam Tabatabaei, Jonathon Taylor, Joaquin Triñanes, Fabian Wagner, Paul Wilkinson, Matthew Winning, Marisol Yglesias-González, Shihui Zhang, Peng Gong, Hugh Montgomery, Anthony Costello

**Affiliations:** Institute for Global Health, University College London, London, UK; School of Agriculture Policy and Development, University of Reading, Reading, UK; Institute for Sustainable Resources, University College London, London, UK; Department of Global Health, Centre for Health and the Global Environment, University of Washington, Seattle, WA, USA; UCL Energy Institute, University College London, London, UK; Department of Health Sciences, University of York, York, UK; Institute for Sustainable Resources, University College London, London, UK; Department of Meteorology, University of Reading, Reading, UK; Institute for Risk and Disaster Reduction, University College London, London, UK; School of Earth and Environment, University of Leeds, Leeds, UK; Centre on Climate Change and Planetary Health, London School of Hygiene & Tropical Medicine, London, UK; School of Population Health, University of Melbourne, Melbourne, VIC, Australia; Department of Earth System Science, Tsinghua University, Beijing, China; Mercator Research Institute on Global Commons and Climate Change, Berlin, Germany; Department of Environment, Climate Change, and Health, World Health Organization, Geneva, Switzerland; Institute of Environmental Sciences, University of Geneva, Geneva, Switzerland; Cardiovascular Epidemiology Unit, Department of Public Health & Primary Care, University of Cambridge, Cambridge, UK; Institute for Sustainable Resources, University College London, London, UK; School of Government, University of Birmingham, Birmingham, UK; Economic Analysis of Climate Impacts and Policy Division, Centro Euro-Mediterraneo sui Cambiamenti Climatici, Venice, Italy; Institute for Environmental Design and Engineering, University College London, London, UK; Natural Resources Institute, University of Greenwich, London, UK; Department of Environmental Health Sciences and Yale Center on Climate Change and Health, Yale University, New Haven, CT, USA; Department of Global Health, Centre for Health and the Global Environment, University of Washington, Seattle, WA, USA; Department of Civil and Environmental Engineering, Northeastern University, Boston, MA, USA; Institute for Sustainable Resources, University College London, London, UK; Department of Fish and Wildlife Conservation, Virginia Polytechnic Institute and State University, Blacksburg, VA, USA; Department of Geography, University College London, London, UK; Department of Health Sciences, University of York, York, UK; NUS Yong Loo Lin School of Medicine, National University Singapore, Singapore; UCL Energy Institute, University College London, London, UK; Rollins School of Public Health, Emory University, Atlanta, GA, USA; Finnish Meteorological Institute, Helsinki, Finland; Facultad de Salud Publica y Administracion, Universidad Peruana Cayetano Heredia, Lima, Peru; Bartlett Faculty of the Built Environment, University College London, London, UK; Department of Global Health, Centre for Health and the Global Environment, University of Washington, Seattle, WA, USA; UCL Energy Institute, University College London, London, UK; Data Science Lab, Hertie School, Berlin, Germany; Public Group International, London, UK; Heat and Health Research Incubator, Faculty of Medicine and Health, University of Sydney, Camperdown, NSW, Australia; Institute for Global Health, University College London, London, UK; Energy, Climate, and Environment Program, International Institute for Applied Systems Analysis, Laxenburg, Austria; Department of Environmental Health, School of Public Health, Boston University, Boston, MA, USA; Health and Environmental International Trust, Nelson, New Zealand; School of Global Studies, University of Sussex, Sussex, UK; NUS Yong Loo Lin School of Medicine, National University Singapore, Singapore; School of Health, Nelson Marlborough Institute of Technology, Nelson, New Zealand; Rollins School of Public Health, Emory University, Atlanta, GA, USA; Department of Earth System Science, Tsinghua University, Beijing, China; Center on Global Energy Policy, Columbia University, New York, NY, USA; Air Quality and Greenhouse Gases Programme, International Institute for Applied Systems Analysis, Laxenburg, Austria; Barcelona Supercomputing Center, Centro Nacional de Supercomputacion, Barcelona, Spain; Catalan Institution for Research and Advanced Studies and Barcelona Supercomputing Center, Barcelona, Spain; Institute for Global Health, University College London, London, UK; Department of Public Health and Clinical Medicine, Section of Sustainable Health, Umeå University, Umeå, Sweden; Department of Genetics and Microbiology, Universitat de Barcelona, Barcelona, Spain; Department of Geography, University College London, London, UK; Center for Energy Markets, Technical University of Munich, Munich, Germany; MRC Epidemiology Unit, University of Cambridge, Cambridge, UK; School of Geography, Earth and Atmospheric Sciences, University of Melbourne, Melbourne, VIC, Australia; Barlett School of Sustainable Construction, University of London, London, UK; Department of Public Health, Environment, and Society, London School of Hygiene & Tropical Medicine, London, UK; Copenhagen Center for Social Data Science, University of Copenhagen, Copenhagen, Denmark; Mercator Research Institute on Global Commons and Climate Change, Berlin, Germany; Institute for Environmental Design and Engineering, University College London, London, UK; Preventative Medicine and Public Health Research Centre, Psychosocial Health Research Institute, Iran University of Medical Sciences, Tehran, Iran; Department of Technology, Management and Economics Sustainability, Technical University of Denmark, Lyngby, Denmark; Data Science Lab, Hertie School, Berlin, Germany; MRC Unit The Gambia at LSHTM, London School of Hygiene & Tropical Medicine, London, UK; Department of Environment, Climate Change, and Health, World Health Organization, Geneva, Switzerland; Department of Epidemiology and Global Health, Umeå University, Umeå, Sweden; Centre for Humans and Machines, Max Planck Institute for Human Development, Berlin, Germany; Institute for Global Health, University College London, London, UK; UCL Energy Institute, University College London, London, UK; Department of Arts, Media, and Digital Technologies, Nelson Marlborough Institute of Technology, Nelson, New Zealand; Iranian Fisheries Research Institute, Agricultural Research, Education, and Extension Organisation, Tehran, Iran; Cooperative Institute of Research in Environmental Sciences, University of Colorado Boulder, Boulder, CO, USA; Iranian Fisheries Research Institute, Agricultural Research, Education, and Extension Organisation, Tehran, Iran; Grantham Research Institute on Climate Change and the Environment, London School of Economics and Political Science, London, UK; Heidelberg Institute for Global Health and Interdisciplinary Centre for Scientific Computing, University of Heidelberg, Heidelberg, Germany; Harvard Medical School, Harvard University, Boston, MA, USA; Heidelberg Institute for Global Health and Interdisciplinary Centre for Scientific Computing, University of Heidelberg, Heidelberg, Germany; Department of Anesthesiology, Yale University, New Haven, CT, USA; Rollins School of Public Health, Emory University, Atlanta, GA, USA; WHO–WMO Joint Climate and Health Office, Geneva, Switzerland; Melbourne Medical School, University of Melbourne, Melbourne, VIC, Australia; Finnish Meteorological Institute, Helsinki, Finland; Environmental Change Institute, University of Oxford, Oxford, UK; Department of Environmental Health, School of Public Health, Boston University, Boston, MA, USA; Institute of Tropical Aquaculture and Fisheries, Universiti Malaysia Terengganu, Malaysia; Department of Civil Engineering, Tampere University, Tampere, Finland; Department of Electronics and Computer Science, Universidade de Santiago de Compostela, Santiago, Spain; Energy, Climate, and Environment Program, International Institute for Applied Systems Analysis, Laxenburg, Austria; Department of Public Health, Environment, and Society, London School of Hygiene & Tropical Medicine, London, UK; Institute for Sustainable Resources, University College London, London, UK; Centro Latinoamericano de Excelencia en Cambio Climático y Salud, Universidad Peruana Cayetano Heredia, Lima, Peru; Department of Earth System Science, Tsinghua University, Beijing, China; Department of Geography, University of Hong Kong, Hong Kong Special Administrative Region, China; Centre for Human Health and Performance, University College London, London, UK; Institute for Global Health, University College London, London, UK

## Abstract

The 2022 report of the *Lancet* Countdown is published as the world confronts profound and concurrent systemic shocks. Countries and health systems continue to contend with the health, social, and economic impacts of the COVID-19 pandemic, while Russia’s invasion of Ukraine and a persistent fossil fuel overdependence has pushed the world into global energy and cost-of-living crises. As these crises unfold, climate change escalates unabated. Its worsening impacts are increasingly affecting the foundations of human health and wellbeing, exacerbating the vulnerability of the world’s populations to concurrent health threats.

During 2021 and 2022, extreme weather events caused devastation across every continent, adding further pressure to health services already grappling with the impacts of the COVID-19 pandemic. Floods in Australia, Brazil, China, western Europe, Malaysia, Pakistan, South Africa, and South Sudan caused thousands of deaths, displaced hundreds of thousands of people, and caused billions of dollars in economic losses. Wildfires caused devastation in Canada, the USA, Greece, Algeria, Italy, Spain, and Türkiye, and record temperatures were recorded in many countries, including Australia, Canada, India, Italy, Oman, Türkiye, Pakistan, and the UK. With advancements in the science of detection and attribution studies, the influence of climate change over many events has now been quantified.

Because of the rapidly increasing temperatures, vulnerable populations (adults older than 65 years, and children younger than one year of age) were exposed to 3·7 billion more heatwave days in 2021 than annually in 1986–2005 (indicator 1.1.2), and heat-related deaths increased by 68% between 2000–04 and 2017–21 (indicator 1.1.5), a death toll that was significantly exacerbated by the confluence of the COVID-19 pandemic.

Simultaneously, the changing climate is affecting the spread of infectious diseases, putting populations at higher risk of emerging diseases and co-epidemics. Coastal waters are becoming more suitable for the transmission of Vibrio pathogens; the number of months suitable for malaria transmission increased by 31·3% in the highland areas of the Americas and 13·8% in the highland areas of Africa from 1951–60 to 2012–21, and the likelihood of dengue transmission rose by 12% in the same period (indicator 1.3.1). The coexistence of dengue outbreaks with the COVID-19 pandemic led to aggravated pressure on health systems, misdiagnosis, and difficulties in management of both diseases in many regions of South America, Asia, and Africa.

The economic losses associated with climate change impacts are also increasing pressure on families and economies already challenged with the synergistic effects of the COVID-19 pandemic and the international cost-of-living and energy crises, further undermining the socioeconomic determinants that good health depends on. Heat exposure led to 470 billion potential labour hours lost globally in 2021 (indicator 1.1.4), with potential income losses equivalent to 0·72% of the global economic output, increasing to 5·6% of the GDP in low Human Development Index (HDI) countries, where workers are most vulnerable to the effects of financial fluctuations (indicator 4.1.3). Meanwhile, extreme weather events caused damage worth US$253 billion in 2021, particularly burdening people in low HDI countries in which almost none of the losses were insured (indicator 4.1.1).

Through multiple and interconnected pathways, every dimension of food security is being affected by climate change, aggravating the impacts of other coexisting crises. The higher temperatures threaten crop yields directly, with the growth seasons of maize on average 9 days shorter in 2020, and the growth seasons of winter wheat and spring wheat 6 days shorter than for 1981–2010 globally (indicator 1.4). The threat to crop yields adds to the rising impact of extreme weather on supply chains, socioeconomic pressures, and the risk of infectious disease transmission, undermining food availability, access, stability, and utilisation. New analysis suggests that extreme heat was associated with 98 million more people reporting moderate to severe food insecurity in 2020 than annually in 1981–2010, in 103 countries analysed (indicator 1.4). The increasingly extreme weather worsens the stability of global food systems, acting in synergy with other concurrent crises to reverse progress towards hunger eradication. Indeed, the prevalence of undernourishment increased during the COVID-19 pandemic, and up to 161 million more people faced hunger during the COVID-19 pandemic in 2020 than in 2019. This situation is now worsened by Russia’s invasion of Ukraine and the energy and cost-of-living crises, with impacts on international agricultural production and supply chains threatening to result in 13 million additional people facing undernutrition in 2022.

**A debilitated first line of defence:**

With the worsening health impacts of climate change compounding other coexisting crises, populations worldwide increasingly rely on health systems as their first line of defence. However, just as the need for healthcare rises, health systems worldwide are debilitated by the effects of the COVID-19 pandemic and the energy and cost-of-living crises. Urgent action is therefore needed to strengthen health-system resilience and to prevent a rapidly escalating loss of lives and to prevent suffering in a changing climate. However, only 48 (51%) of 95 countries reported having assessed their climate change adaptation needs (indicator 2.1.1) and, even after the profound impacts of COVID-19, only 60 (63%) countries reported a high to very high implementation status for health emergency management in 2021 (indicator 2.2.4).

The scarcity of proactive adaptation is shown in the response to extreme heat. Despite the local cooling and overall health benefits of urban greenspaces, only 277 (27%) of 1038 global urban centres were at least moderately green in 2021 (indicator 2.2.3), and the number of households with air conditioning increased by 66% from 2000 to 2020, a maladaptive response that worsens the energy crisis and further increases urban heat, air pollution, and greenhouse gas emissions.

As converging crises further threaten the world’s life-supporting systems, rapid, decisive, and coherent intersectoral action is essential to protect human health from the hazards of the rapidly changing climate.

**Health at the mercy of fossil fuels:**

The year 2022 marks the 30th anniversary of the signing of the UN Framework Convention on Climate Change, in which countries agreed to prevent dangerous anthropogenic climate change and its deleterious effects on human health and welfare. However, little meaningful action has since followed. The carbon intensity of the global energy system has decreased by less than 1% since the UNFCCC was established, and global electricity generation is still dominated by fossil fuels, with renewable energy contributing to only 8·2% of the global total (indicator 3.1). Simultaneously, the total energy demand has risen by 59%, increasing energy-related emissions to a historical high in 2021. Current policies put the world on track to a catastrophic 2·7°C increase by the end of the century. Even with the commitments that countries set in the Nationally Determined Contributions (NDCs) updated up until November 2021, global emissions could be 13·7% above 2010 levels by 2030—far from the 43% decrease from current levels required to meet Paris Agreement goals and keep temperatures within the limits of adaptation.

Fossil fuel dependence is not only undermining global health through increased climate change impacts, but also affects human health and wellbeing directly, through volatile and unpredictable fossil fuel markets, frail supply chains, and geopolitical conflicts. As a result, millions of people do not have access to the energy needed to keep their homes at healthy temperatures, preserve food and medication, and meet the seventh Sustainable Development Goal (to ensure access to affordable, reliable, sustainable, and modern energy for all). Without sufficient support, access to clean energy has been particularly slow in low HDI countries, and only 1·4% of their electricity came from modern renewables (mostly wind and solar power) in 2020 (indicator 3.1). An estimated 59% of healthcare facilities in low and middle-income countries still do not have access to the reliable electricity needed to provide basic care. Meanwhile, biomass accounts for as much as 31% of the energy consumed in the domestic sector globally, mostly from traditional sources—a proportion that increases to 96% in low HDI countries (indicator 3.2). The associated burden of disease is substantial, with the air in people’s homes exceeding WHO guidelines for safe concentrations of small particulate air pollution (PM_2·5_) in 2020 by 30-fold on average in the 62 countries assessed (indicator 3.2). After 6 years of improvement, the number of people without access to electricity increased in 2020 as a result of the socioeconomic pressures of the COVID-19 pandemic. The current energy and cost-of-living crises now threaten to reverse progress toward affordable, reliable, and sustainable energy, further undermining the socioeconomic determinants of health.

Simultaneously, oil and gas companies are registering record profits, while their production strategies continue to undermine people’s lives and wellbeing. An analysis of the production strategies of 15 of the world’s largest oil and gas companies, as of February 2022, revealed they exceed their share of emissions consistent with 1·5°C of global heating (indicator 4.2.6) by 37% in 2030 and 103% in 2040, continuing to undermine efforts to deliver a low carbon, healthy, liveable future. Aggravating this situation even further, governments continue to incentivise fossil fuel production and consumption: 69 (80%) of 86 countries reviewed had net-negative carbon prices (ie, provided a net subsidy to fossil fuels) for a net total of US$400 billion in 2019, allocating amounts often comparable with or even exceeding their total health budgets (indicator 4.2.4). Simultaneously, wealthier countries failed to meet their commitment of mobilising the considerably lower sum of $100 billion annually by 2020 as agreed at the 2009 Copenhagen Accord to support climate action in “developing countries”, and climate efforts are being undercut by a profound scarcity of funding (indicator 2.1.1). The impacts of climate change on global economies, together with the recession triggered by COVID-19 and worsened by geopolitical instability, could paradoxically further reduce the willingness of countries to allocate the funds needed to enable a just climate transition.

**A health-centred response for a thriving future:**

The world is at a critical juncture. With countries facing concurrent crises, the implementation of long-term emissions-reduction policies risks being deflected or defeated by challenges wrongly perceived as more immediate. Addressing each of the concurrent crises in isolation risks alleviating one, while worsening another. Such a situation is emerging from the response to COVID-19, which has so far has not delivered the green recovery that the health community proposed, and, on the contrary, is aggravating climate change-related health risks. Less than one third of $3·11 trillion allocated to COVID-19 economic recovery is likely to reduce greenhouse gas emissions or air pollution, with the net effect likely to increase emissions. The COVID-19 pandemic affected climate action at the city level, and 239 (30%) of 798 cities reported that COVID-19 reduced financing available for climate action (indicator 2.1.3).

As countries search for alternatives to Russian oil and gas, many continue to favour the burning of fossil fuels, with some even turning back to coal. Shifts in global energy supplies threaten to increase fossil fuel production. Even if implemented as a temporary transition, these responses could reverse progress on air quality improvement, irreversibly push the world off track from meeting the commitments set out in the Paris Agreement, and guarantee a future of accelerated climate change that threatens human survival.

On the contrary, in this pivotal moment, a health-centred response to the current crises would still provide the opportunity for a low-carbon, resilient future, which not only avoids the health harms of accelerated climate change, but also delivers improved health and wellbeing through the associated co-benefits of climate action. Such response would see countries promptly shifting away from fossil fuels, reducing their dependence on fragile international oil and gas markets, and accelerating a just transition to clean energy sources. A health-centred response would reduce the likelihood of the most catastrophic climate change impacts, while improving energy security, creating an opportunity for economic recovery, and offering immediate health benefits. Improvements in air quality would help to prevent the 1·2 million deaths resulting from exposure to fossil fuel-derived ambient PM_2·5_ in 2020 alone (indicator 3.3), and a health-centred energy transition would enhance low-carbon travel and increase urban green spaces, promoting physical activity, and improving physical and mental health. In the food sector, an accelerated transition to balanced and more plant-based diets would not only help reduce the 55% of agricultural sector emissions coming from red meat and milk production (indicator 3.5.1), but also prevent up to 11·5 million diet-related deaths annually (indicator 3.5.2), and substantially reduce the risk of zoonotic diseases. These health-focused shifts would reduce the burden of communicable and non-communicable diseases, reducing the strain on overwhelmed health-care providers. Importantly, accelerating climate change adaptation would lead to more robust health systems, minimising the negative impacts of future infectious disease outbreaks and geopolitical conflicts, and restoring the first line of defence of global populations.

**Emerging glimmers of hope:**

Despite decades of insufficient action, emerging, albeit few, signs of change provide some hope that a health-centred response might be starting to emerge. Individual engagement with the health dimensions of climate change, essential to drive and enable an accelerated response, increased from 2020 to 2021 (indicator 5.2), and coverage of health and climate change in the media reached a new record high in 2021, with a 27% increase from 2020 (indicator 5.1). This engagement is also reflected by country leaders, with a record 60% of 194 countries focusing their attention on the links between climate change and health in the 2021 UN General Debate, and with 86% of national updated or new NDCs making references to health (indicator 5.4). At the city level, local authorities are progressively identifying risks of climate change on the health of their populations (indicator 2.1.3), a first step to delivering a tailored response that strengthens local health systems. Although the health sector is responsible for 5·2% of all global emissions (indicator 3.6), it has shown impressive climate leadership, and 60 countries had committed to transitioning to climate-resilient and/or low-carbon or net-zero carbon health systems as part of the COP26 Health Programme, as of July, 2022.

Signs of change are also emerging in the energy sector. Although total clean energy generation remains grossly insufficient, record high levels were reached in 2020 (indicator 3.1). Zero-carbon sources accounted for 80% of investment in electricity generation in 2021 (indicator 4.2.1), and renewable energies have reached cost parity with fossil fuel energies. As some of the highest emitting countries attempt to cut their dependence on oil and gas in response to the war in Ukraine and soaring energy prices, many are focusing on increasing renewable energy generation, raising hopes for a health-centred response. However, increased awareness and commitments should be urgently translated into action for hope to turn into reality.

**A call to action:**

After 30 years of UNFCCC negotiations, the *Lancet* Countdown indicators show that countries and companies continue to make choices that threaten the health and survival of people in every part of the world. As countries devise ways to recover from the coexisting crises, the evidence is unequivocal. At this critical juncture, an immediate, health-centred response can still secure a future in which world populations can not only survive, but thrive.

## Introduction

Because of human activity, the global mean surface temperature is 1·1°C higher than the pre-industrial average, and the past seven years were the warmest on record.^1^ Climate change is increasing the frequency and intensity of many extreme weather and weather-related events, resulting in severe damage to the natural and social systems on which health depends. The environmental changes caused by climate change are also driving shifts in the geographic range of climate-sensitive infectious diseases, affecting food and water security, worsening air quality, and damaging socioeconomic systems. While the world coped with the ongoing COVID-19 pandemic, weather events of unprecedented intensity took place in 2021 and 2022: record temperatures of nearly 50°C in British Columbia claimed 570 lives,^2^ floods in Australia, Canada, China, Malaysia, Pakistan, South Sudan, and western Europe led to thousands of deaths, hundreds of thousands of people displaced from their homes, and billions of US dollars in losses,^3,4^ and wildfires caused devastation in the USA, Greece, Algeria, and Türkiye. Yet energy-related greenhouse gas emissions rebounded to a historical record in 2021,^5^ and atmospheric CO_2_ reached its highest concentration in more than 2 million years.^6^

Existing policies put the world on track to reaching 2·4–3·5°C above pre-industrial times by 2100, and there is a 48% chance that the 1·5°C threshold proposed in the Paris Agreement will be exceeded within 5 years.^7–9^ COVID-19 recovery efforts have thus far been unable to deliver the transformation that the health community and others called for,^10^ and ongoing geopolitical conflicts make the 1·5°C threshold less likely to be met. The findings in this report show the urgency of climate action and can inform an aligned response to compounding crises, to protect the health of present and future generations (panel 1).

## Taking stock of progress on health and climate change

The *Lancet* Countdown: tracking progress on health and climate change is an international, transdisciplinary collaboration of 51 academic institutions and UN agencies, monitoring the changing health profile of climate change.^11^

The 43 indicators (panel 2) have been refined continuously for seven years, and reflect the consensus of 99 multidisciplinary researchers, the guidance of the *Lancet* Countdown’s Scientific Advisory Group and High-Level Advisory Board, and the support of *The Lancet* and the Wellcome Trust. Most indicators have been refined this year to improve the monitoring of associations between climate change and health. New and re-introduced metrics monitor the impact of extreme temperature on food insecurity; exposure to wildfire smoke; household air pollution; the alignment of the fossil fuel industry with a healthy future; and health considerations in each country’s Nationally Determined Contributions (NDCs). All new or substantially modified indicators were assessed by an independent expert panel for appropriateness and robustness,^12,13^ and some existing indicators were independently assessed to ensure continued relevance and rigour.

This report, more concise than previous iterations, is supplemented by an online data visualisation platform that can be used to see indicators in full detail and geographical resolution. Reports from the *Lancet* Countdown regional centres in Asia (Tsinghua University, China), Europe (Barcelona Supercomputing Center, Spain), South America (Universidad Peruana Cayetano Heredia, Peru), and Australia (Macquarie University and The University of Sydney) offer more detailed regional assessments than this report. Newly established centres are working to explore in further depth the association between health and climate change in Small Island Developing States (University of the West Indies, Jamaica) and Africa (Medical Research Council Unit, The Gambia). With these expanding local networks, the *Lancet* Countdown now brings together more than 250 researchers from almost 100 institutions worldwide.

As countries attempt to meet Paris Agreement commitments, *Lancet* Countdown indicators are contributing to national and international climate and health monitoring systems, and have been incorporated into the European Climate and Health Observatory and the climate and health assessment of the Italian National Institute of Health (Istituto Superiore di Sanità).^14^ In 2023, the UNFCCC will run the first Global Stocktake, an assessment of collective progress towards meeting Paris Agreement goals, designed to help countries adjust efforts to meet climate targets. Taking stock of the health impacts of climate action, this report can help countries realise the ambition of making the Paris Agreement the “most important public health agreement of the century”.^15^

## Section 1: health hazards, exposures, and impacts

Climate change is affecting the health of people worldwide directly with increased exposure to extreme weather, and indirectly with impacts on the physical, natural, and social systems on which health depends. Climatic changes are also amplifying the existing threats to food and water security, built infrastructure, essential services, and livelihoods.

Section 1 tracks the health hazards, exposures, and impacts of climate change, with indicators that monitor vulnerabilities now detailed in section 2. Indicators have been improved and expanded to provide a comprehensive overview of the effects of climate change on health^13^ and the effects of climatic and demographic changes on health-related outcomes. Three new sub-indicators track the influence of wildfires on exposure to PM_2·5_ air pollution (indicator 1.2.1), the associations between heat and extreme precipitation with online sentiment expressions (indicator 1.2.3), and the increasing impact of extreme heat on global food security (indicator 1.4.1).

### Indicator 1.1: health and heat

Climate change is leading to an increase in average global temperatures and in the frequency, intensity, and duration of heatwaves.^16^ Exposure to extreme heat is associated with acute kidney injury, heatstroke,^17^ adverse pregnancy outcomes,^18,19^ worsened sleep patterns,^20^ impacts on mental health, worsening of underlying cardiovascular and respiratory disease, and increases in non-accidental and injury-related deaths.^21^ Exposure to extreme heat also affects health indirectly by restricting people’s capacity to work and exercise.^22–26^ Older people, pregnant women, newborn babies, people who are socially deprived, and people working outdoors are particularly at risk.^27,28^

#### Indicator 1.1.1: exposure to warming—headline finding: from 2000 to 2021, populations were exposed to an average increase in summer temperature two times higher than the global mean

Inhabited land areas warm up faster than oceans. By overlapping gridded temperature and population data, this indicator shows that the average temperatures humans were exposed to during summer seasons in 2021 were 0·6°C higher than the average in 1986–2005, representing twice the global mean temperature increase in the same period (0·3°C).

#### Indicator 1.1.2: exposure of vulnerable populations to heatwaves—headline finding: in 2012–2021, children younger than 1 year experienced 600 million more person-days of heatwaves, and adults older than 65 years experienced 3·1 billion more than in 1986–2005

**Figure 1:**
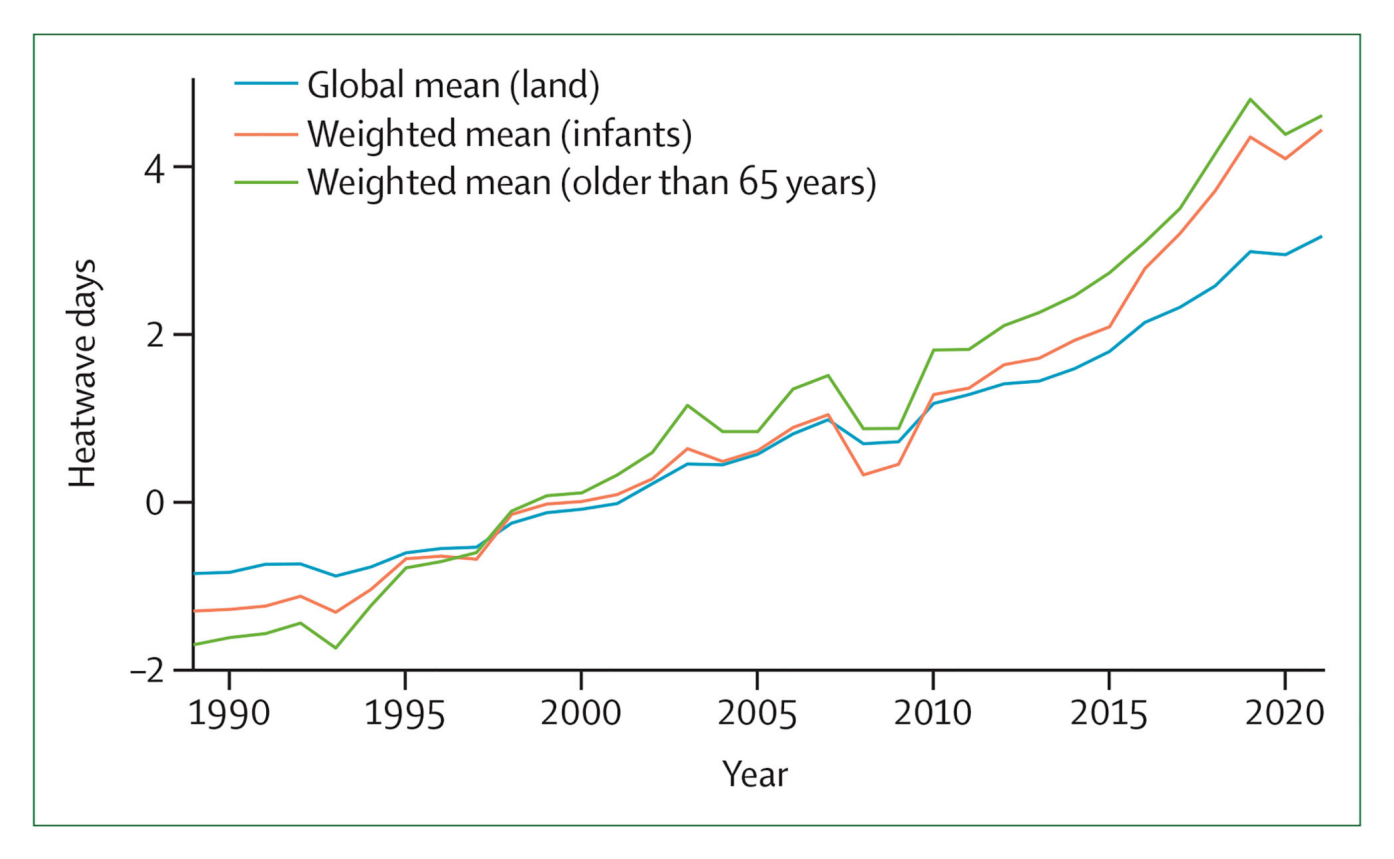
Change in heatwave days compared with the 1986–2005 baseline (10-year rolling mean) Heatwave days are presented as mean-weighted by land surface area, mean-weighted by infant population, and mean-weighted by the population older than 65 years.

Between 2021 and 2022, record temperatures were registered in Oman, the Middle East,^29^ Australia,^30^ many Mediterranean countries, and Canada.^31^ This indicator overlaps daily temperature and demographic data to track the exposure of vulnerable age groups to heatwaves (a period of 2 or more days in which both the minimum and maximum temperatures are higher than the 95th percentile of temperatures in 1986–2005, as defined previously in the literature).^32,33^ During the period 2012–21, children younger than 1 year experienced 600 million more person-days of heatwaves (4·4 more days per child) annually compared with the average in 1986–2005, and adults older than 65 years experienced 3·1 billion more days (3·2 more days per person; figure 1). In 2021, people older than 65 years in Canada experienced a record of 47 million more person-days of heatwaves (2·4 million in children under 1 year) than annually in 1986–2005, mainly due to an unprecedented heatwave that was at least 150 times more likely to have occurred because of climate change (panel 3).

#### Indicator 1.1.3: heat and physical activity—headline finding: over the past 10 years, high heat posed at least a moderate heat stress risk during light outdoor physical activity, for an average additional 281 hours on per person per year, compared with 1991–2000

Regular physical activity contributes to a healthy body-weight, improves physical and mental health,^59–61^ and helps to prevent many non-communicable diseases.^62^ However, hot weather reduces the likelihood of engaging in exercise, and increases the risk of heat illness when it is done.^22–24^ This indicator has been improved to track the daily hours during which physical activity would entail heat stress risk.^63^ Compared with the baseline average in 1991–2000, the number of annual hours of moderate-risk of heat stress during light outdoor physical activity increased globally in 2012–21 by an average of 281 (33% increase) hours per person and high-risk heat stress increased by 238 (42%) hours per person. The greatest rise occurred in medium HDI countries, with an increase in the hours of moderate risk of heat stress during light outdoor physical activity of 310 (20%) hours per person and an increase of high-risk of 296 (26%) hours per person, annually.

#### Indicator 1.1.4: change in labour capacity—headline finding: in 2021, heat exposure led to the loss of 470 billion potential labour hours, a 37% increase from the period 1990–99. 87% of the losses in low HDI countries were in the agricultural sector

**Figure 2:**
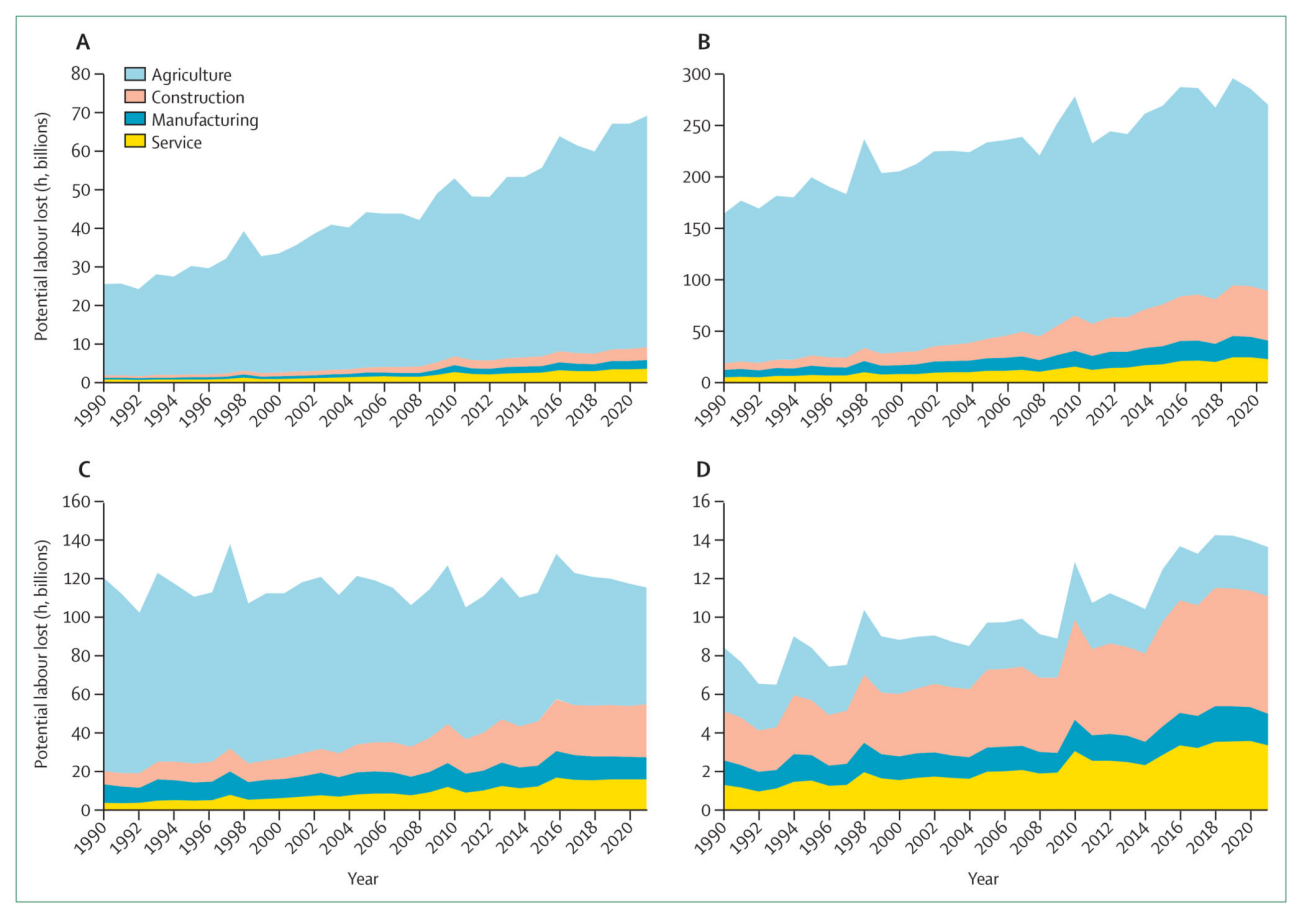
Potential labour lost because of heat-related factors in each sector, assuming all work is done in sun exposure Low HDI (A), medium HDI (B), high HDI (C), and very high HDI (D) groups (2019 HDI country group). HDI=human development index.

Heat exposure affects labour productivity and puts the health of exposed workers at risk. The resulting labour loss undermines livelihoods and the socioeconomic determinants of health.^64^ This indicator monitors the potential work hours lost because of heat exposure and solar radiation, in an improvement from previous reports, by associating wet bulb globe temperature with the typical metabolic rate of workers in specific economic sectors. Since 1999, the potential hours lost increased by 5·6 billion hours per year (figure 2). In 2021, 470 billion hours were lost—an increase of 37% from the annual average in 1990–99, and an average of 139 hours lost per person. Two thirds of all labour hours lost globally in 2021 were in the agricultural sector. This proportion was highest in low HDI countries, at 87%.

#### Indicator 1.1.5: heat-related mortality—headline finding: heat-related mortality for people older than 65 years increased by approximately 68% between 2000–04 and 2017–21

A study of 43 countries published in May, 2021, estimated that 37% of heat-related deaths are attributable to human-induced climate change.^65^ However, insufficient data sharing and reporting restrict the capacity to produce accurate estimates globally, to assess adaptation measures, and to identify vulnerable populations.^11,13^ Using a generalised exposure–response function to provide an estimate of heat-related deaths globally, this indicator finds that annual heat-related mortality of people older than 65 years increased by an estimated 68% between 2000–04 and 2017–21.

### Indicator 1.2: health and extreme weather events

Detection and attribution studies show the increasing influence of anthropogenic climate change on weather extremes (panel 3).^66^ Resulting direct injuries and death are often compounded with impacts on sanitation and service provision, forced displacement, loss of assets and infrastructure, economic losses, and adverse mental health outcomes, often having long-lasting effects.^67–70^ This group of indicators, complemented by indicators 2.3.2 and 4.1.1, details the association between climate change, extreme weather events, and health.

#### Indicator 1.2.1: wildfires—headline finding: human exposure to days of very-high or extremely-high fire danger increased in 61% of countries from 2001–04 to 2018–21

Wildfires affect health with thermal injuries, exposure to wildfire smoke, loss of physical infrastructure, and impacts on mental health and wellbeing.^71–73^ Drier and hotter conditions increasingly favour the occurrence, intensity, and spread of wildfires, and undermine control efforts.^74^ This indicator uses remote sensing to track exposure to days of high meteorological wildfire danger and wildfire exposure, with improved consideration of cloud cover in the detection of wildfire spots in this year’s report. New to this report, the indicator incorporates atmospheric modelling (IS4FIRES-SILAM model) to track exposure to wildfire smoke (PM_2·5_).^75,76^

**Figure 3:**
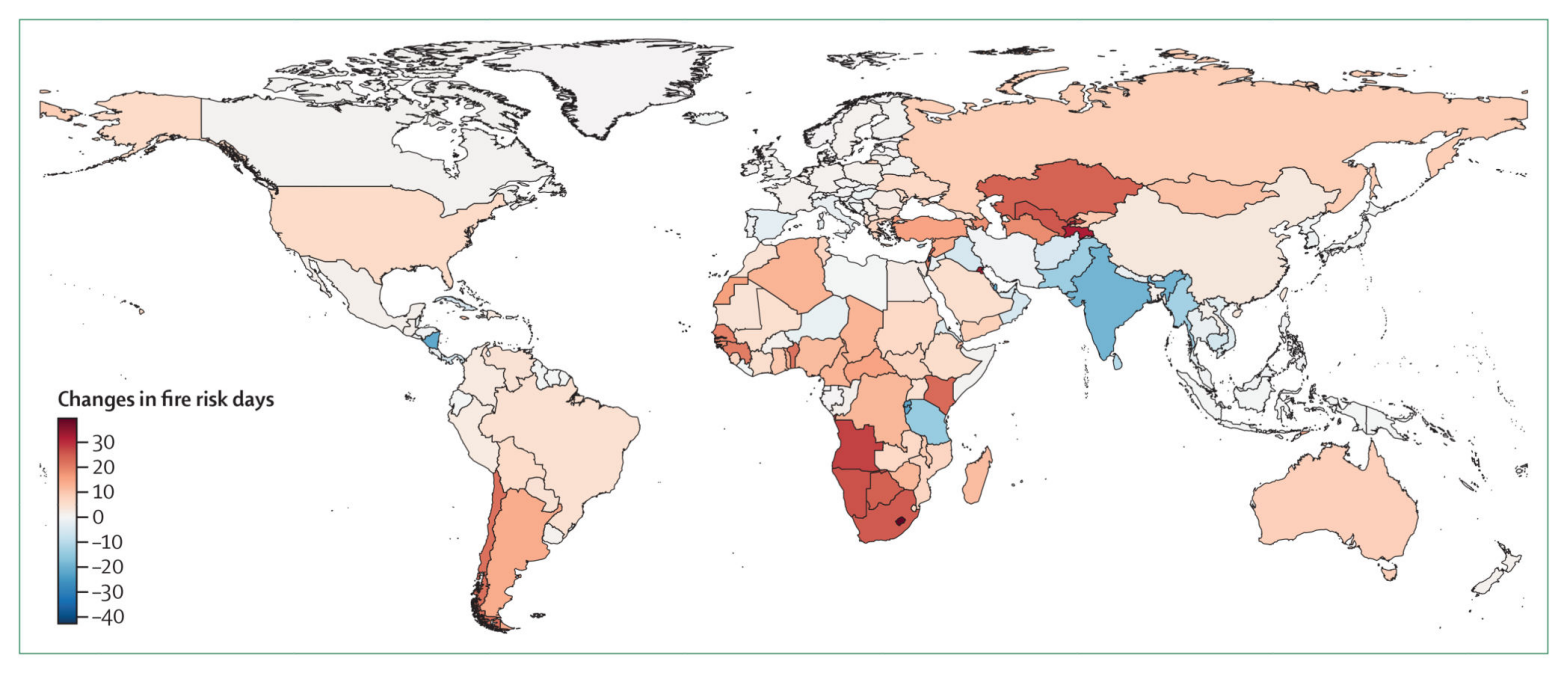
Population-weighted mean changes in extremely-high and very-high fire danger days in 2018–21 compared with 2001–04 Large urban areas with a population density of 400 people or more per km^2^ are excluded.

Globally, people experienced an average of nine more days of very-high or extremely-high meteorological wildfire danger in 2018–21 compared with 2001–04, with 110 (61%) of 181 countries having an increase (figure 3)—a pattern caused by climate variation rather than demographic shifts. The yearly average wildfire exposure increased by 9·17 million person-days between 2003–06 and 2018–21. Increases were observed in 21 (64%) of 33 low HDI countries compared with 27 (42%) of 65 very high HDI countries, which could reflect differences in wildfire prevention and management.

Population exposure to wildfire-derived PM_2·5_ was modelled with the SILAM chemistry transport model.^77^ Data show a statistically significant increase in 16·5% of the global land surface from 2003 to 2021, and a significant decrease in 8·8% of the surface land area.

#### Indicator 1.2.2: drought—headline finding: on average, 29% more global land area was affected by extreme drought for at least one month in a year in 2012–21 than in 1951–60

Droughts put food and water security at risk, threaten sanitation, affect livelihoods, and increase the risk of wildfires and infectious disease transmission.^66,78^ This indicator uses the 6-monthly Standard Precipitation and Evapotranspiration Index (SPEI6) to capture changes in extreme drought (SPEI ≤–1·6) due to changes in precipitation and temperature-driven evapotranspiration.^79^ In the period 2012–21, on average, almost 47% of global land area was affected by at least 1 month of extreme drought each year, an increase of 29% from the period 1951–60. The Middle East and north Africa, where 41 million people lack access to safe water and 66 million do not have basic sanitation services,^80^ was particularly affected, with some areas experiencing more than 10 extra months of extreme drought.

#### Indicator 1.2.3: extreme weather and sentiment—headline finding: heatwaves during 2021 were associated with a statistically significant decrease of 0·20 percentage points in the number of tweets expressing positive sentiment, whereas extreme precipitation days were associated with a statistically significant decrease of 0·26 percentage points

Heatwaves and extreme weather increase the risk of mental health disorders (panel 4).^21,81,82^ This indicator uses a multivariate ordinary least squares fixed effects model to monitor the influence of heatwaves on online sentiment expression (measured here as the sentiment expressed in tweets), in addition to the effect of extreme precipitation, which is new to this year’s report.^111^ This model was used to analyse 7·7 billion tweets from 190 countries and adjusts by month, calendar date, and location. Days of extreme precipitation during 2021 reduced the percentage of tweets that had positive expression by a statistically significant 0·26 percentage points, the highest recorded reduction in positive expression during extreme precipitation days since 2015. Since 2015, heatwave days and days of extreme precipitation have consistently worsened sentiment expression. In 2021, heatwave days increased the proportion of tweets that expressed negative sentiment by a statistically significant 0·20 percentage points, the largest effect in the historical series (from 2015 to 2021). The heatwave in the Pacific Northwest, in 2021, increased negative sentiment by 9·8 times and decreased positive sentiment by 3·7 times the average effects of heatwaves in 2015–20. The extreme rainfall events in western Europe, in 2021, increased negative sentiment by 4·9 times and decreased positive sentiment by 6·6 times the average effect of extreme precipitation on sentiment in 2015–20.

### Indicator 1.3: climate suitability for infectious disease transmission—headline finding: the climatic suitability for the transmission of dengue increased by 11·5% for *Aedes aegypti* and 12·0% for *Aedes albopictus* from 1951–60 to 2012–21; the length of the transmission season for malaria increased by 31·3% in the highlands of the Americas and 13·8% in the highlands of Africa from 1951–60 to 2012–21

Climate change is affecting the distribution and transmission of many infectious diseases, including vector-borne, food-borne, and waterborne diseases.^112–114^ This indicator monitors the influence of the changing climate on the potential for transmission for key infectious diseases that are a public health concern.

**Figure 4:**
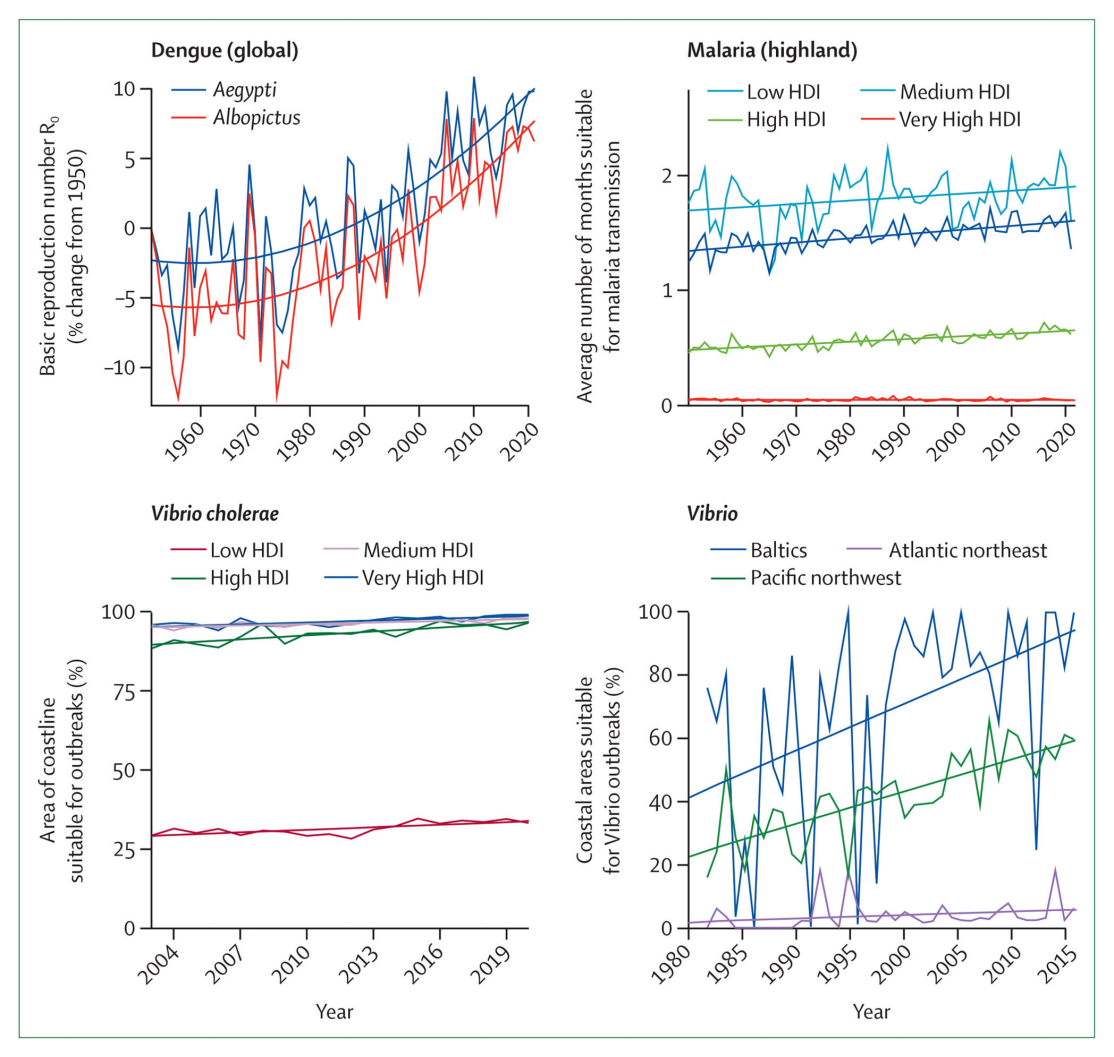
Change in climate suitability for infectious diseases Thin lines show the annual change. Thick lines show the trend since 1951 (for malaria), 1951 (for dengue), 1982 (for *Vibrio* bacteria), and 2003 (for *Vibrio cholerae*). HDI=human development index.

With the increased movement of people and goods, urbanisation, and climate change, *Aedes*-transmitted arboviruses spread rapidly in the past two decades, and half the world population now lives in countries where dengue is present.^115–117^ Combining data on temperature, rainfall, and population, this indicator tracks the basic reproduction number (R0) for dengue, Zika, and chikungunya as a proxy for their transmissibility and, new to this report, the number of months suitable for their transmission. On average, during 2012–21, the R0 increased by 11·5% for the transmission of dengue by *Aedes aegypti* and 12·0% for the transmission of dengue by *Aedes albopictus*; and 12·0% for the transmission of chikungunya by *A albopictus*, and 12·4% for the transmission of Zika by *A aegypti* compared with 1951–60, globally (figure 4). During the same period, the length of the transmission season increased for all arboviruses by approximately 6%.

The number of months suitable for the transmission of *Plasmodium falciparum* by *Anopheles* mosquitoes was computed with temperature, precipitation, and humidity thresholds, and, new to this year’s report, land classes (appendix 5 p 46) suitable for the vector. The number of suitable months in highland areas (≥1500 m above sea level) increased by 31·3% in the WHO region of the Americas, and 13·8% in Africa between 1951–60 and 2012–21.

Non-cholera *Vibrio* bacteria survive in brackish waters, and can cause gastroenteritis if ingested in contaminated food, and potentially lethal wound infections if direct contact is made with contaminated water.^118^ Between 2014–21 and 1982–89, because of changes in sea-salt concentrations and temperature, the area of coastline suitable for *Vibrio* pathogens increased from 47·5% to 86·3% in the Baltic; from 30·0% to 57·1% in the US northeast; and from 1·2% to 5·7% in the Pacific Northwest; three regions where *Vibriosis* is regularly reported. An extra 4·3% of the coastal waters in northern latitudes (40–70°N) had temperatures suitable for *Vibrio* during 2014–21 compared with 1982–89, with 2021 being the second most suitable year on record (11·3% of the coastal area suitable), making brackish waters in these latitudes increasingly suitable for *Vibrio* transmission.

The ongoing seventh cholera pandemic, which started in the 1960s, is responsible for more than 2·8 million cholera cases and 95 000 deaths annually.^119,120^ Although inadequate sanitation is the main enabler, climate conditions are increasingly favouring the survival of *Vibrio cholerae* in natural waters, keeping an environmental reservoir and favouring its spread.^114^ Deploying an ecological niche model, this indicator estimates that an additional 3·5% of global coastal waters have become suitable for the transmission of cholera since 2003–05.

### Indicator 1.4: food security and undernutrition—headline finding: compared with 1981–2010, increased temperatures in 2021 shortened crop growth seasons globally by 9·3 days for maize, 1·7 days for rice, and 6·0 days for winter and spring wheat, and heatwave days were associated with 98 million more people reporting moderate to severe food insecurity in 2020

Food insecurity is increasing globally, with 720–811 million people hungry in 2020. Climate change is exacerbating risks of malnutrition via multiple and interconnected mechanisms (panel 5). Less-educated and lower-income households have an increased chance of experiencing food insecurity,^121^ and due to social roles and reduced land ownership, women, and the households they lead, might be more prone to malnutrition.^122–124^

High temperatures during growing seasons lead to fast crop maturation, which reduces the maximum potential yield that could be achieved with no limitations of water or nutrients. Combining temperature and crop growth data, the first part of this indicator shows that, compared with the average during 1981–2010, average crop growth season lengths in 2021 continue to shorten globally for all staple crops tracked: by 9·3 days for maize, 1·7 days for rice, and more than 6·0 days for winter and spring wheat.

Rising atmospheric CO_2_ concentrations are also increasing sea surface temperature, temperatures of inland water bodies, acidifying oceans, and reducing their oxygenation, which exacerbates coral reef bleaching and affects marine and inland fishery productivity.^125–129^ With a shift to farm-based fish products of reduced nutritional quality, climate change is thus putting marine food security at risk.^130–132^ The average sea surface temperature in coastal waters of 142 countries increased globally by nearly 0·7°C in 2019–21 compared with 1980–82.

**Figure 5:**
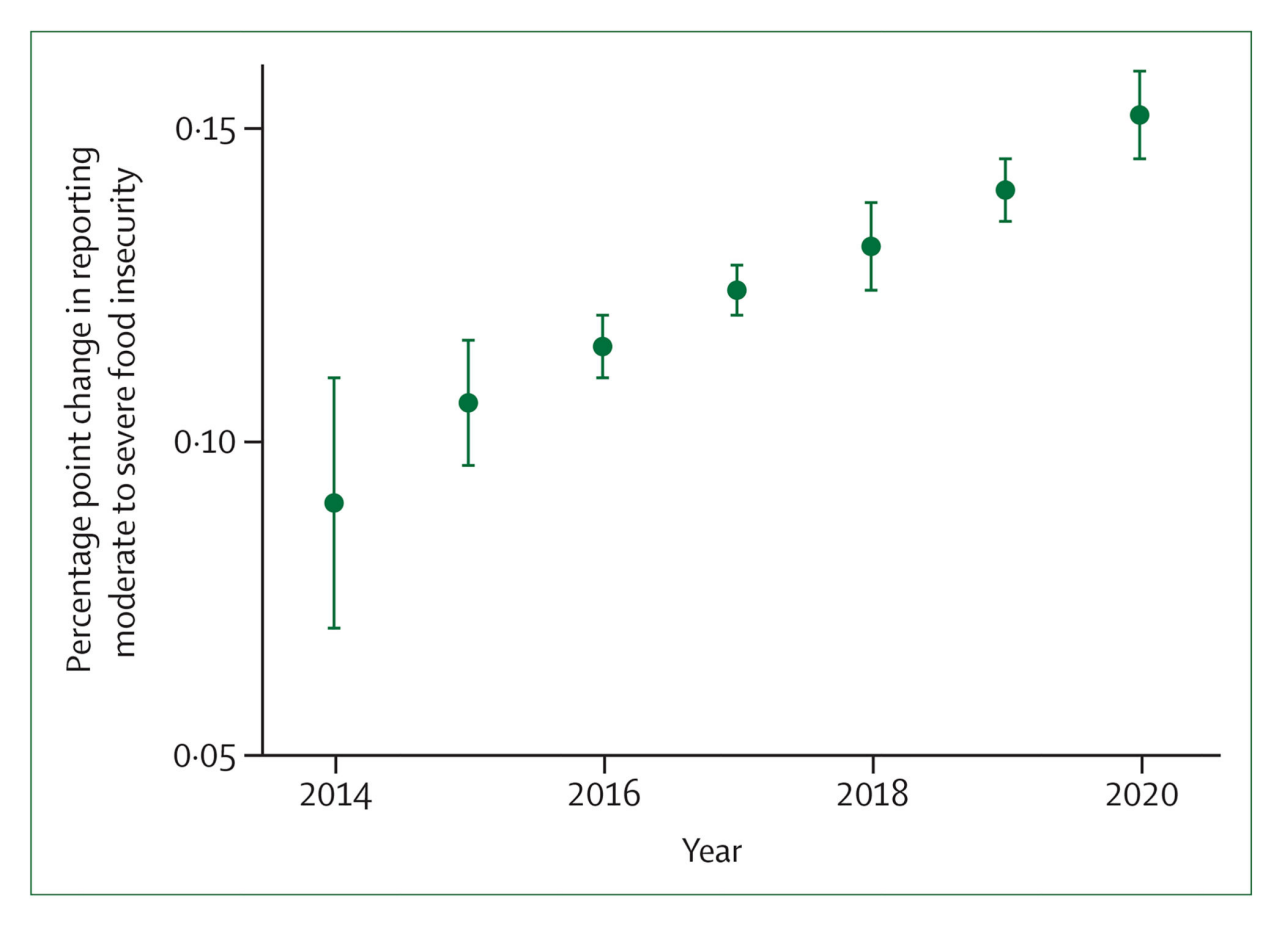
Change in the percentage of people reporting moderate to severe food insecurity because of heatwave days occurring during major crop growing seasons Heatwave days are shown as a percentage point change. Major crop seasons were maize, rice, sorghum, and wheat.

New to this year’s report, the third part of this indicator examines the impact of heatwave days during crop growth season of maize, rice, sorghum, and wheat, on self-reported experience of food insecurity. The indicator combines data from the Food and Agriculture Organization of the UN Food Insecurity Experience Scale from 103 countries with temperature, using a time-varying regression.^133,134^ Compared with 1981–2010, increases in the number of heatwave days resulted in an increase of 3·7 percentage points in self-reported moderate to severe food insecurity in 2020, approximately equivalent to an additional 98 million people reporting moderate or severe food insecurity (figure 5; panel 5).

## Conclusion

With an average global surface heating of 1·1°C, climate change is increasingly affecting mental and physical health. Changing climatic conditions are increasing the risk of heat-related illness (indicators 1.1.1–1.1.5), changing the pattern of infectious disease transmission (indicator 1.3), increasing health risks from extreme events (indicators 1.2.1–1.2.3), putting sanitation at risk, and having multidimensional impacts on food and water security (indicator 1.3 and panel 5). These impacts often occur simultaneously, exacerbating the pressure on health and health-supporting systems, and potentially triggering cascading impacts on the social and natural systems that good health depends upon.

With the world projected to heat by 2·4–3·5°C by 2100, this section details the urgency of accelerating mitigation and adaptation to prevent the devastating health outcomes of a heating world.

## Section 2: adaptation, planning, and resilience for health

With rapidly increasing climate change-related health hazards, transformative, proactive, and effective adaptation measures are immediately required to manage the health threats of unavoidable global heating, reducing exposure and vulnerabilities, and increasing resilience.^66^ Because of the interconnected and multifactorial nature of health determinants and climate impacts, adaptation should be integrated across sectors, and into policies and programmes in health systems, governments, and private corporations.^66^

Three groups of indicators are presented in this section. Indicators 2.1.1–2.1.2 detail adaptation plans and risk and vulnerability assessments—key first steps in health adaptation. The implementation of health adaptation measures and their financing are detailed in indicators 2.2.1–2.2.5. The final set of indicators, shown in section 1 in previous *Lancet* Countdown reports, have been improved to assess population vulnerabilities, resilience and adaptation interventions, and the risks associated with changing climate hazards (indicators 2.3.1–2.3.3).

### Indicator 2.1: assessment and planning of health adaptation

Evidence-based policy making requires comprehensive evaluation of the health threats of climate change. Climate change and health risk vulnerability and adaptation assessments identify vulnerable populations, assess the influence of existing policies, programmes, and health systems capacities in building resilience, and identify future adaptation needs. These indicators monitor the extent to which such assessments are being done, and the contribution of assessments in informing adaptation plans that can protect populations from climate-related health impacts.

#### Indicator 2.1.1: national assessments of climate change impacts, vulnerability, and adaptation for health—headline finding: in 2021, 48 (51%) of 95 countries reported having completed a climate change and health vulnerability and adaptation assessment, but these only strongly influenced resource allocation in nine countries

With data from the 2021 WHO Health and Climate Change Global Survey,^165^ this indicator monitors whether countries have completed a health vulnerability and adaptation assessment. Although 48 (51%) of 95 countries reported completing an assessment, only nine reported that its findings strongly influenced the allocation of human and financial resources to address health risks of climate change, and only 18 reported that assessments strongly informed the development of health policies and programmes (appendix 5 p 68).

#### Indicator 2.1.2: national adaptation plans for health—headline finding: 49 (52%) of 95 countries reported having a national health and climate change plan in place in 2021

This indicator monitors whether countries have a national health and climate change plan in place, based on data from the 2021 WHO Health and Climate Change Global Survey.^165^ Only about half of countries (49/95) reported having a national health and climate change plan in place. Of these countries, 62 (65%) indicated a moderate or lower level of implementation, with 67 (70%) countries citing insufficient finance as a main barrier. As part of the new COP26 Health Programme Initiative on Climate Resilient Health Systems,^166^ 59 countries committed to conducting a vulnerability and adaptation assessment and using the findings to inform the development of a Health National Adaptation Plan, which contributes to the UNFCCC’s National Adaptation Plan process. Implementing commitments to the COP26 Health Programme will strengthen access to climate finance, inform national roadmaps for investments in climate-resilient and sustainable health systems, and support the implementation of critical health adaptation interventions.

#### Indicator 2.1.3: city-level climate change risk assessments—headline finding: 725 (78%) of 930 cities reporting to CDP’s global survey had completed or were in the process of undertaking city-level climate change risk assessments

More than half of the world’s population live in cities;^167^ with local interventions, cities are crucial for adaptation to climate change. With data reported to CDP,^168^ this indicator shows that, in the past 5 years, the number of cities that declared having undertaken climate assessments increased from 205 (46%) of 449 respondents in 2016, to 725 (78%) of 930 respondents in 2021, reflecting an increased recognition of the city-level impacts of climate change. Although 849 (91%) of the 930 cities responding to this question were in very high or high HDI countries, responding cities from low and medium HDI countries increased by 70%, from 24 (5%) of 471 in 2020, to 41 (8%) of 522 in 2021. 530 (64%) of 822 cities reported that climate change threatened public health or health services. In a shift from last year’s reporting, infectious diseases were identified as the most prominent climate-related health hazard (identified by 382 cities), followed by heatwaves (339 cities) and poor air quality (267 cities). The COVID-19 pandemic affected climate action at the city level, with 310 (39%) of 805 cities reporting that the pandemic increased emphasis on climate action, and only 116 (14%) reporting that the pandemic decreased this emphasis. However, 242 (30%) of 798 cities reported that COVID-19 reduced financing available for climate change, whereas only 178 (22%) reported an increase in financing.

### Indicator 2.2: enabling conditions, adaptation delivery, and implementation

Interventions in health-related sectors can reduce climate-related exposure, vulnerability, and hazards, minimising risks to health and wellbeing.^66^ Interventions should be integrated across sectors, and include health system strengthening, capacity building, behaviour change, early warning systems, physical infrastructure, and climate-smart agriculture, with adequate financing essential to their implementation. Indicators in this section track progress on implementing these interventions.

#### Indicator 2.2.1: climate information for health—headline finding: in 2021, less than 40% of countries had climate-informed health surveillance systems in place for vector-borne, waterborne, or airborne diseases

Building preparedness and delivering an adequate response to climate hazards requires health systems to have access to, and use, climate information. This indicator uses data from the 2021 WHO Health and Climate Change Global Survey, to monitor the use of climate information for health surveillance and early warning systems.^165^

In 2021, 30 (39%) of 78 countries reported having climate-informed health surveillance systems for vector-borne diseases, 25 (32%) for waterborne diseases, 23 (35%) of 65 countries for airborne diseases, and 14 (21%) of 66 countries for zoonoses. However, only six (13%) of 47 countries had surveillance for mental health risks and eight (11%) of 70 countries had surveillance for food-borne diseases.

As extreme weather intensifies, climate-informed health early warning systems can help to restrict and respond to its health impacts. About a third (28) of 84 countries reported having climate-informed health early warning systems in place for heat-related events and 26 (30%) of 86 countries reported having them in place for other extreme weather events. Half (n=13) of the 26 very high HDI countries had health early warning systems for extreme weather events compared with only six (19%) of 31 low or medium HDI countries. Whereas 16 (64%) of the 25 very high HDI countries had climate-informed health early warning systems for heat-related events, only four (13%) of 30 low or medium HDI countries had them in place.

#### Indicator 2.2.2: air conditioning: benefits and harms—headline finding: despite helping to prevent heat-related illness, air conditioning was also responsible for 0·9 gigatonnes of CO_2_ emissions and 24 000 deaths attributable to PM_2·5_ exposure in 2020

Although air conditioning is effective at protecting against heat-related illness,^169^ 1·8–4·1 billion people in LMICs exposed to heat stress do not have any indoor cooling, and air conditioning is often unaffordable in these countries.^170,171^ Air conditioning also contributes to greenhouse gas emissions, air pollution, urban heat island effects, power outages, and energy poverty.^172–176^ With data from the International Energy Agency (IEA),^177^ this indicator reports that about a third of households globally had air conditioning in 2020, an increase of 66% from 2000. The use of air conditioning in 2020 was responsible for 0·9 gigatonnes (Gt) of CO_2_ emissions and 24 000 deaths from PM_2·5_ exposure. Sustainable cooling alternatives need to be rolled out rapidly to avoid the worst health impacts from rising temperatures (panel 6).

#### Indicator 2.2.3: urban green space—headline finding: in 2021, only 27% of urban centres were classified as moderately green or more

**Figure 6:**
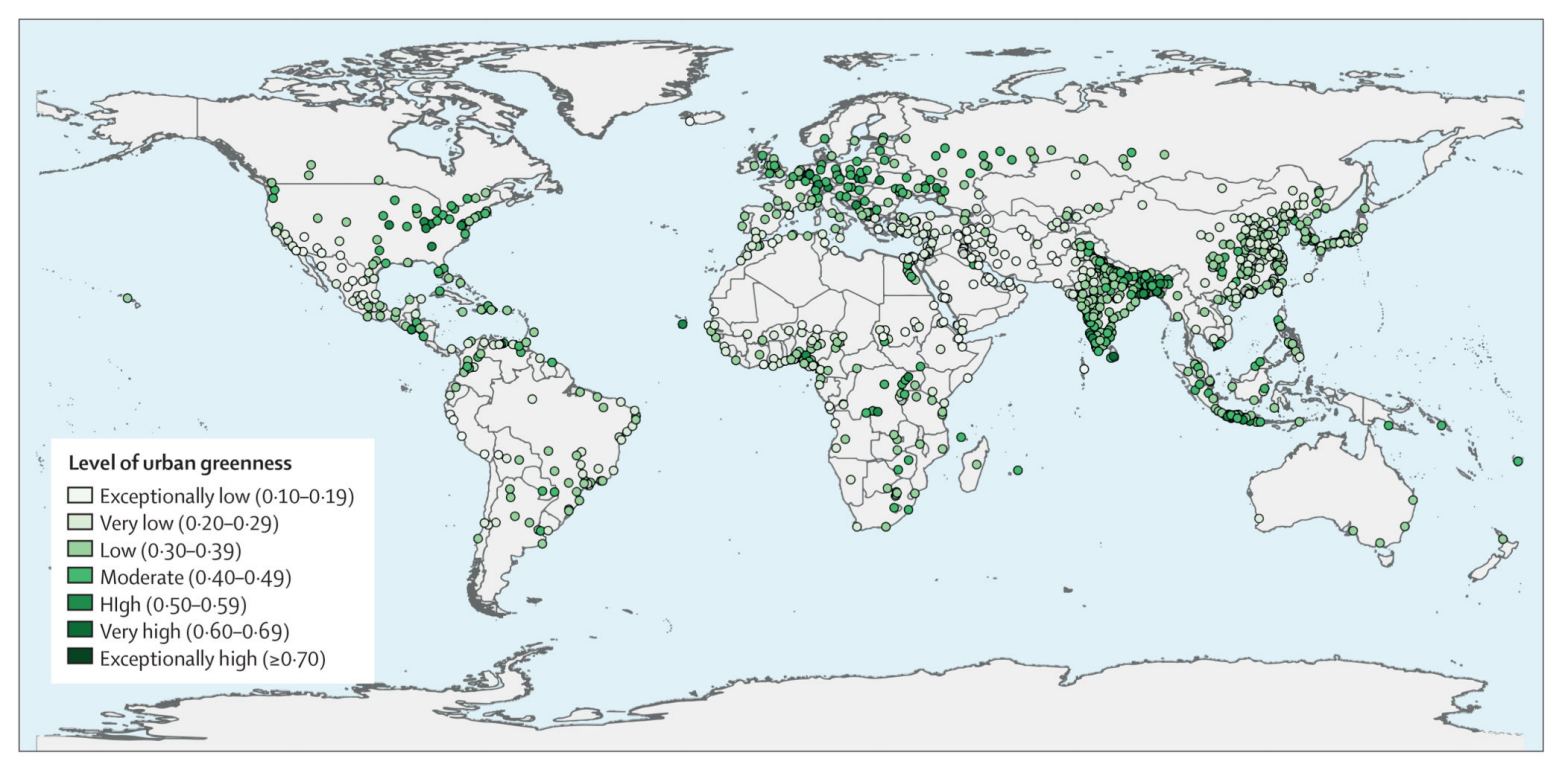
Level of urban greenness in urban centres with more than 500 000 inhabitants in 2021 The numbers in brackets show the population-weighted NDVI level, which is used as a measure of urban greenness. NDVI=normalised difference vegetation index.

Nature-based solutions can contribute to climate change adaptation and have ecosystem benefits.^66^ Green spaces reduce urban heat islands, positively affect physical and mental health, and provide adaptation to extreme heat.^193–195^ This indicator reports population-weighted normalised difference vegetation index (NDVI) as a proxy for green space exposure in the 1038 urban centres that have over 500 000 inhabitants. Despite increasingly extreme heat, average global exposure to urban green space has remained consistently low since 2015 (mean NDVI 0·34), and just 27% (278 out of 1038) of urban centres were moderately-green or above in 2021 (figure 6; appendix 5 p 79). Only 33% of cities in very high HDI countries, and 39% of those in medium HDI countries, had at least moderate levels of greenness; a proportion that is even lower in high and low HDI countries (16% for both).

#### Indicator 2.2.4: health adaptation-related funding—headline finding: only 15% of $1·14 billion under the Green Climate Fund went towards adaptation activities with health benefits in 2021

Financial resources are essential to implementation health adaptation interventions.^66^ This indicator uses transactional data from Kmatrix’s Adaptation and Resilience to Climate Change dataset to monitor global spending, with the potential to support adaptation in health-care sectors and in sectors of health relevance (eg, agriculture, water, and built environment). In the fiscal year 2020–21, US$21·78 billion was spent in transactions that could support health and health-care adaptation (5·6% of total adaptation-related spending), and $111·2 billion (28·5%) was spent in transactions with the potential to deliver adaptation in health-relevant sectors. In a reversal of trends from previous years, the share of spending in these two sectors with respect to total adaptation-related spending decreased slightly (by less than 0·1%).

The second part of this indicator monitors global multilateral funding for health-related adaptation projects by the Green Climate Fund. In 2021, the Green Climate Fund approved $726 million for 15 adaptation projects and $414 million for eight mitigation and adaptation projects. Of the approved funding, only 15% ($166 million) went to projects with benefits that included increased resilience of health and wellbeing. Furthermore, of the 54 concept notes submitted for adaptation and cross-cutting projects ($1·6 billion), only four focused on health systems ($218 million), none of which were approved. These findings highlight a deficit in the prioritisation of health within adaptation funding.

#### Indicator 2.2.5: detection, preparedness, and response to health emergencies—headline finding: only 112 (63%) of 177 countries reported high to very high implementation status for health emergency management in 2021

This indicator monitors implementation of core capacity 7 (health emergency management) of the International Health Regulations (IHR). With small changes from previous years, emergency management under core capacity 7 is now comprised of three capacity requirements: planning for health emergencies, management of health emergency response, and emergency logistic and supply chain management. In 2021, 112 (63%) of 177 countries reported high to very high implementation (capacity score of 61–100) of health emergency management. Considering HDI, large disparities existed, with only 35% of low or medium HDI countries reporting high to very high implementation status of health emergency management compared with 88% of very high HDI countries.

The COVID-19 pandemic prompted a review of the IHR by the World Health Assembly in 2020.^196,197^ Proposed reforms include regular country reviews and monitoring mechanisms, increased support for their implementation, and improved information sharing, all of which can help strengthen health systems from health hazards related to climate change. Climate change emergency preparedness and response requires a multisectoral approach with strengthened leadership and coordination of international financial and health institutions, and increased ability to address public health misinformation. These preparedness and response measures would deliver subsequent benefits across the whole health system.^196,198^

### Indicator 2.3: vulnerabilities, health risk, and resilience to climate change

Climate change adaptation aims to reduce human exposure and vulnerability to climate hazards, minimising health risks, and ultimately minimising climate change-related health impacts. These indicators provide an insight into the effectiveness of adaptation and health system strengthening in modifying climate-related health risks.

#### Indicator 2.3.1: vulnerability to mosquito-borne diseases—headline finding: improvements in health care contributed to a 43% decrease in vulnerability to severe dengue outcomes in low HDI countries from 1990 to 2019, whereas urbanisation increased vulnerability by 5% in very high HDI countries

Dengue incidence increased eight-fold in the past two decades, driven by population movement, international trade, urbanisation, and increasing climatic suitability (indicator 1.3).^115–17,199,200^ Although controlling the spread is challenging,^201^ timely and adequate treatment is essential to prevent severe health outcomes.^66,202,203^ This indicator tracks the relative vulnerability to severe adverse dengue outcomes in countries that have the suitable climatic conditions for dengue outbreaks (R0>1, as per indicator 1.3), combining two main determinants of dengue vulnerability: health-care access and quality (by using mortality from key preventable diseases as a proxy), and the proportion of population in urban environments.^199,204^ Between 1990 and 2019, improvements in health care contributed to a 43% reduction in vulnerability to severe dengue outcomes in low HDI countries, and a 23% reduction in medium HDI countries. However, urbanisation increased vulnerability to dengue in very high HDI countries by 5%.

#### Indicator 2.3.2: lethality of extreme weather events—headline finding: the average lethality per climate-related disaster has decreased from 837 deaths in 1980–89 to 46 in 2012–21, and is negatively associated with health-care spending

The number of reported climate and weather-related disasters has increased five-fold in the past 50 years.^205^ Using data from the Centre for Research on the Epidemiology of Disasters,^206^ data in this indicator show that the proportion of all climate-related events that were deadly has increased steadily since at least 1980. However, the lethality of these events has decreased globally from an average of 837 deaths per event in 1980–89 to 46 in 2012–21 (p<0·031). The average number of people affected per disaster is negatively correlated with GDP, HDI, and the percentage of GDP spent on health care, with the percentage of GDP showing the strongest correlation. With extreme weather events becoming increasingly frequent and severe, these results highlight the importance of strengthening health systems, including with the implementation of the priorities outlined in the Sendai Framework for Disaster Risk Reduction.^207^ Because of the socially defined gender differences in the impacts and response of extreme events, a gender-sensitive approach is particularly needed.^208^

#### Indicator 2.3.3: migration, displacement, and rising sea levels—headline finding: 149·6 million people were settled less than 1 metre above current sea level, in regions increasingly at risk from the hazards of the rising seas in 2021

The global mean sea level increased by 3·7 mm per year between 2006 and 2018, and will reach 0·28–1·01 m or more by 2100, depending on climate change mitigation efforts, ice sheet collapse, and local factors.^73,209–12^ With land elevation and population data, this indicator reports that there were 149·6 million people living less than 1 m above sea level in 2020, a slight increase from the 145·2 million people settled there in 2010. These populations are at risk of flooding, coastal and riverbank erosion, severe storms, soil and water salinisation, spread of infectious diseases, and permanent inundation.^212–14^ With insufficient adaptation, human relocation (forced, or as a proactive adaptation measure) could be a response, and its health impacts will largely depend on the support given to migrant populations.^66^ The development of policies to protect the health of migrant and immobile populations is crucial. As of December, 2021, 45 policies connecting climate change and migration were identified in 37 countries.

## Conclusion

The indicators in this section exhibit some signs of progress in the adaptation to climate change, with national and city-level assessment of the climate-related health risks gradually increasing, and evidence suggesting that the strengthening of health systems might have reduced the impact of extreme events. However, data show that the pace and scale of climate change adaptation, planning, and resilience is far from what is necessary to reduce the health impacts of climate change. Despite increasing temperatures, only 27% of urban centres have at least a moderate level of greenness, and just 28 (33%) of 84 countries report having heat-related early warning systems for health. Funding to support health adaptation remains grossly insufficient and is seldom influenced by vulnerability and adaptation assessments. In 2022, unprecedented global health, economic, and conflict events have critically worsened public health, with climate change exacerbating the impacts of many of these events. Without global coordination, transparency, and cooperation between governments, communities, civil society, businesses, and public health leaders, the world will remain vulnerable to international emergencies. The gap between the health impacts of climate change, and adaptation investment and implementation continues to increase, to the detriment of all.

## Section 3: mitigation actions and health co-benefits

Due to COVID-19-related responses, anthropogenic CO_2_ emissions decreased by 5·4% in 2020, the largest decrease in the past 25 years.^215^ However, with little structural change to limit fossil fuel use, emissions rebounded in 2021, by 6%, reaching an all-time high.^216^ The current 1·1°C of warming proved to be already dangerous to health (section 1). To limit the temperature rise to 1·5°C higher than pre-industrial levels, emissions should decrease by 45% from 2010 levels by 2030. However, even if commitments in every country’s NDCs were met, emissions in 2030 would be 13·7% higher than 2010 levels.^217^ The grossly insufficient decarbonisation, compounded by geopolitical conflict, has made it vastly more challenging to limit the temperature rise to 1·5°C, and the window of opportunity to limit the temperature rise is rapidly closing.^8^

Accelerated decarbonisation would not only prevent the most catastrophic health impacts of accelerated heating, but, if designed to maximise health benefits, could also save millions of lives with healthier diets, more active lifestyles, and improved air quality.^218^ Indicators in this section monitor the world’s efforts to reduce greenhouse gas emissions in energy (indicators 3.1 and 3.2), transport (indicator 3.4), food and agriculture (indicator 3.5), and health care (indicator 3.6), and monitor the health benefits that could arise from prioritising health in mitigation policies.

### Indicator 3.1: energy system and health—headline finding: the carbon intensity of the global energy system decreased by less than 1% since 1992, the year the UNFCCC was adopted and energy-related emissions CO_2_ emissions reached a record high in 2021

**Figure 7:**
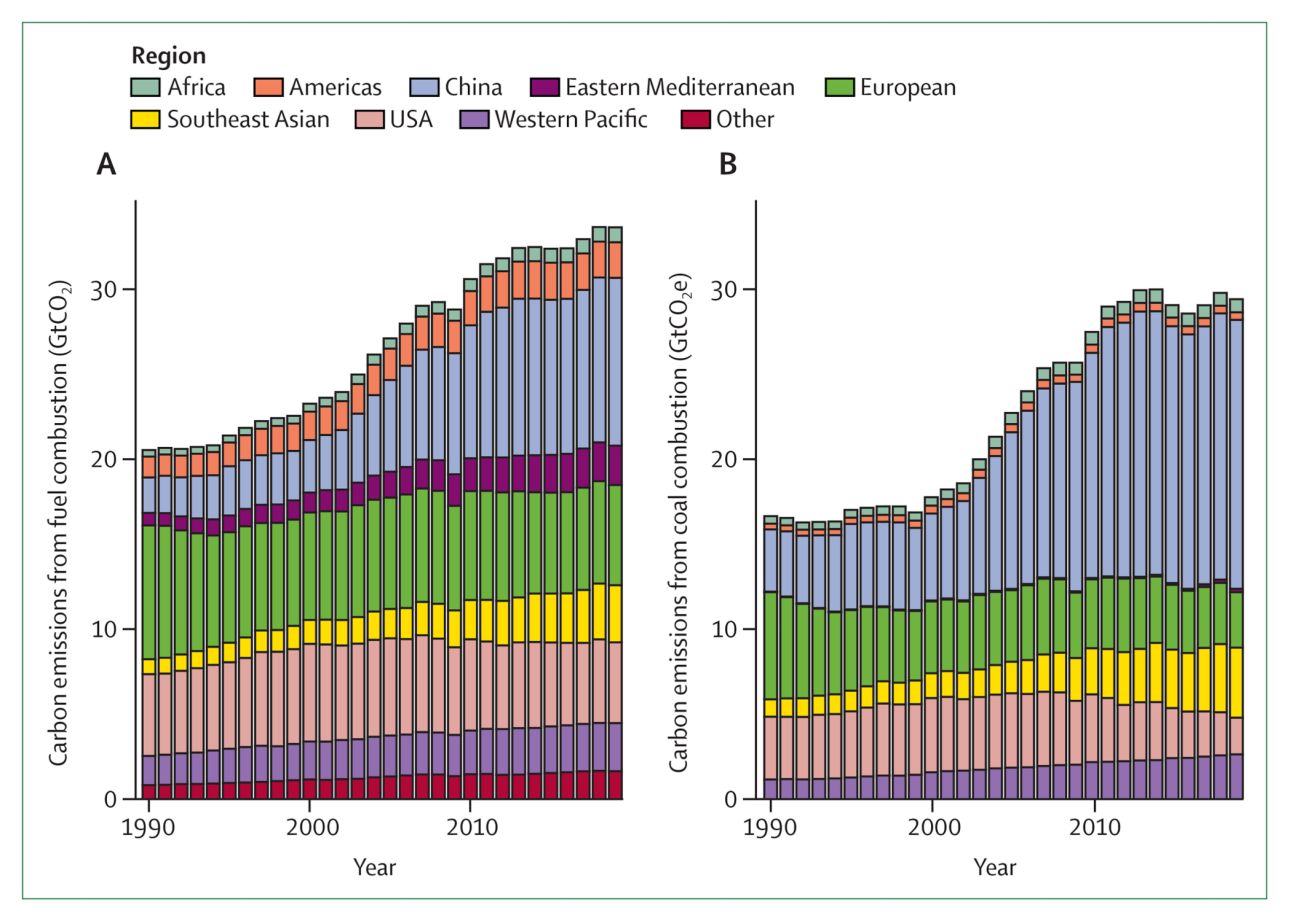
Greenhouse gas emissions from the global energy system (A) Global CO__2__ emissions from fossil fuel use. Preliminary and modelled values shown for 2020. (B) Global CO__2__ emissions from the use of coal. GtCO__2__e=gigatons equivalent CO__2__.

Energy systems are the largest single source of greenhouse gas emissions and are major contributors to air pollution. Global energy system transition to renewables is not only crucial for climate change mitigation,^8^ but could also contribute to universal, affordable, and clean energy;^219^ reduce air pollution; and decrease dependence on international markets and foreign policies. With data from the International Energy Agency, this indicator shows that the carbon intensity of the global energy system continued to decrease in 2019 for the seventh consecutive year, to 55·4 tCO_2_/TJ. However, this is still not enough to keep global warming at 1·5°C, with a reduction of less than 1% from 1992 levels, the year the UNFCCC was adopted. At the pace recorded since 2014, fully decarbonising the energy system would take 150 more years. The increasing demand for energy means fossil fuel use is still rising, and fossil fuel-derived CO_2_ emissions increased again in 2021 by 6·0%, after a 5·1% decrease in 2020 during the COVID-19 pandemic (figure 7), putting CO_2_ emissions at a record high.^216^

Phasing out coal is particularly urgent because of its high greenhouse gas emissions and air pollution intensity. However, coal provides 26·7% of global energy supply, 2·8 percentage points more than in 1992. Responsible for 54% of global coal energy use in 2019, China’s coal expansion has been a major contributor to the rise in global greenhouse gas emissions since the early 2000s, with emissions at 7tCO_2_ per person in 2019, now equivalent to the Organisation for Economic Co-operation and Development (OECD) average.^220^

Growth in renewable electricity reached record levels in 2020, with the installation of 139 GW of solar PV and 93 GW of wind power. This corresponded to 90% of new electricity installation in 2020,^221^ and to renewables providing 8·2% of global electricity, twice the levels in 2013. However, big differences exist between countries globally, and only 1·4% of the electricity of low HDI countries is produced from modern renewables (mostly solar, wind, and geothermal), compared with 9·5% in very high HDI countries. Concerningly, 59% of health care facilities in low and middle-income countries lack the reliable energy services they need to provide basic care;^222^ only 2·2% of total world energy comes from renewable sources; and total fossil fuel use has increased faster. A low-carbon transition can help countries to increase local energy production, gain independence from volatile fossil fuel markets, and reduce energy poverty.

### Indicator 3.2: clean household energy—headline finding: despite improved access to clean fuels, biomass accounted for 31% of global household energy in 2020 and fossil fuels accounted for 26%

**Figure 8:**
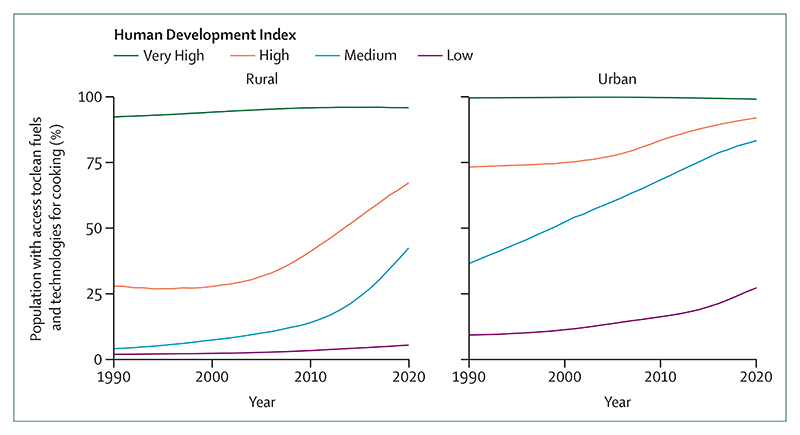
Percentage of the rural and urban population with primary reliance on clean fuels for cooking, by Human Development Index country group

Around 770 million people do not have access to electricity in their homes,^223^ and the use of dirty fuels is leading to high exposure to air pollution.^224^ In parallel, with residential energy contributing to 17% of global greenhouse gas emissions, transitioning to clean fuels in the domestic sector is essential to meet mitigation goals.^225^ With use of IEA data, this indicator reveals that biomass represented the largest individual source of residential energy in 2020, contributing to 31% of residential energy use (a proportion that rises to 96% in low HDI countries); electricity contributed to 25%; and fossil fuels contributed to 26%. Africa improved access to clean energy from 13% to 20% and southeast Asia improved access from 19% to 64% in 2000. However, both regions remain heavily reliant on solid biofuels. Data from WHO indicate that although 86% of the global urban population had access to clean fuels and technologies for cooking in 2020, only 48% of rural populations did. Inequities were also noted between countries, with 98% of the population in very high HDI countries having access to clean fuels and technologies for cooking, against just 13% in low HDI countries (figure 8).

WHO estimates that the use of solid fuels for cooking resulted in 3·8 million deaths attributable to household air pollution in 2016.^226^ Providing the capacity to monitor changes in household air pollution exposure on a yearly basis, this new indicator expands on a previously published model^227^ to estimate household air pollution with a Bayesian hierarchical model that accounts for fuel usage, stove types, socioeconomic variables, and ambient air pollution in 62 countries. This indicator estimates that the use of solid fuels for cooking and heating resulted in a global average PM_2·5_ concentration in people’s homes of 150 µg/m^3^ in 2020 (168 µg/m^3^ in rural households and 91 µg/m^3^ in urban areas). With values broadly exceeding the 5 µg/m^3^ threshold recommended by WHO,^228^ the delayed transition to clean household energies is profoundly affecting people’s health.

Economic hardship during the COVID-19 pandemic has worsened energy insecurity in households in countries of all HDI levels. The number of people without access to electricity increased in 2020 for the first time in six years,^229^ with shifts to the use of biomass and other unreliable fuels increasing exposure to household air pollution.^230,231^ The share of the population without access to electricity in sub-Saharan Africa increased by 3 percentage points to 77% in 2020.^223^ Russia’s invasion of Ukraine threatens to exacerbate energy poverty and the use of unhealthy and unreliable fuels in the domestic sector, with rising energy prices and supply chain disruption.^232,233^

### Indicator 3.3: mortality from ambient air pollution by sector—headline finding: exposure to ambient anthropogenic PM_2·5_ contributed to 3·3 million deaths in 2020, of which 1·2 million were directly related to the combustion of fossil fuels

**Figure 9:**
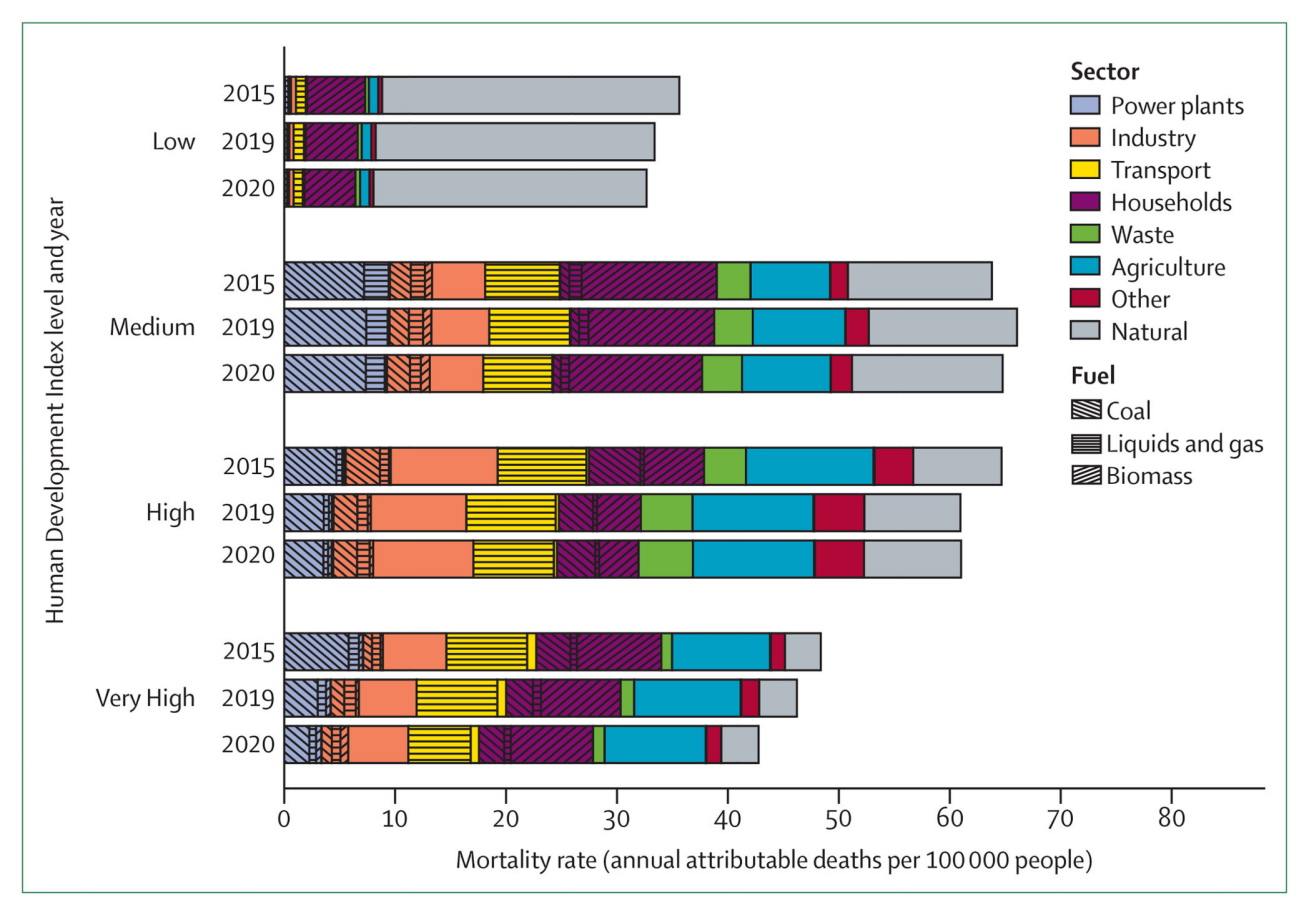
Mortality attributable to ambient PM_2·5_ exposure by region, sector, and fuel source

Exposure to air pollution increases the risk of respiratory and cardiovascular disease, lung cancer, diabetes, neurological disorders, and adverse pregnancy outcomes.^234^ This indicator estimates the mortality attributable to ambient PM_2·5_, combining atmospheric modelling with information about activity in emitting sectors. For this year’s report, baseline mortality data were updated, and attributable deaths from type 2 diabetes were also included.^235^ In 2020, exposure to ambient PM_2·5_ contributed to 4·2 million deaths, unchanged from 2015, and mortality per 100 000 decreased by 5% (figure 9). Of these deaths, 80% (3·3 million) were attributable to anthropogenic emissions; of which 1·2 million (35%) were directly related to the combustion of fossil fuels. Deaths due to coal combustion have decreased by 18% from 687 000 in 2015 to 561 000 in 2020, mostly because of strict air pollution control measures in China and coal phase down in Europe.

### Indicator 3.4: sustainable and healthy road transport—headline finding: use of fossil fuels in road transport decreased by 0·8% in 2019, whereas use of electricity increased by 15·7%

The transport sector contributed to 25% of global CO_2_ emissions in 2019.^5,216,236^ If combined with energy grid decarbonisation, electric vehicles can be an important mitigation tool. The use of electricity for road transport increased by 237% in the past decade, but still represents just 0·3% of total fuel use for road travel. Sales of electric vehicles more than doubled in 2021,^237^ a growth led by China, with nearly 3·4 million sales (12% of the total). However, only 1% of the global car stock is electric.^238^

Road transport decarbonisation with a modal shift to active travel can have health benefits from reduced air pollution, which accounted for 497 000 deaths in 2020 (indicator 3.3) and increased physical activity.^239,240^ Smartphone data suggest that the use of public transit has returned to pre-pandemic levels in 85% of countries for which data are available,^241^ highlighting the need for robust policies that encourage shifts to active travel and public transit modes.

### Indicator 3.5: food, agriculture, and health

The global food system contributes one third of all greenhouse gas emissions.^242^ Emissions from the agricultural sector are dominated by ruminant rearing, mostly mediated by methane emissions and land use change.^243,244^ Shifting to low-carbon, plant-forward diets can help mitigate agricultural emissions as well as have important health co-benefits from improvements in dietary risk factors and mortality from non-communicable diseases.^235,245,246^ These two indicators track agricultural emissions (indicator 3.5.1) and the health impacts of carbon-intensive diets (indicator 3.5.2), identifying the potential health opportunity of agricultural decarbonisation.

#### Indicator 3.5.1: emissions from agricultural production and consumption—headline finding: red meat and milk contribute to 55% of global agriculture emissions

**Figure 10:**
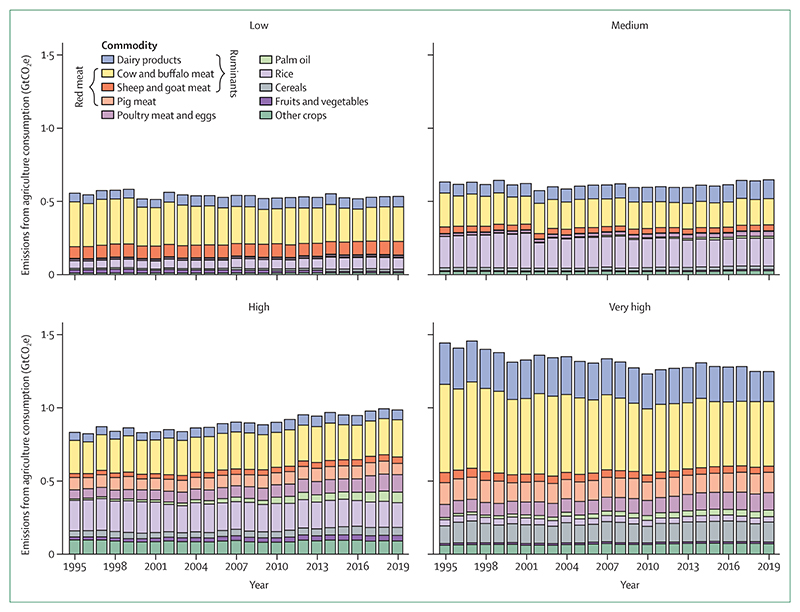
Emissions of greenhouse gases on farms associated with food consumption (production and net imports) per person by Human Development Index level GtCO__2__e=gigatons equivalent CO__2__.

This indicator, improved from previous reports to include data on 140 food types, estimates that emissions from the consumption of agricultural products have remained stable, at around 0·9 tonnes CO_2_ equivalent (tCO_2_e) per person, although total emissions have increased by 31% since 2000 (figure 10). In 2019, 55% of global agricultural emissions came from red meat and dairy products. Per capita emissions from red meat and dairy consumption in very high HDI countries were twice the emissions in the rest of the world (0·8 tCO_2_e per person *vs* 0·4 tCO_2_e per person). Increases in palm oil production account for some of the greatest changes since 2000, for which emissions in southeast Asia (mainly Indonesia) increased over 600%.

#### Indicator 3.5.2: diet and health co-benefits—headline finding: in 2019, 11·5 million deaths were attributable to imbalanced diets, with 17% associated with a high intake of red and processed meat and dairy products

This indicator monitors the health burden from unhealthy diets and, new to this year, the burden of imbalanced energy intake.

In 2019, 11·5 million deaths were attributable to imbalanced diets. 17% (2 million) of them were related to red and processed meat and dairy consumption, of which 93% were in high and very high HDI countries. In low and medium HDI countries, the low consumption of fresh fruit and vegetables was the major contributor to diet-related mortality, at 44% of all diet-related deaths in low HDI countries, and 37% in medium HDI countries.

### Indicator 3.6: health-care sector emissions—headline finding: from 2018 to 2019, emissions from the health-care sector increased by more than 5%, reaching 5·2% of global greenhouse gas emissions

**Figure 11:**
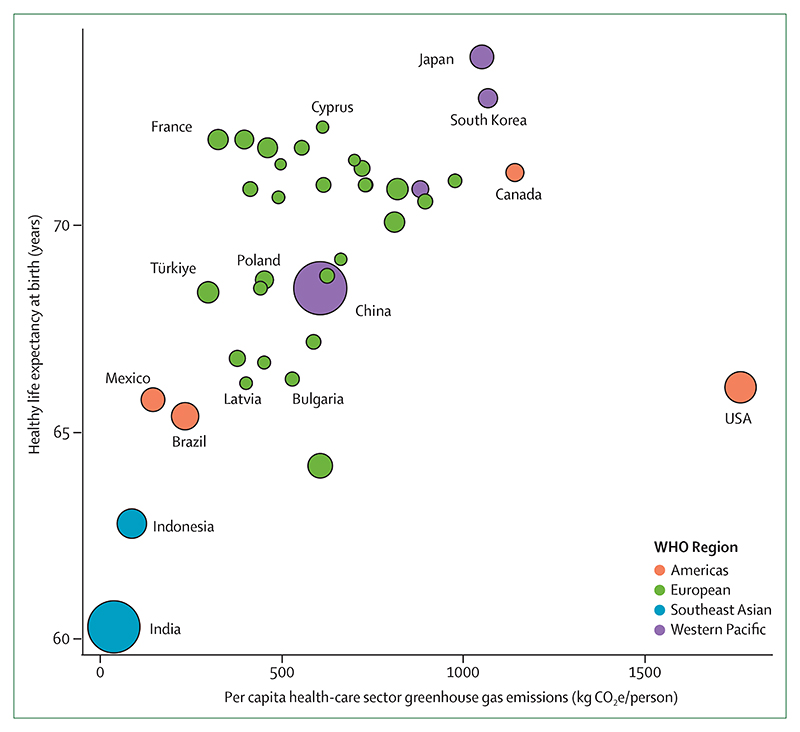
National greenhouse gas emissions per person from the health-care sector against the healthy life expectancy at birth in 2019, by WHO region The point circle size is proportional to country population. kgCO__2__e=kilograms of carbon dioxide equivalent.

Because of the health impacts of climate change, health systems need to lead decarbonisation, to comply with their duty of not harming health. This indicator monitors health-care sector emissions combining health-care expenditure data with a global, environmentally-extended multi-region input–output model. The indicator estimates that, in 2019, the health-care sector contributed to approximately 5·2% (2·7 GtCO_2_e) of global greenhouse gas emissions, an increase of more than 5% from the previous year. Of the 37 health systems analysed individually, the USA had the most emissions per person—50 times the emissions of India (figure 11). Yet, the USA has the sixth lowest healthy life expectancy at birth (66·2 years). Per capita emissions in the 10 countries with the highest life expectancy ranged from 1065 kgCO_2_e per person in South Korea, to 321 kgCO_2_e per person in France, highlighting that high-quality health care can be achieved with lower emissions. Recent decarbonisation commitments made as part of the COP26 health programme since November, 2021, by more than 50 national health services provide hope for emerging progress (panel 7).^247^

## Conclusion

After COVID-19 pandemic lockdowns were lifted and restrictions were eased, CO_2_ emissions rebounded to record levels in 2021. With each year that global greenhouse gas emissions do not fall, reaching net-zero by 2050 becomes more challenging, putting lives at increased risk from climate change.

Although impacts of COVID-19 on the indicators in this section are still emerging, many of the challenges for mitigation and health co-benefits have been entrenched since the start of the pandemic, including the domestic over-reliance on biomass, record levels of coal extraction in China, and rebounding emissions from road transport. The energy crisis, worsened by Russia’s war on Ukraine, threatens to deteriorate this situation, further under-mining progress and exacerbating energy poverty. However, increasing energy efficiency, conservation, and the use of renewable energy sources could give healthier, more resilient, and self-sufficient energy systems. Millions of lives could be saved each year by accelerating transition to cleaner fuels, healthier diets, and active modes of travel.

## Section 4: economics and finance

Limiting the global temperature rise to 1·5°C requires rapid decarbonisation in all economic sectors. Although the initial investment required for a low-carbon transformation is substantial, the investment would lead to immediate economic and health co-benefits, in addition to avoiding long-term climate change impacts.^249,250^ With the right incentives, and market and governance conditions, the necessary private sector investment is available. However, wealthier parties to the UNFCCC have failed to meet their commitment of delivering the smaller sum of US$100 billion annually to support climate action in “developing” countries that they committed to 13 years ago;^267^ a commitment essential not only for attaining global climate goals, but also to ensure a just transition.^8^ In addition, the energy crisis, worsened by the COVID-19 pandemic and exacerbated by the war in Ukraine, is increasing energy poverty, and exposing further dimensions of the human costs of a fossil fuel-dependent global energy system. Indicators in this section detail the economic costs of climate change, and monitor the transition to a low-carbon, healthy, and just global economy.

### Indicator 4.1: the economic impact of climate change and its mitigation

Climate change is causing additional health-care costs, loss of labour productivity, and economic losses through the damage caused by climate-related extreme events. These costs and losses subsequently affect household incomes and national economies. Indicators in this section monitor the economic costs associated with the health impacts of climate change, showing the potential benefits from accelerated climate action.

#### Indicator 4.1.1: economic losses from climate-related extreme events—headline finding: around 84% of global economic losses due to climate-related extreme events in 2021 affected very high HDI countries, double the global average loss as a proportion of GDP. While half of their losses were insured, the majority of losses in other countries were uninsured

The loss of infrastructure and resulting economic losses due to extreme events can exacerbate health impacts with disruption of essential services and effects on the social determinants of health. This indicator monitors the economic losses from climate-related extreme events, with data provided by Swiss Re.^251^

In 2021, climate-related extreme events induced measurable economic losses of US$253 billion, with 84% of these losses in very high HDI countries. As a proportion of GDP, losses in the very high HDI group are double the global average. Nearly half of these losses were insured, although insured losses represented only 8% of all losses in high HDI countries and 5% in medium HDI countries, with almost 0% in the low HDI country group. These high levels of uninsured losses worsen the economic burden of climate change in low HDI countries, with losses either not being replaced, or individuals and institutions incurring the cost of replacement.

#### Indicator 4.1.2: costs of heat-related mortality—headline finding: the monetised value of global heat-related mortality was estimated to be $144 billion in 2021, equivalent to the average income of 12·4 million people

**Figure 12:**
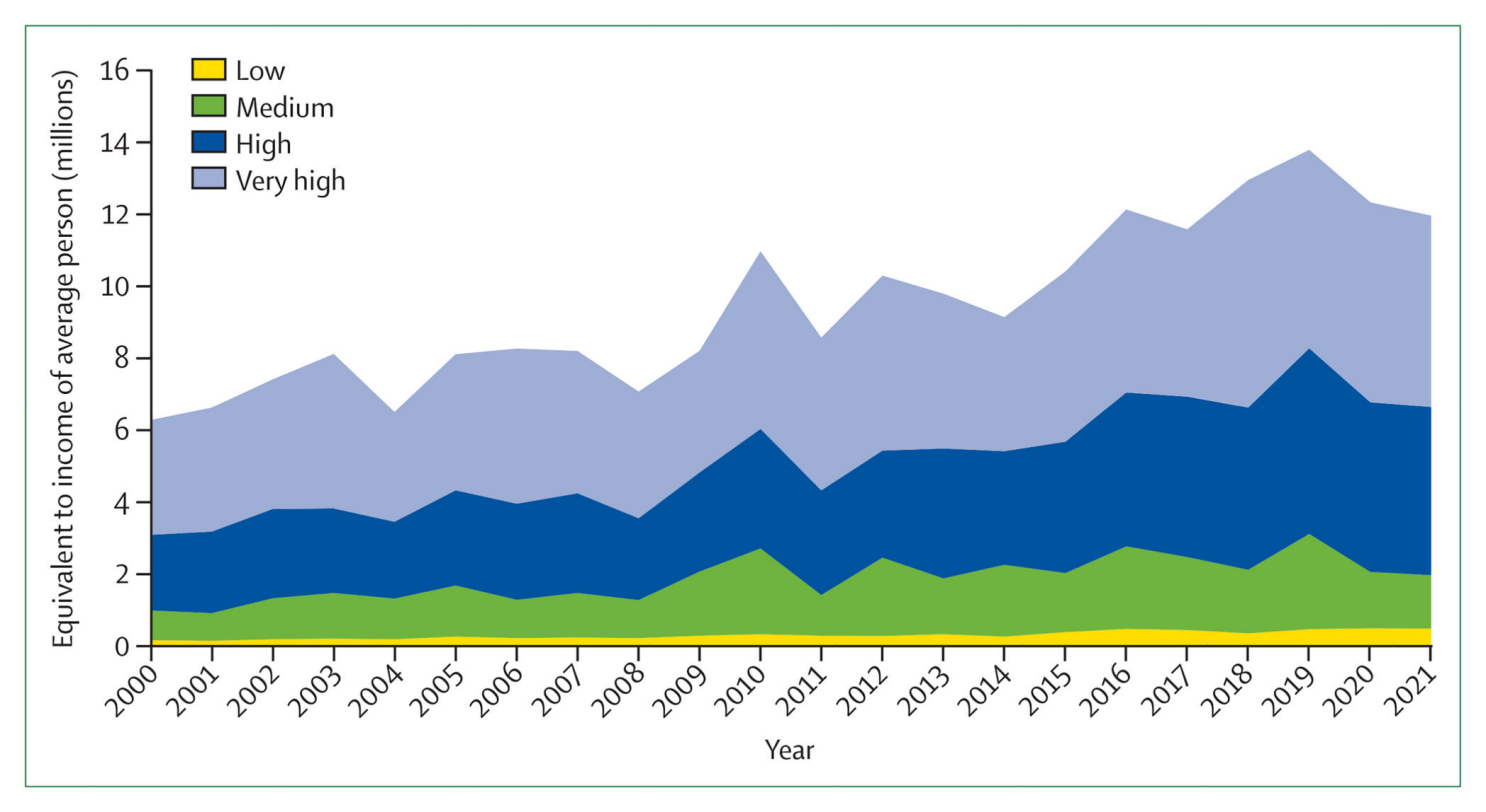
Monetised value of heat-related mortality (in terms of equivalence to the average income) by Human Development Index country groups from 2000 to 2021

This indicator combines estimates of years of life lost data from indicator 1.1.6 with the value of statistical life year to estimate the monetised loss caused by heat-related mortality. The valuation of life across varying HDI levels presents methodological and ethical challenges, which this indicator addresses by presenting the cost of deaths attributable to heat as the proportion of GDP and the equivalent annual average income in the countries concerned. From 2000 to 2021, monetised losses increased at an average rate of $4·9 billion each year, equivalent to 0·16% of gross world product in 2021 (figure 12). The past 6 years show the highest losses, at an average equivalent to the income of 12·4 million people, 73% higher than in 2000–05. In 2021, very high HDI countries incurred the highest losses, equivalent to 5·3 million of their populations’ average income, with losses equivalent to 4·7 million in high HDI countries, 1·48 million in medium HDI countries, and 0·51 million in low HDI countries.

#### Indicator 4.1.3: loss of earnings from heat-related reduction in labour capacity—headline finding: the global potential loss of income from reduction in labour capacity due to extreme heat was US$669 billion in 2021. The agricultural sector was the most severely affected, incurring 82% of the average losses in low HDI countries and 71% in medium HDI countries

This indicator quantifies the loss of earnings that could result from heat-related labour capacity loss, combining data from indicator 1.1.4 with hourly wage data from the International Labour Organization.

**Figure 13:**
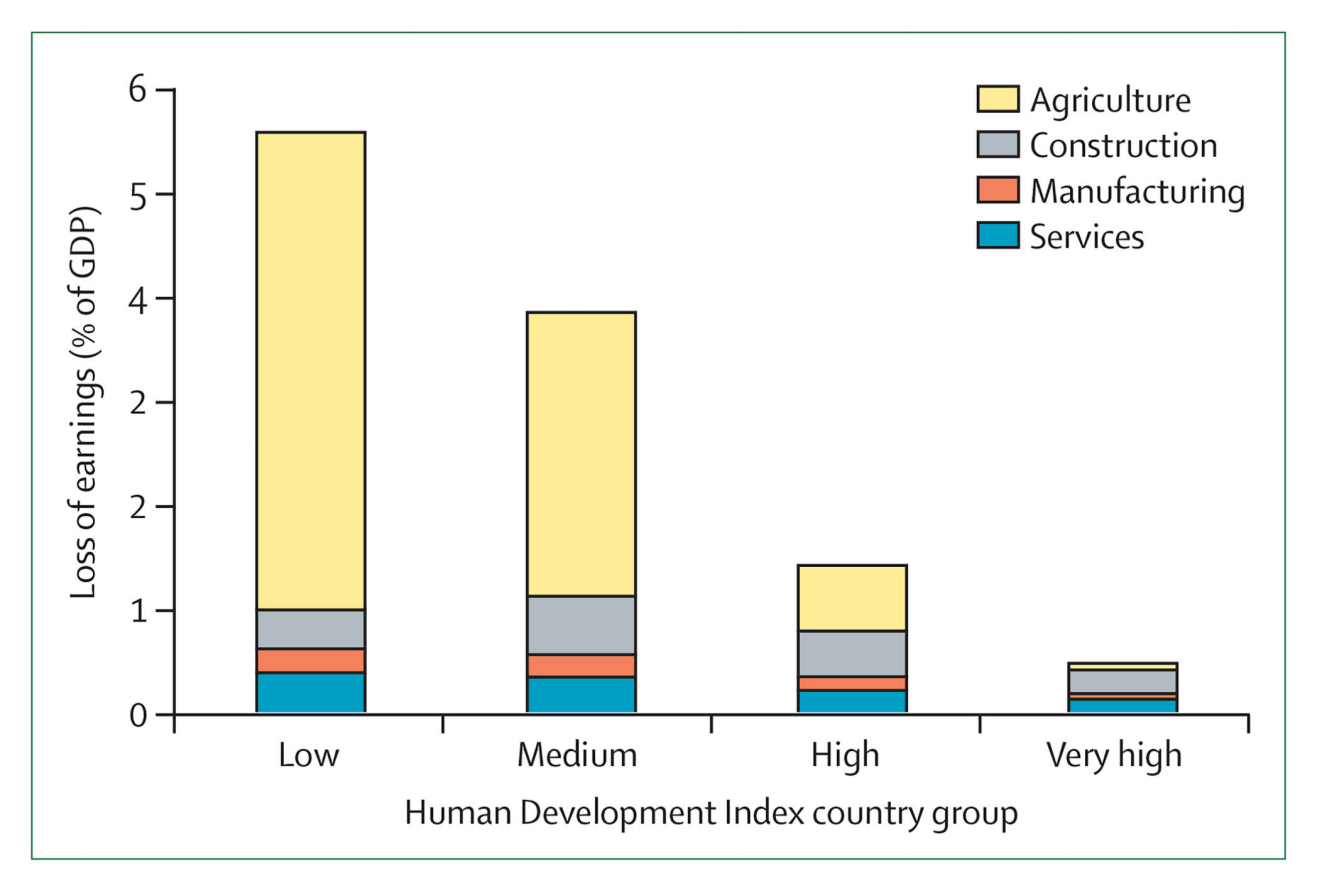
Average potential loss of earnings per Human Development Index country group as a result of potential labour loss due to heat exposure Losses are presented as share of gross domestic product and sector of employment.

The global potential loss of earnings was US$669 billion in 2021, equivalent to 0·72% of gross world product in 2021. In 2021, average relative income losses were equivalent to 5·6% of GDP for low HDI countries and 3·9% of GDP for medium HDI countries—the highest average relative income losses (figure 12; figure 13). Of all global losses, 40% occurred in the agricultural sector. Often being among the world’s poorest, agricultural workers in low HDI countries had losses of 82% and those in medium HDI countries had losses of 71%.^252–254^ Affecting individual finances, these losses impact on people’s wellbeing, food security, and the social determinants of health,^2^ and cascade through the economies of the nations they live in.

#### Indicator 4.1.4: costs of the health impacts of air pollution—headline finding: the monetised costs of premature mortality due to air pollution amounted to US$2·3 trillion in 2020, the equivalent of 2·7% of gross world product

This indicator places an economic value on the years of life lost from exposure to anthropogenic ambient PM_2·5_ as per indicator 3.3. Whereas costs relative to average income and GDP decreased between 2019 and 2020 in all HDI groups, the total cost amounted to US$2·3 trillion in 2020—the equivalent of 2·7% of gross world product. The high HDI country group has the greatest costs relative to per capita income, equivalent to the average annual income of 92·3 million of its people. The medium HDI group has the greatest costs relative to the size of their collective economies, equivalent to nearly 4% of GDP.

### Indicator 4.2: the economics of the transition to net zero-carbon economies

Meeting the Paris Agreement goals requires a low-carbon transition of the whole economy. Indicators in this section monitor jobs and investment in low-carbon energy, net carbon pricing, and the effect of global trade on emissions. A new indicator quantifies the extent to which the activities of oil and gas firms align with the pathways needed to keep global mean temperature rise to under 1·5°C of heating.

#### Indicator 4.2.1: clean energy investment—headline finding: between 2020 and 2021, investment in global energy supply investment increased by 14%; zero-carbon sources accounted for 80% of investment in electricity generation in 2021

As described in the previous section, phasing out fossil fuels, particularly coal, and investing in low-carbon energy supply is essential for both mitigating climate change and for reducing premature mortality caused by air pollution. With data from the IEA, this indicator monitors trends in global investment in energy supply and energy efficiency.

Between 2020 and 2021, total investment increased by 14%, with investment increasing in all forms of energy supply and energy end use efficiency, except coal for electricity generation. In 2021, electricity generation accounted for 28% of investment. Of this investment, 80% was invested in zero-carbon sources. However, fossil fuels still account for more than 90% of non-electricity sector investment. Energy efficiency accounted for 15% of all investment—an increase from 13% in 2020. To fulfil net-zero global emissions by 2050, investment in low-carbon energy, efficiency, and electricity networks needs to nearly quadruple by 2030, and account for at least 90% of all energy investment.^255^

#### Indicator 4.2.2: employment in low-carbon and high-carbon industries—headline finding: with more than 12 million employees, direct and indirect employment in renewable energy exceeded direct employment in fossil fuel extraction for the first time in 2020

Employees in fossil fuel extraction industries, particularly coal mining, can have a greater incidence of non-communicable disease than the general population.^256^ Increasing employment in the renewable industry could improve health and livelihoods. In addition, it could improve gender balance, with a greater proportion of women employed in the renewable sector than in the fossil fuel industry.^257^

This indicator shows that more than 12 million people were employed directly or indirectly by the renewable energy industry in 2020—an increase of 5% from 2019. For the first time, direct and indirect employment in the renewable energy sector exceeded direct employment in the fossil fuel extraction industry, which recorded 10·5 million employees (a decrease of 10% from 2019), reaffirming that renewable energy could support job security, now and in the future.

#### Indicator 4.2.3: funds divested from fossil fuels—headline finding: the global value of funds committing to fossil fuel divestment between 2008 and 2021 was $40·23 trillion, with health institutions accounting for $54 billion

By divesting holdings in fossil fuel companies, organisations can both reduce the social licence of fossil fuel companies to operate, and hedge against the risk of losses caused by stranded assets in an increasingly decarbonised world.^258,259^ This indicator tracks the value of funds divested from fossil fuels, with data provided by stand.earth and 350.org.

From 2008 until the end of 2021, 1506 organisations, with assets worth at least US$40·23 trillion, have committed to divestment. Of these organisations, only 27 are health institutions, with assets totalling $54 billion. The value of new funds committed to divesting in 2021 was $9·42 trillion, with no new health institutions divesting.

#### Indicator 4.2.4: net value of fossil fuel subsidies and carbon prices—headline finding: 69 (80%) of the 86 countries reviewed had a net-negative carbon price in 2019, hindering the transition from fossil fuels. The resulting net loss of government revenue was in many cases equivalent to large proportions of the national health budget

**Figure 14:**
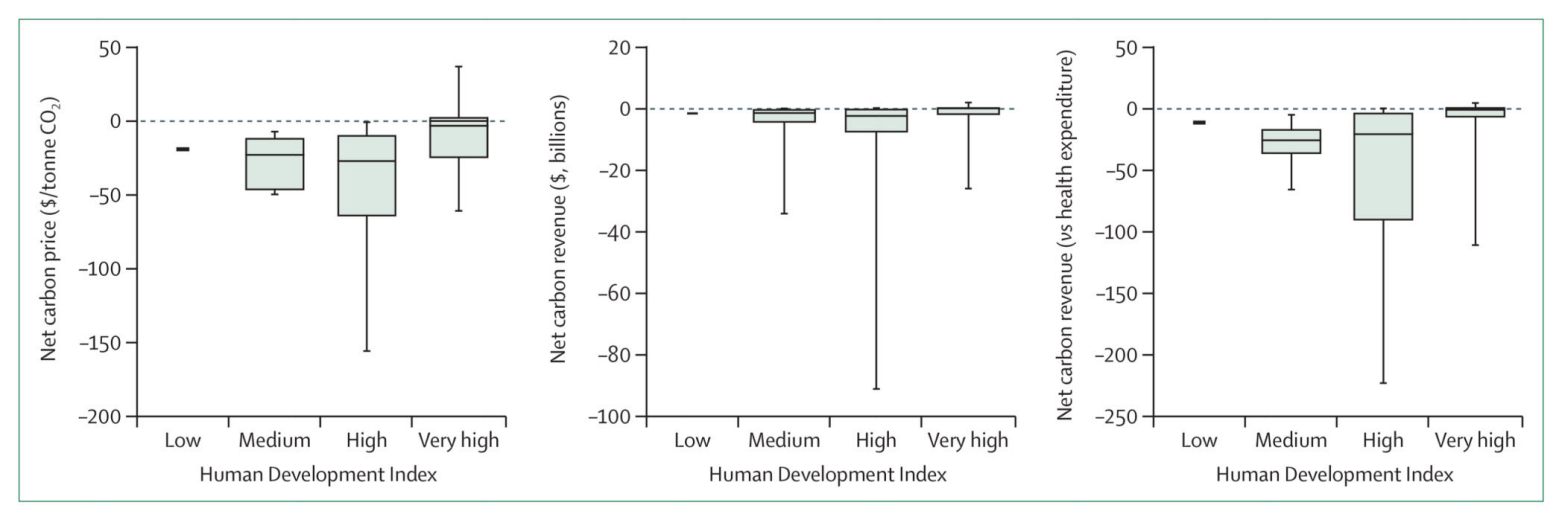
Net carbon prices (left), net carbon revenues (centre), and net carbon revenue as a share of current national health expenditure (%; right) in 86 countries in 2019 Arranged by Human Development Index country group: low (n=1), medium (n=7), high (n=24), and very high (n=54). Boxes show the interquartile range, horizontal lines inside the boxes show the medians, and the brackets represent the full range from minimum to maximum. Currency is US$.

Carbon prices help economies transition from high-carbon fuels, but many governments subsidise fossil fuels, encouraging health-harming emissions and slowing the low-carbon transition. This indicator compares carbon prices and monetary fossil fuel subsidies to calculate net economy-wide average carbon prices and revenues in 86 countries, responsible for 92% of global CO_2_ emissions. In 2019, 42 countries had a system for carbon pricing, but only 17 produced a net-positive carbon price—all of which were very high HDI countries (figure 14). 69 (80%) of 86 countries reviewed had net-negative carbon prices (ie, provided a net subsidy to fossil fuels) for a net total of US$400 billion that year, with ten countries each exceeding $10 billion of net subsidies. In 31 countries, net subsidies exceeded 10% of national health spending, and exceeded 100% in 5 countries.

Redirecting government support from subsidising fossil fuels to low-carbon power generation, health protection, public health promotion, and health care is likely to deliver net benefits to health and wellbeing.^260,261^ International financing mechanisms are needed to support low-income countries that are most affected by fluctuating energy costs in their transition to sustainable energy sources, particularly with the current energy crisis, and to safeguard all dimensions of human health.^261^

#### Indicator 4.2.5: production-based and consumption-based of CO_2_ and PM_2·5_ emissions—headline finding: in 2020, 18% of CO_2_ and 17% of PM_2·5_ global emissions were from the production of goods and services traded between countries of different HDI levels. The very high HDI country group was the only group with net outsourcing of both CO_2_ and PM_2·5_ emissions from its consumption

The production of goods and services results in local greenhouse gas and PM_2·5_ emissions, which can be monitored with production-based emission accounting. However, these goods and services are often consumed in different locations than where they were produced. Consumption-based emission accounting allocates emissions to countries according to their consumption of goods and services. This indicator uses an environmentally-extended multi-region input–output model, and the same air pollution modelling described in indicator 3.3,^262–264^ to assess each country’s consumption-based and production-based contribution to CO_2_ and PM_2·5_ emissions.

In 2020, 18% of CO_2_ and 17% of PM_2·5_ global emissions were from the production of goods and services traded between countries of different HDI levels. Emissions were 3% lower for CO_2_ and 7% lower for PM_2·5_ than the year before—likely to be a result of restrictions during the COVID-19 pandemic. In 2020, the very high HDI country group contributed the most consumption-based (47%) CO_2_ emissions, whereas the high HDI country group contributed the most production-based (46%) CO_2_ emissions. However, consumption-based emissions per person were highest in very high HDI countries, 1·3 times higher than the global average, and 26·3 times higher than emissions per person in low-HDI countries.

**Figure 15:**
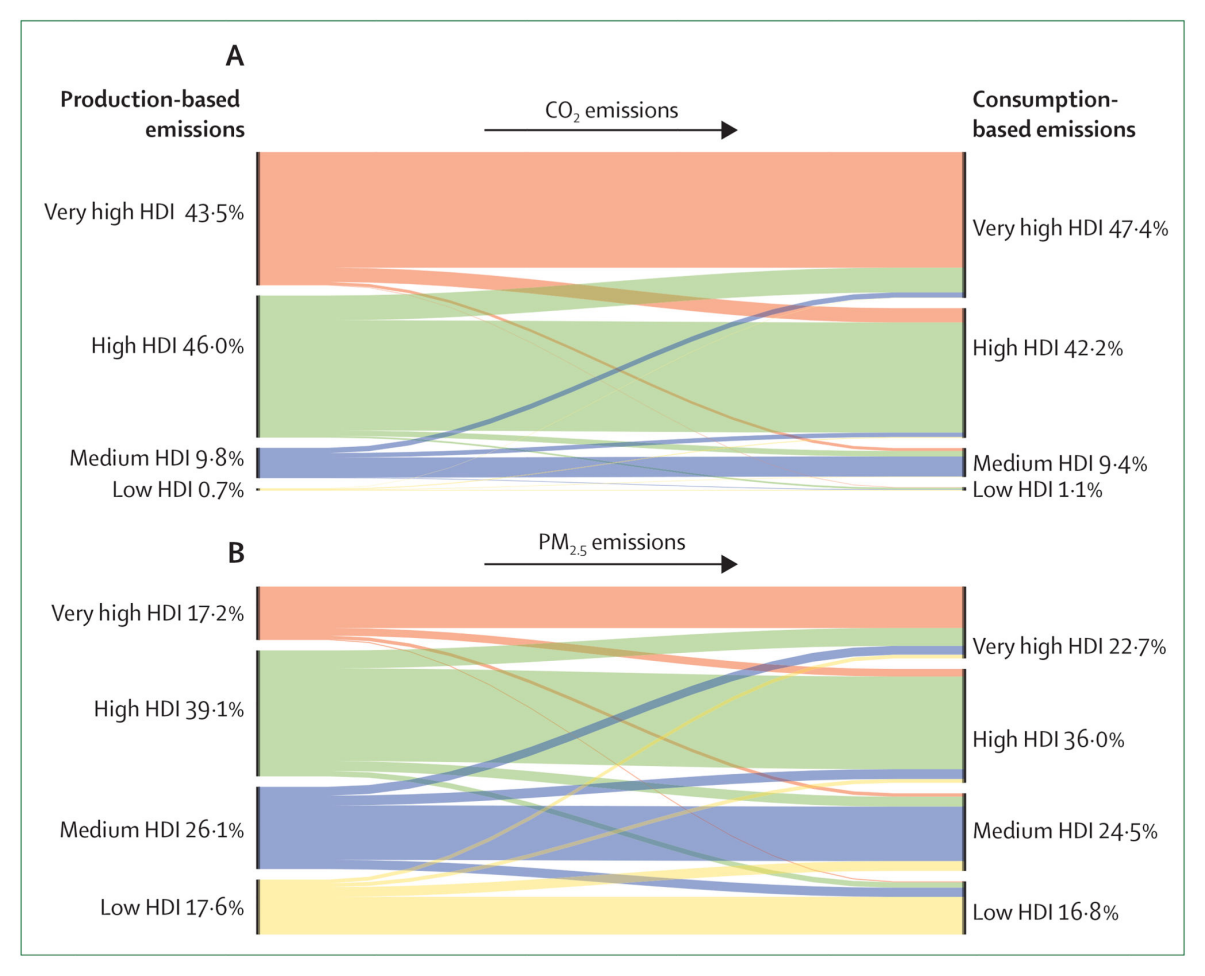
CO_2_ and PM_2·5_ emissions emitted in the production of goods and services traded between countries in 2020, grouped by HDI HDI=Human Development Index.

High HDI countries were the biggest contributors to both production-based (39%) and consumption-based (36%) PM_2·5_ emissions, even if their contribution share decreased from 2019 (figure 15). PM_2·5_ emissions per person were largest in low HDI countries, a reflection of poor air quality control measures and the use of more polluting fuels. The very high HDI country group was the only group with higher consumption-based than production-based emissions of both CO_2_ and PM_2·5_ emissions.

#### Indicator 4.2.6: compatibility of fossil fuel company strategies with the Paris Agreement—headline finding: the current strategies of 15 of the largest oil and gas companies would lead to production exceeding their share of levels consistent with limiting the global average surface temperature rise to 1·5°C by 37% in 2030, and 103% in 2040

Emissions from oil and gas need to be reduced drastically to enable a healthy future.^8,265^ This indicator assesses the extent to which current oil and gas company production strategies are compatible with Paris Agreement goals, regardless of their claims and commitments. The indicator uses data from the Rystad energy database on commercial activities for the eight largest publicly listed international oil and gas companies (IOCs) by production volume, and the seven largest state-owned national oil and gas companies (NOCs). These IOCs accounted for 14% of total global production in 2021 and NOCs accounted for 28% (42% overall). Projected emissions based on current strategies are compared with a pathway compliant with 1·5°C, assuming constant market shares at the 2015–19 average.

**Figure 16:**
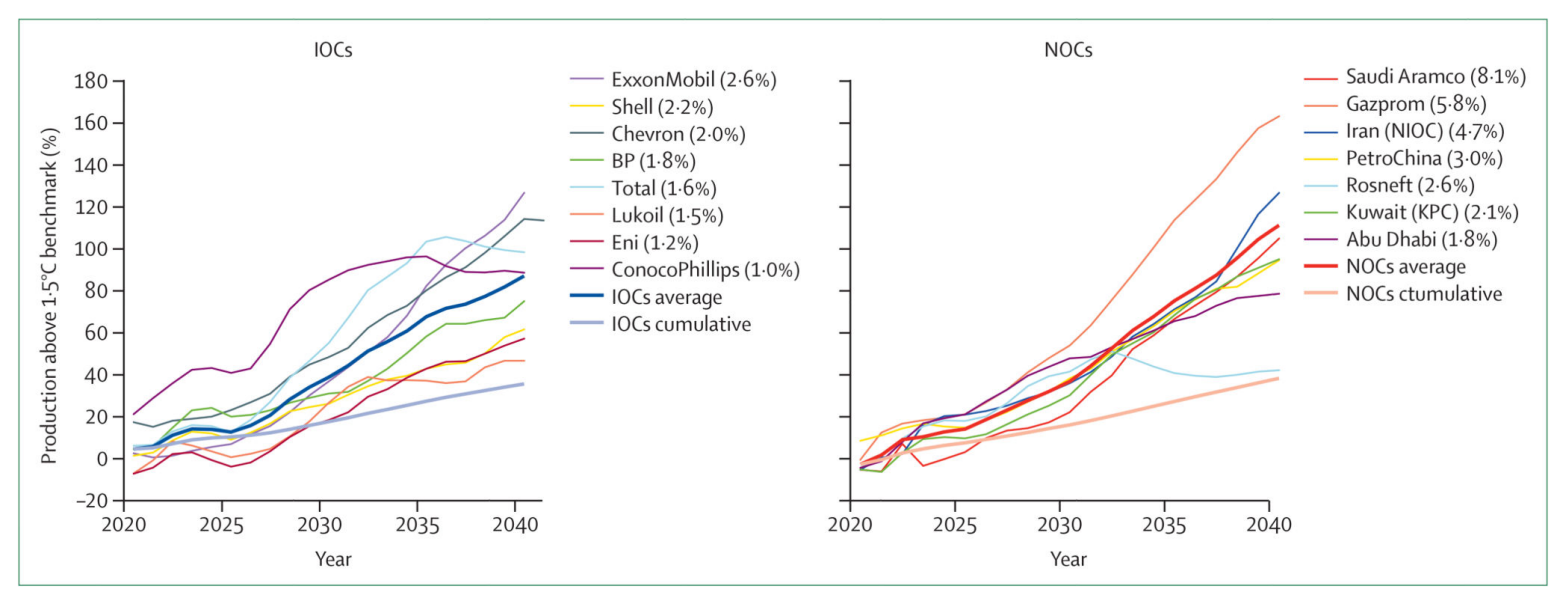
Compatibility of large oil and gas company production strategies with the Paris Agreement climate target of 1·5°C Percentages in brackets in the legend represent the average 2015–19 global market share for each company. IOCs=international oil and gas companies. NOCs=national oil and gas companies.

Data in this indicator suggest that the production strategies of these companies would generate greenhouse gas emissions that exceed their share compatible with 1·5°C by an average of 39% for these IOCs, and 37% for the NOCs, in 2030. These excess emissions would rise to 87% for IOCs and 111% for NOCs in 2040 (figure 16). Cumulative production from 2020 to 2040 is projected to exceed their share of the 1·5°C target by 36% for IOCs and 38% for NOCs.

According to these results, the activities of some of the largest oil and gas companies are far from compliant with the goals of the Paris Agreement. Strong government action and pressure from civil society could be essential to bring about such compliance, with a faster transition from fossil fuels to low-carbon energy sources.

## Conclusion

Indicators in this section highlight some of the extensive costs associated with the health impacts of climate change (panel 8). The economic impacts of climate change are affecting livelihoods and the socioeconomic conditions that good physical and mental health depend on. Substantial and sustained investment in the low-carbon transition is essential to minimise these impacts for a healthy future. Both governments and the private sector have crucial roles in making this happen. Indicators show that investments and employment are slowly transitioning from fossil fuels to clean energy, and divestment from fossil fuel assets is also increasing. However, the pace needs to be accelerated to prevent devastating economic and health impacts of climate change. Yet, governments continue to incentivise a carbon-intensive and health-harming economy by subsidising fossil fuels to a level of value often equivalent to substantial proportions of national health budgets. Meanwhile, oil and gas companies are on track to exceed their share of maximum emissions compatible with 1·5°C of heating by more than 100% in 2040. Increased regulations, scrutiny, and accountability mechanisms need to be urgently implemented to ensure the energy sector aligns its activities with agreed climate targets. Governments worldwide must urgently accelerate this transition, by setting regulations and redirecting investment to a low-carbon, healthy, and energy-resilient future.

## Section 5: public and political engagement

The integration of health and climate policies is essential for a rapid climate transition that protects human health,^270,271^ particularly in countries and communities that have contributed least to rising global temperatures, yet are the most affected by them.^272–75^ Public and political engagement with the health dimensions of climate change is essential to deliver equity-focused climate policies at speed and scale, and to close implementation gaps.^276,277^

This section focuses on key domains of public and political engagement in health and climate change: engagement by the mainstream media (indicator 5.1), individuals (indicator 5.2), the scientific community (indicator 5.3), governments (indicator 5.4), and the corporate sector (indicator 5.5). Where relevant, data is analysed as from 2007, the year before the UN World Health Assembly made a multilateral commitment to protect people’s health from climate change.^278^ Where relevant, the analysis includes engagement with climate change adaptation and pandemic preparedness, to capture engagement with key dimensions of a coordinated response to climate change and the COVID-19 pandemic (appendix 5 p 164).

### Indicator 5.1: media engagement in health and climate change—headline finding: coverage of health and climate change in media reached a record of 14 474 articles in 2021; however, this coverage only constitutes a small proportion of climate change coverage

Newspapers, in their print and online versions, are a widely used source of public information that influence public perceptions on climate change,^279,280^ governments,^281^ and the social media agenda.^282^ This indicator covers analysis articles in newspapers across 37 countries, including China’s People’s Daily, and its method is based on keyword searches (in English, German, Portuguese, Spanish, and Chinese) of relevant newspaper databases.

**Figure 17:**
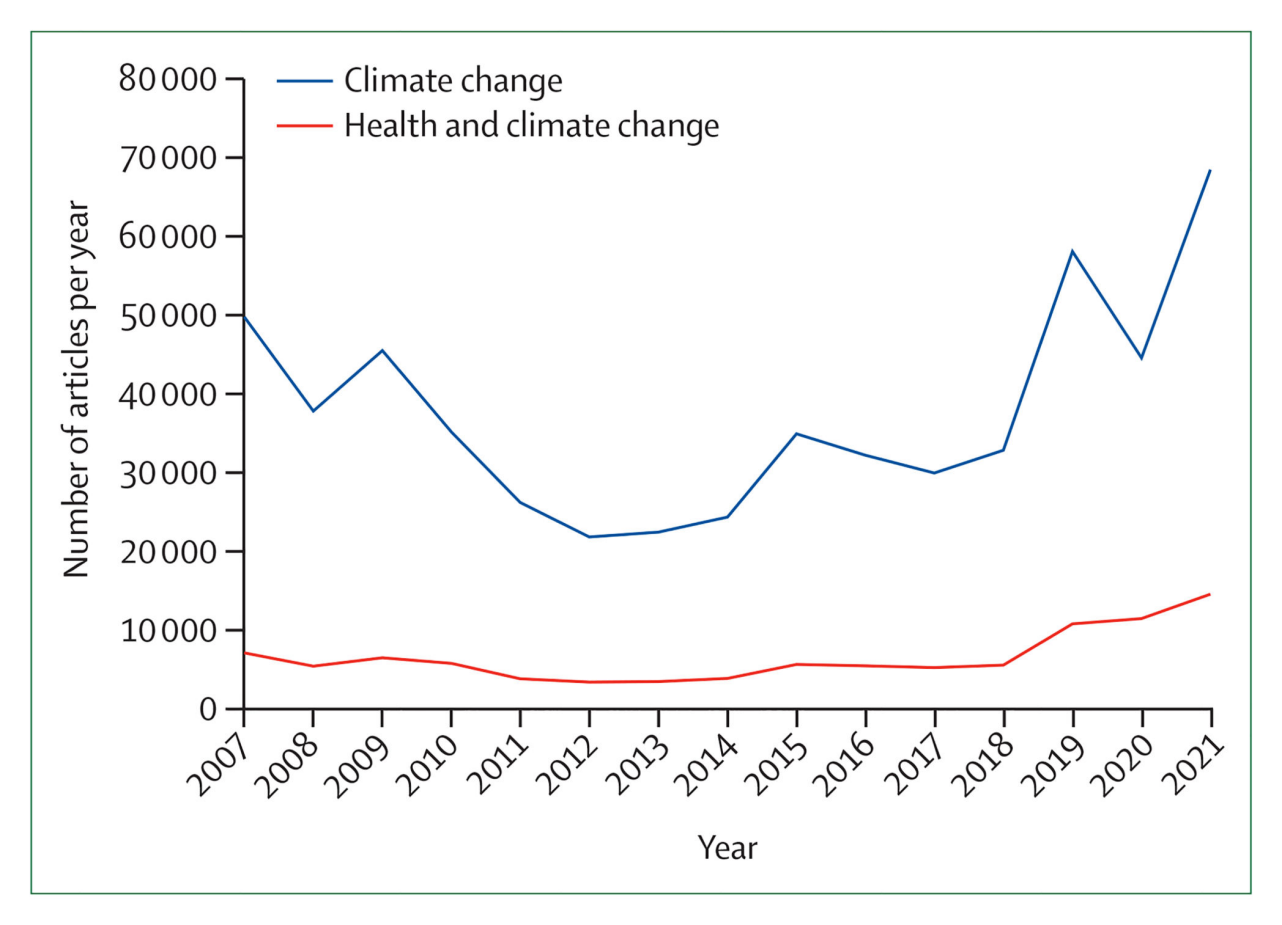
Newspaper coverage of health and climate change in 36 countries from 2007 to 2021

In 2021, global coverage of both climate change and health reached a new record high, with 14 474 articles that year, 27% more than in 2020 (figure 17). In China’s People’s Daily, climate change coverage also reached its highest recorded level. Coverage of health and climate change remained scarce, with only 1% of People’s Daily articles relating to both issues; none of these articles covered pandemic preparedness and only one referenced adaptation.

In English language newspapers (n=51) across 24 countries, 2554 (20%) of 13 017 of articles referring to both health and climate change also referred to adaptation, and 6258 (48%) referred to the pandemic. Very few (645; 5%) referred to health, climate change, adaptation, and the pandemic.

### Indicator 5.2: individual engagement in health and climate change—headline finding: individual engagement in health and climate change increased by 19% between 2020 and 2021, but health and climate change are topics that people did not frequently engage with at the same time

This indicator is based on global use of the online encyclopaedia Wikipedia, an information source with increasing coverage and comprehensiveness and wide public reach,^283–287^ that amplifies the diffusion of science.^288,289^

The indicator tracks people’s movements between articles on health and on climate change (known as clickstream statistics), based on the English Wikipedia, the most popular language edition in multiple countries worldwide.^290,291^

Users click between articles on health or on climate change, with these domains heavily co-visited internally. There are fewer connections between domains: health and climate change are seldom topics that people engage with at the same time. Of all click views leading to a climate change-related article, 0·3% came from a health-related article; of click views leading to a health-related article, 0·02% came from a climate change-related article. These movements increased by 19% from 2020 to 2021, reversing the decline between 2019 and 2020. The COVID-19 pandemic continued to be a key driver of online engagement on health and climate change; for example, COP26 coincided with a higher engagement on health and climate change, but this was mainly driven by interest in the pandemic situation in its host country.

### Indicator 5.3: scientific engagement in health and climate change—headline finding: the number of scientific papers investigating health and climate change increased by 22% from 2020 to 2021

Scientific engagement is tracked in peer-reviewed journals, the primary source of scientific evidence for the media and governments.^287,292^ This indicator uses an enhanced method in this year’s report, with supervised machine learning and associated methods (topic modelling and geoparsing) to map scientific articles on health and climate change over time,^293^ extending the time period to 1985–2021 from the previous report.

**Figure 18:**
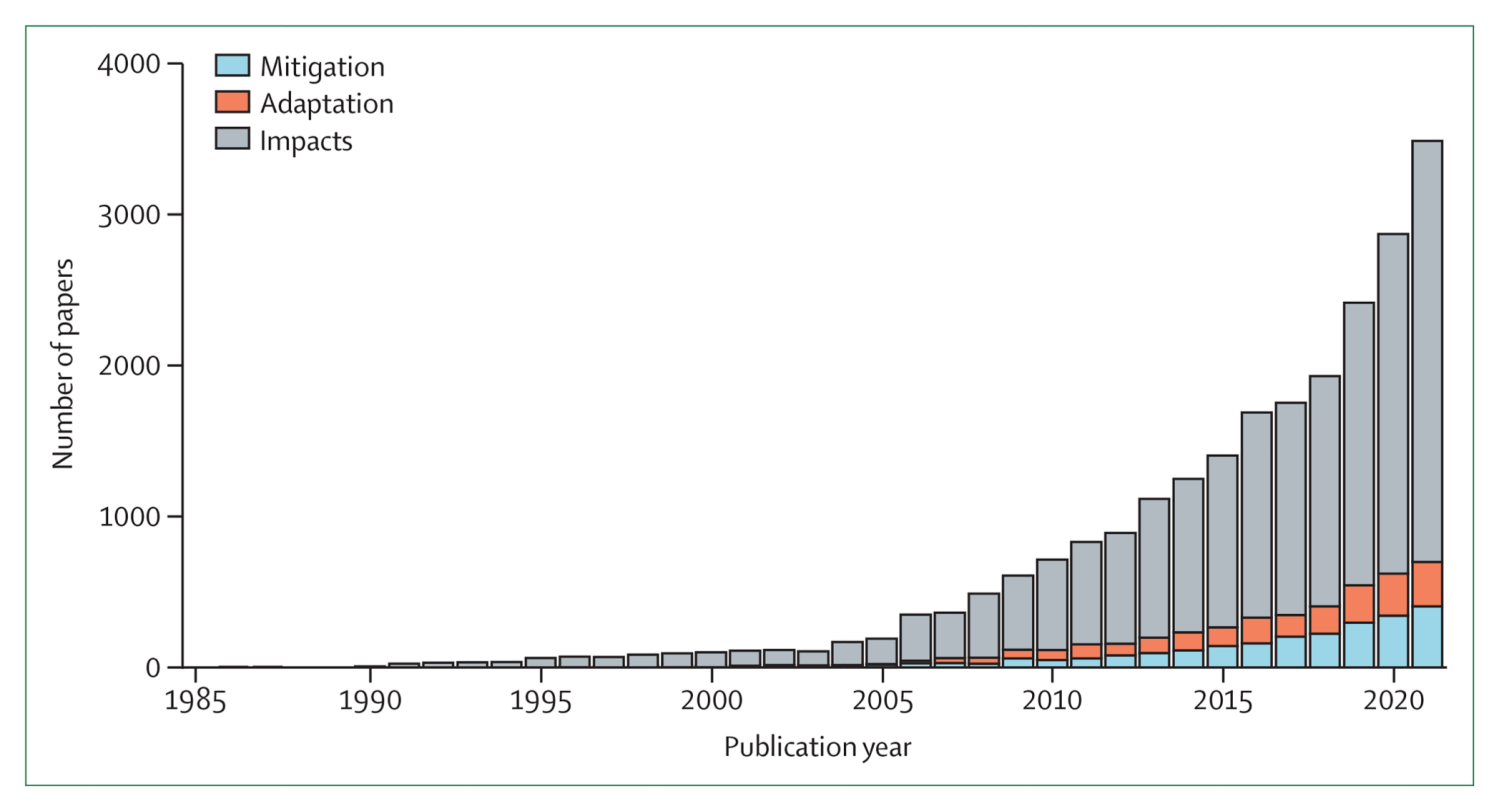
Number of scientific papers on health and climate change, with focus (impacts, mitigation, adaptation) indicated, from 1985 to 2021

In 2021, more than 3200 articles engaged with health and climate change, an increase of 22% compared with 2020 (figure 18). However, this number is a very small proportion of scientific articles on climate change and on climate impacts.^294^ The majority of health and climate change articles were located in, and led by, authors in WHO regions of Western Pacific and the Americas. As research on the health implications of climate change continues to dominate (86% of articles), climate solutions (mitigation and adaptation) are being given increasing attention. 20% of health and climate change articles engaged with pandemic preparedness.

### Indicator 5.4: government engagement in health and climate change—headline finding: the proportion of countries referring to the association between health and climate change increased in both the 2021 UN General Assembly (to 60%) and in updated NDC submissions (to 86%)

Government engagement, essential for climate action,^295^ is tracked by two indicators: the first tracks statements made by national leaders at the UN General Debate (UNGD), at the UN General Assembly, the policy making body of the UN.^296^ The second monitors mentions of health and climate change in NDCs—the major policy instrument set under the Paris Agreement to protect health from “dangerous anthropogenic interference with the climate system”.^297^ Analysis is based on the UNGD text corpus (a database that holds the text transcripts of speeches made at the UNGD),^298^ and on content analysis of the first and the updated NDCs accessed from the UNFCCC interim registry.^299–301^

**Figure 19:**
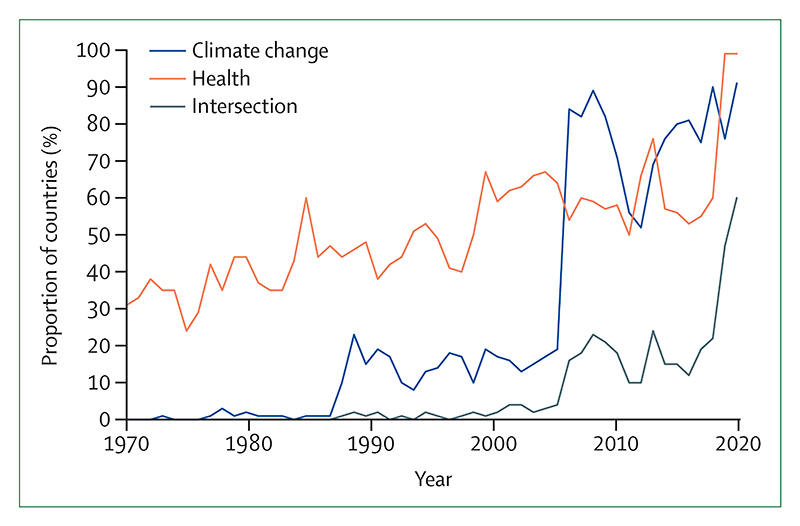
Proportion of countries referring to health and climate change The proportion of countries referring to health, climate change, and the intersection between climate change in the UN General debates from 1970 to 2021 is shown.

In 2021, the proportion of countries referring to the association between health and climate change at the UNGD increased to 60%, its highest recorded level, from 47% in 2020 (figure 19). As in 2020, the COVID-19 pandemic was the main driver for this engagement. St Lucia’s UNGD address noted that “The COVID-19 pandemic and the climate change challenge […] provide us with a harsh and timely reminder that human health and planetary health are linked.”^302^

Countries with low HDI, particularly Small Independent Developing States (SIDS), continue to lead engagement: 76% of SIDS discussed the association between health and climate change in the 2021 UN General Debate. However, increasing engagement with health and climate change is evident in all countries, including those with high and very high HDI.

Increased engagement with health is evident in updated or new NDCs submitted by 126 UN member states (including the one representing 27 EU nations). Of these NDCs, 86% refer to health, an increase from 82% in the first NDCs. The increase is greatest for member states in the high HDI category, which all now refer to health, followed by the very high HDI group (71% made references in the updated NDCs, in an increase from 65% in the first round). The proportions have slightly declined for the medium (87% to 86%) and low (94% to 86%) HDI groups. Most health references are about adaptation needs or efforts (83% of the NDCs mentioned health compared with 87% in the first round), and 40% are also about climate change mitigation (from 18%).

References to the health sector also increased from 74% in the first round to 81% in the second round. Health-care infrastructure was a particular focus, having increased from 39% to 73%. For example, Albania’s second NDC outlines how “health facilities could be damaged by climate-related changes, such as SLR [sea level rise], heavy rains or extreme temperatures”.^303^

### Indicator 5.5: corporate sector engagement in health and climate change—headline finding: engagement in health and climate change increased in 2021 to its highest level among companies in the UN Global Compact, with 38% of corporations referring to the association between health and climate change

The indicator tracks engagement in health and climate change in the annual Communication of Progress (COP) among companies signed up to the UN Global Compact,^304^ the world’s largest corporate sustainability framework operating across 165 countries without restriction by sector or company size.^305,306^ In an improvement from previous iterations, in which only English-language COPs were analysed, COPs in all languages are now included.

Engagement in health and climate change reached its highest level in 2021, with 38% of corporations referring to the association between health and climate change in their COP report. However, as in previous years, there was increased corporate engagement in climate change (87%) and health (72%) as separate issues. Engagement in the association between health and climate change was greatest in companies based in the Western Pacific (53% COPs) and southeast Asia (43%) regions.

## Conclusion

Engagement in health and climate change reached its highest recorded level in 2021, with climate change solutions becoming an increasing focus of health and climate change engagement (eg, in scientific research and the enhanced NDCs). As in previous years, government engagement is led by countries most vulnerable to a climate crisis not of their making.^270,307,308^

As in 2020, the COVID-19 pandemic continues to be a major driver of health and climate change engagement. In the media, a large proportion of English language newspapers engaging with health and climate change referred to the pandemic. The pandemic also drove engagement by individuals and by government leaders in health and climate change. This raises the question of whether increased engagement is contingent on the pandemic context.

Although health and climate change engagement increased in 2021, there is more engagement with health and climate change as separate issues, a pattern evident in individual Wikipedia users’ activities, government leaders’ speeches at the UNGD, and companies reports to the UN Global Compact. Similarly, media and scientific engagement in climate change continues to surpass engagement in health and climate change. Despite mounting evidence of the health burden of climate change, health and climate change have yet to be securely associated in the public, political, and corporate domains that are key to climate action.

## Conclusion: the 2022 report of the *Lancet* Countdown

In its seventh iteration, the 2022 report of the *Lancet* Countdown shows the direst findings yet. At 1·1°C of heating,^73^ climate change is increasingly undermining every pillar of good health and compounding the health impacts of the current COVID-19 pandemic and geopolitical conflicts. The health harms of extreme heat exposure are rising, affecting mental health, undermining the capacity to work and exercise, and resulting in annual heat-related deaths in people older than 65 years increasing by 68% from 2000–04 to 2017–21 (indicators 1.1.1–1.1.5). more frequent and extreme weather events are increasingly affecting physical and mental health directly and indirectly, with economic losses particularly over-burdening low HDI countries, in which losses are mostly uninsured (indicators 1.2.1–1.2.3 and 4.1.1). The changing climate is exacerbating the risk of infectious disease outbreaks (indicator 1.3) and threatening global food security (panel 5), with heatwave days associated with 98 million more people experiencing food insecurity in 2020 than in 1981–2010 (indicator 1.4).

These health impacts add additional pressure on overwhelmed health systems (panel 7). With a further 0·4°C temperature rise probably unavoidable, accelerated adaptation is more urgent than ever. Yet, national and city authorities are not acting fast enough and adaptation funding remains grossly insufficient (indicators 2.1.1, 2.1.2, and 2.2.4). The increased use of air conditioning and scant implementation of nature-based solutions (indicators 2.2.2–2.2.3) reflects a drift towards unplanned, maladaptive responses. Concerningly, and at least partly caused by wealthier countries’ failure to meet climate their finance commitments (panel 8), the adaptation response is often slower in low HDI countries, increasing their vulnerability to a climate crisis that they have had little, if any, contribution to.

Despite these profound health impacts, mitigation efforts remain inadequate to avert a catastrophic temperature rise.^8^ CO_2_ emissions from fuel combustion increased by 6% in 2021 (indicator 3.1) and agricultural greenhouse gas emissions have increased by 31% since 2000 (indicator 3.5.1). The inaction came with major health costs: fossil fuels contributed to 1·3 million deaths from ambient PM_2·5_ exposure in 2020; the over-dependence on solid fuels, worsened by the energy crisis, increased exposure to indoor air pollution (indicators 3.3 and 3.2);^230,231,309^ and consumption of carbon-intensive meat and dairy resulted in 2 million deaths in 2019. Meanwhile, governments provide billions of dollars annually for fossil fuel subsidies (indicator 4.2.4).

However, some indicators provide a glimmer of hope. Government engagement with health and climate change reached record levels in 2021, and the updated NDCs reflect increased awareness of the need to protect health from climate change hazards (indicator 5.4). Renewable electricity generation and electric vehicle use reached record growth, and investments and employment in the clean energy industry are slowly increasing (indicators 3.1, 3.4, 4.2.1, and 4.2.2). If sustained, these shifts could provide energy security, better jobs, cleaner air, and a path for a green COVID-19 recovery. Meanwhile, the health sector is increasingly preparing to face climate hazards (indicator 2.2.1), with 60 countries committing to developing climate-resilient and/or low-carbon or net zero-carbon health systems at COP26.^247^ An expanding number of countries are starting to develop their own observatories, to monitor and identify progress on health and climate change. However, this could come too little too late.

With countries facing multiple crises simultaneously, their policies on COVID-19 recovery and energy sovereignty will have profound, and potentially irreversible consequences for health and climate change. However, accelerated climate action would deliver cascading benefits, with more resilient health, food, and energy systems, and improved security and diplomatic autonomy, minimising the health impact of health shocks. With the world in turmoil, putting human health at the centre of an aligned response to these concurrent crises could represent the last hope of securing a healthier, safer future for all.

## Supplementary Material

Arabic translation of the executive summary

Chinese translation of the Executive Summary

French translation of the executive summary

Spanish translation of the executive summary

Supplementary appendix

## Panel 1 Key findings of the 2022 report of the *Lancet* Countdown

- Climate change is undermining every dimension of global health monitored, increasing the fragility of the global systems that health depends on, and increasing the vulnerability of populations to the coexisting geopolitical, energy, and cost-of-living crises.
- Climate change is increasingly undermining global food security, exacerbating the effects of the COVID-19, geopolitical, energy, and cost-of-living crises. New analysis of 103 countries shows that days of extreme heat, increasing in frequency and intensity due to climate change, accounted for an estimated 98 million more people reporting moderate to severe food insecurity in 2020 than the average in 1981–2010 (indicator 1.4).
- Well-prepared health systems are essential to protect populations from the health impacts of climate change. However, global health systems have been drastically weakened by the effects of the COVID-19 pandemic, and the funds available for climate action decreased in 239 (30%) of 798 cities (indicator 2.1.3), with health systems increasingly being affected by extreme weather events and supply chain disruptions too.
- Insufficient climate change adaptation efforts have left health systems vulnerable to climate change-related health hazards. Only 48 of 95 countries have assessed their climate change adaptation needs (indicator 2.1.1) and only 63% of countries reported high to very high implementation status for health emergency management in 2021 (indicator 2.2.5). Increasing adaptation to climate change has the potential to simultaneously improve the capacity of health systems to manage both future infectious disease outbreaks and other health emergencies (indicator 2.3.1).
- Mitigation of the energy sector is crucial to keep the rise in global mean surface temperatures within the 1·5°C target set in the Paris Agreement. However, the energy sector is still heavily reliant on fossil fuels. Its carbon intensity decreased by less than 1% since the year the UNFCCC was signed, and a simultaneous increase in energy demand of 59% has increased total energy sector emissions to record high levels in 2021 (indicator 3.1). Now, as countries seek alternatives to Russian fossil fuels, many are backsliding to coal, and shifts in global energy supplies risk a net increase in fossil fuel production and consumption.
- The slow adoption of renewable energies, which contribute to only 2.2% of total global energy supply (indicator 3.1), means households remain vulnerable to highly volatile international fossil fuel markets, and millions lack access to reliable, clean sources of fuel. Traditional biomass accounts for 31% of the energy consumed in the domestic sector globally, and for 96% of that in low HDI countries (indicator 3.2). New analysis shows that the air in people’s homes in 62 countries analysed exceeded WHO guidelines for safe concentrations of small particulate air pollution (PM__2·5__) in 2020, by 30-fold on average (indicator 3.2). The current energy and cost-of-living crises, now threatens to worsen enery poverty.
- A new indicator this year reveals that, on the basis of their existing production strategies and market shares, 15 of the largest oil and gas companies would exceed their share of greenhouse gas emissions compatible with the 1·5°C climate target by an average of 87% (publicly-listed international companies) and 111% (state-owned national companies) in 2040 making the goals of the Paris Agreement unattainable (indicator 4.2.6).
- In 2019, 69 (80%) of 86 countries reviewed had net-negative carbon prices (ie, provided a net subsidy to fossil fuels) for a net total of US$400 billion. These subsidies exceeded 10% of national health spending in 31 countries and exceeded 100% in 5 countries (indicator 4.2.4). Meanwhile, climate efforts are being undercut by a profound scarcity of funding (indicator 2.1.1).
- A health-centred response to the coexisting climate, energy, and cost-of-living crises provides an opportunity to deliver a healthy, low-carbon future. Transitioning to clean energy and improved energy efficiency can avert the most catastrophic climate change impacts, while also improving energy security, supporting economic recovery, preventing the 1·2 million annual deaths resulting from exposure to fossil fuel-derived ambient PM__2·5__ (indicator 3.3), and improving health outcomes by promoting active forms of travel for greener cities. The associated reduction in the burden of disease will in turn reduce the strain on overwhelmed healthcare providers, and enable better care.
- The media, the scientific community, corporations, and country leaders are increasingly engaging in health and climate change (indicators 5.1–5.5), and new analysis shows that 86% of updated or new Nationally Determined Contributions now reference health (indicator 5.4).
- Countries are attempting to cut their dependence on international oil and gas supplies in response to the war in Ukraine and energy crisis, with some focusing on increasing renewable energy generation, raising hopes that a health-centred response could be emerging. However, the increased engagement and commitments must be urgently translated into action for hope to turn into reality.

## Panel 2 The indicators of the 2022 report of the *Lancet* Countdown

### Health hazards, exposures, and impacts

1.1 Health and heat
  1.1.1 Exposure to warming
  1.1.2 Exposure of vulnerable populations to heatwaves
  1.1.3 Heat and physical activity
  1.1.4 Change in labour capacity
  1.1.5 Heat-related mortality
1.2 Health and extreme weather events
  1.2.1 Wildfires
  1.2.2 Drought
  1.2.3 Extreme weather and sentiment
1.3 Climate suitability for infectious disease transmission
1.4 Food security and undernutrition

### Adaptation, planning, and resilience for health

2.1 Assessment and planning of health adaptation
  2.1.1 National assessments of climate change impacts, vulnerability, and adaptation for health
  2.1.2 National adaptation plans for health
  2.1.3 City-level climate change risk assessments
2.2 Enabling conditions, adaptation delivery, and implementation
  2.2.1 Climate information for health
  2.2.2 Air conditioning: benefits and harms
  2.2.3 Urban greenspace
  2.2.4 Health adaptation-related funding
  2.2.5 Detection, preparedness, and response to health emergencies
2.3 Vulnerabilities, health risk, and resilience to climate change
  2.3.1 Vulnerability to mosquito-borne disease
  2.3.2 Lethality of extreme weather events
  2.3.3 Migration, displacement, and rising sea levels

### Mitigation actions and health co-benefits

3.1 Energy system and health
3.2 Clean household energy
3.3 Premature mortality from ambient air pollution by sector
3.4 Sustainable and healthy transport
3.5 Food, agriculture, and health
  3.5.1 Emissions from agricultural production and consumption
  3.5.2 Diet and health co-benefits
3.6 Health-care sector emissions

### Economics and finance

4.1 Economic impact of climate change and its mitigation
  4.1.1 Economic losses due to climate-related extreme events
  4.1.2 Costs of heat-related mortality
  4.1.3 Loss of earnings from heat-related labour capacity loss
  4.1.4 Costs of the health impacts of air pollution
4.2 Economics of the transition to zero-carbon economies
  4.2.1 Clean energy investment
  4.2.2 Employment in low-carbon and high-carbon industries
  4.2.3 Funds divested from fossil fuels
  4.2.4 Net value of fossil fuel subsidies and carbon prices
  4.2.5 Production-based and consumption-based attribution of CO__2__ and PM__2·5__ emissions
  4.2.6 Compatibility of fossil fuel company strategies with the Paris Agreement

### Public and political engagement

5.1 Media coverage of health and climate change
5.2 Individual engagement in health and climate change
5.3 Scientific engagement in health and climate change
5.4 Government engagement in health and climate change
5.5 Corporate sector engagement in health and climate change

## Panel 3 Detection and attribution studies: the influence of climate change in health-harming extreme events

Detection and attribution studies are increasingly highlighting the influence of climate change on weather-related morbidity and mortality and are being applied in public health to inform decision making.^34^ However, only a small proportion of all extreme events that occur are being assessed, with few studies on the events that affect the highly vulnerable low or middle HDI countries. Expanding the coverage and funding available for detection and attribution studies and strengthening their health assessment can help to elucidate the health costs of climate change and provide compelling evidence to support climate action.^35,36^

Detection and attribution studies were published for 31 discrete weather-related events occurring between 2019 and 2021. All except two of the analysed events occurred in high or very high HDI countries. Detection and attribution studies were published for extreme heat, heavy precipitation and floods, wildfires, storms, tornadoes, cyclones, or drought events. Climate change was shown to have increased the likelihood or severity of 84% of these events (24 studies), in which more than 113 300 deaths were registered. All but one of the nine extreme heat events studied, which caused 13 480 deaths, were found to have been made more likely or more intense because of climate change. Climate change decreased the likelihood or severity of just three events, which were all linked to extreme rainfall events, reflecting the climate-induced alteration of hydrological cycles. Most of the events studied had cascading effects on health systems, and most were compounded by concurrent crises. Some key examples are detailed below, while the full list of events is available in the appendix 5 (p 20).

### Australia’s Black Summer

Australia’s 2019–20 bushfires (sometimes referred to as Australia’s Black Summer) were unprecedented in scale, intensity, and the extent of damage caused. Anthropogenic climate change increased the probability of bushfires by more than 30%.^37,38^ The fires directly caused some 450 deaths, 1300 emergency asthma presentations, and 1120 cardiovascular and 2030 respiratory admissions,^39^ in addition to worsening mental health outcomes and displacing 47 000 people.^40–42^ These bushfires contributed to 715 megatonnes of CO__2__ emissions, equivalent to around 0·2% of global greenhouse gas emissions that year.^43^

### South African drought

Between 2015 and 2019, South Africa’s Western Cape record drought was two to nine times more likely to happen because of climate change.^44,45^ In a neighbouring rural region, the drought restricted the provision of and access to HIV care, resulting in restricted treatment for patients.^46^ Although health data were limited, vulnerable populations were likely to be disproportionately exposed to the drought, resulting in adverse health outcomes,^47^ including mental health.^48^

### Floods in western Europe

In July 2021, north-western Europe was exposed to devastating floods, primarily driven by heavy rainfall that was 1·2 to 9 times more likely to happen because of climate change.^49^ The floods directly killed more than 200 people in Europe.^50,51^ Health was also impacted as a result of damage to pharmacies, hospitals, and clinics; scarce potable water; destruction of sewerage infrastructure; and disruption of health-care services, including the administration of COVID-19 vaccines.^50,52^

### North American heat dome

In June–July, 2021, northwest North America experienced a 6-day heatwave that was at least 150 times more likely to have happened because of climate change and was “virtually impossible” without it,^53^ directly causing at least 569 excess deaths in British Columbia, Canada, and more than 100 deaths in Washington, USA.^54,55^ Material deprivation and reduced access to urban green spaces were found to have increased mortality risk.^56,57^ Alaska, Idaho, Oregon, and Washington, USA, registered more than 1000 heat-related emergency service presentations, a 69-fold increase in the same period the year before.^58^

### South Asian heatwave

During March–April, 2022, India and Pakistan experienced a heatwave that was 30 times more likely to have happened because of climate change.^2^ Despite widespread underreporting, 90 deaths were attributed^3^ with reduced wheat yields that have further worsened global shortages caused by the war in Ukraine. The full health impacts of lost income, increased hospitalisations, and food and energy insecurity, in addition to the outburst flood of a glacial lake and forest fires, are not yet quantified.^2^

## Panel 4 Mental health and climate change

Climate change is affecting mental health, psychological wellbeing, and their social and environmental determinants.^81–85^ Acute temperature increase, heatwaves, and humidity have been associated with worsened mental health outcomes and increased suicidality.^86,87^ Through indirect pathways, hazards such as droughts can disrupt agricultural production, affect livelihoods, and cause food and water scarcity and other hardships that affect family relationships, increase stress, and negatively impact mental health, with differences between genders.^88–90^ Climate change can also exacerbate conflict and violence (including gender-based violence),^91–93^ and can influence people’s decision to migrate, which can in turn affect mental health and wellbeing.^94^ Climate change can also impact the mental health of populations who either choose to stay or are unable to migrate, with studies showing that mental health can be compromised by the feeling of being trapped.^95–97^

Marginalised and vulnerable populations are often disproportionately affected by mental health impacts related to climate change, which can worsen pre-existing mental health inequalities, especially where health care is inadequate. Indigenous people may be more strongly affected by climate change-induced ecological breakdown.^98,99^ Older people, women, and religious or ethnic minorities are particularly at risk of adverse mental health outcomes, and young people have been shown to be more prone to anxiety, phobias, depression, stress-related conditions, substance abuse, sleep disorders, reduced capacity to regulate emotions, and increased cognitive deficits.^100^ The increasingly visible effects of the climate crisis have given rise to emerging concepts, such as climate change anxiety, solastalgia, eco-anxiety, and ecological grief.

Integrating mental health considerations within adaptation, mitigation, and disaster risk reduction (DRR) efforts could both reduce climate change-related mental health risks and deliver mental health co-benefits. Actions to reduce heat and ambient air pollution through urban redesign, such as improved shade and green space, walkable neighbourhoods, and improved active and public transport infrastructure, can provide mental health co-benefits by promoting physical activity, better sleep quality, increased social connectivity, providing more cooling spaces, and increasing exposure to greenness.^101,102^ Climate activism can be associated with increased mental wellbeing,^103^ although it might increase distress for others,^104^ which emphasises the importance of including mental health considerations when designing climate policies. Yet, despite multiple associations between climate change and poorer mental health, few National Adaptation Plans (7/18 documents assessed by WHO) and Nationally Determined Contributions (10/197 documents representing 9/197 parties assessed by Climate Watch) consider mental health and psychosocial implications.^105^ Only 28% of countries report having a functional programme that integrates mental health and psychosocial support within preparedness and disaster risk reduction, including for climate-related hazards.^106^

The persistent lack of standardised definitions, stigmatisation and lack of recognition of mental health in many places, and scarcity of data on mental health impacts and care hinders the capacity to identify populations at risk and develop targeted resilience strategies, to monitor and assess the mental health implications of climate change and climate action, and ultimately to develop mental health indicators.^107–110^

Nonetheless, the world has sufficient experience and evidence to guide immediate action. Rapidly accelerating efforts to address the impacts of climate change on mental health and psychosocial wellbeing is essential to protect all dimensions of human health.^108^

## Panel 5 Climate change and food insecurity

Food security requires people to have physical and economic access to sufficient, safe, and nutritious food that meets their dietary needs and food preferences for an active and healthy life, at all times.^135,136^

In 2015, the world committed to ending malnutrition and achieving global food security by 2030 (SDG2).^137^ However, the prevalence of undernourishment has increased since 2017.^138^ Government-imposed restrictions during the COVID-19 pandemic worsened global malnutrition,^139^ with the number of undernourished people increasing by 161 million to 720–811 million between 2019 and 2020.^138^ Russia’s war on Ukraine is further exacerbating food insecurity: Russia and Ukraine typically supply around 30% of global wheat exports, and 20% of maize, and the expected shortfall in supply, coupled with the energy crisis, is likely to drive further increases in food prices. This could result in an additional 7·6–13·1 million people undernourished globally in 2022. Meanwhile, conflict in places, such as Afghanistan, Burkina Faso, Chad, Democratic Republic of Congo, Ethiopia, Nigeria, Mozambique, Myanmar, Syria, Mali, Niger, and South Sudan, further worsens the food crises in those regions.^140^

This panel details how climate change affects each dimension of global food security and nutrition, and highlights priorities for climate action, providing a cross-cutting assessment of the evidence presented in this report.

### Food availability, access, and stability

Climate change is putting food production, supply chains, and access at risk. Rising temperatures are reducing crop growth duration (indicator 1.4) in many countries, posing a threat to crop yields. The increasing intensity and frequency of extreme weather events, including heatwaves (indicator 1.1.2), droughts (indicator 1.2.2), and wildfires (indicator 1.2.1), can damage crops and agricultural lands, affect livestock, disrupt supply chains, and affect food availability and stability of supplies.^141,142^ Changing environmental conditions affect the spread of crop and livestock pests and diseases, resulting in production losses.^143,144^ Increasing water temperatures and ocean acidification threaten fish stocks, thereby undermining marine food supplies (indicator 1.4), and rising sea levels and sea water intrusion can lead to soil salinisation and crop losses.^145–148^ Exposure to high temperatures and extreme weather events reduces labour capacity, and 65% of all potential hours of labour lost globally occurred in the agricultural sector, with agricultural workers in low and medium HDI countries disproportionately affected (indicator 1.1.4). More broadly, reduced labour capacity can result in lower incomes (indicator 4.1.3), and extreme events can lead to direct economic damages, particularly in LMICs where most losses are not insured (indicator 4.1.1). The resulting economic losses can contribute to reduced purchasing power, undermining food access.

### Food use and malnutrition

Diarrhoeal diseases are the leading cause of malnutrition in children younger than 5 years,^149^ while other infections can severely affect nutrient absorption and utilisation.^150–152^ Climate change therefore increases the risk of malnutrition by increasing the transmission risk of many infectious diseases, such as malaria, dengue, and vibriosis (indicator 1.3), while the increasing incidence of floods, droughts, and other extreme events affects sanitation and disease outbreaks (indicator 1.1.2). Although increasing atmospheric CO_2_ concentration may increase crop yields via the fertilisation effect, it might also reduce the nutritional quality of some grains,^153^ and rising sea levels can increase the salinity of the soils and water supplies (indicator 2.3.3), leading to unhealthy levels of sodium in diets.^154^

### Mitigation, adaptation planning, and resilience for health

Addressing threats to food insecurity requires coordinated and robust action across multiple sectors of governments and societies. There are some signs of progress in this respect: while 10% of the first NDCs made reference to this issue, the proportion increased to 17% in the second NDCs updated from January 2020 to April 2022 (indicator 5.4). 49% of cities identified climate-related risks to food and agriculture assets and services in 2020 (indicator 2.1.2).

Shifting to low-carbon, plant-forward diets would have the multiple benefits of reducing agricultural greenhouse gas emissions (indicator 3.5.1), improving health outcomes (indicator 3.5.2), reducing the diversion of grains to livestock and the demand of land for crop production, water demand, and the risk of agriculture-related zoonotic disease outbreaks.^155,156^ Interventions to increase the resilience of food systems, and improve sanitation and health care can minimise climate-related nutritional risks. Interventions include proactive safety nets, nudge programmes that encourage savings, and mother and child feeding programmes.^157^ Investment in sustainable irrigation methods,^158–160^ drought-resistant crops,^161^ financial support for smallholder agriculture,^162,163^ regional crop storage,^164^ insurance or reinsurance, and early warning systems for extreme weather events that might damage crops, or increase infectious disease transmission, are each likely to be important in specific contexts.

## Panel 6 Heat adaptation strategies with sustainable low-energy cooling

Of all natural disasters, heatwaves cause the most deaths,^178^ with older adults, people with cardiovascular disease, people living with poverty, and people isolated in low-cost housing most at risk.^179^ Air-conditioning can offer effective protection but is expensive and thus inaccessible to many.^169,180^ Peak energy demands from widespread use of air conditioning can overwhelm energy systems and result in electricity blackouts and brownouts, particularly in places that have frail energy infrastructure or limited resources, worsening health impacts.^181^ As more people use air conditioning worldwide, the soaring electricity demand hinders low-energy transition, and contributes to increased greenhouse gas emissions (indicator 3.1)^182^ through the generation of waste heat, air conditioning also intensifies urban heat, and contributes to increased exposure to air pollution (indicator 2.2.2). Sustainable and affordable cooling alternatives are therefore urgently needed.

Modifications in the landscape and built environment can provide local cooling benefits. Water bodies act as heat sinks, and vegetation provides shading and cooling by evapotranspiration.^183^ Reflective roofing and better building insulation can attenuate heat transfer to the individual.^180^ However, such interventions can require long-term changes to urban or regional infrastructure. Alternatively, heat resilience in the immediate-term can be built through the use of low-resource and sustainable cooling behaviours at the personal level. The use of window blinds can help reduce indoor temperatures by blocking solar radiation. If air conditioning is available, moving indoor air with fans increases the upper temperature of thermal discomfort by 3–4oC, allowing for air conditioning to be set at a higher temperature,^184^ reducing the energy demand of air conditioning.^185^ Electric fans provide effective cooling up to at least 40°C for resting and active young adults,^186,187^ and to around 38°C for resting older adults, and use 30 times less energy than air conditioning.^188^ However, in very hot, dry conditions (>45°C with <15% relative humidity), fans should be used with extreme caution because they can worsen physiological heat strain and dehydration;^187^ an effect likely aggravated in older adults. Evaporative coolers, depending on their size, use 2–5 times less energy than air conditioning, and can reduce indoor temperatures by 5–10°C in dry weather (<20–30% relative humidity). However, they are inefficient cooling devices in high humidity (>50–60% relative humidity), unless used with a dehumidifier,^189^ and are dependent on reliable water supply. If electricity is unavailable, including during power blackouts, studies suggest cooling by frequently wetting large skin areas, which can reduce physiological heat strain and improve thermal comfort up to at least 47°C.^190^ Wearing lightweight, water-soaked clothing provides similar benefits up to at least 43°C.^191^ Immersing both feet in cool water (<20°C) for 10 min every 20 min might reduce dehydration and improve thermal comfort up to at least 47°C.^190^ Putting both hands or arms to the elbow in 10°C water can reduce core temperature rises at air temperatures up to 40°C.^192^

Public health campaigns promoting these evidence-based sustainable cooling strategies in advance of and during bouts of extreme heat will not only help reduce energy demand and energy poverty, but also reduce the risk of heat-related morbidity and mortality and help build resilience against rising global temperatures.

## Panel 7 Health care, COVID-19, and climate change

The COVID-19 pandemic has greatly altered patterns of health-care use in countries worldwide, and subsequently their health-care-associated emissions. Many health systems faced major increases in expenditures on personal protective equipment (PPE), diagnostic testing, and provision of critical care, but also had decreases in non-COVID-19 essential and elective care. As a result, greenhouse gas emissions from health care are expected to have shifted substantially in 2020–21, perhaps even decreasing in some countries. Although reducing inefficient health-care usage and associated emissions is a goal of climate change mitigation efforts within health systems, this should not happen at the expense of deferring or avoiding necessary care. Measures of progress for decarbonising the health sector need to focus on both optimal health and reduced greenhouse gas emissions. The pandemic has highlighted risks associated with the health-care sector’s sprawling supply chains, including widespread shortages of basic medicines, equipment, and PPE. Leading health systems should simultaneously focus on reducing these supply chain risks and mitigating greenhouse gas emissions. COP26 resulted in historic commitments by 60 countries thus far, to develop climate-resilient and/or low-carbon or net zero-carbon health systems,^247^ and many are beginning to implement and share best practices that both improve resilience and reduce life-cycle greenhouse gas emissions.^248^

## Panel 8 Financing the response to compounding crises

The economic benefits of keeping temperatures lower than 1·5°C of heating and minimising climate change impacts through accelerated adaptation are expected to outweigh the costs of climate action.^8^ While delivering the needed transition to net-zero emissions will require substantial capital investment,^249^ the money is available. However, it is concentrated in relatively few economies that bear much of the historic responsibility for human-caused climate change, have only moderate direct and immediate geographic vulnerability to climate change, and can most afford to decarbonise and adapt.^266^ Conversely, the lower income countries that have contributed the least to cumulative CO__2__ emissions generally are more vulnerable to climate hazards, and have fewer resources to decarbonise, adapt, and recover from climate-related impacts. In acknowledgement of this, in the 2009 Copenhagen Accord “developed countries commit[ted] to a goal of mobilising jointly US$100 billion dollars a year by 2020 to address the needs of developing countries”.^267^ To date, only $79 billion has been committed,^249^ two thirds of this being in the form of loans, with most of the remainder evenly split between public grants and private finance.^249^ At COP26, it was acknowledged that the $100 billion target would not be met until 2023—^268^a delay that not only jeopardises mitigation goals, but also leaves lower income countries more vulnerable to exacerbated climate change-related loss and damage. The economic impacts of COVID-19 and geopolitical conflicts put the $100 billion target further out of reach.

This failure to meet international funding goals is in stark contrast with how countries have responded to the COVID-19 pandemic, with more than $15 trillion for rescue spending by governments announced globally during 2020 and 2021, and a further $3·11 trillion pledged for recovery spending (concentrated heavily in OECD countries, plus China). Although $92 billion was pledged to improve future pandemic preparedness, and could increase the capacity to manage future climate health hazards,^269^ the net effect of recovery spending is likely to worsen climate change-related health outcomes: less than $1 trillion was allocated to purposes that are likely to reduce greenhouse gas emissions or air pollution, and the net effect of recovery spending is likely to result in increased emissions through direct or indirect investment in carbon-intensive activities.^269^

The COVID-19 response demonstrated the extent to which decision makers in higher income countries are willing and able to rapidly raise and allocate vast sums of public money to tackle what they perceive as a clear and present danger to the health of their population and economy. The paucity of international climate finance reveals a concerning finding: despite the extensive evidence on the unprecedented short-term and long-term dangers of climate change, and the cost-effectiveness of climate action, climate change is not yet viewed as a crisis by the decision makers who could most effectively address it. However, the capacity to mobilise the necessary resources is clear. With the window of opportunity for keeping temperatures below 1·5°C rapidly closing, averting the catastrophic health impacts of climate change depends now on political will.
