# Supplementary material for: The 2022 report of the Lancet Countdown on health and climate change: health at the mercy of fossil fuels
Source: Lancet. Author manuscript; Available in PMC 2024 Nov 15. (PMC7616806; doi:10.1016/S0140-6736(22)01540-9)
Supplement: Arabic translation of the executive summary [file EMS200004-supplement-Arabic_translation_of_the_executive_summary.pdf]

THE LANCET

Supplementary appendix 4

This translation in Arabic was submitted by the authors and we reproduce it as supplied. It has not been peer reviewed. *The Lancet*'s editorial processes have only been applied to the original in English, which should serve as reference for this manuscript.

تم تقديم هذه الترجمة باللغة العربية من قبل المؤلفين ونعيد إنتاجها كما هو مُقدم. إنها لم تخضع لاستعراض الأقران. تم تطبيق عمليات تحرير *لانسيت* فقط على النص الأصلي باللغة الإنجليزية، والذي يجب أن يكون بمثابة مرجع لهذه المخطوطة.

Supplement to: Romanello M, Di Napoli C,Drummond P, et al. The 2022 report of the *Lancet* Countdown on health and climate change: health at the mercy of fossil fuels. *Lancet* 2022; published online Oct 25. https://doi.org/10.1016/S0140-6736(22)01540-9.

تقرير العد التنازلي 2022 لمجلة "ذا لانسيت" بخصوص الصحة وتغير المناخ: الصحة تحت رحمة الوقود الأحفوري

ملخص تنفيذي

يأتي نشر تقرير انسيت العد التنازلي لمجلة "ذا لانسيت" لعام 2022 في الوقت الذي يواجه فيه العالم صدمات عميقة ومتزامنة، حيث لا تزال البلدان والأنظمة الصحية تواصل جهودها لمواجهة الآثار الصحية والاجتماعية والاقتصادية لوباء كوفيد-19، في حين دفع الغزو الروسي لأوكرانيا والاعتماد المفرط المستمر على الوقود الأحفوري العالم إلى أزمة عالمية في الطاقة وتكلفة المعيشة. ومع تكشف هذه الأزمات، يتصاعد تغير المناخ بلا هوادة، وتؤثر آثاره المتفاقمة على نحو متزايد على أسس صحة الإنسان ورفاهه، مما يتسبب في تفاقم هشاشة سكان العالم أمام تهديدات صحية متزامنة.

فخلال عامي 2021 و2022، تسببت الأحوال الجوية المتطرفة في حدوث دمار في كل قارة، مما زاد من الضغط على الخدمات الصحية التي تعاني بالفعل جراء آثار جائحة كوفيد-19. فقد تسببت الفيضانات في كل من أستراليا والبرازيل والصين وأوروبا الغربية وماليزيا وباكستان وجنوب إفريقيا وجنوب السودان في مقتل آلاف الأشخاص وتشريد مئات الآلاف الآخرين وتسببت في خسائر اقتصادية بمليارات الدولارات. كما تسببت حرائق الغابات في دمار كبير في كندا والولايات المتحدة الأمريكية واليونان والجزائر وإيطاليا وإسبانيا وتركيا، وسجلت درجات حرارة قياسية في العديد من البلدان، بما في ذلك أستراليا وكندا والهند وإيطاليا وعمان وتركيا وباكستان والمملكة المتحدة. ومع التقدم في علم دراسات الكشف والإسناد، يمكن الآن قياس تأثير تغير المناخ على العديد من الأحداث.

بفعل الارتفاع السريع في درجات الحرارة، تعرض الضعفاء من السكان (البالغون الذين تزيد أعمارهم عن 65 عامًا، والأطفال الذين تقل أعمارهم عن عام واحد) إلى زيادة 3.7 مليار يوم من موجات الحر في عام 2021 مقارنةً سنويًا مع الفترة 1986-2005 (المؤشر 1.1.2)، وزادت الوفيات المرتبطة بالحرارة بنسبة 68٪ بين 2000-2004 و2017-2021 (المؤشر 1.1.5)، وهو عدد الوفيات الذي تفاقم كثيراً بسبب الاقتران بجائحة كوفيد-19.

في الوقت نفسه، يؤثر المناخ المتغير على انتشار الأمراض المعدية، مما يعرض السكان لخطر الإصابة بالأمراض الناشئة والأوبئة المشتركة. فقد أصبحت المياه الساحلية أكثر ملاءمة لنقل مسببات الأمراض؛ إذ زاد عدد الأشهر المناسبة لانتقال الملاريا بنسبة 31·3٪ في مناطق المرتفعات في الأمريكيتين و 13.8٪ في مناطق المرتفعات بأفريقيا من 1951-60 إلى 2012–21، وارتفع احتمال انتقال حمى الضنك بمقدار 12 ٪ في نفس الفترة (المؤشر 1.3.1). وأدى تلاقي تفشيات حمى الضنك مع وباء كوفيد-19 إلى زيادة الضغط على النظم الصحية، والتشخيص الخاطئ، والصعوبات في إدارة كلا المرضين في العديد من مناطق أمريكا الجنوبية وآسيا وأفريقيا.

تؤدي الخسائر الاقتصادية المرتبطة بتأثيرات تغير المناخ أيضًا إلى زيادة الضغط على الأسر والاقتصادات التي تواجه بالفعل تحديات مع الآثار المجتمعة لوباء كوفيد-19 وتكلفة المعيشة وأزمات الطاقة الدولية، مما يزيد من تقويض المحددات الاجتماعية والاقتصادية التي تعتمد عليها الصحة الجيدة. كما أدى التعرض للحرارة إلى فقدان 470 مليار ساعة عمل محتملة على مستوى العالم في عام 2021 (المؤشر 1.1.4)، مع خسائر محتملة في الدخل تعادل 0·72٪ من الناتج الاقتصادي العالمي، وزيادة إلى 5·6٪ من الناتج المحلي الإجمالي في مؤشر التنمية البشرية المنخفض (مؤشر التنمية البشرية)، حيث يكون العمال أكثر عرضة لآثار التقلبات المالية (المؤشر 4.1.3). وفي الوقت نفسه، تسببت الظواهر الجوية المتطرفة في أضرار بلغت قيمتها 253 مليار دولار أمريكي في عام 2021، لا سيما في البلدان ذات مؤشر التنمية البشرية المنخفض حيث لم يتم التأمين على أي من الخسائر تقريبًا (المؤشر 4.1.1).

بمسارات متعددة ومترابطة، يتأثر كل بُعد من أبعاد الأمن الغذائي بتغير المناخ، مما يؤدي إلى تفاقم آثار الأزمات الأخرى المتعايشة. حيث تهدد درجات الحرارة المرتفعة غلة المحاصيل بشكل مباشر، مع مواسم نمو الذرة في المتوسط 9 أيام أقصر في 2020، ومواسم نمو القمح الشتوي والقمح الربيعي 6 أيام أقصر من 1981-2010 على الصعيد العالمي (المؤشر 1.4). يضيف التهديد الذي تتعرض له غلات المحاصيل إلى التأثير المتزايد للطقس القاسي على سلاسل التوريد، والضغوط الاجتماعية والاقتصادية، وخطر انتقال الأمراض المعدية، مما يقوض توافر الغذاء والوصول إليه واستقراره واستخدامه. يشير تحليل جديد إلى أن الحرارة الشديدة ارتبطت بزيادة 98 مليون شخص أفادوا بانعدام أمنهم الغذائي على نحو معتدل إلى شديد في 2020 مقارنة بالسنة في 1981-2010، في 103 بلدان تم تحليلها (المؤشر 1.4). يؤدي الطقس المتطرف بشكل متزايد إلى تفاقم استقرار النظم الغذائية العالمية، حيث يعمل بالتآزر مع الأزمات المتزامنة الأخرى لعكس مسار التقدم نحو القضاء على الجوع. في الواقع، ازداد انتشار نقص التغذية خلال جائحة كوفيد19 وواجه ما يصل إلى 161 مليون شخص الجوع خلال الجائحة في عام 2020 مقارنة بعام 2019. وقد تفاقم هذا الوضع الآن بسبب الغزو الروسي لأوكرانيا وأزمات الطاقة والتكلفة. المعيشية، مع آثارها على الإنتاج الزراعي الدولي وسلاسل التوريد التي تهدد بالتسبب في 13 مليون شخص إضافي يواجهون نقص التغذية في عام 2022.

خط دفاع أول منهك

مع تفاقم الآثار الصحية لتغير المناخ التي تؤدي إلى تفاقم الأزمات الأخرى المتعايشة، يعتمد السكان في جميع أنحاء العالم بشكل متزايد على النظم الصحية كخط دفاعهم الأول؛ لكن، مع ازدياد الحاجة إلى الرعاية الصحية، أصبحت هذه الأنظمة الصحية منهكة عبر العالم بسبب آثار جائحة كوفيد-19 وأزمات الطاقة وتكلفة المعيشة. لذلك، هناك حاجة إلى اتخاذ إجراءات عاجلة لتعزيز صمود النظام الصحي والحيلولة دون الخسائر السريعة في الأرواح والمعاناة في ظل مناخ متغير. ومع ذلك، أبلغ 48 (51٪) فقط من 95 دولة عن تقييم احتياجات التكيف مع تغير المناخ (المؤشر 2.1.1)، وحتى بعد الآثار العميقة لكوفيد-19، أبلغ 60 (63٪) فقط عن وضع تنفيذي مرتفع إلى مرتفع جدًا لإدارة الطوارئ الصحية في عام 2021 (المؤشر 2-2-4).

تبدو ندرة التكيف الاستباقي في الاستجابة للحرارة الشديدة. فعلى الرغم من التبريد المحلي والفوائد الصحية العامة للمساحات الخضراء الحضرية، إلا أن 277 (27٪) فقط من 1038 مركزًا حضريًا عالميًا كانت خضراء بشكل معتدل على الأقل في عام 2021 (المؤشر 2.2.3)، وزاد عدد الأسر المجهزة بتكييف الهواء بنسبة 66٪ من 2000 إلى 2020، وهي استجابة عسيرة التكيف تؤدي إلى تفاقم أزمة الطاقة وزيادة الحرارة الحضرية وتلوث الهواء وانبعاثات غازات الاحتباس الحراري.

نظرًا لأن الأزمات المتقاربة تهدد الأنظمة الداعمة للحياة في العالم، فإن العمل المشترك السريع والحاسم والمتسق بين القطاعات ضروري لحماية صحة الإنسان من مخاطر المناخ سريع التغير.

الصحة تحت رحمة الوقود الأحفوري

يصادف عام 2022 الذكرى الثلاثين لتوقيع اتفاقية الأمم المتحدة الإطارية بشأن تغير المناخ، والتي اتفقت فيها الدول على منع تغير المناخ الخطير الذي يتسبب فيه الإنسان وآثاره الضارة على صحة الإنسان ورفاهيته. ومع ذلك، لم يتبع ذلك سوى القليل من الإجراءات الهادفة. فقد انخفضت كثافة الكربون في نظام الطاقة العالمي بنسبة تقل عن 1٪ منذ توقيع اتفاقية الأمم المتحدة الإطارية بشأن تغير المناخ، ولا يزال الوقود الأحفوري يهيمن على توليد الكهرباء على الصعيد العالمي، حيث تساهم الطاقة المتجددة بنسبة 8·2% فقط من الإجمالي العالمي (المؤشر 3.1) . في الوقت نفسه، ارتفع إجمالي الطلب على الطاقة بنسبة 59٪، مما أدى إلى زيادة الانبعاثات المرتبطة بالطاقة إلى مستوى مرتفع تاريخي في عام 2021. تضع السياسات الحالية العالم على المسار نحو زيادة كارثية تبلغ 2·7 درجة مئوية بحلول نهاية القرن. حتى مع الالتزامات التي حددتها البلدان في المساهمات المحددة وطنياً (NDCs) المحدثة حتى نوفمبر 2021، يمكن أن تزيد الانبعاثات العالمية بنسبة 13.7٪ عن مستويات عام 2010 بحلول عام 2030 - بعيدًا عن الانخفاض بنسبة 43٪ عن المستويات الحالية المطلوبة لتحقيق أهداف اتفاقية باريس، والحفاظ على درجات الحرارة في حدود التكيف.

لا يؤدي الاعتماد على الوقود الأحفوري إلى تقويض الصحة العالمية من خلال زيادة تأثيرات تغير المناخ فحسب، بل يؤثر أيضًا على صحة الإنسان ورفاهيته بشكل مباشر، من خلال أسواق الوقود الأحفوري المتقلبة وغير المتوقعة، وسلاسل التوريد الهشة، والصراعات الجيوسياسية. نتيجة لذلك، لا يستطيع ملايين الأشخاص الوصول إلى الطاقة اللازمة للحفاظ على منازلهم في درجات حرارة صحية، والحفاظ على الغذاء والدواء، وتحقيق الهدف السابع من أهداف التنمية المستدامة (لضمان الوصول إلى طاقة حديثة وموثوقة ومستدامة وبأسعار معقولة من أجل الجميع). بدون دعم كافٍ، أصبح الوصول إلى الطاقة النظيفة بطيئاً خصوصاً في البلدان ذات مؤشر التنمية البشرية المنخفض، وجاء 1·4٪ فقط من كهربائهم من مصادر الطاقة المتجددة الحديثة (معظمها طاقة الرياح والطاقة الشمسية) في عام 2020 (المؤشر 3.1). ولا يزال ما يقدر بنحو 59٪ من مرافق الرعاية الصحية في البلدان المنخفضة والمتوسطة الدخل لا تحصل على الكهرباء الموثوقة اللازمة لتوفير الرعاية الأساسية. وفي الوقت نفسه، تمثل الكتلة الحيوية ما يصل إلى 31٪ من الطاقة المستهلكة في القطاع المحلي على مستوى العالم، ومعظمها من المصادر التقليدية - وهي نسبة تزيد إلى 96٪ في البلدان ذات مؤشر التنمية البشرية المنخفض (المؤشر 3.2). إن عبء الأمراض المرتبطة به كبير، حيث تجاوز الهواء في منازل الأشخاص إرشادات منظمة الصحة العالمية للتركيزات الآمنة لجسيمات تلوث الهواء الصغيرة (PM2.5) في عام 2020 بمقدار 30 ضعفًا في المتوسط في 62 دولة تم تقييمها (المؤشر 3.2). بعد 6 سنوات من التحسين، ارتفع عدد الأشخاص المحرومين من الكهرباء في عام 2020 نتيجة للضغوط الاجتماعية والاقتصادية لوباء كوفيد-19. وتهدد أزمة الطاقة وتكلفة المعيشة الحالية الآن بعكس مسار التقدم نحو طاقة ميسورة التكلفة وموثوقة ومستدامة، مما يزيد من تقويض المحددات الاجتماعية والاقتصادية للصحة.

في ذات الوقت، تسجل شركات النفط والغاز أرباحًا قياسية، في حين تستمر استراتيجيات الإنتاج الخاصة بها في تقويض حياة الناس ورفاهيتهم. كشف تحليل لاستراتيجيات الإنتاج لـ 15 من كبريات شركات النفط والغاز في العالم، اعتبارًا من فبراير 2022، أنها ستتجاوز نصيبها من الانبعاثات بما يتفق مع 1·5 درجة مئوية من الاحترار العالمي (المؤشر 4.2.6) بنسبة 37٪ في عام 2030 و 103٪ في عام 2040، مواصلة تقويض الجهود المبذولة لتوفير مستقبل منخفض الكربون وصحي ومناسب للعيش. ومما زاد من تفاقم هذا الوضع، استمرار الحكومات في تحفيز إنتاج الوقود الأحفوري واستهلاكه: 69 (80٪) من 86 دولة تمت مراجعة بياناتها، و لديها أسعار كربون سلبية صافية (أي قدمت دعمًا صافيا للوقود الأحفوري) بإجمالي صاف قدره 400 مليار دولار أمريكي في عام 2019،

تخصص مبالغ غالبًا ما يمكن مقارنتها بمبالغ ميزانياتها الإجمالية المخصصة للصحة، بل تجاوزها (المؤشر 4.2.4). في الوقت نفسه، أخفقت البلدان الأكثر ثراءً في الوفاء بالتزامها بحشد المبلغ المنخفض كثيراً المتمثل في 100 مليار دولار سنويًا بحلول عام 2020 على النحو المتفق عليه في اتفاق كوبنهاغن لعام 2009 لدعم العمل المناخي في "البلدان النامية"، لذا تتعرض جهود المناخ لنقص شديدة في التمويل (المؤشر 2.1.1). ومن المفارقات أن آثار تغير المناخ على الاقتصادات العالمية، إلى جانب الركود الناجم عن كوفيد-19 والتي تفاقمت بسبب عدم الاستقرار الجيوسياسي، يمكن أن تقلل من استعداد البلدان لتخصيص الأموال اللازمة لتمكين انتقال مناخي عادل.

استجابة تتمحور حول الصحة من أجل مستقبل مزدهر

يمر العالم بلا شك بمنعطف حرج. ومع مواجهة البلدان لأزمات متزامنة، فإن تنفيذ سياسات الحد من الانبعاثات على المدى الطويل قد انحرفت أو هُزمت بسبب التحديات التي يُنظر إليها خطأً على أنها أكثر إلحاحًا. إن معالجة كل أزمة من الأزمات المتزامنة بمعزل عن غيرها يخاطر بتخفيف إحداها، ومفاقمة أخرى. ينشأ ذلك كما في حالة الاستجابة لكوفيد-19، التي لم تحقق حتى الآن الانتعاش الأخضر الذي اقترحه المجتمع الصحي، بل على العكس من ذلك، فاقمت المخاطر الصحية المرتبطة بتغير المناخ. ومن المرجح أن يقلل أقل من ثلث 3٫11 تريليون دولار المخصصة للانتعاش الاقتصادي لكوفيد-19 من انبعاثات غازات الاحتباس الحراري أو تلوث الهواء، مع احتمال أن يؤدي التأثير الصافي إلى زيادة الانبعاثات. لقد أثرت جائحة كوفيد-19 على العمل المناخي على مستوى المدينة، وأفادت 239 (30٪) من 798 مدينة أن كوفيد-19 قلل التمويل المتاح للعمل المناخي (المؤشر 2.1.3). بينما تبحث البلدان عن بدائل للنفط والغاز الروسي، يواصل الكثيرون تفضيل حرق الوقود الأحفوري، حتى أن البعض عاد إلى الفحم. تهدد التحولات في إمدادات الطاقة العالمية بزيادة إنتاج الوقود الأحفوري. حتى لو تم ذلك كتحول مؤقت، يمكن لهذه الاستجابات عكس التقدم في تحسين جودة الهواء، ودفع العالم بشكل لا رجعة فيه عن المسار الصحيح للوفاء بالالتزامات المنصوص عليها في اتفاقية باريس، وضمان مستقبل يتسارع فيه التغيرالمناخي الذي يهدد بقاء الإنسان.

على العكس من ذلك، في هذه اللحظة المحورية، ستظل الاستجابة التي تتمحور حول الصحة للأزمات الحالية توفر الفرصة لمستقبل منخفض الكربون وقابل للتكيف، والذي لا يتجنب الأضرار الصحية الناجمة عن تغير المناخ المتسارع فحسب، بل يؤدي أيضًا إلى تحسين الصحة والرفاهية من خلال الفوائد المشتركة المرتبطة بالعمل المناخي. مثل هذه الاستجابة ستشهد تحول البلدان على الفور بعيدًا عن الوقود الأحفوري، وتقليل اعتمادها على أسواق النفط والغاز الدولية الهشة، وتسريع الانتقال العادل إلى مصادر الطاقة النظيفة. من شأن الاستجابة التي تتمحور حول الصحة أن تقلل من احتمالية حدوث أكثر آثار تغير المناخ كارثية، مع تحسين أمن الطاقة، وخلق فرصة للتعافي الاقتصادي، وتقديم فوائد صحية فورية. وهذه التحسينات في جودة الهواء من شأنها أن تساعد في منع حدوث 1٫2 مليون حالة وفاة ناجمة عن التعرض لمواد PM2.5 المشتقة من الوقود الأحفوري في عام 2020 وحده (المؤشر 3.3)، كما أن التحول في مجال الطاقة المرتكز على الصحة من شأنه أن يعزز السفر منخفض الكربون ويزيد من المساحات الخضراء، ويعزز النشاط البدني، ويحسن الصحة البدنية والعقلية. وفي قطاع الأغذية، لن يساعد الانتقال السريع إلى أنظمة غذائية متوازنة وأكثر نباتية في تقليل 55٪ من انبعاثات القطاع الزراعي الناتجة عن اللحوم الحمراء وإنتاج الألبان (المؤشر 3.5.1)، ولكن أيضًا سيمنع ما يصل إلى 11.5 مليون حالة وفاة مرتبطة بالنظام الغذائي سنويًا (المؤشر 3.5.2)، ويقلل بشكل كبير من مخاطر الأمراض الحيوانية المنشأ. وستقلل هذه التحولات التي تركز على الصحة من عبء الأمراض المعدية وغير المعدية، مما يقلل الضغط على مقدمي الرعاية الصحية المثقلين. الأهم من ذلك، أن تسريع التكيف مع تغير المناخ سيؤدي إلى أنظمة صحية أكثر قوة، ويقلل من الآثار السلبية لتفشي الأمراض المعدية في المستقبل والصراعات الجيوسياسية، واستعادة خط الدفاع الأول لسكان العالم.

بصيص أمل ناشئ

على الرغم من عقود من العمل غير الكافي، إلا أن ظهور علامات التغيير، وإن كانت قليلة، توفر بعض الأمل في أن استجابة تتمحور حول الصحة قد تبدأ في الظهور. لقد زاد تفاعل الأفراد مع الأبعاد الصحية لتغير المناخ، وهو أمر ضروري لدفع وتمكين الاستجابة السريعة، من 2020 إلى 2021 (المؤشر 5.2)، ووصلت تغطية الصحة وتغير المناخ في وسائل الإعلام إلى مستوى قياسي جديد في عام 2021، مع زيادة بنسبة 27 بالمائة عن عام 2020 (المؤشر 5.1). لقد ظهر هذا التفاعل أيضًا من قبل قادة الدول، حيث سجل 60٪ من 194 دولة تركز اهتمامها على الروابط بين تغير المناخ والصحة في المناقشة العامة للأمم المتحدة لعام 2021، و 86٪ من المساهمات المحددة وطنيًا المحدثة أو الجديدة تشير إلى الصحة (المؤشر 5.4). وعلى مستوى المدينة، تحدد السلطات المحلية بشكل تدريجي مخاطر تغير المناخ على صحة سكانها (المؤشر 2.1.3)، وهي خطوة أولى لتقديم استجابة مخصصة تعزز النظم الصحية المحلية. على الرغم من أن قطاع الصحة مسؤول عن 5٫2٪ من جميع الانبعاثات العالمية (المؤشر 3.6)، فقد أظهر قيادة رائعة في مجال المناخ، والتزمت 60 دولة بالتحول إلى النظم الصحة المتكيفة مع المناخ و / أو منخفضة الكربون أو خالية من الكربون، كجزء من برنامج الصحة COP26، اعتبارًا من يوليو 2022.

كما تظهر بوادر التغيير في قطاع الطاقة. فعلى الرغم من أن إجمالي توليد الطاقة النظيفة لا يزال غير كافٍ بشكل كبير، فقد تم الوصول إلى مستويات عالية قياسية في عام 2020 (المؤشر 3.1). شكلت مصادر الكربون الصفري 80٪ من الاستثمار في توليد الكهرباء في عام 2021 (المؤشر 4.2.1)، ووصلت الطاقات المتجددة إلى تكافؤ التكلفة مع طاقات الوقود الأحفوري. في الوقت الذي

تحاول فيه بعض البلدان ذات الانبعاثات الأعلى خفض اعتمادها على النفط والغاز استجابة للحرب في أوكرانيا وارتفاع أسعار الطاقة، يركز العديد منها على زيادة توليد الطاقة المتجددة، مما يرفع الآمال في استجابة تتمحور حول الصحة. ومع ذلك، ينبغي ترجمة الوعي والالتزامات المتزايدة بشكل عاجل إلى عمل أملاً في التحول إلى حقيقة واقعة.

دعوة إلى التحرك

بعد 30 عامًا من مفاوضات اتفاقية الأمم المتحدة الإطارية بشأن تغير المناخ، تُظهر مؤشرات العد التنازلي لمجلة "لانسيت" أن البلدان والشركات تواصل اتخاذ خيارات تهدد على نحو متزايد صحة الناس وبقائهم في كل جزء من العالم. بينما تبتكر البلدان طرقًا للتعافي من الأزمات القائمة، فإن الأدلة لا لبس فيها في هذا المنعطف الحرج: إن الاستجابة الفورية والمتسقة والمتمحورة حول الصحة ضرورية لتأمين مستقبل قابل للعيش وتوفر فرصة جديدة لإيجاد عالم يمكن للناس فيه، في الحاضر والمستقبل، ليس البقاء على قيد الحياة فحسب، بل الازدهار أيضاً.
