# Supplementary material for: The 2022 report of the Lancet Countdown on health and climate change: health at the mercy of fossil fuels
Source: Lancet. Author manuscript; Available in PMC 2024 Nov 15. (PMC7616806; doi:10.1016/S0140-6736(22)01540-9)
Supplement: Chinese translation of the Executive Summary [file EMS200004-supplement-Chinese_translation_of_the_Executive_Summary.pdf]

THE LANCET

Supplementary appendix 2

This translation in Chinese was submitted by the authors and we reproduce it as supplied. It has not been peer reviewed. *The Lancet*'s editorial processes have only been applied to the original in English, which should serve as reference for this manuscript.

此简体中文译文由作者提交，我方按照提供的版本刊登。此译文并未经过同行审阅。医学期刊《柳叶刀》的编辑流程仅适用于英文原稿，英文原稿应作为此手稿的参考。

Supplement to: Romanello M, Di Napoli C,Drummond P, et al. The 2022 report of the *Lancet* Countdown on health and climate change: health at the mercy of fossil fuels. *Lancet* 2022; published online Oct 25. https://doi.org/10.1016/S0140-6736(22)01540-9.

《柳叶刀倒计时》2022年健康和气候变化报告：化石燃料对健康的影响

执行摘要

《柳叶刀倒计时》发表 2022 年报告时，世界正面临着深刻的、关于当前系统性的冲击。各国和卫生系统继续应对 COVID-19 疫情对健康、社会和经济的影响，而俄罗斯对乌克兰的入侵和对化石燃料的持续过度依赖已将世界推入全球能源和生活成本增加的危机。随着这些危机临近，令气候变化加剧。其不断恶化的影响正日益影响到人类健康和福祉的基础，加剧损耗世界人口增长对健康造成的威胁。

在 2021 年和 2022 年期间，极端天气事件在各大洲都造成了破坏，给已经在努力应对 COVID-19 疫情影响的卫生服务部门增加了更多压力。澳大利亚、巴西、中国、西欧、马来西亚、巴基斯坦、南非和南苏丹的洪水造成数千人死亡，数十万人流离失所，并造成数十亿美元的经济损失。野火在加拿大、美国、希腊、阿尔及利亚、意大利、西班牙和土耳其造成了破坏，许多国家，包括澳大利亚、加拿大、印度、意大利、阿曼、土耳其、巴基斯坦和英国都录得创纪录的温度。随着探测和归因研究科学的进步，气候变化对许多事件的影响现在已经被量化了。

由于气温迅速升高，脆弱人群（65 岁以上的成年人和一岁以下的儿童）在 2021 年面临的热浪天数比 1986-2005 年多 30-70 亿天（指标 1.1.2），2000-04 年和 2017-21 年之间，与热有关的死亡人数增加了 68%（指标 1.1.5），这一死亡人数因 COVID-19 疫情的汇合而明显加剧。

同时，不断变化的气候正在影响传染病的传播，使人口处于新出现的疾病和共同流行病的更高风险中。沿海水域有越来越适合弧菌病原体传播；从 1951-60 年到 2012-21 年，美洲高原地区适合疟疾传播的月份增加了 31-3%，非洲高原地区增加了 13-8%，同期登革热传播的可能性增加了 12%（指标 1.3.1）。登革热爆发与 COVID-19 疫情同时存在，导致南美、亚洲和非洲许多地区的卫生系统压力加重，出现误诊，两种疾病的管理都很困难。

与气候变化影响相关的经济损失也在增加，家庭和经济体同时受到 COVID-19 疫情以及国际生活费用和能源危机的影响承受了压力，进一步破坏了维持良好健康的决定因素，也就是社会经济。2021 年，热暴露导致全球损失了 4700 亿个潜在的劳动时间（指标 1.1.4），潜在的收入损失相当于全球经济产出的 0-72%，在人类发展指数（HDI）低的国家增加到 GDP 的 5-6%，那里的工人最容易受到金融波动的影响（指标 4.1.3）。同时，极端天气事件在 2021 年造成了价值 2530 亿美元的损失，特别是给人类发展指数低的国家的人民带来了负担，这些国家的损失几乎没有被保险（指标 4.1.1）。

通过多种相互关联的途径，粮食安全的每个方面都受到气候变化的影响，加剧了其他并存危机的影响。更高的温度直接威胁着作物产量，2020 年全球玉米的生长季节平均缩短 9 天，冬小麦和春小麦的生长季节比 1981-2010 年缩短 6 天（指标 1.4）。对作物产量的威胁使极端天气对供应链、社会经济压力和传染病传播风险的影响不断增加，破坏了粮食的供应、获取、稳定和利用。新的分析表明，在分析的 103 个国家中，极端高温与 2020 年报告中度至严重粮食不安全的人数比 1981-2010 年每年多出 9800 万人有关（指标 1.4）。越来越极端的天气使全球粮食系统的稳定性恶化，与其他同时发生的危机协同作用，逆转消除饥饿的进展。事实上，在 COVID-19 疫情期间，营养不良的流行率上升，2020 年 COVID-19 疫情期间，面临饥饿的人比 2019 年多出多达 1.61 亿。这种情况现在因俄罗斯入侵乌克兰以及能源和生活费用危机而恶化，对国际农业生产和供应链的影响有可能导致 2022 年面临营养不良的人数增加 1300 万。

衰弱的第一道防线

随着气候变化对健康的影响不断恶化，其他并存的危机也在不断恶化，世界各地的人口越来越依赖卫生系统作为他们的第一道防线。就在医疗保健需求上升的同时，全世界的卫生系统也因 COVID-19 疫情以及能源和生活成本危机的影响而变得脆弱。因此，需要采取紧急行动来加强卫生系统的复原力，并防止因不断变化的气候而导致的更多生命损失和痛苦。然而，报告评估了 95 个国家，其中只有 48 个（51%）适应气候变化的需求（指标 2.1.1），即使在 COVID-19 的巨大影响之后，也只有 60 个（63%）国家在 2021 年卫生应急管理的执行状况为高或非常高（指标 2.2.4）。

主动适应的稀缺性表现在对极端高温的反应上。尽管城市绿地具有局部降温和整体健康效益，但 2021 年全球 1038 个城市中心中只有 277 个（27%）至少是中等程度的绿色（指标 2.2.3），从 2000 年到 2020 年，使用空调的家庭数量增加了 66%，这种不适应的反应加剧了能源危机，进一步增加了城市热量、空气污染和温室气体排放。

随着各种危机进一步威胁到世界的生命支持系统，快速、果断和一致的跨部门行动对于保护人类健康免受快速变化的气候的危害至关重要。

健康受化石燃料的摆布

2022 年是《联合国气候变化框架公约》签署 30 周年，各国在该公约中同意防止危险的人为气候变化及其对人类健康和福祉的有害影响。可是，此后几乎没有什么有意义的行动。自《联合国气候变化框架公约》制定以来，全球能源系统的碳强度下降了不到 1%，全球发电仍以化石燃料为主，可再生能源仅占全球总量的 8-2%（指标 3.1）。同时，能源需求总量增加了 59%，使与能源有关的排放量在 2021 年达到历史最高点。目前的政策使全球气温在本世纪末达到灾难性的 2-7℃的增长。即使各国在截至 2021 年 11 月的国家决定性贡献（NDCs）中做出承诺，

到 2030 年，全球排放量也可能比 2010 年的水平高出 13-7%，远远没有达到《巴黎协定》的目标，即是将温度控制在适应范围内所需的比目前水平减少 43%。

气候变化加速了世界对化石燃料的依赖，影响和破坏全球健康，而且还通过动荡和不可预测的化石燃料市场、脆弱的供应链和地缘政治冲突直接影响到人类的健康和福祉。因此，数以百万计的人无法获得保持家庭健康的温度、保存食物和药物以及实现第七项可持续发展目标（确保人人都能获得负担得起、可靠、可持续和现代的能源）所需的能源。如果没有足够的支持，低人类发展指数国家获得纯净能源的速度尤其缓慢，在 2020 年，他们只有 1-4%的电力来自现代可再生能源（主要是风能和太阳能）（指标 3.1）。据估计，中低收入国家有 59%的医疗设施仍然无法获得提供基本护理所需的可靠电力。同时，在全球范围内，生物质能占家庭部门所消耗能源的 31%，其中大部分来自传统来源，这一比例在低人类发展指数国家增加到 96%（指标 3.2）。与此相关的疾病负担非常巨大，在评估的 62 个国家中，2020 年他们家中的空气高于世卫组织的小颗粒空气污染（PM2-5）安全浓度指南，平均浓度为安全浓度的 30 倍（指标 3.2）。由于 COVID-19 疫情所带来的社会经济压力，纵使经过 6 年的改善，在 2020 年无法获得电力的人数仍有所增加。目前的能源和生活费用危机现在有可能逆向在可负担、可靠和可持续能源方面的漫延，进一步破坏维持健康的社会经济决定因素。

同时，石油和天然气公司的利润创下新高，而他们的生产策略却继续破坏人们的生活和福祉。截至 2022 年 2 月，对世界上最大的 15 家石油和天然气公司的生产战略的分析表明，它们在 2030 年和 2040 年的排放量分别比全球升温 1-5℃（指标 4.2.6）的比例高出 37%和 103%，继续破坏为实现低碳、健康、宜居的未来而做出的努力。使这种情况进一步恶化的是，政府继续激励化石燃料的生产和消费。在接受评估的 86 个国家中，有 69 个国家（80%）在 2019 年实行净负的碳价格（即对化石燃料提供净补贴），净总额为 4000 亿美元，分配的金额往往与他们的卫生预算总额相当，甚至超过他们的卫生预算总额（指标 4.2.4）。同时，较富裕的国家未能履行其承诺，即到 2020 年每年调动 1000 亿美元的低得多的资金，以支持 "发展中国家 "的气候行动，而且针对气候方面而付出的努力正被严重缺乏的资金所削弱（指标 2.1.1）。气候变化对全球经济的影响，加上 COVID-19 引发的经济衰退和地缘政治不稳定的恶化，可能会自相矛盾地进一步降低各国为实现公平地因气候转型而分配所需资金的意愿。

以健康为中心的应对措施，创造繁荣的未来

世界正处于一个关键时刻。由于各国同时面临危机，长期减排政策的实施有可能被错误地认为是更紧迫的挑战所转移或击败。孤立地解决每一个同时发生的危机有可能缓解一个危机，而使另一个危机恶化。这种情况正出现在对应 COVID-19 的对策中，到目前为止，它并没有带来卫生界所建议的绿色复苏，相反，正在加剧与气候变化有关的健康风险。在分配给 COVID-19 经济复苏的 3-11 万亿美元中，只有不到三分之一可能可以减少温室气体排放或空气污染，其

净效应可能会增加排放。COVID-19 疫情影响了城市层面的气候行动，798 个城市中有 239 个（30%）报告说，COVID-19 减少了可用于气候行动的资金（指标 2.1.3）。在各国寻找俄罗斯石油和天然气的替代品时，许多国家继续赞成燃烧化石燃料，一些国家甚至回头使用煤炭。全球能源供应的转变有可能增加化石燃料的生产。即使作为暂时的过渡实施，这些应对措施也会逆转空气质量改善方面的进展，不可逆转地推动世界偏离实现《巴黎协定》所规定的承诺的轨道，并保证未来气候变化加速，威胁人类生存。

相反，在这个关键时刻，以健康为中心应对当前的危机，仍将为建立一个低碳、可变的未来提供机会，这不仅可以避免加速气候变化对健康的伤害，还可以通过气候行动的相关共同利益来改善健康和福祉。这种应对措施将看到各国迅速摆脱化石燃料，减少对脆弱的国际石油和天然气市场的依赖，并加速向纯净能源的公正过渡。一个以健康为中心的应对措施将减少最灾难性的气候变化影响的可能性，同时改善能源安全，创造经济复苏的机会，并提供直接的健康效益。空气质量的改善将有助于防止仅在 2020 年就有 1-2 百万人因接触化石燃料产生的环境 PM2-5 而死亡（指标 3.3），以健康为中心的能源转型将加强低碳旅行，增加城市绿地，促进身体活动，改善身体和心理健康。在食品领域，加速向平衡和更多的植物性饮食过渡，不仅有助于减少 55%的农业部门排放来自红肉和牛奶生产（指标 3.5.1），而且每年可防止多达 1100-500 万与饮食有关的死亡人数（指标 3.5.2），并大幅降低人畜共患疾病的风险。这些以健康为重点的转变将减少传染病和非传染病的负担，减轻不堪重负的医疗保健提供者的压力。重要的是，加速适应气候变化将导致更强大的卫生系统，最大限度地减少未来传染病爆发和地缘政治冲突的负面影响，并恢复全球人口的第一道防线。

新出现的希望之光

尽管几十年来行动不足，但新出现的变化迹象（尽管很少）提供了一些希望，即是以健康为中心的对策可能开始出现。从 2020 年到 2021 年，个人对气候变化的健康层面的参与有所增加，这对推动和促成加速反应至关重要（指标 5.2），媒体对健康和气候变化的报道在 2021 年达到了新的记录，比 2020 年增加了 27%（指标 5.1）。这种参与也反映在国家领导人身上，194 个国家中有 60%的国家在 2021 年的联合国一般性辩论中关注气候变化与健康之间的联系，86%的国家更新或在制定新的国家发展计划提到了健康（指标 5.4）。在城市层面，地方当局正在逐步确定气候变化对其人口健康的风险（指标 2.1.3），这是提供有针对性的应对措施，加强地方卫生系统的第一步。虽然卫生部门占全球总排放量的 5-2%（指标 3.6），但它已展示出令人印象深刻的气候领导力，60 个国家已承诺，在 2022 年 7 月前，作为 COP26 卫生计划的一部分，过渡到具有气候适应性和/或低碳或净零碳卫生系统。

能源部门也出现了变革的迹象。尽管纯净能源发电总量仍然严重不足，但在 2020 年达到了创纪录的高水平（指标 3.1）。在 2021 年，零碳能源占发电投资的 80%（指标 4.2.1），可见可

再生能源已达到与化石燃料能源的成本持平。由于一些排放最高的国家试图减少对石油和天然气的依赖，以及应对乌克兰战争和飙升的能源价格，许多国家正专注于增加可再生能源发电，给以健康为中心的对策带来了希望。然而，提高认识和承诺，应该紧急转化为行动，以便将希望变为现实。

行动呼吁

经过 30 年的《联合国气候变化框架公约》谈判，《柳叶刀倒计时》指标显示，各国和各公司继续做出选择，日益威胁着世界上每个地方人们的健康和生存。在各国想方设法从并存的危机中恢复过来的时候，在这个关键时刻，证据是明确的：立即采取一致的、以健康为中心的应对措施，对于确保一个可居住的未来非常重要，并提供了一个新的机会，使现在和未来的人口不仅可以生存，而且可以繁盛起来。
