# Supplementary material for: The 2022 report of the Lancet Countdown on health and climate change: health at the mercy of fossil fuels
Source: Lancet. Author manuscript; Available in PMC 2024 Nov 15. (PMC7616806; doi:10.1016/S0140-6736(22)01540-9)
Supplement: French translation of the executive summary [file EMS200004-supplement-French_translation_of_the_executive_summary.pdf]

THE LANCET

Supplementary appendix 1

This translation in French was submitted by the authors and we reproduce it as supplied. It has not been peer reviewed. *The Lancet*'s editorial processes have only been applied to the original in English, which should serve as reference for this manuscript.

Cette traduction en français a été proposée par les auteurs et nous l'avons reproduite telle quelle. Elle n'a pas été examinée par des pairs. Les processus éditoriaux du *Lancet* n'ont été appliqués qu'à l'original en anglais et c'est cette version qui doit servir de référence pour ce manuscrit.

Supplement to: Romanello M, Di Napoli C,Drummond P, et al. The 2022 report of the *Lancet* Countdown on health and climate change: health at the mercy of fossil fuels. *Lancet* 2022; published online Oct 25. https://doi.org/10.1016/S0140-6736(22)01540-9.

Le rapport 2022 du Compte à rebours du *Lancet* sur la santé et les changements climatiques : la santé à la merci des combustibles fossiles

Résumé

Le rapport 2022 du Compte à rebours du Lancet est publié alors que le monde est confronté à des chocs systémiques profonds et actuels. Les pays et les systèmes de santé continuent de faire face aux répercussions sanitaires, sociales et économiques de la pandémie de la COVID-19, tandis que l'invasion de l'Ukraine par la Russie et une dépendance excessive persistante aux combustibles fossiles ont plongé le monde dans une crise mondiale de l'énergie et du coût de la vie. Au fur et à mesure que ces crises se développent, les changements climatiques s'intensifient sans relâche. Ses effets croissants affectent de plus en plus les fondements de la santé et du bien-être des personnes, exacerbant la vulnérabilité des populations mondiales aux menaces concomitantes pour la santé.

En 2021 et 2022, des phénomènes météorologiques extrêmes ont dévasté tous les continents, ce qui a accru la pression sur les services de santé déjà aux prises avec les répercussions de la pandémie de la COVID-19. Les inondations en Australie, au Brésil, en Chine, en Europe occidentale, en Malaisie, au Pakistan, en Afrique du Sud et au Soudan du Sud ont fait des milliers de morts, déplacé des centaines de milliers de personnes et causé des milliards de dollars de pertes économiques. Les incendies de forêt ont causé des ravages au Canada, aux États-Unis, en Grèce, en Algérie, en Italie, en Espagne et en Turquie, et des températures record ont été enregistrées dans de nombreux pays, dont l'Australie, le Canada, l'Inde, l'Italie, Oman, la Turquie, le Pakistan et le Royaume-Uni. Avec les progrès de la science des études de détection et d'attribution, l'influence des changements climatiques sur de nombreux événements a maintenant été quantifiée.

En raison de l'augmentation rapide des températures, les populations vulnérables (adultes de plus de 65 ans et enfants de moins d'un an) ont été exposées à 3,7 milliard de jours de canicule de plus en 2021 que chaque année entre 1986-2005 (indicateur 1.1.2), et les décès liés à la chaleur ont augmenté de 68% entre 2000-2004 et 2017-2021 (indicateur 1.1.5), un nombre de décès considérablement exacerbé par la confluence de la pandémie de la COVID-19.

Simultanément, les changements climatiques affectent la propagation des maladies infectieuses, exposant les populations à un risque plus élevé de maladies émergentes et d'épidémies conjointes. Les eaux côtières sont de plus en plus propices à la transmission des agents pathogènes du genre Vibrio ; le nombre de mois propices à la transmission du paludisme a augmenté de 31,3% dans les régions montagneuses des Amériques et de 13,8% dans les régions montagneuses d'Afrique entre 1951–60 et 2012–21, et la probabilité de transmission de la dengue a augmenté de 12% au cours de la même période (indicateur 1.3.1). La coexistence des épidémies de dengue avec la pandémie de la COVID-19 a entraîné une pression accrue sur les systèmes de santé, des diagnostics erronés et des difficultés dans la gestion de ces deux maladies dans de nombreuses régions d'Amérique du Sud, d'Asie et d'Afrique.

Les pertes économiques associées aux répercussions des changements climatiques augmentent également la pression sur les familles et les économies déjà confrontées aux effets synergiques de la pandémie de la COVID-19 et des crises internationales du coût de la vie et de l'énergie, sapant davantage les déterminants socioéconomiques dont dépend une bonne santé. L'exposition à la chaleur a entraîné une perte potentielle de 470 milliards d'heures de travail à l'échelle mondiale en

2021 (indicateur 1.1.4), avec des pertes de revenus potentielles équivalant à 0,72% de la production économique mondiale, passant à 5,6% du PIB dans les pays à faible indice de développement humain (IDH), où les travailleurs sont plus vulnérables aux effets des fluctuations financières (indicateur 4.1.3). En même temps, les phénomènes météorologiques extrêmes ont causé des dommages d'une valeur de 253 milliards de dollars US en 2021, frappant particulièrement les populations des pays à faible IDH dans lesquels pratiquement aucunes pertes n'étaient assurées (indicateur 4.1.1).

À cause des voies multiples et interconnectées, chaque aspect de la sécurité alimentaire est affecté par les changements climatiques, aggravant les répercussions d'autres crises concomitantes. Les températures plus élevées menacent directement les rendements des cultures avec les saisons de croissance du maïs en moyenne 9 jours plus courtes en 2020 et les saisons de croissance du blé d'hiver et du blé de printemps 6 jours plus courtes qu'en 1981–2010 à l'échelle mondiale (indicateur 1.4). La menace qui pèse sur les rendements des cultures s'ajoute à l'impact croissant des conditions météorologiques extrêmes sur les chaînes d'approvisionnement, les pressions socio-économiques et le risque de transmission de maladies infectieuses, ce qui compromet la disponibilité, l'accès, la stabilité et l'utilisation des aliments. Une nouvelle analyse signale que la chaleur extrême était associée à 98 millions de personnes de plus en 2020 qui ont déclaré une insécurité alimentaire modérée à grave par rapport à 1981-2010, dans 103 pays analysés (indicateur 1.4). Les conditions météorologiques de plus en plus extrêmes aggravent la stabilité des systèmes alimentaires mondiaux, agissant en synergie avec d'autres crises simultanées pour inverser les progrès vers l'éradication de la faim. En effet, la prévalence de la sous-alimentation a augmenté pendant la pandémie de la COVID-19, et jusqu'à 161 millions de personnes supplémentaires ont souffert de la faim pendant la pandémie de la COVID-19 en 2020 par rapport à 2019. Cette situation est maintenant aggravée par l'invasion de l'Ukraine par la Russie et les crises de l'énergie et du coût de la vie, avec des impacts sur la production agricole internationale et les chaînes d'approvisionnement menacent d'entraîner 13 millions de personnes supplémentaires à être confrontées à la sous-alimentation en 2022.

Une première ligne de défense affaiblie

Avec l'aggravation des répercussions sur la santé dues aux changements climatiques qui aggravent d'autres crises concomitantes, les populations du monde entier comptent de plus en plus sur les systèmes de santé comme première ligne de défense. Cependant, alors que les besoins en soins de santé augmentent, les systèmes de santé à l'échelle mondiale sont affaiblis par les effets de la pandémie de la COVID-19 et les crises de l'énergie et le coût de la vie. Une action urgente est donc nécessaire pour renforcer la résilience des systèmes de santé et prévenir une escalade rapide des pertes en vies humaines et des souffrances dans un climat changeant. Toutefois, seuls 48 (51%) des 95 pays ont déclaré avoir évalué leurs besoins en matière d'adaptation aux changements climatiques (indicateur 2.1.1) et, même après les impacts profonds de la COVID-19, seuls 60 (63%) pays ont déclaré un état de mise en œuvre élevé à très élevé de la gestion des urgences sanitaires en 2021 (indicateur 2.2.4).

La rareté de l'adaptation proactive est mise en évidence dans la réponse à la chaleur extrême. Malgré le refroidissement local et les avantages globaux des espaces verts urbains pour la santé, seuls 277 (27%) des 1038 centres urbains mondiaux étaient au moins modérément verts en 2021 (indicateur 2.2.3), et le nombre de ménages ayant la climatisation a augmenté de 66% entre 2000 et 2020, une réponse inadaptée qui aggrave la crise énergétique et augmente encore la chaleur urbaine, la pollution atmosphérique et les émissions de gaz à effet de serre.

Étant donné que les crises convergentes menacent davantage les systèmes vitaux du monde, une action intersectorielle rapide, décisive et cohérente est essentielle pour protéger la santé des personnes contre les dangers des changements climatiques rapides.

La santé à la merci des énergies fossiles

L'année 2022 marque le 30e anniversaire de la signature de la Convention-cadre des Nations Unies sur les changements climatiques, dans laquelle les pays ont convenu de prévenir les changements climatiques anthropiques dangereux et leurs effets néfastes sur la santé et le bien-être des populations. Cependant, peu de mesures concrètes ont été prises depuis. L'intensité en carbone du système énergétique mondial a diminué de moins de 1% depuis la création de la CCNUCC, et la production mondiale d'électricité est toujours dominée par les combustibles fossiles, les énergies renouvelables ne représentant que 8,2% du total mondial (indicateur 3.1). Simultanément, la demande totale d'énergie a augmenté de 59%, augmentant les émissions liées à l'énergie à un sommet historique en 2021. Les politiques actuelles placent le monde sur la voie d'une augmentation catastrophique de 2,7°C d'ici la fin du siècle. Même avec les engagements que les pays ont pris dans les contributions déterminées au niveau national (CDN) mis à jour jusqu'en novembre 2021, les émissions mondiales pourraient être supérieures de 13,7% aux niveaux de 2010 d'ici 2030, loin de la baisse de 43% par rapport aux niveaux actuels requis pour atteindre les objectifs de l'Accord de Paris et maintenir les températures dans les limites de l'adaptation.

La dépendance aux combustibles fossiles ne nuit pas seulement à la santé mondiale en raison des répercussions accrues des changements climatiques, mais elle a une incidence directe sur la santé et le bien-être des populations, en raison de marchés volatils et imprévisibles des combustibles fossiles, de chaînes d'approvisionnement fragiles et de conflits géopolitiques. En conséquence, des millions de personnes n'ont pas accès à l'énergie nécessaire pour maintenir leur maison à des températures saines, préserver les aliments et les médicaments et atteindre le 7e objectif de développement durable (assurer l'accès à une énergie abordable, fiable, durable et moderne pour tous). Sans soutien suffisant, l'accès à l'énergie propre a été particulièrement lent dans les pays à faible IDH, et seulement 1,4% de leur électricité provenait d'énergies renouvelables modernes (principalement éolienne et solaire) en 2020 (indicateur 3.1). On estime que 59% des établissements de soins de santé dans les pays à revenu faible ou intermédiaire n'ont toujours pas accès à l'électricité fiable nécessaire pour fournir des soins de base. Parallèlement, la biomasse représente jusqu'à 31% de l'énergie consommée dans le secteur domestique à l'échelle mondiale, principalement à partir de sources traditionnelles, une proportion qui augmente à 96% dans les pays à faible IDH (indicateur 3.2). La charge de morbidité associée est considérable, l'air dans les habitations dépassant de 30 fois en moyenne les recommandations de l'OMS pour des concentrations sûres de pollution atmosphérique par les petites particules (PM2,5) en 2020 dans les 62 pays évalués (indicateur 3.2). Après 6 ans d'amélioration, le nombre de personnes sans accès à l'électricité a augmenté en 2020 en raison des pressions socio-économiques de la pandémie de la COVID-19. La crise actuelle de l'énergie et du coût de la vie menace maintenant d'inverser les progrès vers une énergie abordable, fiable et durable, affaiblissant davantage les déterminants socioéconomiques de la santé.

Simultanément, les sociétés pétrolières et gazières enregistrent des bénéfices records, tandis que leurs stratégies de production continuent de nuire à la vie et au bien-être des personnes. Une analyse des stratégies de production de 15 des plus grandes sociétés pétrolières et gazières du monde, en février 2022, a révélé qu'elles dépassaient leur part d'émissions correspondant à 1,5°C de réchauffement planétaire (indicateur 4.2.6) de 37% en 2030 et de 103% en 2040, continuant de saper les efforts visant à offrir un avenir sain, viable et à faibles émissions de carbone. Aggravant encore cette situation, les gouvernements continuent d'encourager la production et la

consommation de combustibles fossiles : 69 (80%) des 86 pays examinés avaient des prix du carbone négatifs nets (c'est-à-dire une subvention nette aux combustibles fossiles) pour un total net de 400 milliards de dollars US en 2019, allouant des montants souvent comparables ou même supérieurs à leurs budgets totaux de santé (indicateur 4.2.4). En même temps, les pays les plus riches n'ont pas respecté leur engagement de mobiliser la somme considérablement inférieure de 100 milliards de dollars par an d'ici 2020, comme convenu lors de l'Accord de Copenhague de 2009 pour soutenir l'action climatique dans les "pays en développement", et les efforts climatiques sont affaiblis par une grave pénurie de financement (indicateur 2.1.1). Les répercussions des changements climatiques sur les économies mondiales, ainsi que la récession déclenchée par la COVID-19 et aggravée par l'instabilité géopolitique, pourraient paradoxalement réduire davantage la volonté des pays d'allouer les fonds nécessaires pour permettre une transition climatique juste.

Une réponse axée sur la santé pour un avenir prospère

Le monde se trouve à un tournant critique. Les pays étant confrontés à des crises simultanées, la mise en œuvre de politiques de réduction des émissions à long terme risque d'être détournée ou vaincue par des défis perçus à tort comme plus immédiats. Traiter chacune des crises concomitantes de manière isolée risque d'en atténuer une, tout en aggravant une autre. Une telle situation émerge de la réponse à la COVID-19, qui n'a jusqu'à présent pas apporté la reprise verte proposée par le milieu de la santé et, au contraire, aggrave les risques pour la santé liés aux changements climatiques. Moins d'un tiers des 3,11 billions de dollars alloués à la reprise économique liée à la COVID-19 devraient réduire les émissions de gaz à effet de serre ou la pollution atmosphérique, avec l'effet net susceptible d'augmenter les émissions. La pandémie de la COVID-19 a affecté l'action climatique au niveau des villes, et 239 (30%) des 798 villes ont déclaré que la COVID-19 avait réduit le financement disponible pour la lutte contre les changements climatiques (indicateur 2.1.3). Alors que les pays cherchent des alternatives au pétrole et au gaz russes, beaucoup continuent de favoriser la combustion de combustibles fossiles, certains se tournant même vers le charbon. Les changements dans l'approvisionnement énergétique mondial menacent d'augmenter la production de combustibles fossiles. Même si elles étaient mises en œuvre en tant que transition temporaire, ces réponses pourraient inverser les progrès en matière d'amélioration de la qualité de l'air, ce qui pousserait irréversiblement le monde à s'écarter des engagements énoncés dans l'Accord de Paris et garantir un avenir de changements climatiques accélérés qui menacent la survie des populations.

Au contraire, en ce moment charnière, une réponse axée sur la santé aux crises actuelles offrirait toujours la possibilité d'un avenir résilient et à faible émission de carbone, qui non seulement évite les dommages pour la santé de l'accélération des changements climatiques, mais améliore également la santé et le bien-être grâce aux avantages connexes associés à l'action climatique. Une telle réponse permettrait aux pays de s'éloigner rapidement des combustibles fossiles, de réduire leur dépendance à l'égard des marchés internationaux fragiles du pétrole et du gaz et d'accélérer une transition équitable vers des sources d'énergie propre. Une intervention axée sur la santé réduirait la probabilité des effets les plus catastrophiques des changements climatiques, tout en améliorant la sécurité énergétique, en créant des opportunités de reprise économique et en offrant des avantages immédiats pour la santé. L'amélioration de la qualité de l'air contribuerait à prévenir les 1,2 million de décès résultant d'une exposition aux PM2,5 ambiantes dérivées des combustibles fossiles rien qu'en 2020 (indicateur 3.3), et une transition énergétique axée sur la santé favoriserait les déplacements à faibles émissions de carbone et augmenterait les espaces verts urbains, encouragerait l'activité physique et améliorerait la santé physique et mentale. Dans le secteur alimentaire, une transition accélérée vers des régimes alimentaires équilibrés et davantage à base de plantes contribuerait non seulement à réduire les 55% des émissions du secteur agricole

provenant de la production de viande rouge et de lait (indicateur 3.5.1), mais aussi à prévenir jusqu'à 11,5 millions de décès liés à l'alimentation chaque année (indicateur 3.5.2) et à réduire considérablement le risque de maladies zoonotiques. Ces changements axés sur la santé réduiraient le fardeau des maladies transmissibles et non transmissibles, réduisant ainsi la pression sur les prestataires de soins de santé débordés. Il est important de noter que l'accélération de l'adaptation aux changements climatiques conduirait à des systèmes de santé plus robustes, minimiserait les impacts négatifs des futures épidémies de maladies infectieuses et des conflits géopolitiques, et rétablirait la première ligne de défense des populations mondiales.

Des lueurs d'espoir émergentes

Malgré des décennies de mesures insuffisantes, les signes de changement émergents, bien que peu nombreux, donnent l'espoir qu'une réponse axée sur la santé pourrait commencer à émerger. L'engagement individuel à l'égard des dimensions sanitaires des changements climatiques, essentiel pour stimuler et permettre une réponse accélérée, a augmenté de 2020 à 2021 (indicateur 5.2), et la couverture médiatique de la santé et des changements climatiques a atteint un nouveau record en 2021, avec une augmentation de 27% par rapport à 2020 (indicateur 5.1). Cet engagement est également reflété par les dirigeants des pays, avec un record de 60% des 194 pays concentrant leur attention sur les liens entre les changements climatiques et la santé dans le débat général de l'ONU de 2021, et avec 86% des CDN nationales mises à jour ou nouvelles faisant référence à la santé (indicateur 5.4). Au niveau des villes, les autorités locales identifient progressivement les risques des changements climatiques sur la santé de leurs populations (indicateur 2.1.3), première étape vers une réponse adaptée qui renforce les systèmes de santé locaux. Bien que le secteur de la santé soit responsable de 5,2% de toutes les émissions mondiales (indicateur 3.6), il a fait preuve d'un leadership climatique impressionnant, et 60 pays se sont engagés à faire la transition vers une économie résiliente au climat et/ou à faibles émissions de carbone ou à zéro émission nette de carbone dans le cadre du programme de santé de la COP26, en juillet 2022.

Des signes de changement apparaissent également dans le secteur de l'énergie. Bien que la production totale d'énergie propre reste nettement insuffisante, des niveaux record ont été atteints en 2020 (indicateur 3.1). Les sources zéro carbone ont représenté 80% des investissements dans la production d'électricité en 2021 (indicateur 4.2.1), et les énergies renouvelables ont atteint la parité des coûts avec les énergies fossiles. Alors que certains des pays les plus émetteurs tentent de réduire leur dépendance au pétrole et au gaz en réponse à la guerre en Ukraine et à la flambée des prix de l'énergie, beaucoup se concentrent sur l'augmentation de la production d'énergie renouvelable, suscitant l'espoir d'une réponse axée sur la santé. Cependant, une prise de conscience et des engagements accrus doivent être traduits d'urgence en actions pour que l'espoir devienne réalité.

Un appel à l'action

Après 30 ans de négociations de la CCNUCC, les indicateurs du Compte à rebours du Lancet montrent que les pays et les entreprises continuent de faire des choix qui menacent de plus en plus la santé et la survie des populations dans toutes les parties du monde. Alors que les pays cherchent les moyens de se remettre des crises concomitantes, les preuves sont sans équivoque à ce stade critique : une réponse immédiate, alignée et centrée sur la santé est essentielle pour assurer un avenir viable et présente une nouvelle opportunité de créer un monde dans lequel les populations présentes et futures peuvent non seulement survivre, mais également prospérer.
