# Supplementary material for: The 2022 report of the Lancet Countdown on health and climate change: health at the mercy of fossil fuels
Source: Lancet. Author manuscript; Available in PMC 2024 Nov 15. (PMC7616806; doi:10.1016/S0140-6736(22)01540-9)
Supplement: Spanish translation of the executive summary [file EMS200004-supplement-Spanish_translation_of_the_executive_summary.pdf]

THE LANCET

Supplementary appendix 3

This translation in Spanish was submitted by the authors and we reproduce it as supplied. It has not been peer reviewed. *The Lancet*'s editorial processes have only been applied to the original in English, which should serve as reference for this manuscript.

Los autores nos proporcionaron esta traducción al español y la reproducimos tal como nos fue entregada. No la hemos revisado. Los procesos editoriales de *The Lancet* se han aplicado únicamente al original en inglés, que debe servir de referencia para este manuscrito.

Supplement to: Romanello M, Di Napoli C,Drummond P, et al. The 2022 report of the *Lancet* Countdown on health and climate change: health at the mercy of fossil fuels. *Lancet* 2022; published online Oct 25. https://doi.org/10.1016/S0140-6736(22)01540-9.

El informe 2022 de *Lancet* Countdown sobre salud y cambio climático: la salud a merced de los combustibles fósiles

Resumen ejecutivo

El informe 2022 de Lancet Countdown se publica cuando el mundo se enfrenta a profundas y actuales crisis sistémicas. Los países y los sistemas sanitarios siguen lidiando con las repercusiones sanitarias, sociales y económicas de la pandemia de COVID-19, mientras que la invasión rusa de Ucrania y la persistente dependencia excesiva de los combustibles fósiles han empujado al mundo a una crisis energética y del coste de la vida. Mientras estas crisis se desarrollan, el cambio climático se intensifica sin cesar. El empeoramiento de sus efectos está afectando cada vez más a los fundamentos de la salud y el bienestar de los seres humanos, lo que exacerba la vulnerabilidad de las poblaciones del mundo ante las amenazas sanitarias concurrentes.

Durante 2021 y 2022, los fenómenos meteorológicos extremos causaron devastación en todos los continentes, añadiendo más presión a los servicios de salud que ya lidiaban con las repercusiones de la pandemia de COVID-19. Las inundaciones en Australia, Brasil, China, Europa occidental, Malasia, Pakistán, Sudáfrica y Sudán del Sur provocaron miles de muertes, desplazaron a cientos de miles de personas y ocasionaron miles de millones de dólares en pérdidas económicas. Los incendios forestales causaron estragos en Canadá, Estados Unidos, Grecia, Argelia, Italia, España y Turquía, y se registraron temperaturas récord en muchos países, como Australia, Canadá, India, Italia, Omán, Turquía, Pakistán y el Reino Unido. Gracias a los avances en la ciencia de los estudios de detección y atribución, se ha cuantificado la influencia del cambio climático en muchos fenómenos.

Debido al rápido aumento de las temperaturas, las poblaciones vulnerables (adultos mayores de 65 años y niños menores de un año) que abarca 37000 millones estuvieron expuestas a días más de olas de calor en 2021 que en 1986-2005 (indicador 1.1.2), y las muertes relacionadas con el calor aumentaron un 68 % entre 2000-04 y 2017-21 (indicador 1.1.5), un número de muertes que se agravó significativamente por la confluencia de la pandemia de COVID-19.

Al mismo tiempo, el cambio climático está afectando a la propagación de enfermedades infecciosas, poniendo a las poblaciones en mayor riesgo de enfermedades emergentes y coepidemias. Las aguas costeras se están volviendo más aptas para la transmisión de patógenos del género Vibrio; el número de meses aptos para la transmisión de la malaria aumentó en un 31,3 % en las zonas altas de las Américas y en un 13,8 % en las zonas altas de África desde 1951-60 hasta 2012-21, y la probabilidad de transmisión del dengue aumentó en un 12 % en el mismo periodo (indicador 1.3.1). La coexistencia de los brotes de dengue con la pandemia de COVID-19 provocó el agravamiento de la presión sobre los sistemas sanitarios, los diagnósticos erróneos y las dificultades en el manejo de ambas enfermedades en muchas regiones de América del Sur, Asia y África.

Las pérdidas económicas asociadas a las repercusiones del cambio climático también están aumentando la presión sobre las familias y las economías que ya se enfrentan a los efectos sinérgicos de la pandemia de COVID-19 y las crisis internacionales del coste de la vida y de la energía, lo que socava aún más los determinantes socioeconómicos de los que depende la buena salud. La exposición al calor provocó la pérdida de 470 000 millones de horas potenciales de trabajo en todo el mundo en 2021 (indicador 1.1.4), con pérdidas potenciales de ingresos equivalentes al 0.72 % de la producción económica mundial, que aumentan hasta el 5,6 % del PIB en los países con un Índice

de Desarrollo Humano (IDH) bajo, donde los trabajadores son más vulnerables a los efectos de las fluctuaciones financieras (indicador 4.1.3). Mientras tanto, los fenómenos meteorológicos extremos produjeron daños por valor de 253 000 millones de dólares en 2021, que afectaron especialmente a la población de los países con un IDH bajo, en los que casi ninguna de las pérdidas estaba asegurada (indicador 4.1.1).

A través de vías múltiples e interconectadas, todas las dimensiones de la seguridad alimentaria se ven afectadas por el cambio climático, agravando los impactos de otras crisis coexistentes. El aumento de las temperaturas amenaza directamente el rendimiento de los cultivos, ya que las temporadas de crecimiento del maíz se acortarán de media 9 días en 2020, y las del trigo de invierno y el trigo de primavera 6 días menos que en el período 1981-2010 a nivel mundial (indicador 1.4). La amenaza para el rendimiento de los cultivos se suma al creciente impacto del clima extremo en las cadenas de suministro, las presiones socioeconómicas y el riesgo de transmisión de enfermedades infecciosas, lo que socava la disponibilidad, el acceso, la estabilidad y la utilización de los alimentos. Un nuevo análisis sugiere que el calor extremo se asoció con 98 millones más de personas que declararon inseguridad alimentaria de moderada a severa en 2020 que en 1981-2010, en 103 países analizados (indicador 1.4). El clima cada vez más extremo empeora la estabilidad de los sistemas alimentarios mundiales, actuando en sinergia con otras crisis concurrentes para revertir el progreso hacia la erradicación del hambre. De hecho, la prevalencia de la subnutrición aumentó durante la pandemia de COVID-19, y hasta 161 millones de personas más se enfrentaron al hambre durante la pandemia de COVID-19 en 2020 que en 2019. Esta situación se ve ahora agravada por la invasión rusa de Ucrania y las crisis energética y del coste de la vida, con impactos en la producción agrícola internacional y en las cadenas de suministro que amenazan con provocar que 13 millones de personas más se enfrenten a la desnutrición en 2022.

Una primera línea de defensa debilitada

Con el empeoramiento de las repercusiones sanitarias del cambio climático que agrava otras crisis coexistentes, las poblaciones de todo el mundo dependen cada vez más de los sistemas sanitarios como primera línea de defensa. Sin embargo, al mismo tiempo que aumenta la necesidad de atención sanitaria, los sistemas de salud de todo el mundo se ven debilitados por los efectos de la pandemia del COVID-19 y la crisis energética y del coste de la vida. Por lo tanto, es necesario adoptar medidas urgentes para reforzar la capacidad de recuperación de los sistemas sanitarios y evitar una rápida escalada de pérdidas de vidas y sufrimiento en un clima cambiante. Sin embargo, solo 48 (51 %) de los 95 países informaron de que habían evaluado sus necesidades de adaptación al cambio climático (indicador 2.1.1) e, incluso después de los profundos impactos de la COVID-19, solo 60 (63 %) países informaron de un estado de implementación alto o muy alto para la gestión de las emergencias sanitarias en 2021 (indicador 2.2.4).

La escasez de adaptación proactiva se muestra en la respuesta al calor extremo. A pesar de los beneficios locales de refrigeración y de salud en general de los espacios verdes urbanos, solo 277 (27 %) de los 1038 centros urbanos mundiales eran al menos moderadamente verdes en 2021 (indicador 2.2.3), y el número de hogares con aire acondicionado aumentó un 66 % de 2000 a 2020, una respuesta inadaptada que agrava la crisis energética y aumenta aún más el calor urbano, la contaminación del aire y las emisiones de gases de efecto invernadero.

A medida que las crisis convergentes amenazan aún más los sistemas de apoyo a la vida en el mundo, es esencial una acción intersectorial rápida, decisiva y coherente para proteger la salud humana de los peligros del rápido cambio climático.

La salud a merced de los combustibles fósiles

En 2022 se cumple el 30.º aniversario de la firma de la Convención Marco de las Naciones Unidas sobre el Cambio Climático, en la que los países acordaron prevenir el peligroso cambio climático antropogénico y sus efectos nocivos para la salud y el bienestar humanos. Sin embargo, desde entonces se han tomado pocas medidas significativas. La intensidad de carbono del sistema energético mundial ha disminuido en menos de un 1 % desde que se estableció la CMNUCC, y la generación mundial de electricidad sigue estando dominada por los combustibles fósiles, con una contribución de las energías renovables de solo el 8,2 % del total mundial (indicador 3.1). Al mismo tiempo, la demanda total de energía ha aumentado un 59 %, lo que incrementará las emisiones relacionadas con la energía hasta alcanzar un máximo histórico en 2021. Las políticas actuales sitúan al mundo en la senda de un aumento catastrófico de 2,7 °C para finales de siglo. Incluso con los compromisos que los países establecieron en las Contribuciones Determinadas a Nivel Nacional (CDN) actualizadas hasta noviembre de 2021, las emisiones mundiales podrían estar en un 13,7 % por encima de los niveles de 2010 para 2030, muy lejos de la disminución del 43 % con respecto a los niveles actuales necesaria para cumplir los objetivos del Acuerdo de París y mantener las temperaturas dentro de los límites de la adaptación.

La dependencia de los combustibles fósiles no solo está socavando la salud mundial por el aumento de los efectos del cambio climático, sino que también afecta directamente a la salud y el bienestar de las personas, por la volatilidad e imprevisibilidad de los mercados de combustibles fósiles, la fragilidad de las cadenas de suministro y los conflictos geopolíticos. En consecuencia, millones de personas no tienen acceso a la energía necesaria para mantener sus hogares a temperaturas saludables, conservar los alimentos y los medicamentos, y cumplir el 7.º Objetivo de Desarrollo Sostenible (garantizar el acceso a una energía asequible, fiable, sostenible y moderna para todos). Sin el apoyo suficiente, el acceso a la energía limpia ha sido especialmente lento en los países con un IDH bajo, y solo entre el 1,4 % de su electricidad procedía de energías renovables modernas (principalmente eólica y solar) en 2020 (indicador 3.1). Se calcula que el 59 % de los centros de salud de los países de renta baja y media aún no tienen acceso a la electricidad fiable necesaria para prestar la atención básica. Mientras tanto, la biomasa representa hasta el 31 % de la energía consumida en el sector doméstico a nivel mundial, en su mayoría procedente de fuentes tradicionales, una proporción que aumenta hasta el 96 % en los países de IDH bajo (indicador 3.2). La carga de enfermedad asociada es considerable, ya que el aire de los hogares supera las directrices de la OMS sobre las concentraciones seguras de contaminación atmosférica por pequeñas partículas (PM2-5) en 2020 en una media de 30 veces en los 62 países evaluados (indicador 3.2). Después de 6 años de mejora, el número de personas sin acceso a la electricidad aumentó en 2020 como resultado de las presiones socioeconómicas de la pandemia de COVID-19. La actual crisis energética y del coste de la vida amenaza ahora con revertir los avances hacia una energía asequible, fiable y sostenible, socavando aún más los determinantes socioeconómicos de la salud.

Simultáneamente, las empresas de gas y petróleo registran beneficios récord, mientras que sus estrategias de producción siguen minando la vida y el bienestar de las personas. Un análisis de las estrategias de producción de 15 de las mayores empresas de gas y petróleo del mundo, a fecha de febrero de 2022, reveló que superan su cuota de emisiones consistente con 1,5 °C de calentamiento global (indicador 4.2.6) en un 37 % en 2030 y en un 103 % en 2040, lo que sigue socavando los esfuerzos por ofrecer un futuro bajo en carbono, saludable y habitable. Para agravar aún más esta situación, los gobiernos siguen incentivando la producción y el consumo de combustibles fósiles: 69 (80 %) de los 86 países examinados tenían precios del carbono netos negativos (es decir, proporcionaban una subvención neta a los combustibles fósiles) por un total neto de 400 000

millones de dólares en 2019, asignando cantidades a menudo comparables con sus presupuestos sanitarios totales o incluso superiores a ellos (indicador 4.2.4). Simultáneamente, los países más ricos no cumplieron su compromiso de movilizar la suma considerablemente menor de 100 000 millones de dólares anuales para 2020, según lo acordado en el Acuerdo de Copenhague de 2009, para apoyar la acción climática en los "países en desarrollo", y los esfuerzos climáticos se ven minados por una profunda escasez de financiación (indicador 2.1.1). Los impactos del cambio climático en las economías mundiales, junto con la recesión desencadenada por la COVID-19 y agravada por la inestabilidad geopolítica, podrían, paradójicamente, reducir aún más la voluntad de los países de asignar los fondos necesarios para permitir una transición climática justa.

Una respuesta centrada en la salud para un futuro próspero

El mundo se encuentra en una coyuntura crítica. Con los países que se enfrentan a crisis simultáneas, la implementación de políticas de reducción de emisiones a largo plazo corre el riesgo de ser desviada o derrotada por retos erróneamente percibidos como más inmediatos. Si se aborda cada una de las crisis concurrentes de forma aislada, se corre el riesgo de aliviar una de ellas y empeorar otra. Tal situación se desprende de la respuesta a la COVID-19, que hasta ahora no ha aportado la recuperación ecológica que la comunidad sanitaria proponía y, por el contrario, está agravando los riesgos sanitarios relacionados con el cambio climático. Es probable que menos de un tercio de los 3,11 billones de dólares destinados a la recuperación económica de COVID-19 reduzcan las emisiones de gases de efecto invernadero o la contaminación atmosférica, y que el efecto neto sea un aumento de las emisiones. La pandemia de COVID-19 afectó a la acción climática a nivel de las ciudades, y 239 (30 %) de 798 ciudades informaron de que COVID-19 redujo la financiación disponible para la acción climática (indicador 2.1.3). Mientras los países buscan alternativas al gas y petróleo rusos, muchos siguen favoreciendo la quema de combustibles fósiles, y algunos incluso vuelven al carbón. Los cambios en el suministro energético mundial amenazan con aumentar la producción de combustibles fósiles. Incluso si se aplican como una transición temporal, estas respuestas podrían revertir los avances en la mejora de la calidad del aire, alejar al mundo de forma irreversible del cumplimiento de los compromisos establecidos en el Acuerdo de París y garantizar un futuro de cambio climático acelerado que amenace la supervivencia humana.

Por el contrario, en este momento crucial, una respuesta a la crisis actual centrada en la salud ofrecería la oportunidad de un futuro bajo en carbono y resiliente, que no solo evitaría los daños a la salud del cambio climático acelerado, sino que también mejoraría la salud y el bienestar gracias a los beneficios colaterales de la acción climática. Esta respuesta permitiría a los países abandonar rápidamente los combustibles fósiles, reducir su dependencia de los frágiles mercados internacionales del gas y petróleo, y acelerar una transición justa hacia fuentes de energía limpias. Una respuesta centrada en la salud reduciría la probabilidad de los efectos más catastróficos del cambio climático, al tiempo que mejoraría la seguridad energética, crearía una oportunidad de recuperación económica y ofrecería beneficios inmediatos para la salud. Las mejoras en la calidad del aire ayudarían a evitar los 1,2 millones de muertes resultantes de la exposición a las PM2-5 ambientales derivadas de los combustibles fósiles solo en 2020 (indicador 3.3), y una transición energética centrada en la salud potenciaría los viajes con bajas emisiones de carbono y aumentaría los espacios verdes urbanos, promoviendo la actividad física y mejorando la salud física y mental. En el sector alimentario, una transición acelerada hacia dietas equilibradas y más basadas en plantas no solo ayudaría a reducir el 55 % de las emisiones del sector agrícola procedentes de la producción de carne roja y leche (indicador 3.5.1), sino que también evitaría hasta 11,5 millones de muertes anuales relacionadas con la dieta (indicador 3.5.2), y reduciría sustancialmente el riesgo de enfermedades zoonóticas. Estos cambios centrados en la salud reducirían la carga de enfermedades

transmisibles y no transmisibles, reduciendo la presión sobre los abrumados proveedores de atención sanitaria. Y lo que es más importante, acelerar la adaptación al cambio climático conduciría a sistemas sanitarios más robustos, minimizando los efectos negativos de futuros brotes de enfermedades infecciosas y conflictos geopolíticos, y restaurando la primera línea de defensa de las poblaciones mundiales.

Los primeros destellos de esperanza

A pesar de décadas de acciones insuficientes, los incipientes, aunque escasos, signos de cambio proporcionan cierta esperanza de que pueda estar empezando a surgir una respuesta centrada en la salud. El compromiso individual con las dimensiones sanitarias del cambio climático, esencial para impulsar y permitir una respuesta acelerada, aumentó de 2020 a 2021 (indicador 5.2), y la cobertura de la salud y el cambio climático en los medios de comunicación alcanzó un nuevo récord en 2021, con un aumento del 27 % respecto a 2020 (indicador 5.1). Este compromiso también se refleja en los líderes de los países, ya que un 60 % de los 194 países, cifra récord, centraron su atención en los vínculos entre el cambio climático y la salud en el Debate General de la ONU de 2021, y el 86 % de las CND nacionales actualizadas o nuevas hacen referencia a la salud (indicador 5.4). A nivel de las ciudades, las autoridades locales están identificando progresivamente los riesgos del cambio climático para la salud de sus poblaciones (indicador 2.1.3), un primer paso para dar una respuesta adaptada que refuerce los sistemas sanitarios locales. Aunque el sector sanitario es responsable de entre el 5,2 % de todas las emisiones mundiales (indicador 3.6), ha demostrado un impresionante liderazgo climático, y 60 países se han comprometido a realizar la transición a sistemas de salud resistentes al clima o con bajas emisiones de carbono o con emisiones netas de carbono como parte del Programa de Salud de la COP26, a partir de julio de 2022.

También están apareciendo señales de cambio en el sector energético. Aunque la generación total de energía limpia sigue siendo muy insuficiente, en 2020 se alcanzaron niveles récord (indicador 3.1). Las fuentes de carbono cero representaron el 80 % de la inversión en generación de electricidad en 2021 (indicador 4.2.1), y las energías renovables han alcanzado la paridad de costes con las energías de combustibles fósiles. Mientras algunos de los países que más emiten intentan reducir su dependencia del gas y petróleo en respuesta a la guerra de Ucrania y al aumento de los precios de la energía, muchos se centran en aumentar la generación de energía renovable, lo que hace esperar una respuesta centrada en la salud. Sin embargo, la mayor concienciación y los compromisos deben traducirse urgentemente en acciones para que la esperanza se convierta en realidad.

Una llamada a la acción

Tras 30 años de negociaciones de la CMNUCC, los indicadores de Lancet Countdown muestran que los países y las empresas siguen tomando decisiones que amenazan cada vez más la salud y la supervivencia de las personas en todas las partes del mundo. Mientras los países idean formas de recuperarse de las crisis coexistentes, la evidencia es inequívoca en esta coyuntura crítica: una respuesta inmediata, alineada y centrada en la salud es esencial para asegurar un futuro habitable y presenta una nueva oportunidad para ofrecer un mundo en el que las poblaciones presentes y futuras no solo puedan sobrevivir, sino prosperar.
