# Supplementary material for: The 2022 report of the Lancet Countdown on health and climate change: health at the mercy of fossil fuels
Source: Lancet. Author manuscript; Available in PMC 2024 Nov 15. (PMC7616806; doi:10.1016/S0140-6736(22)01540-9)
Supplement: Supplementary appendix [file EMS200004-supplement-Supplementary_appendix.pdf]

THE LANCET

Supplementary appendix

This appendix formed part of the original submission and has been peer reviewed. We post it as supplied by the authors.

Supplement to: Romanello M, Di Napoli C,Drummond P, et al. The 2022 report of the *Lancet* Countdown on health and climate change: health at the mercy of fossil fuels. *Lancet* 2022; published online Oct 25. https://doi.org/10.1016/ S0140-6736(22)01540-9.

The 2022 Report of the *Lancet* Countdown on Health and Climate Change

Appendix

The 2022 Report of the *Lancet* Countdown on Health and Climate Change

This appendix provides methodological details on each of the *Lancet* Countdown's indicators, alongside data sources used, and caveats. Wherever suitable, future plans for the indicators and further analysis are also presented.

Wherever possible and appropriate, each indicator is disaggregated into very high, high, medium, and low human development index (HDI) country groups, as defined by the UNDP. For this purpose, the attained level of HDI in the latest year of data available during the writing of this report (2019) is used, acknowledging that the achievement of a HDI level is the product of several years of work towards improving the parameters that define it. The HDI captures three core dimensions: a long and healthy life (using life expectancy as a proxy), education (monitored by the mean of years of Schooling in a given country), and standard of living (using per-capita gross national income as a proxy).

Unless otherwise specified, the indicators that incorporate retrospective climate data make use of the climate reanalysis datasets, mostly ERA5, but also including ERA5-Land and ORAS5. These datasets incorporate vast amounts of historical observations, including those from satellites, to provide the most complete description of the observed climate as it has evolved during recent decades. Due to their temporal and geographical coverage, these are the most appropriate data for the purposes of the *Lancet* Countdown indicators. Slight discrepancies might exist between reanalysis datasets, and other types of retrospective climatological modelling, which however would only have slight impacts on findings of the indicators here presented.

All monetary values in the *Lancet* Countdown are expressed in 2021 US dollars, unless stated otherwise in the main text or cited sources.

Section 1: Health Hazards, Exposures and Impacts

1.1: Heat and Health

Indicator 1.1.1: Exposure to Warming

Methods

The input data for this indicator have been improved and extended for the 2022 report.

The indicator uses monthly temperature from European Centre for Medium-Range Weather Forecasts (ECMWF) ERA5 climate reanalysis dataset. From this, a baseline global mean temperature grid was first calculated as the average of summer temperatures (June, July, August for the northern hemisphere, December, January, February for the southern hemisphere) from 1986–2005, the same period used by the Intergovernmental Panel on Climate Change (IPCC AR5)[1]. Then global summer temperature changes relative to the 1986–2005 average were calculated for every grid point for every year and weighted by true pixel area to obtain a year-by-year global average. The 'population-weighted' average was calculated by weighting each grid cell by the fraction of the total world population contained within that grid cell. This method allows the difference between global effects of climate change and the effects experienced by the human population to be highlighted.

Population data from 2000 to present are from NASA GPWv4 dataset at 0.25° x 0.25° spatial resolution, the same as ECMWF ERA5. Population data from 1980 to 2000 are from the ISIMIP Histsoc dataset at 0.5° x 0.5° spatial resolution. In the main text both the Histsoc-derived findings (1980-2000) and the GPWv4-derived findings (2000–2021) are presented.

Data

1. Climate data from the European Centre for Medium-Range Weather Forecasts (ECMWF) ERA5 reanalysis.
2. Population data from the NASA Socioeconomic Data and Applications Center (SEDAC) Gridded Population of the World (GPWv4) and The Inter-Sectoral Impact Model Intercomparison Project (ISIMIP) Histsoc dataset.[2,3]

Additional analysis

Population weighted temperatures are increasing 2.5 times faster than global mean temperatures (linear regression slope of 0.032˚C per year compared to 0.015˚C per year; p-value < 0.05). These values are higher than those found in previous Countdown reports (0.027˚C and 0.011˚C in the 2020 edition, respectively). This highlights that a) global warming is especially affecting populated areas and b) ongoing climate change prevention measures are inadequate. In 2021 the global mean summer anomaly was 0.28˚C while the population weighted summer anomaly was 0.65 ˚C relative to the 1986-2005 baseline (Figure 1). Locally, these anomalies can be significantly higher at over 5˚C (Figure 2). Analysis of the anomaly grouped by HDI (Human Development Index) level and WHO (World Health Organisation) region show that the trends are global and do not seem to indicate any particular difference across levels or regions (Figure 3, Figure 4).

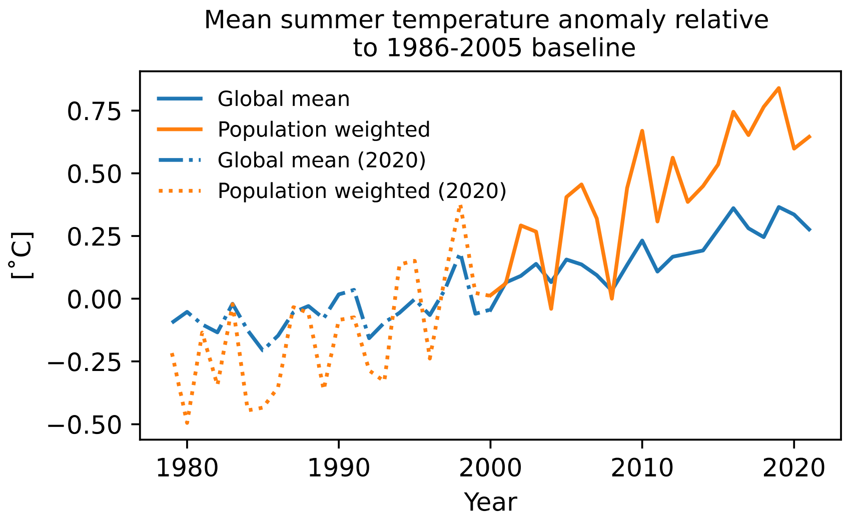

Figure 1: Global mean trends of summer temperature anomaly compared to the population weighted trend (relative to the 1986-2005 baseline). Results before 2000 are drawn from the 2020 edition of the Countdown and are calculated on the lower 0.5° grid resolution.

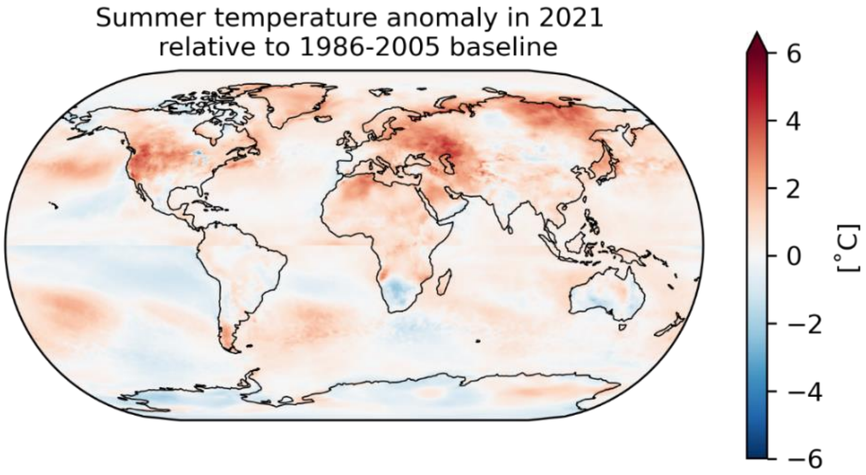

Figure 2. Map of summer temperature anomaly for 2021 relative to the 1986-2005 baseline.

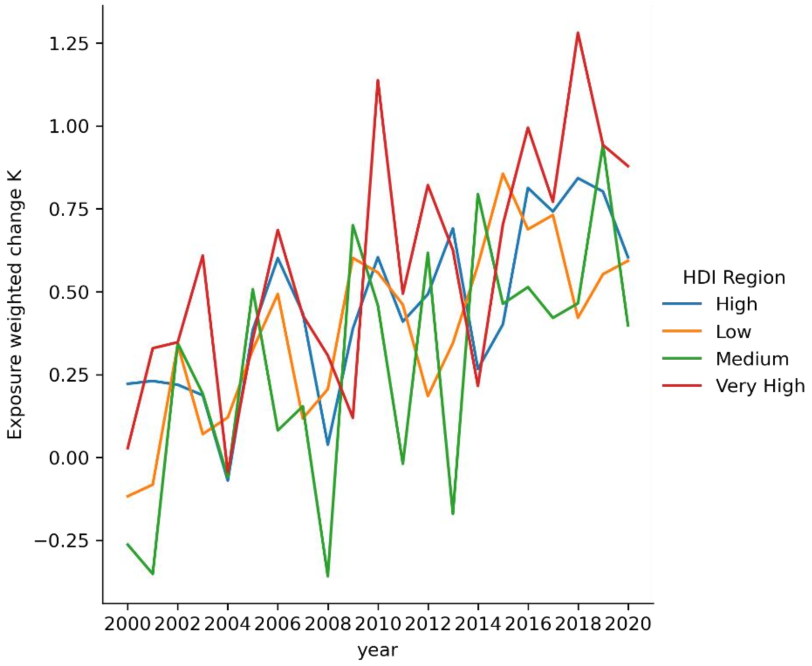

Figure 3. Exposure weighted change in summer temperatures relative to 1986-2005 baseline by HDI level.

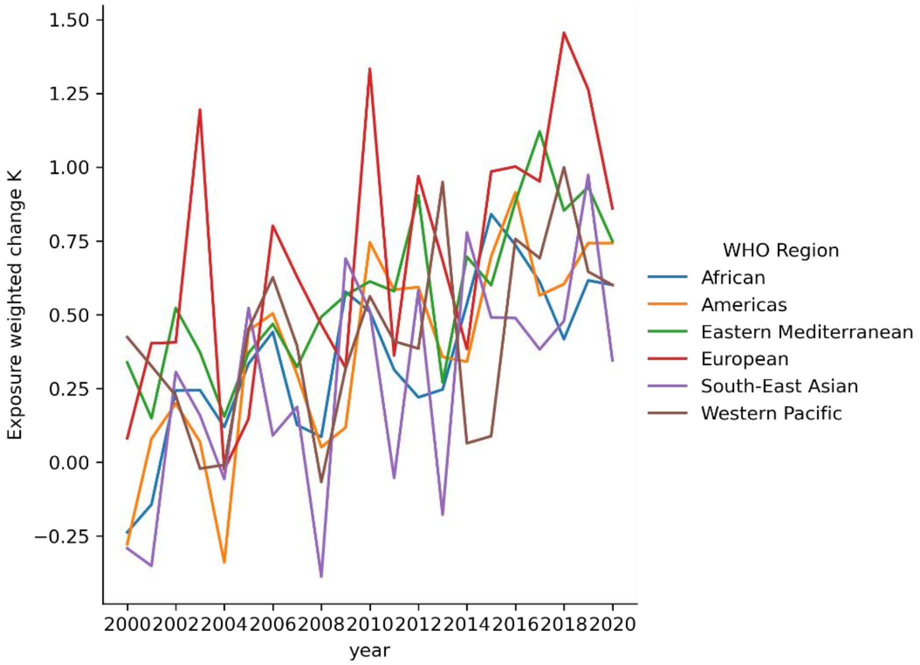

Figure 4. Exposure weighted change in summer temperatures relative to 1986-2005 baseline by WHO region.

Indicator 1.1.2: Exposure of Vulnerable Populations to Heatwaves

Methods

The input data for this indicator have been improved and extended for the 2022 report.

The indicator defines a heatwave as a period of 2 or more days where both the minimum and maximum temperatures are above the 95th percentile of the local climatology (defined on the 1986–2005 baseline). This reflects the definition from published scientific literature on the topic.[4] It also aims to capture the health effects of both direct heat extremes (i.e. caused by high maximum temperatures) and the problems associated with lack of recovery (i.e. caused by high minimum temperatures) over persisting hot periods.[5] The gridded 95th percentile of daily minimum and maximum temperatures, taken from the European Centre for Medium-Range Weather Forecasts (ECMWF) ERA5 dataset, were calculated on a 0.25° x 0.25° global grid for 1986–2005. For each year from 1980 to 2020, the number of heatwave events and total days of heatwaves per year was calculated according to the definition above.

Vulnerable populations are defined as those above the age of 65 and infants between 0 and 1 years old. Previous research has identified these groups as being particularly vulnerable to heatwave impacts on health.[6]

Data inspection has shown that increasing heatwave length can result in fewer discrete heatwave events as they merge into single long events – this is therefore better captured by the person-days metric. To reflect that and in continuity with previous reports, the exposure of vulnerable populations to heatwaves is computed as person-days, i.e., as by multiplying the number of heatwave days by vulnerable population count. In this way, the indicator captures both the changes in duration and in frequency of heatwaves, as well as the changing demographics that might mean more vulnerable people are at risk.[7]

Population and demographic data from NASA GPWv4 were used for the period 2000–2020 as their resolution matches ECMWF ERA5's. For the period pre-2000, the ISIMIP Histsoc dataset was used after being up-sampled to a 0.25° x 0.25° resolution via a 2D linear interpolation of population densities with land area data from NASA GPWv4. As the population data are discontinuous, there can be some inconsistencies between the pre and post 2000 values. Therefore, the indicator is presented as exposure to change rather than change in exposure, as this avoids calculating changes in population across the data discontinuity. The hybrid dataset, available on open access[8] and new to the 2022 report, refers to a population older than 65 years old.

The number of births minus the mortality rate of children under 1 was used as an approximation of the number of children under 1 year old. The United Nation World Population Prospects (UN WPP) data for birth rates were used. UN WPP provides Crude Birth Rate (CBR) and Infant Mortality Rate (IMR) values per country as averages for 5-year periods. To estimate the spatial distribution of births within a country, it was assumed that the spatial distribution of children under one year of age is the same as the spatial distribution of children under 5 as given by the NASA GPWv4 dataset. Furthermore, it was assumed that the IMR within a country is constant for all locations, as sub-national data cannot be applied for this study. For each country, the total number of births was calculated for the mid-period year of the 5-year time periods as *Country population * CBR * (1 - IMR)*. Spatial weighting matrices were derived from the NASA GPWv4 demographic data for under-5s and used to estimate the total births number of infants for each grid cell for each country. Finally, the estimates for the years in between the mid-period years were calculated through linear interpolation.

Data

1. Climate data from the European Centre for Medium-Range Weather Forecasts (ECMWF) ERA5 reanalysis.[9]
2. Hybrid gridded demographic data for the world, 1950-2020, 0.25˚ resolution.[8]
3. Demographic data from the United Nation World Population Prospects (UN WPP).[10]

Caveats

In order to estimate the time evolution of demographics, data from diverse sources were combined in order to obtain estimates of both the spatial and temporal characteristics. This has been subject to limited validation. Some regions have limited demographic data. Others show changes in political boundaries which can cause discontinuities in the spatial assignment of demographic values (e.g. the split in Sudan can be seen as sections of missing data for Infants in Figure 3).

Future form of the indicator

Future versions of the indicator aim to use ECMWF ERA5-Land data at 0.1˚ x 0.1° spatial resolution. The increased data volume at the global level, plus the need to adapt corresponding population data, requires upgrades to the data processing.

Additional analysis

Figure 5 summarises the change in number of heatwave days in 2021 relative to the baseline. Intense events in the Western USA, central Europe, Russia, and the Middle east, Northwest Africa, Central Africa, and South-West Africa and Madagascar are evident. Figure 6 highlights that absolute exposures are larger in the over-65 age group. However, as shown in Figure 9, in the 'low' HDI class countries the exposure of over 65s is much lower than the other classes whereas the values for infants follow the same pattern as the other classes. This is likely related to lower life expectancy in countries in the 'low' HDI class. This trend is not reflected in the breakdown by country or WHO region (Figure 7, Figure 10).

In order to better understand the magnitude of changes in heatwave exposure, comparisons of the total exposure counts (i.e. not relative to the baseline heatwave count) between first and second decades of the 20th century are carried out. Figure 8 illustrates that some regions (notably Africa) have experienced changes of over +400% person-days of heatwave (i.e. 5 times as many person-days) between these two decades.

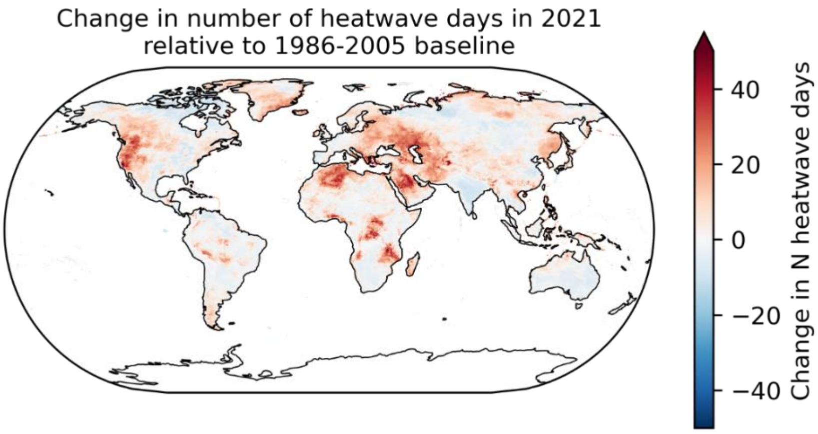

Figure 5. Map of the change in number of heatwave days over land in 2021 relative to the 1986-2005 baseline.

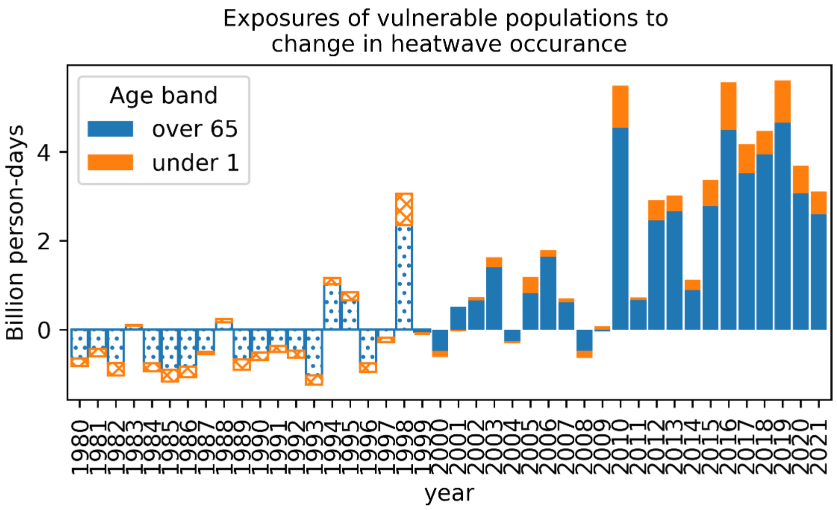

Figure 6. Exposure of people over 65 and infants under 1 year old to change in number of heatwave days relative to the 1986-2005 baseline mean number of days. Hatched bars indicate to calculations using population data from ISIMIP for 1980-2000. Block shaded bars indicate calculations using GPWv4 for 2000-2020.

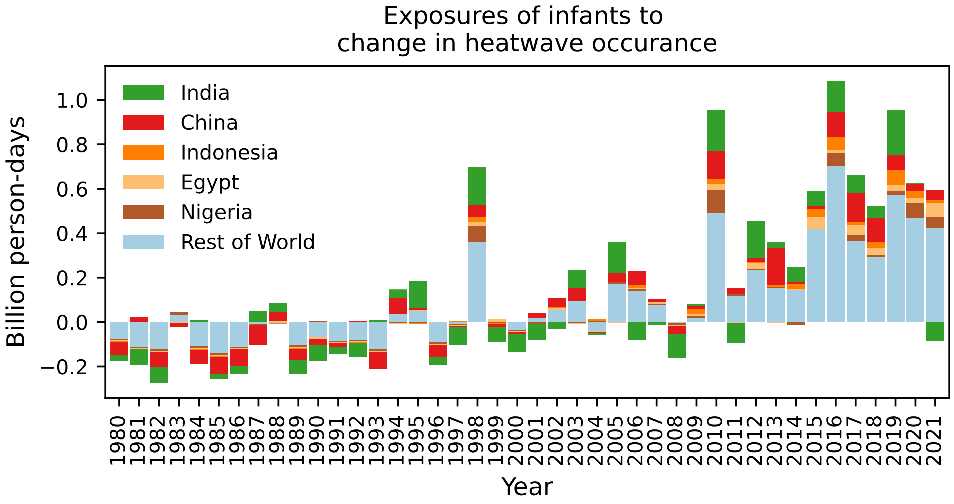

a)

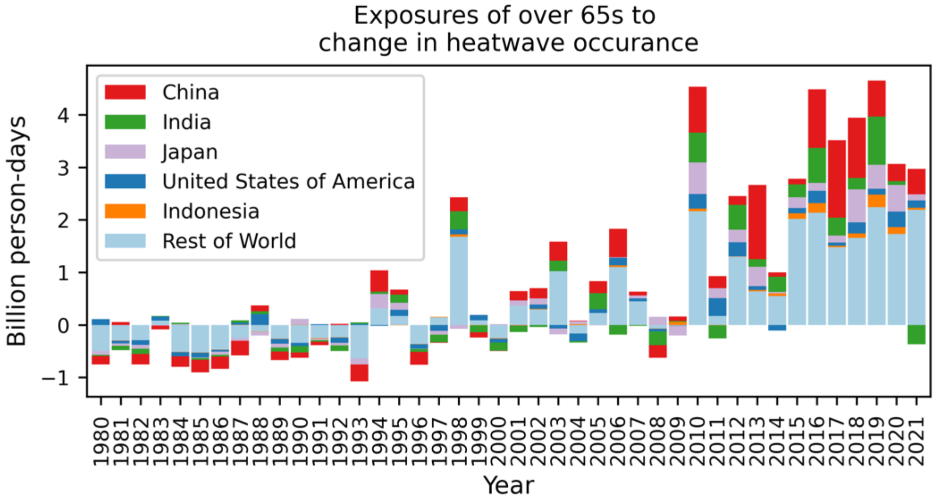

b)

Figure 7. Total exposure of a) infants and b) people over 65 to change in number of heatwave days relative to the 1986-2005 baseline mean number of days

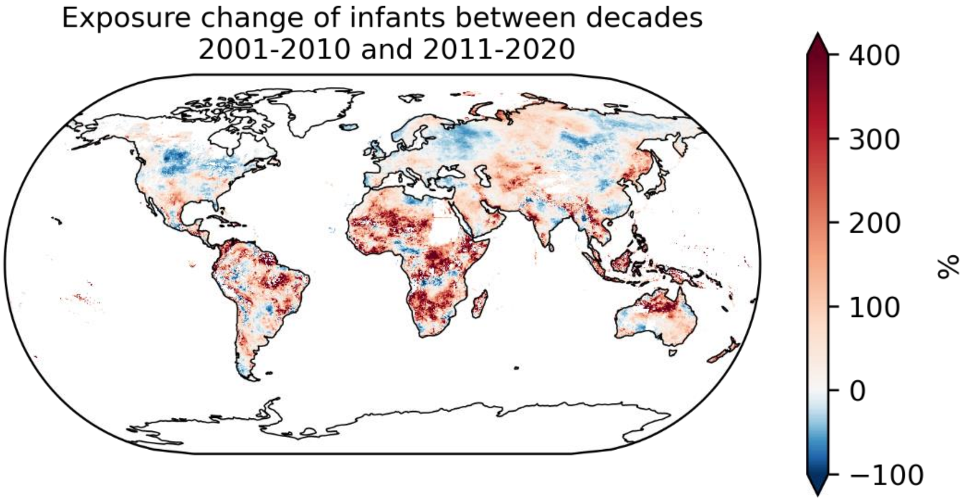

a)

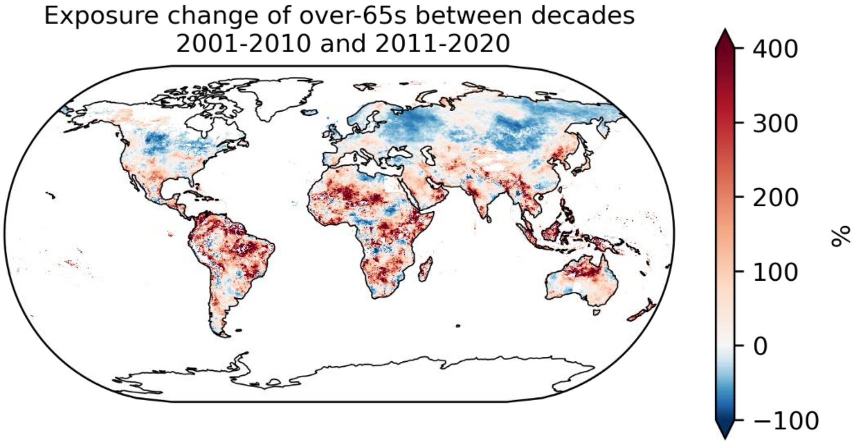

b)

Figure 8. Percentage change in heatwave exposure person-days per year between the 10-year average of 2001-2010 and 2011-2020 for a) infants and b) over-65s.

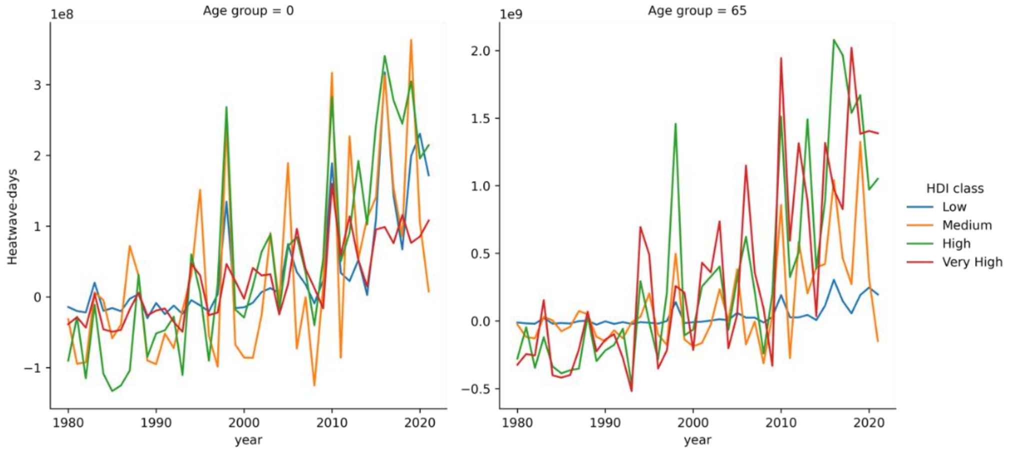

Figure 9. Exposure to change in heatwave days aggregated by HDI level.

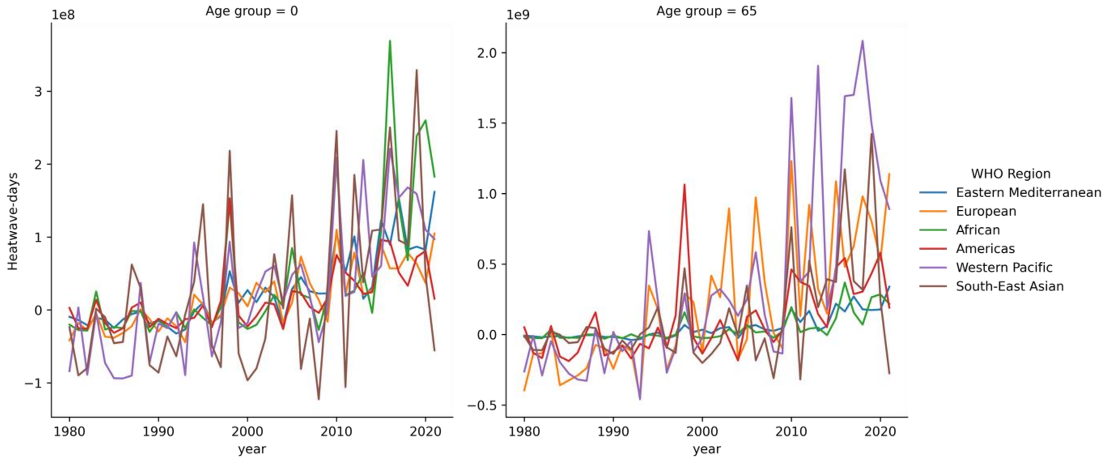

Figure 10. Exposure to change in heatwave days aggregated by WHO region.

Indicator 1.1.3: Heat and Physical Activity

Methods

The methodology for this indicator has been updated and improved from the 2021 report of the *Lancet* Countdown.[11]

Hourly temperature and dew point temperature hourly data were retrieved from European Centre for Medium-Range Weather Forecasts (ECMWF) ERA5 climate reanalysis dataset. While ERA5 data are available from 1979, data from the years 1991 to 2021 were considered for the purposes of this analysis.

Heat stress risk was estimated from these variables in accordance with the 2021 Sports Medicine Australia Extreme Heat Policy, which stratifies estimated heat stress risk into four categories – low, moderate, high, and extreme – based on ambient temperature and relative humidity.[12] Sports and activities are further classified into five risk classification groups based on intensity of the activity and clothing worn.[12] For the purposes of this analysis, the lowest sport risk classification, leisurely walking, was used, as this indicator is meant to be applied to general populations rather than elite athletic populations.

The number of hours in each grid cell with a recorded temperature and humidity combination that exceeded at least the threshold for “moderate”, “high”, and “extremely high” heat stress risk was tabulated for each year from 1991 to 2021. Specifically, the temperature-dependent humidity thresholds were defined using the following functions:

Moderate heat stress risk:

$$f(x) = 312.8741\quad 7 - 2.9756\quad x + 0.7193\quad x2 - 0.0251\quad x3 + 0.0003x4$$

High heat stress risk:

$$f(x) = 534.9217 - 28.1026x + 0.4571{\wedge}2 - 0.00017x3 - 0.000046{\wedge}4$$

Extreme heat stress risk:

$$f(x) = 525.3525 - 26.7262x + 0.4828x2 - 0.00271x3 - 0.000012x4$$

where x is 2-metre temperature in a given hour and $f(x)$ is 2-metre relative humidity (derived from dew point temperature) in a given hour. These threshold functions are defined by Sports Medicine Australia as the boundary above which the risk of heat illness changes and preventive action should be taken:[12]

“moderate” heat stress risk: additional rest breaks should be undertaken

“high” heat stress risk: active cooling strategies (e.g., water dousing) should be implemented

“extreme” heat stress risk: activities should be suspended due to heat

The total number of hours per year exceeding each threshold in each grid cell was then weighted by population. Population weighting was performed by multiplying the number of hours per year that at least exceeded each threshold by the population, as provided by the NASA GPWv4 dataset, in the respective grid cell. The population-weighted potential hours at least exceeding each threshold in a single year were added up for all grid cells in a given country, and these values were divided by the total population of the country in that year to calculate the number of hours per person that at least exceeded the “moderate”, “high” and “extreme” heat stress risk thresholds

Data

1. Climate data from the European Centre for Medium-Range Weather Forecasts (ECMWF) ERA5 reanalysis[9]
2. Population data from the NASA Socioeconomic Data and Applications Center (SEDAC) Gridded Population of the World (GPWv4)[2]

Caveats

It is acknowledged that the estimation of heat stress risk for a given exercise category may not be uniform across the entire population, and that risk estimates in particular may be different for young children and pregnant women. A more detailed interpretation model of heat effects on exercise would incorporate individual factors such as age, health status, physiology, and clothing.[13] Population data for 2021 and 2020 were not available, so population data for these two years were estimated as follows: The fraction that each grid cell represented of the world population was calculated for 2019. These fractions were held constant across 2020 and 2021, and using the estimates of the total world population, which were available for 2020 and 2021 from the United Nations, these fractions were then used to solve 2020 and 2021 populations for each grid cell . Accurate and complete numbers for 2020 and 2021will be included in next year's version and the indicator will be updated to reflect the accurate population numbers. Furthermore, it was assumed that population averages for an entire year were applicable to each hourly grid cell, which may not be accurate, but would still provide a rough estimate of population assuming an even rate of influx and outflux from each cell at the country level.

Future form of the indicator

Results will be updated using each new year of available climate data and, as sports authorities issue their updated threshold guidelines, they will be expressed according to the latest policy developments. Subsequent versions of our indicator will continue to explore methods of assessing how stratified heat stress risk has changed with time.

Additional analysis

The main analysis was conducted for the lowest sport risk classification, leisurely walking, at the level of HDI country group (Figure 11). However, it is possible to also conduct a heat stress risk assessment for a higher sport risk classification representing cycling or running, which for the same heat stress risk, there is a cooler/drier threshold function.[12] Accordingly, for cycling or running, the number of hours in 2021 exceeding the threshold for moderate, high, and extreme heat stress risk increased globally, compared to 1991, by an average of 186 (40% increase), 93 (41%), and 37 (34%) hours per person, respectively. When separated by HDI country group, in 2021 the number of hours exceeding the threshold for moderate, high, and extreme heat stress risk increased, relative to 1991, by 322 (44% increase), 199 (51%), and 106 (58%) hours per person, respectively, for Low HDI countries; 56 (5% increase), -7 (1% decrease), and -41 (13% decrease) hours per person, respectively, for Medium HDI countries; 132 (56% increase), 63 (83%), and 28 (126%) hours per person, respectively, for High HDI countries; and 114 (111% increase), 69 (151%), and 41 (195%) hours per person, respectively, for Very High HDI countries.

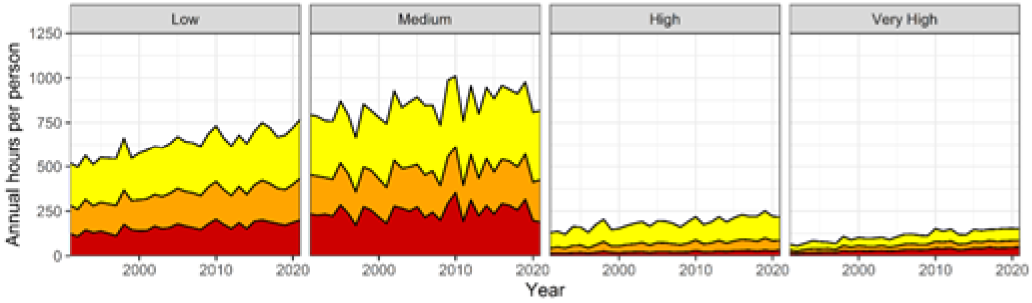

Figure 11: Average annual hours per person that light physical activity entailed at least a moderate, high, or extreme heat stress risk by 2018 country HDI level, 1991-202

Indicator 1.1.4: Change in Labour Capacity

Methods

The methodology for this indicator has been updated and improved from previous reports, by better accounting for the impact of solar radiation on people's capacity to work.

It is based on 68,940 grid cell data (0.5 x 0.5 degrees with boundaries exactly on the degree and half degree co-ordinates) for climate and population. The focus is on trends since the end of the 20th century and on a method that can calculate labour capacity loss at country level. The model data chosen for the calculations was the European Centre for Medium-Range Weather Forecasts (ECMWF) ERA5 reanalysis hourly data on single year levels, and the analysis method is described in detail in the paper by Kjellstrom et al., 2018.[14]

Analysis starts from hourly ambient (t2m) and dew point temperatures (d2m), as well as short wave (solar) radiation downward (ssrd). These inputs are used to derive the hourly heat stress index Wet Bulb Globe Temperature (WBGT) and, from that, the work loss factor (WLF) at three different metabolic rates in both the shade and the sun is calculated. The inclusion of the solar component represents a novelty for the 2022 report.

The full Liljegren formula for calculating WBGT in the sun was used for one year (2010) for all grid cells. This involved also downloading ERA5 surface pressure, surface thermal radiation downwards, total sky direct solar radiation at surface. With this data a good approximation for WBGT *uplift* in the sun was determined from WBGT in the shade. Tested in warm to hot Koppen climate regions, this uplift was 0.0035 * ssrd, which matched the Liljegren WBGT calculation to ±0.2 C. As the Liljegren WBGT calculation[15] also requires air speed, an air movement of 1 m/s was used,the approximate speed at which arms and legs move during work.

For indoor work, exposure was assumed to be atmospheric heat in the shade without effective air conditioning. The impact of heat on labour capacity depends on clothing (assuming light clothing for all) and metabolic rate based on physical work activity. The methodology considers 3 metabolic rates: 200W (light work, sitting or moving around slowly), 300W (medium intensity work) and 400W (heavy labour).

The function relating WLF (the fraction of work hours lost) to an hourly WBGT level is given by the cumulative normal distribution (ERF) function:

$$Loss\ fraction = \frac{1}{2}\left(1 + \mathrm{ERF}\left(\frac{\mathrm{WBGThourly} - \mathrm{WBGTaver}}{\mathrm{WBGT}_{\mathrm{SD}} * \sqrt{2}}\right)\right)$$

where WBGTaver and $\mathrm{WBGT}_{\mathrm{SD}}$ are the parameters (**Error! Reference source not found.**) in the function for a given activity level.

The data were then aggregated to provide estimates of annual WLF between the hours of 6 am - 6 pm local solar time for each grid-cell.

Metabolic rate	WBGTaver	$\mathrm{WBGT}_{\mathrm{SD}}$
200 Watts	35.5	3.9
300 Watts	33.5	3.9
400 Watts	32.5	4.2

Table 1. Input values for labour loss fraction calculation.

For each grid cell, the working age population (15+ years old; as in the ILOSTAT data) for each time period is used as input data as well as the percentages of people in this age range working in 4 sectors: agriculture, construction, manufacturing and "other" sectors, which include the service sector (based on ILOSTAT data). Populations in grid cells that overlap country borders have been apportioned to the countries involved based on population distribution within the cell (variable CountryPop% in the formulas below).

For the work hours lost (WHL), ILO sector proportions are assigned to metabolic rates and sun or indoors/shade calculations applied as shown in **Error! Reference source not found.**:

Metabolic rate:	200W (shade),<br>light work	300W (shade),<br>moderate work	400W (sun),<br>heavy labour
Employment sector:	Other<br>(mainly services)	Manufacturing	Agriculture +<br>Construction

Table 2. Employment sector to metabolic rate assignment

The total annual work hours lost (WHL) for each metabolic rate and country (as well as a global aggregate) are calculated by, first, for each grid cell multiplying each employment sector population by the relevant work loss factor and then, second, summing the resulting sector work hours lost over all grid-cells in each country:

Annual WHL200W (per country) =
Σ(for each country grid-cell): Pop15plus * CountryPop% * Other% * WLF200W

Annual WHL300W (per country) =
Σ(for each country grid-cell): Pop15plus * CountryPop% * Manuf% * WLF300W

Annual WHL400W (per country) =
Σ(for each country grid-cell): Pop15plus * CountryPop% * (Agr% + Constr%) * WLF400W

Then: Total Annual WHL (country) = Annual WHL200W + Annual WHL300W + Annual WHL400W

The annual work hours lost per person (WHLpp) are arrived at by dividing the total annual country WHLs by the total number of employed people in each country for each year. The annual total number of employed people for each country is calculated like:

Annually Employed People (per country) =
(Agr% + Manuf% + Constr% + Other%) * Σ(for each country grid-cell): Pop15plus * CountryPop%

Data

1. Climate data from the European Centre for Medium-Range Weather Forecasts (ECMWF) ERA5 reanalysis.[9]
2. Population data from the NASA Socioeconomic Data and Applications Center (SEDAC) Gridded Population of the World (GPWv4).[2]
3. Sector employment data from ILOSTAT.[16]

Caveats

The distribution of agricultural, construction, manufacturing and other sector workers is only reported at country level, hence this proportion is distributed evenly to all grid cells within each country, and thus does not capture the geographical differences in the proportion of people working in the different sectors.

Calculations for work in the sun (agricultural and construction sectors) assume continuous exposure to solar radiation at all labour times, and do not account for the protective effect of cloud coverage.

Analysis performed with the above-described methodology has shown that the ERA5 data regularly understates temperatures, particularly maximum air temperatures. The ERA5 deviation from the ensemble average of several other data sources varies by location, is generally in the order of 1-4°C lower and is especially pronounced in coastal regions. Combined with often high population concentrations near the coast the WHL results presented here are conservative. As a comparison, when applying the WHL calculations to climate data input sourced from ISIMIP or weather stations, WHL estimates increase by 40%.

Future form of the indicator

Improved methods are currently under development for estimating labour capacity loss from climate and demographic data. Future versions of the indicator may employ these improvements after they have been tested and validated.

Additional analysis

Across the globe, in 2021, 470 billion work hours may have been lost due to heat, equivalent to 83 work hours per employed person (**Error! Reference source not found.**). Twelve countries, the 3 most populous at each HDI (Human Development Index) level, account for > 70% of the total global lost work hours. The 3 countries on the medium HDI level (the 2nd lowest HDI category) rank highest in the world's work hours lost per employee, 2.5-3 times the world average. The 3 biggest countries in the highest HDI category account for the smallest numbers of employment hours lost.

Detailed analysis has also shown that countries with most of their population in the tropical area are worst affected (7 out of 12).

Another aspect of social and health inequity that occupational heat exposure leads to is the difference between

	ISO3 code	Human development level	Latitude	Work hours lost per employed person in 2000	Work hours lost per employed person in 2021	Billions of work hours lost in 2021	% of global
Global				137.4	138.6	470.1	100.0%
Pakistan	PAK	Medium	Sub-trop	373.8	366.7	26.7	5.7%
Bangladesh	BGD	Medium	Trop	377.1	339.3	24.3	5.2%
India	IND	Medium	Trop	340.6	314.3	167.2	35.6%
Democratic Republic of the Congo	COD	Low	Trop	178.9	242.2	8.3	1.8%
Nigeria	NGA	Low	Trop	251.5	241.5	17.5	3.7%
Indonesia	IDN	High	Trop	223.1	201.8	25.7	5.5%
China	CHN	High	Sub-trop	82.3	59.4	44.0	9.4%
Brazil	BRA	High	Trop	75.2	55.4	5.3	1.1%
Ethiopia*	ETH	Low	Trop	33.0	32.9	1.6	0.3%
Japan	JPN	Very High	Sub-trop	21.8	18.6	1.1	0.2%
USA	USA	Very High	Sub-trop	13.3	15.4	2.5	0.5%
Russia	RUS	Very High	Temp	2.7	3.7	0.3	0.1%
Rest of the world				34.5	42.9	145.4	30.9%

** The low impact per employee is linked to the high altitude (with cooler climate) of most of this country*

Table 3. Annual heat-related work hours lost per employed person (agriculture & construction exposed to the sun, all other sectors in shade or indoors) and total WHL in populous countries. Three countries with largest populations in each of the four HDI categories are ranked by WHLpp in 2021.

the impacts on the working class that works at high physical intensity and the white-collar employees with less straining work. The impact on labour capacity increases rapidly with the physical intensity of the work. Another aspect of social and health inequity that occupational heat exposure leads to is the difference between the impacts on the people in labouring jobs that require high physical intensity and those in office or service jobs with less straining work. The impact on labour capacity increases significantly with the physical intensity of the work.

Agricultural workers are the worst affected in many countries (**Error! Reference source not found.**), with the burden often shifting to those in construction in higher income countries, such as the USA. The trend for heat-related employment hours lost is rising in the listed countries, except for China and Brazil, where there has been a major reduction in the agricultural workforce mainly in favour of the service industry.

The global distribution of work hours lost (WHL) in the four workforce sectors is shown in **Error! Reference source not found.**. Agriculture dominates but stays largely constant due to reductions of the agricultural workforce in many low- and middle-income countries. The impact of rising heat is increasing the fastest in construction and *other* sectors (mainly in the service industry).

Because of its definition this indicator is influenced by the changes in population numbers and the distribution of the workforce within countries as well as climate change. WLF (work loss factor) is defined as the fraction of work hours lost for one worker at a specific metabolic rate, and thus describes work capacity loss due to heat independently from population and employment statistics. Figure 13 shows global WLF trends attributable to climate alone. In addition, this chart includes loss factor trends when an agricultural worker (400W metabolic rate) is exposed to the sun. The WLF rate doubling when solar radiation is included is an illustration of how disproportionate an increase by a few degrees of WBGT (solar *uplift* outlined above, typically between 1 and 2.5 degrees) affects human work capacity.

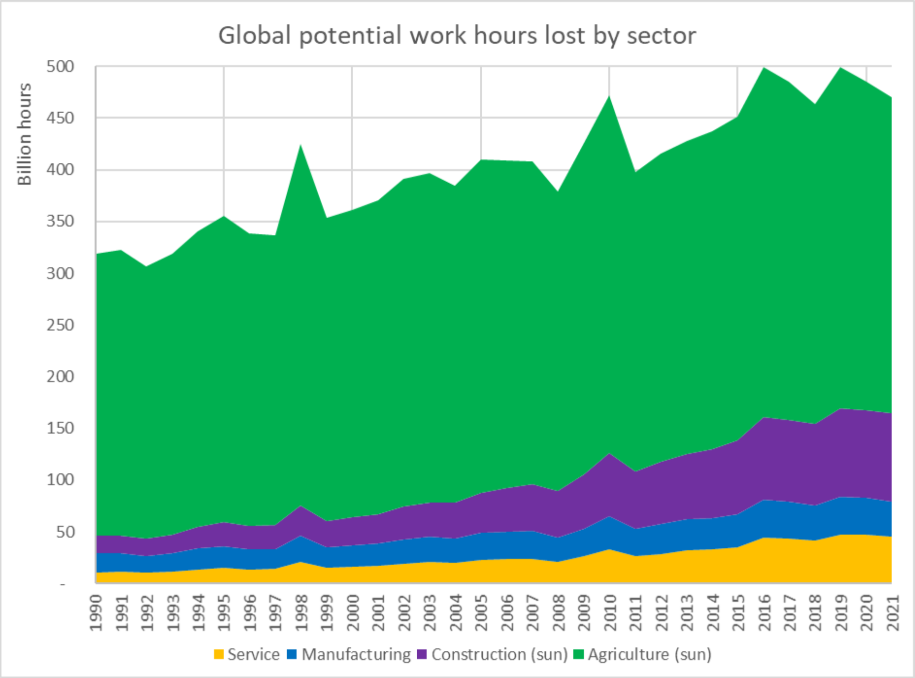

Figure 12. Global potential work hours lost (billions) due to heat by employment sector, 1990-2021.

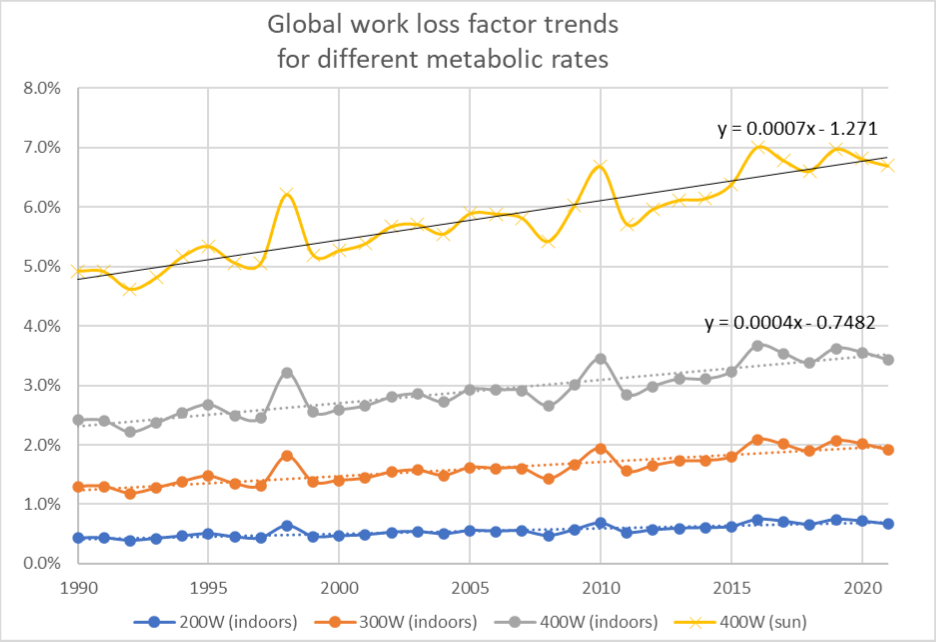

Figure 13. Work hours lost (% of annual hours) depending on physical work intensity, global means, 1990-2021.

Indicator 1.1.5: Heat-Related Mortality

Methods

The methodology for this indicator, which tracks the global total number and spatial pattern of heat-related mortality from 2000 to 2021, remains similar to that described in the 2021 report of the *Lancet* Countdown.[11]

The heat-related excess mortality in one day E is expressed as

$$E \;=\; y_0 \times Pop \times AF \qquad (1)$$

where y_0 is the non-injury mortality rate on that day, *Pop* is the population size and *AF* is the attributable fraction on that day. Because every day's mortality rate is hard to obtain, y_0 is computed as the yearly non-injury mortality rate from the Global Burden of Disease data, divided by 365.

AF is calculated via the relative risk (*RR*) which represents the increase in the risk of mortality resulting from the temperature increase. *RR* is regressed as $RR = exp^{\beta(t-OT)}$, so *AF* is calculated as

$$AF = \frac{RR-1}{RR} = 1 - exp^{-\beta(t-OT)} \quad (2)$$

where *t* is the daily maximum temperature, β is the exposure-response factor and OT is optimum temperature, and both parameters were adopted from Honda et al. (2014).[17] The method was applied to gridded daily temperature data from ECMWF ERA5 dataset, and gridded population data from NASA GPWv4 population dataset and ISIMIP Histsoc records, as with Indicator 1.1.1. As the indicator focuses on a population that is 65 years old or older, age-structure data from United Nation World Population Prospects was also used.

Following WHO definitions, years of life lost (YLL) is calculated as:

$$YLL = \sum_{m=65-69}^{100+} E_m \times LE_m \qquad (3)$$

where YLL is the annual YLL of a certain grid cell, E_m is annual heat-related excess mortality in age group m of the grid, and LE_m represents the standard life expectancy at the age of death in years of age group m. Life

expectancy data were obtained from the Global Burden of Disease Study 2019 (GBD 2019) Results by Institute for Health Metrics and Evaluation (IHME), same with the mortality rate data.[18] Because the mortality rate and life expectancy data of 2020 and 2021 has not yet been released, and the real data were highly affected by Covid-19, which would affect the accuracy of the results, so 2019 data were used instead.

The heat-related mortality and YLL was first calculated at grid level at 0.5° spatial resolution. Then it was accumulated to global level to produce a time-series analysis.

Data

1. Climate data from the European Centre for Medium-Range Weather Forecasts (ECMWF) ERA5 reanalysis.[9]
2. Population data from the NASA Socioeconomic Data and Applications Center (SEDAC) Gridded Population of the World (GPWv4) and The Inter-Sectoral Impact Model Intercomparison Project (ISIMIP) Histsoc dataset.[2,3]
3. Demographic data from the United Nation World Population Prospects (UN WPP).[19]
4. Mortality rate and life expectancy data are from the Global Burden of Disease.[20]

Caveats

This indicator applies a unique exposure-response function across all locations and times. While its use has been demonstrated in different geographies, it does not capture local differences in the health impacts from heat exposure, which can be significant. Also, this analysis assumes exposure-response function is constant. It does not capture changes in response to heat exposure that might happen over time, as a result of acclimation and adaptation. Not capturing these changes could result in an over-estimation of heat-related deaths in later calendar years. Annual average mortality rates are used, rather than daily mortality rates (y_0). Given baseline mortality can be higher in colder months, this may lead to an overestimation of overall mortalities. Nonetheless, the trends of change in mortality due to heat exposure should still be conserved.

Only the heat-related mortality of the 65-and-older population was calculated this time, but more work needs to be done to include working group people.

Additional analysis

Heat-related deaths of people older than 65 years in each country in 2021 is shown in Figure 14. The change in global heat-related mortality and years of life lost is presented in Figure 15. Except for the Eastern Mediterranean, all WHO regions decreased in heat-related deaths in 2021 compared to 2019 (**Table 4**). The largest decrease was in the South-East Asian region, followed by the Western Pacific region. Europe, however, remains the region with the highest number of heat-related deaths.

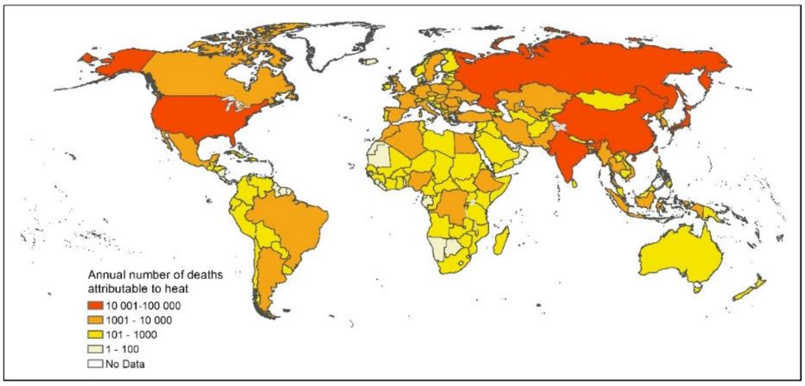

Figure 14. Heat-related deaths of people older than 65 years in each country in 2021.

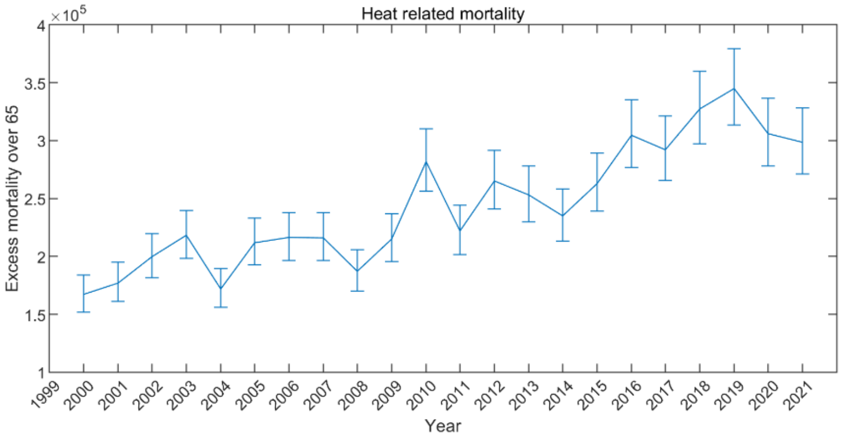

Figure 15. Global heat-related mortality in the 65-and-older population, 2000–2020. The error bars were calculated on the basis of the 95% CIs of the exposure-response function described by Honda et al. (2014)[17]

WHO Region	2019	2020	2021	Change in mortality (2020 to 2019)	Change in mortality (2021 to 2019)
African	16,898	15,953	15,929	-946	-969
Americas	44,246	48,085	39,181	3,839	-5,066
Eastern Mediterranean	15,597	15,298	17,351	-299	1,754
European	107,614	103,695	103,493	-3,917	-4,120
South-East Asian	63,428	34,421	31,321	-29,006	-32,107
Western Pacific	97,101	88,441	91,178	-8,659	-5,922

Table 4. Change of heat-related mortality for the 65-and-older population between 2020 and 2019 for different WHO regions.

For the different HDI (Human Development Index) regions, only the low HDI country group showed a small increase in heat-related mortality in 2021 compared to 2019, with all other groups decreasing to some extent (**Table 5**). The largest decrease was in the medium HDI country group, followed by the high HDI group, while the very high HDI group did not show a significant decrease and maintained the largest number of heat-related mortality.

HDI level	2019	2020	2021	Change in mortality (2020 to 2019)	Change in mortality (2021 to 2019)
Very High	138,772	138,433	132,729	-339	-6,043
High	127,463	115,213	115,377	-12,250	-12,086
Medium	64,848	38,261	36,326	-26,586	-28,521
Low	12,231	12,782	12,550	552	319

Table 5. Change of heat-related mortality between 2020 and 2019 for different HDI levels.

1.2: Health and Extreme Weather Events

Detection and attribution (D&A) studies are increasingly enabling the quantification of climate change influences on single extreme weather events. **Table 6** builds upon the analysis done in the 2020 edition of the *Lancet* Countdown, that similarly classifies detection and attribution studies published between 2015–2020, which correspond primarily to events ending in 2015–2018. Since this time period, there has been a relative increase in the number of events for which D&A studies have been conducted. This analysis includes the 24 D&A studies for discrete events occurring between 2019–2021, categorised as extreme heat events; heavy precipitation events and flooding; wildfires; storms; tornadoes and cyclones; or drought and low precipitation events. Disaggregated by event type, there has been a relative increase in D&A studies conducted on heavy precipitation and flood events and wildfires, and a relative decrease in D&A studies conducted on extreme heat events, storms, tornadoes and cyclones, and drought and low precipitation events.

**Event**	**Date**	**Impact of climate change on likelihood or severity**	**Direct fatalities**
Heatwaves			
European heatwaves	June - July 2019	Increased[21-24]	2500[25]
Hot drought in Yunnan, China	March - June 2019	Increased[24]	No deaths or data not available
North Pacific marine heatwaves	2019	Influence uncertain[24]	No deaths or data not available
Warm wet winter in northwest Russia	2019-2020	Increased[26]	No deaths or data not available
High temperatures in western Europe	May 2020	Increased[27]	1921 (France)[28], 2,556 (UK)[29], 1114 (Portugal)[30]
Siberian heatwave	April-Sept 2020	Increased[31]	No deaths or data not available
Hot wet extremes in South Korea	June-August 2020	Increased[32]	46[32]

Western North American 'heat dome'	June 2021	Increased[33]	669[34,35]
Heavy precipitation and flood events			
Extended rainy winter over the Yangtze River	2018-2019	Decreased[24]	No deaths or data not available
Extreme annual streamflow into Chesapeake Bay from Susquehanna River	2019	Increased[24]	No deaths or data not available
Wet rainy season in Southern China	March-July 2019	Decreased[24]	88[36]
Ottawa River flood	May-June 2019	Increased[24]	1[37]
Warm wet winter in Northwest Russia	2019-2020	Increased[26]	No deaths or data not available
Hot wet extremes in South Korea	June-August 2020	Increased[32]	46
Low North American Monsoon Rainfall	June–September 2020	Increased[38]	No deaths or data not available
High precipitation event over Beijing	February 2020	Increased[39]	No deaths or data not available
Floods in central and southern China	June - July 2020	Decreased[40,41]	219[42]
Flooding in Western Europe	July 2021	Increased[43]	220[43]
Wildfires			
Alaskan fires	July 2019	Increased[24]	0
Wildfire in southwest China	March to May 2019	Increased	31[44]
Australian 'black summer' bushfires	2019-2020	Increased[23,45]	417[46]
Siberian fires	April-Sept 2020	Increased[47]	0
Storms, tornadoes and cyclones			
Hurricane Dorian (the Bahamas)		Increased[24]	319[48]
Typhoons in South Korea	2019	No influence identified[32]	8 (Typhoon Lingling)[49]6, 10 (Typhoon Mitag)[36], 3 (Typhoon Tapah)[50]

Drought and low precipitation events			
Prolonged Drought Western Cape of South Africa	2015-2019	Increased[24,51,52]	No deaths or data not available
Extreme Low Precipitation<br>in Southwestern China	April-June 2019	Increased[24]	No deaths or data not available

Table 6: Events occurring 2019–2021, for which detection and attribution studies published

Indicator 1.2.1: Wildfires

Methods

This indicator has been updated and improved from the 2021 report of the *Lancet* Countdown, better accounting for cloud coverage in the remote sensing of wildfire, and also tracking concentrations of wildfire smoke concentration in terms of fine particulate matter ($PM_{2.5}$).

Wildfire

The change in population exposure to wildfire is represented as the change in the average annual number of person-days exposed to wildfire in each country (Figure 16). Satellite-observed active fire spots were aggregated and spatially joined with gridded global population data from the NASA SEDAC GPW v4.11 dataset on a global 0.1o x 0.1o resolution grid. Grid cells with a population density ≥ 400 persons/km2 were excluded to remove urban heat sources unrelated to wildfires. New to 2022, cloud cover information was incorporated to each grid cell of the satellite-observed active fire data to address the issue of fire spot underestimation due to cloud obscuration.[53] The mean annual number of person-days exposed to wildfire during the most recent four years (2018–2021) was compared with the baseline period of 2003–2006 (Figure 16).

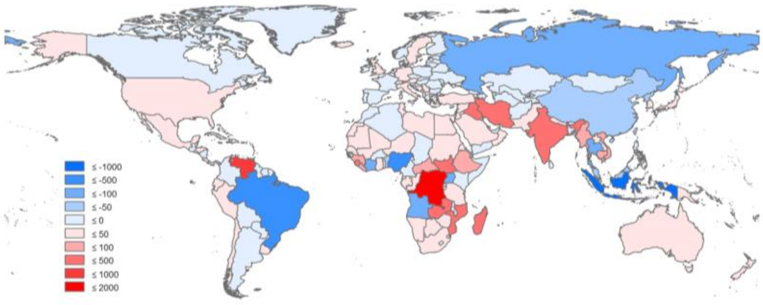

Figure 16: The annual mean number of person-days exposed to wildfire by country in 2018-2021 compared with 2003-2006 (unit: 10,000 persons). Large urban areas with population density ≥ 400 persons/km^2 are excluded.

The fire danger is represented in terms of the Fire Danger Index (FDI). Provided by ECMWF ERA5 atmospheric reanalysis, FDI is a numeric rating with values 1-6 representing very low, low, medium, high, very high and extreme fire danger, respectively. Daily FDI data, available from 3rd January 1979 through 31st December 2021 worldwide, were aggregated so as to obtain the yearly number of days of each fire danger level at every 0.25° x 0.25° grid cell. The changes in mean number of days exposed to very high or extremely high fire danger (defined as FDI ≥ 5) were collected for the 2018-2021 period and compared with the 2001-2004 baseline. Gridded population data[8] re-gridded to the coordinates of the fire danger data (0.25°x0.25°) and linearly interpolated for each year from 2001 through 2020 were used. As for the year of 2021, 2020 population data were considered for consistency. Population data were used to calculate population weighted mean days of fire risk and exclude pixels with population density higher than 400 persons/km^2 in the calculation of population-weighted means. The same exclusion criteria were also applied in the calculation of area-weighted means.

To provide country-averaged data, both the change in population exposure to wildfire and the fire danger were spatially matched with the official country borders used by the World Health Organization. Each country was then linked to United Nations' human development index (HDI).

Wildfire smoke

The frequency and intensity of wildfires is increasing in many regions all over the world, which has been partially attributed to human-caused climate change.[54] However, up to 90% of wildfires are started by humans – either accidentally during, e.g., recreational activity, or deliberately as part of industrial, forest management and exploitation, or agriculture practices.[55] Wildfire smoke is estimated to account for 25% of $PM_{2.5}$ in the United States in recent years.[56] In Europe, estimates suggest the burned area under no adaptation would increase by 200% during this century.[57] Wildfires emit a range of health damaging pollutants including carbon monoxide, polycyclic aromatic hydrocarbons, primary aerosols and precursors to secondary aerosols.[58] Epidemiological studies provide evidence that wildfire smoke is significantly associated with increased risk of mortality and morbidity, particularly respiratory morbidity, which justifies the selection of this parameter.[59,60]

New to 2022, the indicator tracks personal exposure to fire-originated fine particles ($PM_{2.5}$) at the global scale during the last 19 full years, 2003-2021, which correspond to the complete period available from MODIS instruments onboard Aqua and Terra satellites. The information is provided in a gridded form with resolution of 0.5°. The exposure to fire-originated fine particles is computed by the Integrated System for vegetation fires IS4FIRES[61-64] used as a fire information system, and the System for Integrated modeLling of Atmospheric coMposition SILAM[65,66] for atmospheric composition modelling. The procedure has been used previously to estimate the attributable health burden of wildfire smoke episodes in Europe.[66] The indicator generation includes several steps:

1. Data acquisition. The level-2 data of MODIS Fire Radiative Power (FRP) observations MOD14 / MYD14 are downloaded from the NASA web service
2. IS4FIRES. The granules of FRP level-2 data are screened, aggregated, and processed to hourly FRP sets with a predefined land-use-specific diurnal variation
3. IS4FIRES → SILAM. The FRP files are converted to fine-PM emission fluxes using empirical coefficients identified in earlier calibration studies and the global classification of land use from the ECOCLIMAP dataset.[61,64,67] The model also computes plume injection profile following the original methodology[62,63]
4. SILAM. The smoke (fire-induced $PM_{2.5}$) dispersion is simulated with the SILAM model with input from IS4FIRES. The latest operational versions of both systems are used throughout the run to obtain a homogeneous dataset suitable for trend analysis. The spatial resolution of the product is 0.5º (~50 km at the equator) and the temporal resolution is one hour
5. Post-processing. The SILAM output concentration is integrated over each year and the linear trend of concentrations, and its statistical significance, are computed for each grid cell
6. Spatial aggregation. The gridded data are aggregated to the country level and both mean exposure and its trend are computed for each country

Data

1. MODIS Fire Radiative Power (FRP) observations MOD14/MYD14 from the NASA Fire Information for Resource Management System (FIRMS)[68]
2. Cloud cover data from the EarthEnv Global 1-km Cloud dataset[69]
3. Fire danger indices historical data produced by the Copernicus Emergency Management Service for the European Forest Fire Information System (EFFIS)[70]
4. Global classification of land use from the ECOCLIMAP dataset[71]
5. Population data from the NASA Socioeconomic Data and Applications Center (SEDAC) Gridded Population of the World (GPWv4) and from the Hybrid gridded demographic data for the world, 1950-2020 (1.0)[2,8]

Caveats

The fire danger index represents a potential fire risk calculated by meteorological parameters. It does not represent actual fire events. The actual fire events can be also influenced by anthropogenic factors, such as human-induced land use and land cover changes, industrial-scale fire suppression, and human induced ignition. The fire danger index does not account for the potential fertiliser effect of CO_2 and the associated changes in vegetation and thus

the fuel load of fire. Further, it does not consider potential changes in lightning ignitions, which can be affected by climate change, but the effect is highly uncertain.

To-date, MODIS active fire counts and fire radiative energy products are arguably the best source of fire information worldwide. However, as every low-orbit satellite, MODIS suffers from the omission errors. These have two causes: (i) cloud obscuration, including the fire obscuration by own smoke plumes, (ii) limited sensitivity of the instrument causing omission of small fires.[72] The omission error varies widely depending on region and season being close to ~20–30% for European regions during the local fire seasons but reaching 70% in some equatorial areas. For clear-sky retrievals, the detection limit depends on the viewing angle and time of the day. At night, sub-satellite fires of just 4 MW radiative power are detectable, whereas during day at the edge of the viewing area, burns up to 40 MW are non-detectable. The second caveat is the MODIS commission error: misinterpretation of high-temperature non-fire sources as fires: volcanoes, power plants, large factories, gas flares, etc. Most of these sources have been mapped and removed from the dataset using an original persistent-hot-spots mask.[64] However, some of these hot spots pass through this filter due to their intermittent character. In particular, gas flares are active only a fraction of time and cannot be considered as persistent. The effect is well visible in the Middle East and Persian Gulf region, where a vast majority of the hot spots are the gas flares.

Future form of the indicator

Active fire spots as obtained from MODIS represent raw fire information and do not differentiate between wildfire and prescribed burns. Furthermore, the spatial resolution of the indicator is 0.1-degree which may underestimate wildfire exposure and introduce a bias. To correct this, future improvements could consider increasing the indicator's resolution to 0.01-degree using global 1-km datasets. As this is computational- and time-consuming, these improvements will be introduced in the 2023 report.

As for the exposure to fire-originated fine particles, there are ways to partly rectify both the omission and commission errors of MODIS. For the omission error reduction, one can use more satellites, e.g., VIIRS and SLSTR, producing similar products but providing the data at different overpass times. Secondly, utilisation of geostationary instruments, such as SEVIRI for Africa and Southern Europe, HIMAWARI for Asia, GOES East and West for the Americas. Thirdly, the emerging technology of fire data assimilation and fusion can allow for breakthrough improvements, essentially merging the fire models and (incomplete) satellite observations. None of these methods is problem-free but their combination can strongly reduce the omission error and provide high-quality fire data. The commission problem can also be addressed by involving high-resolution satellites, such as VIIRS. It allows a more specific land-use attribution and differentiation between the vegetated and built-up or industrial areas. However, VIIRS itself has a commission problem, which has to be handled simultaneously. Furthermore, this part of the indicator will be developed towards various forms of presentation and aggregation: in addition to the gridded and country-level forms, it will be aggregated with regard to HDI and other relevant indices jointly identified in discussion with the stakeholders and users of the information.

Additional analysis

Figure 17 presents the land area-weighted mean changes in extremely high and very high fire danger days in 2018–2021 relative to 2001–2004 and excludes pixels with population density higher than 400 persons/km^2. This figure indicates an increasing “climate-related hazard” trend in most countries over the last two decades, with the most prominent increases occurring in central Asia, Southern Hemisphere Africa, and Australia.

The population-weighted and land area-weighted mean changes in extremely high and very high fire danger days in 2018–2021 relative to 2001–2004 for each HDI category or each WHO region are shown in Table 7 and Figure 18. Lower HDI countries appear to have a larger growth in climatological danger of wildfire, in terms of both "human exposure to hazard" and “climate-related hazard”. African and European WHO regions appear to have the largest growth in climatological danger of wildfire, while the South-East Asian region has experienced a decrease in wildfire danger risk over the last two decades.

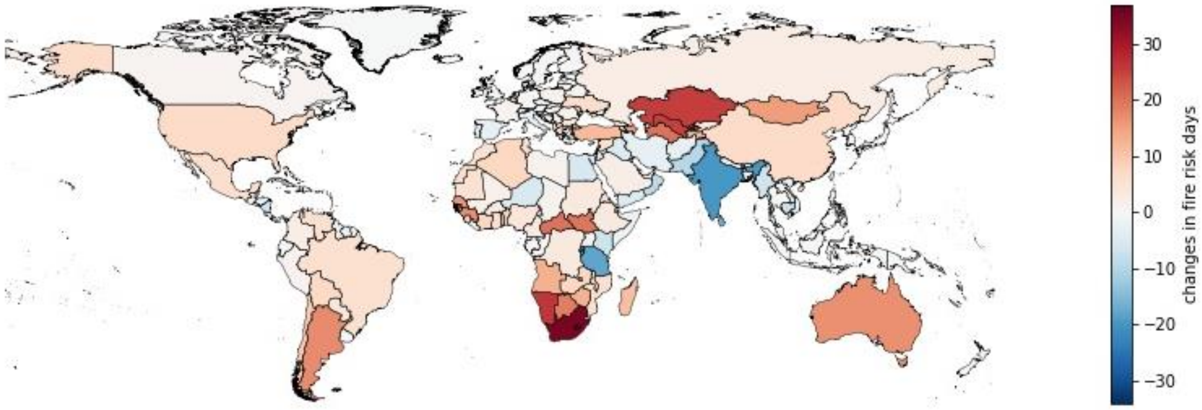

Figure 17. Land area-weighted mean changes in extremely high and very high fire danger days in 2018–2021 compared with 2001–2004. Large urban areas with population density ≥ 400 persons/km^2 are excluded.

HDI level	Population-weighted mean changes		Area-weighted mean changes	
	Mean Change in Fire Danger Days	Number (%) of Countries with Increased Fire Danger Days	Mean Change in Fire Danger Days	Number (%) of Countries with Increased Fire Danger Days
Low	7.11	26 (79%)	5.06	26 (79%)
Medium	3.99	20 (54%)	2.37	19 (51%)
High	3.62	27 (51%)	3.54	29 (55%)
Very High	1.88	36 (55%)	1.29	35 (54%)
WHO region	Population-weighted mean changes		Area-weighted mean changes	
	Mean Change in Fire Danger Days	Number (%) of Countries with Increased Fire Danger Days	Mean Change in Fire Danger Days	Number (%) of Countries with Increased Fire Danger Days
Africa	8.81	36 (77%)	6.69	37 (79%)
Americas	1.61	18 (51%)	2.16	18 (51%)
Eastern Mediterranean	0.39	11 (50%)	-1.53	9 (41%)
Europe	4.19	37 (74%)	3.07	37 (74%)
South-East Asia	-3.02	2 (18%)	-3.30	2 (18%)
Western Pacific	0.68	6 (30%)	1.45	5 (25%)

Table 7. Population-weighted and land area-weighted mean changes in extremely high and very high fire danger days in 2018–2021 compared with 2001–2004 by HDI level and WHO region. The number and percentage of countries with increased exposure by HDI level and WHO region are calculated. Large urban areas with population density ≥ 400 persons/km^2 are excluded.

(a)

(b)

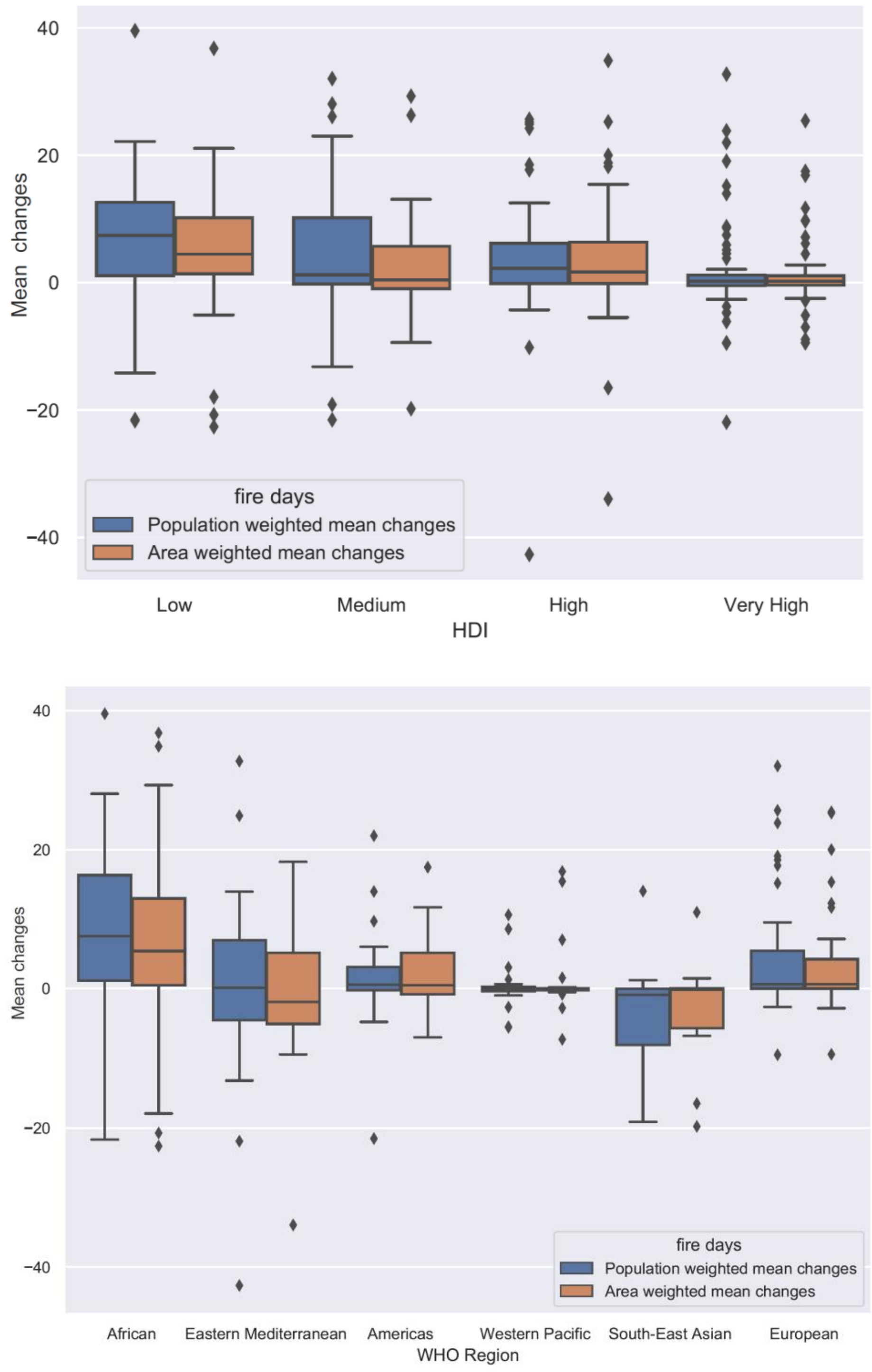

Figure 18. Population-weighted and land area-weighted mean changes in extremely high and very high fire danger days in 2018-2021 compared with 2001–2004 by (a) HDI level and (b) WHO region.

The change in the annual mean number of person-days exposed to wildfire by HDI level and by WHO region in 2018-2021 compared with 2001-2004 are shown in Table 8. After correcting for global cover, low HDI countries appear to have the largest increase of wildfire exposure (+1.34 million persons) and very high HDI countries appear to have a decrease (-0.04 million persons) of wildfire exposure. In addition, 78% medium HDI countries and 73% high HDI countries experienced an increase in wildfire exposure. As for the WHO regions, after correcting for global cloud cover, the African appears to have the largest increase of wildfire exposure (+1.1 million persons) and European appear to have a decrease in wildfire exposure (-0.06 million persons). All the

other WHO regions experienced an increase in wildfire exposure. Note that 91% Eastern Mediterranean countries had an increased exposure.

HDI Level	Change of Exposure	Number of Countries with Increased Exposure	Percentage of Countries with Increased Exposure
Low	134.9	27	84%
Medium	56.0	28	78%
High	6.0	38	73%
Very High	-3.5	37	58%
WHO Region	Change of Exposure	Number of Countries with Increased Exposure	Percentage of Countries with Increased Exposure
African	111.1956	37	82.22
Western Pacific	28.1637	18	75
Americas	10.7151	26	74.29
South-East Asian	33.6232	6	54.55
Eastern Mediterranean	12.0619	20	90.91
European	-5.9951	25	49.02

Table 8. The annual mean number of person-days exposed to wildfire by HDI level and by WHO region in 2018-2021 compared with 2001–2004 (unit: 10,000 persons). The number and percentage of countries with increased exposure are calculated. Large urban areas with population density ≥ 400 persons/km^2 are excluded.

As far as the population exposure to fire-originated fine particles is concerned, this depends, apart from the concentrations themselves, on population distribution and absolute number of people affected by the plumes. Therefore, three quantities are considered: the personal exposure represented by mean concentrations (Figure 20), the population-weighted personal exposure, and the total-population exposure. The personal exposure depends only on fire smoke distribution. The population-weighted exposure additionally depends on the population distribution, and the total population exposure depends on the actual number of people exposed to smoke. Figure 21 shows that, as a global mean, the individual exposure (mean smoke concentrations) has no statistically significant trend: the rising fire intensity in some parts of the world is compensated by its reduction on other regions (Figure 20). The population-weighted exposure, however, shows a noticeable upward trend reflecting a growing fraction of the world population living in a proximity to fire-prone areas. Finally, a strong upward trend of the total-population exposure (Figure 22) is driven by the growing world population.

An interplay of fire danger and actually observed fires can be seen from comparing the exposure to fire smoke with a Canadian Fire Weather Index FWI.[73] FWI is one of the most-widely used fire danger indices.[74] As Figure 23 shows, in most of fire-prone areas the FWI trends are quite well pronounced and often statistically significant but may differ from those of the fire smoke exposure. One of such areas is in Europe: the FWI is predominantly upwards, albeit not always statistically significant (Figure 23), whereas the fire-related exposure has a near-neural or downward trend (Figure 20). One of plausible explanations is a series of measures undertaken in Europe to control the fires, especially in the vulnerable regions of Southern Europe.[57,75]

Analysis of the exposure map aggregated to a country level and its trend (Figure 24) confirmed the above observations and highlighted the asymmetry of the up- and down-wards trends: the upward trends of exposure are, in most cases, strong and statistically significant, except for a few African countries and Mexico. Conversely, the downward exposure trends, formally covering much larger territories, are mostly small and not significant from a statistical standpoint. Exceptions are only Amazonia, Argentina, equatorial Africa, and Kazakhstan, each region having their own reasons for the reduction, mostly not related to climate change. Indeed, as seen from the FWI in Figure 23, there is little correlation between the regions with lowering the weather-driven fire danger and the exposure computed from the actually registered fires.

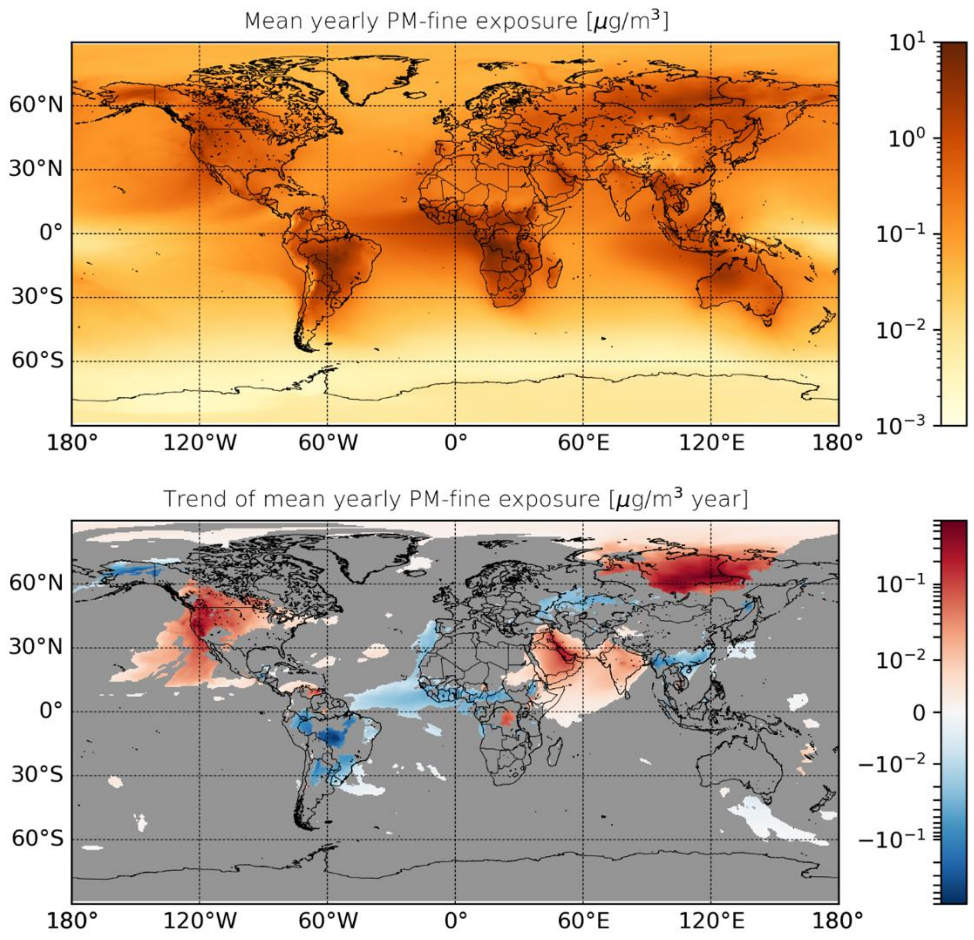

Figure 19: Gridded mean personal exposure to fire-induced PM (upper panel) and its trends (lower panel), 2003 – 2021. Only statistically significant trends (p<0.1) are shown

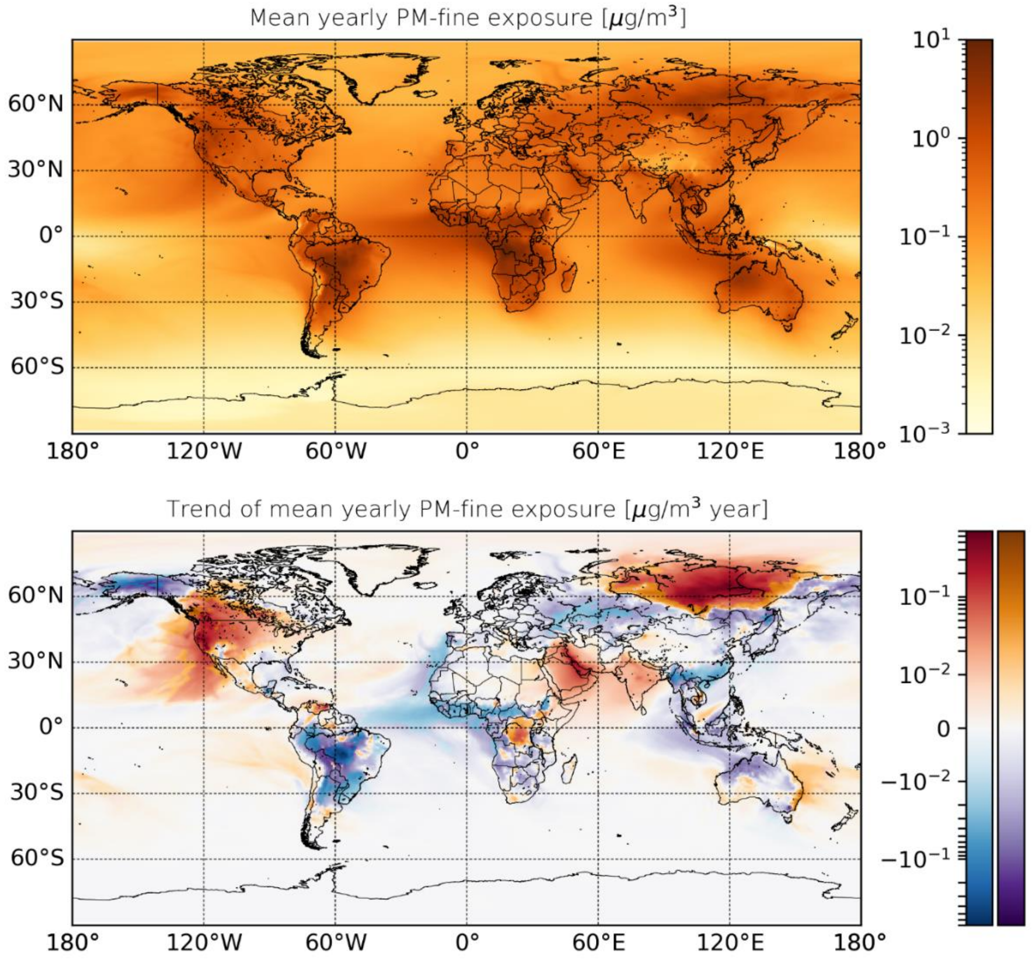

Figure 20. Fire smoke mean exposure in 2003–2021 (upper panel) and its trend (lower panel). The blue-red colour represents statistically significant trend, p < 0.1, whereas violet-brown colours represent the not statistically significant trend, p≥ 0.1

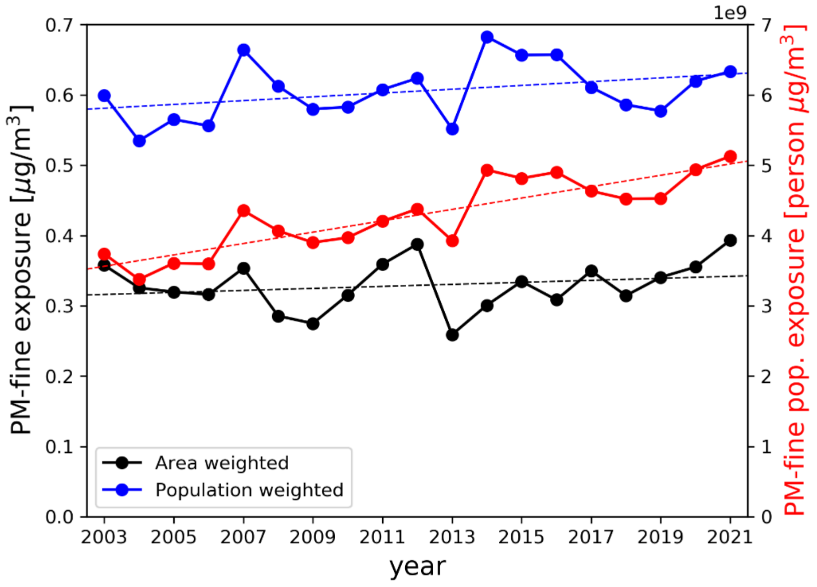

Figure 21. Trends of personal exposure (black line, left-hand vertical axis), population-weighted personal exposure (blue line, left-hand axis), and total population exposure (red line, right-hand vertical axis).

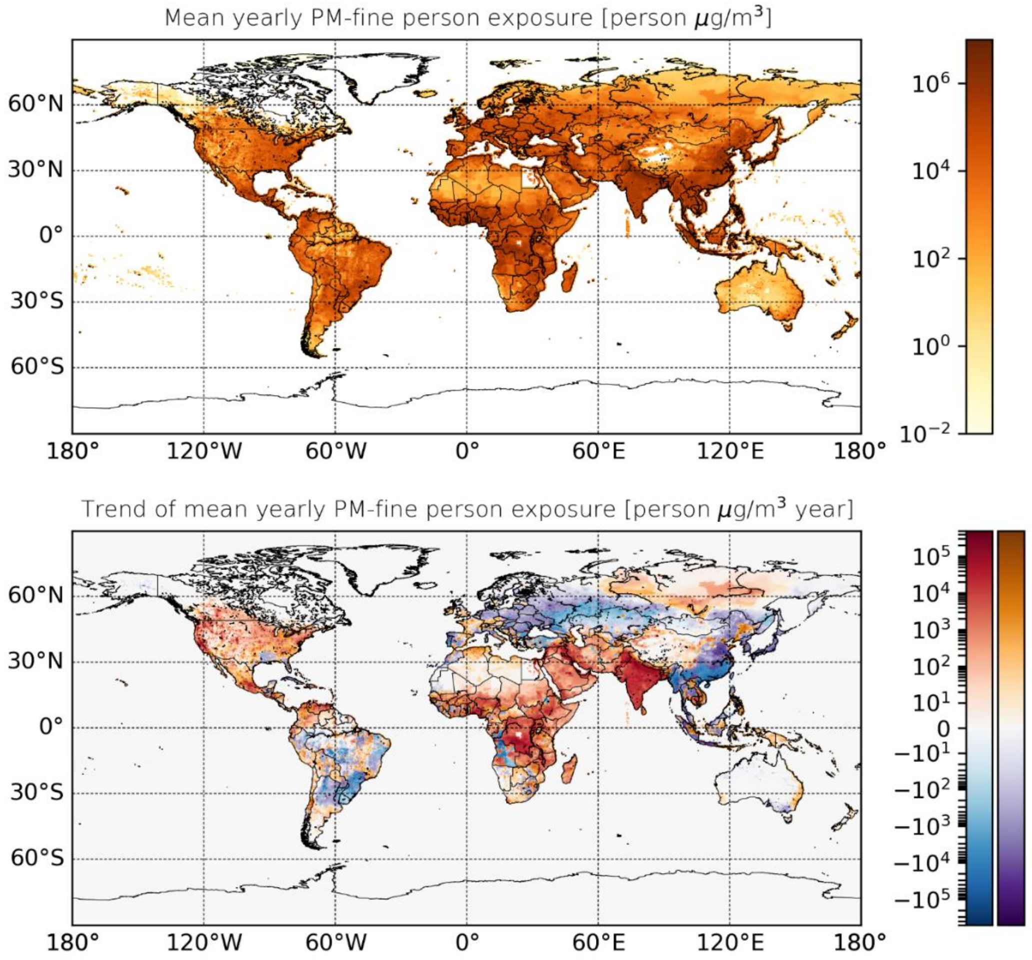

Figure 22. Absolute level and trend of total-population exposure. For trends, the blue-red colours represent statistically significant trends ($p<0.1$), whereas the violet-brown colours show not significant ones ($p>0.1$).

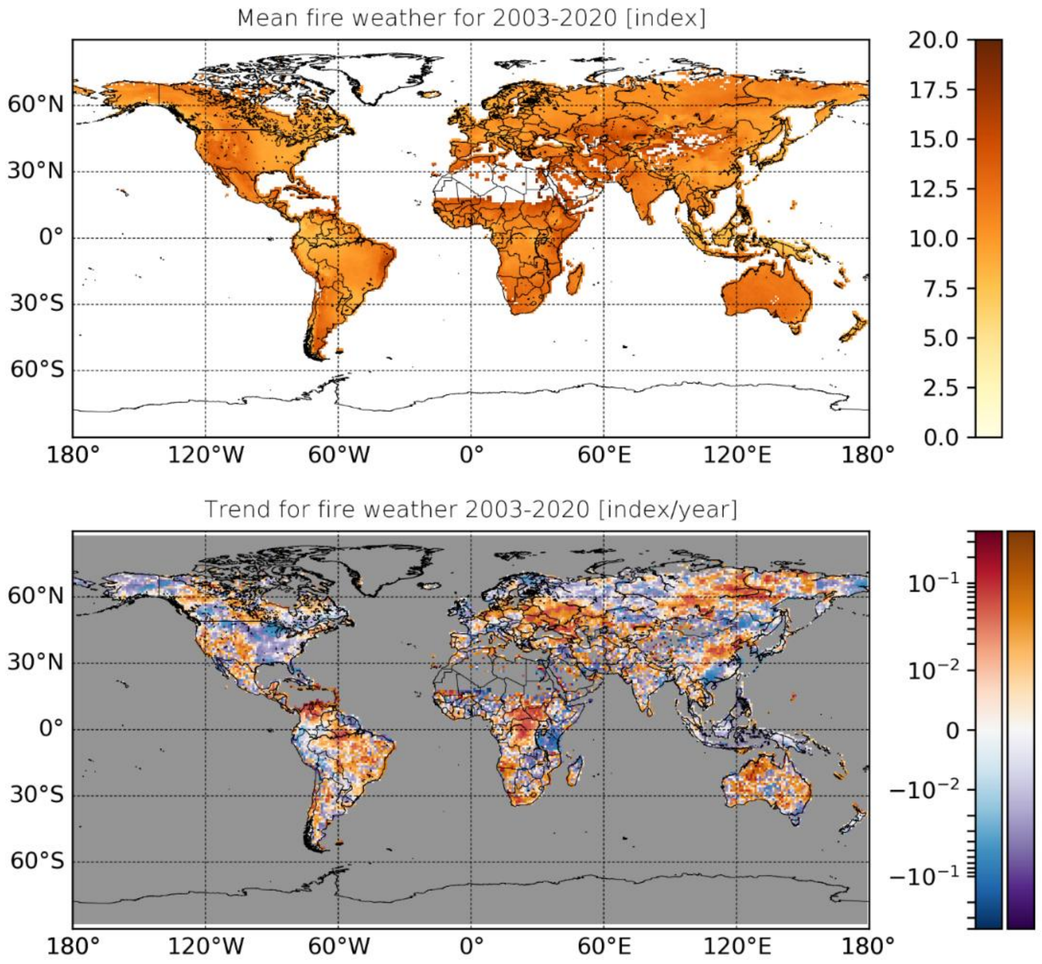

Figure 23. Mean absolute level and trend of the Canadian Fire Weather index. For trends, the blue-red colours represent statistically significant trends (p<0.1), whereas the violet-brown colours show not significant ones (p>0.1).

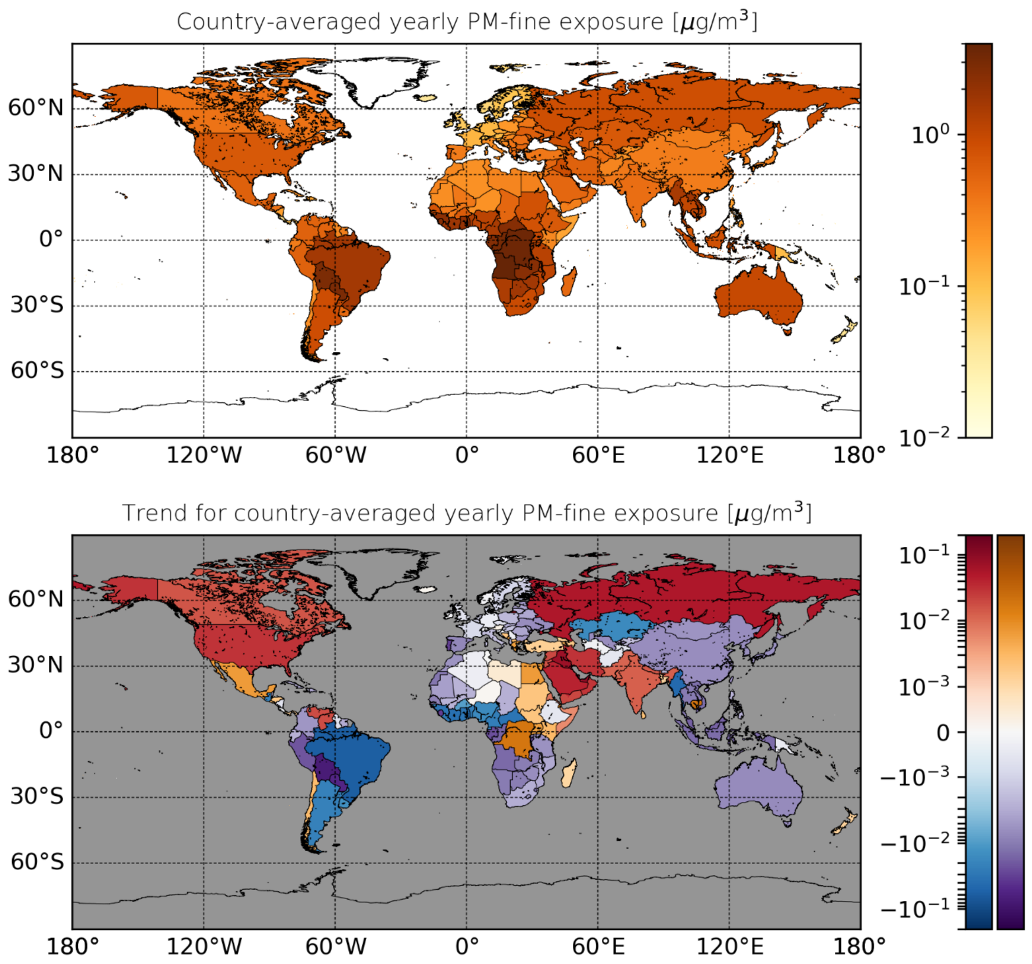

Figure 24. Absolute level and trend of personal fine-PM exposure aggregated to country-level. For trends, the blue-red colours represent statistically significant trends (p<0.1), whereas the violet-brown colours show not significant ones (p>0.1).

Indicator 1.2.2: Drought

Methods

The data source for this indicator has been updated for the 2022 report.

The drought indicator was improved in the 2021 report to include the 6-monthly Standard Precipitation Evapotranspiration Index (SPEI6)[76] as a measure of the land surface affected by drought events. This index allows for both the intensity and the duration of droughts to be taken into account. It captures the influence of both altered precipitation patterns, and of potential evapotranspiration on drought severity.

SPEI6 data are obtained from the SPEI Global Drought monitor. The Global Drought monitor uses mean temperature data from the NOAA NCEP CPC GHCN-CAMS gridded dataset[77] and monthly precipitation data from the 'first guess' Global Precipitation Climatology Centre (GPCC)[78]. GCPC data, which have an original spatial resolution of 0.5º x 0.5°, are interpolated to the resolution of 1° x 1°. Potential evapotranspiration is calculated using the Thornthwaite equation.

The SPEI Global Drought Monitor calculates SPEI values using constantly updated climate data at a global scale with a 1° x 1° spatial resolution and a monthly time resolution. SPEI time scales between 1 and 48 months are provided. For the indicator the 6-monthly SPEI value is used (SPEI6) and the calibration period is set to January 1950 to December 2010. SPEI6 data for 1950-present were downloaded from the Global SPEI Database.[79]

Droughts were defined according to three severity levels using the SPEI thresholds indicated in Table 9, as defined by the Federal Office of Meteorology and Climatology MeteoSwiss.[80] In order to detect excess (unusual) drought events, "excess severe drought events" were defined as yearly counts of months in drought for each grid cell which exceed 2 standard deviations above the mean of the yearly counts of months in drought for the baseline period of 1986-2005. The excess events were defined for each SPEI severity level of drought independently, and the percentage of land area exposed to excess drought events at the different severity levels was calculated.

SPEI value	Description	Frequency of event in respective month
< -1.3	severe drought	1-2 x in 20 years (i.e. 10% if the time)
< -1.6	extreme drought	1-2 x in 40 years (i.e. 5% of the time)
< - 2	exceptional drought	1 x in 50 years or less (i.e. ≤2% of the time)

Table 9. Summary of drought severity thresholds as defined by the Federal Office of Meteorology and Climatology MeteoSwiss.

Data

1. SPEI6 data from the Global SPEI Database, SPEIbase (Consejo Superior de Investigaciones Cientificas).[79]

Caveats

A limitation of this indicator is that it only captures the impacts of climate change on meteorological drought but does not capture the impacts of climate change on hydrological or agricultural drought, which can have major health impacts too. Moreover, it does not measure the direct relationship between a drought and the population living in, or depending on, drought-affected areas. It is not possible to do a population-based weighting because many people affected by a drought may not live in the area affected, e.g., in the case of droughts affecting agricultural areas (which are generally sparsely populated) with impacts on the food supply. It is therefore difficult to determine the trends in persons affected by drought from the trends of severe drought areas.

Further work is required to link reported drought damages in societies to climatic indicators. This would require a better understanding of the exposure factors of populations.

Future form of the indicator

Further development of the indicator will focus on using a combination of indices that capture agricultural hydrological drought, and meteorological drought, and better capture the health implication of drought events.

Additional analysis

The percentage of global land area affected by extreme drought has been increasing since the early 1990s. A linear regression shows that, from 1991 to 2021, the percentage of global land area affected by at least 1 month of extreme drought has been increasing by 0.98% each year ($p = 1.41x10^{-9}$, $R^2 =$ 0.7229) (

Figure **25**). The Middle East and North Africa region, together with the Horn of Africa and South America, were the regions most affected by an increase in the number of months in drought during the period 2012-2021, with respect to the period 1951-1960. (

Figure **26**). An increasing percentage of the global land area is affected by more extreme drought, with sharp increases in the global land area affected by severe, extreme, and exceptional drought events in any given month over the past 20 years (Figure 27).

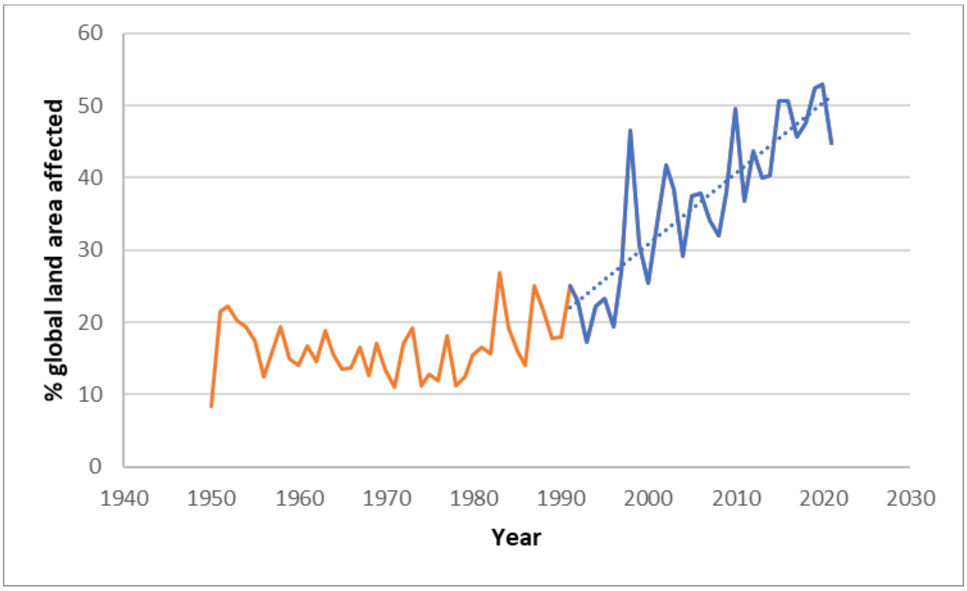

Figure 25. Percentage of the global land area affected by at least 1 month in extreme drought (SPEI6 ≤ -1.6). The dashed blue line represents the linear regression of the segment marked as a continuous blue line (1991-2021). The linear regression has slope = 0.98, p = 1.41x10-9, R^2 = 0.7229.

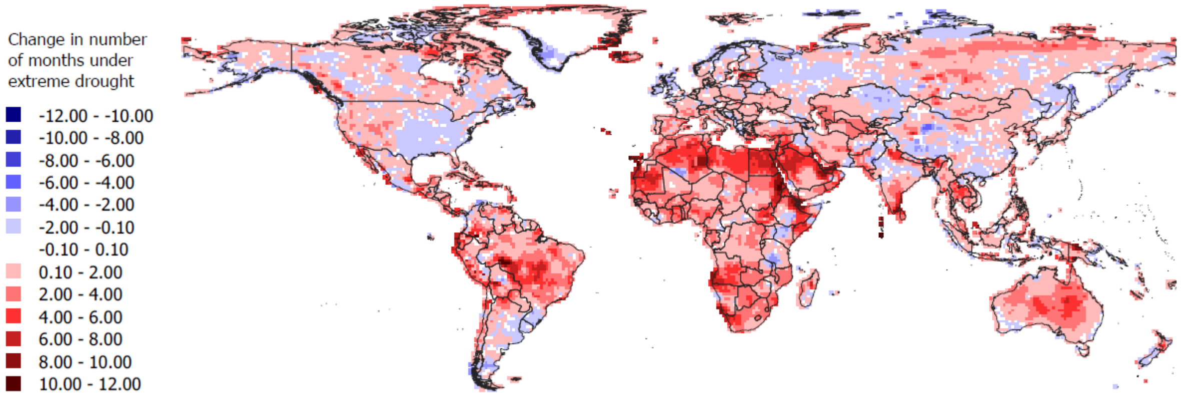

Figure 26. Change in the average annual number of months affected by extreme drought in the period 2012–2021 with respect to period 1951–1960

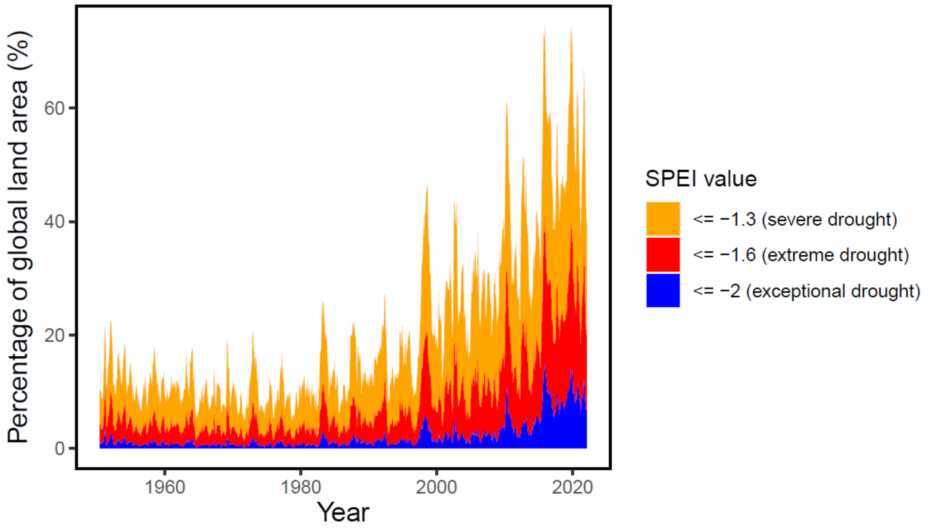

Figure 27. Global land area affected by drought events per month. Severe drought is defined by a SPEI of ≤1.3; extreme drought is defined by a SPEI of ≤1·6 and exceptional drought is defined by a SPEI of ≤2.

Indicator 1.2.3: Extreme weather and Sentiment

Methods

This indicator was first introduced in the 2021 Report of the *Lancet* Countdown on Health and Climate Change.

This second version of the indicator tracks both the effect of heatwaves, defined previously in indicator 1.1.2, and extremely wet days (exceeding the 99th percentile of local daily precipitation), on the sentiments of billions of geolocated expressions across millions of global Twitter users (Figure 28).

High-resolution spatiotemporal data describing human emotional states for large populations are difficult to come by, logistically complex to collect across country borders and cost prohibitive to repeatedly and consistently measure at the resolution and global scale required to track responses over time while controlling for other spatial and temporal factors that might otherwise confound inference between climate-related weather extremes and psychosocial responses. Although no perfect solution is presently available that captures the full spectrum of climate-related mental health burdens, global social media data provides a readily accessible, spatially extensive, highly resolved, social-ecological tool to measure large-scale emotional state responses to climate-related temperature and precipitation extremes.

The indicator is based on a dataset consisting of billions of social media posts, representing nearly all globally geo-localised tweets (within the daily volume limits of the full Twitter Firehose) from 2015–2021. The geo-tagged tweets constitute approximately two percent of all tweets, and thus may be somewhat limited in their generalisability due to opt-in geo-localisation. That said, consistent functional responses to meteorological variables have been uncovered across social media platforms, including massive samples of status posts from Facebook, Chinese Weibo (Twitter-style) posts, and Twitter geo-located data from multiple countries.[81-83] There appears to be little reason to suspect that the Twitter data is substantially biased from the overall relationship between climatic variables and emotional expressions. The functional relationships are nearly identical across platform and location and the estimated effects of heat are consistent with those uncovered in a high frequency national survey study.[84]

The analysis for this indicator followed the methodological approach employed in multiple peer-reviewed publications.[11,82,83,85,86] Climate econometric methods were employed[87,88] to track the causal relationship between observationally measured sentiment expressions and exposure to varying ambient heat and precipitation extremes.

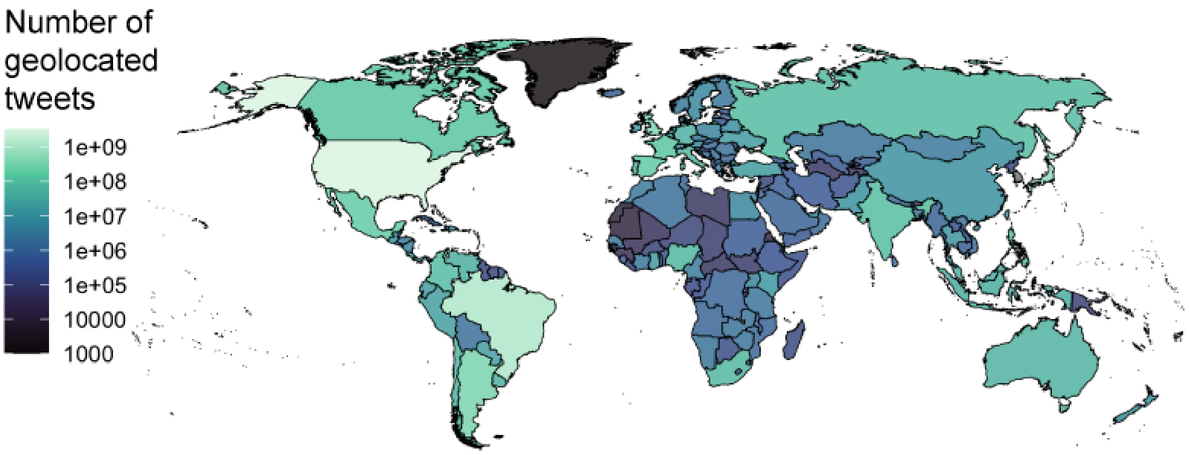

Figure 28. Country-level count of geolocated tweets, 2015-2021. Data includes posts from over 190 countries and 43700 administrative-2 divisions (ex. counties).

The social media data consisted of billions (>7.7 billion) of geolocated tweets collected via the Amazon Web Services servers from the Twitter Streaming API between 2015 and 2021. These tweets spanned the globe, with a median number of unique active daily users of approximately 900,000. Activity was highest in more populous and wealthier countries, though Twitter use continues to expand globally.

The positive and negative valence[89] of each of the Twitter posts was classified using the Linguistic Inquiry Word Count (LIWC) sentiment classification tool[90-92] across thirteen available languages: Dutch, English, French, German, Italian, Japanese, Mandarin, Portuguese, Romanian, Russian, Serbian, Spanish, and Ukrainian which provided broad geographic coverage for our sample. **Table 10** presents the by-language breakdown in the distribution of tweets from the 2022 indicator. Tweets with a 'lang' field matching each respective language are classified using that language's dictionary. This year's indicator employs a wider linguistic inclusion criterion, including tweets from two additional languages with LIWC dictionaries: Japanese and Mandarin.

**Included Language and LIWC Dictionary**	**% of total geotagged tweets in data 2015–2021**
Dutch	0.49%
English	65.60%
French	1.94%
German	0.49%
Italian	0.83%
Japanese	5.72%
Mandarin	0.13%
Portuguese	12.13%
Romanian	0.07%
Russian	1.19%
Serbian	0.01%
Spanish	11.32%
Ukrainian	0.06%

Table 10. By-language breakdown in the distribution of collected tweets

.LIWC is one of the most highly validated psychometric sentiment classification tools and has been employed in multiple studies on the relationship between climatic variables and online emotional expressions.[90,91,93-96] Further, the effects observed via the LIWC classifier have also been observed via the use of alternative classifiers in both the U.S.[81] and Chinese[83] context.

To enable the analysis that underpins this indicator, geolocated social media posts were geospatiotemporally matched with daily 30km gridded ECMWF ERA5 reanalysis ambient 2m air temperature data,[97] precipitation totals, and meteorological controls at the 2nd-administrative level (GADM version 3.6). This ECMWF product provides globally consistent spatial and temporal coverage. Daily 30km gridded meteorological data were employed from the ECMWF ERA5 reanalysis product from 2015 to 2021. Heatwave metrics were calculated employing the methods used by indicator 1.1.2. Further, measures of r99p extreme daily precipitation (>99th percentile precipitation for a given location during the recent historical record using the same 1986–2005 climate normal used in The *Lancet* Countdown's heatwave definition[11]), cloud cover, relative humidity, diurnal temperature range and wind speed were incorporated from the ERA5 data. The r99p "extremely wet day" threshold is an established climatological index for extreme precipitation events and has been widely used to track global increases in extreme precipitation over land in recent decades.[98-101] The calculation of the heatwave indicator followed the procedure outlined in.[11] To aggregate the meteorological variables, weather timeseries were extracted from the gridded ERA5 raster data at the second administrative division-resolution for each day in the data.

The primary spatial unit of analysis for the statistical investigation was the second administrative division-level (ex. county-level). The temporal unit of analysis was the calendar date, resulting in second-administrative-unit-by-day analyses.

To aggregate the sentiment measures to this unit of analysis, procedures previously described were followed.[81] Namely, for both positive and negative sentiment, each tweet was coded as either zero if the tweet contained no matching sentiment terms or one if it contained terms that match the corresponding sentiment. A tweet can express both positive and negative sentiment, only one of the two, or neither. For each day in the data, the average positive sentiment and the average negative sentiment was calculated for each unique user on that day, multiplying by 100 to produce a percentage. Users' scores were then averaged within the same second-division administrative unit together to produce the daily administrative sentiment measures. These measures ranged between 0 and 100.

Models drawn from climate econometrics were employed to estimate the effect of exposure to heatwaves on positive and negative sentiment; modelling the dependent variables as positive and negative sentiment, respectively, the primary independent variable an indicator of whether or not an administrative-unit-day was experiencing a heatwave. The model additionally included an indicator variable for whether a location was experiencing an extremely (>99th percentile) wet day, and controls for other meteorological conditions. To control for potentially confounding factors that may vary over time across different locations calendar-month-by-2nd-administrative region fixed effects were included in the models. Calendar date (ex. "2019-11-01", "2020-11-01") fixed effects for each unique date of observation was also included to account for idiosyncratic day-specific effects and global trends in internet and social media use.[88,102-105]

The multivariate fixed effects model estimated largely replicated that estimated in Baylis et al[81] and is as follows:

$$Y_{jmt} = \beta HEAT_{jmt} + \delta HPRCP_{jmt} + \mathrm{h}(\mu) + \gamma_t + \nu_{jm} + \epsilon_{jmt}$$

Here *j* indexed 2nd-level administrative region units, *m* indexed unique calendar months, and *t* indexed unique calendar dates. Y_{jmt} represented our dependent variables of positive and negative sentiment rates, respectively, $HEAT_{jmt}$ represented our binary heatwave indicator, which equals one if the date is classified as a heatwave in location *m* and equals zero otherwise. $HPRCP$ represented our extreme precipitation indicator. β was our main coefficient of interest, the effect of a heatwave on positive and negative sentiment rates in percentage points. δ was our secondary coefficient of interest, the effect of an extreme precipitation event on sentiment rates, $\mathrm{h}(\mu)$represented our meteorological controls, which included 20 percentage point percentile-bin controls for the temperature observations (with the omitted category of the 40th-60th temperature percentile bin serving as the omitted reference category for $HEAT_{jmt}$). $\mathrm{h}(\mu)$ also included flexibly binned control variables for cloud cover percentages, relative humidity, and wind speed.

Further, γ_t represented date-specific fixed effects that controlled for any idiosyncratic shocks in the data as well as factors that trended similarly over time across all locations. ν_{jm} indicated second-administrative-unit-by-calendar-month fixed effects that controlled for any location-specific seasonal and secular trends that might confound inference. ϵ_{jmt} represented our error term. Based on methodology in Baylis et al,[81] errors on administrative-unit-by-month and date were clustered and the regressions by the number of unique twitter posts in each administrative-unit-day and estimated the model for each year within the data were weighted, giving a β for each year presented (Figure 29).

Lastly, an exploratory subgroup analysis across human development groups by stratifying the global Twitter data according to the UN's Human Development Index (HDI) was conducted. The data were grouped into "high development" countries (operationalized as "very high" and "high" HDI countries) and "developing" country contexts ("medium" and "low" HDI countries), following the HDI-defined classifications,[106] and employed the same model specification as above on the two subgroups.

Data

1. Climate data from the European Centre for Medium-Range Weather Forecasts (ECMWF) ERA5 reanalysis.[107]
2. Geolocated tweets collected via the Twitter Streaming API.

Caveats

Although this indicator has many inferential strengths, particularly as compared to existing survey-based and surveillance-based methods, it is neither a perfect nor exhaustive measure of the subclinical mental health burden of heatwaves and weather extremes.

Countries that did not have Twitter broadly available to the public—such as China—were underrepresented in the indicator, despite the addition of Mandarin tweets this year. Second, geo-tagged tweets constitute approximately two percent of all tweets and thus may be somewhat limited in their generalisability due to opt-in geo-localisation. However very similar effects have been consistently documented across social media platforms, including massive multi-country samples of status posts from Facebook, Chinese Weibo (Twitter-style) posts, and Twitter geo-located data.[81,83,86] There appears to be little reason to suspect that the Twitter data is substantially biased from the overall relationship between climatic variables and emotional expressions. The functional relationships are nearly identical across platform and location.

Third, since higher income populations likely have greater access to adaptive amenities (air conditioning, etc.), the estimates produced by the identification strategy may be conservative (biased towards zero) for those disproportionately exposed to some of the hottest conditions in poorer socioeconomic contexts. However, a recent national analysis in China[83] suggests similar functional response forms across socioeconomic contexts, with very similar magnitudes observed for extreme heat-related responses, suggesting that added income may only smooth the relationship to a more moderate degree, and primarily for cold temperatures rather than warm ones.

Future form of the indicator

Global internet use and social media connectivity are expected to continue to increase over the coming decade, likely further expanding the global reach and coverage of the sample. Whilst the focus of this current version of the indicator is on sentiment responses to heatwaves and precipitation extremes, future iterations can expand to cover expressed responses to additional climate-related environmental stressors, including floods, hurricanes/cyclones/typhoons, fires and smoke. Mirroring the approach taken with heatwaves in the current indicator, these extreme events can be registered using standard definitions, including those specified directly by The *Lancet* Countdown in future annual reports.

Additional analysis

During the baseline period of 2015–2020, local exposure to precipitation extremes and heatwaves reduced the rate of positive expressions and increased the rate of negative expressions. To provide scale for the new 2021 response estimates shown in indicator 1.1.5, the average effect of the spring daylight savings time transition

(DST) on changes in positive and negative sentiments was estimated (Figure 30, leftmost barplot). From 2015–2021, 64 countries within the dataset observed DST on at least one occasion. Compared to the average response to the spring daylight savings time change, the average impact magnitude of a heatwave event on negative sentiment was nearly 5 times larger in 2021. By comparison, the impact of an extreme precipitation event on diminished positive sentiment in 2021 amounted to 70% of the detrimental effect of switching to daylight savings time. Similarly, the impact of a single heatwave on reduced positive sentiment was equivalent to 53% of the reduction induced by the clock change to DST.

The impact signatures of heatwaves and precipitation extremes differed across positive and negative sentiment responses. From 2015–2020, the average impact of extreme precipitation on negative sentiment was over twice as large as the equivalent impact of a heatwave. Conversely, the average impact of a heatwave day on attenuated positive sentiment was over 1.5 times the magnitude of the estimated effect of an extremely wet day. In 2021 both the effect of heatwaves on diminished positive sentiment and the effect of extreme precipitation on elevated negative sentiment were below their corresponding 2015–2020 average effect sizes.

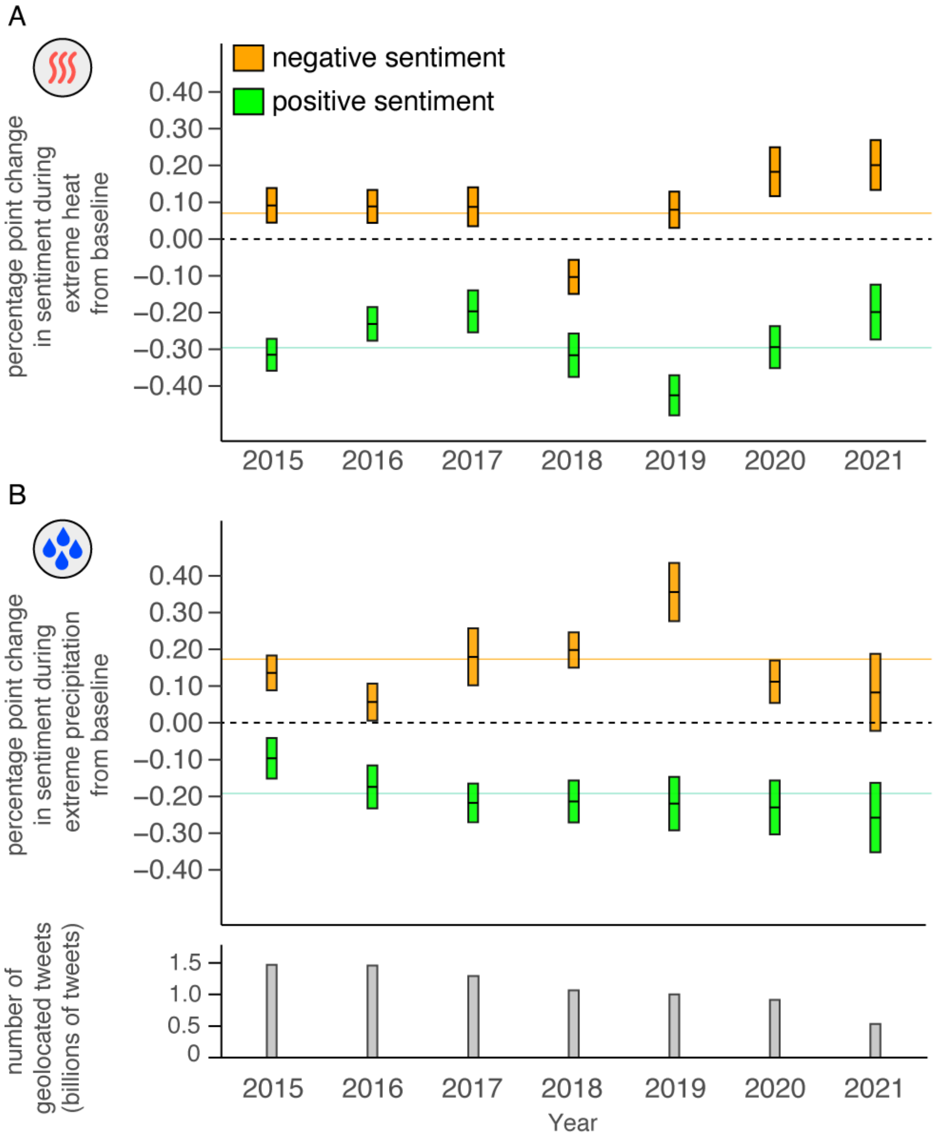

Figure 29: Sentiment responses to heatwaves and extreme precipitation. (A) Annual effect of heatwave exposure on positive (green) and negative (orange) sentiments, derived from the textual expressions of over 7.7 billion geolocated Twitter posts around the world. Boxes depict 95% CIs of the estimated average percentage point change in the rate of sentiment expressions during days with heatwaves compared to the average temperature baseline for each location and year. (B) Effect of exposure to extremely wet (>99th percentile local daily precipitation) days on expressed sentiments compared to local daily average precipitation. Coloured horizontal lines depict the 2015-2020 average effect of climate

extremes on positive (green) and negative (orange) sentiments. Grey bars show the geolocated Tweet count by year of record.

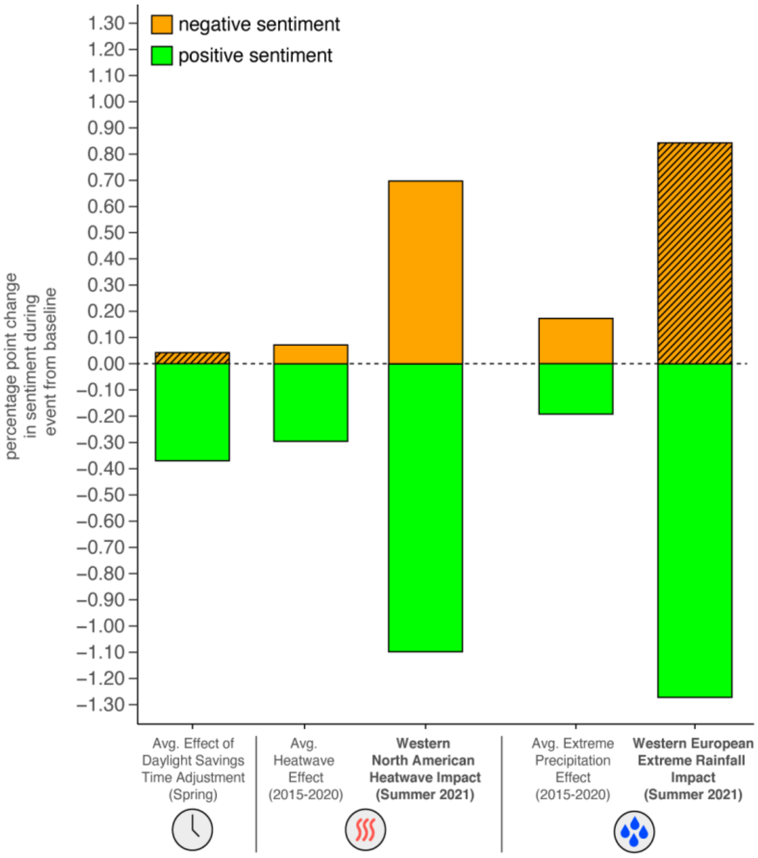

Figure 30. Estimated average effects of the 2021 Western North American Heatwave and Western European Extreme Rainfall events on rates of positive (green) and negative (orange) sentiment expressions on Twitter. For comparison, sentiment effect sizes are also shown for the impact of the spring daylight savings time change, average 2015–2020 heatwave impact and average 2015-2020 extreme precipitation response. Solid bars without stripes indicate response estimates that were also statistically significant at the p <.05 level.

The impact signatures of heatwaves and precipitation extremes differed across positive and negative sentiment responses. From 2015–2020, the average impact of extreme precipitation on negative sentiment was over twice as large as the equivalent impact of a heatwave. Conversely, the average impact of a heatwave day on attenuated positive sentiment was over 1.5 times the magnitude of the estimated effect of an extremely wet day. In 2021 both the effect of heatwaves on diminished positive sentiment and the effect of extreme precipitation on elevated negative sentiment were below their corresponding 2015–2020 average effect sizes.

This year's indicator included a new component analysing the sentiment impacts of specific extreme climate events that transpired in 2021 (Figure 30). All heatwave and extreme rainfall events that underwent rigorous extreme event attribution analyses by the Worldwide Weather Attribution initiative (and that were subsequently found to have been made more likely due to climate change) were selected. For 2021, these events consisted of both the summer Western North American Heatwave and the Western European Extreme Rainfall event. During the last week of June and first week of July 2021, the Pacific Northwest regions of both the United States and Canada sustained record-breaking temperatures, with populated urban areas in several states and provinces exceeding 40°C.[108,109] Over a thousand excess deaths were attributed to this extreme heat event,[110,111] which also saw the highest daily maximum temperature (49.6°C) ever recorded in Canada. Lytton, the town that registered the record, was subsequently destroyed by wildfire the next day.[108] The World Weather Attribution initiative's analysis found that the extreme regional heat was "virtually impossible without human-caused climate change".[112] The analysis of geolocated Twitter posts during this period suggests that exposure to the Western North American

Heatwave amplified negative sentiment nearly ten times as much as the average 2015–2020 heatwave impact, and reduced positive sentiment by over three-and-a-half times as much as the average 2015–2020 impact, constituting exceptionally severe psychosocial effects in the Twitter timeseries (Figure 30).

Shortly thereafter, in mid-July of 2021, historically heavy rainfall across Western Europe interacted with underlying topography and land cover characteristics to produce extreme flooding in the German states of Rhineland-Palatinate and North Rhine-Westphalia, along the river Meuse and adjacent regions. The event was linked to at least 184 fatalities in Germany alone and substantial damage to housing, livelihoods and essential infrastructure.[113] In a subsequent analysis by the World Weather Attribution initiative, the likelihood of such regionally extreme rainfall — the main meteorological driver of this event — was found to have increased due to climate change.[113] The analysis of all coinciding tweets in this region from July 11th to 16th suggests that the Western European Extreme Rainfall event significantly and substantially attenuated positive sentiment, reducing positive expressions over six-and-a-half times as much as an average extreme precipitation event during the 2015–2020 period. The effect of the 2021 Western European Extreme Rainfall event on negative sentiment was nearly five times the 2015–2020 average impact, although there was substantial imprecision in this latter estimate (Figure 30). The relative scale of these observed impacts on expressed human emotional states — in some cases nearly an order of magnitude larger than the typical extreme weather event impact observed from 2015–2020 — underlines the importance of quantifying the impacts of events located at the tails of shifting local weather distributions, including those that exceed historical extreme climate event thresholds.

New to this year's indicator, the sentiment response to extreme precipitation was compared across very high-high HDI countries and medium-low HDI countries (Figure 31). Extreme precipitation was found to reduce positive sentiment to a greater extent in medium-low HDI countries compared to in very high-high HDI countries. Specifically, the attenuating effect of extremely wet days on positive sentiment in medium-low HDI countries over the entire 2015–2021 data period was 50% larger than the estimated impact found among Twitter users in very high-high HDI countries. Conversely, extremely wet days amplified negative sentiment to a greater extent in very high-high HDI regions. Consistent with prior reports, medium-low HDI countries exhibited a greater magnitude increase in negative sentiment but negligible change in positive sentiment during heatwaves compared to very high-high HDI countries. See the appendix of the 2021 report for additional analysis and discussion of the estimated differential sensitivity to heatwaves across human development.

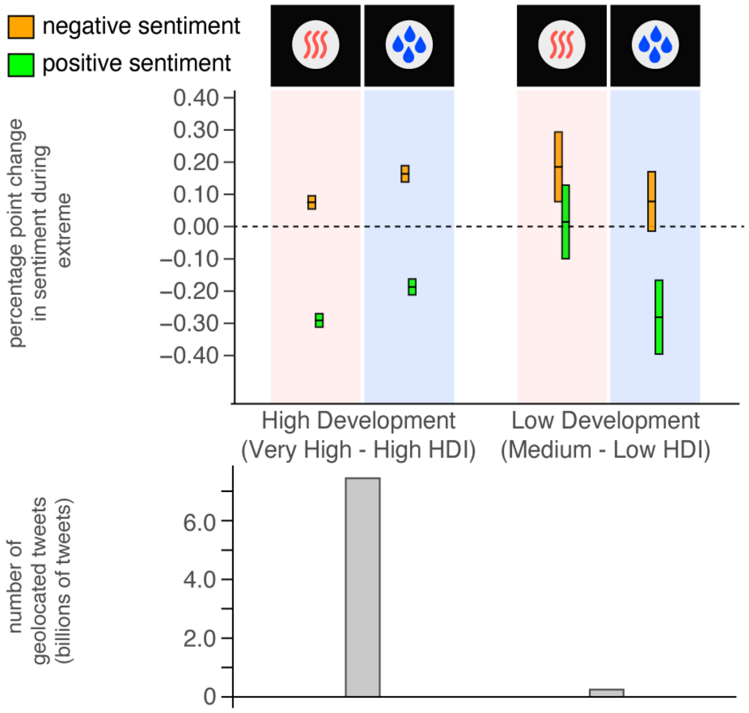

Figure 31. Average impacts of extreme heat and extreme precipitation exposure on the positive and negative sentiments of Twitter expressions from 2015-2021, stratified by Human Development Index

groups. Coloured intervals show 95% CIs of the estimated average percentage point change in rates of positive (green) and negative (orange) sentiment expressions during days with extremes, compared to the location-specific meteorological baseline. Grey bars show the geolocated Tweet count by HDI grouping.

This year's indicator also features an investigation of the responses to extreme heat and precipitation across WHO geographic regions (Figure 32). The effect of heatwave exposure varied geographically, while the impact from extreme precipitation events was more precisely estimated and directionally uniform. Extremely wet days reduced the rate of positive expressions across all regions, with statistically significant impacts observed in the African, Americas, Eastern Mediterranean, European and South-East Asian WHO regions. Across the 2015–2021 data period, the most severe average impact of extremely wet days on reduced positive sentiment was uncovered for the Eastern Mediterranean WHO region (Figure 32D), while comparatively smaller average impacts were evident in the European and Western Pacific regions. Similarly, extremely wet days increased rates of negative expressions across most regions, with significant impacts observed in the Western Pacific, European and Americas regions. Exposure to extreme precipitation elevated negative sentiment to the greatest extent in the Western Pacific region, where the impact of extremely wet days was over three-and-a-half times the 2015–2020 global average effect (Figure 32B).

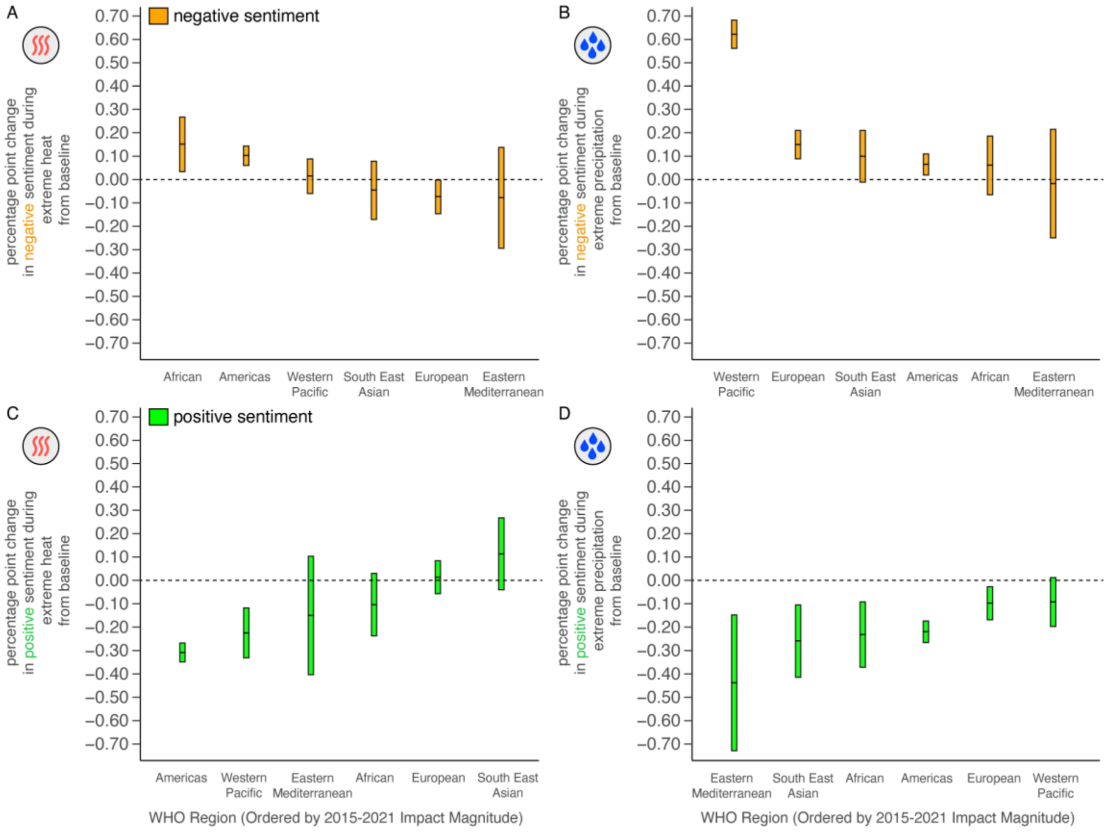

Figure 32. Effects of climate extremes on the rates of positive and negative sentiments of geolocated Twitter expressions from 2015-2021, stratified by WHO geographic regions. (A, C) The estimated average effects of a heatwave day during the 2015-2021 observation period on negative sentiment (orange) and positive sentiment (green) responses across the WHO African, Americas, Eastern Mediterranean, European, South-East Asian and Western Pacific regions. Boxes depict 95% CIs of the estimated average percentage point change in the rate of sentiment expressions during days with extremes compared to the

meteorological baseline for each location. (B, D) The estimated regional sentiment responses to extremely wet days, by WHO geographic region.

While heatwave exposure reduced positive sentiment across most WHO regions globally, statistically significant decreases in positive expressions were only found in the Americas and Western Pacific regions (Figure 32C). Among these regions, heatwave days attenuated rates of positive expressions to the greatest degree in the Americas. The impact of heatwaves on negative sentiment varied to a greater degree across regions, significantly amplifying negative sentiment in the African region as well as in the Americas (Figure 32A). The average impact of a heatwave in the African region on elevated negative sentiment was over twice the 2015–2020 global average effect. By contrast, the estimated effect of heatwaves on negative sentiment did not significantly differ from zero for the Eastern Mediterranean, South-East Asian and Western Pacific regions. Conversely, heatwaves were estimated to significantly reduce negative sentiment in European regions, possibly indicative of underlying climatological or adaptive differences. While globally extensive, Twitter coverage is not equally distributed (with relatively lower coverage in the Eastern Mediterranean, African and South-East Asian regions), limiting the precision of estimates in these regions.

1.3: Climate Suitability for Infectious Disease Transmission

Dengue, Chikungunya and Zika

Methods

The input data for this indicator have been improved and extended for the 2022 report.

Cases of dengue have doubled every decade since 1990, with 58.4 million (23.6 million–121.9 million) apparent cases in 2013, accounting for over 10,000 deaths and 1.14 million (0.73 million–1.98 million) disability-adjusted life-years.[114] Beside global mobility, climate change has been suggested as one potential contributor to this increase in burden.[115] *Aedes aegypti* and *A. albopictus*, the principal vectors of dengue, also carry other important emerging or re-emerging arboviruses, including Yellow Fever, Chikungunya, Mayaro, and Zika viruses, and are likely to be similarly responsive to climate change.

R_0, i.e. the basic reproduction number, which is the expected number of secondary infections resulting from one single primary infected person case in a totally susceptible population ,was computed using the formula $R_0 = Vb_h/r_h$.[116] The vectorial capacity (V), which express the average daily reproductive rate of subsequent cases in a susceptible population resulting from one infected case, was computed using the formula $V = ma^2 b_m p^n/{-lnp}$ where a is the average vector biting rate, b_m *is* probability of vector infection and transmission of virus to its saliva, n is the extrinsic incubation period while p is the daily survival probability. All these parameters are temperature dependent and are further described in the work by Rocklöv et al.[116-118]

The ratio between number of mosquitoes to the number of humans, is central to V and the R_0 value (*m*), but often it is left out or estimated in a simple way. Here a model is used to estimate mosquito populations of *Aedes aegypti* and *Aedes albopictus* separately. The original mosquito-population models provide results in terms of the number of individuals of *Ae. aegypti* per breeding site (*X*), or the number of *Ae. albopictus* per hectare (*Y*).[119,120] In order to appropriately estimate *m*, i.e. mosquito population density per human population density (*p*), *X* was multiplied by $f(p,a,c) = a * g(p,c)$ where *a* equals to the number of breeding-sites per human, and *Y* by $f(p,a/b,c) = a * g(p,c)/b$ where *b* equals the average number of breeding sites per hectare. The function $g(p,c) = p^2/(c^2 + p^2)$ is an increasing sigmoidal function that equals the viability of domesticated mosquito-populations in relation to human population density. Accordingly, $f(p,a,c)$ is the multiplicative factor *m* in V, which allowed to straightforwardly estimate correct values for *a*, *a/b* and *c* by fitting R_0 to R_0-data that was available for a subset of the spatiotemporal points.[121]

Numerically V and abundance estimates was computed at 0.5°x0.5° spatial resolution based on ERA5-Land data[122] resampled from the 0.1°x0.1° original resolution. V and vector abundance were run for both *Aedes* aegypti and *Aedes* albopictus vectors. Gridded population from HYDE 3.2 (History Database of the Global Environment) were used in the computation of R_0. For Dengue (albopictus) and Chikungunya, *Aedes albopictus* vector abundance estimates were used in the computation of *m* while for Dengue (aegypti) and Zika *Aedes aegypti*

abundance estimates were used. Further annual length of transmission season (LTS) was computed by summing the number of months in a year when R_0 was greater than 1 following the work by Colón-González et al.[121]

The gridded R_0 and LST for Dengue (Aedes *aegypti),* Dengue (Aedes *albopictus),* Chikungunya (*Aedes albopictus*) and Zika (*Aedes aegypti*) were extracted and averaged by Country, WHO regions and according to human development index (HDI).

Data

1. Monthly climate data (2m air temperature, 2m dew point temperature, total precipitation) from the European Centre for Medium-Range Weather Forecasts (ECMWF) ERA5-Land reanalysis.[123]
2. Population data from HYDE 3.2.[124]

Caveats

Key caveats and limitations of the V model and its parameterisation are fully described in works by Liu-Helmersson et al.[125,126] and Rocklöv et al.[116] The predicted R0 should not be confused with actual dengue cases, although it is an indicator of the potential for outbreaks.[117,118]

Additional analysis

The risk of outbreak in arboviral diseases is more pronounced for countries with very high HDI while countries with low HDI have observed a decrease in epidemic risk over time (Figure 33). Suitable months for potential outbreaks in *Aedes aegypti* transmitted dengue and Zika have increased in countries with medium HDI while countries with high HDI have observed expansion in suitable transmission months for *Aedes albopictus* transmitted dengue and Chikungunya (Figure 34). In overall, transmission intensity during peak months have increased globally over the years (Figure 35).

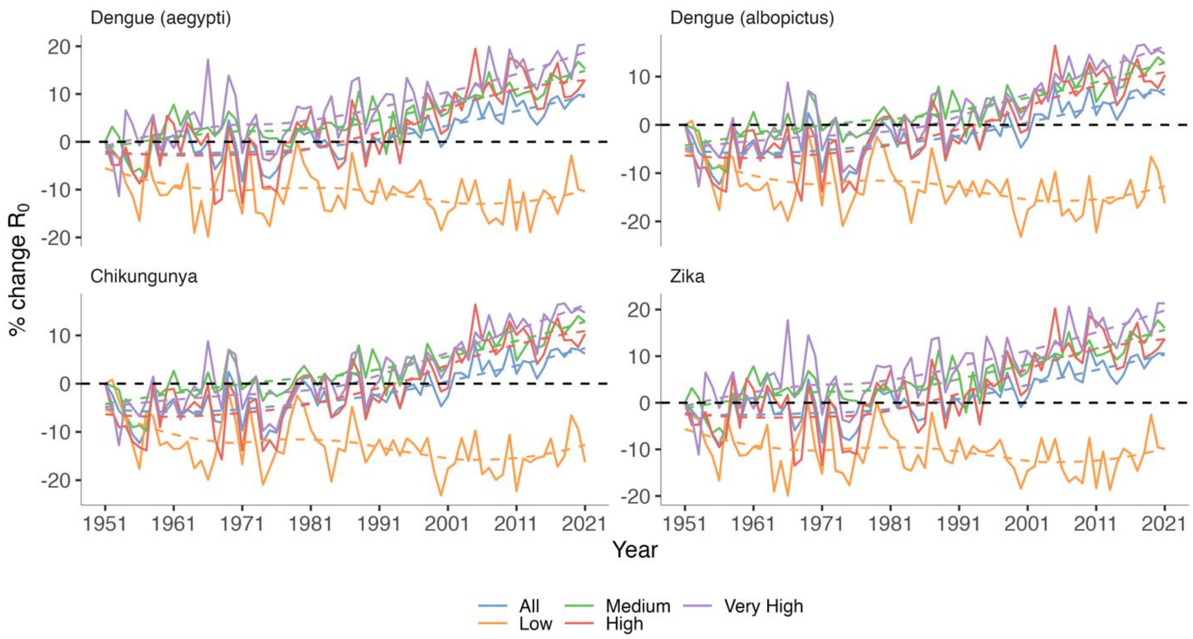

Figure 33. Percentage change in R_0 relative to 1951 by HDI level for Dengue, Chikungunya and Zika in the 1951–2021 period.

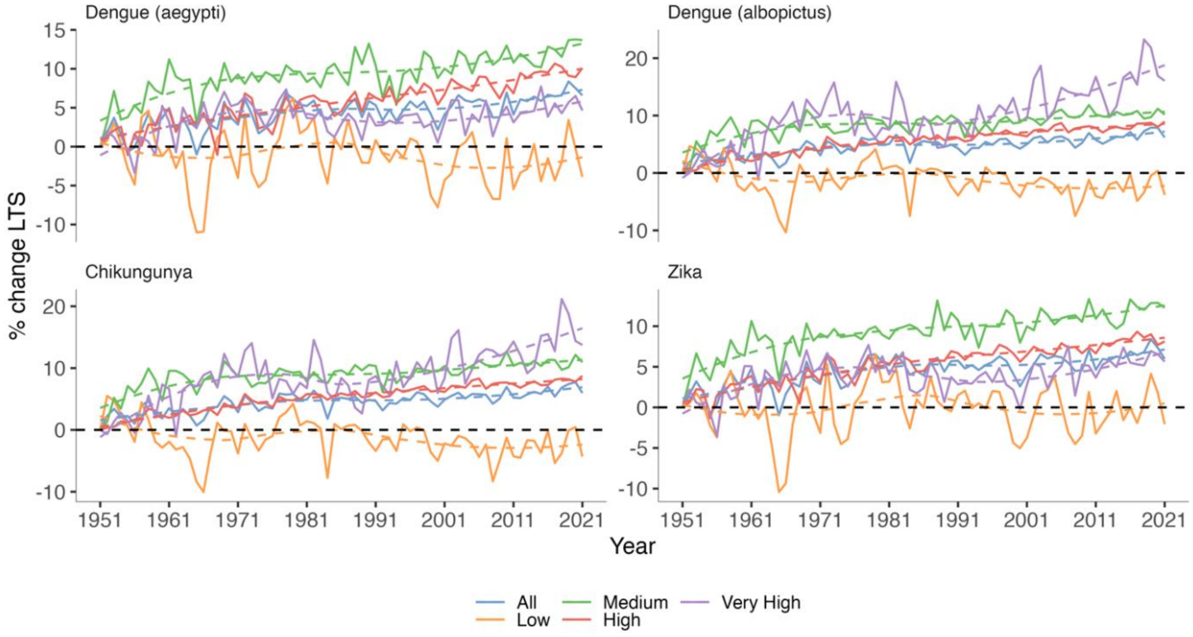

Figure 34. Percentage change in length of transmission season (LST) relative to 1951 by HDI level for Dengue, Chikungunya and Zika in the 1951–2021 period.

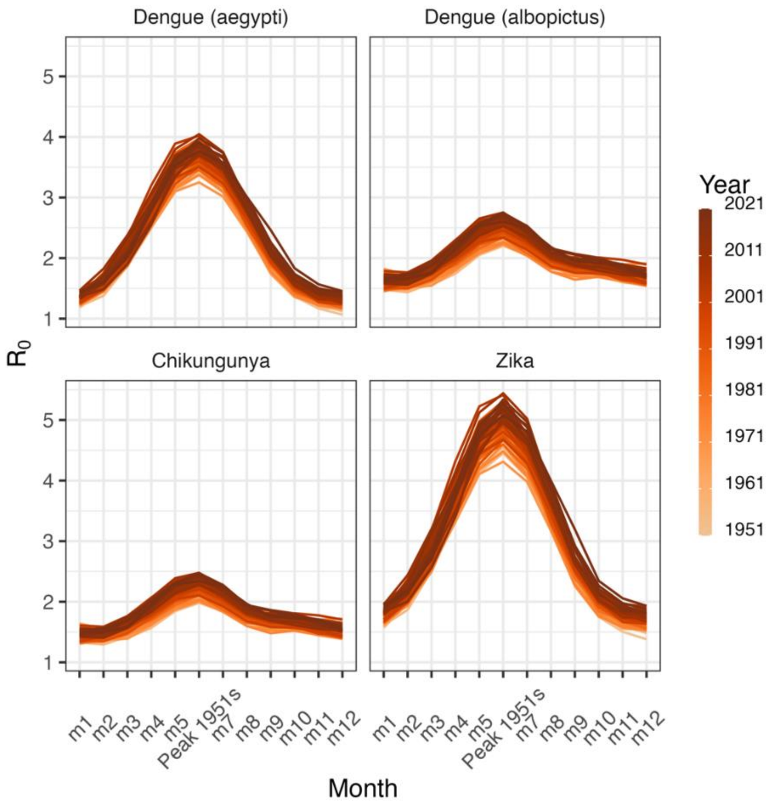

Figure 35. Change in seasonality of global vectorial capacity for Dengue, Chikungunya and Zika in the 1950–2020 period. Countries R_0 centred around the 'peak month' in the baseline year 1951.

Malaria

Methods

The methodology and input data for this indicator have been improved and extended for the 2022 report.

The malaria indicator focuses on determining global changes in the number of months per year suitable for transmission of the malaria parasites over time between high- and lowland areas according to different categories of the UNDP Human Development Index.

The length of the transmission season, measured as the number of months suitable for malaria transmission per year from 1950-2021, was calculated at 0.1° x 0.1° spatial resolution. Climate suitability was based on empirically derived thresholds of precipitation, temperature, and relative humidity for *Plasmodium falciparum*.

Monthly climate information between 1950 and 2021 were obtained from the ERA5-Land climate reanalysis dataset.[122] Relative humidity in percentage was calculated using the August-Roche-Magnus equation, which derives this value by combining dew point temperature and temperature, using the formula below.[127]

$$\boldsymbol{RH = 100 * \frac{\exp\left(\frac{aT_d}{b+T_d}\right)}{\exp\left(\frac{aT}{b+T}\right)}}$$

where a and b are the coefficients 17.625 and 243.04, respectively, and T and T_d are temperature and dew point temperature in °C.

Elevation data were extracted from the JISAO repository, University of Washington.[128]

Land cover data were downloaded from the Copernicus Global Land Monitoring Service repository at 100m resolution.[129] The land cover raster from 2019 was downloaded and assumed to be constant throughout the time series. Suitable land classes were determined according to the literature about the environmental requirements of the dominant vector species (DVS) of human malaria.[130,131] Namely, close and open forests, herbaceous wetlands, cultivated and managed vegetation/agriculture, and permanent water bodies, were considered as potentially suitable areas for settlement of *Anopheles* mosquito populations.

Suitability for a particular month was defined as the coincidence of precipitation accumulation greater than 80 mm, average temperature between 18°C and 33°C, and relative humidity greater than 60%, in land classes suitable for *Anopheles* mosquitoes. These combined values reflected the limits for potential transmission of *Plasmodium falciparum* parasites. The number of months with suitable conditions was calculated at the finest possible resolution, nine kilometres, and later averaged to country, WHO region and HDI level. The yearly products were later stratified by elevation using a threshold of 1500m a.s.l. for splitting low- from highland areas (highlands ≥ 1500m a.s.l.).

Data

1. Monthly climate data (2m air temperature, 2m dew point temperature, total precipitation) from the European Centre for Medium-Range Weather Forecasts (ECMWF) ERA5-Land reanalysis.[123]
2. Elevation data from the University of Washington Joint Institute for the Study of the Atmosphere and Ocean (JISAO).[128]
3. Land cover data from the Copernicus Global Land Monitoring Service.[132]

Caveats

These results are based on climatic data, not malaria case data. The malaria suitability climate thresholds used are based on a consensus of the literature. In practice, the optimal and limiting conditions for transmission are dependent on the particular species of the parasite and vector.[133] Control efforts might limit the impact of these climate changes on malaria or conversely, the climate suitability may either enhance or hamper control efforts.[134]

Additional analysis

The indicator is visualised using time series line plots (Figure 36, Figure 37) and maps containing the change in the number of suitable months between the decades 1950–1959 and 2012–2021, also for WHO region and HDI levels (Figure 38, Table 11, Table 12).

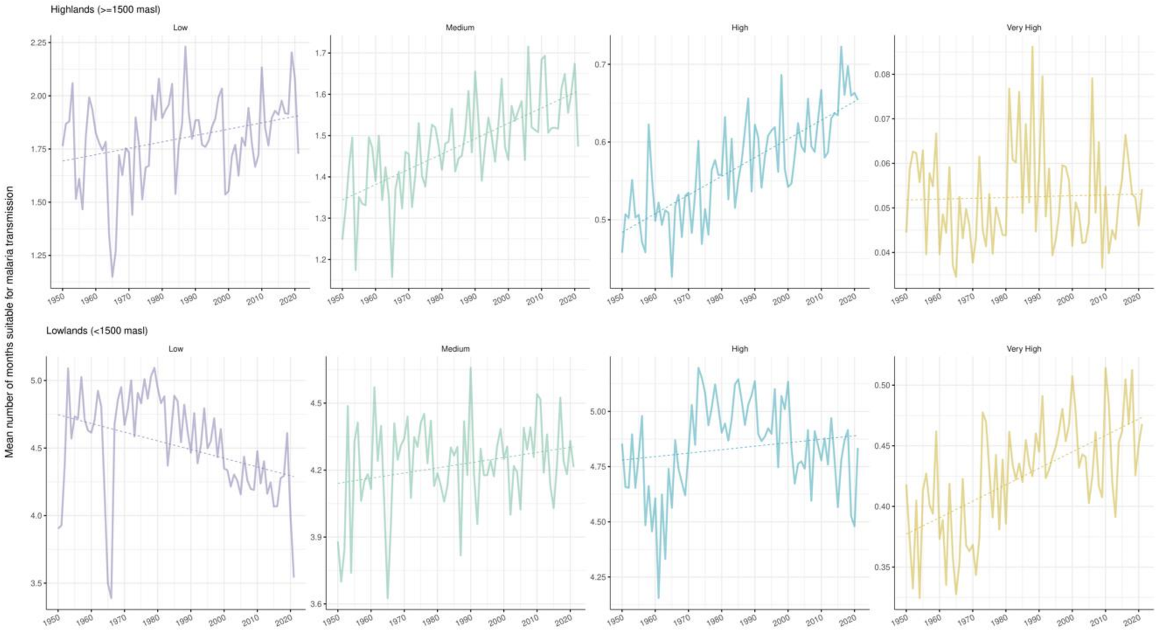

Figure 36. Mean number of months suitable for P. falciparum transmission between 1950 and 2021. Suitability was defined as months with precipitation accumulation greater than 80mm, average temperature between 18°C and 33°C and relative humidity greater than 60%, in land classes suitable for Anopheles mosquitoes. Results stratified by HDI levels are shown for highlands (≥1500m a.s.l.) and lowlands (<1500m a.s.l.). Linear regression was used for trend estimation.

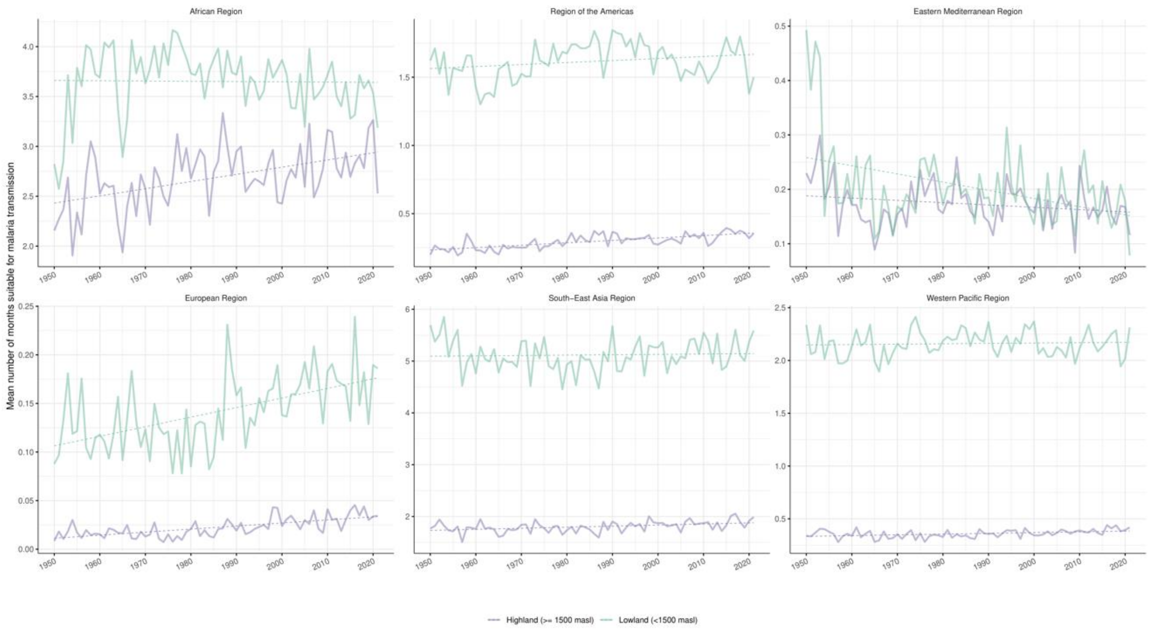

Figure 37. Mean number of months suitable for P. falciparum transmission between 1950 and 2021. Suitability was defined as months with precipitation accumulation greater than 80mm, average temperature between 18°C and 33°C and relative humidity greater than 60%, in land classes suitable for

Anopheles mosquitoes. Results stratified by WHO region are shown for highlands (≥1500m a.s.l.) and lowlands (<1500m a.s.l.). Linear regression was used for trend estimation.

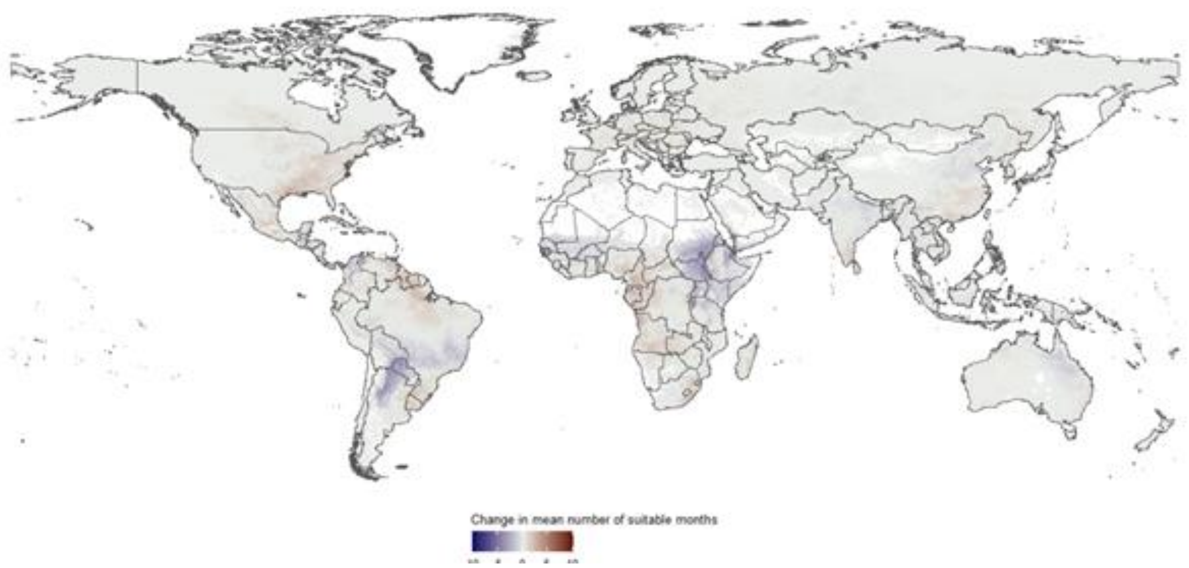

Figure 38. Change in the length of transmission season from 1951–1960 to 2012–2021. Change in length of the malaria transmission season is measured as the number of months per year with precipitation accumulation greater than 80mm, average temperature between 18°C and 33°C and relative humidity greater than 60%, in land classes suitable for *Anopheles* mosquitoes.

HDI level	Highlands *(≥1500 masl)*	Lowlands *(<1500 masl)*
Low	7.6%	-10.2%
Medium	13.4%	3.4%
High	26.67%	1.34%
Very high	-5.7%	16.7%

Table 11. Percentage change in mean number of months suitable for malaria transmission. Comparison between the period 1951–1960 and the period 2012–2021, stratified by HDI level and high/lowlands.

WHO Region	Highlands *(≥1500 masl)*	Lowlands *(<1500 masl)*
African Region	14.9%	-3.4%
Region of the Americas	32.1%	3.3%
Eastern Mediterranean Region	-21.9%	-42.7%
European Region	95.7%	40.7%
South-East Asian Region	9.7%	-0.6%
Western Pacific Region	11.8%	3.5%

Table 12. Percentage change in mean number of months suitable for malaria transmission. Comparison between the period 1951–1960 and the period 2012–2021, stratified by WHO region and high/lowlands.

Vibrio

Methods

The methodology for this indicator remains similar to that described in the 2021 report of the *Lancet* Countdown.[11]

This indicator focuses on mapping environmental suitability for pathogenic *Vibrio* spp. in coastal zones globally (<30km from coast). *Vibrio* spp. are globally distributed aquatic bacteria that are ubiquitous in warm estuarine and coastal waters with low to moderate salinity. *V. parahaemolyticus, V. vulnificus,* and non-toxigenic *V. cholerae* (non-O1/non-O139) are pathogenic in humans. These *Vibrio* species are associated with sporadic cases of gastroenteritis, wound infections, ear infections, or septicaemia in circumscribed localities.

Vibrio ecology, abundances, distributions, and patterns of infection are often strongly mediated by environmental conditions.[135-137] On the basis of the consensus in the literature on what environments *Vibrio* infections may thrive, the indicator uses thresholds of >18°C for Sea Surface Temperature (SST) and <30 PSU for Sea Surface Salinity (SSS). Estimates for SST were obtained from NOAA Optimum Interpolation 1/4 Degree Daily Sea Surface Temperature (OISST) Analysis version 2 for the period 1982-2020. Estimates of SSS were created from daily data obtained from Mercator Ocean Reanalysis.[138]

Here suitability is reported at two levels. First, the percentage of coastline that experienced suitable conditions for *Vibrio* infections was calculated globally and the results summarised across three latitudinal bands (northern latitudes = 40-70°N; tropical latitudes = 25°S-40°N; and southern latitudes = 25-40°S). Second, suitability in three focal regions in which human *Vibrio* infection is frequently observed – the Baltic Sea, the Pacific northwest (PNW), and the northeastern coast of the United States (36-50°N) – was calculated. For the Baltic, PNW, and northeastern coast of the United States the percentage of coastline suitable for *Vibrio* infections are presented. The percentage change figures reported in the main text were calculated relative to a 1980s baseline (8-year average, 1982-89) and considering the average for the last decade (10-year average, 2012-2021; this to illustrate the overall trend accounting for interannual variability) or the most recent year for which data were available (2021). The percentage of coastline has also been aggregated by country, WHO region and HDI level (low, medium, high and very high).

Data

1. Sea surface temperature data from the NOAA Optimum Interpolation 1/4 Degree Daily Sea Surface Temperature (OISST) Analysis version 2 for the period 1982-2020.[139]
2. Sea surface salinity data from the Mercator Ocean Reanalysis.[138]

Caveats

The results are derived on the basis of suitable SST and SSS conditions only, and do not include other potentially important drivers (e.g., globalisation), environmental predictors of pathogenic *Vibrio* infections (e.g., cholorphyll-*a*, turbidity) or disease case data. Nevertheless, these associations have been explored and are reported in the supporting references included above.

In the global analysis, the slope of the trendlines over the time series is mostly flat for the tropical/subtropical region and the southern Hemisphere. However, the SST-only suitability shows a strong upward trend in the southern hemisphere, indicating that on average temperature conditions are also improving growth conditions for *Vibrio* in these areas, while SSS is generally limiting. However, locally suitable SSS conditions will also occur in these regions based on, for example, variation in local rainfall and river runoff, which can make these regions sporadically suitable for *Vibrio* infections.

Future form of the indicator

The *Vibrio* indicator has considered two environmental factors so far, seawater temperature and salinity, missing socioeconomic and demographic aspects, which have been identified as key elements in disease transmission of *Vibrio* illness. The advent of a new generation of models, such as those participating in CMIP6 (Coupled Model Intercomparison Project 6), in combination with the new Shared Socioeconomic Pathways (SSPs), has provided an exceptional opportunity to introduce a wider prospect and more robust projections into the models, integrating an increasing resolution and with key socioeconomic drivers (economic growth, demography, education and technological development).

In the coming years, data from these models will be used to develop new projections within the Inter-Sectoral Impact Model Intercomparison Project (ISIMIP) framework to gain a higher level of integration between different sectors and spatial scales. Climate, population and socioeconomic projections will be joined to generate more realistic estimates of past, present and future changes in Vibrio suitability, and provide a global estimate of the population at risk of vibriosis for the different periods.

Additional analysis

This Latitude-time plot (Hovmoller diagram, Figure 39) indicates poleward expansion of suitable environments for *Vibrio* spp. in this region. For latitudes >°39 and similarly to the Baltic Sea, there is a general widening of the *Vibrio* spp. season as well as an increase in the amount of shoreline affected.

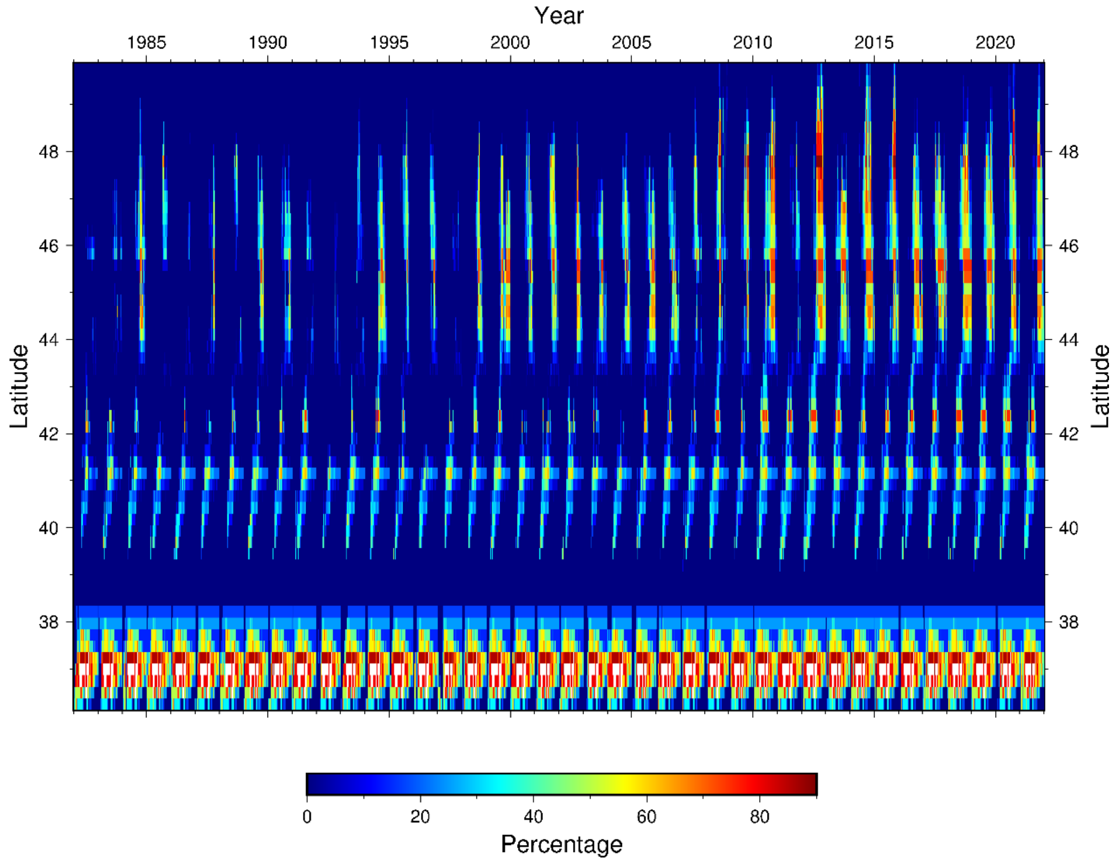

Figure 39. Percentage coastline suitable for Vibrio spp., V. parahaemolyticus, V. vulnificus, and non-toxigenic V. cholerae (non-O1/non-O139), by latitude along the United States northeast coastal region (36°N–50°N).

Considering the percentage of coastline aggregated by WHO region and HDI level, time series show a positive trend, an indication of the expansion of the areas showing suitable conditions for *Vibrio*. The WHO regions of EMR (Eastern Mediterranean Region), EUR (European Region) and WPR (Western Pacific Region) have the largest positive trends, with increases of approximately 0.9%, 0.55%, and 0.5% per decade, respectively. For the HDI, the most noticeable feature is the abrupt increase (resp. decrease) in the percentage values for high level (resp. very high) HDI in 2013, as the net result of Russia switching categories, from high to very high, that year.

Vibrio cholerae

Methods

The methodology for this indicator was improved from the 2021 report of the *Lancet* Countdown.

Cholera is a water-borne disease caused by the bacterium *Vibrio cholerae*, which generally occurs in coastal waters before spreading inland.[140] Improvements in water sanitation have reduced the burden of cholera worldwide.[141] Nevertheless, the ongoing, 7th cholera pandemic generates ~2.8 million cases annually and at least 95,000 deaths per year, mainly in cholera-endemic countries.[142] Cholera control includes safe drinking water, cholera vaccination, and effective and timely treatment. In this indicator global coastal conditions are assessed to estimate the annual global suitability for *V. cholerae* between 2003-2020. New to 2022, the indicator includes the human populations potentially exposed to coastal *V. cholerae* based on transmission risk in the most immediate coastal waters (10 km from the coast).

Analyses were performed following an ecological niche modelling protocol.[143,144] First, a comprehensive dataset of *V. cholerae* occurrence and seawater data was ensembled for the last two decades (Table 14). Second, each *V. cholerae* record was carefully curated following standardised data-cleaning protocols[145,146] to reduce bias and errors; records were linked to coastal water conditions (sea surface temperature and chlorophyll-*a*) of the site and date of *V. cholerae* sample collection. Then, an ecological niche model of *V. cholerae* was developed using a once-class support vector machines model.[147] The model was projected to annual seawater conditions globally to map the specific coastal areas suitable for *V. cholerae* at ~4 km^2 spatial resolution during the period from January 2003 to December 2020.[148] *Vibrio cholerae* annual models were used to estimate human populations in 2000, 2005, 2010, 2015, and 2020, potentially exposed to *V. cholerae* transmission risk in inland areas from the NASA GPWv4 population dataset at 1 km spatial resolution. Annual *V. cholerae* models were compared with a baseline of average conditions between 2003-2005 to identify variation across time. Table 13. Sources of Vibrio cholerae records.

Table 14. Sources of Vibrio cholerae records.

1. Atlantic, S., Martinelli Filho, J. E., Lopes, R. M., Rivera, I. N. G. & Colwell, R. R. *Vibrio cholerae* O1 detection in estuarine and coastal zooplankton. *J. Plankton Res.* **33**, 51–62 (2010).
2. Barbera, A. La *et al.* Aislamiento de *Vibrio* spp. y evaluación de la condición sanitaria de los moluscos bivalvos *Arca zebra* y *Perna perna* procedentes de la costa nororiental del Edo, Sucre, Venezuela. *FCU-LUZ* **14**, 513–521 (2004).
3. Batabyal, P., Mookerjee, S., Einsporn, M. H., Lara, R. J. & Palit, A. Environmental drivers on seasonal abundance of riverine-estuarine *V. cholerae* in the Indian Sundarban mangrove. *Ecol. Indic.* **69**, 59–65 (2016).
4. Binsztein, N. *et al.* Viable but nonculturable *Vibrio cholerae* O1 in the aquatic environment of Argentina. *Appl Env. Microbiol* **70**, 7481–7486 (2004).
5. Bliem, R., Reischer, G., Linke, R., Farnleitner, A. & Kirschner, A. Spatiotemporal dynamics of *Vibrio cholerae* in turbid alkaline lakes as determined by quantitative PCR. *Appl. Environ. Microbiol.* **84**, 1–14 (2018).
6. Dalusi, L., Lyimo, T. J., Lugomela, C., Hosea, K. M. M. & Sjöling, S. Toxigenic *Vibrio cholerae* identified in estuaries of Tanzania using PCR techniques. *FEMS Microbiol. Lett.* **362**, fnv009 (2015).
7. de Menezes, F. G. R. *et al.* Detection of virulence genes in environmental strains of *Vibrio cholerae* from estuaries in northeaster Brazil. *Rev. Inst. Med. Trop. Sao Paulo* **56**, 427–432 (2014).
8. de Menezes, F. G. R. *et al.* Pathogenic *Vibrio* species isolated from estuarine environments (Ceará, Brazil) - antimicrobial resistance and virulence potential profiles. *An. Acad. Bras. Cienc.* **89**, 1175–1188 (2017).
9. Dheenan, P. S. *et al.* Spatial variation of physicochemical and bacteriological parameters elucidation with GIS in Rangat Bay, Middle Andaman, India. *J. Sea Res.* **85**, 534–541 (2014).
10. Di, D. Y. W., Lee, A., Jang, J., Han, D. & Hur, H. G. Season-specific occurrence of potentially pathogenic *Vibrio* spp. on the southern coast of South Korea. *Appl. Environ. Microbiol.* **83**, 1–13 (2017).
11. Escobar, L. E. *et al.* A global map of suitability for coastal *Vibrio cholerae* under current and

future climate conditions. *Acta Trop.* **149**, 202–211 (2015).
12. Esteves, K. *et al.* Rapid proliferation of *Vibrio parahaemolyticus*, *Vibrio vulnificus*, and *Vibrio cholerae* during freshwater flash floods in french mediterranean coastal lagoons. *Appl. Environ. Microbiol.* **81**, 7600–7609 (2015).
13. Esteves, K. *et al. Vibrio cholerae* during freshwater flash floods in french mediterranean coastal lagoons. *Front. Microbiol.* **81**, 7600–7609 (2015).
14. Fang, L., Ginn, A. M., Harper, J., Kane, A. S. & Wright, A. C. Survey and genetic characterization of Vibrio cholerae in Apalachicola Bay, Florida (2012–2014). *J. Appl. Microbiol.* **126**, 1265–1277 (2019).
15. Fernández-Delgado, M. *et al.* Occurrence and virulence properties of *Vibrio* and *Salinivibrio* isolates from tropical lagoons of the southern Caribbean Sea. *Antonie Van Leeuwenhoek* **110**, 833–841 (2017).
16. Fri, J., Ndip, R. N., Njom, H. A. & Clarke, A. M. Occurrence of virulence genes associated with human pathogenic vibrios isolated from two commercial Dusky Kob (*Argyrosmus japonicus*) farms and kareiga estuary in the Eastern Cape Province, South Africa. *International Journal of Environmental Research and Public Health* **14**, (2017).
17. Gardade, L. & Khandeparker, L. Spatio-temporal variations in pathogenic bacteria in the surface sediments of the Zuari estuary, Goa, India. *Curr. Sci.* **113**, 1729–1738 (2017).
18. Gdoura, M. *et al.* Molecular detection of the three major pathogenic vibrio species from seafood products and sediments in Tunisia using real-Time PCR. *J. Food Prot.* **79**, 2086–2094 (2016).
19. Grothen, D. C., Zach, S. J. & Davis, P. H. Detection of intestinal pathogens in river, shore, and drinking water in Lima, Peru. *J. Genomics* **5**, 4–11 (2017).
20. Gyraite, G., Katarzyte, M. & Schernewski, G. First findings of potentially human pathogenic bacteria *Vibrio* in the south-eastern Baltic Sea coastal and transitional bathing waters. *Mar. Pollut. Bull.* **149**, 110546 (2019).
21. Hackbusch, S., Wichels, A., Gimenez, L., Döpke, H. & Gerdts, G. Potentially human pathogenic *Vibrio* spp. in a coastal transect: Occurrence and multiple virulence factors. *Sci. Total Environ.* **707**, 136113 (2020).
22. Izumiya, H. *et al.* A double-quadratic model for predicting *Vibrio* species in water environments of Japan. *Arch. Microbiol.* **199**, 1293–1302 (2017).
23. Khamesipour, R. M., Rahimi, E. & Khodadoostan, A. Occurrence of *Vibrio* spp., Aeromonas hydrophila, *Escherichia coli* and *Campylobacter* spp. in crayfish (*Astacus leptodactylus*) from Iran. *Iran. J. Fish. Sci.* **13**, 944–954 (2014).
24. Kim, J. Y. & Lee, J. L. Multipurpose assessment for the quantification of *Vibrio* spp. and total bacteria in fish and seawater using multiplex real-time polymerase chain reaction. *Journal of the Science of Food and Agriculture* **94**, 2807–2817 (2014).
25. Kokashvili, T. *et al.* Occurrence and Diversity of clinically important *Vibrio* species in the aquatic environment of Georgia. *Front. Public Heal.* **3**, 1–12 (2015).
26. Lipp, E. K. E. *et al.* Direct detection of *Vibrio cholerae* and ctxA in Peruvian coastal water and plankton by PCR. *Appl. Env. Mircrobiol* **69**, 3676 (2003).
27. López, L. *et al.* Estudio piloto para el aislamiento de *Vibrio* spp. en ostras (*Crassostera rhizophorae*) capturadas en la Ciénaga de la Virgen, Cartagena, Colombia. *RSPYN* **11**, 1–6 (2010).
28. Louis, V. R. *et al.* Predictability of *Vibrio cholerae* in Chesapeake Bay. *Appl Env. Microbiol* **69**, 2773–2785 (2003).
29. Machado, A. & Bordalo, A. A. Detection and quantification of *Vibrio cholerae*, *Vibrio parahaemolyticus*, and *Vibrio vulnificus* in Coastal Waters of Guinea-Bissau (West Africa). *Ecohealth* **13**, 339–349 (2016).
30. Main, C. R., Salvitti, L. R., Whereat, E. B. & Coyne, K. J. Community-level and species-specific associations between phytoplankton and particle-associated *Vibrio* species in Delaware's Inland Bays. *Appl. Environ. Microbiol.* **81**, 5703–5713 (2015).
31. Matteucci, G., Schippa, S., Di Lallo, G., Migliore, L. & Thaller, M. C. Species diversity, spatial distribution, and virulence associated genes of culturable vibrios in a brackish coastal Mediterranean environment. *Ann. Microbiol.* **65**, 2311–2321 (2015).

32. Meena, B. *et al.* Studies on diversity of *Vibrio* sp. and the prevalence of hapA, tcpI, st, rtxA&C, acfB, hlyA, ctxA, ompU and toxR genes in environmental strains of *Vibrio cholerae* from Port Blair bays of South Andaman, India. *Marine Pollution Bulletin* 105–116 (2019). doi:10.1016/j.marpolbul.2019.05.011
33. Meyer, J. L., Gunasekera, S. P., Scott, R. M., Paul, V. J. & Teplitski, M. Microbiome shifts and the inhibition of quorum sensing by Black Band Disease cyanobacteria. *ISME J.* **10**, 1204–1216 (2016).
34. Ming, H. *et al.* Enterococci may not present the pollution of most enteric pathogenic bacteria in recreational seawaters of Xinghai bathing Beach, China. *Ecol. Indic.* **110**, 105938 (2020).
35. Mukhopadhyay, A. K. A. *et al.* Molecular epidemiology of reemergent *Vibrio cholerae* O139 Bengal in India. *J. Clin. Microbiol.* **36**, 2149–2152 (1998).
36. Neogi, S. B. *et al.* Environmental and hydroclimatic factors influencing *Vibrio* populations in the estuarine zone of the Bengal delta. *Environ. Monit. Assess.* **190**, 565 (2018).
37. Orozco, R. *et al. Evaluación de la constaminación y calidad microbiológica del agua de mar en las bahías de Ferrol y Samanco*. **56**, (Instituto del Mar del Peru, 1996).
38. Pal, B. B., Khuntia, H. K., Samal, S. K., Das, S. S. & Chhotray, G. P. Emergence of *Vibrio cholerae* O1 biotype El Tor serotype Inaba causing outbreaks of cholera in Orissa, India. *Jpn J infect Dis* **59**, 266–9 (2006).
39. Pascual, M., Rodó, X., Ellner, S. P., Colwell, R. R. & Bouma, M. J. Cholera dynamics and El Nino-Southern Oscillation. *Science* **289**, 1766–1769 (2000).
40. Perkins, T. L. *et al.* Sediment composition influences spatial variation in the abundance of human pathogen indicator bacteria within an estuarine environment. *PLoS ONE* **9**, e112951 (2014).
41. Sack, R. B. *et al.* A 4-year study of the epidemiology of *Vibrio cholerae* in four rural areas of Bangladesh. *J. Infect. Dis.* **21205**, 96–101 (2003).
42. Siboni, N., Balaraju, V., Carney, R., Labbate, M. & Seymour, J. R. Spatiotemporal dynamics of *Vibrio* spp. within the Sydney harbour estuary. *Front. Microbiol.* **7**, 460 (2016).
43. Silva, M. M. *et al.* Dispersal of potentially pathogenic bacteria by plastic debris in Guanabara Bay, RJ, Brazil. *Mar. Pollut. Bull.* **141**, 561–568 (2019).
44. Sneha, K. G. *et al.* Distribution of multiple antibiotic resistant *Vibrio* spp across Palk Bay. *Reg. Stud. Mar. Sci.* **3**, 242–250 (2016).
45. Sulca, M. A., Orozco, R. & Alvarado, D. E. Antimicrobial resistance not related to 1,2,3 integrons and Superintegron in *Vibrio* spp. isolated from seawater sample of Lima (Peru). *Mar. Pollut. Bull.* **131**, 370–377 (2018).
46. Wong, Y. Y. *et al.* Environmental control of *Vibrio* spp. abundance and community structure in tropical waters. *FEMS Microbiol. Ecol.* **95**, fiz176 (2019).
47. Xu, M., Kan, B. & Wang, D. Identifying environmental risk factors of cholera in a coastal area with geospatial technologies. *Int. J. Environ. Res. Public Health* **12**, 354–370 (2015).
48. Yue, Y. *et al.* Influence of climate factors on *Vibrio cholerae* dynamics in the Pearl River estuary, South China. *World J. Microbiol. Biotechnol.* **30**, 1797–1808 (2014).
49. Zaw, M. T. *et al.* Genetic diversity of toxigenic *Vibrio cholerae* O1 from Sabah, Malaysia 2015. *J. Microbiol. Immunol. Infect.* **52**, 563–570 (2019).
50. Zhang, X., Song, Y., Liu, D., Keesing, J. K. & Gong, J. Macroalgal blooms favor heterotrophic diazotrophic bacteria in nitrogen-rich and phosphorus-limited coastal surface waters in the Yellow Sea. *Estuar. Coast. Shelf Sci.* **163**, 75–81 (2015).

Data

1. Satellite-derived sea surface temperature, chlorophyll-*a*, and *V. cholerae* data in global exclusive economic zones (i.e., 200 miles off the coast of each country).[149]
2. Population data from the NASA Socioeconomic Data and Applications Center (SEDAC) Gridded Population of the World (GPWv4).[2]

Caveats

This coarse-scale assessment was able to generate a global-level reconstruction of *V. cholerae* potential distribution in coastal waters. Coarse-scale models, however, could fail to capture fine-scale phenomena. For example, *V. cholerae* bacteria abundance and distribution could be influenced by the presence and composition of zooplankton species,[150] which is information not available for global coastal assessments.

Additional analysis

Results indicate that most regions witnessed an increase in their suitability for *V. cholerae* (Figure 40), a consistent global trend to increase *V. cholerae* transmission risk. The change in coastal areas suitable for *V. cholerae* was assessed along a gradient of human population across global coastal areas. In terms of WHO regions, there was a strong signal for the past five years in the African, American, and Western Mediterranean regions (Figure 41), revealing that populations exposed to coastal *V. cholerae* transmission have increased in the past 18 years (Figure 42). Countries with significant increase in coastal areas suitable for *V. cholerae* growth were grouped according to the human development index (HDI). Strong and significant increase in coastal populations exposed to *V. cholerae* transmission risk was observed in countries with high (r^2=0.95, p=0.004), medium (r^2=0.88, p=0.017), and low (r^2=0.98, p=0.002) HDI, while countries with very-high HDI also showed increase in populations living in areas suitable for coastal *V. cholerae*, although increases were not significant (r^2=0.57, p=0.13). The increase of coastal populations living in areas suitable for *V. cholerae* in the 2003-2020 period was 29% in very-high HDI countries, 56% in high, 63% in medium, and 116% in low HDI countries. The global increase of areas suitable for *V. cholerae* growth correlated with human populations exposed to these areas, especially in high HDI countries (Figure 43).

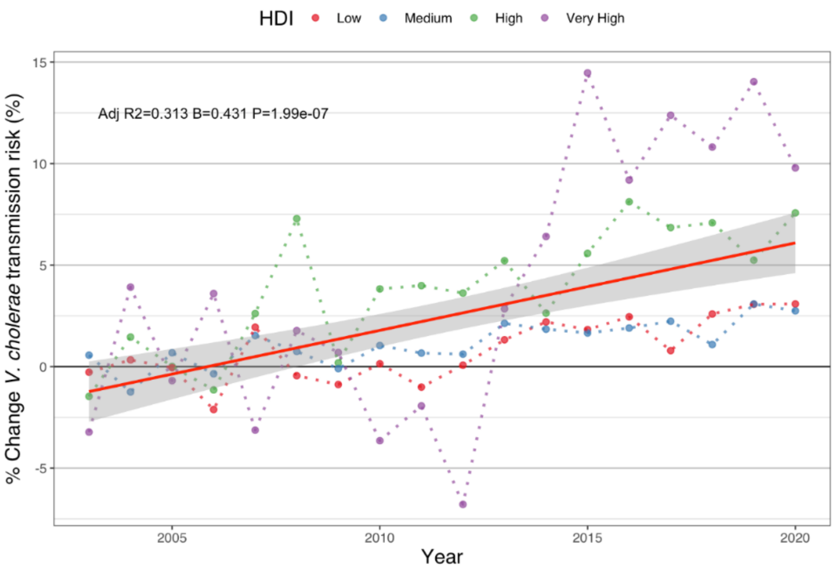

Figure 40. Assessment of percentage change in areas suitable for V. cholerae presence and multiplication in coastal waters globally. Global assessment of coastal areas environmentally suitable for V. cholerae

growth (red line) compared with a baseline of values during the 2003–2005 period. Colours denote indicated HDI levels.

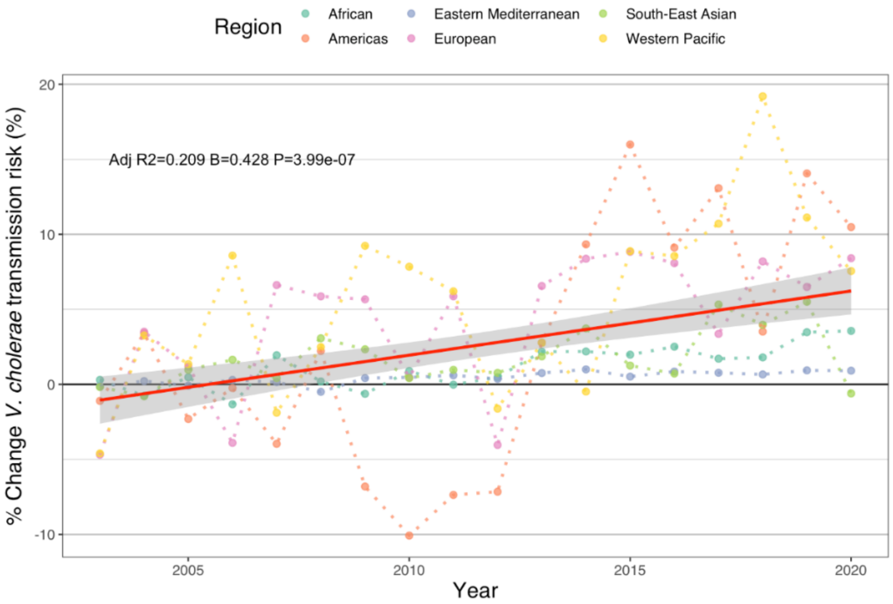

Figure 41. Assessment of percentage change in areas suitable for V. cholerae presence and multiplication in coastal waters globally. Global assessment of coastal areas environmentally suitable for V. cholerae growth (red line) compared with a baseline of values during the 2003–2005 period. Colours denote WHO regions as indicated.

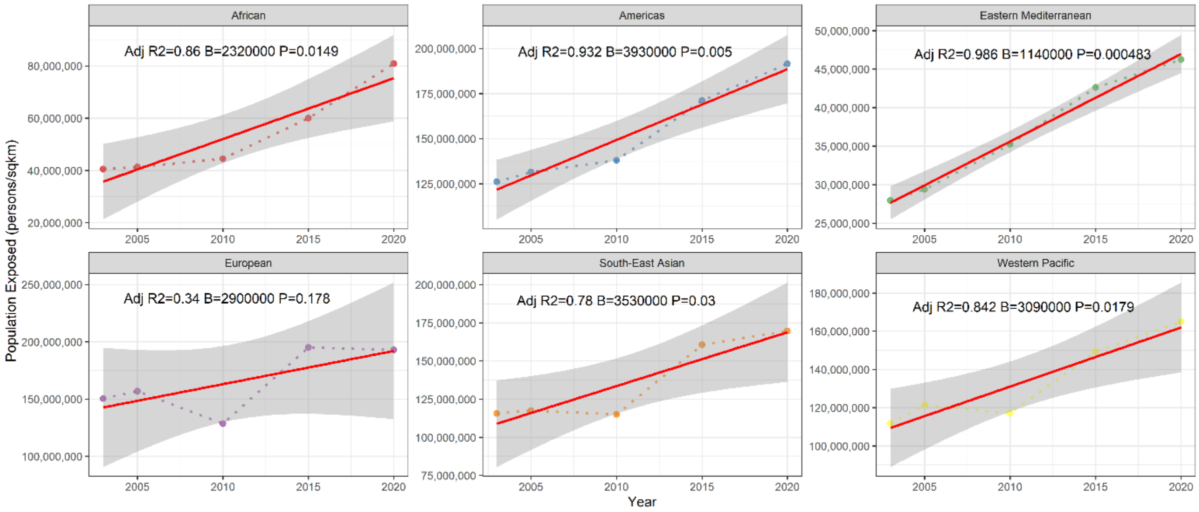

Figure 42. Changes in human population exposure as a function of time from 2003 to 2020. Significant changes were found across time for African (r^2=0.86, p=0.014), Americas (r^2=0.93, p=0.005), Eastern

Mediterranean (r^2=0.98, p<0.001), South-East Asian (r^2=0.78, p=0.029), and Western Pacific (r^2=0.84, p=0.017) region, but not significance was detected for the European region (r^2=0.34, p=0.178).

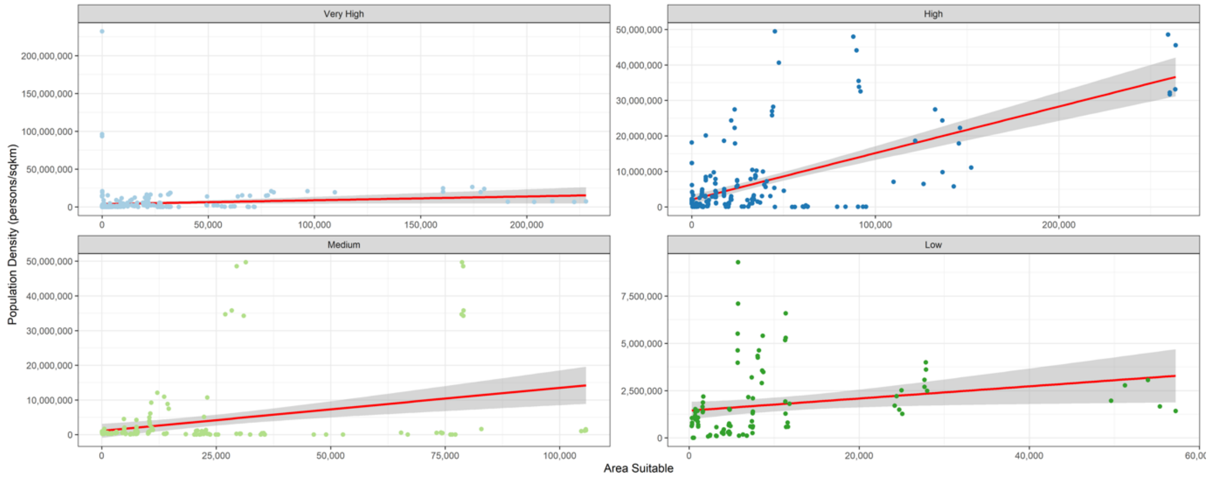

Figure 43. Changes in human population exposure as a function of coastal areas suitable for V. cholerae (V. cholerae transmission risk) from 2003 to 2020. Significant changes were found for global coastal areas (r^2=0.1, p<0.001). When countries were grouped by HDI, significant increase was detected for countries with high (r^2=0.38, p<0.001), medium (r^2=0.10, p<0.001), and low (r^2=0.05, p<0.001) HDI, but not for very-high HDI countries (r^2=0.01, p=0.055).

1.4: Food Security and Undernutrition

This indicator consists of three sub-indicators; the first tracks the change in crop growth duration as a proxy for change in potential crop yield; the second tracks risks to marine food security by monitoring changes in sea surface temperature and the consumption of farmed- or catch-based fish products. The third sub-indicator tracks the impact of climate change and income on the incidence of food insecurity.

Crop yield potential

Methods

The methodology remains similar to that described in the 2021 report of the *Lancet* Countdown.[11] Briefly, crop duration is defined as the time taken in a year to accumulate the reference period (1981-2010) average growing season accumulated temperature total (ATT).[151] The crop duration loss is defined as the percentage change in the time taken (in days) to accumulate the average growing season accumulated temperature.

Crop yield potential is calculated across the area of land under cultivation[152] at 0.25° x 0.25°, and then area-weighted averaged. Climate data are taken from the monthly historical records from ECMWF ERA5 climate reanalysis dataset between January 1980 and December 2021, and synthetic daily data are estimated for each grid cell by applying a regional average daily anomaly to the monthly value. The plot shows the global average annual change in crop growth duration. The horizontal line shows the average difference in crop growth duration over the reference period 1981–2010 (

Figure 44).

Data

1. Climate data from the European Centre for Medium-Range Weather Forecasts (ECMWF) ERA5 reanalysis[9]

Caveats

While reduction in crop growth duration due to higher temperatures during the growing season is usually associated with a reduction in potential yield, the precise relationship depends on location and crop. Potential crop yield is the yield that could be achieved with no limitations on water or nutrients, and in some areas reliant on rainfall variations in water availability are more important than variations in temperature.

Additional analysis

Growth duration is decreasing across all crops monitored (

Figure 44) trends are similar overall. Very high HDI countries witnessed the highest decrease of growth duration for maize and rice crops, and for spring wheat alongside low HDI countries. The growth duration of winter wheat has decreased in low and high HDI countries the most (Figure 45). The WHO classification provides further insights with the highest decreases in growth duration observed in the European region for maize and rice growth, in the African region for winter wheat and in the region of the Americas for spring wheat (Figure 46). It is worth noting the year-to-year variability which is due to the wide range of geographical/climatic conditions covered in each HDI level / WHO region.

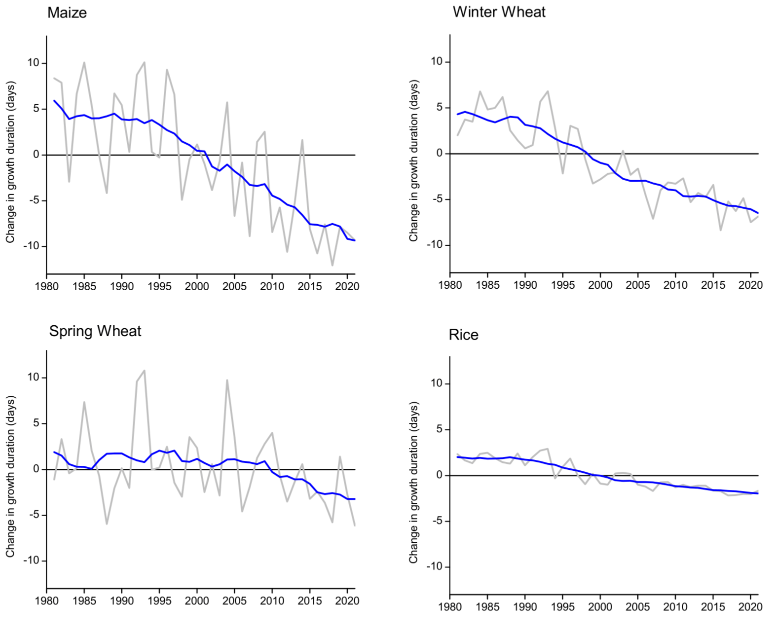

Figure 44: Change in crop growth duration relative to the 1981–2010 global average. The red line represents the annual global area-weighted change in crop growth duration. The blue line represents the running mean of change in crop growth duration over 11 years (5 years before and 5 years after).

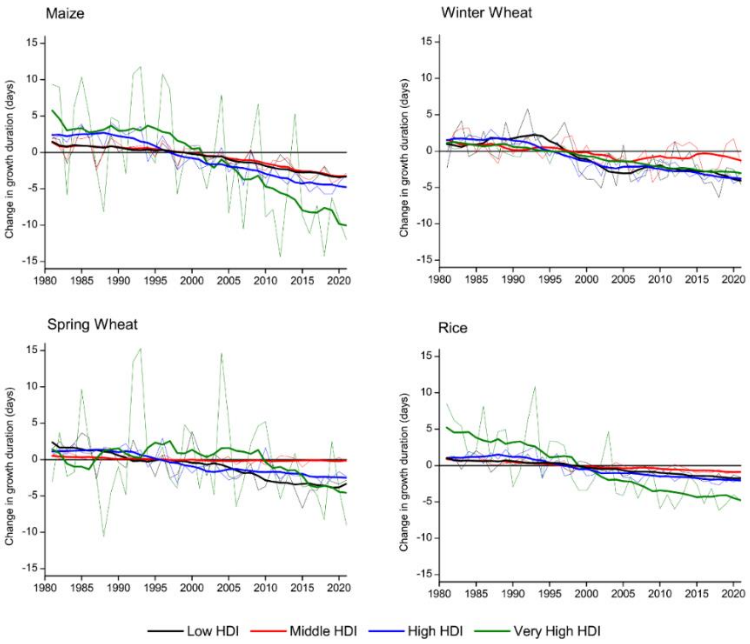

Figure 45. Change in crop growth duration relative to the 1981–2010 average by HDI level. Thinner lines represent the annual global area-weighted change in crop growth duration. Ticker lines represent the running mean of change in crop growth duration over 11 years (5 years before and 5 years after).

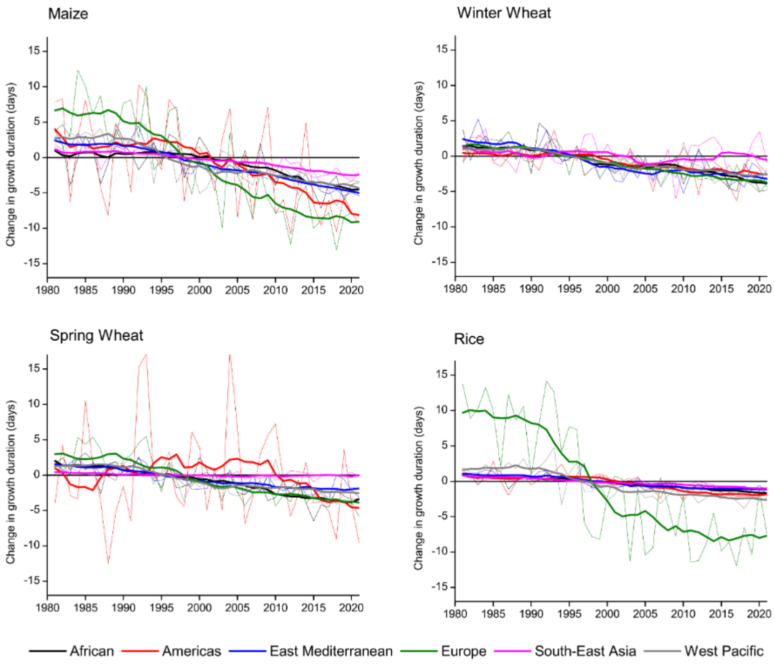

Figure 46. Change in crop growth duration relative to the 1981–2010 global average by WHO region. Thinner lines represent the annual global area-weighted change in crop growth duration. Ticker lines reepresent the running mean of change in crop growth duration over 11 years (5 years before and 5 years after).

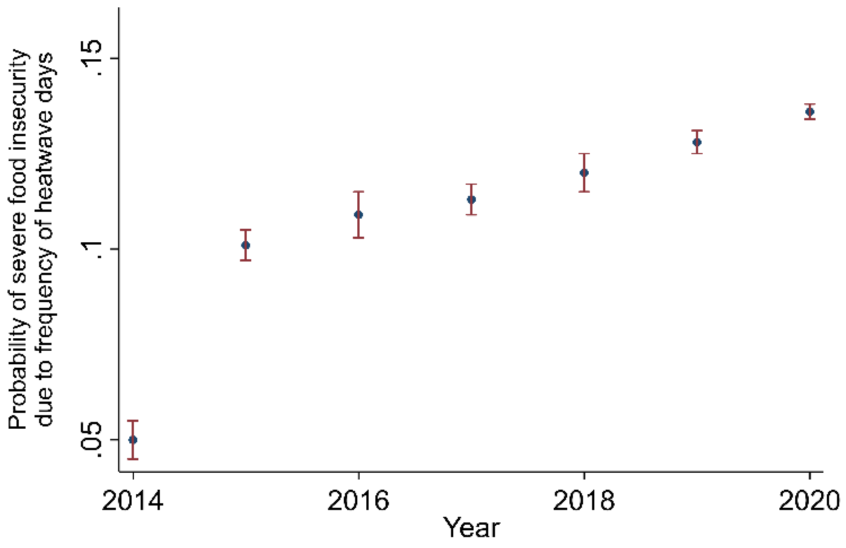

Figure 47. Impact of an increase in the number of heatwave days during the four major crop (maize, rice, sorghum, and wheat) growing seasons on severe food insecurity (percentage-points) using a time-varying regression.

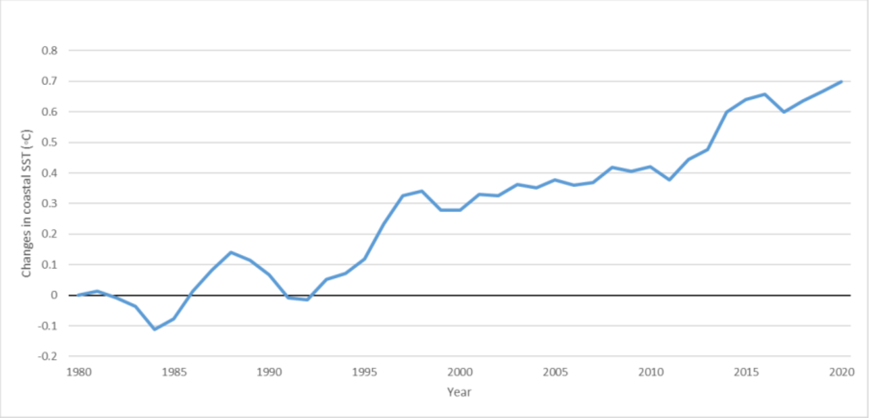

Figure 48: Changes in the 3-year moving average of the global coastal sea surface temperature (SST) compared to 1980

Marine Food productivity

Methods

The methodology for this indicator applies to a wider geographical area and more countries compared with the 2021 Lancet Countdown report.[11] Sixteen major FAO (Food and Agriculture Organization of the United Nations) marine basins and two inland water basins which are important in terms of projected impacts and vulnerabilities associated with climate change were selected. One-hundred forty-two countries located in these basins were chosen to assess changes in sea surface temperature (SST), as well as the deterioration of major coral reef sites and the decreased consumption of capture-based fish.

The input data for this indicator have been improved and extended from the 2021 report. New to 2022, SST data were retrieved from the ORAS5 global ocean reanalysis dataset from 1980 to 2021. A total number of 717,090 grid cells were used to have an accurate estimate for SST variations.

Moreover, the data concerning capture-based and farmed-based per capita fish consumption in the investigated countries from 1980 to 2019 were collected and analysed. Disability-adjusted life years (DALYs) attributed to

diet low in seafood ω3 by the Global Burden of Diseases[153] was provided based on both WHO regions and HDI (Human Development Index) levels.

Data

1. Ocean data from the European Centre for Medium-Range Weather Forecasts (ECMWF) ORAS5 reanalysis.[154]
2. Annual maximum bleaching alert area caused by thermal stress from NOAA Coral Reef Watch Zones, 1985-2021.[155]
3. Capture-based and farmed-based fish consumption per capita from FAO, 1980-2019.[156]
4. Attributable DALYs to diet low in seafood ω3 from GBD, 1990–2019[154]

Caveats

There is a lack of information and data in the available databases such as FAO on fish species composition of the captured and farmed fish products. This could, in turn, lead to some concerns about the methodological approach used to calculate ω3 intake. More specifically, most of the approaches are based on fish intake, which usually ignores or underestimates variations in ω3 contents of different types of fishes, and especially capture-based compared with farmed-based fish. It should also be highlighted that GBD estimates for the association between this dietary risk factor and cardiovascular diseases, as the primary reference for human health impacts, are not based on type and source of seafood products either.

DALY estimates are extracted from the Global Burden of Disease (GBD) study. GBD study uses disease modelling approaches to estimate burden for each disease and attributable burden for each of the risk factors. Due to limited access to high-quality data in many of the countries, especially low or middle- income countries, the estimates might be different from the real situation. On the other hand, GBD study does not differentiate aquaculture and capture-based sources for estimating ω-3 intake.

Fish production data were used as a surrogate for fish consumption. This is not a completely accurate assumption, but there is no comprehensive alternative source of data for all the investigated countries.

Future form of the indicator

Further analysis will be required to connect the different components of the causality chain, i.e., between SST and health impacts. Also, the data revealing the impacts of COVID-19 are expected to be available soon and will be taken into account and included in the analysis.

Additional analysis

The European region and countries with very high HDI still have the lowest average SST, but they have experienced the largest amount of increase in the period between 1980 and 2019 (Table 15). Despite a general increase in per capita fish consumption globally, the share of marine capture-based in total fish consumption has continued decreasing in 2019 (Figure 49). The increasing sea surface temperature well supports the decline in marine capture and the consequent thermal stress-induced deteriorating coral bleaching (Figure 50, Figure 51).

Figure 52 shows the Disability-Adjusted Life Years (DALYs) attributed to diet low in ω-3 fatty acids (per 100,000 population) for all countries with an access to open sea. DALY estimates have been almost constant since 1990 for all ages, and slightly decreased after age standardisation. However, the trends are different for countries in different WHO regions and different levels of HDI. After age-standardisation, the trends for European, Americas, and Eastern Mediterranean regions were decreasing, while they were almost constant or even increasing in other regions (Figure 53). Stratified analysis based on the levels of human development index (HDI) shows a sharper decrease in the countries with very high HDI, compared to other strata of HDI (Figure 54).

	Weight: number of data points				Weight: Number of countries			
	N (data points)	Difference	Mean SST 1980 (◦C)	Mean SST 2021 (◦C)	N (countries)	Difference	Mean SST 1980 (◦C)	Mean SST 2021 (◦C)
WHO regions								
African	53,874	0.5	24.3	24.7	29	0.6	25.5	26.1
Americas	208,608	0.5	17.2	17.6	31	0.4	24.7	24.9
Eastern Mediterranean	43,542	0.6	24.5	25.2	16	0.7	24.8	25.6
European	148,338	1.2	9.5	10.7	30	1.4	12.7	14.2
South-East Asian	86,100	0.4	28.7	29.1	8	0.3	28.6	28.9
Western Pacific	158,670	0.7	24.2	24.8	21	0.6	26.3	26.8
Levels of Human Development								
Low	27,552	0.4	27.0	27.4	17	0.4	26.9	27.4
Medium	84,624	0.5	26.8	27.3	29	0.5	26.5	27.3
High	225,582	0.6	25.8	26.3	38	0.6	25.2	26.3
Very High	356,946	0.8	13.2	14.1	48	1.0	16.6	14.1

Table 15. Sea Surface Temperature (SST) in 1980 and 2019, and changes in 3-year moving average of SST (°C) for the coastal waters by WHO region and HDI levels. The column "Difference" indicates the difference between the 3-year average of SST for the 2019–2021 and the 1980–1982 periods.

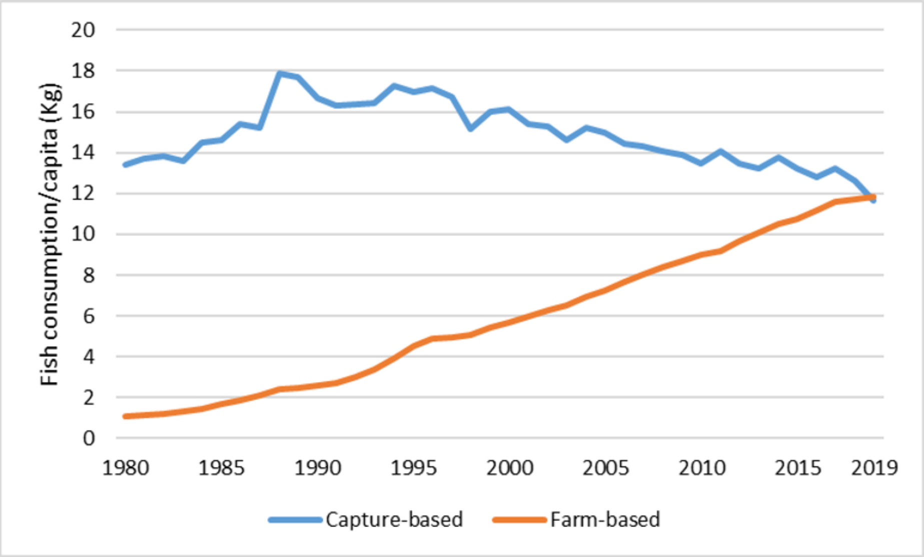

Figure 49. Population weighted average fish consumption per capita in 142 investigated countries/territories, separated by the origin of fish (marine capture-based and farm-based) from 1980 to 2019.

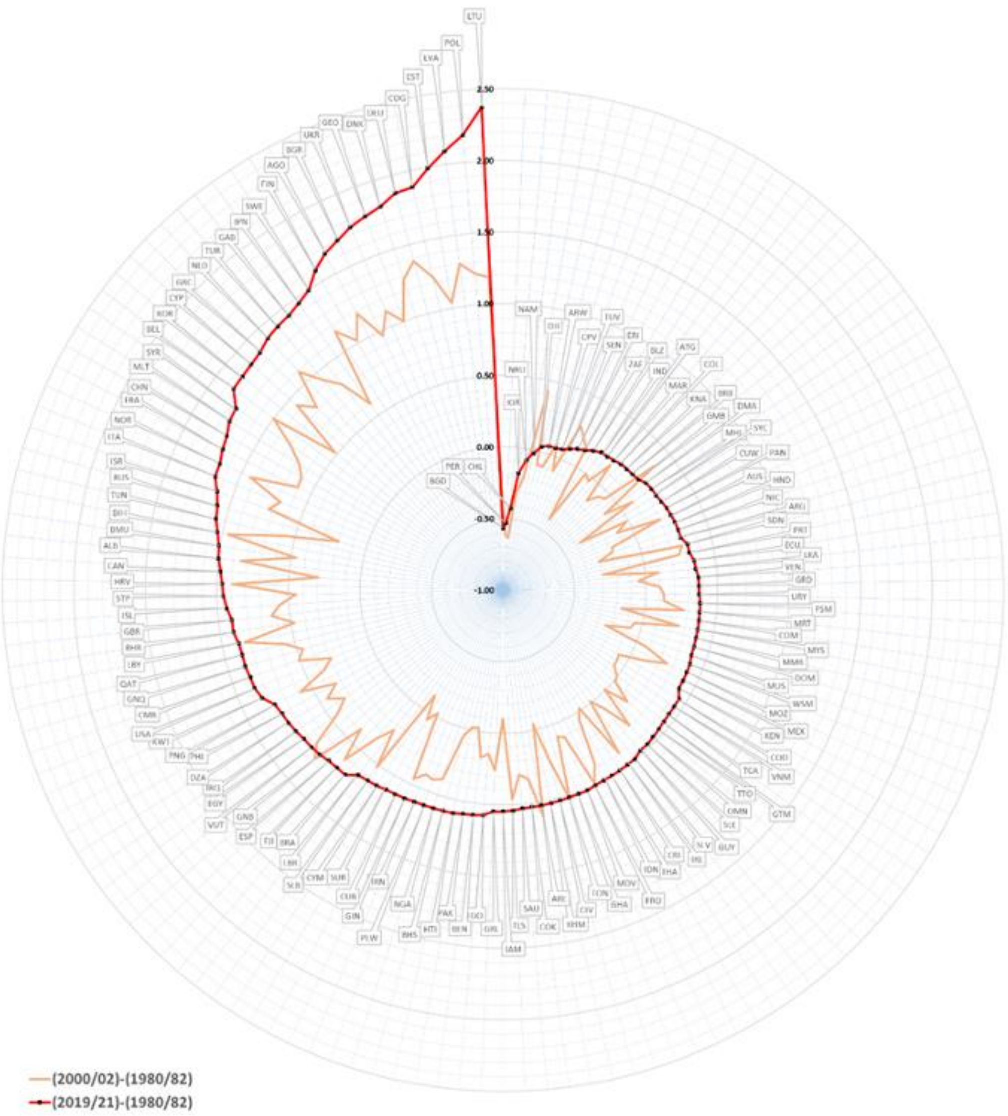

Figure 50. Changes in 3-year moving average sea surface temperature (ºC) for the coastal waters of 142 countries/territories: 2000–2002 compared to 1980–1982 and 2019–2021 compared to 1980–1982. Source: Ocean Reanalysis System 5 (ORAS5).[154]

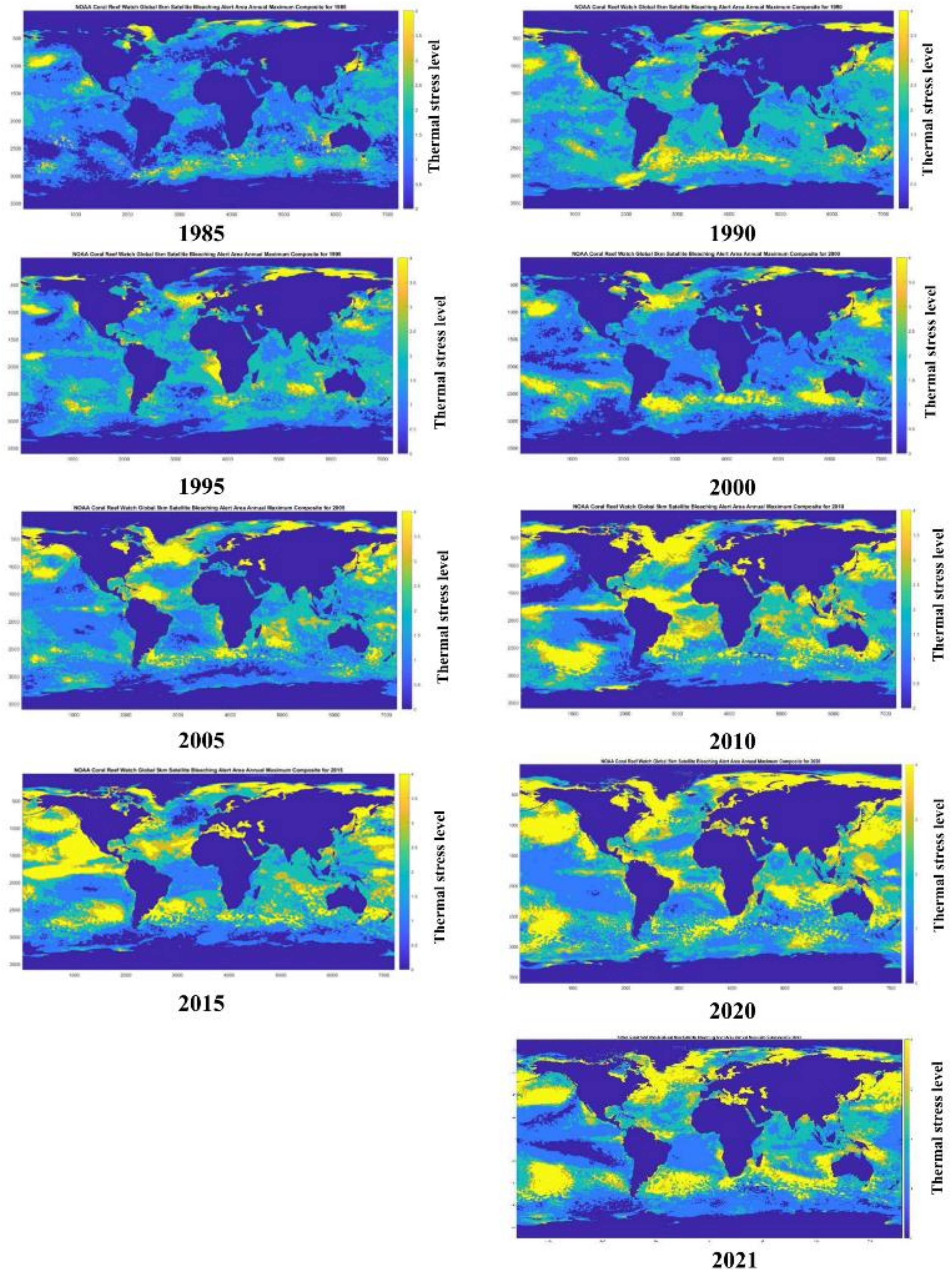

Figure 51. Comparing annual maximum Bleaching Alert Area caused by thermal stress in five-year intervals (1985–2020) and in 2021. Source: NOAA Coral Reef Watch. 1985, updated daily. NOAA Coral Reef Watch Global 5km Satellite Bleaching Alert Area Annual Maximum Composite Version 3.1, Jan. 01,

2020–Jan. 01, 2021. College Park, Maryland, USA: NOAA Coral Reef Watch. Data set accessed January 2022.[157]

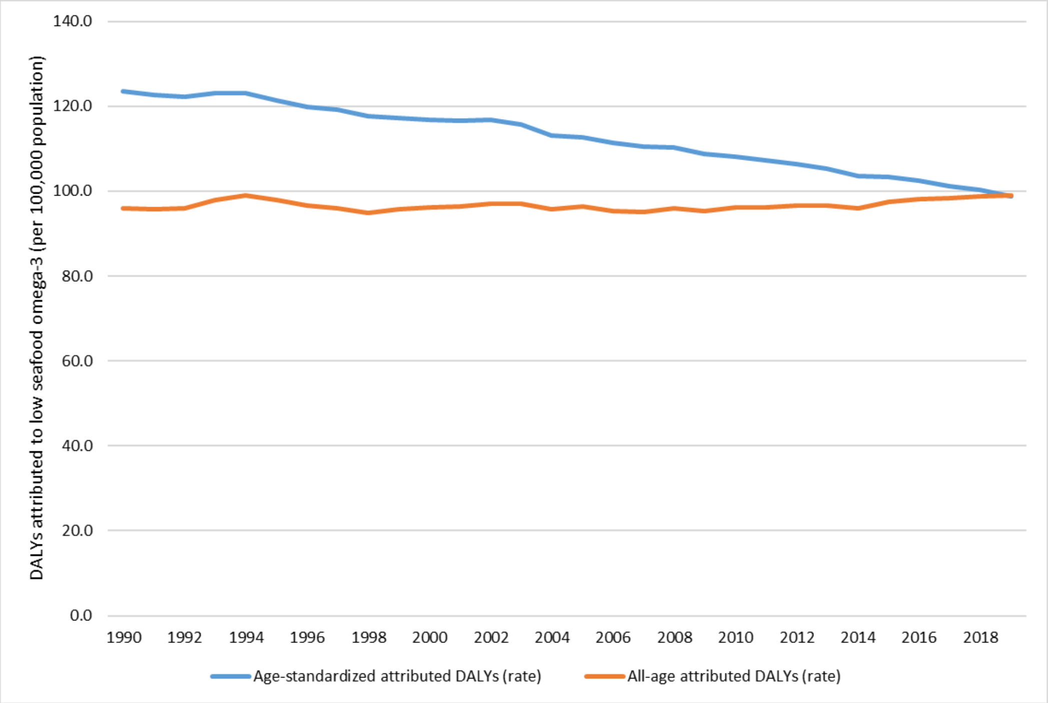

Figure 52. Disability Adjusted Life Years (DALYs) attributable to diet low in seafood omega-3 fatty acids in countries with an access to open sea in the 1990–2019 period.

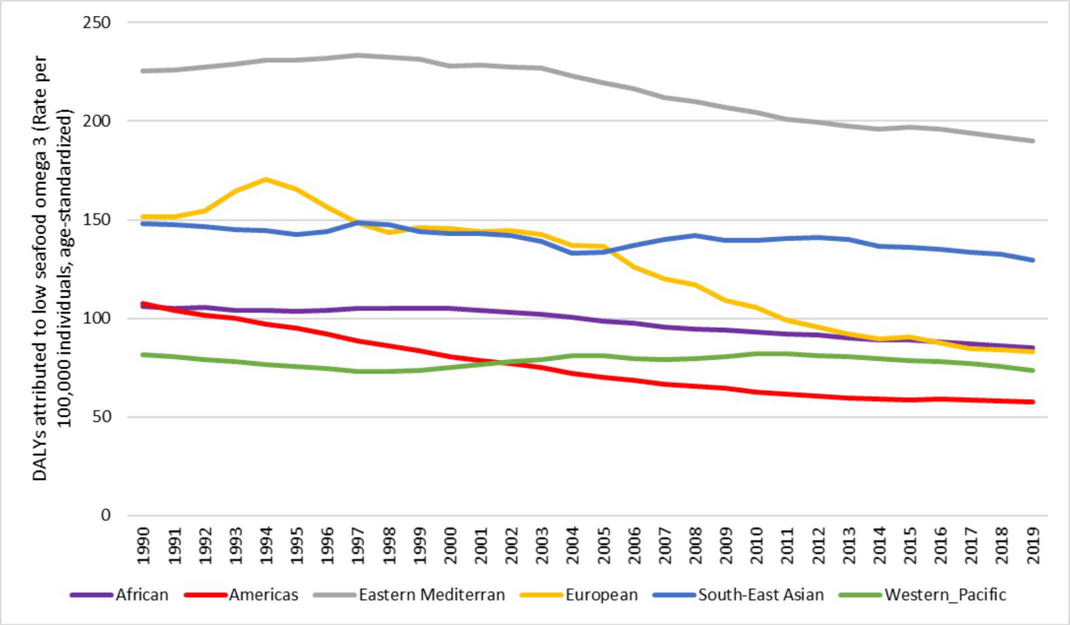

Figure 53. Regional differences in Disability Adjusted Life Years (DALYs) attributable to diet low in seafood ω-3 fatty acids in countries with an access to open sea, age-standardised, in the 1990–2019 period.

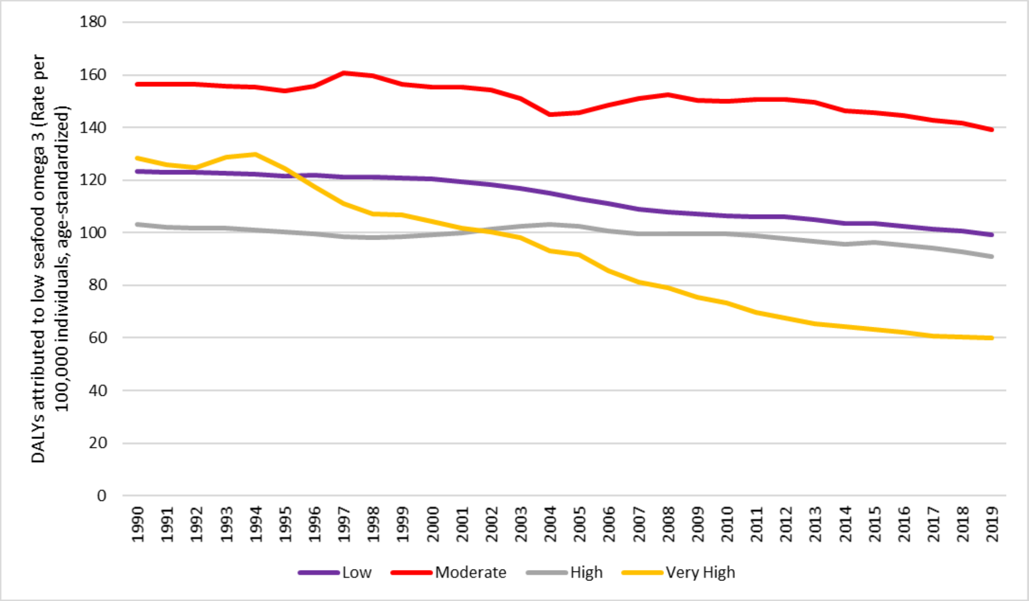

Figure 54. Stratified trends of Disability Adjusted Life Years (DALYs) attributable to diet low in seafood ω-3 fatty acids based on Human Development Index (HDI) in countries with an access to open sea, age-standardised, in the 1990–2019 period.

Food insecurity

Methods

The methodology of this indicator is based on previously published models.[158] To track the impact of climate change and income on the incidence of food insecurity, it uses a panel data regression with coefficients that vary over time. To operationalise the concept of climate change, it focuses on the number of heatwave days during the four major crop growing seasons in each region.[159] A heatwave is defined as a period of at least two days where both the daily minimum and maximum temperatures are above the 95th percentile of the respective climate (indicator 1.1.2) in each region. The gridded 95th percentile of daily minimum and maximum temperatures, taken from the ERA5-Land hourly dataset, [160] were calculated for 1986–2005. The indicator uses the lagged number of heatwaves during the crop growing seasons for each year during 2014–2020.

Increase in the number of heatwave days can affect food insecurity through multiple pathways, including through the impacts of heat stress on crop yields, on agricultural and non-agricultural labour (therefore on crop production and income), on health, on food prices, and on food supply chains. The regression also includes twelve-month Standardized Precipitation Evapotranspiration Index (SPEI) as a measure of drought. SPEI-12 was computed using precipitation data from ERA5-Land monthly averaged dataset and the SPEI package in R.[160,161]

Two dependent variables are used: first, the probability of moderate to severe food insecurity; and second the probability of severe food insecurity; both at the sub-national level. To account for unobserved heterogeneity such as differences in food and storage policies across countries and changes in the prices of food items from year to year, this specification also includes both location and time (year) fixed-effects. The standard errors are clustered at the country-level.[162] The panel data specification can be written as follows:

$$FIES_{it} = \beta_1(\tau_t) + V_{(it)} + \gamma'(\tau_t)X_{(it)} + \alpha_{(i)} + \mu_{(it)}$$

where $FIES_{it}$ is the probability of moderate to severe food insecurity or probability of severe food insecurity, $\boldsymbol{V}_{it}$ is the change in the number of heatwave days during the four major crop growing season, and $\boldsymbol{X}_{it}$ is a vector of relevant variables affecting food insecurity - income, droughts, a dummy to control for the COVID-19 pandemic in 2020. $\boldsymbol{\mu}_{it}$ is a random error term. All variables are recorded for different locations with index $i = 1, \dots, N$ and over a number of years $t = 1, \dots, T$. The time-varying coefficients allow us to examine whether the relationship between temperature anomaly and food insecurity has evolved over time.

In the second-step, a counterfactual analysis is conducted, to explore the extent to which food insecurity may have been affected by climate change.[158] To do this, the cumulative impacts of increasing frequency of heatwaves above the historical norms over the period 1981–2010 are computed. The counterfactual impact of climate change on food insecurity is derived by combining the coefficients from the time-varying regression with the historical norm average on each year for which food security data is available. The effects of increases in the frequency of heatwaves compared to the baseline (1981–2010) under which frequency of heatwaves increases according to its historical trend are then considered.

Data

1. Hourly climate data (2m air temperature) from the European Centre for Medium-Range Weather Forecasts (ECMWF) ERA5-Land reanalysis.[160]
2. Monthly climate data (total precipitation) from ECMWF ERA5-Land reanalysis.[123]
3. Food insecurity from the FAO Food Insecurity Experience Scale[163]

Caveats

The main caveat for temperature anomaly food insecurity indicator is the possible recall bias in the survey data and the bias that may have been induced to interviews during the pandemic being conducted by phone instead of in-person visits. Once data for 2021 and 2022 become available, it will be assessed whether adjustments need to be made.

Future form of the indicator

In the future, disaggregated analysis will be provided by income groups.

Additional analysis

Due to an increase in the number of heatwave days, global severe food insecurity increased by 0·05 percentage-points in (95% CI 0·045–0·55; p<0·001) in 2014 and 0·136 percentage-points (0·134–0·138; <0·001) in 2020, implying an increasing trajectory. Findings also suggest that droughts (SPEI) increased the incidences of food insecurity while households in the lower-income groups had a higher probability of food insecurity. Finally, the pandemic in 2020 also increased the incidences of food insecurity (Figure 47, **Table 16**).

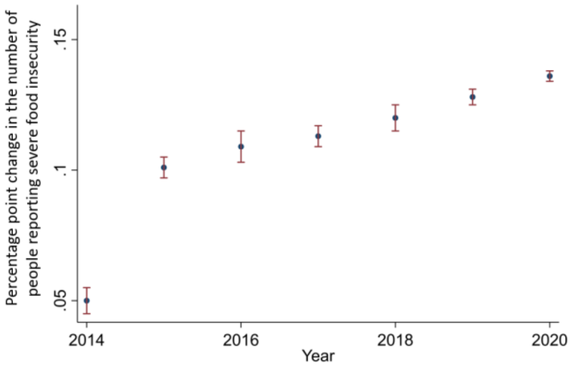

Figure 55: Impact of an increase in the number of heatwave days during the four major crop (maize, rice, sorghum, and wheat) growing seasons on severe food insecurity (percentage-points) using a time-varying regression.

		Moderate to severe	Severe
Low income		0·175	0·144
		(0·170, 0·180)	(0·140, 0·148)
High income		-0·121	-0·116
		(-0·119, -0·125)	(-0·101, -0·131)
Drought (SPEI-12)		0·017	0·012
		(0·015, 0·019)	(0·009, 0·015)
COVID-19 dummy		0·113	0·085
		(0·109, 0·117)	(0·080, 0·090)
Heatwave frequency($_t$)			
	2014	0·090	0·050
		(0·070, 0·110)	(0·045, 0·055)
	2015	0·106	0·101
		(0·096, 0·116)	(0·097, 0·105)
	2016	0·115	0·109
		(0·110, 0·120)	(0·103, 0·115)
	2017	0·124	0·113
		(0·120, 0·128)	(0·109, 0·113)
	2018	0·131	0·120
		(0·124, 0·138)	(0·115, 0·125)
	2019	0·14	0·128
		(0·135, 0·145)	(0·125, 0·131)
	2020	0·152	0·136
		(0·145, 0·159)	(0·134, 0·138)

Table 16: Relationship between heatwave frequency and food insecurity during 2014–2020 using a time-varying regression. 95% confidence intervals in parentheses.

Section 2: Adaptation, Planning, and Resilience for Health

2.1: Assessment and Planning of Health Adaptation

Indicator 2.1.1: National Assessments of Climate Change Impacts, Vulnerability and Adaptation for Health

Methods

The collection of data for this exercise included a voluntary national survey, the WHO Health and Climate Change Global Survey (2021) that was sent to all WHO Member States and a small number of non-Member territories. The survey was completed by the Ministry of Health focal points. Of the 194 WHO member states and non-Member territories, 95 participated in the survey, providing representation from all six WHO regions.

Survey participation has grown substantially from the 40 Member States that completed the 2015 WHO Health and Climate Change Global Survey. The survey was planned to be conducted every two years, although global circumstances have resulted in a three-year gap between surveys.

Validation of the 2021 country reported data was undertaken in multiple steps. First, survey responses were reviewed for missing information or inconsistencies with follow-up questions directed to survey respondents. A summary of responses was shared with WHO regional focal points and key informants for review, comments, and validation. Source documents including national health strategies and plans, and climate change and health vulnerability and adaptation assessments were collected. A desktop review of these source documents was conducted to compare with survey results with follow-up to survey respondents to seek clarification or additional documentation. Findings were also cross referenced with existing external publications. Data detailing all the ministries, institutions and national stakeholders that provided contributions to or review of the survey responses were collected in order to provide insight into the national consultation process of each survey submission. Of the 95 country submissions, 69 surveys were completed in consultation with one to six different stakeholders, ministries, or institutions. Five countries consulted between 10 to 12 stakeholders, ministries, or institutions. 15 countries did not consult with other entities or health programmes. Information was not available for the remaining six countries. Finally, all respondents reviewed and acknowledged the WHO data policy statement on the use and sharing of data collected by WHO in Member States outside the context of public health emergencies.

Of note, due to the ongoing pandemic, the standard data collection procedures were modified to reduce reporting burden on countries that wished to participate in the global survey but that were facing human resource constraints due to pandemic response. In eight cases, WHO prepared pre-filled survey questionnaires with data provided by ministries of health in the previous 2018 survey cycle or using data the countries had published in the 2020/2021 WHO UNFCCC health and climate change country profile when available. These countries were requested to review, revise, and complete the hard copy questionnaires. These hard copy questionnaires were then entered into the online platform by WHO. The same data validation steps as described above were then followed. Additionally, a number of countries requested an extension of the reporting period. As such, there may be a slight increase in the total number of participating countries and the WHO Health and Climate Change Global Survey Report and associated dynamic data dashboard will provide the definitive summary of findings.

Further information on the WHO Health and Climate Change Global Survey, its methodology, and the WHO UNFCCC Health and Climate Change Country Profile Project can be found at https://www.who.int/activities/monitoring-science-and-evidence-on-climate-change-and-health/health-and-climate-global-survey

The WHO questionnaire asks countries whether they have conducted a climate change and health vulnerability and adaptation assessment, defined as “a process and a tool that allows countries to evaluate which populations are most vulnerable to different kinds of health effects from climate change, to identify weaknesses in the systems that should protect them, and to specify interventions to respond. Assessments can also improve

evidence and understanding of the linkages between climate and health within the assessment area, serve as a baseline analysis against which changes in disease risk and protective measures can be monitored, provide the opportunity for building capacity, and strengthen the case for investment in health protection"

Information is collected on:

- The level of coverage of the assessment (Options: "national", "subnational", "other" or "unknown")
- Population groups considered in the assessment (Options: "Children" "Displaced or migrant populations", "The elderly (65+ years of age)", "Indigenous groups", "Populations living in poverty", "Women", "Workers", "Rural populations", "Urban/peri-urban populations", "Unknown", "Other")
- Whether the results of the assessment resulted in the development of new health policies or programs or the revision of existing health policies and/or programs (Options: "No", "Minimally", "Moderately", "Strongly", "Very strongly", "Unknown")
- Whether the results of the assessment influenced the allocation of human and financial resources within the Ministry of Health to address health risks of climate change (Options: "No", "Minimally", "Moderately", "Strongly", "Very strongly", "Unknown")

More information on climate change and health vulnerability and adaptation assessments, can be found in: https://www.who.int/teams/environment-climate-change-and-health/climate-change-and-health/capacity-building/toolkit-on-climate-change-and-health/vulnerability

Data

1. 2021 WHO Health and Climate Change Global Survey. [164]

Caveats

The survey sample is not a representative sample of all countries as this survey was voluntary, however, the inclusion of 95 countries in this survey, despite a global pandemic, demonstrates significant global coverage.

In this analysis, a 'strong' influence was considered any country response that indicated either a 'very strong' or a 'strong' influence. As such, the most precise wording of the results would be 'did the findings of the vulnerability and adaptation assessment have 'at least a strong' influence on health policy and programmes or the allocation of human and financial resources'. This wording caused some confusion. For clarity, this has been worded as simply, 'have a 'strong' influence.

Additional analysis

Full list of countries participating in the 2021 WHO Health and Climate Change Global Survey: Argentina, Azerbaijan, Bahamas, Bahrain, Barbados, Belize, Benin, Bhutan, Bolivia (Plurinational Bolivia State of), Brazil, British Virgin Islands, Brunei Darussalam, Bulgaria, Cabo Verde, Cambodia, Cameroon, Canada, China, Colombia, Comoros, Costa Rica, Côte d'Ivoire, Croatia, Cuba, Cyprus, Czech Republic, Dominica, Dominican Republic, Egypt, El Salvador, Eritrea, Estonia, Ethiopia, Germany, Ghana, Grenada, Guatemala, Guinea, Guyana, Haiti, India, Iran (Islamic Republic of), Israel, Italy, Jamaica, Jordan, Kazakhstan, Kenya, Kyrgyzstan, Lebanon, Lithuania, Madagascar, Malawi, Marshall Islands, Micronesia (Federated States of), Mozambique, Netherlands, Nicaragua, Nigeria, North Macedonia, Occupied Palestinian Territory, Oman, Palau, Papua New Guinea, Paraguay, Peru, Philippines, Poland, Portugal, Republic of Moldova, Rwanda, Saint Kitts and Nevis, Saint Lucia, San Marino, Sao Tome And Principe, Saudi Arabia, Serbia, Seychelles, Sierra Leone, Slovakia, South Africa, Sri Lanka, Suriname, Sweden, Thailand, Togo, Trinidad and Tobago, Turkmenistan, United Republic of Tanzania, United States of America, Uruguay, Vanuatu, Yemen, Zambia, Zimbabwe.

COP26 Health Programme: Supported by the UK government, as the Presidency of COP26, the World Health Organization (WHO), Health Care Without Harm (HCWH) and the UNFCCC Climate Champions, the COP26 Health Programme enables transformational change to protect the health of people and the planet.

Initiatives under the COP26 Health Programme include:

- Building climate resilient health systems
- Developing low carbon sustainable health systems
- Adaptation Research for Health
- The inclusion of health priorities in Nationally Determined Contributions
- Raising the voice of health professionals as advocates for stronger ambition on climate change

Two of the Programme's key initiatives support countries in developing Climate Resilient and Low Carbon Sustainable Health Systems, with countries expected to announce their commitments to these initiatives by COP26 in November 2021. Commitments are anticipated to be implemented in the coming years and will allow countries to develop a roadmap for future investments in climate resilient and low carbon sustainable health systems and facilities.

Commitment 1: Climate resilient health systems:

- Commit to conduct climate change and health vulnerability and adaptation assessments (V&As) at population level and/or health care facility level by a stated target date
- Commit to develop a Health National Adaptation Plan (HNAP) informed by the health V&A, which forms part of the National Adaptation Plan to be published by a stated target date
- Commit to use the V&A and HNAP to facilitate access to climate change funding for health (e.g., project proposals submitted to the Global Environmental Facility, Green Climate Fund, Adaptation Fund, or GCF Readiness programme).

Commitment 2: Sustainable low carbon health systems:

- High ambition/high emitters: Commit to set a target date by which to achieve health system net zero emissions (ideally by 2050)
- All countries: Commitment to deliver a baseline assessment of greenhouse gas emissions of the health system (including supply chains)
- All countries: Commit to develop an action plan or roadmap by a set date to develop a sustainable low carbon health system (including supply chains) which also considers human exposure to air pollution and the role the health sector can play in reducing exposure to air pollution through its activities and its actions

List of countries that have signed on to commitment 1: building climate resilient health systems: Argentina, Bahamas, Bahrain, Bangladesh, Belgium, Belize, Bhutan, Cabo Verde, Canada, Central African Republic, Colombia, Costa Rica, Cote d'Ivoire, the Democratic Republic of Congo, Dominican Republic, Egypt, Ethiopia, Fiji, Germany, Georgia, Ghana, Guinea, Indonesia, Ireland, Jamaica, Jordan, Kenya, Lao People's Democratic Republic, Liberia, Madagascar, Malawi, Maldives, Mauritania, Morocco, Mozambique, Nepal, Netherlands, Nigeria, Norway, occupied Palestinian territory, Oman, Pakistan, Panama, Peru, Rwanda, Sao Tome and Principe, Sierra Leone, Spain, Sri Lanka, United Republic of Tanzania, Togo, Tunisia, United Arab Emirates, Uganda, the United Kingdom, the United states of America, Yemen.

Indicator 2.1.2: National Adaptation Plans for Health

The collection of data for this exercise included a voluntary national survey, the WHO Health and Climate Change Global Survey (2021) that was sent to all WHO Member States and a small number of non-Member territories. The survey was completed by ministry of health focal points. Of the 194 WHO member states and non-Member territories, 95 participated in the survey, providing representation from all six WHO regions.

Survey participation has grown substantially from the 40 Member States that completed the 2015 WHO Health and Climate Change Global Survey. The survey was planned to be conducted every two years, although global circumstances have resulted in a three-year gap between surveys.

Validation of the 2021 country reported data was undertaken in multiple steps. First, survey responses were reviewed for missing information or inconsistencies with follow-up questions directed to survey respondents. A summary of responses was shared with WHO regional focal points and key informants for review, comments, and validation. Source documents including national health strategies and plans, and climate change and health vulnerability and adaptation assessments were collected. A desktop review of these source documents was

conducted to compare with survey results with follow-up to survey respondents to seek clarification or additional documentation. Findings were also cross referenced with existing external publications. Data detailing all the ministries, institutions and national stakeholders that provided contributions to or review of the survey responses were collected in order to provide insight into the national consultation process of each survey submission. Of the 95 country submissions, 69 surveys were completed in consultation with one to six different stakeholders, ministries, or institutions. Five countries consulted between 10 to 12 stakeholders, ministries, or institutions. 15 countries did not consult with other entities or health programmes. Information was not available for the remaining six countries. Finally, all respondents reviewed and acknowledged the WHO data policy statement on the use and sharing of data collected by WHO in Member States outside the context of public health emergencies.

Of note, due to the ongoing pandemic, the standard data collection procedures were modified to reduce reporting burden on countries that wished to participate in the global survey but that were facing human resource constraints due to pandemic response. In eight cases, WHO prepared pre-filled survey questionnaires with data provided by ministries of health in the previous 2018 survey cycle or using data the countries had published in the 2020/2021 WHO UNFCCC health and climate change country profile when available. These countries were requested to review, revise, and complete the hard copy questionnaires. These hard copy questionnaires were then entered into the online platform by WHO. The same data validation steps as described above were then followed. Additionally, a number of countries requested an extension of the reporting period. As such, there may be a slight increase in the total number of participating countries and the WHO Health and Climate Change Global Survey Report and associated dynamic data dashboard will provide the definitive summary of findings.

Further information on the WHO Health and Climate Change Global Survey, its methodology, and the WHO UNFCCC Health and Climate Change Country Profile Project can be found at https://www.who.int/activities/monitoring-science-and-evidence-on-climate-change-and-health/health-and-climate-global-survey

The questionnaire asks whether countries have a national health and climate change plan/strategy in place, defined as "a government plan or strategy which considers the health risks of climate change, and health adaptation and/or health resilience to climate change. It could be part of a broader national climate change plan/strategy that includes health". If they have it, countries are requested to upload the plan documentation. Data is collected on:

- *The year* when the national health and climate change plan/strategy was completed/published.
- The time period covered by the national health and climate change plan/strategy
- Who led its development (options: "Ministry of Health", "Other ministry/government authority with Ministry of Health inputs", "Other ministry/government authority without Ministry of Health inputs", "Unknown", "Other (Specify)")
- Whether the national health and climate change plan/strategy developed as part of:
  - The National Adaptation Plan (NAP) process of the United Nations Framework Convention on Climate Change (UNFCCC).
  - National Portfolios of Actions on Environment and Health
  - National Plan for Poverty Reduction or National Development Plan.
  - A Situation Analysis and Needs Assessment (SANA)
  - Other National Process (Specify:)
  - Unknown/None of the above/Not Applicable

- Whether the development of the national health and climate change plan/strategy was informed by a climate change and health vulnerability and adaptation assessment
- What are the current sources of funding for implementation of the national health and climate change plan/strategy (Options: "Fully governmental/ministerial", "Mix of government and external", "Fully external", "No financing currently available", "Unknown")
- The level of implementation of the national health and climate change plan/strategy. Options:
  - Very high (action is being taken on all of the plan/strategy priorities)
  - High (action is being taken on a majority of the plan/strategy priorities)
  - Moderate (action is being taken on some of the plan/strategy priorities)
  - Low (limited action is being taken on the plan/strategy priorities)
  - None (no action is currently being taken on the plan/strategy priorities)

○ Unknown

- Barriers that have been encountered in implementing the national health and climate change plan/strategy. Options:
  ○ Incomplete or lack of comprehensive plan/strategy
  ○ Lack of endorsement by Ministry of Health
  ○ Insufficient finance/budget
  ○ Insufficient human resource capacity
  ○ Insufficient prioritization or competing priorities
  ○ Insufficient multi-sectoral collaboration
  ○ Insufficient research and evidence
  ○ Insufficient technologies, tools and methods
  ○ COVID-19 related constraints
  ○ Unknown
  ○ Other (specify)

More information on health national adaptation plans (HNAPS), can be found here: https://www.who.int/teams/environment-climate-change-and-health/climate-change-and-health/capacity-building/toolkit-on-climate-change-and-health/adaptation

Data

2021 WHO Health and Climate Change Global Survey.[164]

Caveats

The survey sample is not a representative sample of all countries as this survey was voluntary, however, the inclusion of 95 countries in this survey, despite a global pandemic, demonstrates significant global coverage.

Future form of the indicator

The WHO Health and Climate Change Global Survey is a triennial survey and will continue to be the primary source of data to track this indicator.

The future evolution of this indicator will explore the use of evidence (particularly findings from vulnerability and adaptation assessments) to inform the development of strategies/plans and progress on level of implementation of strategies/plans. With more countries initiating the national adaptation plan (NAP) process, alignment of the health component with the overall NAP will also be more closely monitored and examined. Interim information regarding the specific content of national strategies/plans, as explored in this qualitative analysis, may be re-assessed in the future.

Additional analysis

Full list of countries participating in the 2021 WHO Health and Climate Change Global Survey: Argentina, Azerbaijan, Bahamas, Bahrain, Barbados, Belize, Benin, Bhutan, Bolivia (Plurinational Bolivia State of), Brazil, British Virgin Islands, Brunei Darussalam, Bulgaria, Cabo Verde, Cambodia, Cameroon, Canada, China, Colombia, Comoros, Costa Rica, Côte d'Ivoire, Croatia, Cuba, Cyprus, Czech Republic, Dominica, Dominican Republic, Egypt, El Salvador, Eritrea, Estonia, Ethiopia, Germany, Ghana, Grenada, Guatemala, Guinea, Guyana, Haiti, India, Iran (Islamic Republic of), Israel, Italy, Jamaica, Jordan, Kazakhstan, Kenya, Kyrgyzstan, Lebanon, Lithuania, Madagascar, Malawi, Marshall Islands, Micronesia (Federated States of), Mozambique, Netherlands, Nicaragua, Nigeria, North Macedonia, Occupied Palestinian Territory, Oman, Palau, Papua New Guinea, Paraguay, Peru, Philippines, Poland, Portugal, Republic of Moldova, Rwanda, Saint Kitts and Nevis, Saint Lucia, San Marino, Sao Tome And Principe, Saudi Arabia, Serbia, Seychelles, Sierra Leone, Slovakia, South Africa, Sri Lanka, Suriname, Sweden, Thailand, Togo, Trinidad and Tobago, Turkmenistan, United Republic of Tanzania, United States of America, Uruguay, Vanuatu, Yemen, Zambia, Zimbabwe.

COP26 Health Programme

Supported by the UK government, as the Presidency of COP26, the World Health Organization (WHO), Health Care Without Harm (HCWH) and the UNFCCC Climate Champions, the COP26 Health Programme enables transformational change to protect the health of people and the planet.

Initiatives under the COP26 Health Programme include:

- Building climate resilient health systems
- Developing low carbon sustainable health systems
- Adaptation Research for Health
- The inclusion of health priorities in Nationally Determined Contributions
- Raising the voice of health professionals as advocates for stronger ambition on climate change

Two of the Programme's key initiatives support countries in developing Climate Resilient and Low Carbon Sustainable Health Systems, with countries expected to announce their commitments to these initiatives by COP26 in November 2021. Commitments are anticipated to be implemented in the coming years and will allow countries to develop a roadmap for future investments in climate resilient and low carbon sustainable health systems and facilities.

Commitment 1: Climate resilient health systems

- Commit to conduct climate change and health vulnerability and adaptation assessments (V&As) at population level and/or health care facility level by a stated target date
- Commit to develop a health National Adaptation Plan informed by the health V&A, which forms part of the National Adaptation Plan to be published by a stated target date
- Commit to use the V&A and HNAP to facilitate access to climate change funding for health (e.g., project proposals submitted to the Global Environmental Facility, Green Climate Fund, Adaptation Fund, or GCF Readiness programme).

Commitment 2: Sustainable low carbon health systems

- High ambition/high emitters: Commit to set a target date by which to achieve health system net zero emissions (ideally by 2050)
- All countries: Commitment to deliver a baseline assessment of greenhouse gas emissions of the health system (including supply chains)
- All countries: Commit to develop an action plan or roadmap by a set date to develop a sustainable low carbon health system (including supply chains) which also considers human exposure to air pollution and the role the health sector can play in reducing exposure to air pollution through its activities and its actions

List of countries that have signed on to commitment 1: building climate resilient health systems

Argentina, Bahamas, Bahrain, Bangladesh, Belgium, Belize, Bhutan, Cabo Verde, Canada, Central African Republic, Colombia, Costa Rica, Cote d'Ivoire, the Democratic Republic of Congo, Dominican Republic, Egypt, Ethiopia, Fiji, Germany, Georgia, Ghana, Guinea, Indonesia, Ireland, Jamaica, Jordan, Kenya, Lao People's Democratic Republic, Liberia, Madagascar, Malawi, Maldives, Mauritania, Morocco, Mozambique, Nepal, Netherlands, Nigeria, Norway, occupied Palestinian territory, Oman, Pakistan, Panama, Peru, Rwanda, Sao Tome and Principe, Sierra Leone, Spain, Sri Lanka, United Republic of Tanzania, Togo, Tunisia, United Arab Emirates, Uganda, the United Kingdom, the United states of America, Yemen.

Indicator 2.1.3: City-Level Climate Change Risk Assessments

Methods

Indicator 2.1.3 captures data on at the city level on:

1. cities that have undertaken a climate change risk or vulnerability assessment and;

2. the perceived vulnerability city leaders of their public health assets to climate change

Data

1. 2021 CDP Annual Cities Survey

Caveats

This is a self-reported survey, non-compulsory survey as such data provided may be subjective and response rates can fluctuate, with low uptake in certain areas, particularly the Middle East.

Future form of the indicator

The CDP collect this data annually and it is foreseen that the data collection will continue to 2030. Additional analyses may be conducted using data from the CDP annual survey to monitor associations between city-level health vulnerabilities and track reporting trends over time.

Additional analysis

For their reports for 2020 and 2021, the Cities questionnaire included two questions on COVID-19 asking cities to reflect on:

- impact of COVID-19 on climate action in your city
- impact of COVID-19 economic response on city's budget for financing climate action in your city

	Number	Percentage
Decreased emphasis on climate action	116	14%
Increased emphasis on climate action	310	38.50%
No change on emphasis on climate action	325	40%
Uncertain	54	7%
Total	805	

Table 17 Results from questionnaire on impact of COVID-19 on Climate Action

	Number	Percentage
Increased finance available for climate change	178	22%
No change on finance available for climate change	332	42%
Reduced finance available for climate change	242	30%
Uncertain	46	6%
Total	798	

Table 18 Results from questionnaire on economic impacts of COVID-19 on city's budget for climate action

2.2: Enabling Conditions, Adaptation Delivery and Implementation

Indicator 2.2.1: Climate Information for Health

Methods

The collection of data for this exercise included a voluntary national survey, the WHO Health and Climate Change Global Survey (2021) that was sent to all WHO Member States and a small number of non-Member territories. The survey was completed by ministry of health focal points. Of the 194 WHO member states and non-Member territories, 95 participated in the survey, providing representation from all six WHO regions.

Survey participation has grown substantially from the 40 Member States that completed the 2015 WHO Health and Climate Change Global Survey. The survey was planned to be conducted every two years, although global circumstances have resulted in a three-year gap between surveys.

Validation of the 2021 country reported data was undertaken in multiple steps. First, survey responses were reviewed for missing information or inconsistencies with follow-up questions directed to survey respondents. A summary of responses was shared with WHO regional focal points and key informants for review, comments, and validation. Source documents including national health strategies and plans, and climate change and health vulnerability and adaptation assessments were collected. A desktop review of these source documents was conducted to compare with survey results with follow-up to survey respondents to seek clarification or additional documentation. Findings were also cross referenced with existing external publications. Data detailing all the ministries, institutions and national stakeholders that provided contributions to or review of the survey responses were collected in order to provide insight into the national consultation process of each survey submission. Of the 95 country submissions, 69 surveys were completed in consultation with one to six different stakeholders, ministries, or institutions. Five countries consulted between 10 to 12 stakeholders, ministries, or institutions. 15 countries did not consult with other entities or health programmes. Information was not available for the remaining six countries. Finally, all respondents reviewed and acknowledged the WHO data policy statement on the use and sharing of data collected by WHO in Member States outside the context of public health emergencies.

Of note, due to the ongoing pandemic, the standard data collection procedures were modified to reduce reporting burden on countries that wished to participate in the global survey but that were facing human resource constraints due to pandemic response. In eight cases, WHO prepared pre-filled survey questionnaires with data provided by ministries of health in the previous 2018 survey cycle or using data the countries had published in the 2020/2021 WHO UNFCCC health and climate change country profile when available. These countries were requested to review, revise, and complete the hard copy questionnaires. These hard copy questionnaires were then entered into the online platform by WHO. The same data validation steps as described above were then followed. Additionally, a number of countries requested an extension of the reporting period. As such, there may be a slight increase in the total number of participating countries and the WHO Health and Climate Change Global Survey Report and associated dynamic data dashboard will provide the definitive summary of findings.

Further information on the WHO Health and Climate Change Global Survey, its methodology, and the WHO UNFCCC Health and Climate Change Country Profile Project can be found at https://www.who.int/activities/monitoring-science-and-evidence-on-climate-change-and-health/health-and-climate-global-survey

In the survey, countries are requested to indicate whether, for specific climate-sensitive health risks/outcomes, a health surveillance system exists; if the health surveillance system includes meteorological information; if there is a climate-informed health early warning system (EWS); and if there is a health sector response plan in place. In this survey, meteorological information is understood as short-term weather information, seasonal climate information or long-term climate information
Countries are requested to upload health sector response plan(s) if they are in place.

For each climate-sensitive health risk or outcome, countries are requested to indicate whether:

- A health surveillance system exists
- The health surveillance system includes meteorological information

- Climate-informed health early warning system (EWS) are in place
- A climate-informed health early warning system has been evaluated
- A health sector response plan is in place

The climate sensitive health risks or outcomes considered are:

- Climate sensitive health risks/outcomes
- Air-borne and respiratory illnesses
- Heat-related illness
- Injury and mortality from extreme weather events
- Malnutrition and food-borne diseases
- Mental and psychosocial health
- Noncommunicable diseases (NCDs)
- Vector-borne diseases
- Water-borne diseases and other water-related health outcomes
- Zoonoses
- Impacts on health care facilities
- Other (specify)

More information on climate-informed health surveillance and early warning systems is available in the WHO operational framework for building climate resilient health systems (https://www.who.int/publications/i/item/9789241565073), and in the WHO climate change and health toolkit (https://www.who.int/teams/environment-climate-change-and-health/climate-change-and-health/capacity-building/toolkit-on-climate-change-and-health/early-warning-systems)

Data

1. 2021 WHO Health and Climate Change Global Survey.[164]

Caveats

The survey sample is not a representative sample of all countries as this survey was voluntary, however, the inclusion of 95 countries in this survey, despite a global pandemic, demonstrates significant global coverage.

Future form of the indicator

The WHO Health and Climate Change Global Survey is a triennial survey and will continue to be the primary source of data to track this indicator. The future evolution of this indicator will aim to explore the coverage of surveillance and early warning systems and the extent of evaluation of these systems.

This indicator will be revaluated going forward to continue to find the best analysis of surveillance and warning systems with climate information.

Additional analysis

Full list of countries participating in the 2021 WHO Health and Climate Change Global Survey
Argentina, Azerbaijan, Bahamas, Bahrain, Barbados, Belize, Benin, Bhutan, Bolivia (Plurinational Bolivia State of), Brazil, British Virgin Islands, Brunei Darussalam, Bulgaria, Cabo Verde, Cambodia, Cameroon, Canada, China, Colombia, Comoros, Costa Rica, Côte d'Ivoire, Croatia, Cuba, Cyprus, Czech Republic, Dominica, Dominican Republic, Egypt, El Salvador, Eritrea, Estonia, Ethiopia, Germany, Ghana, Grenada, Guatemala, Guinea, Guyana, Haiti, India, Iran (Islamic Republic of), Israel, Italy, Jamaica, Jordan, Kazakhstan, Kenya, Kyrgyzstan, Lebanon, Lithuania, Madagascar, Malawi, Marshall Islands, Micronesia (Federated States of), Mozambique, Netherlands, Nicaragua, Nigeria, North Macedonia, Occupied Palestinian Territory, Oman, Palau, Papua New Guinea, Paraguay, Peru, Philippines, Poland, Portugal, Republic of Moldova, Rwanda, Saint Kitts and Nevis, Saint Lucia, San Marino, Sao Tome And Principe, Saudi Arabia, Serbia, Seychelles, Sierra Leone,

Slovakia, South Africa, Sri Lanka, Suriname, Sweden, Thailand, Togo, Trinidad and Tobago, Turkmenistan, United Republic of Tanzania, United States of America, Uruguay, Vanuatu, Yemen, Zambia, Zimbabwe.

The main text highlights climate-informed health early warning systems developed for heat and extreme weather and climate informed health surveillance systems for other disease outcomes. The WHO Health and Climate Change Global Survey was given to 95 countries (see before). However, not all questions were answered by all countries. Hence, some percentages will differ in denominator. This survey should be regarded as a representative analysis, showing the trends around which countries dare failing to or are developing climate-informed health surveillance and warning. It is also representative of trends by HDI grouping.

As the surveillance of extreme events grows increasingly important, there needs to be an increased number of surveys around health-specific climate surveillance and warning on a wider array of countries.

Indicator 2.2.2: Air Conditioning: Benefits and Harms

Methods

Premature deaths from ambient $PM_{2.5}$ exposure due to electricity use for air conditioning.

To estimate country/region-specific premature deaths from ambient $PM_{2.5}$ exposure due to electricity use for air conditioning, the proportion of total electricity final consumption used for air conditioning (obtained from IEA) was multiplied by the estimated country/region-specific premature deaths due to $PM_{2.5}$ emissions from electric power plants, taken from Indicator 3.3. Indicator 3.3 estimated premature deaths from ambient $PM_{2.5}$ exposure for 137 countries. To calculate premature deaths from ambient $PM_{2.5}$ exposure for each IEA-defined region, premature deaths from ambient $PM_{2.5}$ exposure across the countries classified into each region were summed.

Data

The IEA kindly provided data for 2000–2020, including revisions based on improved IEA analyses of its 2000–2019 data used in the 2021 *Lancet* Countdown report. These data included the proportion of households with air conditioning; CO2 emissions due to air conditioning (megatons); and proportion of total electricity final consumption used for air conditioning (used in the calculation of premature deaths from ambient $PM_{2.5}$ exposure due to electricity use for air conditioning).

Proportion of households with air conditioning and CO2 emissions due to air conditioning were provided for the entire world and for 22 individual countries and 9 IEA-defined regions that did not include the 22 individual countries. The countries and regions together constituted the entire world.

The following are the individual countries: Canada, United States, Russia, Australia, New Zealand, Italy, France, Denmark, Finland, Sweden, United Kingdom, Norway, Iceland, Germany, Mexico, Japan, South Korea, Brazil, China, India. Indonesia, and South Africa

The following are the 9 regions (the 22 individual countries were not included in the regions):

1. Caspian: Armenia, Azerbaijan, Georgia, Kazakhstan, Kyrgyzstan, Tajikistan, Turkmenistan, Uzbekistan
2. Other Europe: Albania, Austria, Belarus, Belgium, Bosnia and Herzegovina, Bulgaria, Croatia, Cyprus, Czech Republic, Estonia, Former Yugoslav Republic of Macedonia, Gibraltar, Greece, Holy See, Hungary, Ireland, Israel, Kosovo, Latvia, Lithuania, Luxembourg, Malta, Moldova, Monaco, Montenegro, Netherlands, Poland, Portugal, Romania, San Marino, Serbia, Slovak Republic, Slovenia, Spain, Switzerland, Turkey, Ukraine
3. North Africa: Algeria, Egypt, Libya, Morocco, Tunisia
4. Other Africa: Angola, Benin, Botswana, Burkina Faso, Burundi, Cameroon, Cape Verde, Central African Republic, Chad, Comoros, Congo, Côte d'Ivoire, Dem. Republic of the Congo, Djibouti, Equatorial Guinea, Eritrea, Eswatini, Ethiopia, Gabon, Gambia, Ghana, Guinea, Guinea-Bissau, Kenya,

Lesotho, Liberia, Madagascar, Malawi, Mali, Mauritania, Mauritius, Mozambique, Namibia, Niger, Nigeria, Réunion, Rwanda, Sao Tome and Principe, Senegal, Seychelles, Sierra Leone, Somalia, South Sudan, Sudan, Togo, Uganda, Tanzania,vZambia, Zimbabwe

5. Chile and Colombia: Chile, Colombia
6. Other Latin America: Antigua and Barbuda, Argentina, Aruba, Bahamas, Barbados, Belize, Bermuda, Bolivia, British Virgin Islands, Cayman Islands, Costa Rica, Cuba, Curaçao, Dominica, Dominican Republic, Ecuador, El Salvador, Falkland Islands (Malvinas), French Guiana, Grenada, Guadeloupe, Guatemala, Guyana, Haiti, Honduras, Jamaica, Martinique, Montserrat, Netherlands (Caribbean), Netherlands Antilles, Nicaragua, Panama, Paraguay, Peru, Saint Kitts and Nevis, Saint Lucia, Saint Pierre and Miquelon, Saint Vincent and the Grenadines, Sint Maarten (Dutch part), Suriname, Trinidad and Tobago, Turks and Caicos Islands, Uruguay, Venezuela
7. Middle East: Bahrain, Iran, Iraq, Jordan, Kuwait, Lebanon, Oman, Qatar, Saudi Arabia, Syria, United Arab Emirates, Yemen
8. Association of Southeast Asian Nations (ASEAN) countries: Brunei Darussalam, Cambodia, Laos, Malaysia, Myanmar, Philippines, Pitcairn, Singapore, Thailand, Vietnam
9. Other Asia: Afghanistan, Bangladesh, Bhutan, China (Macau, China (Taiwan), Cook Islands, Fiji, French Polynesia, Kiribati, Kuwait, Lebanon, Maldives, Mongolia, Nepal, New Caledonia, North Korea, Pakistan, Palau, Papua New Guinea, Samoa, Solomon Islands, Sri Lanka, Timor-Leste, Tonga, Vanuatu

For the estimation of premature deaths from ambient $PM_{2.5}$ exposure due to electricity use for air conditioning, the data provided by the IEA on proportion of total electricity final consumption used for air conditioning grouped Australia and New Zealand; grouped Italy, France, and Germany; and included Denmark, Finland, Iceland, Norway, and Sweden in "Other Europe." In addition, the following countries were not included in the calculation of IEA-defined-region-level number of premature deaths due to $PM_{2.5}$ emissions from electric power plants because, although they were included in IEA regions, they were not included in the assessment of number premature deaths due to $PM_{2.5}$ emissions from electric power plants in indicator 3.3:

Caspian: Tajikistan, Turkmenistan, Uzbekistan

Other Europe: Gibraltar, Holy See, Kosovo, Monaco, San Marino

Other Africa: Réunion, Sao Tome and Principe, Seychelles, South Sudan

Other Latin America: Antigua and Barbuda, Aruba, Bahamas, Barbados, Belize, Bermuda, British Virgin Islands, Cayman Islands, Costa Rica, Cuba, Curaçao, Dominica, Dominican Republic, El Salvador, Falkland Islands (Malvinas), French Guiana, Grenada, Guadeloupe, Guatemala, Guyana, Haiti, Honduras, Jamaica, Martinique, Montserrat, Netherlands Antilles, Nicaragua, Panama, Saint Kitts and Nevis, Saint Lucia, Saint Pierre and Miquelon, Saint Vincent and the Grenadines, Sint Maarten (Dutch part), Suriname, Trinidad and Tobago, Turks and Caicos Islands

Middle East: Bahrain, Iraq, Jordan, Oman, Qatar, Syria, United Arab Emirates, Yemen ASEAN countries: Pitcairn

Other Asia: China (Macau), Cook Islands, Kuwait, Lebanon, Fiji, French Polynesia, Kiribati, Maldives, New Caledonia, Palau, Papua New Guinea, Samoa, Solomon Islands, Timor-Leste, Tonga, Vanuatu

Caveats

Estimate of number of premature deaths due to $PM_{2.5}$ emissions from air conditioning:

To estimate the number of premature deaths due to $PM_{2.5}$ emissions from air conditioning, the finer the spatial resolution, the more accurate the estimates. The data available for electricity final consumption for air conditioning were at the country or region level. Thus, in a given country/region, it was by necessity assumed that the electricity market is completely connected, so that the share of electricity used for air conditioning can be equally applied to power plant emissions throughout the country/region. This assumption may not be accurate, especially for larger countries/regions.

Notably, the sustainability of air conditioning could be increased through generation of electricity by renewable energy and more efficient air conditioning technology. These measures would reduce both CO2 emissions and the number of premature deaths due to $PM_{2.5}$ emissions from air conditioning. Greenhouse gas emissions from air conditioning could be further reduced through phase-out of hydrochlorofluorocarbon refrigerants in favour of refrigerants that are not greenhouse gases, as called for in the 2016 Kigali Amendment to the Montreal Protocol. Technology to capture and recycle waste heat would make air conditioning even more efficient and would reduce its contribution to the urban heat island effect.

Future form of the indicator

The indicator in the 2021 report estimated the number of heat-related deaths averted by air conditioning in the 65-and-older population by country/region and for the world. There were a number of limitations to these estimates, such that they were considered to be “ballpark” estimates that would need considerable refinement in future years. The intention is to present improved estimates in future years, including all age groups.

In addition, city-level case studies to estimate number of lives saved from air conditioning versus premature deaths from exposure to $PM_{2.5}$ due to air conditioning may be performed. The indicator may be updated each year as new data become available on air conditioning use. Trends in country-level or finer vulnerability to heatwave-related mortality could be assessed with cooling degree days. Finally, metrics related to more efficient cooling (e.g., national building codes, minimum energy performance standards, labelling rules for air conditioners) and progress on implementing the Kigali Amendment may be tracked in the future.

Indicator 2.2.3: Urban Green Space

Methods

Urban area spatial extents were defined by the Global Human Settlement (GHS) program of the European commission.[165] The GHS uses remote sensing and demographic data to define more than 10,000 urban centres worldwide. Cities chosen for the indicator were identified as urban centres larger than 500,000 inhabitants. For countries with urban areas that did not meet this threshold, we selected the most populated city where possible, giving a final count of 1,041 cities and 172 countries. Due to missing data in either the GHS or the Normalized Difference Vegetation Index (NDVI) data, 22 countries (mostly small island states) were not represented in the analysis.

Data on population size for all years were collected from the Center for International Earth Science Information Network (CIESN, Columbia University), which models the distribution of human population at 30 arc-second output resolution.[166]

Green space was estimated using the normalized difference vegetation index (NDVI), the most commonly used satellite-based vegetation index. NDVI calculates the ratio of the differences between near infrared radiation and visible radiation to the sum of these two measures. NDVI values range from -1.0 to 1.0 with negative values indicating water and values close to 1 indicating high levels of vegetation density.[167] Publicly available data from the Landsat satellite, a joint program of the USGS and NASA, were used.[168] Landsat images the Earth’s surface at 30-meter resolution approximately every two weeks. To account for seasonal fluctuations, we computed NDVI for each of the following time periods (with season labels based on the northern hemisphere):

- Winter—December 1 of previous year through February 28
- Spring—March 1 through May 31
- Summer—June 1 through August 31
- Fall—September 1 through November 30

We did this for four different years: 2010, 2015, 2020, and 2021. Landsat7 was used for the first year, and Landsat8 for all subsequent years. For each year and city, a total of four exposure metrics were calculated: peak NDVI (maximum NDVI across the four seasons); annual mean NDVI based on the four-season average NDVI; population-weighted average peak NDVI; and population-weighted mean NDVI. The population weighted NDVI was computed for each city by multiplying each NDVI value (peak and four-season average) by the

population size of the corresponding year within the same 1x1 km raster, summing up over the weighted values within the urban extent, and dividing by the sum of the weights, as shown by the equation below:

$$\frac{\sum_{i=1}^{n}(NDVI_i * population_i)}{\sum_{i=1}^{n}(population_i)}$$

Additional analyses include subsetting the data by climate regions as defined by the Koppen Climate Classification System and recomputing the greenness metrics by climate region and year.[169] Google Earth Engine was used to generate raw data and the R Statistical Software was used for data analysis and management and to compute the four metrics described above. 'Level of Greenness' was defined according to the table below:

**Level of Greenness**	**Population-Weighted Peak NDVI**
Exceptionally Low	<0.20
Very low	0.20-0.29
Low	0.30-0.39
Moderate	0.40-0.49
High	0.50-0.59
Very High	0.60-0.69
Exceptionally High	≥0.70

Table 19 Categorization of Greenness Levels

Data

1. Global Human Settlement Programme of the European Commission (GHS) used to identify urban areas[1]
2. Population size identified from NASA GPWv4[166]
3. Satellite data were downloaded from the publicly available Landsat satellite, a joint program of the US Geological Survey and NASA[168]
4. Global climate regions from the Koppen Climate Classification System[169]

Caveats

This approach has some limitations. First, although satellite-based measures of vegetation have been used extensively to measure greenness, NDVI does not provide information on the quality of greenness (e.g., curated park vs vacant lot), the type of green space (e.g., park vs. forest), the type of vegetation (e.g., shrubs vs. trees) or social characteristics (e.g., level of security). However, studies have demonstrated that NDVI performs adequately when compared with environmental psychologists' evaluations of green spaces. In addition, reviews of the literature on greenness and health have been undertaken and found consistent and strong evidence of associations of higher greenness measured by NDVI, with improvements in birthweights, physical activity, lower mortality rates, and lower levels of depression. Second, missing values from GHS or from Landsat data due to cloud cover or other factors limit the generalizability of the findings.

Future form of indicator

Future versions of this indicator will continue to examine trends over time and will aim to estimate the proportion of each city that is green space, in addition to the overall average greenness of an urban centre. We will also explore options to integrate the greenness indicator with other indicators to investigate the associations between urban green space and multiple measures, including heat-related exposures and health effects, exposure of vulnerable populations, and loss of physical activity and/or labour capacity.

Additional analysis

Year	% > Moderate Greenness
2010	15%
2015	28%
2020	28%
2021	27%

Table 20 Global percent moderate or above (population-weighted average peak-season NDVI ≥0.40)

Year	Pop-weighted average peak-season NDVI
2010	0.28
2015	0.34
2020	0.34
2021	0.34

Table 21 Global average population-weighted peak-season NDVI

Region	2010	2015	2020	2021
**Low**	0.25	0.30	0.29	0.29
**Medium**	0.31	0.37	0.37	0.37
**High**	0.26	0.31	0.32	0.31
**Very High**	0.32	0.36	0.36	0.35

Table 22 Population-weighted peak-season NDVI by HDI group

Region	2010	2015	2020	2021
**Arid**	0.20	0.24	0.25	0.24
**Continental**	0.32	0.37	0.39	0.38
**Polar**	0.14	0.14	0.13	0.13
**Temperate**	0.29	0.35	0.35	0.35
**Tropical**	0.32	0.38	0.38	0.38

Table 23: Population-weighted peak-season NDVI by climate region

Region	2010	2015	2020	2021
**African**	0.27	0.33	0.32	0.31
**Americas**	0.29	0.34	0.34	0.33
**E Mediterranean**	0.18	0.22	0.22	0.21
**European**	0.34	0.38	0.37	0.37
**SE Asian**	0.34	0.40	0.40	0.40
**W Pacific**	0.24	0.30	0.32	0.31

Table 24: Population-weighted peak-season NDVI by WHO region

Region	2010	2015	2020	2021
**Low**	9%	19%	17%	16%
**Medium**	18%	37%	36%	39%
**High**	10%	16%	17%	16%
**Very High**	20%	38%	38%	33%

Table 25:Percent moderate or above by HDI (population-weighted average peak-season NDVI ≥0.40)

Region	2010	2015	2020	2021
**Arid**	4%	4%	5%	6%
**Continental**	24%	48%	44%	43%
**Polar**	0%	0%	0%	0%
**Temperate**	11%	27%	26%	25%
**Tropical**	23%	38%	39%	38%

Table 26 :Regional percent moderate or above (population-weighted average peak-season NDVI ≥0.40)

≥0.40)

Region	2010	2015	2020	2021
**African**	10%	21%	20%	17%
**Americas**	16%	27%	28%	25%
**E Mediterranean**	4%	5%	6%	5%
**European**	25%	44%	45%	40%
**SE Asian**	24%	47%	46%	49%
**W Pacific**	2%	9%	9%	8%

Table 27: Percent moderate or above by WHO region (population-weighted average peak-season NDVI

Region	Peak NDVI	Four-season NDVI	Pop. weighted Peak NDVI	Pop. weighted Four-season NDVI
**Low**	0.29	0.22	0.26	0.19
**Medium**	0.27	0.21	0.25	0.17
**High**	0.32	0.25	0.31	0.20
**Very High**	0.33	0.26	0.32	0.22
**Global Mean**	0.31	0.24	0.28	0.20

Table 28: Estimates of Urban Green Space by HDI (2010)

Region	Peak NDVI	Four-season NDVI	Pop. weighted Peak NDVI	Pop. weighted Four-season NDVI
**Low**	0.34	0.28	0.31	0.25
**Medium**	0.32	0.26	0.30	0.24
**High**	0.38	0.31	0.37	0.31
**Very High**	0.37	0.29	0.36	0.28
**Global Mean**	0.35	0.29	0.24	0.27

Table 29: Estimates of Urban Green Space by HDI (2015)

Region	Peak NDVI	Four-season NDVI	Pop. weighted Peak NDVI	Pop. weighted Four-season NDVI
**Low**	0.34	0.28	0.32	0.25
**Medium**	0.31	0.25	0.29	0.24
**High**	0.38	0.32	0.37	0.31
**Very High**	0.36	0.29	0.36	0.28
**Global Mean**	0.36	0.29	0.34	0.28

Table 30: Estimates of Urban Green Space by HDI (2020)

Region	Peak NDVI	Four-season NDVI	Pop. weighted Peak NDVI	Pop. weighted Four-season NDVI
**Low**	0.34	0.28	0.31	0.25
**Medium**	0.31	0.25	0.29	0.24
**High**	0.38	0.30	0.37	0.30
**Very High**	0.36	0.28	0.35	0.27
**Global Mean**	0.35	0.28	0.34	0.27

Table 31: Estimates of Urban Green Space by HDI (2021)

Climate Region	Peak NDVI	Four-season NDVI	Pop. weighted Peak NDVI	Pop. weighted Four-season NDVI
**Arid**	0.21	0.17	0.20	0.14
**Continental**	0.33	0.23	0.32	0.19
**Polar**	0.15	0.12	0.14	0.11
**Temperate**	0.31	0.24	0.29	0.21
**Tropical**	0.36	0.29	0.32	0.22

Table 32: Estimates of Urban Green Space by Climate Region (2010)

Climate Region	Peak NDVI	Four-season NDVI	Pop. weighted Peak NDVI	Pop. weighted Four-season NDVI
**Arid**	0.26	0.21	0.24	0.20
**Continental**	0.38	0.26	0.37	0.25
**Polar**	0.17	0.14	0.14	0.12
**Temperate**	0.37	0.30	0.35	0.28
**Tropical**	0.40	0.34	0.38	0.32

Table 33: Estimates of Urban Green Space by Climate Region (2015)

Climate Region	Peak NDVI	Four-season NDVI	Pop. weighted Peak NDVI	Pop. weighted Four-season NDVI
**Arid**	0.26	0.21	0.25	0.20
**Continental**	0.40	0.27	0.39	0.26
**Polar**	0.15	0.13	0.13	0.11
**Temperate**	0.36	0.30	0.35	0.29
**Tropical**	0.40	0.34	0.38	0.32

Table 34: Estimates of Urban Green Space by Climate Region (2020)

Climate Region	Peak NDVI	Four-season NDVI	Pop. weighted Peak NDVI	Pop. weighted Four-season NDVI
**Arid**	0.25	0.21	0.24	0.20
**Continental**	0.39	0.26	0.38	0.25
**Polar**	0.16	0.12	0.13	0.10
**Temperate**	0.36	0.29	0.35	0.28
**Tropical**	0.40	0.34	0.38	0.32

Table 35: Estimates of Urban Green Space by Climate Region (2021)

Region	Peak NDVI	Four-season NDVI	Pop. weighted Peak NDVI	Pop. weighted Four-season NDVI
**African**	0.30	0.24	0.27	0.18
**Americas**	0.32	0.26	0.29	0.24
**E Mediterranean**	0.19	0.15	0.18	0.13
**European**	0.35	0.27	0.34	0.21
**SE Asian**	0.36	0.27	0.34	0.22
**W Pacific**	0.27	0.19	0.24	0.17

Table 36: Estimates of Urban Green Space by WHO region (2010)

Region	Peak NDVI	Four-season NDVI	Pop. weighted Peak NDVI	Pop. weighted Four-season NDVI
**African**	0.35	0.28	0.33	0.26
**Americas**	0.36	0.31	0.34	0.29
**E Mediterranean**	0.23	0.20	0.22	0.19
**European**	0.38	0.29	0.37	0.28
**SE Asian**	0.41	0.34	0.41	0.34
**W Pacific**	0.33	0.25	0.30	0.23

Table 37: Estimates of Urban Green Space by WHO region (2015)

Region	Peak NDVI	Four-season NDVI	Pop. weighted Peak NDVI	Pop. weighted Four-season NDVI
**African**	0.34	0.27	0.32	0.26
**Americas**	0.36	0.30	0.34	0.28
**E Mediterranean**	0.24	0.20	0.22	0.19
**European**	0.38	0.29	0.37	0.28
**SE Asian**	0.41	0.35	0.41	0.34
**W Pacific**	0.34	0.26	0.32	0.24

Table 38: Estimates of Urban Green Space by WHO region (2020)

Region	Peak NDVI	Four-season NDVI	Pop. weighted Peak NDVI	Pop. weighted Four-season NDVI
**African**	0.33	0.27	0.32	0.25
**Americas**	0.35	0.30	0.33	0.28
**E Mediterranean**	0.22	0.19	0.21	0.18
**European**	0.38	0.28	0.37	0.27
**SE Asian**	0.41	0.34	0.41	0.33
**W Pacific**	0.33	0.26	0.31	0.24

Table 39: Estimates of Urban Green Space by WHO region (2021)

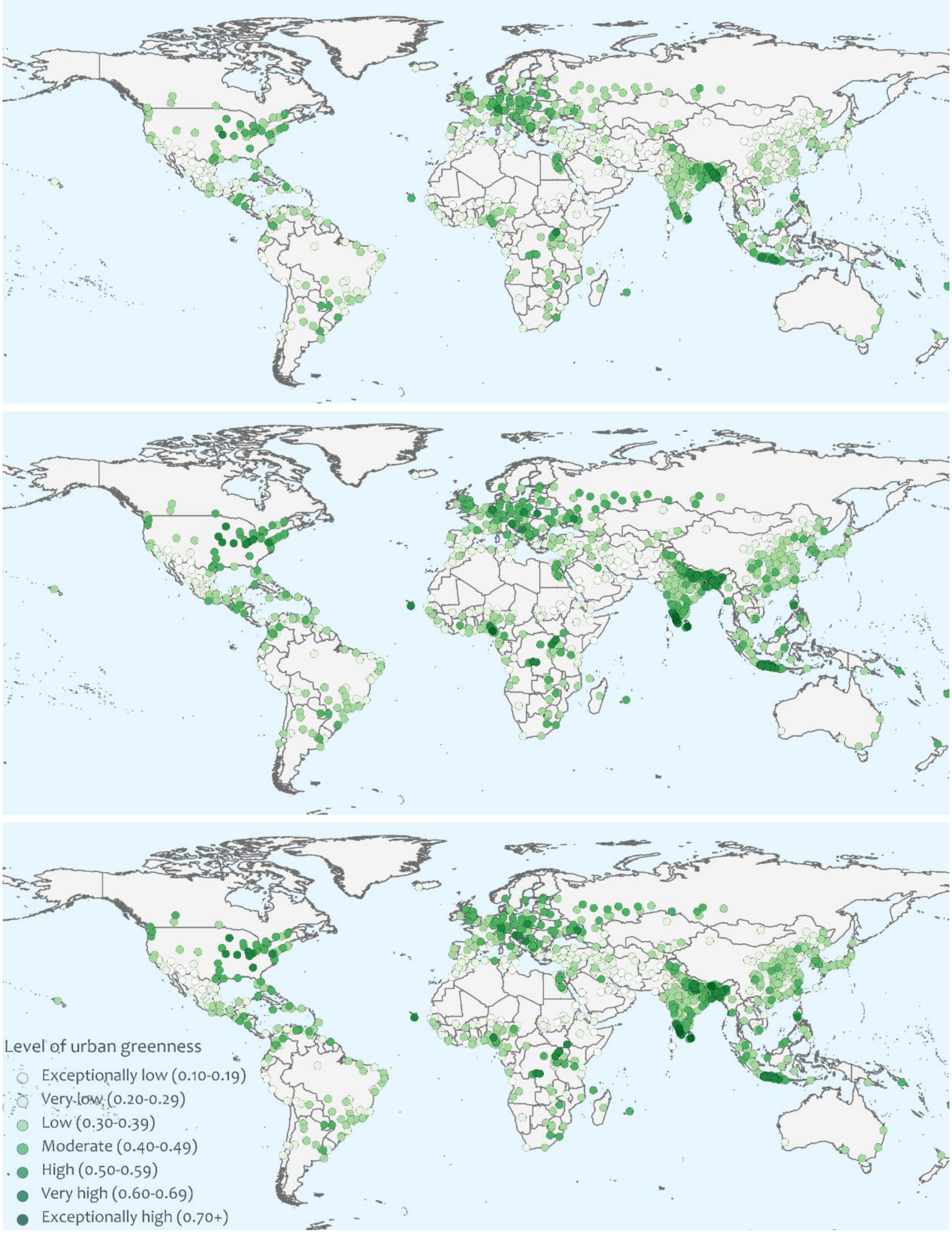

Figure 56 Level of urban greenness in urban centres with more than 500,000 inhabitants in 2010, 2015 and 2020. The numbers in brackets represent the population-weighted NDVI level.

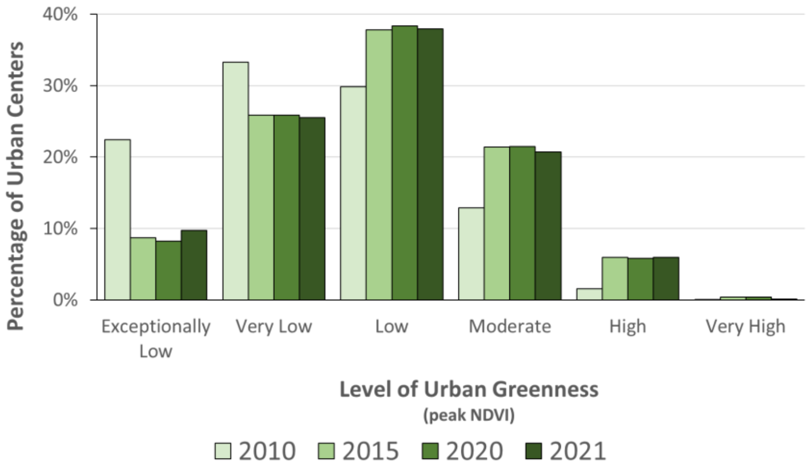

Figure 57 Percentages of cities by urban greenness level over multiple years

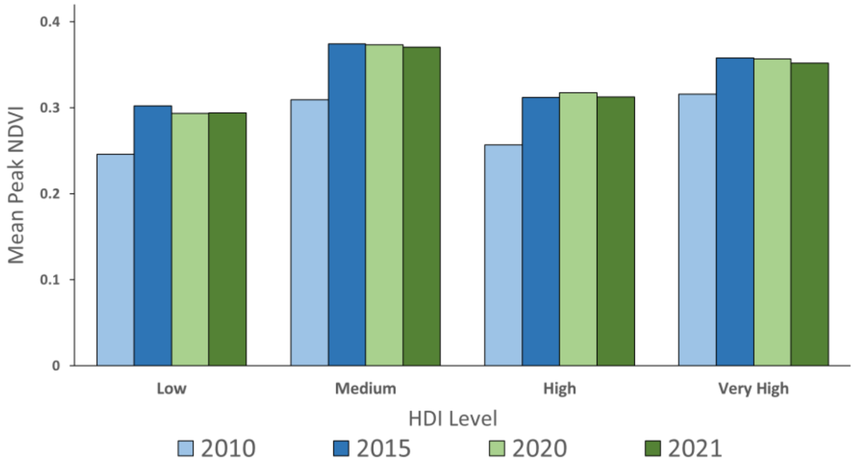

Figure 58 Mean, population-weighted, peak-season NDVI by HDI group and year

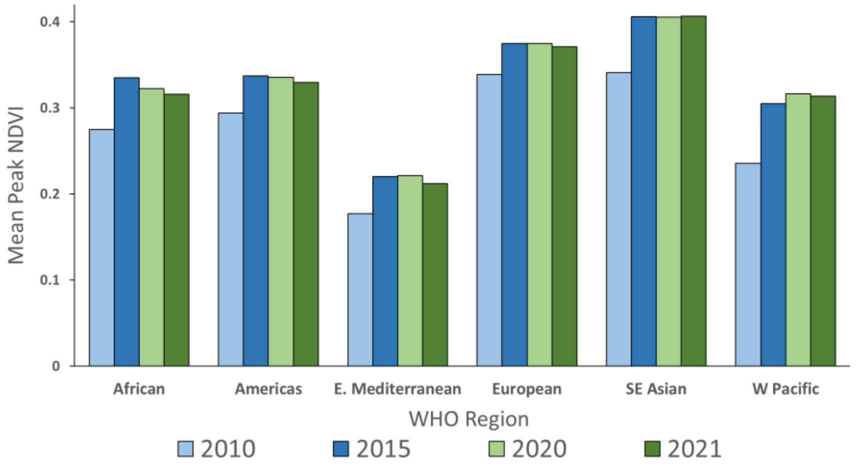

Figure 59 Mean, population-weighted, peak-season NDVI by WHO region and year.

Indicator 2.2.4: Health Adaptation-Related Funding

Methods

The methodology for obtaining the data on potential spending towards health adaptation and health-related adaptation for this indicator remains the same as previous years. Two significant changes were made to the analysis in the last two reporting cycles, however. To present a more cohesive full report, the data for this indicator was converted to USD. Additionally, the definition for health-related spending in non-health sectors was expanded.

The 'Adaptation and Resilience to Climate Change' dataset is the same, annually updated, data source that was used in the 2017–2021 *Lancet* Countdown reports.[11,170-174] It measures spending on economic activities related to adaptation and resilience to climate change. It was developed by the data research firm kMatrix in partnership with numerous stakeholders.[174] It includes the key adaptation measure identified by the IPCC. This classification of adaptation activities was originally developed through attempts by the UK Department for Environment, Food and Rural Affairs to measure adaptation in 2009/2010. The definition of adaptation activities was extended through collaboration with the Greater London Authority in 2014 and updated through a project with Climate-KIC in 2017. This added several new industrial sectors as well as significantly expanding the activities under health and healthcare.

The methodology used for data acquisition and analysis is based on a system called as 'profiling', which was originally developed at Harvard Business School to track and analyse technical and industrial change.[175] This is the basis for building taxonomies of economic activities and value chains, which can then be populated with estimates of key economic metrics like sales value and employment by triangulating transactional and operational business data to estimate economic values. This methodology is particularly valuable in areas where government statistics and standard industry classifications are not available. When measuring an industry or sector, the new taxonomy is populated from the bottom up, searching for evidence for the ideal definition and including only economic activities where sufficient evidence is available.

For each transaction listed in the adaptation economy data, a minimum of seven separate sources must independently record the transaction for it to be confirmed and included in the database. Triangulating data from multiple sources permits large volumes of unsorted, fragmented data of different types from different sources to be processed to arrive at more accurate estimates of transactional value that would not be possible using a single source. For the adaptation economy, data is produced to a confidence level of around 80%. Accessing and analysing multiple types of data is also key to identifying the 'purpose' behind an economic activity, which is key for accurately assigning economic activities to the adaptation dataset. Developing the new definition of adaptation and resilience to climate change involved the top-down taxonomy of the entire 'make and mend' economy, and then adaptation and resilience in all forms. Then these categories were filtered to isolate economic activities that can be strictly identified as being relevant to adaptation and resilience to climate change. The taxonomy of A&RCC is drawn from 11 sectors of the economy at-large: Agriculture & Forestry, Built Environment, Disaster Preparedness, Energy, Health/Health Care, ICT, Natural Environment, Professional Services, Transport, Waste, and Water.[176]

There are a number of activities across different sectors that are 'health-related' in the adaptation and resilience to climate change dataset, outside of the strictly defined healthcare sector. This indicator quantifies spending related to health adaptation in two categories — 1) all spend in health and healthcare sectors; 2) 'health-related' spend in other sectors.

For the 2020 *Lancet* Countdown report, the definition of health-related spending was developed in consultation with experts in climate change adaptation and health. Health-related spending activities in non-health sectors were identified based on the following definition:

Health-related adaptation spend outside of the health sector is spend that occurs:

In the following sectors: agriculture & forestry, built environment, disaster preparedness, energy, transportation, waste, or water sectors; and

Directly impacts one or more basic determinant of health: food, water, air, or shelter. These correspond closely with "physiological needs" in Maslow's Hierarchy of Needs.

Further, spending activities classified as health-related must have an obvious and intuitive relationship to health. A broad definition of shelter is adopted, referring to social interconnectedness, domestic and public dwellings.

Geographical Coverage:

The A&RCC dataset has global coverage for 226 countries and territories. Data has been reported for a subset of countries and territories for whom adaptation spending data, regional and income classifications, and GDP and population estimates are available. This year's indicator covers 177 countries and territories with data reported in the A&RCC dataset, which are: assigned a region in the WHO regional classification, an HDI Classification in the UNDP's Human Development Report, and GDP and population estimates from the IMF World Economic Outlook October 2021 update.[177]

Methods for the later element of the indicator analysing funding allocation through the Green Climate Fund are as follows:

Data Collection:

Data Collection on Funding Approved for Adaptation and Cross-Cutting Projects:

Data were collected from PDF files of Project Approval Documents, accessed via the GCF Project Portfolio, and collated into a spreadsheet.

The GCF Project Portfolio is accessible online from the GCF Website following the Prompts: https://www.greenclimate.fund/ > Projects & Programmes > Lists of Projects. The filter functionality was used to filter projects by 'Theme' [Adaptation] or [Cross-Cutting] and 'Date' [2021].

PDF files of the Project Approval Documents were downloaded from each of the relevant filtered projects, reviewed individually, and key data points were transferred into a spreadsheet, including:

Project Reference Number

Project Name

Project Region

Total GCF Financing

GCF Financing Instrument (Concessional Loan, Grant, Equity, Guarantee)

Percentage of Financing for Adaptation Elements of the Project, outlined in section A.4 Result Area(s)

Percentage of Finance for 'Health, Food, and Water Security' Elements of the Project, outlined in section A.4 Result Area(s)

Project objective described in Section B.3. Project/Programme Description of the Project Approval Document

Presence of GCF Outcome A2.0: Increased resilience of health and wellbeing within the Logical Framework of the Project Approval Document

Transfer of data were validated by two peers, to ensure that no human error was introduced during the process of data collection and transfer into the spreadsheet.

Data Collection on Concept Notes Submitted for Adaptation and Cross-Cutting Projects:

Data were collected from PDF files of Concept Notes, accessed via the GCF Publications and Documents platform, and collated into a spreadsheet.

The GCF Publications and Documents platform is accessible online from the GCF Website following the Prompts: https://www.greenclimate.fund/ > Publications & Documents > Operational Documents. The filter functionality was used to filter projects by 'Type' [Concept Notes] and 'Date' [2021].

PDF files of the Concept Notes were downloaded from each of the relevant filtered projects, reviewed individually, and key data points were transferred into a spreadsheet, including:

Project Name

Project Region

Total GCF Financing

Percentage of Financing for Adaptation Elements of the Project

Percentage of Finance for 'Health, Food, and Water Security' Elements of the Project

Project objective described in Section A.19. Project/Programme rationale, objectives and approach of programme/project of the Concept Note document

Transfer of data were validated by two peers, to ensure that no human error was introduced during the process of data collection and transfer into the spreadsheet.

Other Data Collected for Additional Analysis

In addition to the two sub-indicators above, data were collected on projects which received approval for Project Preparation Funding in 2021, and projects which received approval for Readiness Support in 2021. The methodology for collection of these additional data corresponds to the methodology for the collection of data on Concept Notes, with the slight change that the project 'Type' filter was adjusted for the respective types of documents.

Data Analysis:

Calculating GCF Funding Approved for Adaptation Projects:

This figure represents the sum of 'Total GCF Financing Approved' for projects which:

Had funding approved in 2021 AND

Had 100% financing approved for Adaptation Elements in section A.4 Result Area(s) of the Approved Funding Proposal

Calculating GCF Funding Approved for Adaptation Elements of Cross-Cutting Projects

This figure applies to projects which:

Had funding approved in 2021 AND

Had >0% AND <100% financing approved for Adaptation Elements in section A.4 Result Area(s) of the Approved Funding Proposal, with the remaining funding approved for Mitigation Elements

Financing for adaptation elements for each of these projects was calculated as:

Financing for Adaptation Elements per Project = "Total GCF Financing Approved" * "Percentage of Financing for Adaptation Elements" in section A.4 Result Area(s)

Total GCF Funding Approved for Adaptation Elements of Cross-Cutting projects represented the sum of Financing for Adaptation Elements per Project.

Calculating GCF Funding Approved for Adaptation Projects with Health Co-Benefits

This figure applies to projects which:

Had funding approved in 2021 AND

Had >0% financing approved for Adaptation Elements in section A.4 Result Area(s) of the Approved Funding Proposal, consequently either representing 'Adaptation' projects of 'Cross-Cutting' Projects AND

Had elaborated a metric for GCF Outcome A2.0: Increased resilience of health and wellbeing within the Logical Framework of the Project Approval Document AND

Had indicated GCF Contribution towards Increased Resilience of Health and well-being, and food and water security in section A.4 Result Area(s) AND

Did not identify ‘Health System Resilience’ as the primary objective of the project in Section B.3. Project/Programme Description

Funding directed towards Health Co-Benefits for adaptation elements of these projects was calculated as:

Financing for Health Co-Benefits of Adaptation Elements per Project = “Total GCF Financing Approved” * “Percentage of Financing for Adaptation Elements” in section A.4 Result Area(s) * “Percentage of Financing for Increased Resilience of Health and well-being, and food and water security in section A.4 Result Area(s)”

Total GCF Funding Approved for Adaptation Projects with Health Co-Benefits represented the sum of Financing for Health Co-Benefits of Adaptation Elements per Project.

Calculating GCF Funding Approved for Projects Addressing Health System Adaptation

This figure applies to projects which:

Had funding approved in 2021 AND

Had >0% financing approved for Adaptation Elements in section A.4 Result Area(s) of the Approved Funding Proposal, consequently either representing ‘Adaptation’ projects of ‘Cross-Cutting’ Projects AND

Had elaborated a metric for GCF Outcome A2.0: Increased resilience of health and wellbeing within the Logical Framework of the Project Approval Document AND

Had indicated GCF Contribution towards Increased Resilience of Health and well-being, and food and water security in section A.4 Result Area(s) AND

Did identify ‘Health System Resilience’ as the primary objective of the project in Section B.3. Project/Programme Description

Funding directed towards health system adaptation elements of these projects was calculated as:

Financing for Health System Adaptation per Project = “Total GCF Financing Approved” * “Percentage of Financing for Adaptation Elements” in section A.4 Result Area(s) * “Percentage of Financing for Increased Resilience of Health and well-being, and food and water security in section A.4 Result Area(s)”

Total GCF Funding Approved for Adaptation Projects with Health Co-Benefits represented the sum of Financing for Health System Adaptation per Project.

Calculating GCF Funding Value of Concept Notes Addressing Health System Resilience

This figure applies to projects which:

Had a Concept Note submitted in 2021 AND

Had >0% financing requested in the Concept Note for Adaptation Elements in section A.4 Result Area(s), consequently either representing ‘Adaptation’ projects of ‘Cross-Cutting’ Projects AND

Had indicated GCF Contribution towards Increased Resilience of Health and well-being, and food and water security in section A.4 Result Area(s) AND

Did identify ‘Health System Resilience’ as the primary objective of the project in Section A.19. Project/Programme rationale, objectives and approach of programme/project

Funding requested for health system adaptation elements of these projects was calculated as:

Funding Requested for Health System Adaptation per Project = “Total GCF Financing Requested” * “Percentage of Financing for Adaptation Elements” in section A.4 Result Area(s) * “Percentage of Financing for Increased Resilience of Health and well-being, and food and water security in section A.4 Result Area(s)”

Total funding requested for health system adaptation represented the sum of Funding Requested for Health System Adaptation per Project.

Data

Adaptation and Resilience to Climate Change dataset from kMatrix Ltd, in partnership with University College London.[174]

Green Climate Fund Portfolio Dashboard[178]

Climate Funds Update Data Dashboard[179]

Caveats

Economic activity or transactions are only measured where there is an economic ‘footprint’, i.e., where there is transactional/financial data available to be measured. Therefore, public sector spending without an economic ‘footprint’ (government spending on salaries, for example), cannot be measured. It also not possible to directly identify what percentage of measured spending is public versus private. Values are not currently adjusted for inflation. Values of sales generated are not directly comparable with values derived from national statistics.

The reference period is the financial years 2015/16 to 2020/21.

This indicator provides in-depth analysis of funding approved and concept-notes received by the GCF in 2021 for Health Adaptation projects.

According to the data dashboard of the Climate Funds Update (CFU), the GCF accounts for 53.45% of total global multilaterally governed funds approved focused on climate change from January 1 2003, to December 31 2021.

While this likely represents a good indicator of Climate Change funding trends, it is possible that other Funds show a different trend. This risk has been mitigated through analysis of total climate change funding approved in 2021 using the CFU data dashboard using methodologies from the 2021 *Lancet* Countdown Report, which identified similar trends across funds.

Moreover, this indicator is limited through reliance on manual data-transfer from the GCF website into a spreadsheet. In future years, the ambition is to work with Climate Change funds, starting with the Green Climate Fund, to consistently collect data on funding approved for Health Adaption which will improve the data quality of this indicator.

Future form of the indicator

Further historical data could be available in the future.

Additional analysis

Within approved Project Preparation Funding: In 2021, of the 11 potential adaptation projects that received preparation funding, only three focused on health system adaptation (11% (USD$79 million) of the total).

Indicator 2.2.5: Detection, Preparedness and Response to Health Emergencies

This indicator takes data from the International Health Regulations (IHR (2005)) State Party Self-Assessment Annual Reporting Tool (SPAR).

Under the IHR (2005) all States Parties are required to have or to develop minimum core public health capacities to implement the IHR (2005) effectively. IHR (2005) also states that all States Parties should report to the World Health Assembly annually on the implementation of IHR (2005). In order to facilitate this process, WHO developed an IHR Monitoring questionnaire, interpreting the Core Capacity Requirements in Annex 1 of IHR (2005) into 20 indicators for 13 capacities. Since 2010, this self-reporting IHR monitoring questionnaire is sent annually to National IHR Focal Points (NFPs) for data collection. It contains a checklist of 20 indicators specifically developed for monitoring the development and implementation of 13 IHR capacities. The method of estimation calculates the proportion/percentage of attributes (a set of specific elements or functions which reflect the level of performance or achievement of a specific indicator) reported to be in place in a country.

The core capacities to implement the IHR (2005) have been established by a technical group of experts, as those capacities required to detect, assess, notify, and report events, and to respond to public health risks and emergencies of national and international concern. To assess the development and strengthening of core

capacities, a set of components are measured for each of the core capacities, by considering a set of one to three indicators that measure the status and progress in developing and strengthening the IHR core capacities. Each indicator is assessed by using a group of specific elements referred to as 'attributes' that represents a complex set of activities or elements required to carry out this component. The annual questionnaire has been conducted since 2010 with a response rate of 72% in 2012, 66% in 2016 and 85% in 2017, and 100% of countries reporting at least once since 2010. Annual reporting results are complemented by after action reviews, exercises, and joint external evaluation (JEE).

At the beginning of 2018, in compliance with the recommendations of the IHR Review Committee on Second Extensions for Establishing National Public Health Capacities and on IHR Implementation and following formal global consultations with States Parties held in 2015, 2016, and 2017, and 2018, the WHO Secretariat replaced the IHR Monitoring questionnaire by the "IHR State Party Self-assessment Annual Reporting (SPAR) Tool". This has strong implication for the future of this indicator: preparedness and response capacities have now been merged into one capacity called "C8: National health emergency framework"; one capacity relevant to climate adaptation and resilience has been added ("C9: Health services provision"); and capacity grading has been introduced, which requires countries to grade their capacity indicators in progressive levels from 0 to 5 as opposed to the previous "Yes/No/Not know" answers options. C8 contains three components. A full breakdown of the 0–5 scale for each of the three components is provided in the 2019 *Lancet* Countdown report appendix.

To obtain an implementation rating, data were classified according to the table below:

**Level of Implementation Classification**	**Score**
Low	0–24%
Medium-Low	25–49%
Medium-High	50–74%
High	75–100%

Table 40 Categorisation of 'Level of Implementation' of Core Capacity 8 of the IHR SPAR tool

Data

1. International Health Regulations (2005) Annual Reporting. Data is available through the Global Health Observatory Data Repository for 2010-2017, and through the SPAR interactive for 2021.

Caveats

There are some limitations to considering these capacities as proxies of health system adaptive capacity and system resilience. Most importantly, IHR monitoring questionnaire responses are self-reported. Secondly, the countries that report IHR implementation differ from year to year within these regional aggregate scores. Thirdly, IHR Core Capacity Requirements are not specific to climate change, and hence whilst they provide a proxy baseline, they do not directly measure a country's adaptive capacity in relation to climate driven risk changes. Fourthly, these findings capture potential capacity – not action. Finally, the quality of surveillance for early detection and warning is not shown and neither is the impact of that surveillance on public health. Response systems have been inadequate in numerous public health emergencies and thus the presence of such plans is not a proxy for their effectiveness. Nevertheless, these capacities provide a useful starting point to consider the potential adaptive capacity of health systems globally.

Future form of the indicator

The World Health Assembly resolution WHA73.1 requested the WHO Director-General to initiate a process of impartial, independent and comprehensive evaluation of the WHO-coordinated international health response to COVID-19, including the mechanisms in place under the IHR. Future forms of this indicator will need to evolve along with the outputs of this review.

Multiple different indices exist which measure different elements of health emergency preparedness. This indicator will be improved in collaboration with the WHO, to identify if any of these complementary indices can be integrated to provide a more holistic evaluation of the capacity of health systems to respond to different types of global health emergencies.

2.3: Vulnerabilities, Health Risk and Resilience to Climate Change

Indicator 2.3.1: Vulnerability to Mosquito-Borne Diseases

Methods

This indicator tracks the vulnerability to serious adverse health outcomes from dengue considering susceptibility and coping capacity variables. The countries included in the indicator are those that have shown environmental risk for transmission, with at least one value of R_0 (as defined in indicator 1.3) above1 in the period of consideration (1990–2019).

Vulnerability is computed by dividing the percentage of urban population (UP) scaled 1 to 100, by the percentage of a proxy of healthcare access and quality (HCAQ) scaled 1 – 1000. HCAQ results from the subtraction of 100 - % of deaths by communicable diseases and maternal, prenatal and nutrition conditions obtained the Global Burden of Disease Study 2019.

$$\boldsymbol{Vulnerability} = \boldsymbol{UP})/\ \boldsymbol{HCAQ}$$

Data

1. Global Burden of Disease Collaborative Network. Global Burden of Disease Study 2019 (GBD 2019) Reference Life Table. Seattle, United States of America: Institute for Health Metrics and Evaluation (IHME), 2021[180]
2. World Bank, World Development Indicators.[181] Urban population (% of total population). Available from: https://data.worldbank.org/indicator/SP.URB.TOTL.IN.ZS

Caveats

Countries with predicted R_0<1 where not considered for this indicator; countries with predicted R_0>1 includes those with tropical and sub-tropical zones. The indicator is extrapolated to country level, no estimations at subnational level to differentiate vulnerability between rural and urban settings have been performed. These aspects should be carefully considered for the interpretations of the results.

Countries that reported a high proportion of urban population, such Singapore with a consistent urban population of 100% across 1990–2019, showed an influence lifting the vulnerability average for the other countries, therefore affecting the results for WHO regions and HDI groups too. In that case, the country was analysed separately to avoid misleading results.

Future form of the indicator

An improved version of this indicator will be developed in the future, incorporating other factors linked to vulnerability to dengue in the literature.

Indicator 2.3.2: Lethality to Extreme Events

Methods

Methods The methodology for this indicator remains similar to that described in the 2020 report of the *Lancet* Countdown.[173] The number of occurrences of weather-related disasters (drought, storms, wildfires, floods and extreme temperatures), the number of people affected in each disaster, and the lethality of these events have however been grouped according to the 2019 HDI level for each country over the period from 1990 to 2020.

The methodology uses data from the Centre for Research on the Epidemiology of Disasters (EM-DAT).[182] Here, deaths, as proxy of the lethality of weather-related disasters, are defined as the number of people who lost their life because the disaster happened. People affected are defined as those requiring immediate assistance during a period of emergency; hence requiring basic survival needs such as food, water, shelter, sanitation and immediate medical assistance.

Data

1. EM-DAT at the Centre for Research on the Epidemiology of Disasters (CRED) at the Université Catholique de Louvain, Belgium[182]
2. Human Development Index (HDI) at the United Nations Development Programme, Human Development Reports[183]

Caveats

The EM-DAT database contains a number of possible biases. Firstly, there is a possible bias in missing some disaster events because of under-reporting. EM-DAT classifies an event as a disaster if 10 or more people die; 100 or more people are affected; there is a declaration of a state of emergency; or a call for international assistance. Similarly, there are likely biases in how countries report both the number of deaths and people affected. Numbers of deaths for example may not include mortality from the cascading risks of natural hazards or those that occur as a result of longer causal chains from the hazard. Secondly, estimates of the numbers of people affected have different biases for different countries because of how the concept of "affected people" is defined. This must be considered when comparing countries.

Additional analysis

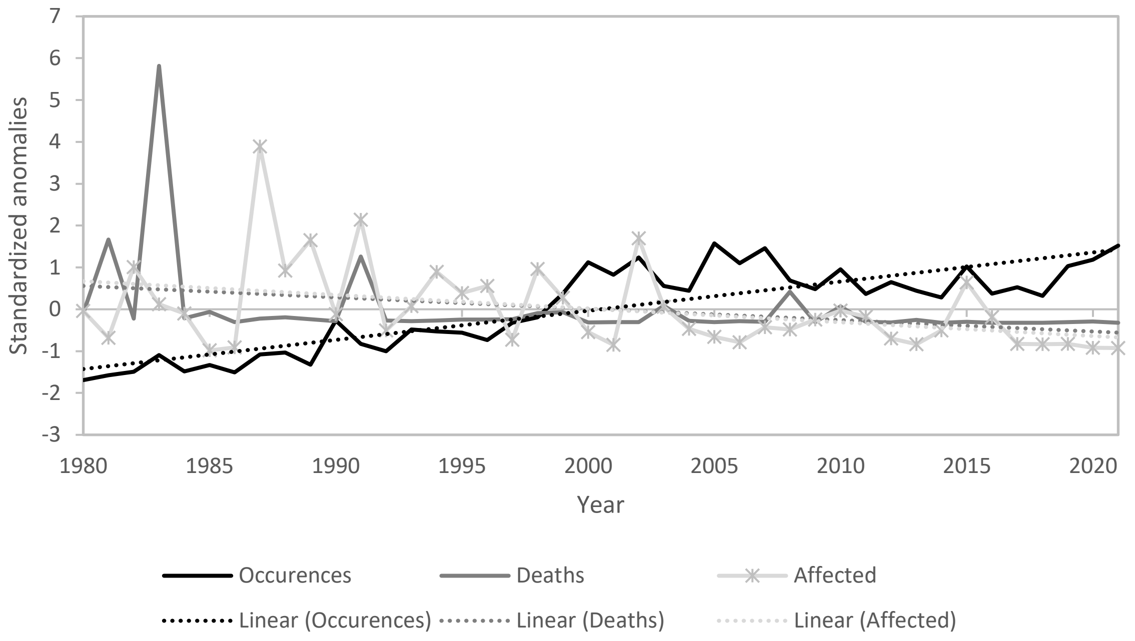

Figure 60: standardised anomalies of the number of extreme events (occurrences), lethality of events (deaths) and number of people affected (affected). The dashed lines represent linear trends, with slopes with the following p-values. Occurrences: p< .00001; deaths p = 0.031, Affected p = 0.008

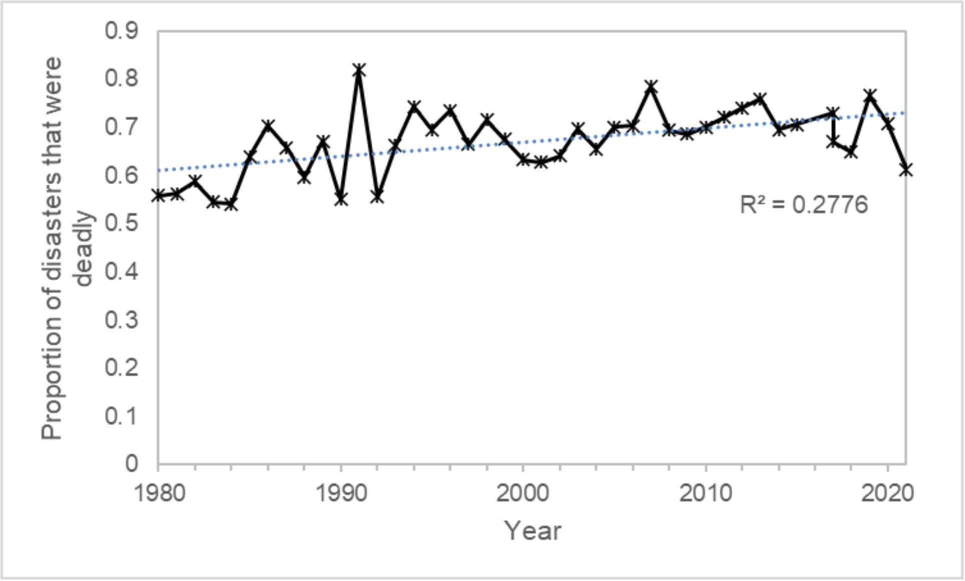

Figure 61: Proportion of all recorded extreme weather events that were deadly

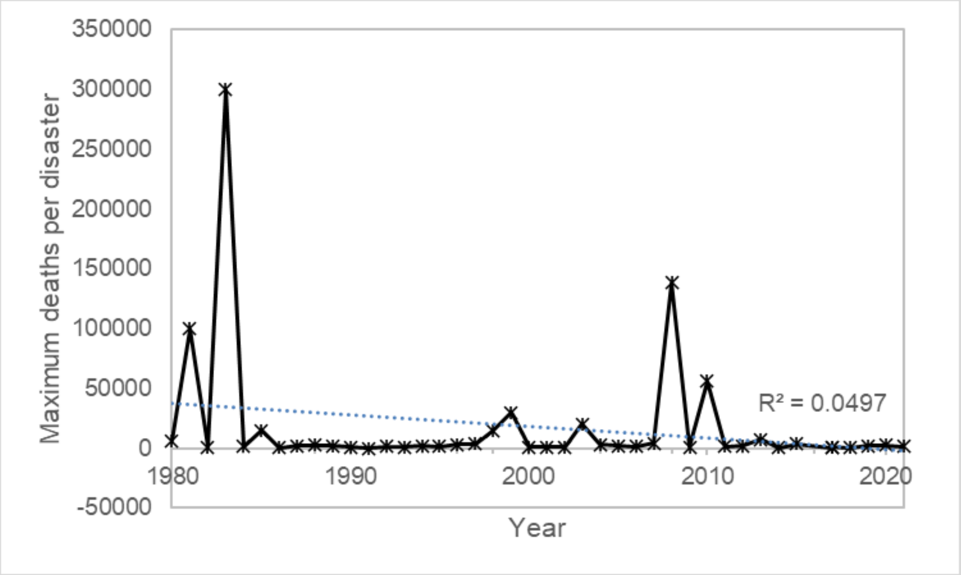

Figure 62: Maximum deaths recorded per extreme weather event

Indicator 2.3.3: Migration, Displacement, and Rising Sea Levels

Population exposure to global mean sea level rise

Methods

The methodology for this indicator remains similar to that described in the 2021 report of the *Lancet* Countdown.[11] By using a bathtub model, this indicator overlays future Global Mean Sea Level Rise (GMSLR) of 1 m with coastal elevation value grid-cells to delineate areas of potential inundation and current global population distribution grid-cells to delineate populations living in areas exposed to absolute GMSLR of 1 m.

In the first step, the Coastal Digital Elevation Model (CoastalDEM) dataset was used to categorise inundated grid-cells under 1m of GMSLR: i.e. 1m of GMSLR. In the second step a gridded population dataset (i.e. Chambers, 2022) was overlaid to estimate population exposure values. These grid-cells were then matched with country boundaries using the Global Administrative Areas (GADM) 3.6 Data Set. Then the grid-cell level data were aggregated to country level (i.e. national population numbers exposed to 1m of GMSLR). Finally, the population exposed to 1m of GMSLR data were overlaid on the sub-national HDI to identify socioeconomic status of populations exposed to GMSLR.

Data

1. GMSLR: Estimated global mean increases in sea-levels[184]
2. Elevation: Coastal Digital Elevation Model (CoastalDEM)[185]
3. Hybrid gridded demographic data for the world[8]
4. Global Administrative Areas (GADM) version 4.0.4, http://www.gadm.org/

Caveats

The global mean sea level rose by 0. 20 [0.15-0.25] m between 1901-2018. For a very high greenhouse gas emission scenario, the global mean sea level is projected to increase up to 1.01 m by 2100 relative to 1995-2014 (IPCC 2021). Due to uncertainty in the Greenland and Antarctic ice sheet melt processes, GMSLR of 2 m by 2100 and 5 m by 2150 cannot be ruled out.[1,186]

Estimates of population exposure to GMSLR vary according to datasets, timeframes, emissions and socioeconomic scenarios, and analytical method.[187] For this indicator, the datasets used, and analytical approach

determine results. CoastalDEM (3-arc second; 90m) is a global coastal digital elevation model that is adjusted to reduce SRTM error.[188] While SLR-related hazards could potentially displace people living in sites of coastal risk, population exposure to SLR is not a proxy indicator for SLR-related population displacement. Displacement can be prevented or forestalled through protection (e.g., armouring coasts) and accommodation (e.g. measures that facilitate living with SLR impacts); some may be unable or unwilling to leave; and people migrate into low-lying coastal sites.[187,189] When protection and accommodation are exhausted or not feasible, retreat from sites of SLR-related risk may occur.

SLR can contribute to health risks including reduced saltwater intrusion of drinking water, food insecurity (e.g., loss or arable land, reduced crop yields), altered infectious disease ecology, and psychosocial impacts.[190,191] For those who retreat, health represents a measure of adaptation. Empirical studies identify diverse consequences of retreat from sites of coastal risk, including for mental health, food security, water supply, sanitation, infectious diseases, injury, and health care access.[192,193]

Future form of the indicator

Plans to improve the methodology, data sources, and/or temporal and geographical coverage of this indicator in subsequent reports

As new, higher spatial resolution and more precise datasets become available, we will update our methods to produce robust estimates of population exposure to future GMSLR.

Additional analysis

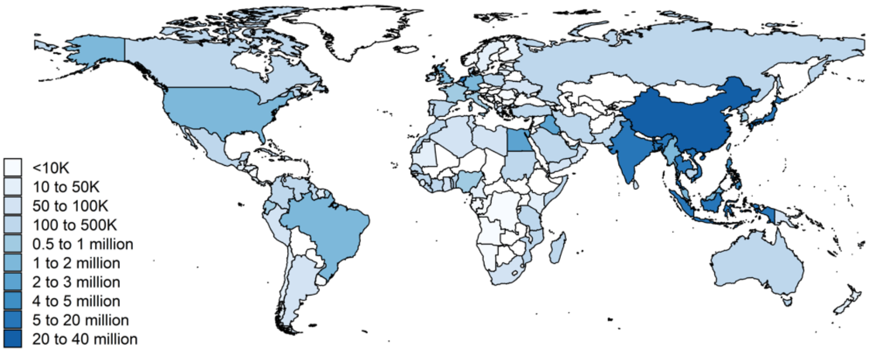

Figure 63 Population exposure to 1m GMSLR

National Policies on Migration

Methods

This component of this indicator on national policies reports:

1a. The number of currently valid national-level policies including legislation for migrants, migration, displacement, displaced people, relocation, and relocated people specifically related to climate change (not climate or disasters), including immobility (trapped populations/non-migration/non-displacement).

1b. The number of such policies mentioning health or well-being along with a qualitative analysis of how health and/or well-being are/is mentioned.

2a. The number of countries with at least one such policy.

2b. The number of such countries whose policies mention health or well-being along with a qualitative discussion of how health or well-being is mentioned.

"Country" refers sovereign state or autonomous non-sovereign territory (not just a sub-national jurisdiction). Multi-lateral, inter-governmental, and international policies are specifically excluded. Explicit mentions of "climate change" and "health" or "well-being" must be present, not implied definitions or references to wider contexts which might (or might not) encompass these points, e.g., "climate", "climate disasters", "humanitarian", and "environment".

The method for identifying national-level policies is:

1. A systematic review, using the keywords which define the indicator
2. Crowd-sourcing and expert queries[44]

Because this search can never know what might have been missed, the numbers reported for this indicator represent minimum counts. Each policy included is also categorised by:
1. (a) Migration/mobility/displacement/relocation from a location,
   (b) migration/mobility/displacement/relocation to a location, and
   (c) immobility/trapped populations.
2. (a) Domestic migration/mobility/displacement/relocation and
   (b) international migration/mobility/displacement/relocation — all immobility, by definition, is domestic

A given policy might be counted in more than one category for 1abc and for 2ab. Some policies do not have an end date, and some do, with both included. Policies which are now out-of-date are retained in a separate list as well as a list of policies considered but not included in this indicator.

Caveats

As documented in previous *Lancet* Countdown reports[11,170-173] and supporting publications,[189,194-199] the main problems with using migration or displacement as a climate change and health indicator are:
1. Attributing movement or immobility to climate change or climate change impacts is not straightforward
2. Attributing health outcomes to movement or immobility is not straightforward

The evidence to back each of these two attribution relationships is currently weak and it is highly debated in the literature whether or not (i) there are or will be links between climate change and migration, displacement, (im)mobility, relocation and (ii) there are or will be links between migration, displacement, (im)mobility, relocation and health/well-being.

This indicator assists in overcoming the attribution problem by:

1. Examining written policies, so attribution is not a concern, because the policies exist, even if attribution is inappropriate
2. Examining how policies mention health/well-being, so again actual attribution is not a concern, because the text on health or well-being either exists or does not exist, even if attribution is inappropriate

If spurious attributions are made in the policies between (i) climate change and migration/displacement/immobility or (ii) migration/displacement/immobility and health or well-being, then this indicator can analyse those attributions and why they might not be defensible, based on the scientific literature. Thus, this indicator provides what is happening at the national level and the appropriateness of these policies in terms of the scientific literature. The key to this approach and to overcoming the caveats is keeping the indicator simple and straightforward, which is why the indicator has been designed in the proposed manner.

Selecting policies, and in particular national policies, does not cover all possibilities, but it serves as an indicator. As well, it is an indicator of how national governments perceive the climate change / (im)mobility / health links, without making a statement on the actual links, which the literature explains is exceptionally difficult. This approach to the indicator also means that misattributions are easily filtered out, such as reporting migration and health links to disasters or climate, both of which are different from links to climate change. Using ‘climate change’ synonymously with ‘climate’, ‘climate-related disasters’, and/or ‘disasters’, is a common mistake in many policies reviewed as well as in the academic literature.

The main caveat is that most of the data is confined to documents in English, with a few other languages on occasion. The advantage is that policies which are not available in English have typically been discussed in English publications, including blogs and news reports, suggesting that much relevant material has been captured. Nonetheless, the numbers reported can only be taken as the minimum, as in ‘at least so many’ policies match the criteria stated. One minor caveat is that the number of countries sometimes changes year-to-year, providing a different baseline. These changes are rarely more than one or two countries per year out of a sample of around 200. Substantial changes to the numbers of countries will be reported if this occurs.

Future Form of the Indicator

Plans to improve the methodology, data sources, and/or temporal and geographical coverage of this indicator in subsequent reports.

The indicator design helps in overcoming these caveats by reporting that the counts provided must be only minimum numbers, because we cannot know what we would have missed. Through publicity, publication, crowd sourcing, and expert connections, this limitation will be overcome because people will provide examples of what we missed. As an indicator, it is important to accept that the numbers are not comprehensive but provide only minimum numbers as a lower-bound baseline.

Section 3: Mitigation Actions and Health Co-Benefits

3.1: Energy System and Health

Indicator 3.1.1: Carbon Intensity of the Energy System

Methods

This indicator contains two components:

- Carbon intensity of the energy system, both at global and regional scales, (1971–2019), in tCO_2/TJ
- Global CO_2 emissions from energy combustion by fuel, in $GtCO_2$ (1972–2019). Global emissions without fuel breakdown are also provided for 2020 and provisionally for 2021

The technical definition is the tonnes of CO_2 emitted for each unit (TJ) of primary energy supplied.

The rationale for the indicator choice is that carbon intensity of the energy system will provide information on the level of fossil fuel use, which has associated air pollution impacts. Higher intensity values indicate a more fossil dominated system, and one that is likely to have a higher coal share. As countries pursue climate mitigation goals, the carbon intensity is likely to reduce with benefits for air pollution.

The indicator is calculated based on total CO_2 emissions from fossil fuel combustion divided by Total Energy Supply (TES). TES reflects the total amount of primary energy used in a specific country, accounting for the flow of energy imports and exports.

The data is available for most countries of the world, for the period 1971–2019.

Data

1. This indicator is based on based on the IEA dataset, CO_2 Emissions From Fuel Combustion: CO_2 Indicators, accessed via the UK data service,[200] and supplemented with additional data for 2020[201] and 2021[202]

Caveats

IEA data are generated using both direct input from national governments and modelling. As such, while they represent the best available data on national CO_2 emissions from fuel, they are subject to caveats which vary by energy commodity and country. Full details are given in the CO_2 Emissions From Fuel Combustion documentation.[203]

The IEA updated its methodology in late 2021 to include additional industrial sources of CO_2 in their emissions estimates, as well as GHG emissions sources.[204] These additions are not presently available at country level.

Additional analysis

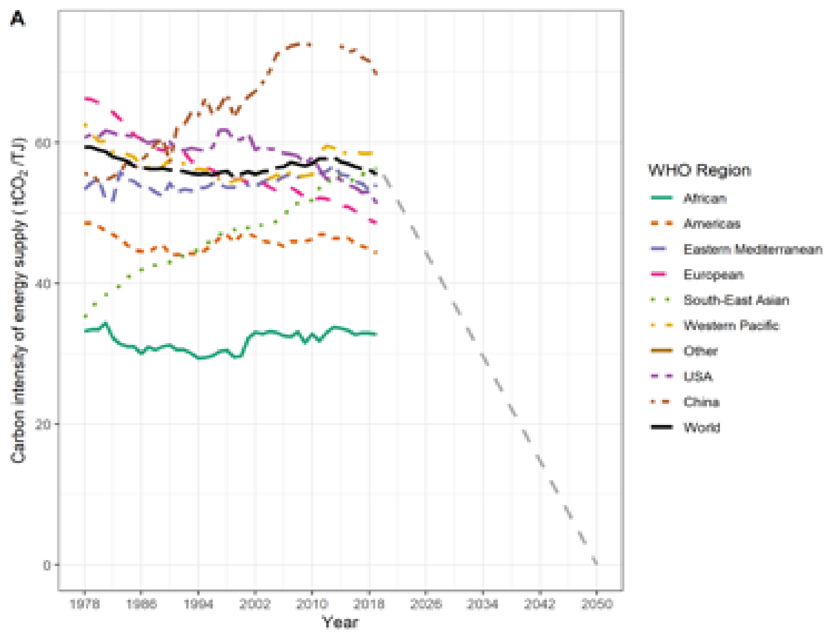

Figure 64: global carbon intensity of energy use by WHO region. Linear trajectory towards net-zero 2050 also shown.

Indicator 3.1.2: Coal Phase-Out

Methods

Two indicators are used here:

1. Total primary coal supply by region / country (in exajoules, EJ);
2. Share of electricity generation from coal (% of total generation from coal) and global generation from coal (in TWh)

These indicators are important to enable tracking of changes in coal consumption at a regional and country level. Due to the level of coal used for power generation, a second indicator tracks the contribution to electricity generation from coal power plants in selected countries. As countries pursue climate mitigation goals, the use of coal is likely to reduce with resulting benefits for air pollution.

The indicator on primary energy coal supply is an aggregation of all coal types used across all sectors (from the IEA energy balances). The data are available for most countries of the world, for the period 1978–2019.

The indicator on the share of electricity generation from coal is estimated based on electricity generated from coal plant as a percentage of total electricity generated. Regional data are available from 1990–2019; pre-1990 data are not used due to incomplete time series.

Countries or regions with large levels of coal use (as a share of generation, or in absolute terms), have been selected to show in the figures.

The following types of coal are added to produce the total primary coal supply:

‘Anthracite’, ‘Coking coal’, ‘Lignite’, ‘Other bituminous coal’, ‘sub-bituminous coal’

Data

1. This indicator is based on the extended energy balances from the International Energy Agency. The specific dataset is called World Extended Energy Balances (for 2021), and is sourced via the UK data service.[205]

Caveats

IEA data are generated using both direct input from national governments and modelling. As such, they are subject to caveats which vary by energy commodity and country. Full details are given in the IEA World Energy Balances documentation.[206] This documentation also covers changes to methodology in previous editions of IEA World Energy Balances. A typical example of the way data can be impacted by methodology updates by reporting countries is as follows, relating to Belgium 'New data on consumption cause breaks in time series for primary solid biofuels between 2011 and 2012'. However, since data are aggregated, the impacts on overall trends is minimal.

Additional analysis

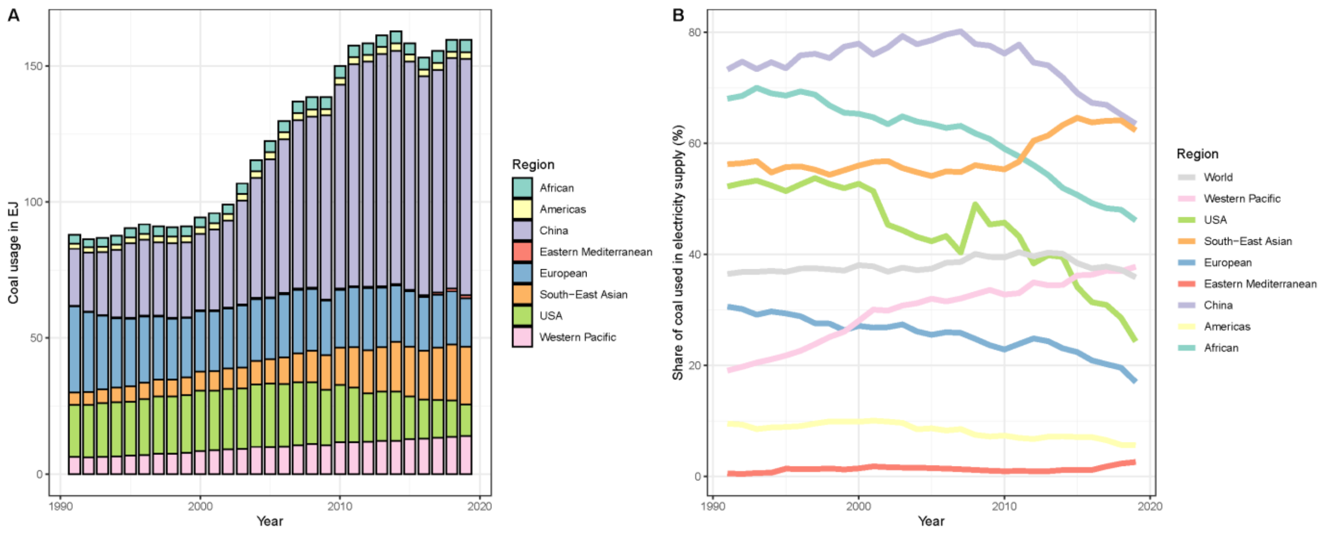

Figure 65. Panel A: TES of coal in selected countries and WHO regions, 1990-2019. Panel B: Share of coal used in electricity in selected countries and WHO regions, 1990-2019

Indicator 3.1.3: Zero-Carbon Emission Electricity

Methods

Two indicators are used here, and presented in two ways:

1. Total low carbon electricity generation, in absolute terms (TWh) and as a % share of total electricity generated (to include nuclear, and all renewables); and
2. Total renewable generation (wind and solar), in TWh, and as a % share of total electricity generated

The increase in the use of low carbon and renewable energy for electricity generation will push other fossil fuels, such as coal, out of the mix over time, resulting in an improvement in air quality, with benefits to health.

The renewables (wind and solar) indicator has been used to allow for the tracking of rapidly emergent renewable technologies. For both indicators, generation, rather than capacity, has been chosen as a metric as the electricity generated from these technologies is what actually displaces fossil-based generation. Countries with large levels of low carbon generation (as shares, or in absolute terms), or with higher fossil dependency, have been selected.

The data are taken from the IEA extended energy balances.[205] The absolute level indicators are total gross electricity generated aggregated from the relevant technology types. The share indicators are estimated as the low carbon or renewable generation as a % of total generation.

The data are available for most countries of the world, for the period 1971–2019. Only the period from 1990 has been used, due to data gaps for selected countries prior to 1990.

The following IEA variable names are added to produce total low carbon electricity generation:

‘Nuclear, ‘Hydro’, ‘Geothermal’, ‘Solar photovoltaics’, ‘Solar thermal’, ‘Tide, wave and ocean’, ‘Wind’

The following IEA variable names are added to produce total renewable electricity generation:

‘Geothermal’, ‘Solar photovoltaics’, ‘Solar thermal’, ‘Tide, wave and ocean’, ‘Wind’

Data

1. This indicator is based on the extended energy balances from the International Energy Agency. The specific dataset is called World Extended Energy Balances, and is sourced via the UK data service (http://stats.ukdataservice.ac.uk/).[205]

Caveats

IEA data are generated using both direct input from national governments and modelling. As such, they are subject to caveats which vary by energy commodity and country. Full details are given in the IEA World Energy Balances documentation.[206] This documentation also covers changes to methodology in previous editions of IEA World Energy Balances. A typical example of the way data can be impacted by methodology updates by reporting countries is as follows, relating to Belgium ‘New data on consumption cause breaks in time series for primary solid biofuels between 2011 and 2012’. However, since data are aggregated, the impacts on overall trends is minimal.

Additional analysis

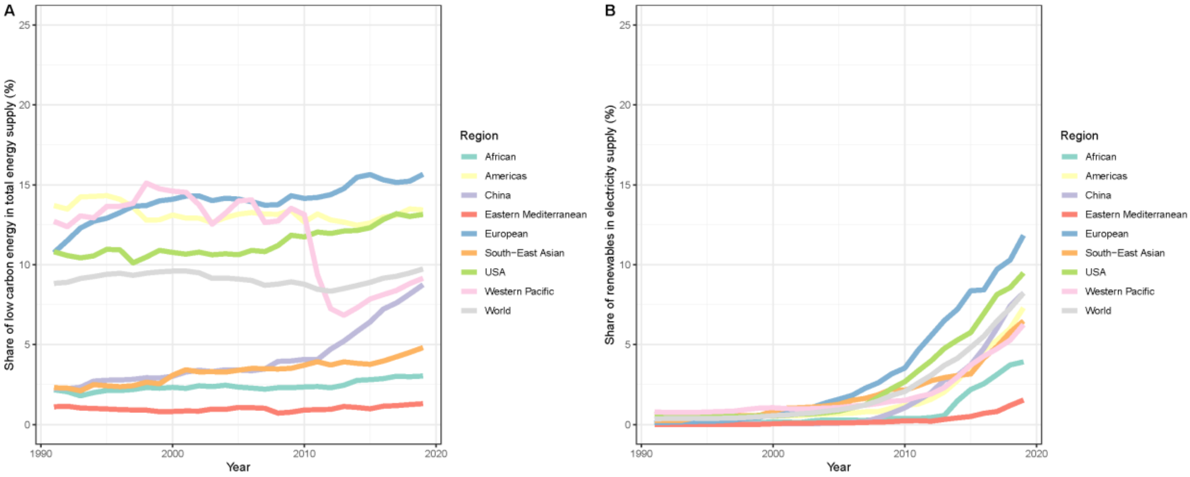

Figure 66. A) Share of total energy supply provided by low carbon energy sources by WHO region. B) Share of electricity generation provided by modern renewables (wind, solar and geothermal) by WHO region

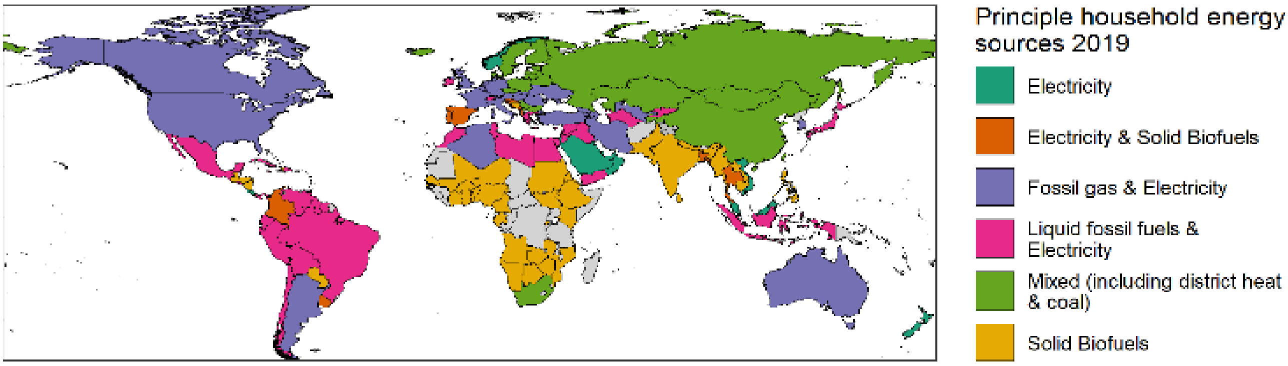

Figure 67: Principal household energy sources in 2019.

Indicator 3.2: Clean household energy

Use of clean fuels in the domestic sector

The 2022 report presents a combination of data from the WHO (which feeds into the Sustainable Development Goal 7) and fuel consumption in the residential sector produced by the International Energy Agency (IEA).

Access to clean energy is defined by the IEA (2020) as:

"a household having reliable and affordable access to both clean cooking facilities and to electricity, which is enough to supply a basic bundle of energy services initially, and then an increasing level of electricity over time to reach the regional average".[207]

Within SDG 7.1.2 (proportion of population with primary reliance on clean fuels and technology) “Clean” fuels are defined by emission rate targets and specific fuel recommendations included in the WHO guidelines for indoor air quality: household fuel combustion.[208]

This indicator is modelled with household survey data compiled by WHO[209], which uses Bayesian methods to impute yearly estimates of primary reliance on clean fuels and technologies for cooking, heating and lighting, using survey-based estimates between 2000 and 2017 and modelling projections for 2018 and 2019.[210,211].

The use of energy in the residential sector is drawn from the IEA extended global residential modelling produced in the World Energy Outlook from the ‘World Extended Energy Balances’ 2021 edition, which covers all countries or major regions in the world.[212] The values are measured in EJ and cover all fuels supplied for consumption within the residential sector (IEA flow code QGFLOW076) final energy demand.

The specific IEA variables were combined in the following way:

`Solid biofuels` = Charcoal + `Primary solid biofuels`

`Coal, coke and peat` = `Hard coal (if no detail)` + BKB + `Petroleum coke` + `Patent fuel`+`Coke oven coke`+`Brown coal (if no detail)`+Peat+`Gas coke`+`Peat products`+`Coking coal`+`Sub-bituminous coal`+`Other bituminous coal`+Lignite+Anthracite+Bitumen

`Other biofuels` = `Other liquid biofuels` + Biogasoline + `Non-specified primary biofuels and waste`+`Biogases`+`Biodiesels`

`Liquid fossil fuels` =`Paraffin waxes`+`Other oil products`+`Naphtha`+ `Gas/diesel oil excl. biofuels` + Lubricants + `Natural gas liquids`+ `Other kerosene`+`Liquefied petroleum gases (LPG)`+`Fuel oil`+`Motor gasoline excl. biofuels`+`Crude oil`

`Waste & other` = `Municipal waste (non-renewable)`+`Municipal waste (renewable)`+`Industrial waste`+`Refinery gas`+ `Blast furnace gas`+`Gas works gas`+`Coke oven gas`+`Oil shale and oil sands`,

Finally, Natural gas, Heat, Solar thermal, Geothermal and Electricity variables were provided directly from IEA flow QGFLOW076.

The visualisation accompanying this indicator shows the principal household energy sources by country. The full breakdown of the fuels used in these regions is given in **Error! Reference source not found.**.

**Principle household energy sources**	**Heat**	**Liquid fossil fuels**	**Solid biofuels**	**Natural gas**	**Coal, coke and peat**	**Electricity**
Mixed including district heat & coal	27.3%	4.2%	18.9%	12.6%	10.9%	25.1%
Fossil Gas & electricity	4.1%	7.6%	10.5%	52.5%	0.8%	24.5%
Solid biofuels	0.5%	4.2%	80.9%	2.2%	0.2%	11.8%
Liquid fossil fuels & electricity, some biomass	0.4%	38.2%	16.9%	4.9%	1.1%	36.7%
Electricity	0.1%	14.2%	9.2%	4.0%	0.3%	70.2%

Table 41 The mean shares of household energy source by regional type

Data

1. Healthy fuels for cooking were provided by the WHO.[209-211]
2. The additional energy usage and access is based on data from the IEA World Energy Balances 2021.[212]

Caveats

The data from the IEA on residential energy flows and energy access provide an indication of both the access to electricity and the proportion of the different types of energy used within the residential sector. These provide an important picture on how access and use might be interacting.

IEA data are generated using both direct input from national governments and modelling. As such, they are subject to caveats which vary by energy commodity and country. Full details are given in the IEA World Energy Balances documentation.[206] This documentation also covers changes to methodology in previous editions of IEA World Energy Balances. A typical example of the way data can be impacted by methodology updates by

reporting countries is as follows, relating to Belgium 'New data on consumption cause breaks in time series for primary solid biofuels between 2011 and 2012'. However, since data are aggregated here by HDI level, the impacts on overall trends is minimal.

Future form of the indicator

The WHO are in the process of updating the household energy survey database which underpins this indicator. Future forms of the indicator may be able to be coupled more directly with the negative health outcomes related to the use of dirty fuels in the home.

Additional analysis

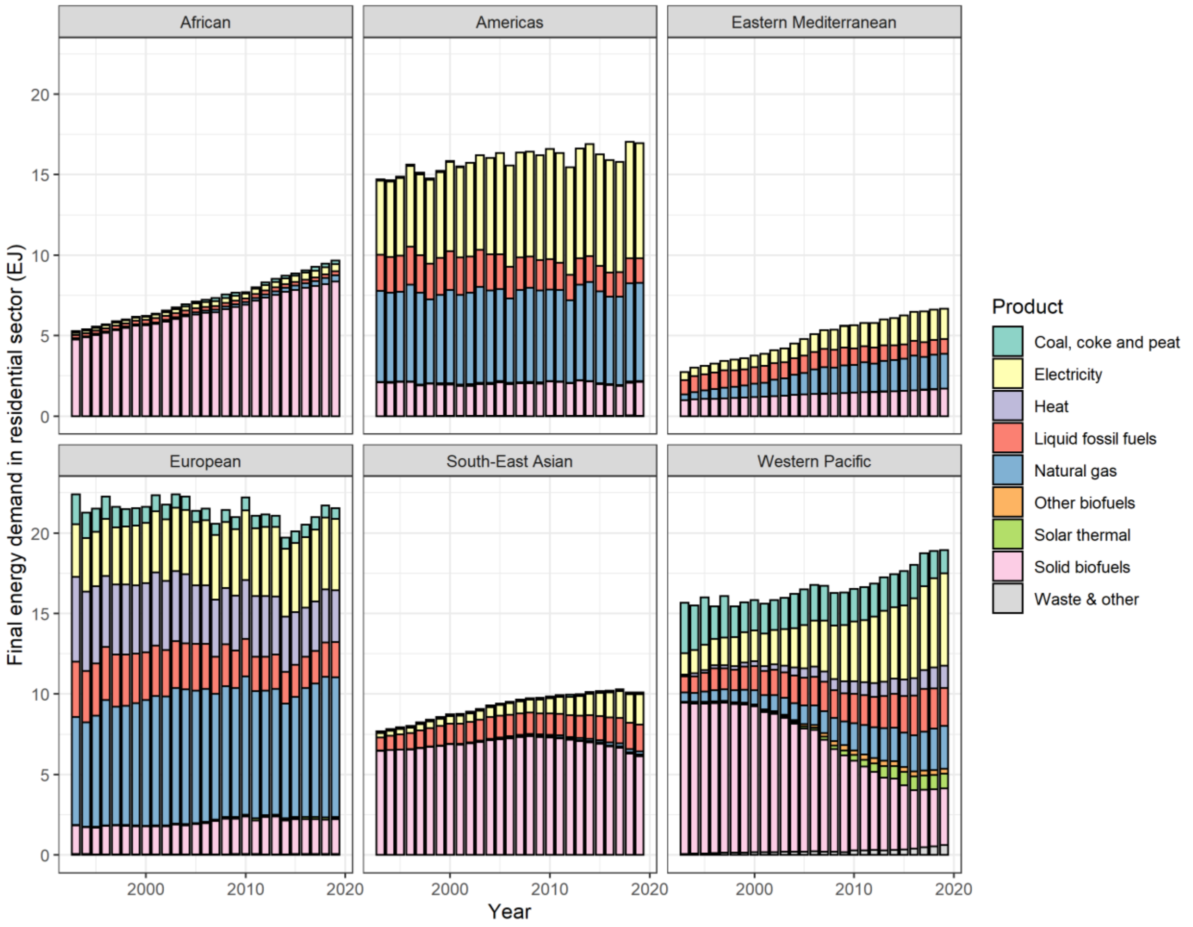

Figure 68. Total energy use in the residential sector by WHO Region and product (EJ).

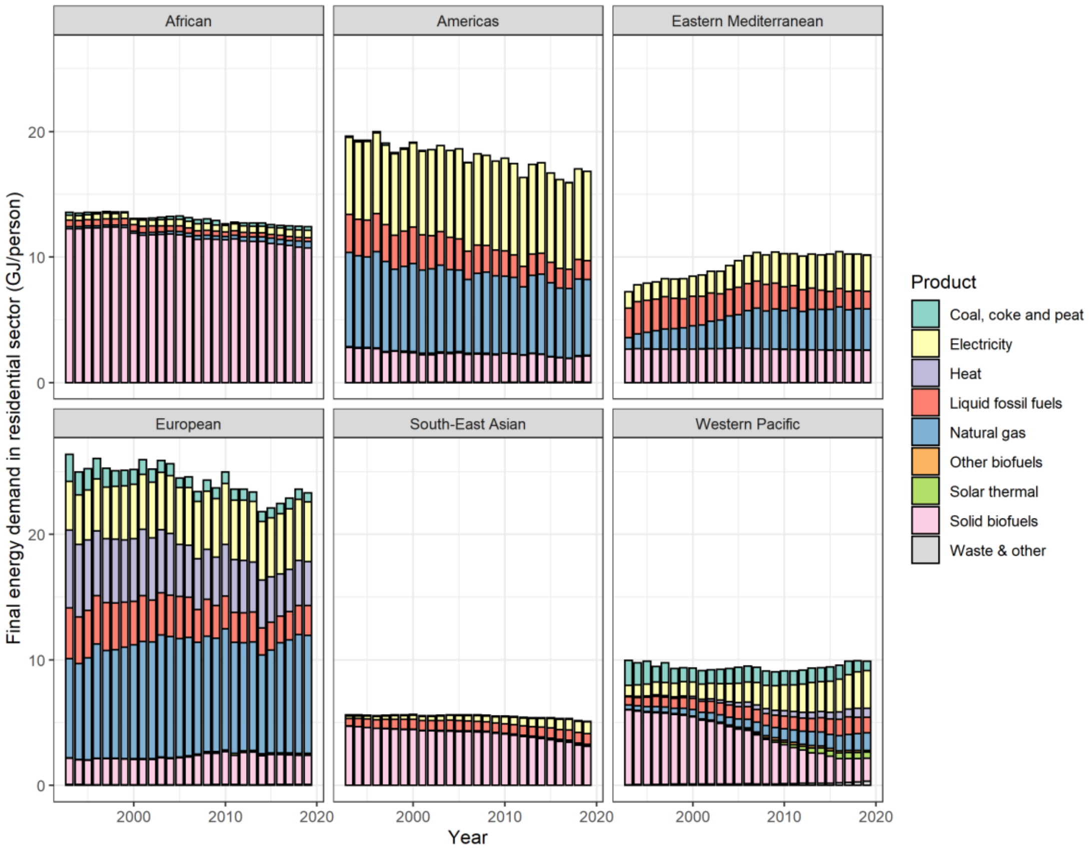

Figure 69 Per capita energy use in the residential sector by WHO Region and product (GJ/person)

Household air pollution

Methods

Existing estimates of global household air pollution attributable mortality from GBD and WHO are based on information on the frequency of use of different fuels in the population. These are presented relative to the outdoor air pollution estimates (e.g. the additional mortality caused by household fuels above that caused by outdoor air pollution). The new indicator complements this work via a method tailored for the Lancet Countdown process which can 1) link the health effects of household fuels to their role in climate change accounting for the GHG and $PM_{2.5}$ emissions, and 2) complement how outdoor air pollution mortality is estimated in the Countdown by using the same inputs, and 3) be updated yearly.

A Bayesian hierarchical $PM_{2.5}$ exposure model was developed using sample data of personal exposure from an updated World Health Organization Global HAP database,[213] [209] while wood, crop residues, and dung is combined into the category of 'biomass' and LPG, Natural gas, and biogas into category of 'gas'.[214] Variables were selected from monitored data available in 282 peer-reviewed studies covering the years 1996 to 2021 to develop Bayesian models for the personal $PM_{2.5}$ exposure (sample size, n = 260). Bayesian hierarchical models were built to generate accurate $PM_{2.5}$ exposure coefficients and variance around the estimates from the sample data and apply to IIASA GAINS modelled data for predicting $PM_{2.5}$ personal exposure globally. This model provides estimates on $PM_{2.5}$ personal exposure levels based on average 24-hour period.

The hierarchical model incorporating the following predictors for each country:
(i) fuel types (biomass, charcoal, coal, gas, electricity),
(ii) traditional/improved stove,

(iii) urban/rural location,
(iv) population weighted heating degree days,
(v) population weighted ambient $PM_{2.5}$,
(vi) GNI index,
(vii) Education index
(viii) season (winter/summer/whole year)

The model resulted in a Bayesian R^2 of 0.67 for personal exposure (µg/m^3). Annual average $PM_{2.5}$ personal exposure were estimated for 71 countries in five WHO regions (African Region, Eastern Mediterranean Region, Region of the Americas, South-East Asian Region, Western Pacific Region). We exclude the European Region due to high uncertainty in the estimated exposure values. The model estimates that members of households using solid fuels for cooking and heating experience an average personal $PM_{2.5}$ exposure of 150 µg/m^3 globally in 2020 (168 µg/m^3 in rural households and 91 µg/m^3 in urban dwellings).

Attributable premature mortality due to personal exposure is estimated at national level (per 100,000 population) using the standard comparative risk assessment (CRA) approach. This involves calculation of population attributable fractions based on the estimated $PM_{2.5}$ personal exposure for each country (and separately for urban and rural populations), assuming that, on average, 60 % of the time is spent indoor house.[213,215] This exposure is then converted into an estimate of excess deaths using Global Burden of Disease functions. In order to compare the results with the WHO estimates[1], we use three following weighted averages to quantify the mortality rates for the number of attributable deaths per 100,000 individuals for solid fuels at national level: (i) Proportion of people using each fuel type (biomass, charcoal, coal, gas, electricity) in each country and for urban and rural settings.[214] (ii) Proportion of people using each stove type (traditional, improved) in each country for urban and rural settings. (iii) Proportion of people living in urban and rural setting in each country.

Exposure to the above concentrations would have resulted in 81 deaths per 100 000 in rural settings and 65 deaths per 100 000 in urban settings. However, given possible increases in the amount of time people spent indoors during COVID-19-related restrictions, these values might have been even higher during 2020.[216]
The household air pollution model includes ambient $PM_{2.5}$ exposure from GAINS as an input. The mortality estimates currently include some degree of overlap with estimates of mortality due to ambient air pollution, which is also the case for the WHO estimates.[217]

Data

- Ambient $PM_{2.5}$ concentrations for 2020 from IIASA[218].
- Fuel Type: IIASA GAINS model via IEA [216].
- Stove Type [219]
- Heating Degree Days for the year 2000 (1985-2015) provided by NASA [220].
- Education index and GNI index provided by United Nations Development Programme (UNDP). Year 2019 [221].
- WHO Global HAP Database [209] .
- WHO. Household air pollution attributable death rate (per 100 000 population) [222].
- WHO. Proportion of population with primary reliance on fuels and technologies for cooking, by fuel type (%) [208]
- Baseline mortality data: GBD national estimates for males and females[223].[224]
- Exposure-response functions for attributable premature mortality: GBD2019 MR-BRTs, cause, and age specific, for 6 diseases.[225]

Caveats

The indicator provides useful information as to the variation of $PM_{2.5}$ exposure for a given fuel use and stove type and urban/rural locations as well as their health impacts. The inclusion of ambient air pollution for urban and rural locations (obtained from IIASA GAINS modelled gridded data) and the heating degree days for the same urban and rural areas are the two unique predictors used here for the first time in Bayesian $PM_{2.5}$ exposure models at global scale.

One challenge is the combination/overlap of ambient $PM_{2.5}$ and household $PM_{2.5}$ exposure, which may lead to double counting mortality.

Indoor air pollution is complex and impacted by a number of different factors including housing characteristics (e.g. ventilation rate, kitchen locations, window in kitchen, roofing materials) which are not typically captured in all the monitored data. Updating the sample data with information on these and related factors should greatly improve the future predictions as to household air pollution.

Another challenge concerns the measured/monitored household air pollution data (e.g. studies included in the WHO database). More specifically, the concerns are as follows: rather limited number of households monitored in each study; each study uses different monitoring technology to collect the data; and data collected from different measurement periods as well as different analytic methods used for data processing in each study. Nevertheless, using Bayesian predictive models developed in this study allows us to explore a wide range of $PM_{2.5}$ exposures depending on fuel use, stove types, and for differences urban and rural locations of countries worldwide.

As regards COVID-19, several studies reported the increase in indoor exposure to $PM_{2.5}$ during the lockdown particularly in households located in rural areas.[226,227] This could potentially have negative impact on human health. Further data from the year 2020 are needed to estimate accurately the exposure and the health impact of global COVID-19 lockdowns, especially in rural areas where the use of solid fuels is very common.

Future form of the indicator

The availability of monitored household air pollution data from around the world is continuously growing and improving, and we will continually update our current database with recent available monitored data to update the Bayesian models based on the new information. This helps us to estimate the $PM_{2.5}$ exposure and related health impacts for those missing regions/countries in the current study.

We will link the health effects of household fuels to their role in climate change accounting for the GHG and $PM_{2.5}$ emissions. Furthermore, we intend to provide evidence as to whether switching to cleaner fuels may (or may not) lead to a substantial reduction in GHG emissions.
As data on fuel use during the COVID-19 pandemic become available in future years, the estimates for exposure to indoor air pollution in 2020 will be further refined

Additional analysis

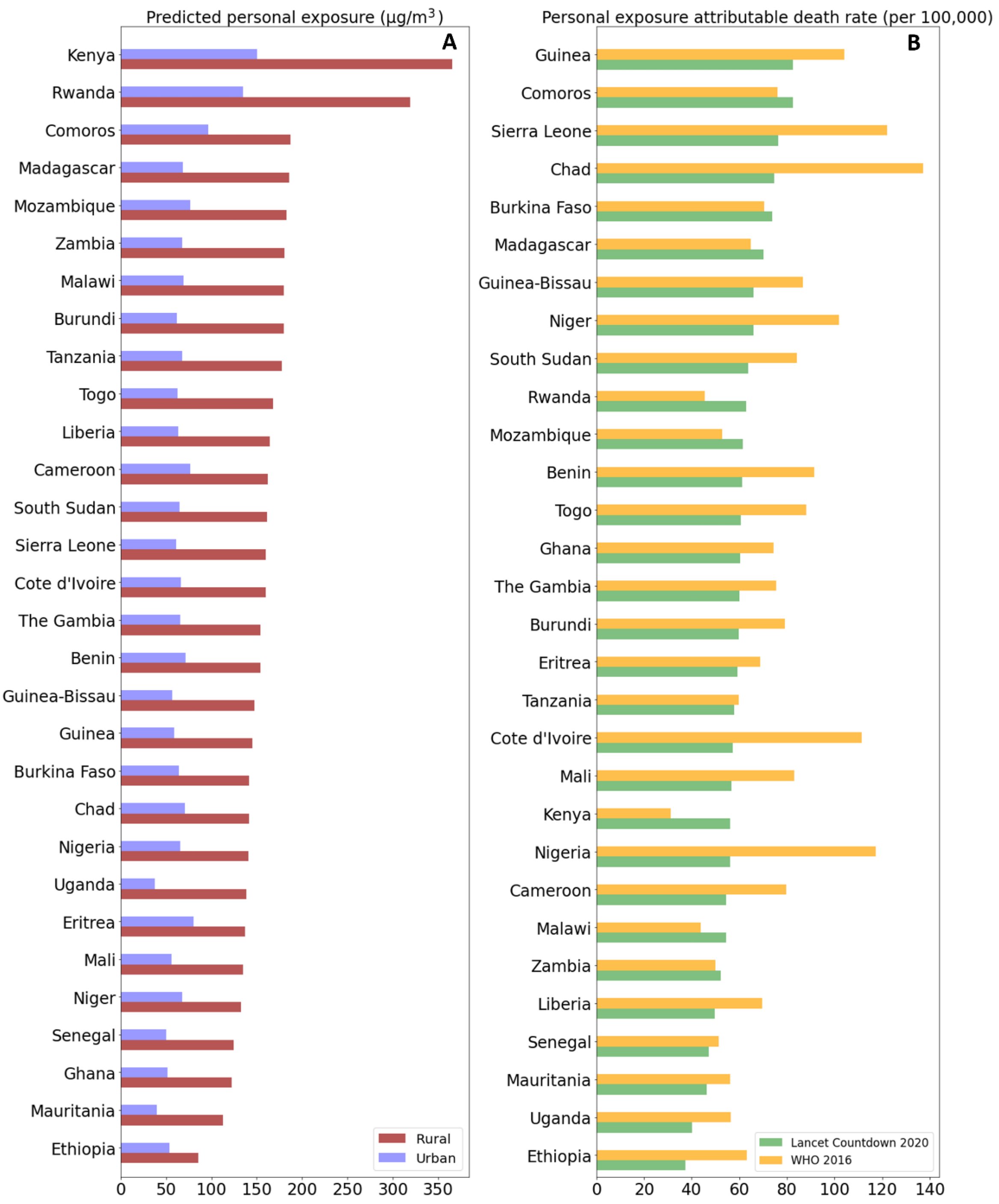

Figure 70: Exposure to household air pollution for countries in the WHO Africa Region. A. Annual average PM $_{2.5}$ personal exposure for solid fuels (biomass, charcoal, coal) at national level in rural and urban locations. B. Comparison between estimates of personal exposure attributable death rate (per 100,000) for individuals using solid fuels (biomass, charcoal, coal) at national level from the *Lancet* Countdown's indicator for 2020, and from WHO estimates for 2016.[217]

Link with COVID-19

The association between household air pollution and Covid-19 in the developing world has, unfortunately, not been well documented and the results obtained are somewhat unclear. One study on China indicates that while there was a decrease in ambient $PM_{2.5}$ the lockdown period from 25 January to 25 February 2020, the total population-weighted indoor and outdoor exposure to $PM_{2.5}$ during that period increased by 5.7 μg m^{-3} in compare with the preceding four weeks.[227] This increase was primarily attributable to increase in the indoor $PM_{2.5}$ concentration by 3.1 μg m^{-3} during the lockdown. The principal cause of indoor air pollution is the use of solid fuels such as coal and biomass for cooking and heating. This use is much more common in rural areas (67.5% as the share of solid fuels in the household energy mix) than in urban areas (4.7%).[227] Because of the high air pollution and the long time spent indoor in China, another study shows that the average daily $PM_{2.5}$ concentrations increased by 17.4 μg/m^3 in the kitchens and by 5.1 μg/m^3 in the living rooms during lockdown, both increases of which are attributable to more fuel consumption for cooking and heating during the lockdown than in normal periods in rural homes.[226]

3.3: Mortality from Ambient Air Pollution by Sector

Methods

This indicator quantifies contributions of individual source sectors to ambient $PM_{2.5}$ exposure and its health impacts. Contributions from coal have been highlighted across all sectors.

Estimates of sectoral source contributions to annual mean exposure to ambient $PM_{2.5}$ were calculated using the GAINS model,[228] which combines bottom-up emission calculations with atmospheric chemistry and dispersion coefficients.

Energy statistics are taken from the IEA World Energy Statistics for 2015, from the IEA World Energy Outlook 2020[229] for 2019 and from the World Energy Outlook 2021[216] for 2020. Data on energy consumption in individual sectors are imported into GAINS, matching the sectors of the World Energy Statistics and downscaling to the 180 GAINS global regions. They are then merged with GAINS information on application of emission control technologies in each region and their emission factors to calculate emissions of $PM_{2.5}$ and its precursor gases SO_2, NOx, NH_3, and non-methane VOC.

Ambient $PM_{2.5}$ concentrations are calculated from the region and sector specific emissions by applying atmospheric transfer coefficients, which are a linear approximation of full chemistry-transport models. Atmospheric transfer coefficients in GAINS are based on full year perturbation simulations with the EMEP Chemistry Transport Model[230] at 0.1°×0.1° resolution (for low-level sources) / 0.5°×0.5° resolution (for all other sources) using meteorology of 2015. In Europe, the resolution is slightly different but the principle is the same. Calculations for Europe are described in detail by Kiesewetter et al. (2015)[231], calculations for the rest of the world are described by Amann et al.[232] Calculated ambient $PM_{2.5}$ concentrations have been validated against in-situ observations from the WHO's Urban Ambient Air Pollution Database (2018 update)[233], and other sources where available (e.g. Chinese statistical yearbook) and show in general good agreement with monitoring data up to urban background level (local variation at roadside stations is not captured by the resolution of a few kilometres).

Deaths from total ambient $PM_{2.5}$ for regions other than Europe are calculated following the methodology of the Global Burden of Disease studies. Exposure-response relationships have been updated for this report to be consistent with the Global Burden of Disease 2019 study.[234] The MR-BRT curves were obtained from the public release site[235] and relative risks for six diseases IHD, COPD, stroke, lung cancer, ALRI, and type 2 diabetes calculated from them. The latter has been added this year. We used 1000 draws of the MRBRT curve for each disease and age group (where age specific) and scaled them to have RR=1 at the theoretical minimum-risk exposure level (taken from 1000 corresponding draws, average 4.15μgm^{-3}). Exposure levels below the TMREL level are assigned RR=1.

The update to the GBD-2019 exposure-response relationships resulted in a significant increase in attributable mortality beyond the numbers published in the previous editions of the *Lancet* Countdown, which were based on the integrated exposure response relationships (IERs) developed within the Global Burden of Disease 2013 study.[236]

Disease and age specific baseline mortality rates are taken from the GBD Results database[237] and have been updated to the 2019 data. The shares of different diseases were applied to age-specific total deaths taken from UN World Population Prospects (2017 update);[238] for 2019, the statistics were interpolated linearly between 2015 and 2020.

For Europe, this indicator follows the WHO Europe methodology and apply Exposure-response relationships for all-cause non-accidental mortality among the total population over 30 years of age. This year, concentration-response relationships have been updated to those reported in the systematic review for the 2021 WHO Air Quality Guidelines[239]. Other details are described in Kiesewetter et al. (2015).[231]

Attribution of estimated deaths from AAP to polluting sectors was done proportional to the contributions of individual sectors to population-weighted mean $PM_{2.5}$ in each country.

Data

1. Energy: IEA World Energy Balances for 2015,[240] World Energy Outlook 2020 (for the year 2019)[229], World Energy Outlook 2021[216] (for the year 2020)
2. Other activities: Agricultural livestock data are based on FAO statistics and projections[241] and fertilizer use is based on data from the International Fertilizer Association[242]
3. UN World Population Prospects, 2017 update[238]
4. Global Burden of Disease 2019 study,[234] MR-BRT curves obtained from the public release site[235]

Caveats

The indicator relies on model calculations which are inherently uncertain. The resolution of approximately seven to ten km is deemed appropriate for urban background levels of $PM_{2.5}$ but may underestimate exposure in case of strong local $PM_{2.5}$ increments. The meteorology year is fixed to 2015.

Uncertainty in the shape of integrated exposure-response relationships (IERs) make the quantification of health burden inherently uncertain.

Different dose-response relationships are used for Europe[239] and rest of the world.[243]

The non-linearity of the CRFs used for non-European countries complicates the translation between the mortality burden attributed to an individual source, which is calculated proportional to the source contribution to ambient $PM_{2.5}$, and the effect of mitigating this source. While a reduction of emissions would lead to a roughly proportional reduction of ambient $PM_{2.5}$, this would not necessarily result in a proportional reduction of the health burden. In highly polluted environments, the health benefits of a marginal reduction of emissions would be disproportionately smaller than the relative change in concentrations.

3.4: Sustainable and Healthy Road Transport

Methods

Fuel use data (by fuel type) from the IEA World Extended Energy Balances are divided by corresponding population statistics from the United Nations, Department of Economic and Social Affairs, Population Division (Figure 65).

The fuel flows from the IEA are combined in the following way:

Biofuels = Biodiesels +Biogasoline +Biogases + Other liquid biofuels

Fossil fuels = Natural gas liquids+Natural gas+Motor gasoline excl. biofuels+Liquefied petroleum gases (LPG)+Refinery gas+White spirit & SBP+Kerosene type jet fuel excl. biofuels+Gas/diesel oil excl. biofuels+Lubricants+Naphtha+Fuel oil+Other kerosene+Other oil products+Bitumen

Electricity is given by the existing IEA total.

Totals for a given year and country are then divided by the corresponding country population, and then summed to produce the final estimate. This avoids including the population of the countries that are not covered by the IEA.

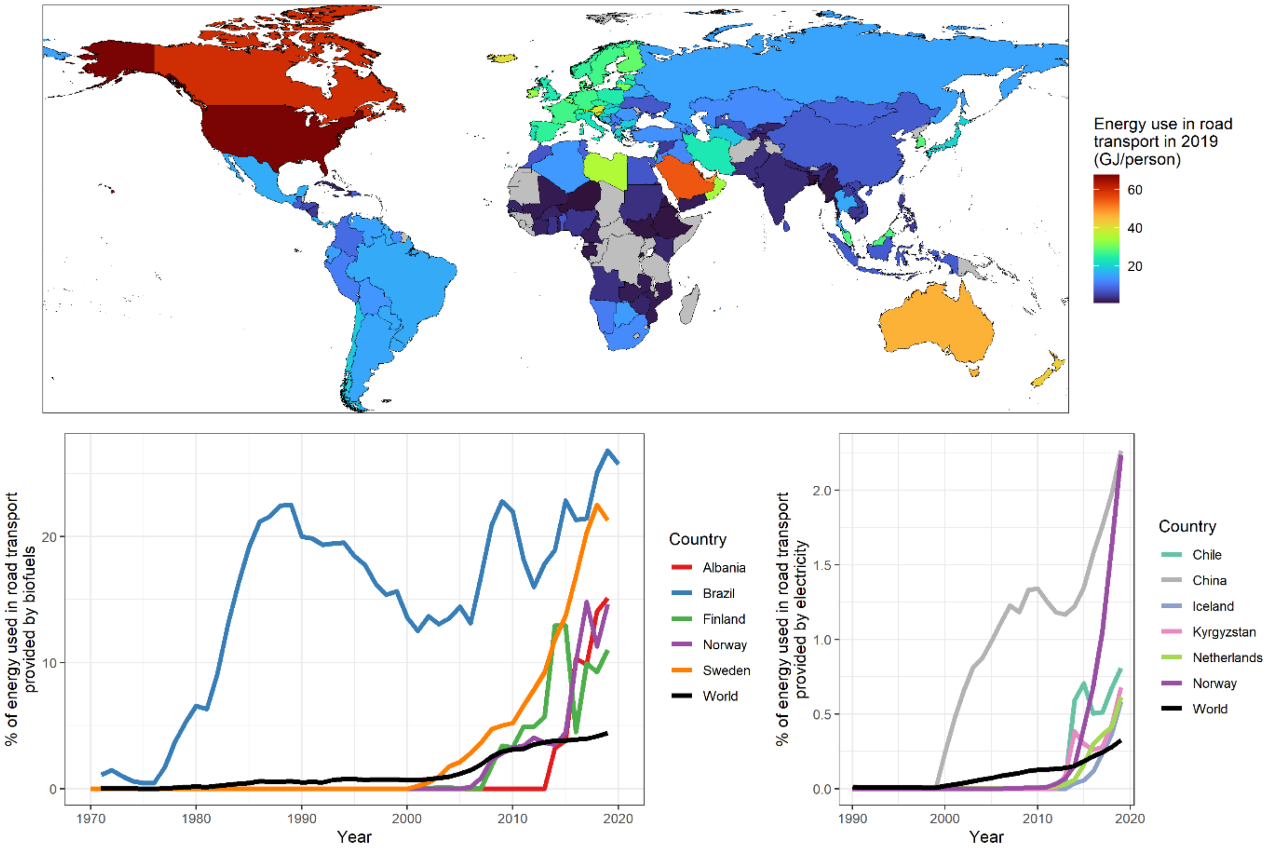

Figure 71: Upper panel: Per capita energy use in 2019, Lower panels: share of energy provided by biofuels (left) and electricity (right).

Data

1. Fuel use data is from the IEA, World Extended Energy Balances [205]
2. UN Population estimates, 2019 edition [244]

Caveats

This indicator captures change in total fuel use and type of fuel use for transport, but it does not capture shifts in modes of transport used. In particular, it does not capture walking and cycling for short trips, which can yield substantial health benefits through increased physical activity. [245]

Alongside the fossil fuel combustion pollutants, tyre wear accounts for an estimated 3-7% of airborne $PM_{2.5}$ particulates worldwide [246].

Future form of the indicator

An ideal fuel use indicator would capture the direct health impacts of the use of transport fuels, with country- and urban-level specificity within the global coverage. In turn, the co-benefits of transitioning to less-polluting

fuels would be quantified directly in terms of reduced exposures to air pollution and their corresponding health impact.

To capture sustainable uptake more fully a future indicator could collate information on the proportion of total distance travelled by different modes of transport based on comprehensive local survey data. Other data on sustainable travel infrastructure, for instance the presence of cycle schemes, would also be useful. The data described below in the additional analysis section provided from smartphone data serves to expand the picture provided by IEA data alone. Further development of data of this type may be possible in future reports.

Additional analysis

Figure 72 provides monthly, country level data from smartphones provided by Apple Inc. [247]. These data provide insight into changes in transport type usage. However, several caveats apply to these data. The data are collected based on navigation application queries, and thus may not capture trips which are made habitually for which the user does not require directional information. While smartphone saturation is high in most rich countries, the average smartphone user is not representative of inhabitants of a country as a whole, and it is likely that they represent data collected from higher socio-economic sectors of society. Changes over time to the

population that use smartphones of this type are also not controlled for in these data. Taken together, these caveats suggest caution is required in the interpretation of the information gathered from smartphones.

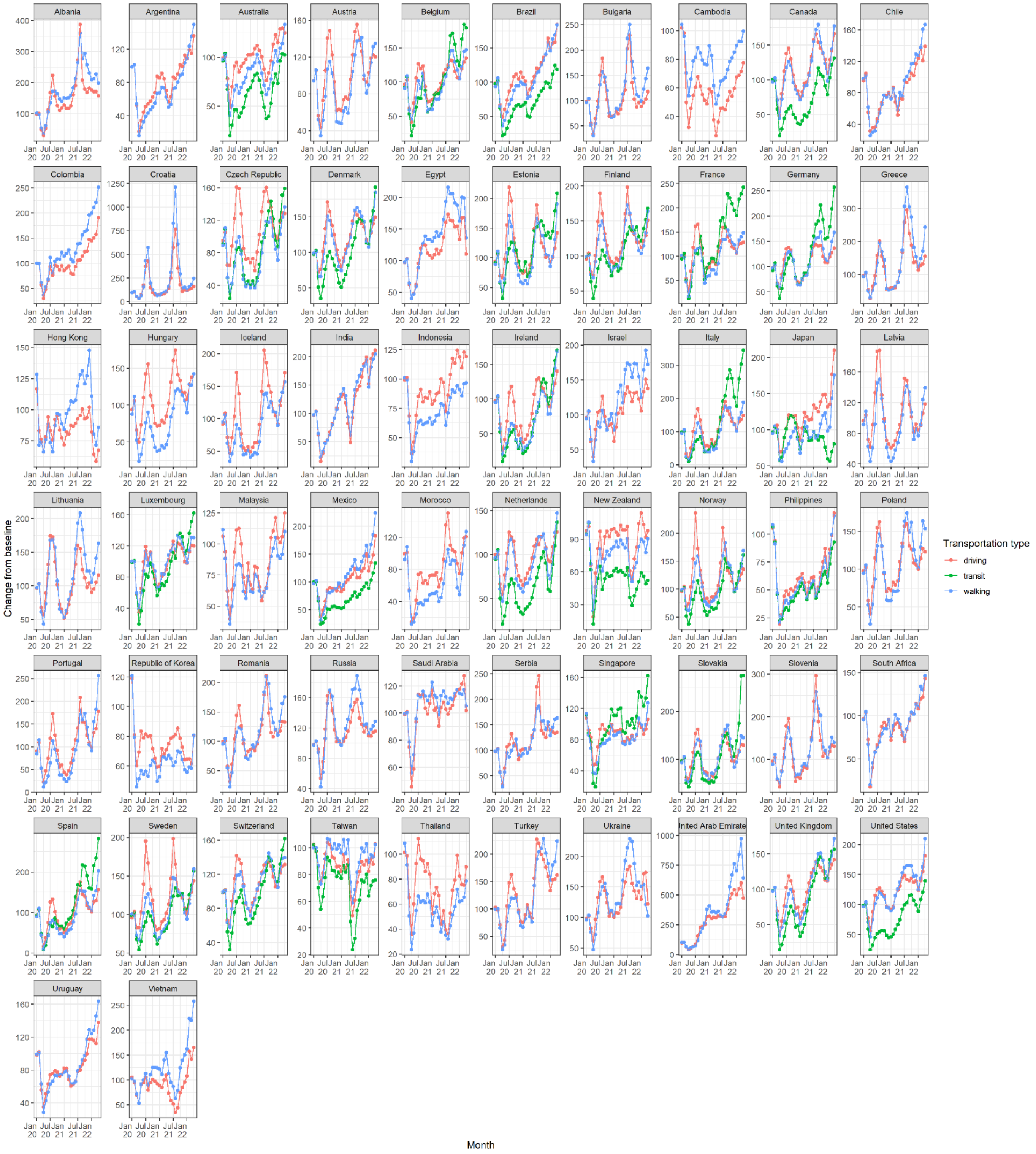

Figure 72 Monthly mobility data by transport type, updated to April 2022

3.5: Food, Agriculture, and Health

3.5.1: Emissions from Agricultural Production and Consumption

Methods

The 2022 update of this indicator radically increases the number of commodities considered. GHG emissions from agricultural production and consumption now incorporates new classes of fruits, vegetables, nuts, pulses and legumes and other crops. While these additional crops tend to have much lower carbon intensity than animal derived products, their inclusion provides a more complete picture of the agricultural commodities used in the global food system.

The methods by which the estimates of GHG for food products is devided into two sections, one covering livestock and the second covering crops.

Livestock products

Emissions intensities for the year 2000 are calculated in the following manner as in Dalin et al. [248] The following livestock species are included:

Ruminant	Non-Ruminant
Cattle, dairy<br><br>(FAO Item Code 960)	Chicken, broilers<br><br>(FAO Item Code 1053)
Cattle, non-dairy<br><br>(FAO Item Code 961)	Chicken, layers<br><br>(FAO Item Code 1052)
Buffaloes<br><br>(FAO Item Code 946)	Swine, market<br><br>(FAO Item Code 1049)
Goats<br><br>(FAO Item Code 1016)	Swine, breeding<br><br>(FAO Item Code 1079)
Sheep<br><br>(FAO Item Code 976)	

All livestock categories also include secondary products—such as cheese in the case of milk—where data were available. Cattle products comprise beef meat and milk and buffalo meat and milk. Sheep and goat products comprise meat and milk. Poultry products comprise meat and eggs of chickens, geese, ducks, and turkeys. Swine products include pork and secondary processed commodities, such as ham and bacon.

Emissions from enteric fermentation and manure management are obtained from Herrero et al.[249].

For manure left on pasture, rates from the GLOBIOM model were used[250] and a linear N_2O emission model applied.[251]

This information is presented in tonne carbon dioxide equivalent (CO_2e) per tropical livestock unit (tlu), which is converted to livestock head using the table below.[252]	Head per tlu
Bovine (Buffalo, Cattle (dairy), Cattle(non-dairy))	1.43
Small Ruminants (Goats, Sheep)	10
Poultry (Chicken)	100
Swine	5

The emissions per head are divided into world regions (as in the GLOBIOM model) and, for ruminants, livestock system (combination of climates from arid to humid, and practices from rangeland to feedlots, c.f. Herrero et al. 2013).[249] To convert these emissions to country values, an average is made across the region-system pairs within each country, weighted by the number of animals.

To obtain the emissions from grazing, the synthetic fertilizer applied to grassland from Chang et al.[253] is used as input to the N_2O emission model.[251] Animal products' emissions are also incorporated into the feed crop-related emissions proportionally to the feed ingredients consumed by animals—by species, region and systems— using feed data from Herrero et al. 2013.[249] These emissions from feed crops and grazed grasslands are then added to the direct livestock emissions (from enteric fermentation, manure management, and manure left on pasture) to provide overall emissions rates for each livestock species in the year 2000.

Finally, emissions intensity values for each livestock commodity (egg, meat, milk) and country are obtained by dividing CO_2e values by the output of milk/meat/egg per head from Herrero et al. 2013.[249]

Crop Products:

The emissions from fertilizer (synthetic and manure) application, rice cultivation and cultivation from organic soils for 172 crops for the year 2000 are obtained from Carlson et al. 2017,[254] who use IPCC methodology and a non-linear N_2O emission model. Crop types corresponding to "fodder" and "fibre" types are then excluded for this report, leaving 147 crops which are directly consumed by humans.

Crops used for livestock feed are excluded from the "crops" emissions, as they are included in the intensity of livestock production; the FAO reports this in the following way: "Cereal crops harvested for hay or harvested green for food, feed or silage or used for grazing are therefore excluded".[252]

Production values 2001–2019

Since the emission intensity of production is not constant over time, its values by commodity (for both livestock and crop products) were scaled using the FAO values as an index. The FAO produces GHG emissions intensity values by animal commodity and broad crop category (distinguishing rice, which, unlike other crops, emits large amounts of methane) for the countries covered by their analysis. However, these values are volatile at the country level, so regional values were used here. The percentage change from the year 2000 value was applied to the values derived from Herrero et al.,[249] Chang et al.[253] and Carlson et al.,[254] outlined above (methodology from Dalin et al. [248]). At the time of publication, the values for 2018 had not been published by the FAO, so the intensity scaling was assumed to be the same as in 2017. This will be updated in future years. Any missing values in scaling factor were assumed to be 1 (constant emission intensity). Any intensity values missing for a given country were given the regional average for that year and commodity, although practically this had little impact, because missing values only corresponded to countries which had very low or no production of the commodity in question.

Consumption emissions

The GHG emissions associated with agricultural commodity consumption uses FAO production and trade data to estimate the total GHG emissions footprint associated with each of the commodities considered in a given

country. This method is used by Dalin et al.[255] for tracing water consumption in global food networks but is adapted here to calculated GHG footprint. The basic equation the indicator follows is:

Consumption = production + imports - exports

FAO production and trade data are used in the following manner. For a given commodity the national production values in tonnes are converted into CO_2e values using the GHG emissions intensity values supplied by indicator 3.5.1 GHG production estimates (via Carlson et al. 2017[254]) associated with producing that tonnage of the commodity. Next, secondary commodities are converted in primary equivalent values by multiplying the trade tonnage by the value derived from Dalin et al. 2017.[255] For example, the primary equivalences for wheat products are as shown in Table 42:

Bran, wheat	1.01
Bread	0.88
Bulgur	1.05
Cereals, breakfast	1.18
Flour, wheat	1.01
Macaroni	1.01
Pastry	0.88
Wafers	0.88
Wheat	1.00

Table 42: Primary equivalences for wheat products

These values are then converted into GHG emissions equivalent, based on the GHG emissions intensity. For a given year, the trade balances are corrected to take into account that a given commodity may have been produced in one country, processed in another and finally imported into a third, using an algorithm developed by Kastner et al 2011.[256]

Data

1. National annual production of animal products items (tonnes) – FAOSTAT (2021 update)[252]
2. National annual trade (country-country) of animal products items (tonnes) – FAOSTAT (2021 update)[252]
3. Correspondence of items across item lists with different grouping – FAOSTAT[252]
4. GHG emissions intensity per country of animal products – provided by LC 3.7 GHG production estimates including grassland and feed crop emissions (via Herrero et al. 2013 and Dalin et al. 2019)[248,249] Definitions: Animal types: bovine cattle (beef and buffalo), sheep and goat ruminants, pigs, poultry (chicken, ducks, geese and turkeys)
5. National annual production of crops (tonnes) – FAOSTAT (2021 update)[252]
6. National annual trade (country-country) of crop products (tonnes) – FAOSTAT (2021 update)[252]
7. GHG emissions intensity of crop products for each country– provided by Carlson et al. (2017)[254]

Additional analysis

A substantial amount of CO_2e is associated with food that it is not consumed, whether that be during the food production process, transportation loses, or being wasted at the plate. The volumes of food considered here include food that is wasted or lost in transport, but not the additional emissions associated with the decomposition of food waste. The IPPC estimates that between 8–10% of total anthropogenic GHG emissions are associated with food loss and waste.[257] However, wastage is not equally distributed by country, with one analysis finding that high income nations waste six times by weight the amount that low-income ones.[258]

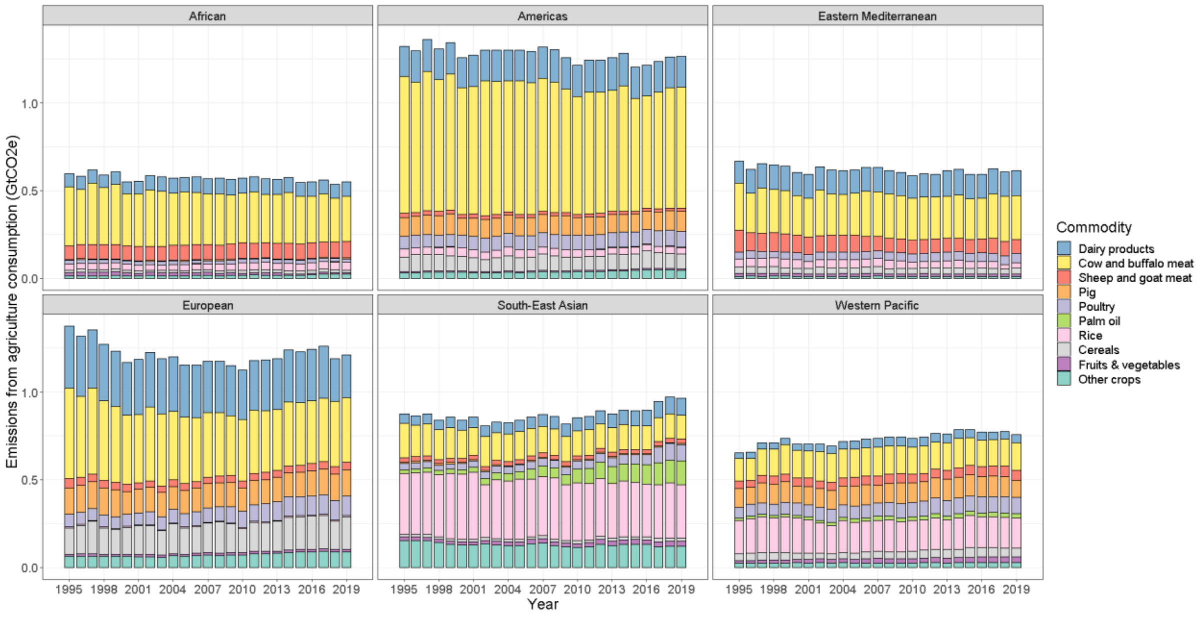

Figure 73 Emissions of greenhouse gases on farms associated with food consumption (production and net imports) by WHO region (GtCO$_2$e).

Caveats

In the context of this indicator, *consumption* refers to the net balance of food products entering a country within a given year, i.e., national production and *net* imports together, which could also be referred to as “national supply”. Here net imports refers to imports minus exports. It does not refer to the total GHG emissions attributable to food consumed by individuals. Indeed, at present, this indicator only considers the emissions associated with food production described above and does not take into account emissions associated with food transport and processing, storage and decomposition, and use change and deforestation.[259]

This indicator does not account for emissions associated with land conversion to agriculture (such as deforestation) but does consider emissions form cultivation of organic soils (such as peatland).

For livestock, data on stock numbers has been extracted from FAO database, however, some data is missing for some years, most notably Somalia (missing data 2000–2011) for non-dairy cattle. Data on grazing emissions from small islands is also missing, and therefore imputed using regional average values as described above.

The emission factors differ from FAO numbers:

- For livestock, this is due to calculation of emissions of enteric fermentation, manure management and manure left on pasture at GLOBIOM region (n=29) and livestock system (n=8) level whereas the FAO use subcontinental (n=9) and climatic level (n=3).[252]
- For crops, this is due to the FAO assuming slightly higher synthetic N application, greater manure N inputs, and a linear emissions factor of 1%, in contrast to a mean of 0.77% used by the non-linear model of Carlson et al. (2017).[254]

Agricultural consumption emissions estimates are derived directly from FAO trade values (re-organised as producer-consumer trade only with the algorithm), as described above. Therefore, these values differ from the production estimates, which are based on extrapolating year 2000 figures. On average across all years, the estimate of total emissions due to consumption are 2.25% above production values, and do not differ by more than 10% in any given year. The sole exception to this is the estimates of the differences between production and consumption by WHO region shown in the figure in the main text. For this figure the production values are derived directly from FAO values.

Future form of the Indicator

As highlighted above, the indicator does not take into account emissions associated with food transport and processing, storage and waste, land use change and deforestation. According to analysis from the IPCC,[257] when analysed on a 100-year global warming potential basis, yearly agricultural emissions between 2007 and 2016 were on average 6.2 ± 1.4 $GtCO_2e$ yr^{-1}, rising to 11.1 ± 2.9 $GtCO_2e$ yr^{-1} when taking into account land-use and land-use change, such as deforestation and the degradation of peatlands. While the indicator here does incorporate the impact of peatland drainage, emissions associated with deforestation are currently not included. Including an estimate of deforestation in this indicator will be explored for future publications.

3.5.2 Diet and health co-benefits

Methods

Baseline consumption data

Baseline food consumption was estimated by adopting estimates of food availability from the FAO's food balance sheets, and adjusting those for the amount of food wasted at the point of consumption.[260,261] This proxy for food consumption was disaggregated by age and sex by adopting the same age and sex-specific trends as observed in dietary surveys.[262]

An alternative would have been to rely on a set of consumption estimates that has been based on a variety of data sources, including dietary surveys, household budget and expenditure surveys, and food availability data.[263,264] However, neither the exact combination of these data sources, nor the estimation model used to derive the data have been made publicly available. For some individual countries, using dietary surveys would also have been an alternative. However, underreporting is a persistent problem in dietary survey,[265,266] and regional differences in survey methods would have meant that the results would not be comparable between countries. In contrast to dietary surveys, waste-adjusted food-availability estimates indicate levels of energy intake per region that reflect differences in the prevalence of overweight and obesity across regions.[267]

Food balance sheets report on the amount of food that is available for human consumption.[260] They reflect the quantities reaching the consumer, but do not include waste from both edible and inedible parts of the food commodity occurring in the household. As such, the amount of food actually consumed may be lower than the quantity shown in the food balance sheet depending on the degree of losses of edible food in the household, e.g., during storage, in preparation and cooking, as plate-waste, or quantities fed to domestic animals and pets, or thrown away.

The waste-accounting methodology developed by the FAO was followed to account for the amount of food wasted at the household level that was not accounted for in food availability estimates.[261] Table 43 provides an overview of the parameters used in the calculation.

For each commodity and region, food consumption was estimated by multiplying food availability data with conversion factors (*cf*) that represent the amount of edible food (e.g., after peeling) and with the percentage of food wasted during consumption (*1-wp(cns)*). The difference in wastage for roots and tubers, fruits and vegetables, and fish and seafood, also accounted for differences between the proportion that is utilised fresh (pct_{frsh}) and the proportion that utilised in processed form (pct_{prcd}). The equation used for each food commodity and region was:

$$Consumption = Availability \cdot \frac{pct_{frsh}}{100} \cdot cf_{frsh} \cdot \left(1 - \frac{wp(cns_{frsh})}{100}\right)$$

$$+ Availability \cdot \frac{pct_{prcd}}{100} \cdot cf_{prcd} \cdot \left(1 - \frac{wp(cns_{prcd})}{100}\right)$$

Food group	Item	Region						
		Europe	USA, Canada, Oceania	Indus-trialized Asia	Sub-Saharan Africa	North Africa, West and Central Asia	South and Southeast Asia	Latin America
cereals	wp(cns)	25	27	20	1	12	3	10
roots and tuber	pct_{prcd}	73	73	15	50	19	10	80
	wp(cns)	17	30	10	2	6	3	4
	$wp(cns_{prcd})$	12	12	12	1	3	5	2
oilseeds and pulses	cns	4	4	4	1	2	1	2
fruits and vegetables	pct_{prcd}	60	60	4	1	50	5	50
	wp(cns)	19	28	15	5	12	7	10
	$wp(cns_{prcd})$	15	10	8	1	1	1	1
milk and dairy	wp(cns)	7	15	5	0.1	2	1	4
eggs	wp(cns)	8	15	5	1	12	2	4
meat	wp(cns)	11	11	8	2	8	4	6
fish and seafood	pct_{prcd}	*40% for low-income countries, and 96% for all others.*						
	wp(cns)	11	33	8	2	4	2	4
	$wp(cns_{prcd})$	10	10	7	1	2	1	2

Conversion factors: maize, millet, sorghum: 0.69; wheat, rye, other grains: 0.78; rice: 1; roots: 0.74 (0.9 for industrial processing); nuts and seeds: 0.79; oils: 1; vegetables: 0.8 (0.75 for industrial processing); fruits: 0.8 (0.75 for industrial processing); beef: 0.715; lamb: 0.71; pork: 0.68; poultry: 0.71; other meat: 0.7; milk and dairy: 1; fish and seafood: 0.5; other crops: 0.78

Table 43. Percentage of food wasted during consumption (cns), and percentage of processed utilisation (pctprcd). The percentage of fresh utilisation is calculated as 1-pctprcd. Conversion factors to edible portions of foods are provided below the table.

Comparative risk assessment

The mortality and disease burden attributable to dietary and weight-related risk factors was estimated by calculating population impact fractions (PIFs) which represent the proportions of disease cases that would be avoided when the risk exposure was changed from a baseline situation to a counterfactual situation. For calculating PIFs, using the general formula:[268-270]

$$PIF = \frac{\int RR(x)P(x)dx - \int RR(x)P'(x)dx}{\int RR(x)P(x)dx}$$

where $RR(x)$ is the relative risk of disease for risk factor level x, $P(x)$ is the number of people in the population with risk factor level x in the baseline scenario, and $P'(x)$ is the number of people in the population with risk factor level x in the counterfactual scenario. It was assumed that changes in relative risks follow a dose-response relationship,[269] and that PIFs combine multiplicatively, i.e. $PIF = 1 - \prod_i (1 - PIF_i)$ where the i's denote independent risk factors.[269,271]

The number of avoided deaths due to the change in risk exposure of risk i, $\Delta deaths_i$, was calculated by multiplying the associated PIF by disease-specific death rates, DR, and by the number of people alive within a population, P:

$$\Delta deaths_i(r,s,a,d) = PIF_i(r,s,a,d) \cdot DR(r,s,a,d) \cdot P(r,s,a)$$

where PIFs are differentiated by region *r*, sex *s*, age group *a*, and disease/cause of death *d*; the death rates are differentiated by region, sex, age group, and disease; the population groups are differentiated by region, sex, and age group; and the change in the number of deaths is differentiated by region, sex, age group, and disease.

Publicly available data sources were used to parameterize the comparative risk analysis. Mortality and population data were adopted from the Global Burden of Disease project.[272] Baseline data on the weight distribution in each country were adopted from a pooled analysis of population-based measurements undertaken by the NCD Risk Factor Collaboration.[267]

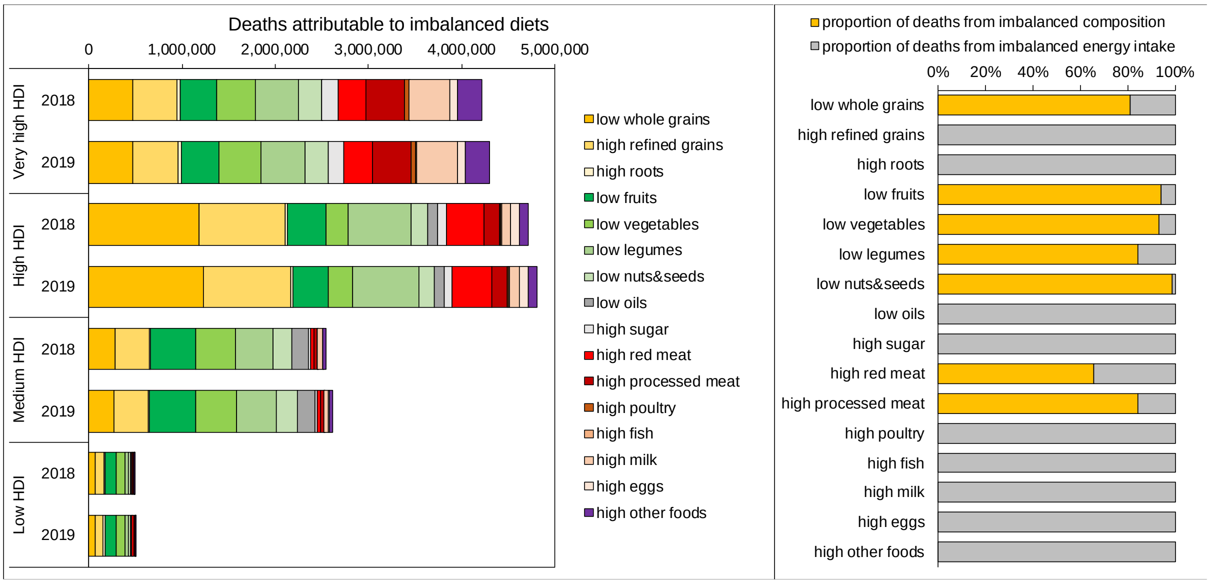

Figure 74: Deaths attributable to diet-related risks in 2018 and 2019 by risk factor and development region (left) and composition of attributable deaths into those from imbalanced composition and imbalanced energy intake (right).

The relative risk estimates that relate the risk factors to the disease endpoints were adopted from meta-analyses of prospective cohort studies for dietary and weight-related risks.[273] In line with the meta-analyses, non-linear dose-response relationships were included for fruits, vegetables, and nuts and seeds, and assumed linear dose-response relationships for the remaining risk factors. As the analysis was primarily focused on mortality from chronic diseases, the focus was on adults aged 20 year or older, and the relative-risk estimates were adjusted for attenuation with age based on a pooled analysis of cohort studies focussed on metabolic risk factors,[274] in line with other assessments.[270,275]

Table 44 Relative risk parameters (mean and low and high values of 95% confidence intervals) for dietary risks and weight-related risks. **Table** 44 provides an overview of the relative-risk parameters used. For the counterfactual scenario, minimal risk exposure levels (TMRELs) was defined as follows: 300 g/d for fruits, 500 g/d for vegetables, 100 g/d for legumes, 20 g/d for nuts and seeds, 125 g/d for whole grains, 0 g/d for red meat, 0 g/d for processed meat, and no underweight, overweight, or obesity. The TMRELs are in line with those defined by the Nutrition and Chronic Diseases Expert Group (NutriCoDE),[275] with the exception that a higher value for vegetables was used, and zero was used as minimal risk exposure for red meat, in each case based on a more comprehensive meta-analysis.[276,277]

The selection of risk-disease associations used in the health analysis was supported by available criteria used to judge the certainty of evidence, such as the Bradford-Hill criteria used by the Nutrition and Chronic Diseases Expert Group (NutriCoDE),[275] the World-Cancer-Research-Fund criteria used by the Global Burden of Disease project,[278] as well as NutriGrade (Table 44).[279] The certainty of evidence supporting the associations of dietary risks and disease outcomes as used here were graded as moderate or high with NutriGrade,[277,280,281] and/or

assessed as probable or convincing by the Nutrition and Chronic Diseases Expert Group,[275] and by the World Cancer Research.[282] The certainty of evidence grading in each case relates to the general relationship between a risk factor and a health outcome, and not to a specific relative-risk value.

Food group	Endpoint	Unit	RR mean	RR low	RR high	Reference
Processed meat	CHD	50 g/d	1.27	1.09	1.49	Bechthold et al (2019)
	Stroke	50 g/d	1.17	1.02	1.34	Bechthold et al (2019)
	Colorectal cancer	50 g/d	1.17	1.10	1.23	Schwingshackl et al (2018)
	Type 2 diabetes	50 g/d	1.37	1.22	1.55	Schwingshackl et al (2017)
Red meat	CHD	100 g/d	1.15	1.08	1.23	Bechthold et al (2019)
	Stroke	100 g/d	1.12	1.06	1.17	Bechthold et al (2019)
	Colorectal cancer	100 g/d	1.12	1.06	1.19	Schwingshackl et al (2018)
	Type 2 diabetes	100 g/d	1.17	1.08	1.26	Schwingshackl et al (2017)
Fruits	CHD	100 g/d	0.95	0.92	0.99	Aune et al (2017)
	Stroke	100 g/d	0.77	0.70	0.84	Aune et al (2017)
	Cancer	100 g/d	0.94	0.91	0.97	Aune et al (2017)
Vegetables	CHD	100 g/d	0.84	0.80	0.88	Aune et al (2017)
	Cancer	100 g/d	0.93	0.91	0.95	Aune et al (2017)
Legumes	CHD	57 g/d	0.86	0.78	0.94	Afshin et al (2014)
Nuts	CHD	28 g/d	0.71	0.63	0.80	Aune et al (2016)
Whole grains	CHD	30 g/d	0.87	0.85	0.90	Aune et al (2016b)
	Cancer	30 g/d	0.95	0.93	0.97	Aune et al (2016b)
	Type 2 diabetes	30 g/d	0.65	0.61	0.70	Aune et al (2016b)
Underweight	CHD	15<BMI<18.5	1.17	1.09	1.24	Global BMI Collab (2016)
	Stroke	15<BMI<18.5	1.37	1.23	1.53	Global BMI Collab (2016)
	Cancer	15<BMI<18.5	1.10	1.05	1.16	Global BMI Collab (2016)
	Respiratory disease	15<BMI<18.5	2.73	2.31	3.23	Global BMI Collab (2016)
Overweight	CHD	25<BMI<30	1.34	1.32	1.35	Global BMI Collab (2016)
	Stroke	25<BMI<30	1.11	1.09	1.14	Global BMI Collab (2016)
	Cancer	25<BMI<30	1.10	1.09	1.12	Global BMI Collab (2016)
	Respiratory disease	25<BMI<30	0.90	0.87	0.94	Global BMI Collab (2016)
	Type 2 diabetes	25<BMI<30	1.88	1.56	2.11	Prosp Studies Collab (2009)
Obesity (grade 1)	CHD	30<BMI<35	2.02	1.91	2.13	Global BMI Collab (2016)
	Stroke	30<BMI<35	1.46	1.39	1.54	Global BMI Collab (2016)
	Cancer	30<BMI<35	1.31	1.28	1.34	Global BMI Collab (2016)
	Respiratory disease	30<BMI<35	1.16	1.08	1.24	Global BMI Collab (2016)
	Type 2 diabetes	30<BMI<35	3.53	2.43	4.45	Prosp Studies Collab (2009)
Obesity (grade 2)	CHD	30<BMI<35	2.81	2.63	3.01	Global BMI Collab (2016)
	Stroke	30<BMI<35	2.11	1.93	2.30	Global BMI Collab (2016)
	Cancer	30<BMI<35	1.57	1.50	1.63	Global BMI Collab (2016)
	Respiratory disease	30<BMI<35	1.79	1.60	1.99	Global BMI Collab (2016)
	Type 2 diabetes	30<BMI<35	6.64	3.80	9.39	Prosp Studies Collab (2009)
Obesity (grade 3)	CHD	30<BMI<35	3.81	3.47	4.17	Global BMI Collab (2016)
	Stroke	30<BMI<35	2.33	2.05	2.65	Global BMI Collab (2016)
	Cancer	30<BMI<35	1.96	1.83	2.09	Global BMI Collab (2016)
	Respiratory disease	30<BMI<35	2.85	2.43	3.34	Global BMI Collab (2016)
	Type 2 diabetes	30<BMI<35	12.49	5.92	19.82	Prosp Studies Collab (2009)

Table 44 Relative risk parameters (mean and low and high values of 95% confidence intervals) for dietary risks and weight-related risks.

Not all available risk-disease associations that were graded as having a moderate certainty of evidence and showed statistically significant results in the meta-analyses that included NutriGrade assessments were included in the analysis.[277,280,281] That was because for some associations, such as for milk and fish, more detailed meta-analyses (with more sensitivity analyses) were available that indicated potential confounding with other major dietary risks or health status at baseline.[283-285] Such sensitivity analyses were not presented in the meta-analyses that included NutriGrade assessments, but they are important for health assessments that evaluate changes in multiple risk factors (Table 45).

Food group	Endpoint	Association	Certainty of evidence
Fruits	CHD	reduction	NutriCoDE: probable or convincing;<br>NutriGrade: moderate quality of meta-evidence
	Stroke	reduction	NutriCoDE: probable or convincing<br>NutriGrade: moderate quality of meta-evidence
	Cancer	reduction	WCRF: strong evidence (probable) for some cancers<br>NutriGrade: moderate quality of meta-evidence for colorectal cancer
Vegetables	CHD	reduction	NutriCoDE: probable or convincing<br>NutriGrade: moderate quality of meta-evidence
	Cancer	reduction	WCRF: strong evidence (probable) for non-starchy vegetables and some cancers<br>NutriGrade: moderate quality of meta-evidence for colorectal cancer
Legumes	CHD	reduction	NutriCoDE: probable or convincing<br>NutriGrade: moderate quality of meta-evidence
Nuts and seeds	CHD	reduction	NutriCoDE: probable or convincing<br>NutriGrade: moderate quality of meta-evidence
Whole grains	CHD	reduction	NutriCoDE: probable or convincing<br>NutriGrade: moderate quality of meta-evidence
	Cancer	reduction	WCRF: strong evidence (probable) for colorectal cancer<br>NutriGrade: moderate quality of meta-evidence for colorectal cancer
	Type-2 diabetes	reduction	NutriCoDE: probable or convincing<br>NutriGrade: high quality of meta-evidence
Red meat	CHD	increase	NutriGrade: moderate quality of meta-evidence
	Stroke	increase	NutriGrade: moderate quality of meta-evidence
	Cancer	increase	WCRF: strong evidence (probable) for colorectal cancer<br>NutriGrade: moderate quality of meta-evidence for colorectal cancer
	Type-2 diabetes	increase	NutriCoDE: probable or convincing<br>NutriGrade: high quality of meta-evidence
Processed meat	CHD	increase	NutriCoDE: probable or convincing<br>NutriGrade: moderate quality of meta-evidence
	Stroke	increase	NutriGrade: moderate quality of meta-evidence
	Cancer	increase	WCRF: strong evidence (convincing) for colorectal cancer<br>NutriGrade: moderate quality of meta-evidence for colorectal cancer
	Type-2 diabetes	increase	NutriGrade: high quality of meta-evidence

NutriCoDE: Nutrition and Chronic Diseases Expert Group

NutriGrade: Grading of Recommendations Assessment, Dvelopment, and Evaluation (GRADE) tailored to nutrition research

WCRF: World Cancer Research Fund

Table 45 Overview of existing ratings on the certainty of evidence for a statistically significant association between a risk factor and a disease endpoint. The ratings include those of the Nutrition and Chronic Diseases Expert Group (NutriCoDE),[275] the World Cancer Research Fund,[282] and NutriGrade.[277,280,281] The ratings relate to the risk-disease associations in general, and not to the specific relative-risk factor used for those associations in this analysis.

Weight-related risks are connected to imbalanced energy intake. To highlight this connection, the weight-related disease burden was attributed to consuming too much or too little of specific foods. For that purpose, the current energy intake by food group in each country was first compared to a dietary pattern that minimises both diet and

weight-related risks, and then attributed the proportion of energy intake of under and over-consumed foods to the proportion of deaths attributable to underweight on the one hand and to overweight and obesity on the other. The minimal-risk patterns were based on recommendations for optimal energy intake given the sex, age, and height structure of each country,[267,286] the TMREL values used in the dietary risk assessment,[273,275,277,287-290] and food-based recommendations for healthy and sustainable diets for the remaining food groups.[291] The recommendations were implemented as minimum and maximum values, which preserved a country's intake if it was within recommendations (Table 46).

Food group	Recommended intake		Source
	Min	Max	
Fruits	300	>300	Aune et al (2017)
Vegetables	500	>500	Aune et al (2017)
Legumes	100	>100	Micha et al (2017), Afshin et al (2017)
Nuts and seeds	20	>20	Micha et al (2017), Aune et al (2016a)
Whole grains	125	225	Micha et al (2017), Aune et al (2016b)
Red meat	0	0	GBD 2019 (2020), Bechthold et al (2017)
Processed meat	0	0	GBD 2019 (2020), Bechthold et al (2017)
Oils	40	80	Willett et al (2019)
Sugar	0	31	Willett et al (2019)
Roots	0	100	Willett et al (2019)
Milk	0	250	Willett et al (2019)
Eggs	0	13	Willett et al (2019)
Poultry	0	29	Willett et al (2019)
Fish	0	28	Willett et al (2019)

Table 46 Food-based recommendations used to construct minimal risk dietary patterns. The recommendations include minimal risk exposure levels for dietary risks (upper rows) and food-based recommendations for a healthy and sustainable diets (lower rows).

For the different diet scenarios, uncertainty intervals were calculated associated with changes in mortality based on standard methods of error propagation and the confidence intervals of the relative risk parameters. For the error propagation, the error distribution was approximated of the relative risks by a normal distribution and used that side of deviations from the mean which was largest. This method leads to conservative and potentially larger uncertainty intervals as probabilistic methods, such as Monte Carlo sampling, but it has significant computational advantages, and is justified for the magnitude of errors dealt with here (<50%) (see e.g., IPCC Uncertainty Guidelines).

Data

Table 47 provides an overview of the data sources used for this indicator.

Type	Coverage	Source
Exposure data:		
Food consumption data	Country-level	Food availability data adjusted for food waste at the household level and for age and sex-specific trends.[260-262] Estimates of energy intake were in line with trends in body weight across countries.[267]
Weight estimates	Country-level	Baseline data from pooled analysis of measurement studies differentiated by sex and age with global coverage.[267]
Health analysis:		
Relative risk estimates	General	Adopted from meta-analysis of prospective cohort studies.[273,276,277,280,281,287,292] The certainty of evidence for the risk-disease associations were rated as moderate to high by NutriGrade.[277,280,281]
Mortality and population data	Country-level	Adopted from the Global Burden of Disease project by country, sex, and age group.[272]

Table 47 Overview of data sources

Caveats

In the comparative risk assessment, relative risk factors were used that are subject to the caveats common in nutritional epidemiology, including small effect sizes and potential measurement error of dietary exposure, such as over and underreporting and infrequent assessment.[293] For the calculations, it was assumed that the risk-disease relationships describe causal associations, an assumption supported by the existence of statistically significant dose-response relationships in meta-analyses, the existence of plausible biological pathways, and supporting evidence from experiments, e.g., on intermediate risk factors.[273,275,277,280,281,288,289,292,294-296] However, residual confounding with unaccounted risk factors cannot be ruled out in epidemiological studies. Additional aspects rarely considered in meta-analyses are the importance of substitution between food groups that are associated with risks, and the time lag between dietary exposure and disease.

To address potential confounding, risk-disease associations were omitted that became non-significant in fully adjusted models, in particular those related milk intake,[283,284] and to fish intake.[285,297-299] The quality of evidence in meta-analyses that covered the same risk-disease associations as used here was graded with NutriGrade as moderate or high for all risk-disease pairs included in the analysis (Table 44).[277,280,281] In addition, the Nutrition and Chronic Diseases Expert Group and the World Cancer Research Fund graded the evidence for a causal association of ten of the 12 risk-disease associations included in the analysis as probable or convincing,[275,282] The relative health ranking of leading risk factors found in the analysis was similar to existing rankings that relied on different relative-risk parameters and exposure data.[281]

As exposure data, a proxy of food consumption was used that was derived from estimates of food availability that were adjusted for the amount of food wasted at the point of consumption.[260,261] An alternative would have

been to rely on a set of consumption estimates that has been based on a variety of data sources, including dietary surveys, household budget and expenditure surveys, and food availability data.[263,264] However, neither the exact combination of these data sources, nor the estimation model used to derive the data have been made publicly available. For some individual countries, using dietary surveys would also have been an alternative. However, underreporting is a persistent problem in dietary survey,[265,266] and regional differences in survey methods would have meant that the results would not be comparable between countries. In contrast to dietary surveys, waste-adjusted food-availability estimates indicate levels of energy intake per region that reflect differences in the prevalence of overweight and obesity across regions.[267]

3.6: Healthcare Sector Emissions

Methods

This indicator is in the form of healthcare-associated GHG emissions per capita per year, including direct emissions from healthcare facilities as well as emissions from the consumption of goods and services supplied by other sectors. Results are calculated by assigning aggregate national health expenditures from WHO to final demand for 'Health and Social Work' sectors in the EE-MRIO model. Environmental satellite accounts including GHG emissions accompany each EE-MRIO model. Consumption-based GHG emissions are then calculated using the standard Leontief inverse technique [300]

Results for years after the MRIO model year are achieved through deflation of healthcare expenditure data. Both WIOD and EXIOBASE3 MRIO models were run for this analysis and results compared; WIOD results are shown following the prior year report methods, while EXIOBASE3 results showed severe year-to-year volatility in some results that could not be readily explained.[301,302] WIOD tables are in US dollars, while EXIOBASE3 tables are in euros. For expenditure years after the model baseline, WHO expenditure data in nominal US dollars expenditures are converted to nominal national currencies using market exchange rates, deflated in national currencies to baseline year using consumer price indices from the World Bank, and converted to baseline model year currency (dollars or euros) using market exchange rates.[303,304]

The *Lancet* Countdown reported healthcare sector GHG emissions for the first time in 2019.[171] In that report, reflecting 2016 conditions, global healthcare emissions were found to contribute approximately 4.6% of global emissions, with large disparities in per capita emissions of more than 40x across the countries studied. Independent research by Pichler et al. on CO_2 emissions (excluding other GHGs) associated with health care in OECD countries (excluding Chile) as well as India and China found a contribution of 4.4% in 2014, while an NGO effort covering all GHG emissions estimated 4.4% in 2014.[305,306] The Pichler et al. work considered temporal trends and introduced adjustments into the emissions satellite accounts of the EE-MRIO model EORA to reflect shifts in major GHG emissions sources that occurred between the baseline model year and when each healthcare expenditure occurred. Based on this suggestion, the *Lancet* Countdown modelling approach was updated in 2020 in the same way, using the PRIMAP database of national GHG emissions to adjust emissions by sector relative to the baseline year.[307] Subsequent analysis by Lenzen et al.[308] using the EORA EE-MRIO model including all GHGs found that healthcare contributes of 4.4% of global GHG emissions (close to the *Lancet* Countdown's estimate of 4.6% for that year), as well as 2.8% of particulate matter, 3.5% of NO_x, and 3.6% of SO_2 emissions in 2015.

Data

1. Environmentally extended multi-region input-output tables: WIOD 2013 release with environmental accounts, latest model year 2011, latest emissions account year 2009, air emissions include CO_2, CH_4, N_2O, NO_x, SO_x, CO, NMVOC, and NH_3;
2. Per capita health expenditure data is from the World Health Organization's Global Health Expenditure Database; the latest reporting year is 2019. [309] Population data is also from the WHO[304]
3. Market exchange rates are from UN Statistics Division[310]

4. Consumer price indices are from the World Bank[303]
5. Healthy life expectancy at birth (both sexes) is from the World Health Organization's Global Health Observatory for reporting year 201 [309]

Caveats

As only total health expenditure data are available from WHO, all expenditures are assigned to Final Demand, with no separation for investment.

MRIO models are built from aggregated top-down statistical data. Results do not reflect individual health care systems' power purchase agreements for renewable energy or any offsetting activities. Results do not include direct emissions of waste anaesthetic gases from clinical operations nor emissions from metered dose inhalers, as these are not currently reported consistently in national emissions inventories.

Future form of the indicator

This indicator could be extended to include health damages from GHG and air pollutant emissions from the healthcare sector.

Section 4: Economics and Finance

4.1: The Economic Impact of Climate Change and its Mitigation

Indicator 4.1.1: Economic Losses due to Climate-Related Extreme Events

Methods

The Swiss Re Institute provided the data for this indicator. The Swiss Re Institute sigma catastrophe database is an international commercial database recording both natural and man-made disasters from 1970 and has over 12,000 entries.

The term 'natural catastrophe' refers to an event caused by natural forces. Such an event generally results in a large number of individual losses involving many insurance policies. The scale of the losses resulting from a catastrophe depends not only on the severity of the natural forces concerned, but also on man-made factors, such as building design or the efficiency of disaster control in the afflicted region.

Natural catastrophes are categorised as shown in Table 48

Category	Peril Group	Peril
	Earthquake	Earthquake
		Tsunami
		Volcano eruption
	Weather-related	Storm
		Flood
		Hail
		Cold, frost
		Drought, bush fires, heat waves
		Other natural catastrophes

Table 48: Categorisation of natural catastrophes in the data proided by the Swiss Re Institute

For this indicator, only data for 'weather-related' events is presented.

Total (insured and uninsured) economic losses reported by Swiss Re are all the financial losses directly attributable to a major event, i.e., damage to buildings, infrastructure, vehicles etc. This also includes losses due to business interruption as a direct consequence of the property damage. Insured losses are gross of any reinsurance, be it provided by commercial or government schemes. Total loss figures do not include indirect financial losses – i.e., loss of earnings by suppliers due to disabled businesses, estimated shortfalls in GDP and non-economic losses, such as loss of reputation or impaired quality of life. Insured losses refer to all insured losses except liability. To calculate uninsured losses, insured losses are subtracted from total losses.

Data are collected from a variety of sources, both internal and external. These include professional insured claims aggregators as well as insurance associations. Among the sources are also official government data, when available. Economic loss data can be estimated on the basis of Swiss Re proprietary catastrophe risk models. Also, if insured loss data are available, economic loss data are estimated on the basis of the local insurance

penetration and other event-specific information (such as damages to public infrastructure, number of buildings damaged or destroyed etc.).

Minimum threshold apply to inclusion in the database. At least one of the following must apply, for events recorded in 2021 (with economic values changing each year following changes to US CPI):

- **Insured losses (claims):** $ 22.5 million (maritime disasters), $ 45 million (aviation), $ 55.8 million (other)
- **Economic losses**: $ 111.7 million
- **Casualties**: Dead or missing: 20; Injured: 50; Homeless: 2000

Loss values are presented in US$, or if initially expressed in local currency, converted to US$ using year-end exchange rates.

Prior to the 2021 report, country data were then summed into the four World Bank income groups. From the 2021 report, country data are summed into the four HDI classifications (Very High, High, Medium, Low). Further information on the methodology of the sigma explorer database can be found here: https://www.sigma-explorer.com/documentation/Methodology_sigma-explorer.com.pdf. Total insured and uninsured losses are then divided by total GDP for each year and HDI group. GDP data are taken from the IMF's World Economic Outlook (October 2021 Edition). All values are in current prices.

Data

1. Swiss Re Institute sigma catastrophe database[311]
2. IMF World Economic Outlook (October 2021)[312]

Caveats

Only events with measurable economic losses above the threshold levels are included. Each natural catastrophe event recorded is assigned a direct economic loss, and where applicable, an insured loss. Where available, data is taken from official institutions, but where not, estimates are calculated. The process for estimation depends on what data is available. For example, if loss estimates from insurance market data is available, this data may be combined with data on insurance penetration and other event-specific information to estimate total economic losses. If only low-quality information is available, such as a description of the number of homes damaged or destroyed, assumptions on value and costs are made. Some data (including both losses and GDP values) may be revised compared to previous reports, due to updated information or detailed measurement approaches.

Additional analysis

		Insured Losses/$1000 GDP	Uninsured Losses/$1000 GDP
2010	Very High	0.71	0.70
	High	0.13	6.03
	Medium	0.07	6.86
	Low	0.00	0.26
2011	Very High	1.17	0.98
	High	1.17	3.65
	Medium	0.01	2.59
	Low	0.03	0.59

2012	Very High	1.40	1.41
	High	0.07	1.69
	Medium	0.00	1.05
	Low	0.08	1.04
2013	Very High	0.72	0.69
	High	0.20	2.75
	Medium	0.22	1.60
	Low	0.01	0.06
2014	Very High	0.54	0.45
	High	0.13	1.82
	Medium	0.29	4.46
	Low	0.00	0.11
2015	Very High	0.57	0.46
	High	0.06	1.17
	Medium	0.37	1.86
	Low	0.00	0.94
2016	Very High	0.79	0.67
	High	0.12	2.78
	Medium	0.15	1.80
	Low	0.13	2.23
2017	Very High	2.69	2.94
	High	0.13	1.40
	Medium	0.02	1.23
	Low	0.00	0.95
2018	Very High	1.50	1.00
	High	0.05	0.80
	Medium	0.09	1.39
	Low	0.01	0.22
2019	Very High	0.95	0.67
	High	0.04	1.11
	Medium	0.13	3.46
	Low	0.15	3.22
2020	Very High	1.52	0.73
	High	0.15	1.50
	Medium	0.22	5.98

	Low	0.00	0.57
2021	Very High	1.67	1.74
	High	0.10	1.21
	Medium	0.09	1.62
	Low	0.00	0.17

Table 49. Insured and uninsured losses from climate-related extreme events 2010–2021, by HDI group.

Indicator 4.1.2: Costs of Heat-related Mortality

Methods

This indicator used the value of statistical life-year (VSLY) to monetise the years of life lost (YLL) caused by heat-related mortality (data for which is provided by indicator 1.1.6). Compared to last year's method that used the value of a statistical life (VSL) to monetise mortality, the usage of VSLY can reflect age structure differences of heat-related mortalities across countries. VSLY measures how people value the discounted years of remaining life.[313] VSL can be interpreted as the discounted sum of VSLY of each year remained in life, therefore, mathematically, the VSLY can be derived from the VSL and how many remaining years people are expected to live at certain age (Eq.2). As for the change of VSLY to age, some studies assumed that VSLY is constant across age span, while others assumed that VSLY will increase before mid-age and then decrease till death, which is an Inverted-U shape.[314]169 countries spanning six World Health Organization (WHO) regions were included in the estimation. Population and GDP per capita are taken from the World Bank[315]and OECD[316] statistics. The life table used to derive remaining years of life, was taken from WHO.[317]

The same ratio between VSLY and GDP-per-capita is assumed for each country for years 2000–2019, and data from OCED countries was used as the basis to derive the ratio on account of data availability and method consistency across reports in different years. The assumption is shown in Eq. (1), where Y denotes the gross domestic product (GDP) per capita, i denotes the country i in WHO regions, t denotes time.

$$\frac{VSLY_{it}}{Y_{it}} = \frac{VSLY_{OECD}}{Y_{OECD}} \quad (1)$$

The relationship between VSL and VSLY can be obtained by years of remaining life at death (L) and discount rate (r), which was demonstrated in Eq.(2). The average VSL applicable for the OECD countries (VSL_{OECD}) was estimated US$3.83 million ($2015) in 2015, and average GDP per capita for OECD countries was $40,494 ($2015) in 2015. Here it is assumed the VSLY remains constant for each remaining life year because only mortality of people aging over 65 is considered, where the fluctuations of VSLYs are very small even under the Inverted-U assumption[314]. The discount rate used here is 3%.

$$VSLY_{it} = \frac{VSL_{it} \cdot r}{1-(1+r)^{-L}} \quad (2)$$

In order to calculate the monetised value of years of life loss (YLL) relative to per-capita GDP (R), Eq.(3) was applied, where YLL is multiplied by the fixed VSLY-to-GDP per capita-ratio produced by Eq.(1).

$$R_{it} = \frac{VSLY_{it} * YLL_{it}}{Y_{it}} = \frac{VSLY_{OECD}}{Y_{OECD}} * YLL_{it} \quad (3)$$

In order to calculated to monetised value of years of life loss as a proportion of GDP (V), Eq.(4) was applied, where YLL as a proportion of total population (P) is multiplied by the fixed VSLY-to-GDP per capita-ratio in OECD countries.

$$V_{it} = \frac{VSLY_{it} * YLL_{it}}{GNI_{it}} = \frac{VSLY_{it} * YLL_{it}}{Y_{it} * P_{it}} = \frac{VSLY_{OECD}}{Y_{OECD}} * \frac{YLL_{it}}{P_{it}} \quad (4)$$

Country-level results are aggregated according to both WHO regions and HDI level. Considering data availability, some countries in WHO regions are not included: Cabo Verde, Sao Tome and Principe, Saint Vincent and the Grenadines, US Virgin Islands, Samoa, Eritrea, Andorra, Antigua and Barbuda, Bahrain, Barbados, Cook Islands, Dominica, Grenada, Kiribati, Maldives, Malta, Marshall Islands, Micronesia, Monaco, Montenegro, Nauru, Niue, Palau, Saint Kitts and Nevis, Saint Lucia, San Marino, Seychelles, Singapore, South Sudan, Tonga, Tuvalu. The population of these countries accounts for 0.3% of total population in WHO regions.

Data

1. Heat-related mortality data is provided by indicator 1.1.6 in section 1
2. Population in each country are taken from World Bank[315]
3. GDP per capita in OECD members are taken from OECD statistics[316]
4. VSL in OECD are taken from OECD report on Mortality Risk Valuation in Environment, Health and Transport Policies[318]
5. Years of remaining life are obtained from WHO[317]

Additional Analysis

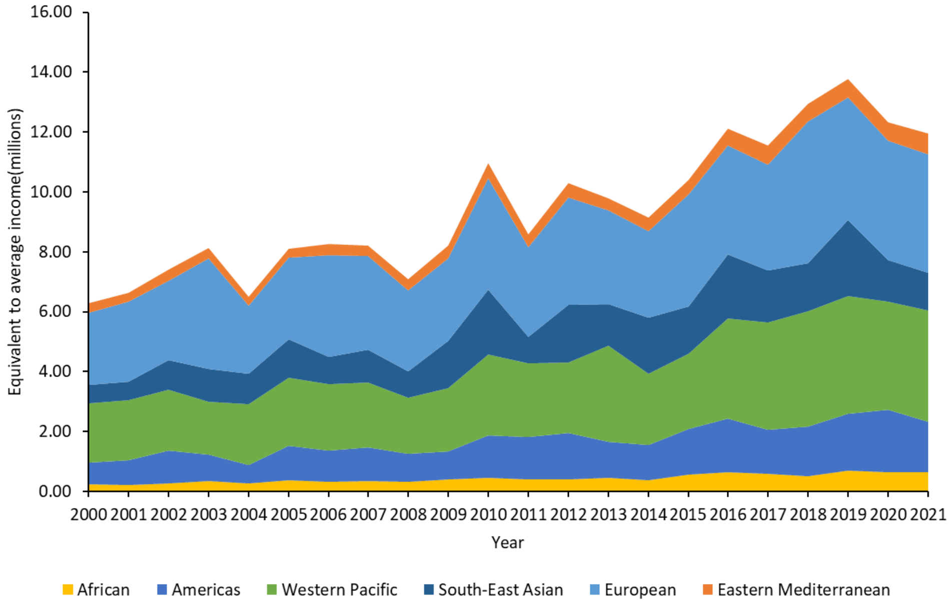

Figure 75: Monetized value of heat-related mortality (in terms of equivalent to GDP per capita) by WHO regions from 2000 to 2021

Caveats

The caveats of this indicator would mainly be in two aspects. Since VSLY is derived from VSL, the uncertainties and ethical concerns on VSL mentioned in last year's caveats also applies to the usage of VSLY. On the other hand, here it is assumed the VSLY is constant at different ages, while some studies argue that the distribution of VSLY to age is Inverted-U shaped.[314] If people under 65 are also taken into account, then the Inverted-U assumption should be considered. The relationship between economic costs of heat-related mortality, per capita GDP across countries, and carbon emissions, was analysed and the correlations were not statistically significant. In the future, with heat-related mortality data with more detailed social groups aggregations, this indicator might explore further inequalities.

Indicator 4.1.3: Potential Loss of Earnings from Heat-Related Labour Capacity Reduction

Methods

Indicator 1.1.4 provides data on heat-related labour capacity loss, in terms of lost work hours, at country scale across four sectors (services, manufacturing, construction and agriculture) for the years 1990-2020 inclusive. In order to calculate potential loss of earnings from this labour capacity loss, it was necessary to compile a dataset of average earnings per hour for each of these countries, sectors and years.

Earnings and income statistics were compiled from the ILOSTAT databases held by the ILO, within the category 'Statistics on Wages'.[319] ILOSTAT includes a number of indicators which are of potential relevance to deriving the average annual hourly wages for the required countries and years. There are variations in the coverage of these indicators, with none having an entirely comprehensive coverage of the countries, sectors and years required for this indicator. Multiple ILOSTAT indicators were therefore used to fill as many gaps as possible. The three main indicator sets used were:

- Mean nominal monthly earnings of employees by sex and economic activity: annual
- Mean nominal monthly earnings of employees by sex and occupation: annual
- Mean nominal hourly earnings of employees by sex and occupation: annual

Within each of these indicator sets, the employment activities most accurately reflecting the four required sectors were selected. In some cases, more than one such activity was available, due to different reporting conventions (for example, the set of activities under ISCO-08 being an update from ISCO-88). Full descriptions of ILO indicators and classifications are available on the ILOSTAT website.[320]

Each indicator and activity was available in US dollar and local currency units. US dollar units were preferred, however in each indicator and activity case, the number of returns in local currency units was slightly higher, so these were selected as well in case more data points could be covered by doing so.

The following tables set out for each of the four employment sectors, the ILOSTAT indicators and activity definitions that were selected in order to supply as much of used the required data as possible. In each table the indicator, activity and currency combinations are arranged in the order of preference with which they were used.

	Indicator	Activity	Currency
1	Mean nominal monthly earnings of employees by sex and economic activity: annual	Aggregate: Trade, transportation, accommodation and food, and business and administrative services	US Dollars
2		Aggregate: Trade, transportation, accommodation and food, and business and administrative services	Local currency
3	Mean nominal monthly earnings of employees by sex and occupation: Annual	ISCO-08: 5. Service and sales workers	US Dollars
4		ISCO-08: 5. Service and sales workers	Local currency
5		ISCO-88: 5. Service workers and shop and market sales workers	US Dollars
6		ISCO-88: 5. Service workers and shop and market sales workers	Local currency
7	Mean nominal hourly earnings of employees by sex and occupation: Annual	ISCO-08: 5. Service and sales workers	US Dollars
8		ISCO-08: 5. Service and sales workers	Local currency
9		ISCO-88: 5. Service workers and shop and market sales workers	US Dollars
10		ISCO-88: 5. Service workers and shop and market sales workers	Local currency
11	Mean nominal monthly earnings of employees by sex and economic activity: annual	ISIC Rev.4: N. Administrative and support service activities	US Dollars
12		ISIC Rev.4: N. Administrative and support service activities	Local currency
13		ISIC Rev. 3.1: K. Real estate, renting and business activities	US Dollars
14		ISIC Rev. 3.1: K. Real estate, renting and business activities	Local currency
15		ISIC Rev.2: 8. Financing, insurance, real estate and business services	US Dollars
16		ISIC Rev.2: 8. Financing, insurance, real estate and business services	Local currency

Table 50: Indicators, activity classes and currencies selected to gather data from the ILOSTAT databases on earnings in the services sector, in order of preference

	Indicator	Activity	Currency
1	Mean nominal monthly earnings of employees by sex and economic activity: annual	Aggregate: Manufacturing	US Dollars
2		Aggregate: Manufacturing	Local currency
3		ISIC Rev.4: C. Manufacturing	US Dollars
4		ISIC Rev.4: C. Manufacturing	Local currency
5		ISIC Rev. 3.1: D. Manufacturing	US Dollars
6		ISIC Rev. 3.1: D. Manufacturing	Local currency
7		ISIC Rev.2: 3. Manufacturing	US Dollars
8		ISIC Rev.2: 3. Manufacturing	Local currency
9	Mean nominal monthly earnings of employees by sex and occupation: Annual	ISCO-08: 8. Plant and machine operators, and assemblers	US Dollars
10		ISCO-08: 8. Plant and machine operators, and assemblers	Local currency
11		ISCO-88: 8. Plant and machine operators and assemblers	US Dollars
12		ISCO-88: 8. Plant and machine operators and assemblers	Local currency
13	Mean nominal hourly earnings of employees by sex and occupation: Annual	ISCO-08: 8. Plant and machine operators, and assemblers	US Dollars
14		ISCO-08: 8. Plant and machine operators, and assemblers	Local currency
15		ISCO-88: 8. Plant and machine operators and assemblers	US Dollars
16		ISCO-88: 8. Plant and machine operators and assemblers	Local currency

Table 51: Indicators, activity classes and currencies selected to gather data from the ILOSTAT databases on earnings in the manufacturing sector, in order of preference

	Indicator	Activity	Currency
1	Mean nominal monthly earnings of employees by sex and economic activity: annual	Aggregate: Agriculture	US Dollars
2		Aggregate: Agriculture	Local currency
3		ISIC Rev.4: A. Agriculture; forestry and fishing	US Dollars
4		ISIC Rev.4: A. Agriculture; forestry and fishing	Local currency
5		ISIC Rev.3.1: A. Agriculture, hunting and forestry	US Dollars
6		ISIC Rev.3.1: A. Agriculture, hunting and forestry	Local currency
7		ISIC Rev.2: 1. Agriculture, hunting, forestry and fishing	US Dollars
8		ISIC Rev.2: 1. Agriculture, hunting, forestry and fishing	Local currency
9	Mean nominal monthly earnings of employees by sex and occupation: Annual	ISCO-08: 6. Skilled agricultural, forestry and fishery workers	US Dollars
10		ISCO-08: 6. Skilled agricultural, forestry and fishery workers	Local currency
11		ISCO-88: 6. Skilled agricultural and fishery workers	US Dollars
12		ISCO-88: 6. Skilled agricultural and fishery workers	Local currency
13	Mean nominal hourly earnings of employees by sex and occupation: Annual	ISCO-08: 6. Skilled agricultural, forestry and fishery workers	US Dollars
14		ISCO-08: 6. Skilled agricultural, forestry and fishery workers	Local currency
15		ISCO-88: 6. Skilled agricultural and fishery workers	US Dollars
16		ISCO-88: 6. Skilled agricultural and fishery workers	Local currency

Table 52: Indicators, activity classes and currencies selected to gather data from the ILOSTAT databases on earnings in the agricultural sector, in order of preference

	Indicator	Activity	Currency
1	Mean nominal monthly earnings of employees by sex and economic activity: annual	Aggregate: Construction	US Dollars
2		Aggregate: Construction	Local currency
3		ISIC Rev.4: F. Construction	US Dollars
4		ISIC Rev.4: F. Construction	Local currency
5		ISIC Rev. 3.1: F. Construction	US Dollars
6		ISIC Rev. 3.1: F. Construction	Local currency
7		ISIC Rev.2: 5. Construction	US Dollars
8		ISIC Rev.2: 5. Construction	Local currency
9	Mean nominal monthly earnings of employees by sex and occupation: Annual	ISCO-08: 9. Elementary occupations	US Dollars
10		ISCO-08: 9. Elementary occupations	Local currency
11		ISCO-88: 9. Elementary occupations	US Dollars
12		ISCO-88: 9. Elementary occupations	Local currency
13	Mean nominal hourly earnings of employees by sex and occupation: Annual	ISCO-08: 9. Elementary occupations	US Dollars
14		ISCO-08: 9. Elementary occupations	Local currency
15		ISCO-88: 9. Elementary occupations	US Dollars
16		ISCO-88: 9. Elementary occupations	Local currency

Table 53: Indicators, activity classes and currencies selected to gather data from the ILOSTAT databases on earnings in the manufacturing sector, in order of preference

A spreadsheet tool was developed to select the relevant data points for all available countries in order of indicator preference – if there was no data point for a given country, year and sector in the first priority indicator, the data point was sought in the next indicator, and so on until a data point was found, or all indicators had been tried.

Monthly earnings data were converted to hourly values using a standard assumption of 40 hours per week and 4.33 weeks per month, i.e., 173.2 hours per month.

Data in nominal local currency units were converted to nominal US dollars at market exchange rates using IMF International Financial Statistics.[321] Nominal US dollar values were converted to real 2021 US dollar values using the US dollar consumer price index from the IMF World Economic Outlook database.[312]

Even after searching 16 variations of ILO indicator, activity and reporting currency for each sector, there were still considerable gaps, with around two thirds of required data points unfilled. In addition, there was a small number of clearly erroneous data points – e.g., with hourly earnings rates orders of magnitude too high, possibly caused by incorrect recording of the currency in which the data were reported, or by episodes of rapid inflation and currency devaluation, with which the recorded market exchange rates were not keeping track.

In order to fill the gaps with no data, as well as to correct data points that were clearly erroneous, a gap filling process was undertaken, using other data points to stand in for the missing or erroneous data. This process was undertaken after all of the data had been corrected to real 2021 US dollar values, so that all of the data were already expressed in constant values. Wherever possible, gaps were filled using data from a different year but from the same sector and country. Where data was available in years before and after the gaps in the same sector and country, linear interpolation was used to fill the gaps. If no future year was available, data were filled using the nearest past year. Likewise, if no previous year was available, the nearest future year was used. If there were no data points available at all for a certain sector or country, the data were taken from the same sector of a different country that was as comparable as possible to the country with missing data. Identification of a reasonably comparable country was achieved primarily by selecting one as close as possible on the HDI scale, within the same or similar region, of a similar size, and with a reasonable number of datapoints. If there were no countries from a similar world region with a similar HDI ranking, the closest possible country on the HDI scale was selected, regardless of its geographic proximity.

A small number of countries have not been given an HDI value and hence could not be included in the analysis.

This process resulted in estimates of hourly earnings for the four sectors, for the years 1990-2021 inclusive, for 188 countries. These hourly earnings data were multiplied by the corresponding values for work hours lost (WHL)

in each country, sector, and year, to provide a quantification of potential earnings lost. The WHLs used assumed that work in the agricultural and construction sectors took place in the sun.

These total lost earnings were expressed as a percentage of the country's GDP in each relevant year. GDP data in nominal US dollars at market exchange rates were downloaded from the IMF World Economic Outlook database,[2] and rendered in constant 2021 US dollars using the GDP deflator index from the same source. Gaps in this GDP data for some countries and years imposed a small further restriction on the coverage of this indicator, and not all of the same countries are available for all years. The maximum country-coverage of the indicator is 183 countries, during the years 2002–2021 inclusive. Results presented as the average value for countries in each of the four HDI groups.

Data

1. Data on working hours lost from indicator 1.1.4
2. Data on earnings by country and sector from ILOSTAT[319]
3. Exchange rate data from IMF International Financial Statistics[321]
4. US Dollar CPI and GDP deflator index from the IMF World Economic Outlook database[312]
5. Country GDP data from the IMF World Economic Outlook database[312]

Caveats

There are several important caveats associated with the analysis:

- The ILOSTAT data do not cover all of the countries, years and sectors required, hence some gap filling was required, as described above. Whilst reasonable care has taken to identify appropriate estimates, these gap filled data are subject to uncertainties
- Whilst reasonable efforts have been made to correct for clearly erroneous data points, the analysis is dependent on the reliability of the ILOSTAT data, which could be subject to uncertainties in reporting, collection and processing
- The use of different combinations of ILOSTAT indicators and activity classes, rather than one single indicator and one activity class per sector, was necessary to increase data coverage as much as possible. Nonetheless this entails risks of inconsistencies, for example associated with different classifications and reporting methods
- The conversion of monthly data to hourly was carried out on the basis of a standard assumption of 4.33 weeks per month, and 40 hours per week. Real monthly working times will vary from these assumptions to a greater or lesser extent in different countries

All of these issues mean that caution should be exercised when examining results for any particular country. In addition, it must be emphasised that the results produced are the *potential* loss of earnings, rather than actual. The indicator is not based on evidence as to whether time off work was in fact taken. Further, if time was taken off work, the bearer of the costs of the lost labour could have varied between countries and sectors. In some instances, workers may have been able to claim sick pay, in which case the losses would have been borne by the employer through paying for non-productive time. In other instances, no arrangements for sick pay may have been in place, in which case it would have been the worker who would have borne the cost through a direct loss of earnings due to the inability to work.

Finally, the indicator by definition is an estimate of potential loss of earnings from formal paid sectors. In many countries informal and unpaid labour is also significant. Such activities could include domestic work and small-scale agriculture.[312,322,323] The impacts on productivity and health of extreme heat on workers involved in so-called informal sectors, would be in addition to the monetised estimates quantified by this indicator.

Additional analysis

The main *Lancet* Countdown report text provides a comparison of the results of the all HDI countries. The following graphs present the same analysis as applied to low, medium, high, and very high HDI countries.

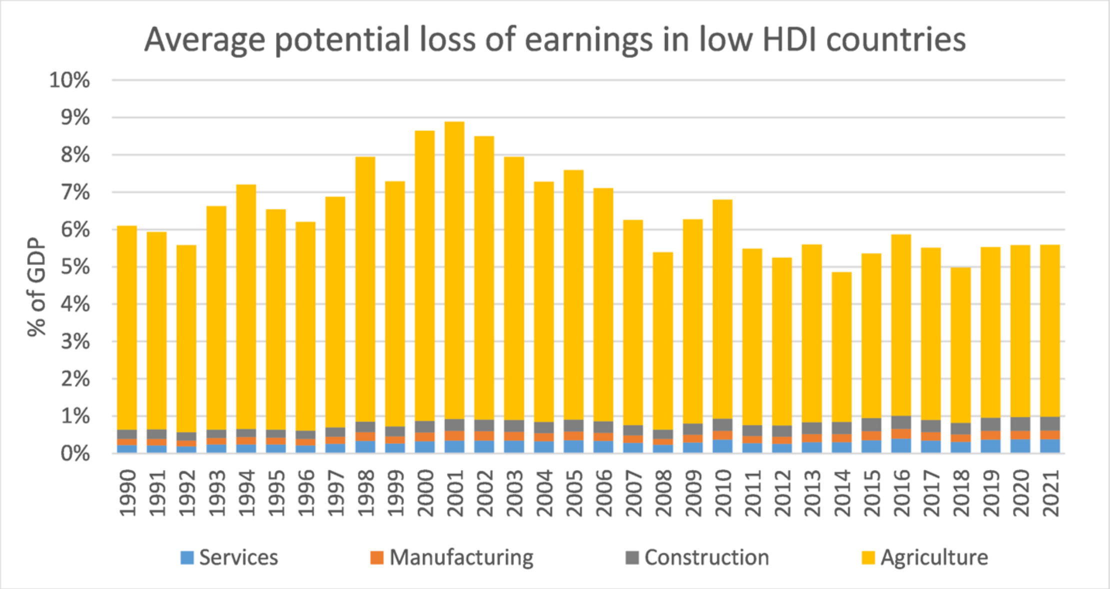

Figure 76: Average potential loss of earnings from heat-related labour capacity reduction as a share of GDP for low HDI countries, by sector of employment

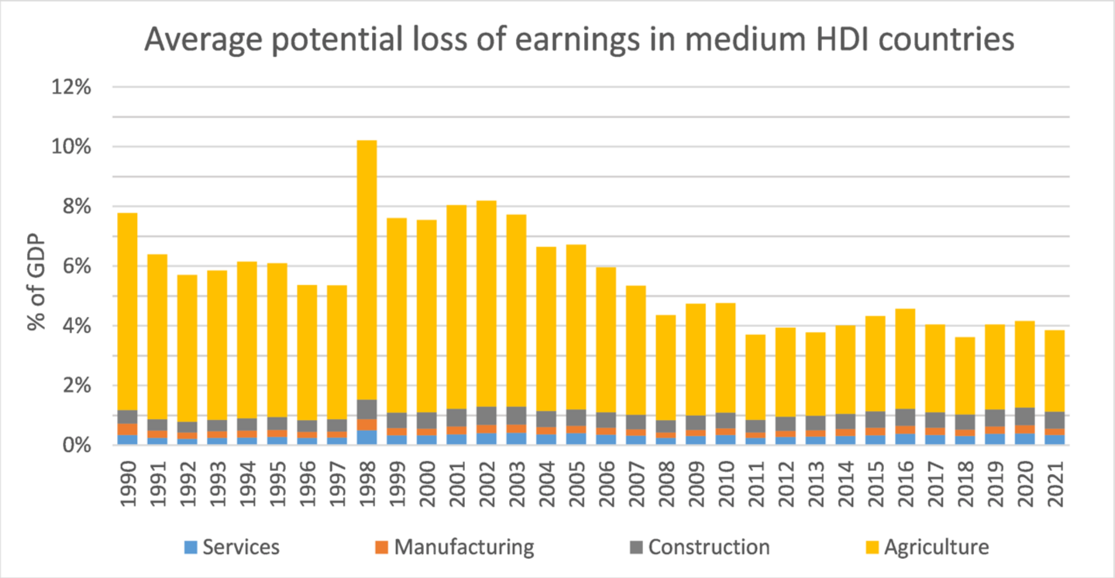

Figure 77. Average potential loss of earnings from heat-related labour capacity reduction as a share of GDP in medium HDI countries, by sector of employment.

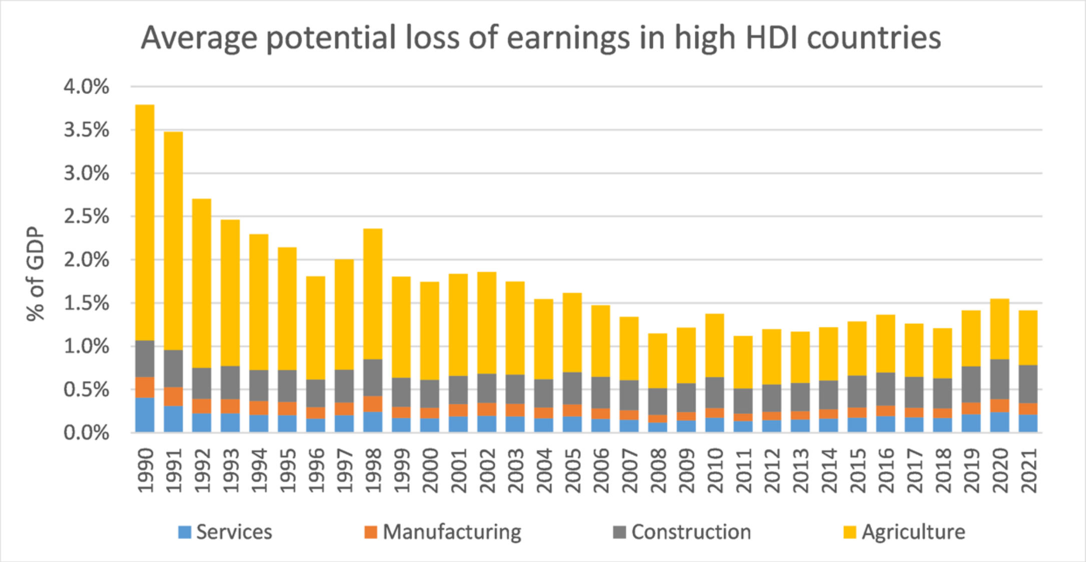

Figure 78. Average potential loss of earnings from heat-related labour capacity reduction as a share of GDP in high HDI countries, by sector of employment.

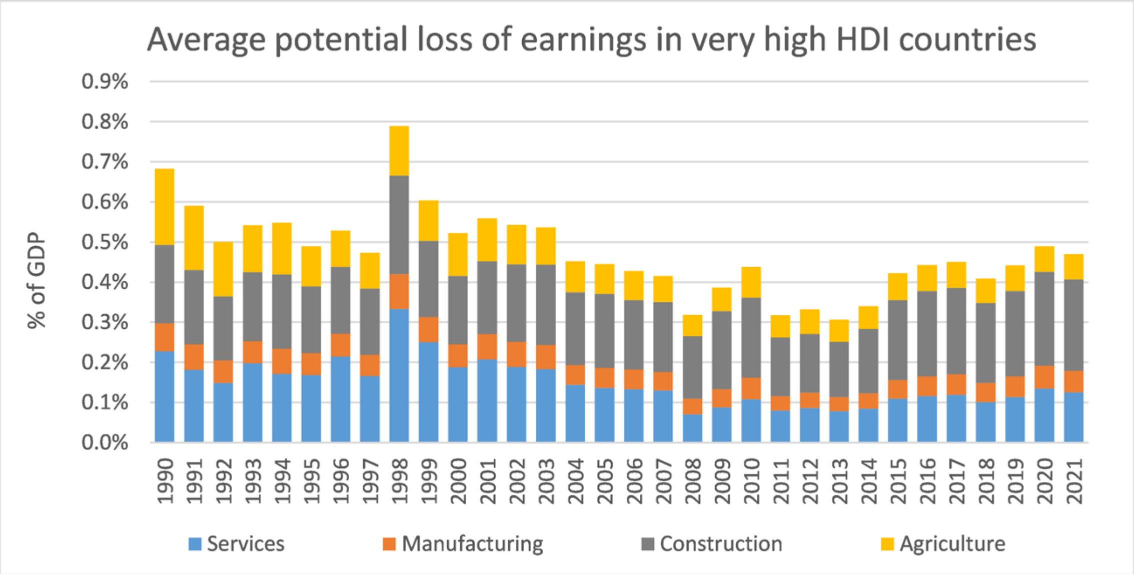

Figure 79. Average potential loss of earnings from heat-related labour capacity reduction as a share of GDP for very high HDI countries, by sector of employment.

Indicator 4.1.4: Costs of the Health Impacts of Air Pollution

Methods

Indicator 3.3 provides data on deaths attributable to both natural and anthropogenic ambient air pollution. Years of life lost (YLLs) were calculated from the age-specific attributable deaths by summing over the remaining life expectancy at the age of death for each attributable death. To determine YLLs attributable to anthropogenic causes only, the total YLLs are reduced to the country- and year-specific proportion of total deaths attributable to anthropogenic sources only in indicator 3.3. The YLLs calculated this way are a conservative estimate since the remaining life expectancy in real world conditions are used, rather than hypothetical conditions with no pollution,

which would be larger. YLLs were calculated for 137 individual countries, for 2019 and 2020. Each country was then classified according to both its HDI category and WHO region (see **Table 54** and **Table 55** below for country classifications). For the WHO region calculations, four ‘rest of world’ regions were also added (see Table 56). It was not possible to use these regions for the HDI classification, due the heterogeneity of classifications of the countries that constitute each region.

The YLLs for each category and region were then summed. To determine the economic value of the YLLs for each category and region relative to per capita average annual income in each, the results were multiplied by the fixed ratio of the Value of a Statistical Life Year (VSLY) to GDP per capita derived by indicator 4.1.2. To calculate the economic value of the YLLs relative to total GDP for each year, the results of this first calculation were multiplied by average GDP per capita (calculated from the sum of GDP for each category and region, inflated to $ 2021 from 2019 and 2020 current prices, divided by the sum of the population for each category and region), and then divided by the sum of GDP in $ 2021 for the category or region in question.

GDP and GDP inflator data were taken from the International Monetary Fund (IMF), and population data were taken from the United Nations (UN). The data and methods used to calculate the fixed ratio between VSLY and GDP per capita are described in indicator 4.1.2.

HDI	Country
Very High	Argentina, Australia, Austria, Belarus, Belgium, Brunei, Darussalam, Bulgaria, Canada, Chile, Croatia, Cyprus, Czechia, Denmark, Estonia, Finland, France, Georgia, Germany, Greece, Hungary, Iceland, Ireland, Israel, Italy, Japan, Kazakhstan, Latvia, Lithuania, Luxembourg, Malaysia, Malta, Mauritius, Montenegro, Netherlands, New Zealand, Norway, Poland, Portugal, Republic of Korea, Romania, Russian Federation, Saudi Arabia, Serbia, Singapore, Slovakia, Slovenia, Spain, Sweden, Switzerland, Turkey, United Kingdom, United States of America, Uruguay
High	Albania, Algeria, Armenia, Azerbaijan, Bolivarian Republic of Venezuela, Bolivia, Bosnia and Herzegovina, Botswana, Brazil, China, Colombia, Ecuador, Egypt, Gabon, Indonesia, Islamic Republic of Iran, Libya, Mexico, Mongolia, North Macedonia, Paraguay, Peru, Philippines, Republic of Moldova, South Africa, Sri Lanka, Thailand, Tunisia, Ukraine, Vietnam
Medium	Angola, Bangladesh, Bhutan, Cabo Verde, Cambodia, Cameroon, Comoros, Congo, Equatorial Guinea, Eswatini, Ghana, India, Kenya, Kyrgyzstan, Lao People's Democratic Republic, Morocco, Myanmar, Namibia, Nepal, Pakistan, Zambia, Zimbabwe
Low	Afghanistan, Benin, Burkina Faso, Burundi, Central African Republic, Chad, Cote d'Ivoire, Democratic Republic of the Congo, Djibouti, Eritrea, Ethiopia, Gambia, Guinea, Guinea Bissau, Lesotho, Liberia, Madagascar, Malawi, Mali, Mauritania, Mozambique, Niger, Nigeria, Rwanda, Senegal, Sierra Leone, Sudan, Togo, Uganda, United Republic of Tanzania

Table 54: countries in each HDI group included in the calculation of costs of air pollution

WHO	Country
African	Algeria, Angola, Benin, Botswana, Burkina Faso, Burundi, Cabo Verde, Cameroon, Central African Republic, Chad, Comoros, Congo, Cote d'Ivoire, Democratic Republic of the Congo, Equatorial Guinea, Eritrea, Eswatini, Ethiopia, Gabon, Gambia, Ghana, Guinea, Guinea Bissau, Kenya, Lesotho, Liberia, Madagascar, Malawi, Mali, Mauritania, Mauritius, Mozambique, Namibia, Niger, Nigeria, Rwanda, Senegal, Sierra Leone, South Africa, Togo, Uganda, United Republic of Tanzania, Zambia, Zimbabwe
Americas	Argentina, Bolivarian Republic of Venezuela, Bolivia, Brazil, Canada, Chile, Colombia, Ecuador, Mexico, Paraguay, Peru, United States of America, Uruguay
Eastern Mediterranean	Afghanistan, Djibouti, Egypt, Islamic Republic of Iran, Libya, Morocco, Pakistan, Saudi Arabia, Somalia, Sudan, Tunisia
European	Albania, Armenia, Austria, Azerbaijan, Belarus, Belgium, Bosnia and Herzegovina, Bulgaria, Croatia, Cyprus, Czechia, Denmark, Estonia, Finland, France, Georgia, Germany, Greece, Hungary, Iceland, Ireland, Israel, Italy, Kazakhstan, Kyrgyzstan, Latvia, Lithuania, Luxembourg, Malta, Montenegro, Netherlands, North Macedonia, Norway, Poland, Portugal, Republic of Moldova, Romania, Russian Federation, Serbia, Slovakia, Slovenia, Spain, Sweden, Switzerland, Turkey, Ukraine, United Kingdom
South-East Asian	Bangladesh, Bhutan, Democratic People's Republic of Korea, India, Indonesia, Myanmar, Nepal, Sri Lanka, Thailand
Western Pacific	Australia, Brunei Darussalam, Cambodia, China, Japan, Lao People's Democratic Republic, Malaysia, Mongolia, New Zealand, Philippines, Republic of Korea, Singapore, Vietnam

Table 55: countries in each WHO region group included in the calculation of costs of air pollution

Region	WHO	Country
1	Americas	Aruba, Barbados, Bahamas, Cuba, Dominican Republic, Grenada, French Guiana*, Guadeloupe*, Guyana, Haiti, Jamaica, Saint Lucia, Martinique*, Puerto Rico*, Suriname, Trinidad and Tobago, Saint Vincent and the Grenadines, United States Virgin Islands*
2	Americas	Belize, Costa Rica, Guatemala, Honduras, Nicaragua, Panama, El Salvador
3	Eastern Mediterranean	Tajikistan, Turkmenistan, Uzbekistan
4	European	United Arab Emirates, Bahrain, Iraq, Jordan, Kuwait, Lebanon, Oman, Occupied Palestinian Territory, Qatar, Syrian Arab Republic*, Yemen

*Population and GDP excluded from the calculations due to lack of data of either one or other data point.

Table 56: countries in each ‘rest of word’ region group included in the calculation of costs of air pollution under WHO calculations

Data

1. IMF World Economic Outlook (October 2021)[312]
2. UN World Population Prospects 2019[244]

Caveats

See indicator 3.3, for caveats related to the calculation of reduced life expectancy.

Caveats regarding the calculation of VSLY are discussed under indicator 4.1.2. Countries not listed in the tables above have been excluded from the analysis, due to the lack of individual characterisation in the model used to calculate YLLs. Democratic People's Republic of Korea is excluded from the WHO regional analysis due to the lack of reliable GDP data (and is not classified under the HDI). Somalia is excluded from the HDI analysis, as it is not classified. Data for 2019 differs to those presented in the 2021 report due to a combination of updated data and improved dose-response functions in the model used to produce YLLs, as described under indicator 3.3

Additional analysis

Table 57 and

Table 58 tabulate the results for each approach, for 2019 and 2020, for the HDI classification and WHO regions, respectively.

	Relative to average annual per-capita income		GDP-equivalent	
	(2019)	(2020)	(2019)	(2020)
Very High	39,879,185	36,889,084	2.6%	2.4%
High	100,657,141	100,290,681	3.51%	3.47%
Medium	166,256,639	158,376,645	7.99%	7.52%
Low	16,393,513	16,106,346	1.88%	1.80%

Table 57: economic value of YLLs by HDI group

	Relative to average annual per-capita income		GDP-equivalent	
	(2019)	(2020)	(2019)	(2020)
African	14,922,340	14,640,200	1.4%	1.3%
Americas	8,510,832	7,853,270	0.8%	0.8%
Eastern Mediterranean	34,446,180	32,605,971	4.9%	4.6%
European	38,251,827	35,763,991	4.1%	3.8%
South-East Asian	155,771,662	148,208,038	7.9%	7.4%
Western Pacific	78,373,529	78,345,880	4.1%	4.1%

Table 58: economic value of YLLs by WHO region

4.2: The Economics of the Transition to Net Zero-Carbon Economies

Indicator 4.2.1: Clean energy investment

Methods

The data for this indicator is sourced from the annual IEA World Energy Investment publication. Key categories of investment are defined as follows

Power sector – investment in coal, oil, gas, nuclear and renewable electricity generation capacity, and electricity networks and battery storage. Renewables includes pumped-hydro storage.

Other supply – investment in coal, natural gas, oil and renewable energy supply for non-electricity purposes. This includes upstream mining, drilling and pipeline infrastructure. Renewable energy includes modern liquid and gaseous bioenergy, low-carbon hydrogen, as well as hydrogen-based fuels that do not emit any CO_2 from fossil fuels directly when used and also emit very little when being produced.

Energy efficiency – An energy efficiency investment is defined as the incremental spending on new energy-efficient equipment or the full cost of refurbishments that reduce energy use.

For most sectors, 'investment' is defined as ongoing capital spending on assets. For some sectors, such as power generation, this investment is spread out evenly from the year in which a new plant or upgrade of an existing one begins its construction to the year in which it becomes operational. For other sources, such as upstream oil and gas and liquefied natural gas (LNG) projects, investment reflects the capital spending incurred over time as production from a new source ramps up or to maintain output from an existing asset. This definition and differs from the definition previously employed by the IEA before 2019, in which investment was defined as overnight capital expenditure.

Data

1. IEA World Energy Investment 2022.[324]

Caveats

Other areas of expenditure, including operation and maintenance, research and development, financing costs, mergers and acquisitions or public markets transactions, are not included. Investment estimates are derived from IEA data for energy demand, supply and trade, and estimates of unit capacity costs, For more information, see EA World Energy Investment 2022.

Additional analysis

Values presented below are in US$2021, billion.

	2015	2016	2017	2018	2019	2020	2021
**Power Sector**	**816**	**837**	**828**	**837**	**854**	**867**	**926**
**Coal**	**80**	**71**	**66**	**65**	**62**	**56**	**52**
**Oil and gas**	**80**	**83**	**77**	**65**	**67**	**55**	**67**
**Nuclear**	**28**	**34**	**37**	**34**	**35**	**40**	**44**
**Renewables**	**310**	**318**	**326**	**359**	**393**	**418**	**446**
**Electricity networks & storage**	**318**	**330**	**322**	**315**	**296**	**298**	**318**
**Other supply**	**1134**	**943**	**951**	**978**	**945**	**739**	**851**
**Coal**	**114**	**95**	**88**	**89**	**105**	**95**	**105**
**Oil**	**592**	**474**	**501**	**526**	**491**	**352**	**421**
**Natural gas**	**351**	**299**	**287**	**290**	**278**	**224**	**252**
**Renewable**	**78**	**76**	**75**	**73**	**72**	**68**	**73**
**Energy Efficiecny**	**264**	**295**	**281**	**266**	**289**	**260**	**328**
**Electrification**	**26**	**27**	**35**	**60**	**58**	**64**	**99**
**Total**	**2,240**	**2,102**	**2,094**	**2,141**	**2,145**	**1,930**	**2,204**

Table 59: Energy investments 2015-2021.

Indicator 4.2.2: Employment in Low-Carbon and High-Carbon Industries

Methods

The data for this indicator is sourced from IRENA (renewables) and IBISWorld (fossil fuel extraction).[325-327]Renewable industries included are:

- Hydropower;
- Solar heating/cooling;
- Solar photovoltaic;
- Wind energy;
- Bioenergy;
- Other technologies.

Bioenergy includes liquid biofuels, soil biomass and biogas. 'Other technologies' includes geothermal energy, ground-based heat pumps, concentrated solar power, municipal and industrial waste, and ocean energy. Fossil fuel extraction values include direct employment, whereas renewable energy jobs include direct and indirect employment (e.g., equipment manufacturing), except for large hydropower (direct employment only).

Due to an improvement in data collection and estimation methodology, employment values reported for fossil fuel extraction are in some years substantially higher than those reported in the 2018 *Lancet* Countdown report. Similarly, an improvement to the methodology for estimating hydropower has altered historic values for Hydropower (previously called 'large' hydropower), and Other Technologies (which previously included small hydropower). From 2018, 'Other Technologies' now also includes employment related to ground-based heat pumps.

Data

1. Data for employment in renewables from IRENA[327]
2. Data for employment in fossil fuel extraction from IBISWorld: oil and gas exploration and production; and coal mining[325,326]

Caveats

Fossil fuel extraction values include direct employment, whereas renewable energy jobs include direct and indirect employment (e.g., equipment manufacturing), with the exception of hydropower.

Future form of the indicator

Additional analysis

	Million Jobs								
	2012	2013	2014	2015	2016	2017	2018	2019	2020
Hydropower	1.66	2.21	2.04	2.16	2.06	1.99	2.05	1.96	2.20
Other Technologies	0.22	.023	0.19	0.2	0.24	0.16	0.18	0.18	0.27
Solar Heating/Cooling	0.89	0.5	0.76	0.94	0.83	0.81	0.8	0.82	0.82
Wind Energy	0.75	0.83	1.03	1.08	1.16	1.15	1.16	1.17	1.25
Bioenergy	2.4	2.5	2.99	2.88	2.74	3.05	3.18	3.58	3.52
Solar Photovoltaic	1.36	2.27	2.49	2.77	3.09	3.37	3.68	3.75	3.98

Fossil Fuel Extraction	11.81	12.19	12.41	12.30	12.26	11.98	12.00	11.67	10.53

Table 60: Employment in renewable energy and fossil fuel extraction industries.

Indicator 4.2.3: Funds Divested from Fossil Fuels

Methods

The data for this indicator is collected and provided by stand.earth and 350.org.[328] Prior to this report, they represented the total assets (or assets under management, AUM) for institutions that have publicly committed to divest (for which data is available), with non-US$ values converted using the market exchange rate when the commitment was made, and thus did not directly represent the actual sums divested from fossil fuel companies. For the data used in this report, AUM data has been updated to 2021 levels. A company is committed to 'divestment' if it falls into any of the following five categories:

- 'Fossil Free' - An institution or corporation that does not have any investments (direct ownership, shares, commingled mutual funds containing shares, corporate bonds) in fossil fuel companies (coal, oil, natural gas) and committed to avoid any fossil fuel investments in the future

- 'Full' - An institution or corporation that made a binding commitment to divest (direct ownership, shares, commingled mutual funds containing shares, corporate bonds) from any fossil fuel company (coal, oil, natural gas).

- 'Partial' - An institution or corporation that made a binding commitment to divest across asset classes from some fossil fuel companies (coal, oil, natural gas), or to divest from all fossil fuel companies (coal, oil, natural gas), but only in specific asset classes (e.g. direct investments, domestic equity).

- 'Coal and Tar Sands' - An institution or corporation that made a binding commitment to divest (direct ownership, shares, commingled mutual funds containing shares, corporate bonds) from any coal and tar sands companies.

- 'Coal only' - An institution or corporation that made a binding commitment to divest (direct ownership, shares, commingled mutual funds containing shares, corporate bonds) from any coal companies.

Eight organisations that were originally recorded as non-healthcare institutions have been considered as such for the purpose of this indicator (London School of Hygiene and Tropical Medicine, The Royal College of General Practitioners, New Zealand Nurses Organisation, HESTA, HCF, Berliner Ärzteversorgung, Doctors for the Environment Australia, and the Royal College of Emergency Medicine). Divestment commitments by the American Medical Association, which divested in 2018, was not included in the data provided by 350.org, and was added separately.

Data

1. Stand.earth and 350.org Global Fossil Fuel Divestment Commitments Database[328]

Caveats

Data on the number of institutions that have divested, and the value of their assets is dependent on institutions reporting this information to Stand.earth and 350.org.

Additional analysis

The cumulative value of divestment (both global total and for healthcare institutions) is presented below (**Table 61**). Organisations that have divested but for which no date of divestment (a total of $2.56 billion) are recorded in a separate column, with the total assumed to begin in 2008 in the absence of more detailed information.

	US$ million (2021 data)		
	**Global**	**Global (including data with no divestment date)**	**Healthcare Institutions**
2008	$16	$2,562,392	$-
2009	$17	$2,562,392	$-
2010	$17	$2,562,392	$-
2011	$84	$2,562,460	$-
2012	$3,773	$2,566,148	$-
2013	$9,337	$2,571,712	$-
2014	$441,744	$3,004,120	$37,809
2015	$2,569,415	$5,131,791	$38,103
2016	$3,559,418	$6,121,794	$41,010
2017	$5,571,770	$8,134,145	$53,189
2018	$8,873,411	$11,435,787	$54,093
2019	$12,421,949	$14,984,325	$54,105
2020	$28,445,346	$31,007,722	$54,173
2021	$37,865,431	$40,427,807	$54,185

Table 61: Cumulative fossil fuel divestment.

Due to confidentiality issues, the full dataset is not available for publication. However, interested readers may visit the www.divestmentdatabase.org for further information.

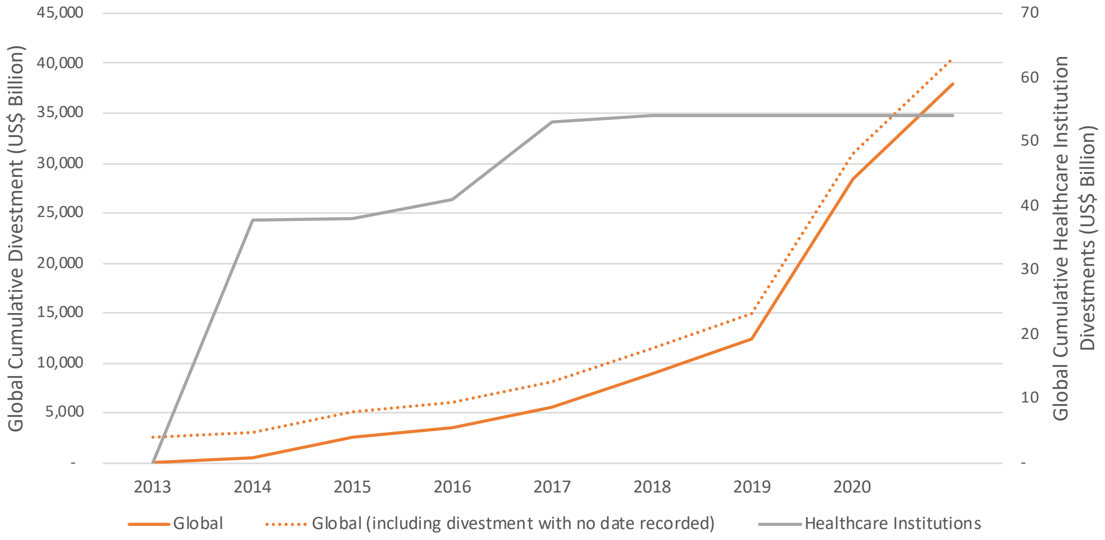

Figure 80. Cumulative divestment – Global total and in healthcare institutions

Indicator 4.2.4: Net Value of Fossil Fuel Subsidies and Carbon Prices

Methods

Fossil Fuel Subsidies

Data for fossil fuel subsidies were taken from two sources. The IEA provides data on fossil fuel consumption subsidies for 42 countries,[329] calculated using its 'price gap' approach – the difference between the end-user prices paid for fossil fuels in the country, and reference prices that account for the full cost of supply.[330] However, the countries provided in this list are mainly non-OECD. The OECD itself provides estimates of fossil fuel subsidies within the 37 OECD countries, plus Argentina, Armenia, Azerbaijan, Belarus, Brazil, China, Georgia, India, Indonesia, Moldova, Russia, South Africa and Ukraine—a total of 50 countries.[331] OECD's estimates are derived from a bottom-up inventory of subsidy mechanisms within each country, and include production and consumption support, infrastructure investments, incentives and R&D. It divides the type of support into three broad categories: Consumer Support Estimate (CSE), Producer Support Estimate (PSE) and General Services Support Estimate (GSE).

Combining the IEA and OECD datasets allows a coverage of 80 countries, after accounting for overlaps and the omission of countries not covered by the *Lancet* Countdown. The OECD describes an approach for combining these two datasets, and reconciling different estimates for the countries covered by both.[332] This involves selecting line items in the OECD inventory that correspond to the price-gap definition of subsidies that is the basis of the IEA data – i.e. measures that bring about reduced consumer prices: 'conceptually, an OECD estimate derived from individual measures that capture transfers to consumers from producers and taxpayers should match the IEA price-gap estimates' (p.22-3).[332]

The description of this approach suggests that in the few cases of countries whose subsidies have been calculated by both OECD and IEA, the OECD estimate would be expected to be the larger of the two.[332] However, analysis of overlapping countries suggests that it is in fact more often the IEA estimate that is larger. This analysis is described in more detail in the appendix of the 2020 *Lancet* Countdown report. The conclusion drawn from this is that attempting to separate some line items from the OECD estimates that seem more directed at consumers is not a reliable way of reconciling the two estimates – on the contrary, in several cases it makes the gap between the two larger by making the OECD estimate smaller. Consequently, in considering countries that overlap between the two datasets as part of preparing this indicator, a comparison was made simply between the total OECD estimate and the total IEA estimate.

Following a simple rule of thumb proposed by OECD, in order to decide which estimate to use in overlapping cases, the source that produces the larger cumulative total for a given country over the years being considered, was the one chosen as the source for that country for this indicator.[332]

Carbon prices and revenues

Information on carbon prices and carbon pricing revenues was sourced from the World Bank Carbon Pricing Dashboard.[333] Revenues from each recorded instrument were allocated to the nation state within which the instrument operated. Shares of the EU ETS revenues were allocated to each of the participants in the EU ETS – that is the 28 members of the EU (which included the UK for the years considered in this analysis), plus Iceland and Norway. Liechtenstein is also an EU ETS member but could not be included in this analysis due to lack of CO_2 emissions data. The allocation of EU ETS revenues was made to participating states on the basis of their share of the emissions of all EU ETS states, calculated using IEA CO_2 emissions data.[334] This was considered an acceptable simplification given that for the period 2013–2020, 88% of allowances were allocated for auction to participating states in proportion to their emissions.[335]

Countries were included in the analysis if data were available for CO_2 emissions, and either fossil fuel subsidies or carbon pricing instruments. This yielded a list of 86 countries accounting for 92% of global CO_2 emissions in 2019.[334]

Net carbon price and revenue calculations

In reality at present, both carbon prices and fossil subsidies are typically applied to individual sectors or fuels, and do not cover the entire economy. Within different particular jurisdictions the sectors covered by subsidies and

carbon prices are often not identical. As such the only way of producing a consistent indicator across multiple countries was to average out both subsidies and prices across the CO_2 emissions of the whole economy, resulting in net average economy-wide carbon prices and revenues. Each country's total fossil fuel subsidies were subtracted from its total carbon price revenues to produce a net carbon revenue. These figures were divided by the relevant total country CO_2 emissions for each year, using data from the IEA,[334] resulting in the net carbon price. The net carbon revenue was expressed as a proportion of national expenditure on health, using current annual (i.e. not including capital) health expenditure data from the WHO's Global Health Expenditure Database.[336]

Currency standardisation

All money values are expressed in real 2021 US$. Both the OECD Inventory and the IEA fossil fuel subsidy database provide data in real 2020 US$. These units were corrected to real 2021 values, using the GDP deflator for the US dollar, from the IMF.[312] The World Bank carbon pricing revenue data and the WHO health expenditure data are given in nominal US dollars, so again the US GDP deflator from IMF[312] was applied to correct to real 2021 values.

Data

1. Fossil fuel subsidies data from the IEA[329] and OECD[331]
2. Carbon pricing data from the World Bank Carbon Pricing Dashboard[333]
3. CO_2 emissions from fuel combustion from IEA[334]
4. Health expenditure data from WHO[336]
5. US Dollar GDP deflator index from the IMF World Economic Outlook database[312]

Caveats

The principal caveat is that the indicator is strongly dependent on the reliability of the main datasets from the IEA, OECD and World Bank. It is possible that data on individual countries may not be fully comprehensive due to reporting errors, lack of information or other issues, as indeed is acknowledged by OECD.[332] The indicator should be considered as a way of illustrating global trends, and caution should be exercised in attempting to draw out specific conclusions relating to individual countries covered by the indicator.

The nature of indicators that draw on multiple datasets is that the most recent year on which they can report is defined by the most recent year that is common to all datasets used. In this case that year was 2019, which was due to this being the most recent complete year for both CO_2 emissions from fuel combustion and health expenditure.

The economy-wide net carbon price was derived by dividing fossil fuel subsidies and carbon pricing revenues by total CO_2 emissions. This fits well with the subsidies, as these are for fossil fuels, the principal source of CO_2. However, some of the carbon pricing instruments from which the revenue was assessed are not only for fossil fuel combustion but apply to other sectors and non-CO_2 gases. There is therefore a slight inconsistency between the sectoral coverage of the subsidies and the carbon pricing instruments.

Additional analysis

The relevant section in the main report shows net carbon prices, net carbon revenues, and net carbon revenues as a proportion of health spending, by 2019 HDI grouping, for the year 2019. The following graphs show results for the same three indicators with all countries grouped together, and for the years 2010–2019 inclusive. Results for years 2016–2017 differ from those reported in the 2020 Countdown report due to an increased number of countries included in the analysis.

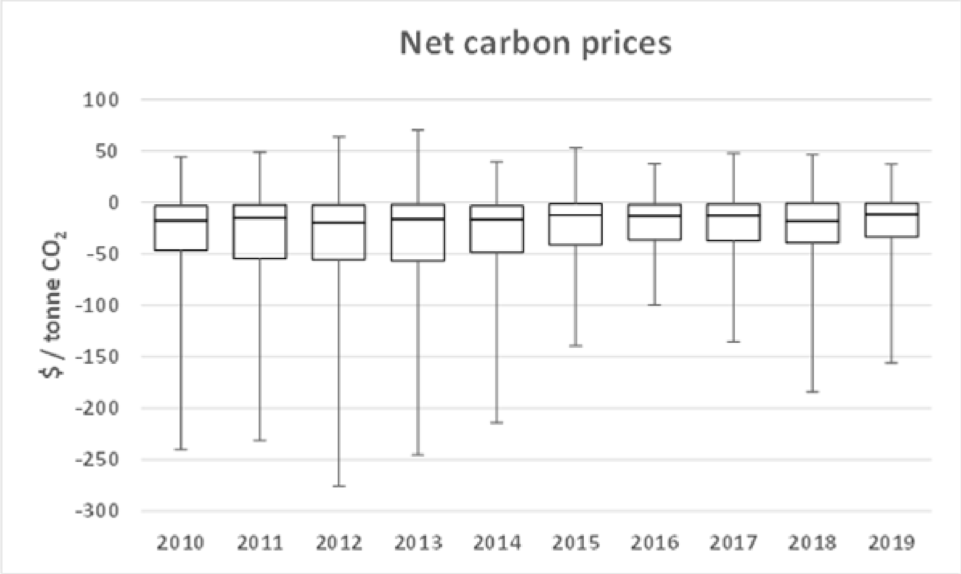

Figure 81. Net carbon prices for all countries included in the analysis, 2010–2019 inclusive. Boxes show the interquartile range (IQR), horizontal lines inside the boxes show the medians, and the brackets represent the full range from minimum to maximum.

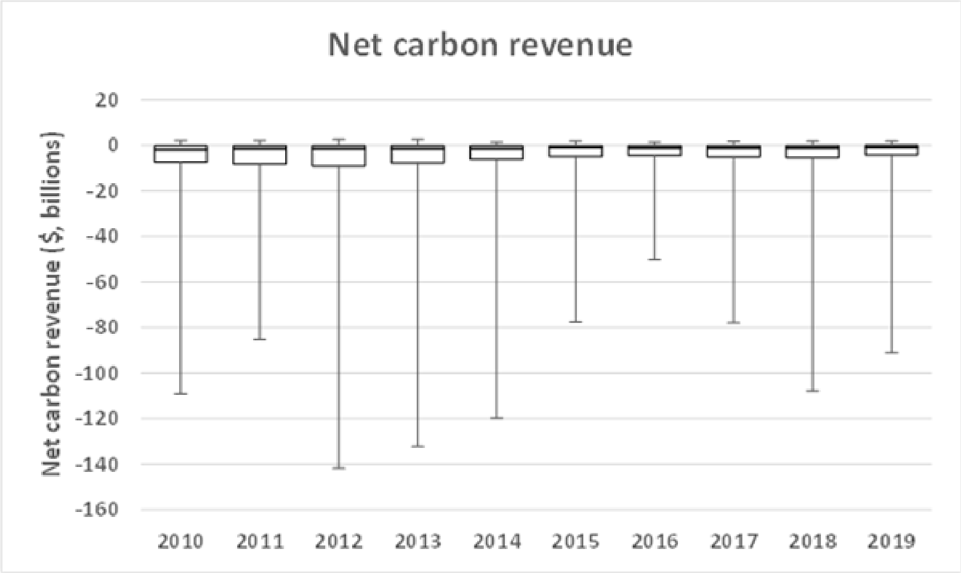

Figure 82. Net carbon revenue for all countries included in the analysis, 2010–2019 inclusive. Boxes show the interquartile range (IQR), horizontal lines inside the boxes show the medians, and the brackets represent the full range from minimum to maximum.

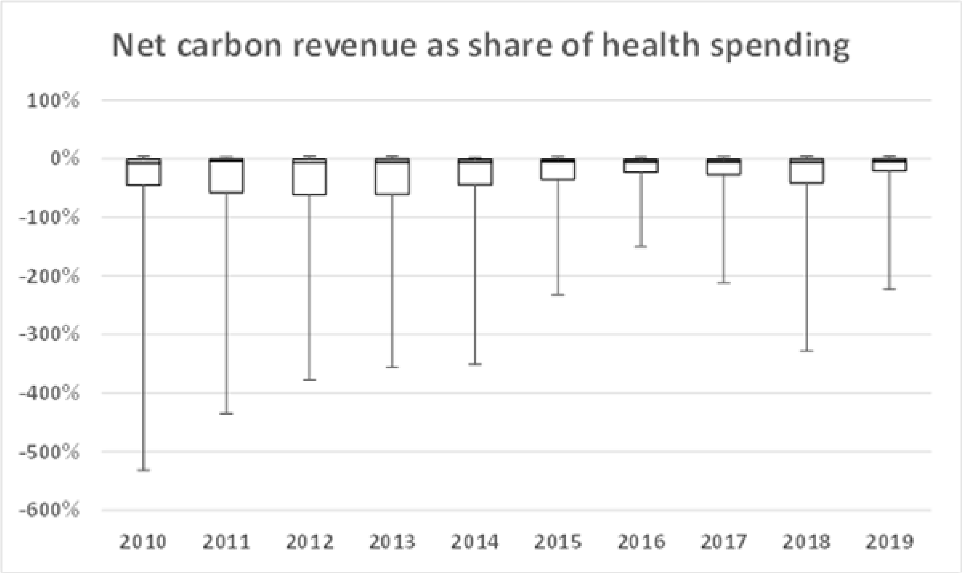

Figure 83. Net carbon revenue expressed as the equivalent share of current (i.e., not capital) annual health spending, for all countries included in the analysis, 2010–2019 inclusive. Boxes show the interquartile range (IQR), horizontal lines inside the boxes show the medians, and the brackets represent the full range from minimum to maximum.

Indicator 4.2.5: Production and Consumption-based Attribution of CO_2 and $PM_{2.5}$ Emissions

Methods

Environmentally Extended Multi-Regional Input-Output Analysis

There are two approaches to measure emissions: production-based (sometimes referred to as territorial-based) accounting and consumption-based accounting. Production-based emissions occur within the geographical territory of a nation, while consumption-based emissions encompass the emissions from the nation's domestic final consumption, as well as those caused by the production of its imports. Since both CO_2 emissions via climate change, and air pollution directly, are detrimental to human health, understanding of the responsibilities of emissions across borders is crucial in the globalised world. This indicator estimates $PM_{2.5}$ and CO_2 emissions embodied in international trade, and then calculates national $PM_{2.5}$ and CO_2 emissions from the consumption perspective. Thus, the responsibility of these emissions and the associated environmental and human health consequences can be distributed for international environmental policy formulation.

Environmentally extended multi-regional input-output (EEMRIO) analysis is used in the calculation of consumption-based emissions.[337] The EEMRIO analysis can reflect production and consumption structures and interdependencies between economic sectors across regions. The relationships between final use and emissions are estimated via Leontief inverse matrix, which is expressed as follows in equation (1):

$$\mathrm{C} = \mathrm{E} \cdot \mathrm{L} \cdot \mathrm{F} = \mathrm{E} \cdot (\mathrm{I} - \mathrm{A})^{-1} \cdot \mathrm{F} \qquad \textbf{(1)}$$

C is the total consumption-based emissions, CO_2 or $PM_{2.5}$ emissions in this case. It is mapped directly to emissions inventories. *E* is the row vector of the production-based emission intensity defined as the emissions per unit of output. *F* is the vector of final demand. and *L* is the Leontief inverse matrix calculated by $(I\text{-}A)^{-1}$, where *I* is the identity matrix, and *A* is the technical coefficient matrix describing the inter-sectoral and inter-regional flows per unit of output.

Consumption-based accounting encompasses emissions from domestic final consumption and those caused by the production of its imports, while production-based accounting measures emissions which take place within national territory. The above relationship can also be expressed as follows:

$$C_{CBA} = C_{PBA} - C_{exp} + C_{imp} \quad \textbf{(2)}$$

where C_{CBA} is the consumption-based emissions, C_{imp} is the emissions embodied in imports, C_{PBA} is the production-based emissions, and C_{exp} is the emissions embodied in exports.

Emission Inventory Mapping with GAINS

To construct the production-based $PM_{2.5}$ emission inventory with the GAINS model,[228] the workflow illustrated in Figure 84 is followed. First, an intermediary aggregation level to which emissions from the GAINS source categories are aggregated is defined.[i] In a second step these aggregated or grouped emissions are distributed among the relevant MRIO sectors according to a specific rule. This process is repeated until the emissions from all relevant GAINS source categories havWG4 e been mapped to the relevant MRIO sectors.

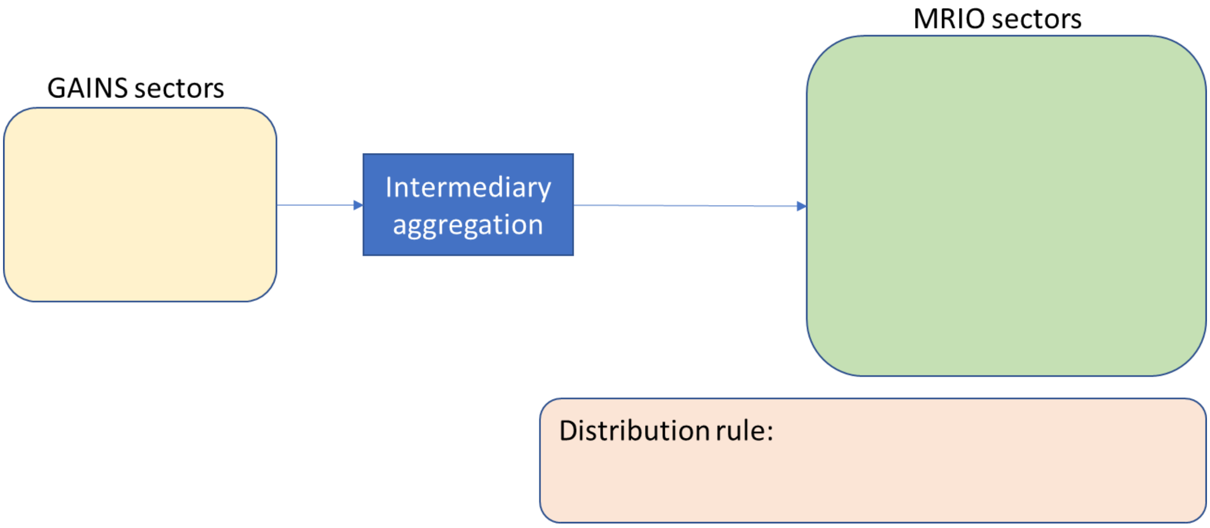

Figure 84. Generic approach for mapping the GAINS sectoral emissions to MRIO sectors.

In practice, the GAINS source categories are clustered into three groups, so that there are three rounds of mappings. These groupings correspond to energy-related emissions (except trucking, see below), process-related emissions, and trucking-related emissions. In a final step, for each MRIO sector the contributions from the three rounds of mappings are summed so that a total emission can be associated with each MRIO sector. In all calculations determining the relative energy share of an MRIO sector in the total energy, the use of electricity is ignored, since the emissions from electricity production are accounted for elsewhere.

On the GAINS side, trucking is related to the sectors TRA_RD_HDT and TRA_RD_LD4T and the fuel-related activities, such as diesel, gasoline, LPG etc, as well as km-related emissions such as abrasion, tyres and braking. On the MRIO side, diesel consumption from road transport by MRIO sector is used to determine the share of each sector in the total. In some countries significant amounts of diesel is also used by cars, a fact that is neglected here. Figure 85 illustrates the mapping process for trucking-related emissions between GAINS and the MRIO sectors.

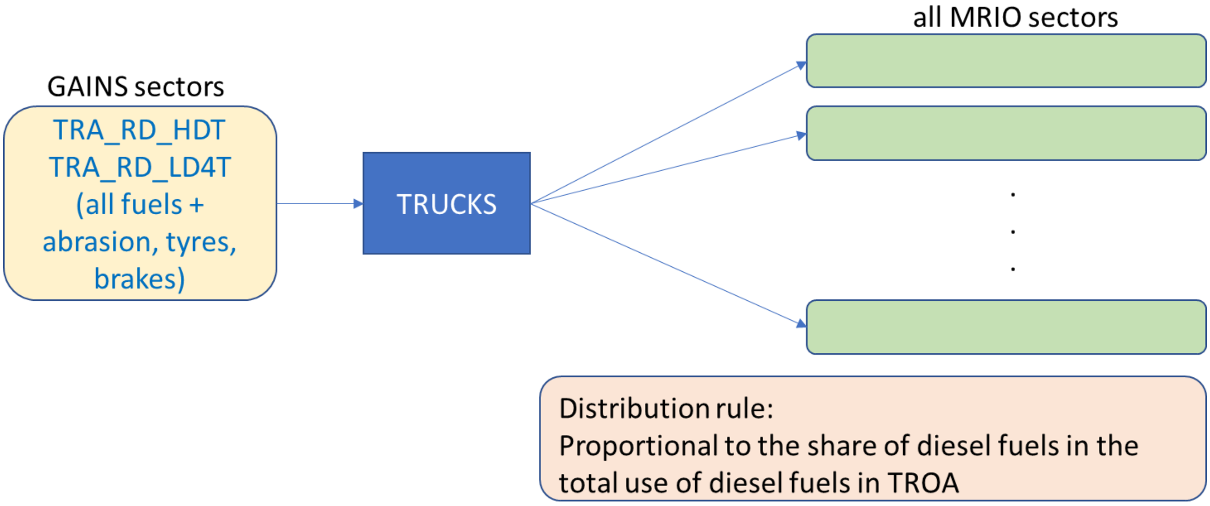

Figure 85 Mapping of trucking-related emissions

The trucking-related emissions in region *r* for MRIO sector *m* are thus calculated as:

$$\mathrm{Em}_r(m,t) = \mathrm{Em}_r(\mathrm{TRUCKS}) \cdot \mathrm{sh}_r(m,\mathrm{diesel}) \qquad \textbf{(3)}$$

where

$$\mathrm{sh}_r(m,\mathrm{diesel}) = \frac{\mathrm{TROA}_r(m,\mathrm{diesel})}{\sum_{m'} \mathrm{TROA}_r(m',\mathrm{diesel})} \qquad \textbf{(4)}$$

is the share of sector *m* in the road transport related diesel consumption in region *r*, and $\mathrm{Em}_r(\mathrm{TRUCKS})$ are the total trucking related emissions in region *r* as calculated by GAINS.

Once the trucking-related emissions and energy use has been separated out what is relevant for distributing the remaining energy (but not trucking-related emissions) is generally the total final energy consumption minus the diesel consumption in TROA. Thus, non-trucking related final energy consumption excluding electricity is referred to as the relevant final energy consumption in each MRIO sector that is used to determine the shares for distributing energy-related emissions into MRIO sectors.

In the mapping of energy-related emissions, intermediary clusters for energy-related emissions are defined as follow:

Table 62. Aggregated energy-related sectors, their description and coverage in terms of GAINS sectors as well as MRIO clusters.

**Label**	**Description**	**GAINS sector coverage**	**MRIO clusters**
ELE_COAL	Coal-fired power plants	All power plants combusting coal or solid biomass[ii]	coal_electricity

[ii] It seems that no specific provision for biomass was made and thus it is included here.

ELE_OIL	Oil-fired power plants	All power plants combusting heavy fuel oil or diesel	oil_electricity
ELE_GAS	Gas-fired power plants	All power plants combusting natural gas	gas_electricity
AGR_MACH	Agricultural machinery	TRA_OT_AGR, DOM_OTH	cultivation + livestock_farming + items_dom_oth
IND_IS	Iron and steel industry	IN_OC_ISTE	manuf_is
IND_NFME	Non-ferrous metals	IN_OC_NFME	manuf_nfme
IND_NMMI	Non-metallic minerals	IN_OC_NMMI[iii]	manuf_bricks + manuf_cem + manuf_nmmi
IND_CHEM	Chemical industries	IN_BO_CHEM, IN_OC_CHEM	manuf_chem + manuf_fert + manuf_chem_nec
IND_CON	Conversion industries, incl. refineries	IN_BO_CON, CON_COMB	ind_conversion
PPAPER	Pulp and paper	IN_BO_PAP, IN_OC_PAP	manuf_paper
OTH_IND	Other industries	All IN_XX_OTH sectors	other_industries
SERVICES	Services	DOM_COM subsectors, MSW	items_services
RAIL	Trains	TRA_OT_RAI	rail
Ships	Sea-going ships	TRA_OTS_X	ships
INW	Ships on inland waterways	TRA_OT_INW	inw
CONSTRUCTION	Construction machinery	TRA_OT_CNS, TRA_OT_LD2, TRA_OTH_LB	construction

The following approach is used for the mapping. Emissions from GAINS sectors (third column Table 62) are aggregated to an intermediary sector (first column) and then distributed among the MRIO sectors belonging to the clusters in the final column using their relative shares in the energy consumption. This is illustrated further for agricultural machinery and combustion devices in Figure 86.

[iii] In GAINS energy-related emissions in NMMI (largely cement production) are all absorbed into process-related emissions, see below.

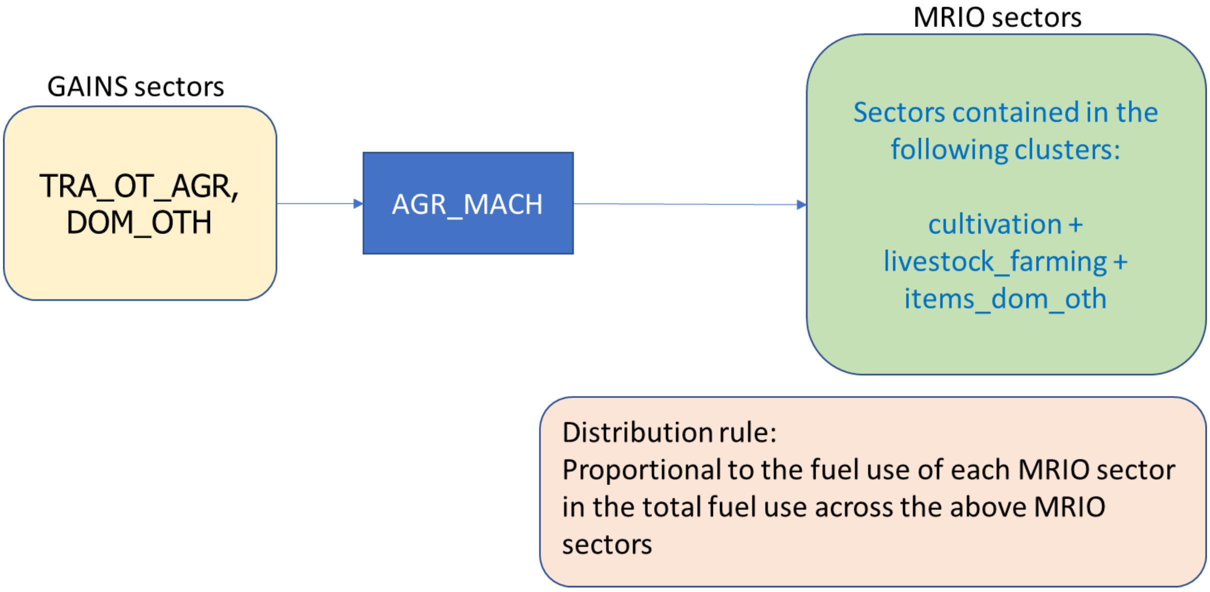

Figure 86 Approach for distributing emissions from agricultural machinery and devices (mobile and stationary) to MRIO sectors.

The energy related emissions in region *r* for MRIO sector *m* are thus:

$$\mathrm{Em}_r(m, e) = \sum_{label} \mathrm{Em}_r(\mathrm{label, e}) \cdot \mathrm{sh}_r(m, \mathrm{e, label}) \qquad \textbf{(5)}$$

Where the sum is running over all labels given in Table 62 and the share

$$\mathrm{sh}_r(m, \mathrm{label, e}) = \frac{\mathrm{FE}_r^*(m, label)}{\sum_{m'} \mathrm{FE}_r^*(m', label)} \qquad \textbf{(6)}$$

is the share of MRIO sector *m* in the final energy demand (minus trucking) in the total final energy demand (minus trucking) in cluster *label* in region *r*.

Process-related emissions are calculated in GAINS separately from energy-related emissions, i.e., there are separate source categories for these in GAINS. Again, intermediary aggregation sectors, this time relevant for the processes, are defined as follows:

**Label**	**Description**	**GAINS sector coverage**	**MRIO clusters**
AGR_PROC	Process emissions related to cultivation	FCON_X, AGR_ARABLE, WASTE_AGR, APPLIC_X, GRAZE_X, STH_NPK, STH_AGR	cultivation
PROC_CATTLE	Emissions related to cattle farming	AGR_COWS, AGR_BEEF	Cattle farming (single sector)
PROC_PIG	Emissions related to pig farming	AGR_PIGS	Pigs farming (single sector)
PROC_POULT	Emissions related to poultry farming	AGR_POULT	Poultry farming (single sector)

PROC_OTANI	Emissions related to farming of other animals	AGR_OTANI	Meat animals nec (single sector)
PROC_BRICK	Emissions related to brick production	PR_BRICK	manuf_bricks
PROC_CEM	Emissions related to cement production	PR_CEM, PR_LIME	manuf_cem
PROC_NMMI	Emissions related to other non-metallic minerals	PR_NMMI, PR_GLASS	manuf_nmmi
PROC_IS	Emissions related to iron and steel production	PR_EARC, PR_BAOX, PR_HEARTH, PR_CAST, PR_SINT, PR_SINT_F, PR_PIGI, PR_PIGI_F, PR_CAST_F	manuf_is
PROC_ALU	Emissions related to aluminium production	PR_ALPRIM, PR_ALSEC	manuf_alu
PROC_FERT	Emissions related to fertilizer production	PR_FERT, FERTPRO	manuf_fert
PROC_CHEM	Emissions related to other chemical processes	PR_SUAC, PR_CBLACK	manuf_chem
PROC_PULP	Emissions related to paper and pulp production	PR_PULP	manuf_paper
PROC_CONVERSION	Emissions related to energy conversion	PR_REF, PR_COKE, STH_COAL, PR_PELL	ind_conversion
PROC_COAL_MINE	Emissions related to coal mining	MINE_HC, MINE_BC, PR_BRIQ	mining_coal_io
PROC_OTHER_MINE	Emissions related to other mining	STH_FEORE, MINE_OTH, STH_OTH_IN	mining_other_io
PROC_SM_IND	Emissions related to other small industries	PR_SMIND_F, OTHER_VOC, PR_OT_NFME, PR_OTHER, OTHER_PM	other_industries
PROC_CONSTRUCT	Emissions related to construction activities	CONSTRUCT	construction

Table 63 Aggregated process-related sectors, their description and coverage in terms of GAINS sectors as well as MRIO clusters.

The process related emissions in region *r* for MRIO sector *m* are thus:

$$\mathrm{Em}_r(m,p) = \sum_{label} \mathrm{Em}_r(\mathrm{label},\mathrm{p}) \cdot \mathrm{sh}_r(m,\mathrm{e},\mathrm{label}) \quad \textbf{(7)}$$

Where the sum is running over all labels given in Table 63 and the share

$$\mathrm{sh}_r(m, \mathrm{label}, \mathrm{e}) = \frac{\mathrm{FE}_r^*(m, label)}{\sum_{m'} \mathrm{FE}_r^*(m', label)} \quad \textbf{(8)}$$

is the share of MRIO sector *m* in the final energy demand (minus trucking) in the total final energy demand (minus trucking) in cluster *label* in region *r*. The main difference to the energy related emissions is that the clusters are different, and thus the shares for each sector within a cluster may be different.

As noted above it is a simplification to distribute the process emissions proportional to the energy use in the MRIO sector within its corresponding cluster, and refinements could be made on the basis of information which of the MRIO sectors within a cluster are mostly related to the process emissions and in which proportion.

The total emissions associated with MRIO sector *m* is then simply the sum of the above energy-related, process-related, and trucking-related emissions of $PM_{2.5}$:

$$\mathrm{Em}_r(m) = \mathrm{Em}_r(m, e) + \mathrm{Em}_r(m, p) + \mathrm{Em}_r(m, t) \quad \textbf{(9)}$$

EXIOBASE[338] is used for the global MRIO table and CO_2 emission inventory for the year 2020. In EXIOBASE, 44 territories and 5 rest of the world regions are covered in the resolution of 163 industrial sectors. The associated CO_2 emission inventory is mapped on a one-to-one sectorial resolution. Hence, consumption-based CO_2 can be easily obtained using equation (1).

To present the results in HDI country groups, the 44 territories are aggregated in accordance with HDI classification developed by UNDP. In the case of the 5 rest of the world regions, disaggregation of both consumption-based and production-based CO_2 inventories has been conducted in proportion to the national total 2020 production-based CO_2 emissions provided by the Global Carbon Project 2021.[339] Since the 2020 MRIO table and CO_2 emission inventory in EXIOBASE is an extrapolation from historical data, it does not reflect the reduced economic output and CO_2 emissions occurred in 2020 due to the Covid-19 pandemic. Hence adjustments are made accordingly to both the world MRIO table and CO_2 emission inventory in EXIOBASE. Specifically, change ratio of countries' GDPs[340] from 2019 to 2020 are used to adjust for the domestic intermediate and final consumptions for all countries in the global MRIO table. Change ratio of countries' exports[341] from 2019 to 2020 are used to adjust for the intermediate and final exported consumptions for all countries in the global MRIO table. 2020 global CO_2 emission data from the Global Carbon Project 2021[339] is used to adjust the total CO_2 emissions of countries. Similarly, upon the derivation of production-based $PM_{2.5}$ emission inventory using GAINS model, consumption-based $PM_{2.5}$ emission inventory can be easily obtained using equation (1). As for the 5 rest of the world regions, production-based emissions are disaggregated in proportion to 2015 $PM_{2.5}$ emission inventory of EDGAR database.[342] Consumption-based emission ratio of the 5 rest of the world regions is estimated based on CO_2 emission inventories.

Having consumption-based and production-based inventories for both CO_2 and $PM_{2.5}$ emissions ready, countries are grouped according to HDI levels for results analysis

World Bank population data is used to calculate the per capita CO_2 and $PM_{2.5}$ emissions of different HDI development groups, in accordance with previous calculations, by simply dividing emissions with populations.

Data

1. Multi-region environmentally extended input-output tables: EXIOBASE[338]
2. National total production-based CO_2 emissions: the Global Carbon Project 2021[339]
3. Change ratio of countries' GDPs[340]
4. Change ratio of countries' exports: WTOSTAT, World Trade Organization[341]
5. $PM_{2.5}$ emission inventory: GAINS[228]
6. $PM_{2.5}$ emission inventory: EDGAR database.[342]
7. Population data: World Bank[340]

Caveats

The GAINS model separating $PM_{2.5}$ emissions into three groupings appears necessary for the following reasons. First, a simplification here is done just on the basis of the total fuel use, rather than on the basis of fuel specific data, though this could be further refined in the next version of this mapping tool. Second, process-related emissions are typically related to specific sectors and thus distributing the emissions among the same cluster as the energy-related emissions seems to introduce a smearing out that is not justified. Thus, process emissions from GAINS are distributed not across all MRIO sectors, but only across those that can be clearly identified with a particular process, and those for which a process emission cannot be further resolved. Finally, trucking-related emissions are distributed among all sectors on the basis of their diesel consumption. It is assumed that the relative share of diesel consumption for road transport in each MRIO sector is generally a good proxy for the relative share in the trucking-related emissions.

In the stage of emission inventory disaggregation, simplifications and assumptions may bring uncertainties into the results. When disaggregating the five rest of the world regions, unavailable data are either filled by emissions from previous years or estimated based on the structure of embodied emissions of other pollutants. The analysis can be updated when more accurate emission inventory becomes available in the future.

A number of simplifications have been made that could be refined in the next version of the mapping tool to increase the accuracy of the mapping. The mapping in this exercise is a viable tool to relate process-based calculations to consumption-based accounting frameworks. However, it is understood that the linking of frameworks that were built with different purposes (MRIO as an inventory relating economic inputs to economic outputs; GAINS as an integrated tool for air quality policy decision support based on forward looking scenarios) may result in conceptual anomalies. Furthermore, while numerical results are provided at high sectoral and regional resolution, it is important to keep in mind that at this level the results are more uncertain than at an aggregated level. Further to the mapping process, assumptions and estimations made due to unavailable data points in the inventories will exacerbate uncertainties.

Future form of the indicator

In the future, the present methodology will be refined to reflect additional insights that will arise through the application of the method to different circumstances or updated inventories.

Additional analysis

Figure 87 illustrates the differences in per capita CO_2 and $PM_{2.5}$ emissions of different HDI groups in 2020. The very high HDI country group was the only group that had higher consumption-based production-based emissions of both pollutants, with an exception for CO_2 emissions of low HDI group. It reveals the transfer of pollution burden caused by international trade. The very high HDI countries had the largest per capita CO_2 emissions, while the low HDI countries had the largest per capita $PM_{2.5}$ emissions.

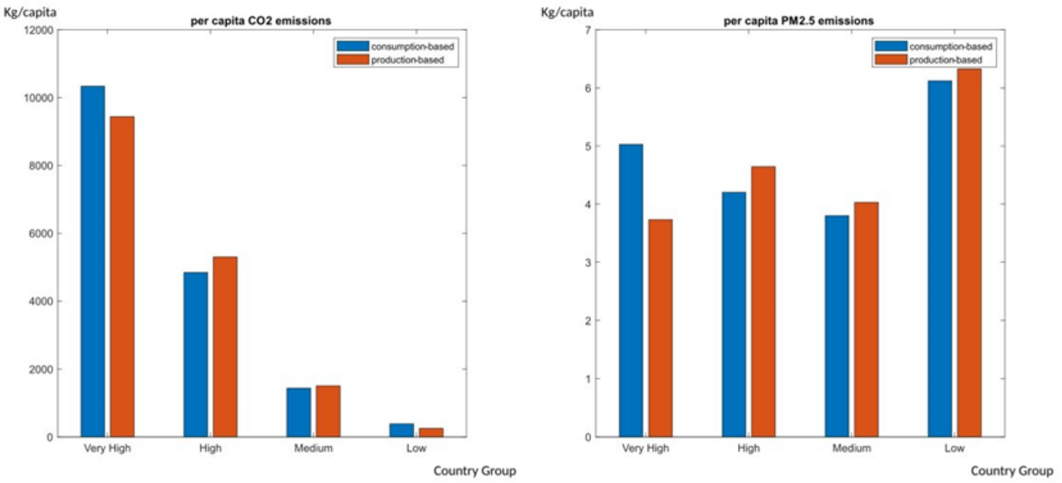

Figure 87: Per capita CO_2 and $PM_{2.5}$ emissions by HDI group

Indicator 4.2.6: Compatibility of Fossil Fuel Company Strategies with the Paris Agreement

Methods

Absolute emission targets

Carbon dioxide (CO_2) is responsible for around three-quarters of total greenhouse gas emissions as measured in gigatonnes of CO_2-equivalent.[343] Climate change has multiple direct impacts on human health, as identified throughout the *Lancet* Countdown. Thus, reducing CO_2 emissions from fossil fuels in order to reduce climate change will bring about significantly improved health outcomes.

This indicator connects CO_2 emissions to the activities of major oil and gas companies that extract these fossil fuels, and analyses the extent to which their future production plans are consistent with the need to reduce CO_2 emissions in order to avoid dangerous climate change. In particular, this indicator focuses on production projections based on actual corporate activities, which may not always reflect declared targets or aspirations. Those companies whose business strategies fall short of what is required can be said to be posing a danger to public health.

The indicator tracks the gap between the projected production of oil and gas companies based on their actual activities, and production trajectories consistent with the Paris target of 1.5°C of warming. The indicator is expressed as a percentage of the projected production of each company is above or below a pathway consistent with the Paris targets. If the indicator value is positive, the company projection is above the climate-consistent plan, and therefore not consistent with the climate target. The indicator analyses both international, publicly traded oil companies (IOCs) and national oil companies (NOCs), which in many cases have larger production volumes than IOCs but are subject to less public or shareholder scrutiny.

A number of organisations analyse the activities of oil and gas companies relative to climate targets, many of them aimed at investors. The Transition Pathways Initiative (TPI) publishes an annual assessment of around sixty large publicly owned O&G companies. However their data is based on companies' own disclosures and reports, so may be more aspirational rather than based on actual production projections, and excludes some of the large state-owned NOCs.[344] The Science-Based Targets Initiative (SBTi) helps businesses set science-based emissions reduction targets, but these are based on companies' own submissions rather than objective assessments of actual activity.[345] The annual Production Gap Report (PGR) tracks the discrepancy between fossil fuel production and climate-consistent production levels, but focuses on 15 countries rather than O&G companies, and relies on government production projections from national energy outlooks and targets.[346] Climate Action 100+ produce an annual Net Zero Company Benchmark;[347] their indicators primarily focuses on ambition, governance and disclosure, but a capital allocation alignment indicator generated by Carbon Tracker

Initiative (CTI) evaluates the alignment of company actions with the Paris Agreement. CTI also publish an annual Two Degrees of Separation report determining potential transition risk exposure for upstream oil and gas companies, finding that the asset stranding risk of unsanctioned (pre-FID) assets is severe.[348] In 2020 Oil Change International (OCI) published production projections for eight IOCs, but this was a one-off report restricted to a small number of companies.[349] A recent journal paper found a discrepancy between the discourse and actions of four oil majors, but focussed on historic actions only.[350]

It is best practice in assessing corporate strategies to consider both absolute and intensity emission targets.[351-353] This prevents a situation where a company scores favourably by reducing its absolute emissions, but merely because it's production is decreasing while its emission intensity (kgCO_2/MJ) could actually be increasing. Or where a company improves its emission intensity but releases more emissions overall because its production is growing. However, an emissions intensity target is not considered here due to the challenges in projecting improvements in operational efficiency (*e.g.*, reduced flaring or leakage) or transitions to lower-carbon fuels such as renewables or nuclear. Instead this indicator uses absolute reduction targets, which are the most meaningful for reducing global total atmospheric emissions.[351]

Emission Benchmarks

An internationally-recognised standard for reporting emissions used by many companies is the GHG Protocol Corporate Standard, developed in 2004 by the World Resources Institute (WRI) and World Business Council for Sustainable Development (WBCSD).[354,355] This divides emissions into Scope 1 (direct emissions), Scope 2 (indirect energy emissions) and Scope 3 (other indirect emissions). Scope 3 emissions (and Use of Sold Products in particular) can be much higher than Scope 1 and 2 emissions (*e.g.* 86% of total emissions of European companies recently reported to the Carbon Disclosure Project, CDP), particularly for energy companies.[356,357]

Corporate benchmarks for companies operating in different sectors can be established using the Sectoral Decarbonisation Approach (SDA), developed in 2015 by the CDP, WRI, and WWF[352] and used by organisations including TPI and SBTi.[351,358] However the SDA is primarily designed for Scope 1 and 2 emissions, and most companies focus their efforts on scopes 1 and 2 emissions over which they have more direct control.[353] Also, the SDA is intended primarily to help companies in homogenous energy intensive sectors (sectors that can be described with a single physical indicator) rather than the oil and gas sector.[351] Indeed SBTi regards science-based emission reductions for fossil fuel companies as complex, and has paused the validation of targets from this sector while it develops a methodology for companies in the oil and gas sector which should be available later in 2022.[359] TPI have generated a sectoral decarbonisation pathway for the O&G sector, but this is an emissions intensity pathway which is not being considered for this indicator.[358]

Paris-compliant least-cost pathways typically generate projections for future global oil and gas production. This oil and gas has to be produced by O&G companies, so this production data can be used to represent the pathway O&G companies must take to be Paris-compliant. In addition, this production data (as opposed to consumption) can be assumed to cover Scope 1, 2 and 3 emissions. Hence this indicator uses oil and gas production data emanating from Paris-compliant modelling to generate Paris-compliant benchmarks for the O&G industry, rather than an SDA approach that generates separate Scope 1, 2 and 3 emission targets.

O&G sector benchmarks are typically derived using climate-compliant pathways from either the IEA or the IPCC. SBTi selected 20 1.5°C scenarios from an ensemble of over 400 peer-reviewed IPCC pathways for their analysis.[360-362] Likewise the Production Gap Report is based on a grouping of 19 IPCC 1.5°C.[346] Alternatively TPI, Carbon Tracker and OCI used IEA pathways; [348,349,358] some considered both. IEA scenarios provide a greater amount of sectoral granularity,[361] and this indicator (in alignment with TPI) uses the IEA's Net Zero Emissions by 2050 (NZE) scenario for its 1.5°C scenario from the IEA's World Energy Outlook 2021 report.[363] The NZE limits global temperature rise to 1.5°C without a temperature overshoot with a 50% probability. Unlike many IPCC 1.5°C scenarios, the NZE reaches net zero emissions in 2050 in the energy and industrial process sectors, and has a lower reliance on uncertain technologies such as CCUS (carbon capture, utilisation and storage), CDR (carbon direct removal) and bioenergy.[364] Oil and gas supply decline markedly by 2050 in this scenario but does not reach zero, indicating a significant reliance on offsetting technologies in other sectors.

Emission Projections

A company's future oil and gas production (and hence emissions) can be estimated from changes in its reserves and investments as recorded in their company reports. However this is a challenging and complex process, for example with different definitions of reserves in different regions, reserves being bought and sold according to market conditions, and an observed recent sector-wide reduction in reserve holdings.[365] Hence a number of analyses base their future production projections on data from Rystad Energy,[347-349] an independent oil and gas consultancy that maintains a database of every oil and gas project in the world.[366] Historical and projected production data were downloaded for this indicator from the Rystad Energy UCube database on 24th February 2022 *i.e*. before the 2022 crisis in Ukraine had an effect on O&G firm strategies.

Each company needs its own benchmark pathway against which its projected production is assessed. This is generated by assigning each company its own market share, based on its average market share over the historical period 2015-2019, relative to actual oil and gas production data from IEA's Key World Energy Statistics.[367] The company is then allocated this fraction of the oil and gas production trajectory contained in the IEA scenarios. Typically this market share is assumed to be constant over time,[352,358] though uncertainties over changing market shares may limit targets to, for example, 15 years ahead.[352] Rystad data for the period 2015-19 indicated that some firms noticeably changed market share, but many remained at similar levels. Rystad projections for the future indicated that projected productions for many firms relative to each other remained relatively stable, suggesting that many firms can be expected to rebound from short-term volatility in production. Nevertheless, this assumption about constant market share can be expected to generate misleading comparisons for some firms, particularly smaller ones, and the effect of this can be reduced by assessing firms in groups.

Here, companies are grouped into publicly listed International Oil Companies (IOCs), including the widely-known Oil Majors, and state-owned National Oil Companies (NOCs) that in many cases have higher production levels but lower scrutiny than IOCs. The eight largest IOCs and seven largest NOCs by production volume in 2021 have been included in this indicator, together accounting for 42% of total global production.

Data

1. 1.5°C pathways from IEA WEO 2021.[368]
2. Oil and Gas firm production projection data from Rystad Energy[366]
3. Historical oil and gas production data from IEA Key World Energy Statistics.[367]

Caveats

There are several caveats to consider with this indicator.

The IEA benchmarks used in this analysis only have 50% probability of maintaining temperatures below the 1.5°C target. Although typical for this sort of analysis, it needs to be remembered that, even if O&G firms follow the Paris-compliant pathways outlined here, there is still a substantial change that temperature targets will be exceeded.

This indicator uses projections of future production of O&G firms from the Rystad Energy database. Although a leading database in the sector, there is a significant possibility that O&G firms will follow different projection pathways to the ones projected by Rystad. These uncertainties are likely to increase over time, meaning projections in the long-term are less certain than in the shorter-term.

O&G firms are assumed here to have constant market shares. This assumption is typical for this sort of analysis but can be expected to introduce errors for at least some firms that increase over time. This can be at least partly addressed by aggregating firms into groupings such as IOCs and NOCs.

Future form of the indicator

In upcoming years, this indicator will monitor the extent to which oil and gas company strategies are compliant with the goals of the Paris Agreements, as production strategies change.

Section 5: Public and Political Engagement

5.1. Media Engagement in Health and Climate Change

5.1.1. Global Coverage of Health and Climate Change

Methods

Intersecting trends in coverage of climate change and health were identified in 66 newspaper sources from January 2007 through December 2021. The 66 sources are located across 36 countries, in four languages, and spanning the six World Health Organization (WHO) regions: African Region, Region of the Americas, South-East Asia Region, European Region, Eastern Mediterranean Region, and Western Pacific Region. These sources were monitored through Nexis Uni, Proquest and Factiva databases accessed via the University of Colorado and University of York libraries.

The 2022 report of the *Lancet* Countdown adopts the search strategy developed for the 2020 and 2021 *Lancet* Countdown reports within these three databases. The search strategy was revised for the 2020 report to increase the precision of the indicator; that is, to reduce the number of ‘false positives’, while retaining the maximum number of ‘true positives’. This was done by retaining those terms that a) produced relevant data, and b) had a low degree of polysemy (i.e. words that have fewer meanings *or* words used in fewer disciplines/domains). Testing for interaction *between* terms also enabled fewer terms to be used (for example, it was found that the term ‘morbidity’ would usually pull in the term ‘mortality’, when related to humans).

The terms were translated once the strategy had been finalised with certain terms presenting difficulties in translation. The English terms ‘hay-fever’ and ‘West Nile’, for example, correlated with more than one term in Spanish and Portuguese and the decision was made to include all relevant terms in the respective search strategies.

For the final strategy, search functions were compared across databases to ensure consistency, as different databases utilise different search filter operators. The searches were conducted with the following key words in English, Spanish, Portuguese and German respectively:

English: (climate change OR global warming) AND (health OR illness OR epidemiolog* OR malnutrition OR morbidity OR fatalit* OR diarrh* OR malaria OR chikungunya OR west nile OR dengue OR hay-fever OR zika)

German: (Klimawandel OR Globale Erwärmung) AND (Gesundheit OR Krankheit OR Epidemiolog* OR Mangelernährung OR Morbidität OR Sterblich* OR Durchfall* OR Malaria OR Chikungunya OR West-Nil-Virus OR Dengue-Fieber OR Heuschnupfen OR Zika)

Portuguese: (mudanças climáticas OR aquecimento global) AND (saúde OR doença OR epidemiologi* OR desnutrição OR morbilidade OR fatalidade* OR diarr* OR malária OR chikungunya OR nilo do oeste OR vírus do nilo OR dengue OR febre dos fenos OR rinite alérgica OR zika)

Spanish: (cambio climático OR calentamiento global) AND (salud OR enfermedad* OR epidemiología OR epidemiólog* OR desnutrición OR malnutrición OR morbosidad OR muert* OR diarrea* OR malaria OR paludismo OR chikungunya OR nilo del oeste OR nilo occidental OR virus del nilo OR dengue OR fiebre del heno OR rinitis alérgica OR zika)

The signal of the search strategies above was found to be strong enough (over 80% relevance in a systematically randomised sample of 500) to allow a more parsimonious approach to this indicator, requiring no screening of articles during the extraction of the data.

Two separate searches were also undertaken with the inclusion of adaptation (“adapt*” OR “resilien*”) and pandemic (“pandemic”) terms. Only the searches in English sources (in Nexis Uni and Factiva) were undertaken. These additional searches covered 2021 only.

Results were obtained from the databases by entering the relevant search strategy along with the relevant date. Counting occurred month by month and the number of returns for each source was recorded on a Microsoft Excel spreadsheet. Primary counting took place for each source along with a secondary independent count of a systematically randomised 20% sample by another researcher. Tertiary counts were undertaken where any mismatch occurred between primary and secondary counts. All counts were agreed by the whole research team.

Using the Excel spreadsheet constructed through the phases of counting, the data was organised in numerous ways for a better understanding of the patterns in coverage. These included by WHO region, by the most recent (2020) Human Development Index categories, and by individual source. The average scores for each month (and aggregated into annual averages) were used as an adjustment for the number of sources selected per region or index category.

Data

1. Three databases were used for the core health and climate change search strategy: Nexis Uni; Proquest; and Factiva databases accessed via the University of Colorado libraries. The 66 newspaper sources are located across 36 countries, in four languages, and spanning the six World Health Organization (WHO) regions.

2. Two databases were used for the health, climate change, and adaptation, and the health, climate change, and pandemic search strategies: Nexis Uni; and Factiva databases accessed via the University of Colorado libraries. The 51 newspaper sources are located across 24 countries and span the six World Health Organization (WHO) regions.

Caveats

In developing the search strategy for the 2020 and 2021 *Lancet* Countdown reports it was found that a significant portion of articles may mention both climate change and health but do not engage with them as integrated issues. Including this coverage remains important as it brings both sets of issues – health and climate change – onto the public agenda and into public awareness.

Future form of the indicator

The 2023 report will look to diversify its sources to integrate more from countries in the low and medium HDI groups.

Additional analysis

Total media engagement with health and climate change across all sources (2007–2021)

Figure 88 shows the total co-coverage of health and climate change across all sources between 2007 and 2021. For the fourth year in a row, 2020–2021 shows an increase in co-coverage of health and climate change over the previous year. This increase is larger than that of the previous year with a +27% percentage change. Since 2015, the year of the Paris Agreement, a 96% increase can be observed in health and climate change coverage, despite a three-year plateau up to 2018.

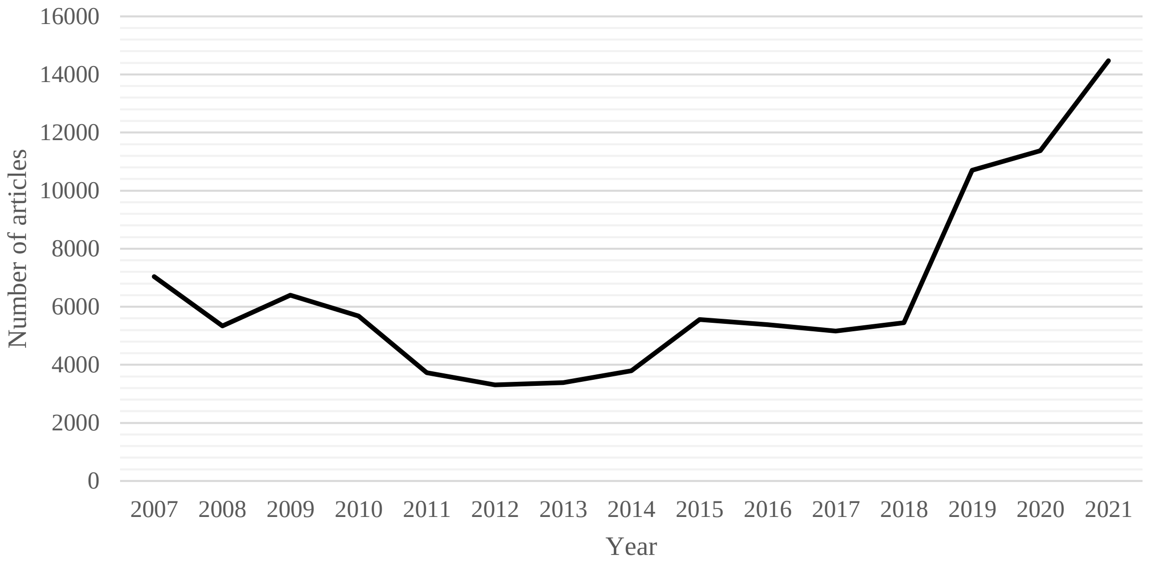

Figure 88: Total media engagement measured by number of articles with health and climate change co-coverage, across all sources (2007–2021)

Percentage change in climate change and health and climate change coverage

Where coverage of health and climate change had a higher percentage change than climate change from 2019 to 2020, the reverse is true from 2020 to 2021: a 54% increase in climate change coverage compared to a 20% increase in health and climate change. The increase in coverage of climate change from 2020 to 2021 is the second highest in the period from 2007 to 2021, after 2018 to 2019; Figure 89).

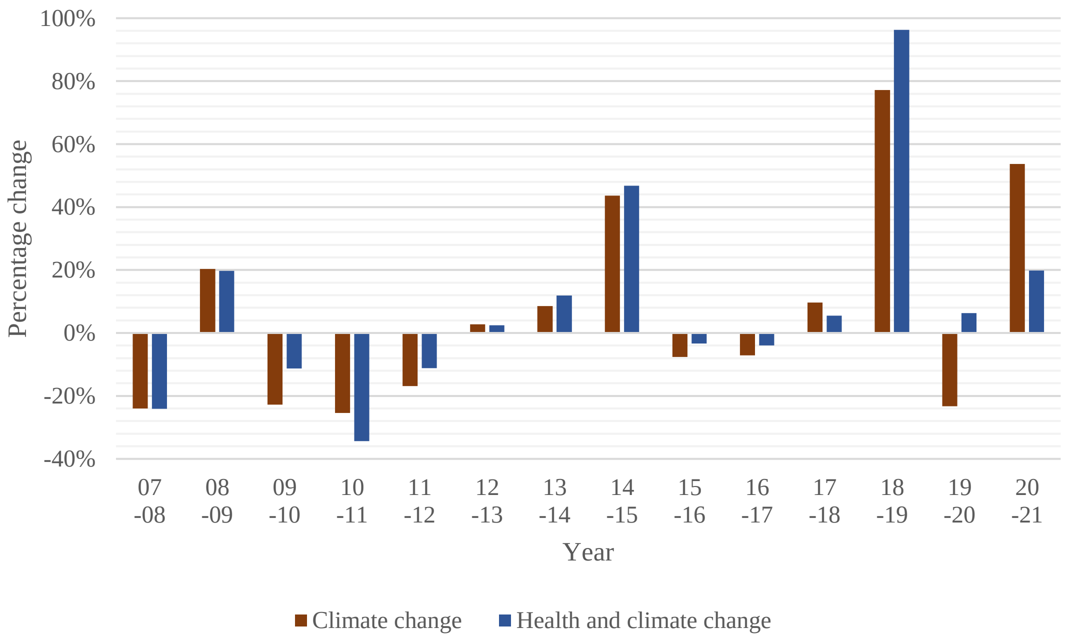

Figure 89: Annual percentage change in coverage of climate change and health and climate change from 2007 to 2021

Total media engagement across all sources in 2020

Figure 90 shows the total number of articles containing both a health and climate change key word in 2021. A notable increase in co-coverage can be observed from August (1040) to October (1580) in the build-up to the

26th Conference of the Parties (COP) in Glasgow. Into November, however, it begins to drop with a large trough in December (899).

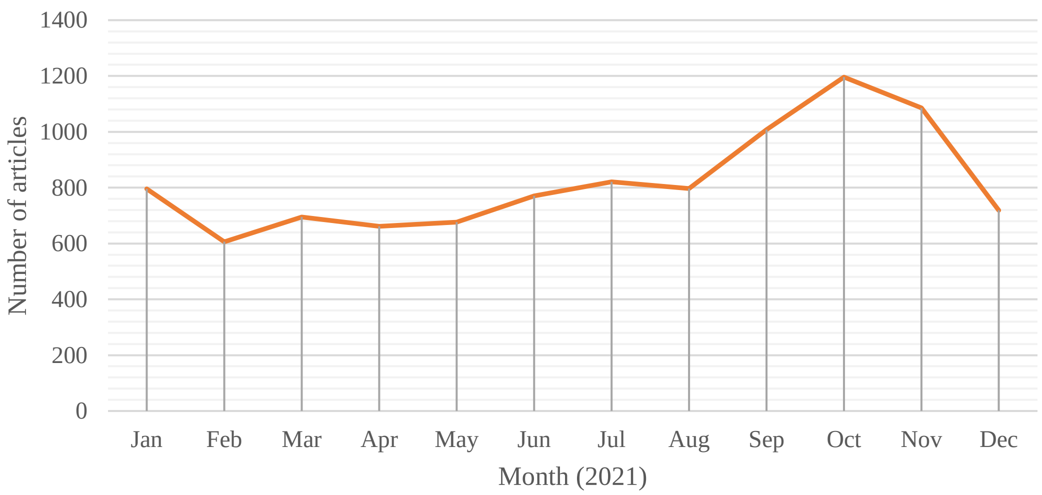

Figure 90: Total media engagement measured by number of articles with health and climate change co-coverage, across all sources in 2021

Geographical distribution of newspaper coverage

Figure 91 shows the average number of articles per year with co-coverage of health and climate change by WHO region. The European region has the highest co-coverage in 2021 with an average of 1713 per source, followed by the Americas with 1137 per source. The Eastern Mediterranean (244) and the African (262) regions have the lowest co-coverage.

Figure 91 also shows that all regions have increasing co-coverage. The African region has the highest percentage change from 2020 to 2021 (+47%). All the other regions have between 23% and 29% percentage increase, except for the Eastern Mediterranean which has only an 8% increase from 2020 to 2021.

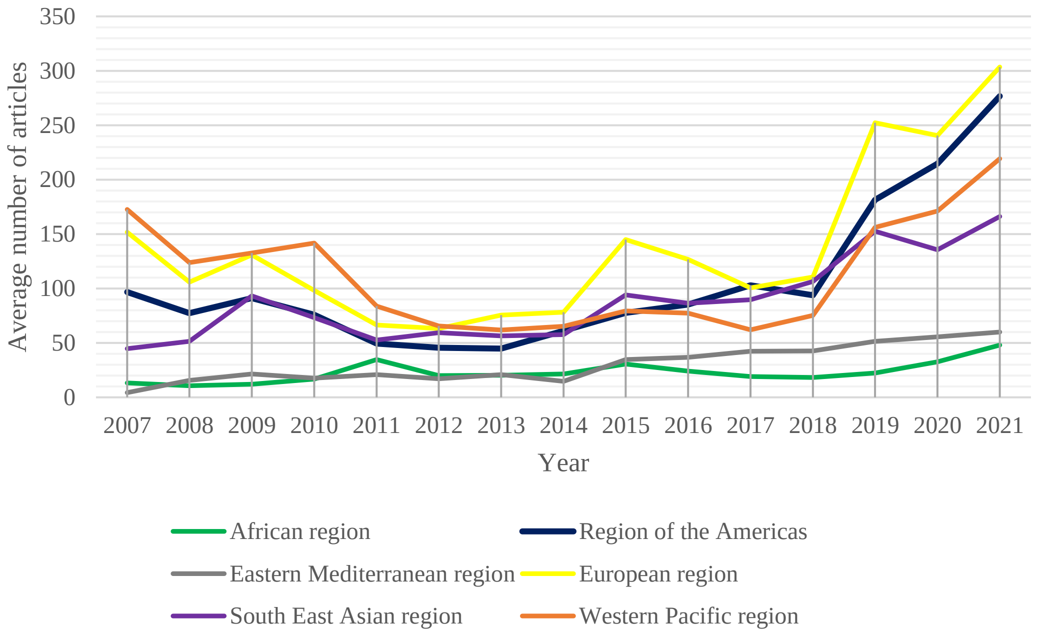

Figure 91: Average annual media engagement by WHO region from 2007 to 2021.

Distribution of newspaper coverage by Human Development Index

Figure 92 presents the average number of articles per year containing health and climate change key words by HDI (2020) classification group (no sources in the low HDI group were used). Co-coverage across all three HDI groups increased from 2020 into 2021 with each reaching its highest average in the period from 2007 to 2021.

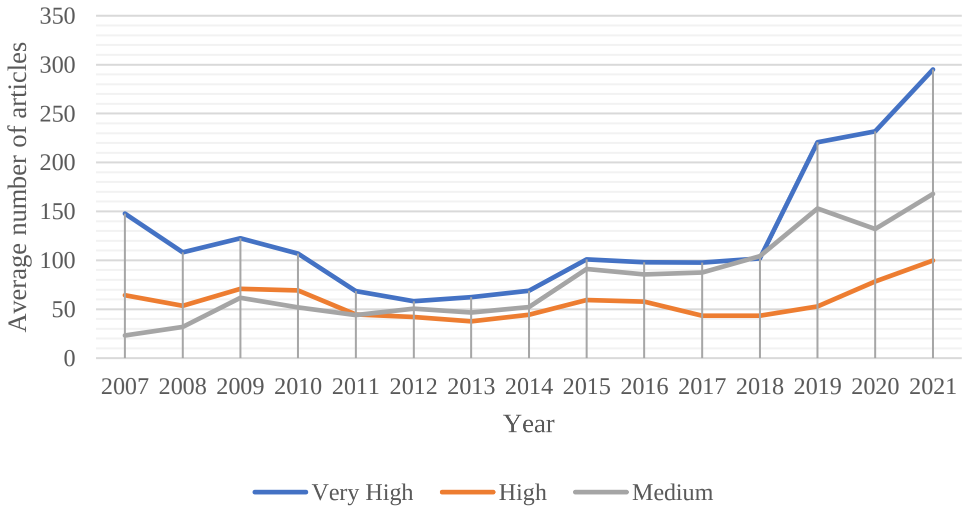

Figure 92: Average annual media engagement by HDI classification group from 2007 to 2021.

Figure 93 shows the average media engagement with health and climate change across 2021 by HDI group. Sources from countries in the very high and medium groups peaked leading up to COP26 in October, whereas those from countries in the high group peak in November, the month of COP. The average co-coverage is consistently higher across sources from countries in the very high group, followed by the medium group.

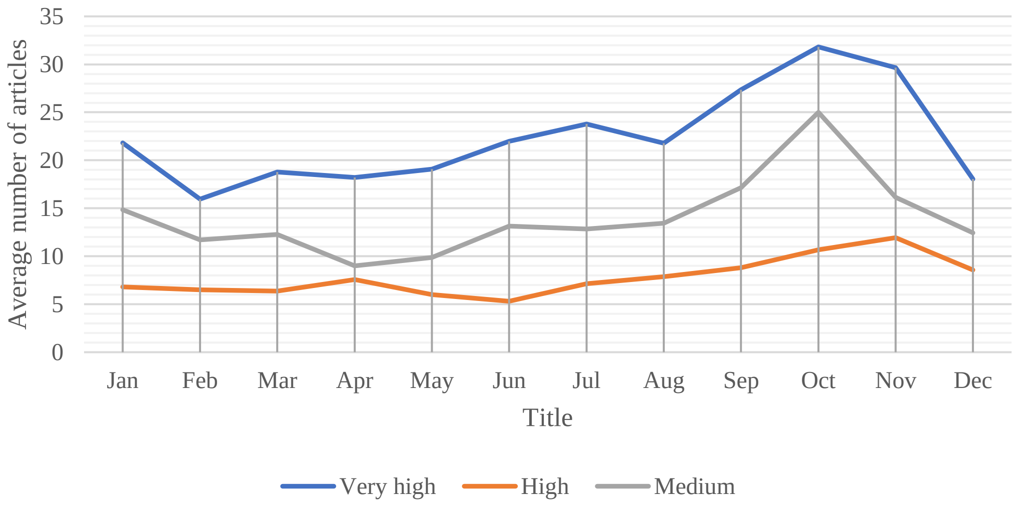

Figure 93: Average media engagement by HDI classification group in 2021.

Figure 94 shows the monthly co-coverage of health and climate change, across 2021, as well as with adaptation keywords and with pandemic keywords. It also shows the number of articles each month where these all combine. Figure 95 shows the monthly proportion of each of these. 63% of articles with co-coverage of health and climate change also mentioned a pandemic keyword, as high as 78% in January. Just over a quarter (26%) of articles with co-coverage of health and climate change also mentioned an adaptation keyword, though this is as high as just under a third (31%) in both April and July.

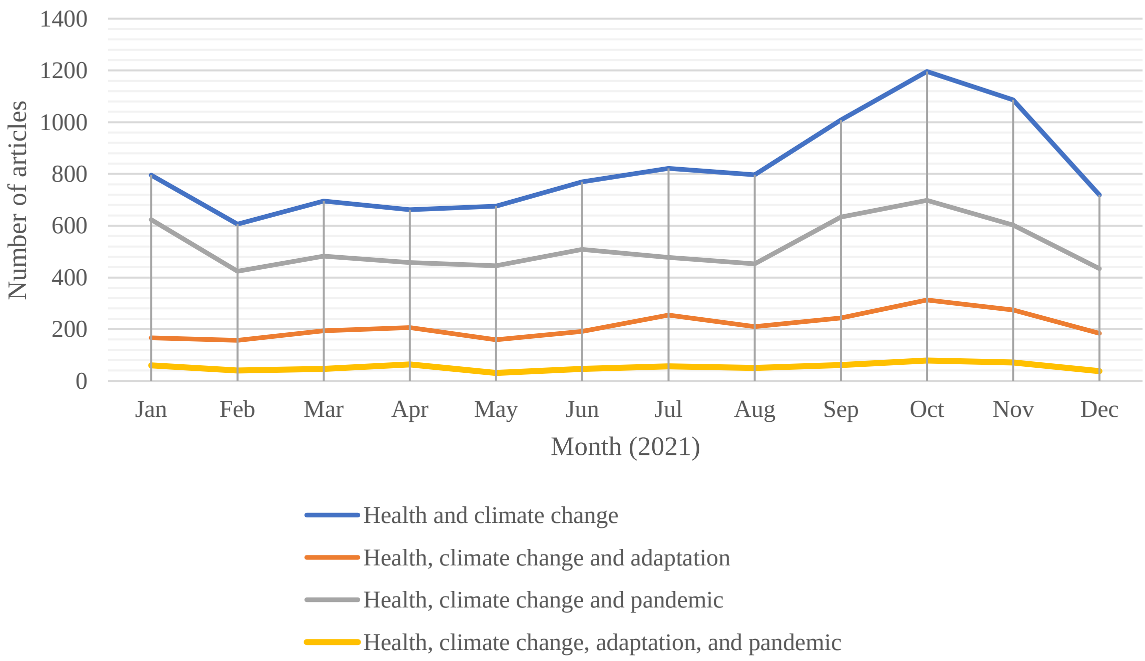

Figure 94: Comparison of 2021 results for four searches: 1) health and climate change, 2) health, climate change, and adaptation, 3) health, climate change, and pandemic, and 4) health, climate change, adaptation, and pandemic.

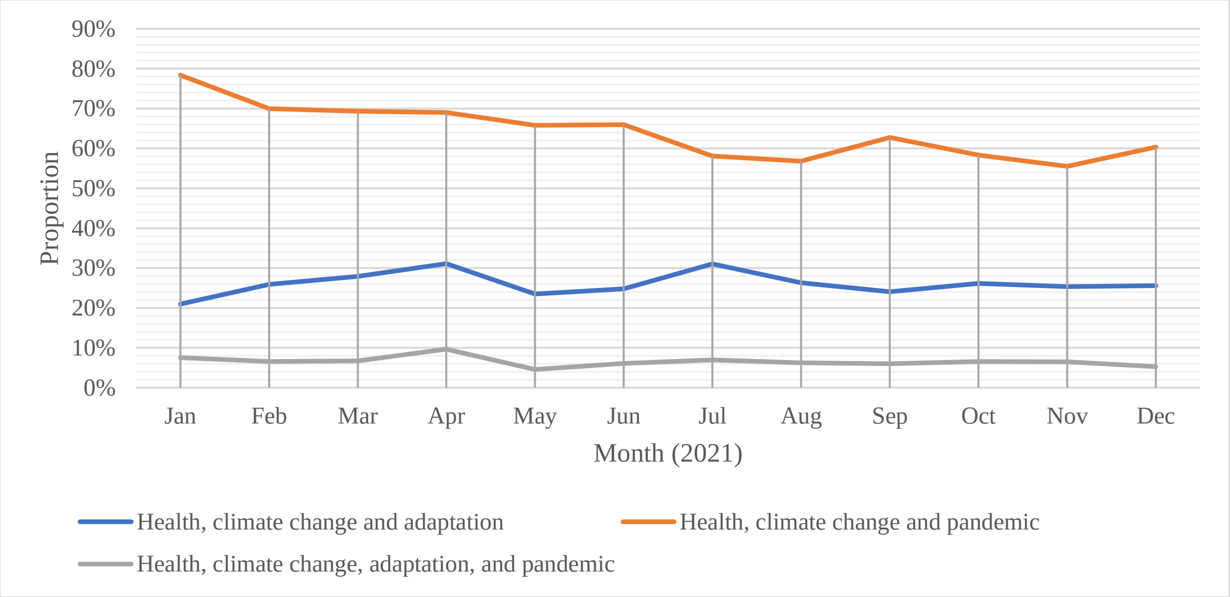

Figure 95: Proportion of health and climate change co-coverage also covering 1) adaptation key words, 2) pandemic keywords, and 3) both adaptation and pandemic keywords, over 2021.

5.1.2. Media Coverage of Health and Climate Change in China's People's Daily

Method

The methodology of the 2021 *Lancet* Countdown report was used here: trawling all articles and then searching for keywords within the text using a filtering process by score and keywords ratio (step 4 of method below).

In addition, this analysis explored the number of health and climate change articles also related to adaptation and the number of articles also related to pandemic preparedness between 2008–2021.

Step 1: trawling all 2021 articles

All articles that were published in "People's Daily" in 2021 were trawled (http://paper.people.com.cn/rmrb/html/2022-03/05/nbs.D110000renmrb_01.htm)

Step 2: Searching for "Climate Change" topic articles

The articles were searched for climate change keywords (Table 64, column 1). Eight new keywords, relating to extreme weather, have been added since the previous *Lancet* Countdown report (Table 64, red). To ensure comparability with previous years' results, these new terms were searched for retrospectively (Figure 96).

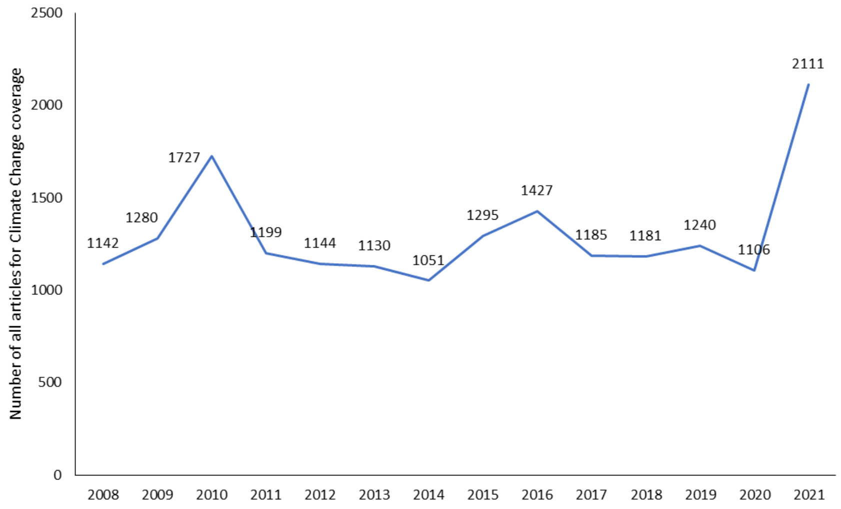

Figure 96: Number of articles identified in People's Daily by searching for climate change keywords.

Step 3: identification of articles with both climate change and health keywords (first-round search)

First round filtering aimed to identify articles that have both climate change and health keywords (see Table 64 for all keywords and Table 65 for English translations). The results are the basis for the second-round search in step 4.

Step 4: machine filtering of results from step 3 by score and ratio (second-round search)

The articles obtained from step 3 were first scored based on the frequency of appearance of the keywords shown in the articles. For example, if the keywords of climate change and health appeared 12 times in one article, then the **score** for this article is 12. If the keyword found is one of the "mis-hit words" (a "mis-hit words" is defined as the phrase that contains a keyword but with different meaning), the appearance will not be counted as one score. Lists of "mis-hit words" can be found in the fourth column of Table 64.

The **ratio** of times of appearance of the keywords to the total number of characters in the article (short for "the ratio" thereafter) was also calculated. When the score and the ratio of one article are both higher than the manually-set thresholds, the article was considered as a relevant article for health and climate change. Via this step, the numbers of relevant articles are illustrated by the grey line in Figure 97.

The threshold of score for each article is set to be 10, meaning the times of appearance of the keywords from both climate change and health in one article should be no less than 10. The threshold of ratio for each article is set to be no less than 1%, meaning in every 100 characters in the article, there should be no less than one keyword.

If the two thresholds were set too low, it would increase the workload of manual screening and increase the "false rate" of machine filtering. If the two thresholds were too high, it might exclude "true" articles. The thresholds for score and ratio were therefore set at 10% and 1% respectively.

Step 5: manual screening of the results after machine filtration

The fifth step involved manually screening the filtered articles. Health and climate change 'true' articles were retained (Figure 97, orange line).

Figure 97 shows the difference before and after screening: before there were 139 (the highest over the period covered), but only ten articles were retained which is lower than the average for the period.

These results indicate a large number of false positive articles in 2021. A manual check of the false positives demonstrates that this is due a large number of articles where public health and climate change are two separate topics mentioned in the speech of national leaders. Titles of the ten positive articles are presented in **Error! Reference source not found.**.

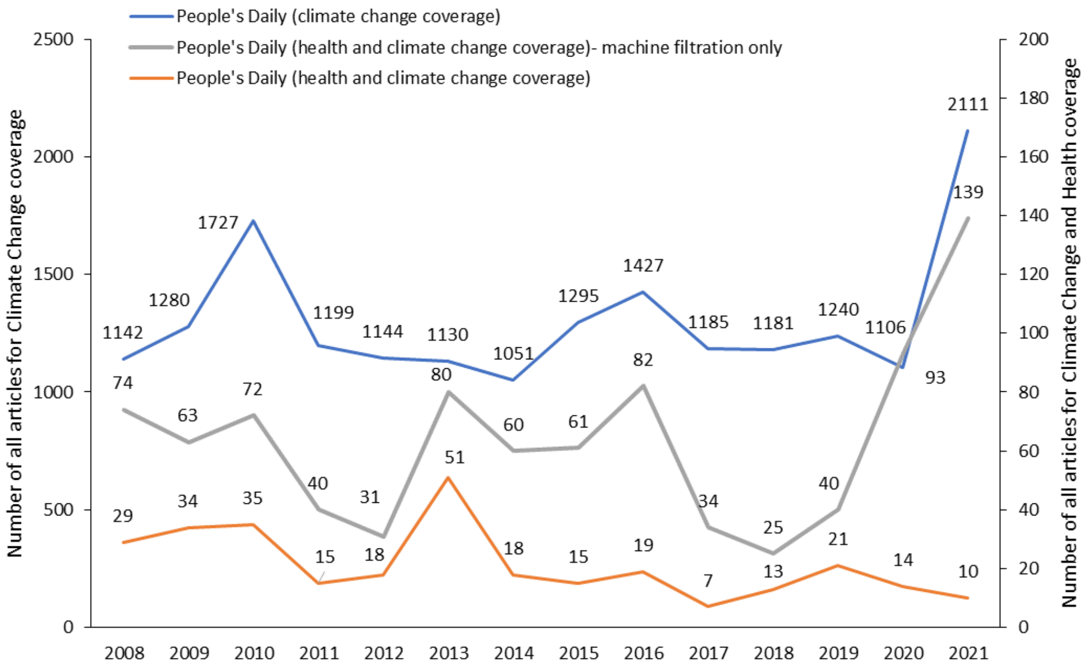

Figure 97: Numbers of all articles for climate change only (blue line), health and climate change after machine filtering only (grey line), and health and climate change after machine filtering and manual screening (orange line).

Step 6: searching for climate change adaptation topic articles

This step identified articles with at least one climate change adaptation keyword as well as keywords for health and climate change. Climate change adaptation keywords are presented in Table 66. Six articles contain climate change adaptation keywords. Titles of these six articles are presented in **Error! Reference source not found.**.

Step 7: manual screening of the results of climate change adaptation topic after machine filtration

Articles retained through the filtering stage were manually screened. Five of the six articles were true positive articles. The only false positive article was due to the ambiguous meaning of the keyword “resilience”.

气候变化关键词	气候变化二级关键词	健康关键词	剔除词
**气候变化**	霾	疟疾	口蹄疫
**全球变暖**	空气污染	腹泻	黑烂病
**温室**	大气污染	感染	珊瑚死亡
**极端天气**		肺炎	沙虫死亡
**全球环境变化**		流行病	高温加热
**低碳**		公共卫生	低碳水
**可再生能源**		卫生	健康发展
**碳排放**		发病	生态健康
**二氧化碳排放**		营养	河流健康
**气候污染**		精神障碍	生态环境健康

**气候**		发育	
**全球升温**		传染	
**再生能源**		疾患	
**CO2排放**		症	
**污染**		瘟疫	
**极端气候**		流感	
**高温**		流行感冒	
**变暖**		治疗	
**排放**		保健	
**环境变化**		健康	
**升温**		死亡	
**全球温升**		精神疾病	
**热浪**		精神病	
**暴雨**		登革热	
**气温**		饥饿	
**洪水**		粮食	
**洪灾**		有害	
**气候反常**		皮肤病	
**野火**		风湿	
**山火**		呼吸系统疾病	
**雪灾**		人类健康	
**低温**		人体健康	
**年代际**		身体健康	
**冰雪**		心脏病	
**可持续发展**		糖尿病	
**海洋酸化**		疾病	
**静稳**		热死	
**温室气体**		口罩	
**寒潮**		防护	
**强降雪**			
**暴雪**			
**台风**			
**干旱**			
**水灾**			

极端降雨			
冻害			

Table 64: Chinese keywords for the search in People's Daily

Keywords of "Climate Change"	Sub- level keywords of "Climate Change"	Keywords of "Health"	Removal words
**Climate change**	Haze	Malaria	Aftosa
**Global worming**	Air pollution	Diarrhea	Black shank
**Greenhouse**	Atmospheric Pollution	Infected	Coral death
**Extreme weather**		Pneumonia	Sandworm death
**Global environment change**		Epidemic	Heating to higher temperature
**Low carbon**		Public health	Low carbohydrate
**Carbon dioxide emissions**		Hygiene	Healthy development
**Renewable energy**		Disease outbreak	Ecological health
**Carbon Production**		Nutrition	River health
**Air pollution**		Mental disorders	Eco-environmental health
**Climate**		Growth	
**Global worming**		Infection	
**Renewable energy**		Affection	
**CO_2 emissions**		Symptom	
**~~Pollution~~**		Epidemic	
**Extreme weather**		Flu	
**High temperature**		Influenza	
**Warming**		Treatment	
**Emission**		Health care	
**Environmental change**		Health	
**Warming**		Death	
**Global warming**		Mental disease	
**Heat wave**		Mental illness	
**Rainstorm**		Dengue	

**Temperature**		Hunger	
**Flood**		Food	
**Flood**		Harmful	
**Abnormal weather**		Skin disease	
**Wildfire**		Rheumatism	
**Mountain fire**		Respiratory diseases	
**Snowstorm**		Human health	
**Low temperature**		Body health	
**Interdecadal**		Heart disease	
**Ice and snow**		Diabetes	
**Sustainable development**		Illnesses	
**Ocean acidification**		Heat death	
**Stagnant**		Mask	
**Greenhouse gas**		Protection	
**Cold wave**		Survive	
**Heavy snowfall**			
**Blizzard**			
**Typhoon**			
**Drought**			
**Flood**			
**Extreme rainfall**			
**Frost damage**			

Table 65: English translation of the Chinese keywords

适应/韧性	Adaptation/resilience
疫情应对	Pandemic preparedness

Table 66: Adaptation and pandemic preparedness keywords for the search in People’s Daily

Data

All the articles from 2008 to the present published on People’s Daily, taken from the official website of People’s Daily.

Additional information

Titles of the articles in 2021

文章名字	Titles of the article

寒潮也由“变暖”而来	Cold wave also comes from "warming”
当悟“人与自然是生命共同体”	Understanding that "people and nature are a community of life"
首届气候适应峰会闭幕	First Climate Adaptation Summit concludes
改革和完善全球治理体系	Reforming and improving the global governance system
加强国际合作，共同应对气候变化	Strengthening international cooperation to address climate change
印尼洪灾已致68人死亡70人失踪	Floods in Indonesia kill 68, leave 70 missing
积极采取行动应对气候变化	Actions to address climate change
地中海沿岸国家加强火灾防控	Fire prevention and control strengthened in Mediterranean coastal countries
共同应对气候变化挑战	Climate change challenges to be tackled together
各国采取气候行动确保疫情后绿色复苏	Countries take climate action to ensure green recovery after outbreak

Table 67: Title of the health and climate change articles in People’s Daily.

文章名字	Title of the article
适应气候变化是现实的选择	Adaptation to climate change is a realistic option
增强适应气候变化能力保障可持续发展	Enhancing climate change adaptation capacity to ensure sustainable development
适应气候变化提高防灾减灾能力	Adaptation to climate change to improve disaster prevention and mitigation capabilities
全面落实国家适应气候变化战略	To settle down National Climate Change Adaptation Strategy
增强城市发展韧性 提高城市应对重大公共卫生事件能力	Enhancing the resilience of cities to respond to major public health events
首届气候适应峰会闭幕	First Climate Adaptation Summit concludes

Table 68: Titles of the articles of climate change adaptation coverage in climate & health articles in People’s Daily.

People’s Daily: http://paper.people.com.cn/rmrb/html/2022-03/05/nbs.D110000renmrb_01.htm

5.2. Individual Engagement in Health and Climate Change

Methods

This indicator provides an individual-level indicator of public engagement. It tracks engagement with climate change and health through people's usage of the online encyclopaedia Wikipedia. Over the years, Wikipedia has grown to be a major and trusted source of information that has outpaced traditional encyclopaedias in terms of reach, coverage, and comprehensiveness.[iv] It is regularly listed among the ten most-visited websites worldwide.[v] The English edition covers more than six million articles and over 130,000 active editors. People around the world use it to engage in topics they are interested in. Fortunately, the traffic that goes to Wikipedia – and even that which goes to individual articles of the encyclopaedia – can be analysed over time because the Wikimedia foundation makes these statistics available to everyone for free. This makes it a global indicator of what people pay attention to on a daily basis. What is more – and of particular relevance in the context of this report – is that the platform's health content makes it one of the most frequently used resources for information on health on the internet.[369]

The indicator

To investigate to what extent people do not only pay attention to climate change and human health in isolation, but also to the connection between both, *clickstream statistics* from the English Wikipedia were drawn upon.

Clickstream refers to a dataset provided by the Wikimedia foundation.[vi] It reports "streams of clicks", or in other words: how people get to a Wikipedia article and what links they click on. This is reported on a monthly basis and in pairs of resources, the first being where the visit came from, the second which page was visited. This gives an indicator of monthly-level global attention towards one issue (if both articles are representative of the same issue) or two issues (if articles come from different domains, such as climate change and health). By looking at climate change – health articles pairs, an indicator of attention towards climate change consequences for human health over time is generated.

Measurement strategy

The approach to using clickstream data as an indicator of public engagement in climate change and health is based on the following premises: (1) The Wikipedia platform is a globally used source for information on a multitude of topics.[vii] (2) Citizens use the platform to inform themselves about topics they are interested in. (3) By tracking engagement with Wikipedia articles that are related to climate change as well as with articles on health, it is possible to identify public engagement with the relationship between both topics.

The following behavioural patterns are relevant for the validity of the measure as a proxy for public engagement with climate change and health:

(a) A person is generally interested in the nexus between climate change and public health and informs her/himself about the topic online by, e.g., reading the Wikipedia article on *Effects of climate change on human health* (https://en.wikipedia.org/wiki/Effects_of_climate_change_on_human_health)

(b) A person is interested in climate change and the consumption of information about the topic then sparks interest in its consequences for human health. For instance, the person reads the article on

---

[iv] Giles, Jim. 2005. "Internet encyclopaedias go head to head." Nature 483(15 December):900–901.

[v] Alexa. 2018. "The top 500 sites on the Web." https://www.alexa.com/topsites.

[vi] See https://meta.wikimedia.org/wiki/Research:Wikipedia_clickstream.

[vii] See https://stats.wikimedia.org/wikimedia/squids/SquidReportPageViewsPerCountryOverview.htm for an overview of Wikipedia usage by country and languages.

Climate change (https://en.wikipedia.org/wiki/Climate_chage) and then turns to the article on *Malnutrition* (https://en.wikipedia.org/wiki/Malnutrition)

(c) A person is interested in a certain aspect of human health or consequences of climate change with an immediate impact on human health, and then turns its attention to climate change issues. For instance, the person reads the article on *Malaria* (https://en.wikipedia.org/wiki/Malaria) and then turns to the article on *Climate change* (https://en.wikipedia.org/wiki/Climate_change)

Indicator construction

In order to use the Wikipedia viewership statistics as a proxy for public engagement with climate change and health, it is key to select articles that are representative of these topics. A semi-automated approach was implemented to generate the populations of articles related to climate change on the one hand and health on the other. Related articles were searched for using the internal Wikipedia search function based on an initial set of keywords.

Keywords

For climate change articles, the keywords were: *ipcc, sphere, climat, sea ice, sea level, co2, glacial, ozone, green new, climate change, changing climate, climate emergency, climate action, climate crisis, climate decay, global warming, green house, extreme temperature, temperature record, extreme weather, global environmental change, climate variability, greenhouse, greenhouse-gas, low carbon, ghge, ghges, renewable energy, carbon emission, carbon emissions, carbon dioxide, carbon-dioxide, co2 emission, co2 emissions, climate pollutant, climate pollutants, decarbonization, decarbonisation, carbon neutral, carbon-neutral, carbon neutrality, climate neutrality, net-zero, net zero.*

For health articles, the seed keywords were: *epidemy, disease, malaria, diarrhoea, infection, sars, measles, pneumonia, epidemic, pandemic, public health, health care, healthcare, epidemiology, mortality, morbidity, nutrition, illness, infectious, ncd, non-communicable disease, noncommunicable disease, communicable disease, air pollution, nutrition, malnutrition, mental disorder, stunting, epidemy, public health, health care, infectious, non-communicable disease, noncommunicable disease, communicable disease, syndrome, diagnosis, psychiatric, epidemiolog, disorder, pediatric, osis, itis, icide, hunger, fever, asthma, cancer.*

Article processing

For each search using one of the keywords, the first 100 results were extracted and identified and that led to an article with a minimum word count of 300, ensuring that the articles that were chosen as seed articles had been given a certain degree of attention by Wikipedia editors, therefore being more likely to link to other relevant articles.

Next, the articles collected were screened via the Wikipedia search for categories, which are used on Wikipedia to categorise pages in a meaningful way (e.g., using categories such as *Climate change* or *Effects of climate change*). Those categories were then themselves screened for relevant articles. All additional articles were once more filtered such that those with a title matching one of the initial keywords was chosen. For the health-related articles, several articles were excluded manually as they turned out to be irrelevant for the purposes of this study. Health topics are covered extensively on Wikipedia, but the priority was articles and topics that, in principle, can be related to climate change. In addition, the Wikipedia page on the effects of climate change on human health[viii] offers a variety of links to further health-related articles. A fact that was exploited to draw up a curated list of relevant health articles that was added to the overall list. The complete list of articles is listed under *Additional Information*.

For the clickstream analysis, the set of articles was extended by also taking "second-level pages" into account, that is pages that are linked to the initially identified set of climate change or health articles and that are also

---

[viii] See https://en.wikipedia.org/wiki/Effects_of_climate_change_on_human_health.

somewhat related to climate change or health. Sometimes, people might not directly jump from one of the major articles on climate change to another one on health, but travel through an intermediary page e.g., a possible individual stream of clicks could be: *Climate change* → *Human impact on the environment* → *Respiratory disease*. The clickstream data only allow the identification of click volume for pairs of articles, but by extending the network, clickstreams involving relevant pages that are linked in the original set of articles can be captured.

Technically, the fact that the population of health articles is far larger than the population of climate change articles does not invalidate the measurement strategy. It seems plausible that there are many more articles on health-related than on climate change-related topics because the health field is so much broader, which is one reason why the health articles cluster in the network plot is not that dense – some health topics are really far apart from each other, although both could be covering health issues that are affected by climate change. But this should not directly affect the metrics. Even if there are many more health than climate change articles, it could still be that health topics are mentioned (and clicked on) much more often in climate change articles than the other way around. What is key in this analysis is not that one or the other topic is more extensively covered on the platform, but the patterns of co-visiting.

Data

Publicly available data from the Wikimedia foundation is drawn upon for this study. Data from all platforms, i.e. accesses to Wikipedia via desktop machines, mobile browsers, and mobile apps is considered.

The clickstream data were downloaded from the Wikimedia Dumps (https://dumps.wikimedia.org/other/clickstream/). Spider traffic (i.e. traffic generated by automated bots crawling the platform) is excluded. Referer-resource pairs (i.e. the pairs of the article of origin and the target article) that had less than 10 clicks were removed in the original dataset. The expectation because of this is that the actual clickstream traffic is underreported. However, it is not expected to add any systematic bias to the indicators, in particular since interest lies mainly in changes of engagement over time.

Clickstream data are available from November 2017 onwards and this report will focus on data from 2018 to 2021. The analyses are limited to the English Wikipedia.

The benefits of the Wikipedia usage metadata for the purpose of tracking public engagement in climate change and health are that these data (a) are globally available, (b) cover the time period of interest, (c) are collectible at virtually no cost, and, perhaps most importantly, (d) have high face validity to measure engagement in this very specific topic. Reading articles on Wikipedia is motivated by attention towards a particular issue. Individuals invest time to inform themselves about a topic, which is one manifestation of engagement. Aggregate reading behaviour can therefore be seen as an *a priori* valid approximation of public issue engagement.

Caveats

All clickstream information is only available at the aggregate level. It is not possible to link the data to information about individuals who visited the platform. Also, the data are not geo-referenced, so it is not possible to infer where page visits came from. Although the English Wikipedia is predominantly used in English-speaking countries, — according to the Wikimedia Traffic Analysis Report;[ix] about 40% of the traffic on the English Wikipedia comes from the United States — it is a globally popular resource. It makes up for 50% of the global traffic to all Wikipedia language editions. Therefore, it can be seen as a global indicator of public attention that is somewhat biased towards attention from countries such as the United States, United Kingdom, India, Canada, and Australia. Extending the analyses to other language editions will help to remedy this bias and uncover potential geographic engagement heterogeneity in the future.

More generally, the measure represents an online proxy for an offline phenomenon. In addition, it is sensitive towards the selection of articles used to capture engagement. The global popularity of the platform, which consistently ranks among the ten most visited websites worldwide, speaks in favour of its usefulness for this application. However, more direct indicators of public engagement, such as survey-based measures, might provide a useful supplement and source for validation in the future.

[ix] See https://stats.wikimedia.org/wikimedia/squids/SquidReportPageViewsPerLanguageBreakdown.htm.

While the data are available for free, access to future data depends on the Wikimedia API. There is no indication of Wikimedia restricting access in the future. Instead, Wikimedia has invested in data quality and making access more robust and convenient.

Additional information

List of English Wikipedia articles used to track public engagement in climate change

1997 United Nations Climate Change Conference, 1998 United Nations Climate Change Conference, 1999 United Nations Climate Change Conference, 2000 United Nations Climate Change Conference, 2001 United Nations Climate Change Conference, 2002 United Nations Climate Change Conference, 2003 United Nations Climate Change Conference, 2004 United Nations Climate Change Conference, 2005 United Nations Climate Change Conference, 2006 United Nations Climate Change Conference, 2007 United Nations Climate Change Conference, 2008 United Nations Climate Change Conference, 2009 United Nations Climate Change Conference, 2010 United Nations Climate Change Conference, 2011 United Nations Climate Change Conference, 2012 United Nations Climate Change Conference, 2013 United Nations Climate Change Conference, 2014 People's Climate March, 2014 UN Climate Summit, 2014 United Nations Climate Change Conference, 2015 United Nations Climate Change Conference, 2016 United Nations Climate Change Conference, 2017 People's Climate March, 2017 United Nations Climate Change Conference, 2018 United Nations Climate Change Conference, 2019 in climate change, 2019 UN Climate Action Summit, 2019 United Nations Climate Change Conference, 2020 in climate change, 2021 in climate change, 2021 Leaders Summit on Climate, 2021 United Nations Climate Change Conference, 4 Degrees and Beyond International Climate Conference, A Green New Deal, Abrupt climate change, Academy of Climate Change Education and Research, Action for Climate Empowerment, Advisory Group on Greenhouse Gases, American Association of State Climatologists, American College & University Presidents' Climate Commitment, Amundsen-Nobile Climate Change Tower, Antarctic sea ice, APEC Climate Center, Arctic Climate Impact Assessment, Arctic sea ice decline, Asia-Pacific Partnership on Clean Development and Climate, Asilomar International Conference on Climate Intervention Technologies, Atmosphere of Earth, Attorney General of Virginia's climate science investigation, Attribution of recent climate change, Australian Greenhouse Office, Australian Renewable Energy Agency, Aventine Renewable Energy, Aviation and climate change, Avoiding Dangerous Climate Change (2005 conference), Bali Declaration by Climate Scientists, Bangladesh Climate Change Resilience Fund, Bangladesh Climate Change Trust, Bay Area Climate Collaborative, Biber-Danube interglacial, Bioclimatology, Bjerknes Centre for Climate Research, Boulder Climate Action Plan, Bristol Youth Strike 4 Climate, Business action on climate change, C40 Cities Climate Leadership Group, California Climate Action Registry, California Climate Credit, California Climate Executive Orders, Camp for Climate Action, Campaign against Climate Change, Canadian Youth Climate Coalition, Carbon dioxide, Carbon dioxide (data page), Carbon dioxide angiography, Carbon dioxide clathrate, Carbon dioxide cleaning, Carbon dioxide flooding, Carbon dioxide generator, Carbon dioxide in Earth's atmosphere, Carbon Dioxide Information Analysis Center, Carbon dioxide reforming, Carbon dioxide removal, Carbon dioxide scrubber, Carbon Neutral Cities Alliance, Carbon neutrality, Carbon Neutrality Coalition, Carbon-dioxide laser, Carbon-neutral fuel, CCS and climate change mitigation, Center for Climate and Energy Solutions, Center for Climate and Life, Center for Climate Systems Research, Center for Negative Carbon Emissions, Center for the Study of Carbon Dioxide and Global Change, Centre for Climate Change Economics and Policy, Centre for International Climate and Environmental Research, Centre for Renewable Energy, Centre for Renewable Energy Systems Technology, Chemosphere (journal), Chesapeake Climate Action Network, Chicago Climate Action Plan, Chicago Climate Exchange, Cities for Climate Protection program, Citizens Convention for Climate, Citizens' Climate Lobby, Civil Society Coalition on Climate Change, Climate, Climate action, Climate Action Network, Climate Action Network Latin America, Climate Action Tracker, Climate Alliance, Climate and Clean Air Coalition to Reduce Short-Lived Climate Pollutants, Climate and Development, Climate and Development Knowledge Network, Climate and Ecological Emergency Bill, Climate and energy, Climate apocalypse, Climate appraisal, Climate as complex networks, Climate Audit, Climate Capitalism, Climate Case Ireland, Climate categories in viticulture, Climate Central, Climate change, Climate Change (Scotland) Act 2009, Climate Change Accountability Act (Bill C-224), Climate change acronyms, Climate Change Act 2008, Climate change adaptation, Climate change adaptation strategies on the German coast, Climate Change Agreement (UK), Climate change and agriculture in the United States, Climate change and birds, Climate change and children, Climate change and cities, Climate change and ecosystems, Climate Change and Emissions Management Amendment Act, Climate change and fisheries, Climate change and gender, Climate change and indigenous peoples, Climate change and infectious

diseases, Climate change and invasive species, Climate change and poverty, Climate Change and Sustainable Energy Act 2006, Climate change and wildfires, Climate change art, Climate Change Authority, Climate Change Capital, Climate Change Commission, Climate Change Committee, Climate Change Denial, Climate Change Denial Disorder, Climate change education, Climate change feedback, Climate change in Afghanistan, Climate change in Alabama, Climate change in Alaska, Climate change in Algeria, Climate change in American Samoa, Climate change in Antarctica, Climate change in Argentina, Climate change in Arizona, Climate change in Arkansas, Climate change in Australia, Climate change in Austria, Climate change in Bangladesh, Climate change in Belgium, Climate change in Brazil, Climate change in California, Climate change in Cambodia, Climate change in Canada, Climate change in China, Climate change in Colorado, Climate change in Connecticut, Climate change in Cyprus, Climate change in Delaware, Climate change in Europe, Climate change in Fiji, Climate change in Finland, Climate change in Florida, Climate change in France, Climate change in Georgia (U.S. state), Climate change in Germany, Climate change in Ghana, Climate change in Greenland, Climate change in Grenada, Climate change in Guam, Climate change in Guatemala, Climate change in Honduras, Climate change in Idaho, Climate change in Illinois, Climate change in India, Climate change in Indiana, Climate change in Indonesia, Climate change in Iowa, Climate change in Iraq, Climate change in Israel, Climate change in Japan, Climate change in Jordan, Climate change in Kansas, Climate change in Kentucky, Climate change in Kenya, Climate change in Kyrgyzstan, Climate change in Liberia, Climate change in Louisiana, Climate change in Luxembourg, Climate change in Maine, Climate change in Malaysia, Climate change in Maryland, Climate change in Massachusetts, Climate change in Mexico, Climate change in Michigan, Climate change in Minnesota, Climate change in Mississippi, Climate change in Missouri, Climate change in Montana, Climate change in Morocco, Climate change in Nebraska, Climate change in Nepal, Climate change in Nevada, Climate change in New Hampshire, Climate change in New Jersey, Climate change in New Mexico, Climate change in New York (state), Climate change in New York City, Climate change in New Zealand, Climate change in Nigeria, Climate change in North Carolina, Climate change in North Dakota, Climate change in North Korea, Climate change in Norway, Climate change in Ohio, Climate change in Oklahoma, Climate change in Oregon, Climate change in Pakistan, Climate change in Pennsylvania, Climate change in popular culture, Climate change in Puerto Rico, Climate change in Rhode Island, Climate change in Russia, Climate change in Saskatchewan, Climate change in Scotland, Climate change in Senegal, Climate change in South Africa, Climate change in South Asia, Climate change in South Carolina, Climate change in South Dakota, Climate change in South Korea, Climate change in Spain, Climate change in Sri Lanka, Climate change in Suriname, Climate change in Sweden, Climate change in Taiwan, Climate change in Tanzania, Climate change in Tennessee, Climate change in the Arctic, Climate change in the Caribbean, Climate change in the Gambia, Climate change in the Middle East and North Africa, Climate change in the Netherlands, Climate change in the Philippines, Climate change in the Republic of Ireland, Climate change in the United Kingdom, Climate change in the United States, Climate change in Turkey, Climate change in Tuvalu, Climate change in Utah, Climate change in Vermont, Climate change in Vietnam, Climate change in Virginia, Climate change in Washington, Climate change in Washington, D.C., Climate change in West Virginia, Climate change in Wisconsin, Climate change in Wyoming, Climate Change Levy, Climate change litigation, Climate change mitigation, Climate change mitigation framework, Climate change mitigation scenarios, Climate Change Performance Index, Climate change policy of California, Climate change policy of the George W. Bush administration, Climate change policy of the United States, Climate Change Response (Emissions Trading) Amendment Act 2008, Climate Change Response (Zero Carbon) Amendment Act, Climate Change Response Act 2002, Climate change scenario, Climate Change Science Program, Climate Change TV, Climate change vulnerability, Climate Change: Global Risks, Challenges and Decisions, Climate classification, Climate commitment, Climate communication, Climate Consortium Denmark, Climate crisis, Climate Data Operators, Climate debt, Climate Denial Crock of the Week, Climate Disclosure Standards Board, Climate Doctrine of the Russian Federation, Climate Dynamics, Climate emergency declaration, Climate emergency declarations in Australia, Climate emergency declarations in the United Kingdom, Climate engineering, Climate ensemble, Climate ethics, Climate fiction, Climate footprint, Climate forcing, Climate governance, Climate Hawks Vote, Climate Hustle, Climate inertia, Climate Investment Funds, Climate justice, Climate Justice Action, Climate Justice Alliance, Climate Justice Now!, Climate Law and Governance Initiative, Climate Leadership Council, Climate Mirror, Climate model, Climate Monitoring and Diagnostics Laboratory, Climate movement, Climate of Agra, Climate of Albania, Climate of ancient Rome, Climate of Argentina, Climate of Armenia, Climate of Australia, Climate of Azerbaijan, Climate of Bangladesh, Climate of Barcelona, Climate of Bihar, Climate of Bilbao, Climate of Brazil, Climate of Budapest, Climate of Buenos Aires, Climate of Cebu, Climate of Chile, Climate of Colombia, Climate of Cyprus, Climate of Delhi, Climate of Dubai, Climate of East Anglia, Climate of Ecuador, Climate of

Egypt, Climate of Estonia, Climate of Finland, Climate of Ghana, Climate of Gibraltar, Climate of Greece, Climate of Gujarat, Climate of Himachal Pradesh, Climate of Hungary, Climate of Iceland, Climate of India, Climate of Indonesia, Climate of Ireland, Climate of Italy, Climate of Kaziranga National Park, Climate of Kolkata, Climate of Kosovo, Climate of Lisbon, Climate of Madrid, Climate of Malta, Climate of Manitoba, Climate of Mexico, Climate of Moscow, Climate of Mumbai, Climate of Myanmar, Climate of New England, Climate of New Zealand, Climate of Nova Scotia, Climate of Pakistan, Climate of Paraguay, Climate of Peru, Climate of Porto, Climate of Puerto Rico, Climate of Rajasthan, Climate of Romania, Climate of Rome, Climate of Russia, Climate of Saudi Arabia, Climate of Seoul, Climate of Serbia, Climate of Sochi, Climate of South Africa, Climate of South Brazil, Climate of Southeast Brazil, Climate of Spain, Climate of Svalbard, Climate of Tamil Nadu, Climate of Tasmania, Climate of the British Isles, Climate of the Falkland Islands, Climate of the Philippines, Climate of the United Kingdom, Climate of the United States, Climate of Turkey, Climate of Uruguay, Climate of Uttar Pradesh, Climate of Valencia, Climate of Venezuela, Climate of Vietnam, Climate of West Bengal, Climate of Zambia, Climate One, Climate Policy (journal), Climate prediction, Climate Prediction Center, Climate psychology, Climate Research (journal), Climate resilience, Climate restoration, Climate risk, Climate risk insurance, Climate risk management, Climate Savers Computing Initiative, Climate Science Legal Defense Fund, Climate Science Rapid Response Team, Climate security, Climate sensitivity, Climate Solutions Caucus, Climate Solutions Road Tour, Climate spiral, Climate Stewardship Acts, Climate TRACE, Climate variability and change, Climate Vulnerability Monitor, Climate Vulnerable Forum, Climate Watch, Climate Week NYC, Climate-adaptive building shell, Climate-Alliance Germany, Climate-friendly gardening, Climate-friendly school, Climate-smart agriculture, Climate: Long range Investigation, Mapping, and Prediction, Climatic Change (journal), Climatic geomorphology, Climatic regions of Argentina, Climatic Research Unit documents, Climatic Research Unit email controversy, Climatological normal, Climatology, Cloud formation and climate change, Co-benefits of climate change mitigation, CO2 Coalition, CO2 fertilization effect, CO2 is Green, CO2balance, Committee on Climate Change Science and Technology Integration, Cool It: The Skeptical Environmentalist's Guide to Global Warming, Copenhagen Climate Challenge, Copper in renewable energy, Criticism of the IPCC Fourth Assessment Report, Danube-Gunz interglacial, Debate over China's economic responsibilities for climate change mitigation, Decarbonisation measures in proposed UK electricity market reform, Deep Decarbonization Pathways Project, Deforestation and climate change, Dendroclimatology, Department of the Environment, Climate and Communications, Description of the Medieval Warm Period and Little Ice Age in IPCC reports, Desert climate, Disability and climate change, Drawdown (climate), Earth rainfall climatology, East Asia Climate Partnership, Economic impacts of climate change, Economics of climate change, Economics of climate change mitigation, Economists' Statement on Climate Change, Ed Hawkins (climatologist), Effects of climate change, Effects of climate change on agriculture, Effects of climate change on human health, Effects of climate change on island nations, Effects of climate change on marine mammals, Effects of climate change on oceans, Effects of climate change on plant biodiversity, Effects of climate change on terrestrial animals, Effects of global warming, Effects of global warming on human health, Effects of global warming on humans, Effects of global warming on the United Arab Emirates, Electrochemical reduction of carbon dioxide, Euro-Mediterranean Center on Climate Change, European Assembly for Climate Justice, European Climate Change Programme, European Climate Exchange, European Climate Forum, European Climate Foundation, European Climate, Infrastructure and Environment Executive Agency, European Renewable Energy Council, European Union climate and energy package, Evangelical Climate Initiative, Exposing Microorganisms in the Stratosphere, Extreme weather, ExxonMobil climate change controversy, Federal Ministry for Economic Affairs and Climate Action, Fennec (climate program), Fourth National Climate Assessment, Freedom of Information requests to the Climatic Research Unit, G8 Climate Change Roundtable, Garnaut Climate Change Review, Generation Climate Europe, Geologic temperature record, German Climate Action Plan 2050, German Climate Consortium, German Renewable Energy Sources Act, Glasgow Climate Pact, Global Atmosphere Watch, Global Climate Action (portal), Global Climate Action Summit, Global Climate and Energy Project, Global Climate Coalition, Global Climate Network, Global climate regime, Global Covenant of Mayors for Climate & Energy, Global Day of Climate Action 2020, Global Environmental Change, Global Historical Climatology Network, Global Roundtable on Climate Change, Global temperature record, Global warming conspiracy theory, Global warming controversy, Global warming game, Global warming hiatus, Global Warming Policy Foundation, Global Warming Pollution Reduction Act of 2007, Global warming potential, Global Warming Solutions Act of 2006, Global warming taxes, Global Warming: The Signs and The Science, Global Warming: What You Need to Know, Glossary of climate change, Gorgon Carbon Dioxide Injection Project, Great March for Climate Action, Green Climate Fund, Green House Data, Green New Deal, Greenhouse and icehouse Earth, Greenhouse debt, Greenhouse Development Rights, Greenhouse effect,

Greenhouse gas, Greenhouse gas emissions, Greenhouse gas emissions by Australia, Greenhouse gas emissions by China, Greenhouse gas emissions by Russia, Greenhouse gas emissions by the United Kingdom, Greenhouse gas emissions by the United States, Greenhouse gas emissions by Turkey, Greenhouse gas emissions in Kentucky, Greenhouse gas inventory, Greenhouse gas monitoring, Greenhouse Gas Pollution Pricing Act, Greenhouse Gases Observing Satellite, Greenhouse Mafia, Greenhouse Solutions with Sustainable Energy, Ground level ozone, Gussing Renewable Energy, High Level Advisory Group on Climate Financing, High Plains Regional Climate Center, Highest temperature recorded on Earth, Historical climatology, History of climate change policy and politics, History of climate change science, Holocene climatic optimum, Homogenization (climate), How Global Warming Works, How to Prepare for Climate Change, Human rights and climate change, Ice cap climate, IDA Indoor Climate and Energy, Illustrative model of greenhouse effect on climate change, Index of climate change articles, India Climate Collaborative, Indian Network on Climate Change Assessment, Indian Youth Climate Network, Indigenous Peoples Climate Change Assessment Initiative, Individual action on climate change, Inside Climate News, Instrumental temperature record, Intergovernmental Panel on Climate Change, Interim Climate Change Committee, International Climate Change Partnership, International Comprehensive Ocean-Atmosphere Data Set, International Conference on Climate Change, International Geosphere-Biosphere Programme, International Indigenous Peoples Forum on Climate Change, International Journal of Climatology, International Journal of Greenhouse Gas Control, International Renewable Energy Agency, International Satellite Cloud Climatology Project, IPCC Fifth Assessment Report, IPCC First Assessment Report, IPCC Fourth Assessment Report, IPCC list of greenhouse gases, IPCC Second Assessment Report, IPCC Sixth Assessment Report, IPCC Summary for Policymakers, IPCC supplementary report, 1992, IPCC Third Assessment Report, Johannesburg Renewable Energy Coalition, Journal for Geoclimatic Studies, Journal of Applied Meteorology and Climatology, Journal of Climate, Laboratoire des sciences du climat et de l'environnement, Land surface effects on climate, Life-cycle greenhouse gas emissions of energy sources, Liquid carbon dioxide, List of books about renewable energy, List of climate change books, List of climate change initiatives, List of climate engineering topics, List of climate research satellites, List of climate scientists, List of countries and territories by extreme temperatures, List of countries by carbon dioxide emissions, List of countries by carbon dioxide emissions per capita, List of countries by greenhouse gas emissions, List of countries by greenhouse gas emissions per person, List of extreme temperatures in Australia, List of extreme temperatures in Canada, List of extreme temperatures in Denmark, List of extreme temperatures in Finland, List of extreme temperatures in France, List of extreme temperatures in Germany, List of extreme temperatures in Italy, List of extreme temperatures in Portugal, List of extreme temperatures in Spain, List of extreme temperatures in Sweden, List of extreme temperatures in Vatican City, List of extreme weather records in Pakistan, List of films about renewable energy, List of ministers of climate change, List of people associated with renewable energy, List of periods and events in climate history, List of renewable energy companies by stock exchange, List of renewable energy organizations, List of renewable energy topics by country and territory, List of school climate strikes, List of U.S. states and territories by carbon dioxide emissions, Lists of renewable energy topics, London Climate Change Agency, Low Carbon Communities, Low Carbon Vehicle Event, Lowest temperature recorded on Earth, Maldivian Youth Climate Network, Mandatory renewable energy target, Mayors National Climate Action Agenda, Media coverage of climate change, Mercator Research Institute on Global Commons and Climate Change, Midwestern Greenhouse Gas Reduction Accord, Minister for Climate Change (New Zealand), Ministry of Climate Change (Pakistan), Ministry of Climate, Energy and Utilities (Denmark), Ministry of Economic Affairs and Climate Policy, Ministry of Electricity and Renewable Energy (Egypt), Ministry of Environment, Forest and Climate Change, Ministry of New and Renewable Energy, Ministry of Power and Renewable Energy, Monthly Climatic Data for the World, Muslim Seven Year Action Plan on Climate Change, National Action Plan for Climate Change, National Climate Assessment, National Climate Change Secretariat, National Climatic Data Center, National Initiative on Climate Resilient Agriculture, National Oceanic and Atmospheric Administration Climate and Societal Interactions Program, National Renewable Energy Action Plan, National Solar Conference and World Renewable Energy Forum 2012, Nature Climate Change, New England Governors and Eastern Canadian Premiers Climate Change Action Plan 2001, New South Wales Greenhouse Gas Abatement Scheme, New York City Panel on Climate Change, New Zealand Climate Science Coalition, Nigeria Renewable Energy Master Plan, Noordwijk Climate Conference, North African climate cycles, Nuclear power proposed as renewable energy, NZ Climate Party, Ocean storage of carbon dioxide, Office of Energy Efficiency and Renewable Energy, OneClimate, Ozone, Ozone Action Day, Ozone depletion, Ozone depletion and climate change, Pacific Climate Warriors, Palaeogeography, Palaeoclimatology, Palaeoecology, Pan-African Media Alliance on Climate Change, Pan-Canadian Framework on Clean Growth and Climate Change, Pastoral Greenhouse Gas Research Consortium, People's Climate

Movement, Photochemical reduction of carbon dioxide, Photoelectrochemical reduction of carbon dioxide, Physical properties of greenhouse gases, Poland National Renewable Energy Action Plan, Political economy of climate change, Politics of climate change, Portal:Climate change, Portal:Renewable energy, Potsdam Institute for Climate Impact Research, Premier's Climate Change Council, Presbyterian Church (U.S.A.) Carbon Neutral Resolution, Presidential Climate Action Plan, Program for Climate Model Diagnosis and Intercomparison, Program on Energy Efficiency in Artisanal Brick Kilns in Latin America to Mitigate Climate Change, Proxy (climate), Psychological impact of climate change, Public opinion on climate change, Punjab Renewable Energy Systems Pvt. Ltd., R20 Regions of Climate Action, Rapid Climate Change-Meridional Overturning Circulation and Heatflux Array, Recognizing the duty of the Federal Government to create a Green New Deal, Reflective surfaces (climate engineering), Regional climate levels in viticulture, Regional Greenhouse Gas Initiative, Regulation of greenhouse gases under the Clean Air Act, Renewable energy, Renewable Energy (journal), Renewable energy and mining, Renewable Energy Certificate (United States), Renewable Energy Certificate System, Renewable Energy Certificates Registry, Renewable energy commercialization, Renewable energy cooperative, Renewable energy debate, Renewable energy in Brunei, Renewable energy in developing countries, Renewable energy in Luxembourg, Renewable energy industry, Renewable Energy Payments, Renewable energy policy of Bangladesh, Renewable energy sculpture, Renewable Energy Sources and Climate Change Mitigation, Renewable energy transition, Runaway greenhouse effect, Running on Climate, San Diego Climate Action Plan, San Francisco Climate Action Plan, Save the Climate, School Strike for Climate, Scientific consensus on climate change, Scorcher: The Dirty Politics of Climate Change, Sea ice emissivity modelling, Sea ice growth processes, Sea ice thickness, Sea level rise, Seawater greenhouse, September 2019 climate strikes, Singularity (climate), Skin temperature (of an atmosphere), Soft climate change denial, Soil-plant-atmosphere continuum, Solar activity and climate, Solar Renewable Energy Certificate, South Pacific Sea Level and Climate Monitoring Project, Space mirror (climate engineering), Space-based measurements of carbon dioxide, Special Report on Climate Change and Land, Special Report on the Ocean and Cryosphere in a Changing Climate, State of the Climate, Stratospheric Processes And their Role in Climate, Subtropical climate vegetated roof, Supercritical carbon dioxide, Surface Ocean Lower Atmosphere Study, Surveys of scientists' views on climate change, Sweden National Renewable Energy Action Plan, Table of historic and prehistoric climate indicators, Tarawa Climate Change Conference, Task Force on Climate Related Financial Disclosures, Temperature record of the last 2,000 years, Template:Climate change in Canada, Template:Climate-change-stub, Template:Climate-journal-stub, Template:Climate-stub, Territorial Approach to Climate Change, The Climate Group, The Climate Mobilization, The Climate Reality Project, The Climate Registry, The Doubt Machine: Inside the Koch Brothers' War on Climate Science, The Great Derangement: Climate Change and the Unthinkable, The Great Global Warming Swindle, The Greenhouse Conspiracy, The Islamic Declaration on Global Climate Change, The New Climate War, Theoretical and Applied Climatology, Thornthwaite climate classification, Tianjin Climate Exchange, Tipping points in the climate system, Top contributors to greenhouse gas emissions, Toronto Conference on the Changing Atmosphere, Transatlantic Climate Bridge, Transient climate response to cumulative carbon emissions, Transportation and Climate Initiative, Trewartha climate classification, Tropical cyclones and climate change, Tropospheric ozone depletion events, U.S. Climate Action Partnership, U.S. Climate Change Technology Program, U.S. Special Presidential Envoy for Climate, UK Climate Assembly, UK Health Alliance on Climate Change, United Kingdom Climate Change Programme, United Kingdom National Renewable Energy Action Plan, United Nations Climate Change conference, United Nations Special Envoy on Climate Change, United States Climate Alliance, United States federal register of greenhouse gas emissions, United States House Select Committee on Energy Independence and Global Warming, United States House Select Committee on the Climate Crisis, United States rainfall climatology, UNSW School of Photovoltaic and Renewable Energy Engineering, Urban climatology, Urbanization and Global Environmental Change Project, US Climate Reference Network, VAMOS Ocean-Cloud-Atmosphere-Land Study, Vatican Climate Forest, Wadebridge Renewable Energy Network, Walloon platform for the IPCC, Waterborne disease and climate change, Weather, Climate, and Society, Western Climate Initiative, Western Hemisphere Warm Pool, Weyburn-Midale Carbon Dioxide Project, White House National Climate Advisor, White House Office of Energy and Climate Change Policy, Women in climate change, World Climate Change Conference, Moscow, World Climate Conference, World Climate Programme, World Mayors Council on Climate Change, World People's Conference on Climate Change, World Renewable Energy Network, World Wide Views on Global Warming, Wuppertal Institute for Climate, Environment and Energy, XCO2, Yale Program on Climate Change Communication, Young Voices on Climate Change, Youth Climate Movement

List of English Wikipedia articles used to track public engagement in health

1510 influenza pandemic, 1557 influenza pandemic, 1775–1782 North American smallpox epidemic, 1793 Philadelphia yellow fever epidemic, 1837 Great Plains smallpox epidemic, 1847 North American typhus epidemic, 1856 Guam smallpox epidemic, 1862 Pacific Northwest smallpox epidemic, 1870 Barcelona yellow fever epidemic, 1881–1896 cholera pandemic, 1896 Gloucester smallpox epidemic, 1906 malaria outbreak in Ceylon, 1915 typhus and relapsing fever epidemic in Serbia, 1918 flu pandemic in India, 1961–1975 cholera pandemic, 1974 smallpox epidemic in India, 1985 World Health Organization AIDS surveillance case definition, 1994 expanded World Health Organization AIDS case definition, 1998 Winter Olympics flu epidemic, 2009 swine flu pandemic, 2009 swine flu pandemic in India, 2009 swine flu pandemic timeline, 2013 Swansea measles epidemic, 2018 Madagascar measles outbreak, 2021 South Sudan disease outbreak, 412 BC epidemic, Abrazo Community Health Network, Academy of Nutrition and Dietetics, Access Health CT, Accredited Social Health Activist, Action on Smoking and Health, Acute eosinophilic pneumonia, Adenovirus infection, Adult-onset Still's disease, AdventHealth, AdventHealth Celebration, AdventHealth Lake Wales, AdventHealth Nicholson Center, AdventHealth Ocala, AdventHealth Orlando, AdventHealth Shawnee Mission, AdventHealth station, AdventHealth Wauchula, Adventist Health, Adventist Health Bakersfield, Adventist Health Community Care-Hanford, Adventist Health Feather River, Adventist Health Glendale, Adventist Health Portland, Affordable Medicines Facility-malaria, Africa Centres for Disease Control and Prevention, Africa Fighting Malaria, African Health Economics and Policy Association, African Malaria Network Trust, African Nutrition Leadership Programme, Against Malaria Foundation, Aging-associated diseases, Aid and relief efforts during the COVID-19 pandemic in Malaysia, Air pollution, Air pollution and traffic congestion in Tehran, Air pollution forecasting, Air pollution in Hong Kong, Air pollution in Macau, Air pollution sensor, Air Quality Health Index (Canada), Airport malaria, Akureyri disease, Alcohol and health, Alcoholic liver disease, Alcoholic lung disease, Alexander disease, Alliance for Health Policy and Systems Research, Alliance for Healthy Cities, AllianceHealth Durant, Alzheimer's disease biomarkers, Alzheimer's Disease Cooperative Study, Alzheimer's disease in the media, Alzheimer's Disease Neuroimaging Initiative, Amazon Malaria Initiative, American Association for the Study and Prevention of Infant Mortality, American Association of Public Health Dentistry, American Association of Public Health Physicians, American College of Epidemiology, American Journal of Epidemiology, American Public Health Association, American School Health Association, American Sexual Health Association, American Society for Nutrition, American Society for Parenteral and Enteral Nutrition, AMITA Health, Anaerobic infection, Andersen healthcare utilization model, Animal nutrition, AnMed Health Women's & Children's Hospital, Annals of Epidemiology, Anti-AQP4 disease, Anti-IgLON5 disease, Antidiarrhoeal, Antimalarial medication, Apparent infection rate, Applications of sensitivity analysis in epidemiology, Asia Pacific Leaders Malaria Alliance, Aspiration pneumonia, Aspirin exacerbated respiratory disease, Association for Community Health Improvement, Association of Medical Microbiology and Infectious Disease Canada, Association of Public Health Laboratories, Atrium Health, Atrium Health Cabarrus, Atrium Health Mercy, Atrium Health Pineville, Atrium Health Union, Atrium Health University City, Atrium Health Wake Forest Baptist, Atypical pneumonia, Australian Health Protection Principal Committee, Australian Longitudinal Study on Women's Health, Australian Measles Control Campaign, Autoimmune disease, Autoimmune disease in women, Autoimmune inner ear disease, Autoimmune skin diseases in dogs, Autoinflammatory diseases, Autosomal dominant polycystic kidney disease, Autosomal recessive polycystic kidney disease, Avalere Health, Bacterial pneumonia, Balwadi Nutrition Programme, Bamrasnaradura Infectious Diseases Institute, Bandim Health Project, Bangladesh Institute of Child and Mother Health, Bangladesh Institute of Child Health, Bangladesh National Nutrition Council, Baptist Health System, Batten disease, Baumol's cost disease, Bayfront Health Punta Gorda, Bayfront Health St. Petersburg, Behavior change (public health), Belgian Health Care Knowledge Centre, BENTA disease, Bill of health, Bills of mortality, Binswanger's disease, Biochemistry of Alzheimer's disease, Biologically based mental illness, Biphasic disease, Black Maternal Health Caucus, Black Women's Health Study, Blackheart (plant disease), Blood-borne disease, Bloodstream infections, Blount's disease, Bluetongue disease, Bombay plague epidemic, Bone health, Borna disease, Brazilian Health Regulatory Agency, British government response to the COVID-19 pandemic, Bronchopneumonia, Brookwood Baptist Health, Busselton Health Study, Caerphilly Heart Disease Study, Calcium pyrophosphate dihydrate crystal deposition disease, California Center for Public Health Advocacy, Canada Health Act, Canada Health and Social Transfer, Canadian Classification of Health Interventions, Canadian Public Health Association, Canadian Society for Epidemiology and Biostatistics, Canavan disease, Cancer Epidemiology (journal), Cancer Epidemiology, Biomarkers & Prevention, Canine vector-borne disease, Capitation (healthcare), Cardiovascular disease, Caribbean Public Health Agency, Carlos III Health Institute,

Caroli disease, Carolinas College of Health Sciences, Carondelet Health Network, Carrion's disease, Case management (mental health), Castleman disease, Cat-scratch disease, Catheter-associated urinary tract infection, Causes of mental disorders, Center for Global Infectious Disease Research, Center for Infectious Disease Research and Policy, Center for World Health and Medicine, Central Epidemic Command Center, Centre for Health and International Relations, Centre for Health Protection, Centre for History in Public Health, London School of Hygiene and Tropical Medicine, Chagas disease, Chicago 1885 cholera epidemic myth, Child Health and Nutrition Research Initiative, Child health in Uganda, Child mortality, Childhood chronic illness, Children's Healthcare of Atlanta, Children's Healthcare of Atlanta at Egleston, Children's Healthcare of Atlanta at Hughes Spalding, Children's Healthcare of Atlanta at Scottish Rite, Children's right to adequate nutrition in New Zealand, Chinese Center for Disease Control and Prevention, Chinese Classification of Mental Disorders, Chlamydia pneumoniae, Chloroquine and hydroxychloroquine during the COVID-19 pandemic, Cholera outbreaks and pandemics, Choosing Healthplans All Together, Chronic active EBV infection, Chronic kidney disease, Chronic Lyme disease, Chronic obstructive pulmonary disease, Cinematography in healthcare, Circle of Health International, Classification of mental disorders, Classification of pneumonia, Climate change and infectious diseases, Clinical epidemiology, Clinical Epidemiology (journal), Clinical Health Promotion, Clinical nutrition, Clinton health care plan of 1993, Clostridioides difficile infection, CNS demyelinating autoimmune diseases, Coalition for Epidemic Preparedness Innovations, Cognitive epidemiology, Cohorts for Heart and Aging Research in Genomic Epidemiology, Coinfection, Cold agglutinin disease, Colorado Department of Health Care Policy and Financing, Commission on Health Research for Development, Commission on the Accreditation of Healthcare Management Education, Common disease-common variant, CommonSpirit Health, Community Dentistry and Oral Epidemiology, Community health, Community health agent, Community Health Clubs in Africa, Community Health Systems, Community-acquired pneumonia, Comparison of the healthcare systems in Canada and the United States, Compartmental models in epidemiology, Comprehensive Health Assessment Program, Compression of morbidity, Computational epidemiology, Cone Health, Cone Health Behavioral Health Hospital, Cone Health Women's Hospital, Conflict epidemiology, Congenital cytomegalovirus infection, Congenital malaria, Consortium of Universities for Global Health, Contagious bovine pleuropneumonia, Contagious disease, Convention on Long-Range Transboundary Air Pollution, Core Cities Health Improvement Collaborative, Corn stunt disease, Coronary artery disease, Coronavirus diseases, Coughs and sneezes spread diseases, Council on Education for Public Health, COVID-19 pandemic, COVID-19 pandemic by country and territory, COVID-19 pandemic in Abkhazia, COVID-19 pandemic in Afghanistan, COVID-19 pandemic in Akrotiri and Dhekelia, COVID-19 pandemic in Albania, COVID-19 pandemic in Algeria, COVID-19 pandemic in American Samoa, COVID-19 pandemic in Andorra, COVID-19 pandemic in Angola, COVID-19 pandemic in Antigua and Barbuda, COVID-19 pandemic in Argentina, COVID-19 pandemic in Armenia, COVID-19 pandemic in Asia, COVID-19 pandemic in Australia, COVID-19 pandemic in Austria, COVID-19 pandemic in Azerbaijan, COVID-19 pandemic in Bahrain, COVID-19 pandemic in Bangladesh, COVID-19 pandemic in Barbados, COVID-19 pandemic in Belarus, COVID-19 pandemic in Belgium, COVID-19 pandemic in Belize, COVID-19 pandemic in Benin, COVID-19 pandemic in Bhutan, COVID-19 pandemic in Bolivia, COVID-19 pandemic in Bosnia and Herzegovina, COVID-19 pandemic in Botswana, COVID-19 pandemic in Brazil, COVID-19 pandemic in Brunei, COVID-19 pandemic in Bulgaria, COVID-19 pandemic in Burkina Faso, COVID-19 pandemic in Burundi, COVID-19 pandemic in Cambodia, COVID-19 pandemic in Cameroon, COVID-19 pandemic in Canada, COVID-19 pandemic in Cape Verde, COVID-19 pandemic in Chad, COVID-19 pandemic in Chile, COVID-19 pandemic in Colombia, COVID-19 pandemic in Costa Rica, COVID-19 pandemic in Crimea, COVID-19 pandemic in Croatia, COVID-19 pandemic in Cuba, COVID-19 pandemic in Cyprus, COVID-19 pandemic in Denmark, COVID-19 pandemic in Djibouti, COVID-19 pandemic in Dominica, COVID-19 pandemic in East Timor, COVID-19 pandemic in Easter Island, COVID-19 pandemic in Ecuador, COVID-19 pandemic in Egypt, COVID-19 pandemic in El Salvador, COVID-19 pandemic in England, COVID-19 pandemic in Equatorial Guinea, COVID-19 pandemic in Eritrea, COVID-19 pandemic in Estonia, COVID-19 pandemic in Eswatini, COVID-19 pandemic in Ethiopia, COVID-19 pandemic in Europe, COVID-19 pandemic in Fiji, COVID-19 pandemic in Finland, COVID-19 pandemic in France, COVID-19 pandemic in French Polynesia, COVID-19 pandemic in Gabon, COVID-19 pandemic in Gagauzia, COVID-19 pandemic in Georgia (country), COVID-19 pandemic in Germany, COVID-19 pandemic in Ghana, COVID-19 pandemic in Gibraltar, COVID-19 pandemic in Greece, COVID-19 pandemic in Greenland, COVID-19 pandemic in Grenada, COVID-19 pandemic in Guatemala, COVID-19 pandemic in Guernsey, COVID-19 pandemic in Guinea, COVID-19 pandemic in Guinea-Bissau, COVID-19 pandemic in Guyana, COVID-19 pandemic in Haiti, COVID-19 pandemic in Honduras, COVID-19 pandemic in Hong Kong, COVID-19 pandemic in Hungary, COVID-19 pandemic in Iceland, COVID-19 pandemic in

India, COVID-19 pandemic in Indonesia, COVID-19 pandemic in Iran, COVID-19 pandemic in Iraq, COVID-19 pandemic in Israel, COVID-19 pandemic in Italy, COVID-19 pandemic in Ivory Coast, COVID-19 pandemic in Jamaica, COVID-19 pandemic in Japan, COVID-19 pandemic in Jersey, COVID-19 pandemic in Jordan, COVID-19 pandemic in Kazakhstan, COVID-19 pandemic in Kenya, COVID-19 pandemic in Kiribati, COVID-19 pandemic in Kosovo, COVID-19 pandemic in Kuwait, COVID-19 pandemic in Kyrgyzstan, COVID-19 pandemic in Laos, COVID-19 pandemic in Latvia, COVID-19 pandemic in Lebanon, COVID-19 pandemic in Liechtenstein, COVID-19 pandemic in Lithuania, COVID-19 pandemic in Luxembourg, COVID-19 pandemic in Macau, COVID-19 pandemic in mainland China, COVID-19 pandemic in Malaysia, COVID-19 pandemic in Malta, COVID-19 pandemic in Moldova, COVID-19 pandemic in Monaco, COVID-19 pandemic in Mongolia, COVID-19 pandemic in Montenegro, COVID-19 pandemic in Myanmar, COVID-19 pandemic in Nepal, COVID-19 pandemic in New South Wales, COVID-19 pandemic in Normandy, COVID-19 pandemic in North Korea, COVID-19 pandemic in North Macedonia, COVID-19 pandemic in Northern Cyprus, COVID-19 pandemic in Northern Ireland, COVID-19 pandemic in Norway, COVID-19 pandemic in Oman, COVID-19 pandemic in Pakistan, COVID-19 pandemic in Poland, COVID-19 pandemic in Portugal, COVID-19 pandemic in Qatar, COVID-19 pandemic in Queensland, COVID-19 pandemic in Romania, COVID-19 pandemic in Russia, COVID-19 pandemic in San Marino, COVID-19 pandemic in Saudi Arabia, COVID-19 pandemic in Scotland, COVID-19 pandemic in Serbia, COVID-19 pandemic in Sevastopol, COVID-19 pandemic in Singapore, COVID-19 pandemic in Slovakia, COVID-19 pandemic in Slovenia, COVID-19 pandemic in South Asia, COVID-19 pandemic in South Australia, COVID-19 pandemic in South Korea, COVID-19 pandemic in South Ossetia, COVID-19 pandemic in Southeast Asia, COVID-19 pandemic in Spain, COVID-19 pandemic in Sri Lanka, COVID-19 pandemic in Svalbard, COVID-19 pandemic in Sweden, COVID-19 pandemic in Switzerland, COVID-19 pandemic in Syria, COVID-19 pandemic in Taiwan, COVID-19 pandemic in Tajikistan, COVID-19 pandemic in Tasmania, COVID-19 pandemic in Thailand, COVID-19 pandemic in the Australian Capital Territory, COVID-19 pandemic in the Bahamas, COVID-19 pandemic in the Central African Republic, COVID-19 pandemic in the Comoros, COVID-19 pandemic in the Cook Islands, COVID-19 pandemic in the Czech Republic, COVID-19 pandemic in the Democratic Republic of the Congo, COVID-19 pandemic in the Dominican Republic, COVID-19 pandemic in the Donetsk People's Republic, COVID-19 pandemic in the European Union, COVID-19 pandemic in the Faroe Islands, COVID-19 pandemic in the Federated States of Micronesia, COVID-19 pandemic in the Gambia, COVID-19 pandemic in the Guantanamo Bay Naval Base, COVID-19 pandemic in the Isle of Man, COVID-19 pandemic in the Kurdistan Region, COVID-19 pandemic in the Luhansk People's Republic, COVID-19 pandemic in the Maldives, COVID-19 pandemic in the Netherlands, COVID-19 pandemic in the Northern Territory, COVID-19 pandemic in the Philippines, COVID-19 pandemic in the Republic of Artsakh, COVID-19 pandemic in the Republic of Ireland, COVID-19 pandemic in the Republic of the Congo, COVID-19 pandemic in the State of Palestine, COVID-19 pandemic in the United Arab Emirates, COVID-19 pandemic in the United Kingdom, COVID-19 pandemic in Transnistria, COVID-19 pandemic in Turkey, COVID-19 pandemic in Turkmenistan, COVID-19 pandemic in Ukraine, COVID-19 pandemic in Uzbekistan, COVID-19 pandemic in Vatican City, COVID-19 pandemic in Victoria, COVID-19 pandemic in Vietnam, COVID-19 pandemic in Wales, COVID-19 pandemic in Western Australia, COVID-19 pandemic in Yemen, COVID-19 pandemic on Charles de Gaulle, COVID-19 pandemic on Diamond Princess, Creativity and mental health, Creutzfeldt-Jakob Disease Surveillance System, Creutzfeldt–Jakob disease, Critical illness insurance, Crohn's disease, Cryptogenic organizing pneumonia, Cytomegaloviral disease, Cytomegalovirus infection, Dance and health, Deen Dayal Mobile Health Clinic, Degenerative disc disease, Degenerative disease, Dementia and Alzheimer's disease in Australia, Dengue pandemic in Sri Lanka, Depression of Alzheimer disease, Derzsy's disease, Desquamative interstitial pneumonia, Diabetic foot infection, Diagnosis of malaria, Diarrheal disease, Diarrhoea, Dignity Health, Dignity Health St. Joseph's Hospital and Medical Center, Director-General of the World Health Organization, Directorate of Health, Disability and women's health, Discovery of disease-causing pathogens, Disease, Disease burden, Disease cluster, Disease Control Priorities Project, Disease diffusion mapping, Disease ecology, Disease management (health), Disease outbreak, Disease resistance, Disease surveillance, Disease vector, Disease X, Disease-modifying osteoarthritis drug, Diseases, Diseases of abnormal polymerization, Diseases of despair, Diseases of poverty, Disseminated disease, Doctor of Public Health, Dole Nutrition Institute, Drugs for Neglected Diseases Initiative, Dukes' disease, Dunedin Multidisciplinary Health and Development Study, Dust pneumonia, E-epidemiology, Early-onset Alzheimer's disease, Ecological health, Economic epidemiology, Economic impact of the COVID-19 pandemic in Malaysia, Economic impact of the COVID-19 pandemic in the Republic of Ireland, Ecosystem health, Effects of climate change on human health, Ehrlichiosis ewingii infection, EMBRACE Healthcare Reform Plan, Emerging infectious disease, Emerging Themes in

Epidemiology, Endemic (epidemiology), Endogenous infection, Engineering World Health, Environmental disease, Environmental epidemiology, Environmental health, Environmental health ethics, Environmental health officer, Environmental health policy, Eosinophilic pneumonia, Epidemic, Epidemic curve, Epidemic Diseases Act, 1897, Epidemic Intelligence Service, Epidemic models on lattices, Epidemic polyarthritis, Epidemic typhus, Epidemics (journal), Epidemiology, Epidemiology (journal), Epidemiology and Infection, Epidemiology and Psychiatric Sciences, Epidemiology data for low-linear energy transfer radiation, Epidemiology in Country Practice, Epidemiology in Relation to Air Travel, Epidemiology of asthma, Epidemiology of attention deficit hyperactive disorder, Epidemiology of bed bugs, Epidemiology of binge drinking, Epidemiology of breast cancer, Epidemiology of cancer, Epidemiology of chikungunya, Epidemiology of child psychiatric disorders, Epidemiology of childhood obesity, Epidemiology of depression, Epidemiology of diabetes, Epidemiology of malnutrition, Epidemiology of measles, Epidemiology of metabolic syndrome, Epidemiology of plague, Epidemiology of pneumonia, Epidemiology of schizophrenia, Epidemiology of syphilis, Epidemiology of typhoid fever, Epstein–Barr virus-associated lymphoproliferative diseases, Eradication of infectious diseases, Escape Fire: The Fight to Rescue American Healthcare, Essence (Electronic Surveillance System for the Early Notification of Community-based Epidemics), Establishment of the World Health Organization, EuroHealthNet, European Centre for Disease Prevention and Control, European Health Examination Survey, European Health Forum Gastein, European health management association, European Journal of Epidemiology, European Observatory on Health Systems and Policies, European Parliament Committee on the Environment, Public Health and Food Safety, European Programme for Intervention Epidemiology Training, European Prospective Investigation into Cancer and Nutrition, European Public Health Alliance, European Public Health Association, European Society for Clinical Nutrition and Metabolism, European Society for Paediatric Infectious Diseases, European Society of Clinical Microbiology and Infectious Diseases, European Union response to the COVID-19 pandemic, European Working Group for Legionella Infections, Evacuations by India related to the COVID-19 pandemic, Evacuations by the Philippines related to the COVID-19 pandemic, Evaluation & the Health Professions, Evolution of Infectious Disease, Evolutionary epidemiology, Face masks during the COVID-19 pandemic, Fair Share Health Care Act, Febrile infection-related epilepsy syndrome, Federal aid during the COVID-19 pandemic in Canada, Federal Service for Surveillance in Healthcare, Federation of European Nutrition Societies, Feminist health centers, Fifth disease, Finnish Institute for Health and Welfare, Fire breather's pneumonia, First Nations nutrition experiments, Focus of infection, Foodborne illness, Foot-and-mouth disease, Free-market healthcare, Fungal infection, Fungal pneumonia, Gamaleya Research Institute of Epidemiology and Microbiology, Gastrointestinal disease, Gender disparities in health, General Health Questionnaire, General Health System (Cyprus), Genetic epidemiology, Genetic Epidemiology (journal), George Institute for Global Health, Germ theory of disease, German government response to the COVID-19 pandemic, Ghana Infectious Disease Centre, Ghanaian government response to the COVID-19 pandemic, GIS and public health, Global Acute Malnutrition, Global Alliance for Improved Nutrition, Global Alliance on Health and Pollution, Global Burden of Disease Study, Global Coalition Against Pneumonia, Global Forum for Health Research, Global health, Global Health Council, Global Health Delivery Project, Global Health Initiatives, Global Health Innovative Technology Fund, Global Health Observatory, Global Health Security Agenda, Global Health Security Index, Global Health Security Initiative, Global Health Share Initiative, Global Infectious Disease Epidemiology Network, Global Initiative for Chronic Obstructive Lung Disease, Global Malaria Action Plan, Global mental health, Global Network for Neglected Tropical Diseases, Global Public Health Intelligence Network, Global Research Collaboration for Infectious Disease Preparedness, Global Strategy for Women's and Children's Health, Globalization and disease, Glossary of the COVID-19 pandemic, Goal-oriented health care, Gold Coast Influenza Epidemic, Graduate School of Health Economics and Management, Grand Challenges In Global Health, Graves' disease, Groningen epidemic, Grossman model of health demand, Group B streptococcal infection, Gulf War Health Research Reform Act of 2014, Handbook of Religion and Health, HCA Healthcare, Health (Preservation and Protection and other Emergency Measures in the Public Interest) Act 2020, Health 21, Health Action International, Health administration, Health advocacy, Health Alliance International, Health and Social Protection Federation, Health and wealth, Health and welfare trust, Health belief model, Health Books International, Health Canada, Health care access among Dalits in India, Health Care Card, Health Care Compact, Health care efficiency measures, Health care finance in the United States, Health Care for Women International, Health care in Argentina, Health care in Australia, Health Care in Canada Survey, Health care in Colombia, Health care in Cyprus, Health care in France, Health care in Liberia, Health care in Mozambique, Health care in Panama, Health care in Poland, Health care in Saudi Arabia, Health care in Spain, Health care in the Philippines, Health care in Turkey, Health care in Venezuela, Health care prices in the United States, Health care ratings, Health care rationing, Health care reforms proposed during the Obama

administration, Health care system in Japan, Health care system of the elderly in Germany, Health care systems by country, Health care time and motion study, Health Code, Health consequences of the Deepwater Horizon oil spill, Health crisis, Health Data Insight, Health departments in the United States, Health disaster, Health Dynamics Inventory, Health economics, Health economics (Germany), Health Economics, Policy and Law, Health education, Health Education & Behavior, Health effect, Health effects of chocolate, Health effects of phenols and polyphenols, Health effects of salt, Health effects of tea, Health effects of wine, Health Emergencies Programme (WHO), Health equity, Health fair, Health For All, Health geography, Health human resources, Health humanities, Health impact assessment, Health in All Policies, Health in Northern Cyprus, Health in Poland, Health informatics in China, Health Information National Trends Survey, Health information on Wikipedia, Health insurance, Health insurance coverage in the United States, Health insurance in China, Health insurance marketplace, Health literacy, Health marketing, Health measures during the construction of the Panama Canal, Health Metrics Network, Health of Hillary Clinton, Health of Towns Association, Health policy, Health policy and management, Health policy in Bangladesh, Health politics, Health promotion, Health promotion in higher education, Health Promotion International, Health Promotion Practice, Health Protection Surveillance Centre, Health risk assessment, Health Sciences Online, Health security, Health Security Express, Health services research, Health Services Workers' Union, Health Spending Account, Health Star Rating System, Health surveillance, Health system, Health systems strengthening, Health Threat Unit, Health Utilities Index, Health-related embarrassment, Healthcare in Afghanistan, Healthcare in Albania, Healthcare in Algeria, Healthcare in Angola, Healthcare in Austria, Healthcare in Azerbaijan, Healthcare in Bahrain, Healthcare in Belgium, Healthcare in Brazil, Healthcare in Canada, Healthcare in Chennai, Healthcare in China, Healthcare in Croatia, Healthcare in Cuba, Healthcare in Denmark, Healthcare in Egypt, Healthcare in Estonia, Healthcare in Ethiopia, Healthcare in Finland, Healthcare in Georgia (country), Healthcare in Germany, Healthcare in Ghana, Healthcare in Greece, Healthcare in Hungary, Healthcare in Iceland, Healthcare in India, Healthcare in Indonesia, Healthcare in Iran, Healthcare in Iraq, Healthcare in Israel, Healthcare in Italy, Healthcare in Kenya, Healthcare in Kuwait, Healthcare in Luxembourg, Healthcare in Madagascar, Healthcare in Malawi, Healthcare in Malta, Healthcare in Mexico, Healthcare in Moldova, Healthcare in Nicaragua, Healthcare in Norway, Healthcare in Peru, Healthcare in Portugal, Healthcare in Qatar, Healthcare in Romania, Healthcare in Russia, Healthcare in Rwanda, Healthcare in Saint Helena, Healthcare in San Marino, Healthcare in Senegal, Healthcare in Serbia, Healthcare in Sierra Leone, Healthcare in Singapore, Healthcare in Slovakia, Healthcare in South Africa, Healthcare in South Korea, Healthcare in Taiwan, Healthcare in Tanzania, Healthcare in Thailand, Healthcare in the Czech Republic, Healthcare in the Isle of Man, Healthcare in the Netherlands, Healthcare in the State of Palestine, Healthcare in Tristan da Cunha, Healthcare in Uganda, Healthcare in Ukraine, Healthcare in Zambia, Healthcare number 1450, Healthcare rationing in the United States, Healthcare reform debate in the United States, Healthcare reform in China, Healthcare reform in the United States, Healthcare Spending Account, Healthcare transport, HealthCare Volunteer, HealthCare.gov, HealthConnect, Healthlink Worldwide, HealthMap, HealthOne, HealthRight International, Healthy city, Healthy community design, Healthy development measurement tool, Healthy diet, Healthy Life Years, HealthyWomen, Heat illness, High-deductible health plan, High-dependency unit (mental health), Hispanic Health Council, History of health care reform in the United States, History of malaria, History of mental disorders, History of the COVID-19 pandemic in the United Kingdom, History of USDA nutrition guidelines, Holozoic nutrition, Hong Kong Health Code, Hookworm infection, Hospital-acquired infection, Hospital-acquired pneumonia, Hospital, patients, health, territories, Household air pollution, How to Have Sex in an Epidemic, Human genetic resistance to malaria, Human Heredity and Health in Africa, Human nutrition, Human papillomavirus infection, Hypertensive disease of pregnancy, Idiopathic disease, Idiopathic interstitial pneumonia, Idiopathic multicentric Castleman disease, Idiopathic orbital inflammatory disease, Idiopathic pneumonia syndrome, IgG4-related disease, IgG4-related ophthalmic disease, IgG4-related skin disease, Illinois Health Benefits Exchange, Imagine No Malaria, Impact of the COVID-19 pandemic on children, Impact of the COVID-19 pandemic on education in Ghana, Impact of the COVID-19 pandemic on education in the Republic of Ireland, Impact of the COVID-19 pandemic on education in the United Kingdom, Impact of the COVID-19 pandemic on Gaelic games, Impact of the COVID-19 pandemic on human rights in Argentina, Impact of the COVID-19 pandemic on politics in Malaysia, Indian government response to the COVID-19 pandemic, Indian migrant workers during the COVID-19 pandemic, Indian state government responses to the COVID-19 pandemic, Indigenous health in Australia, Inequality in disease, Infant mortality, Infant nutrition, Infection, Infection Control Society of Pakistan, Infection prevention and control, Infection rate, Infections associated with diseases, Infectious disease (medical specialty), Infectious Disease (Notification) Act 1889, Infectious Disease Pharmacokinetics Laboratory, Infectious diseases, Infectious Diseases Institute, Infectious Diseases Society of America, Inflammatory bowel disease,

Inflammatory demyelinating diseases of the central nervous system, Influenza pandemic, Influx of disease in the Caribbean, Institute for Health Metrics and Evaluation, Institute of Public Health (Bangladesh), Integrated Disease Surveillance Programme, Integrated Management of Childhood Illness, Intentional contagion of infection, Inter-Agency Task Force for the Management of Emerging Infectious Diseases, Interdisciplinary Association for Population Health Science, Intermountain Healthcare, International Association of National Public Health Institutes, International Centre for Migration and Health, International Classification of Diseases, International Classification of Functioning, Disability and Health, International Conference on Emerging Infectious Diseases, International health, International Health Regulations, International Journal of Epidemiology, International Journal of Men's Health, International Lyme and Associated Diseases Society, International Men's Health Week, International Network of Health Promoting Hospitals and Health Services, International Partnership on Avian and Pandemic Influenza, International reactions to the COVID-19 pandemic in Italy, International Society for Environmental Epidemiology, International Society for Infectious Diseases in Obstetrics and Gynaecology, International Society for Pharmacoepidemiology, International Union of Air Pollution Prevention and Environmental Protection Associations, International Volcanic Health Hazard Network, Intestinal infectious diseases, Intradialytic parenteral nutrition, Irish Nutrition and Dietetic Institute, Iron Triangle of Health Care, Isolation (health care), ITU-WHO Focus Group on Artificial Intelligence for Health, James C. Robinson (health economist), James Thornton (health economist), Jembrana disease, Johns Hopkins Center for Health Security, Joondalup Family Health Study, Journal of Clinical Epidemiology, Journal of Epidemiology, Journal of Epidemiology and Biostatistics, Journal of Epidemiology and Community Health, Journal of Exposure Science and Environmental Epidemiology, Journal of Health Economics, Journal of Urban Health, Kawasaki disease, Kettering Health, Kettering Health Dayton, Kettering Health Main Campus, Kids for World Health, Kivu Ebola epidemic, Korea Disease Control and Prevention Agency, Krabbe disease, Kyasanur Forest disease, Laboratory diagnosis of viral infections, Laboratory-acquired infection, Landscape epidemiology, Latent period (epidemiology), Legacy Health, Legionnaires' disease, Lenox Health Greenwich Village, Leveraging Agriculture for Improving Nutrition and Health, Lifestyle disease, Lipid pneumonia, List of AdventHealth hospitals, List of autoimmune diseases, List of countries by health insurance coverage, List of countries by total health expenditure per capita, List of diseases eliminated from the United States, List of epidemics, List of foodborne illness outbreaks, List of foodborne illness outbreaks by death toll, List of foodborne illness outbreaks in the United States, List of infections of the central nervous system, List of infectious diseases, List of infectious diseases causing flu-like syndrome, List of insect-borne diseases, List of Legionnaires' disease outbreaks, List of mental disorders, List of national public health agencies, List of nutrition guides, List of people with motor neuron disease, List of pneumonia deaths, List of sexually transmitted infections by prevalence, List of types of malnutrition, Liver disease, Liverpool Neurological Infectious Diseases Course, Lobar pneumonia, Localized disease, London Declaration on Neglected Tropical Diseases, Lower Mississippi Valley yellow fever epidemic of 1878, Lower respiratory tract infection, Lutheran Health Network, Lyme disease, Lyme Disease Awareness Month, Lyme disease microbiology, Lymphocytic interstitial pneumonia, Lysosomal storage disease, Madras motor neuron disease, Malaria, Malaria and the Caribbean, Malaria antigen detection tests, Malaria Atlas Project, Malaria Consortium, Malaria Control Project, Malaria culture, Malaria Day in the Americas, Malaria Eradication Scientific Alliance, Malaria Journal, Malaria No More, Malaria No More UK, Malaria Policy Advisory Committee, Malaria prophylaxis, Malaria vaccine, Malarial nephropathy, MalariaWorld, Malnutrition, Malnutrition in India, Malnutrition in Kerala, Malnutrition in Peru, Malnutrition in South Africa, Malnutrition in Zimbabwe, Management of Crohn's disease, Managerial epidemiology, Marburg virus disease, Massachusetts smallpox epidemic, Maternal health, Maternal health in Angola, Maternal health in Rwanda, Maternal mortality ratio, Mayaro virus disease, Measles, Measles & Rubella Initiative, Measles hemagglutinin, Measles morbillivirus, Measles resurgence in the United States, Measles vaccine, Medical officer of health, Medical students' disease, Medicines for Malaria Venture, Mekong Basin Disease Surveillance, Melanie's Marvelous Measles, Men's health, Men's health in Australia, Meningococcal disease, Mental disorder, Mental disorders and gender, Mental disorders in fiction, Mental health, Mental health consumer, Mental health first aid, Mental health literacy, Mental illness, Mental illness denial, Mental illness in ancient Greece, Mental illness portrayed in media, Minister of State for Care and Mental Health, Minister of State for Health (UK), Ministry of Health (Panama), Ministry of Health and Welfare (South Korea), Ministry of Health Promotion and Sport (Ontario), Miscarriage and mental illness, Mission Health System, Mississippi Health Project, Mitochondrial disease, Mixed connective tissue disease, Mobile source air pollution, MOG antibody disease, Molecular epidemiology, Morbidity and Mortality Weekly Report, Mosquito-borne disease, Mosquito-malaria theory, Motor neuron disease, Motor Neurone Disease Association, Mount Sinai Health System, MRC Human Nutrition Research, Muesli belt malnutrition, Multifactorial diseases,

Multimorbidity, Music therapy for Alzheimer's disease, My Health Record, Mycobacterium avium-intracellulare infection, Mycoplasma hominis infection, Mycoplasma pneumonia, National Air Pollution Symposium, National Association for Public Health Policy, National Association of County and City Health Officials, National Center for Disease Control and Public Health (Georgia), National Centre for Disease Control, National Centre for Infectious Diseases, National Children's Center for Rural and Agricultural Health and Safety, National Comorbidity Survey, National Emerging Infectious Diseases Laboratories, National Foundation for Infectious Diseases, National Fund for Health Insurance, National Health Accounts, National Health Act 1953, National Health and Nutrition Examination Survey, National health insurance, National Health Insurance (Japan), National Health Interview Survey, National Health Mission, National Health Policy, National Hotel disease, National Institute for Communicable Diseases, National Institute for Health and Care Excellence, National Institute for Health and Disability Insurance, National Institute for Public Health and the Environment, National Institute of Health, Islamabad, National Institute of Malaria Research, National Institute of Nutrition, Hyderabad, National Institute of Public Health of Japan, National Malaria Eradication Program, National Perinatal Epidemiology Unit, National Prostate Health Month, National Public Health Emergency Team (2020), National public health institutes, National Public Health Organization (Greece), National School of Public Health (Spain), Native American disease and epidemics, Native American Women's Health Education Resource Center, Navicent Health Baldwin, Necrotizing pneumonia, Neglected tropical disease research and development, Neglected tropical diseases, Neglected tropical diseases in India, Neonatal infection, Network for Capacity Development in Nutrition, Neuroepidemiology (journal), Nevada Health Link, New Mexico Health Insurance Exchange, NewYork-Presbyterian Healthcare System, Nigel Edwards (health), Nigeria Centre for Disease Control, NINCDS-ADRDA Alzheimer's Criteria, Nipah virus infection, Noma (disease), Non-alcoholic fatty liver disease, Non-communicable disease, Non-communicable diseases, Non-pharmaceutical intervention (epidemiology), Non-specific interstitial pneumonia, Northwell Health, Norwegian Institute of Public Health, Notifiable disease, Notifiable diseases in Sweden, Notifiable diseases in the United Kingdom, Novant Health, Novant Health Forsyth Medical Center, Nurses' Health Study, Nutrition, Nutrition analysis, Nutrition and cognition, Nutrition and Education International, Nutrition and pregnancy, Nutrition education, Nutrition Foundation of the Philippines, Nutrition in classical antiquity, Nutrition psychology, Nutrition scale, Nutrition transition, Nutritional anthropology, Nutritional epidemiology, Nutritional gatekeeper, Nutritional genomics, Nutritional neuroscience, Nutritional rating systems, Nutritional science, NutritionDay, Nutritionist, Obstacles to receiving mental health services among African American youth, Occult pneumonia, Occupational exposure to Lyme disease, Occupational safety and health, Office for Health Improvement and Disparities, Office on Women's Health, One Health, Opportunistic infection, Oregon Medicaid health experiment, OSF HealthCare, Ottawa Charter for Health Promotion, Outline of air pollution dispersion, Overseas Student Health Cover, Overwhelming post-splenectomy infection, Oxford Brookes Centre for Nutrition and Health, Pacific Society for Reproductive Health Trust, Paediatric and Perinatal Epidemiology, Paget's disease of bone, Pan American Health Organization, Pandemic, Pandemic fatigue, Pandemic Influenza Preparedness Framework, Pandemic predictions and preparations prior to the COVID-19 pandemic, Pandemic Preparedness and Response Act, Pandemic prevention, Pandemic Severity Assessment Framework, Pandemic severity index, Papaya Bunchy Top Disease, Parasitic disease, Parasitic pneumonia, Parenteral nutrition, Parkinson's disease, Partnership for Maternal, Newborn & Child Health, Pathogens and Global Health, Pay for performance (healthcare), Pelvic inflammatory disease, Personally Controlled Electronic Health Record, Pervasive developmental disorder, Pervasive developmental disorder not otherwise specified, Peyronie's disease, Philippine government response to the COVID-19 pandemic, Pinta (disease), Pinworm infection, Plague (disease), Plant nutrition, Pneumococcal infection, Pneumococcal pneumonia, Pneumocystis pneumonia, Pneumonia, Pneumonia (non-human), Pneumonia jacket, Pneumonia of unknown etiology (PUE) surveillance system, Pneumonia severity index, Pogosta disease, Population health, Population health policies and interventions, Population, health, and the environment, Portal:Pandemics, Postorgasmic illness syndrome, Pott disease, Pravastatin or atorvastatin evaluation and infection therapy - thrombolysis in myocardial infarction 22, Prebiotic (nutrition), Pregnancy-associated malaria, President's Malaria Initiative, Prevalence of mental disorders, Prevention of mental disorders, Preventive nutrition, Price-Pottenger Nutrition Foundation, Primary Health Centre (India), Prime Healthcare Services, Priority-setting in global health, Private health services plan, Program for Jewish Genetic Health, Progressive disease, Providence Health & Services, Providence St. Joseph Health, Psychiatric epidemiology, Psychogenic disease, Public health, Public Health Agency of Canada, Public Health Agency of Sweden, Public Health Emergency of International Concern, Public Health England, Public health insurance option, Public health intervention, Public health observatory, Public health policy, Public health problems in the Aral Sea region, Public Health Scotland, Public health system in India, Public Health Wales, Publicly funded

health care, Pullorum disease, Qapqal disease, Race and health, RAND Health Insurance Experiment, Rare disease, Reactive airway disease, Real-time outbreak and disease surveillance, Refugee health care in Canada, Regional Forum on Environment and Health in Southeast and East Asian Countries, Regional Health Agency, Reproductive health care for incarcerated women in the United States, Reproductive system disease, Respiratory tract infection, Rheumatoid disease of the spine, Royal Commission on the Future of Health Care in Canada, Rural health care in Australia, Russian government response to the COVID-19 pandemic, Salt and cardiovascular disease, Samaritan Health Services, Saprotrophic nutrition, SARS, SARS-CoV-2 Alpha variant, SARS-CoV-2 Delta variant, SARS-CoV-2 Gamma variant, SARS-CoV-2 Kappa variant, SARS-CoV-2 Mu variant, SARS-CoV-2 Omicron variant, SARS-CoV-2 Theta variant, Scandinavian Journal of Work, Environment & Health, School-based health centers, Science diplomacy and pandemics, Second plague pandemic, Self-rated health, Sentara Healthcare, Services for mental disorders, Sexual and reproductive health, Sexual and Reproductive Health Matters, Sexual health clinic, Sexually transmitted infection, Shona Holmes health care incident, Sickle cell disease, Single-payer healthcare, Sissel v. United States Department of Health & Human Services, Skin and skin structure infection, Skin infection, Smallpox epidemic, Social impact of the COVID-19 pandemic in Malaysia, Social Psychiatry and Psychiatric Epidemiology, Society for Family Health Nigeria, Society of Infectious Diseases Pharmacists, Socioeconomic status and mental health, South African Malaria Initiative, South Texas Center for Emerging Infectious Diseases, Southern tick-associated rash illness, Spanish National Health System, Spatial and Spatio-temporal Epidemiology, Spatial epidemiology, Specific replant disease, Sports nutrition, St. Patrick Hospital and Health Sciences Center, St. Vincent's Health System, Stateville Penitentiary Malaria Study, Statistics of the COVID-19 pandemic in Argentina, Statistics of the COVID-19 pandemic in Australia, Statistics of the COVID-19 pandemic in Bangladesh, Statistics of the COVID-19 pandemic in Brazil, Statistics of the COVID-19 pandemic in Chile, Statistics of the COVID-19 pandemic in Germany, Statistics of the COVID-19 pandemic in India, Statistics of the COVID-19 pandemic in Indonesia, Statistics of the COVID-19 pandemic in Italy, Statistics of the COVID-19 pandemic in Japan, Statistics of the COVID-19 pandemic in Malaysia, Statistics of the COVID-19 pandemic in Poland, Statistics of the COVID-19 pandemic in Portugal, Statistics of the COVID-19 pandemic in Russia, Statistics of the COVID-19 pandemic in Serbia, Statistics of the COVID-19 pandemic in Singapore, Statistics of the COVID-19 pandemic in Tamil Nadu, Statistics of the COVID-19 pandemic in Thailand, Statistics of the COVID-19 pandemic in the United Kingdom, STOP Foodborne Illness, Strengthening the reporting of observational studies in epidemiology, Streptococcus pneumoniae, Study of Health in Pomerania, Suicide epidemic, Superinfection, Susceptibility and severity of infections in pregnancy, Sutter Health, Sweating sickness epidemics, Swedish Association of Health Professionals, Swedish government response to the COVID-19 pandemic, Systemic disease, Taiwan Centers for Disease Control, Target Malaria, Template:Ascension Health, Template:Eradication of infectious disease, Template:Gram-positive actinobacteria diseases, Template:Infectious-disease-stub, Template:Malaria, Template:Pervasive developmental disorders, Template:Plant nutrition, Template:Vertically transmitted infection, Template:Women's health, Tenet Healthcare, Texas Health Huguley Hospital Fort Worth South, The European Journal of Health Economics, The Far West Baby Health Clinic Cars, The Global Fund to Fight AIDS, Tuberculosis and Malaria, The Journal of Mental Health Policy and Economics, The Medical Center, Navicent Health, The Office of Health Economics, Theiler's disease, Third plague pandemic, Tick-borne disease, Tick-Borne Disease Alliance, Timeline of global health, Timeline of peptic ulcer disease and Helicobacter pylori, Timeline of the COVID-19 pandemic in Afghanistan, Timeline of the COVID-19 pandemic in Argentina, Timeline of the COVID-19 pandemic in Australia, Timeline of the COVID-19 pandemic in Bangladesh, Timeline of the COVID-19 pandemic in Brazil, Timeline of the COVID-19 pandemic in Canada, Timeline of the COVID-19 pandemic in Croatia, Timeline of the COVID-19 pandemic in Fiji, Timeline of the COVID-19 pandemic in Ghana, Timeline of the COVID-19 pandemic in India, Timeline of the COVID-19 pandemic in India (2021), Timeline of the COVID-19 pandemic in Indonesia, Timeline of the COVID-19 pandemic in Indonesia (2020), Timeline of the COVID-19 pandemic in Indonesia (2021), Timeline of the COVID-19 pandemic in Italy, Timeline of the COVID-19 pandemic in Ivory Coast, Timeline of the COVID-19 pandemic in Japan, Timeline of the COVID-19 pandemic in Malaysia, Timeline of the COVID-19 pandemic in Malta, Timeline of the COVID-19 pandemic in Mexico, Timeline of the COVID-19 pandemic in Nepal, Timeline of the COVID-19 pandemic in New Zealand, Timeline of the COVID-19 pandemic in Pakistan, Timeline of the COVID-19 pandemic in Romania, Timeline of the COVID-19 pandemic in Russia, Timeline of the COVID-19 pandemic in Serbia, Timeline of the COVID-19 pandemic in Singapore, Timeline of the COVID-19 pandemic in Spain, Timeline of the COVID-19 pandemic in Sweden, Timeline of the COVID-19 pandemic in Thailand, Timeline of the COVID-19 pandemic in the Philippines, Timeline of the COVID-19 pandemic in the Republic of Ireland, Timeline of the COVID-19 pandemic in the United Kingdom, Timeline of

the COVID-19 pandemic in the United States, Timeline of the COVID-19 pandemic in Trinidad and Tobago, Timeline of the COVID-19 pandemic in Turkey, Timeline of the COVID-19 pandemic in Uruguay, Timeline of the COVID-19 pandemic in Vietnam, Timeline of the SARS-CoV-2 Omicron variant, Top dying disease, Trauma model of mental disorders, Treatment of mental disorders, Tropical disease, Typhus epidemic in Goose Village, Montreal, UCLA Health, UCLA Health Training Center, UCSC Malaria Genome Browser, UK Health Alliance on Climate Change, UK Health Security Agency, Undernutrition in children, Uni Health, Unicentric Castleman disease, UnityPoint Health, UnityPoint Health - Allen Hospital, Universal Declaration on the Eradication of Hunger and Malnutrition, University of Edinburgh School of Health in Social Science, Ureaplasma urealyticum infection, Use of technology in treatment of mental disorders, Usual interstitial pneumonia, Vaccine-preventable diseases, Value-based health care, Vanguard Health Systems, Variant Creutzfeldt–Jakob disease, Vegetarian nutrition, Venereal Disease Research Laboratory test, Ventilator-associated pneumonia, Vermont health care reform, Vertically transmitted infection, Very early onset inflammatory bowel disease, Victorian Health Promotion Foundation, Vietnamese government response to the COVID-19 pandemic, Viral disease, Viral disease testing, Viral pneumonia, Virgin soil epidemic, Waterborne disease and climate change, Waterborne diseases, Weather and climate effects on Lyme disease exposure, Western African Ebola virus epidemic, Whitecoat Health Service Directory, Whole Health Action Management, Wilt disease, Women's health, Women's health movement in the United States, Working Environment (Air Pollution, Noise and Vibration) Convention, 1977, Workplace health promotion, World Chagas Disease Day, World Health Assembly, World Health Organization, World Health Organization Composite International Diagnostic Interview, World Malaria Day, World Pneumonia Day, XMEN disease, Your Health Idaho

List of English Wikipedia articles used to track public engagement in the COVID-19 pandemic

Boroughs of Montreal during the COVID-19 pandemic, British government response to the COVID-19 pandemic, Chloroquine and hydroxychloroquine during the COVID-19 pandemic, Coronavirus disease 2019, Coronavirus diseases, COVID-19 pandemic, COVID-19 pandemic by country and territory, COVID-19 pandemic cases, COVID-19 pandemic death rates by country, COVID-19 pandemic deaths, COVID-19 pandemic in Abkhazia, COVID-19 pandemic in Afghanistan, COVID-19 pandemic in Akrotiri and Dhekelia, COVID-19 pandemic in Albania, COVID-19 pandemic in Algeria, COVID-19 pandemic in American Samoa, COVID-19 pandemic in Andorra, COVID-19 pandemic in Angola, COVID-19 pandemic in Antigua and Barbuda, COVID-19 pandemic in Argentina, COVID-19 pandemic in Armenia, COVID-19 pandemic in Australia, COVID-19 pandemic in Austria, COVID-19 pandemic in Azerbaijan, COVID-19 pandemic in Bahrain, COVID-19 pandemic in Bangladesh, COVID-19 pandemic in Barbados, COVID-19 pandemic in Belarus, COVID-19 pandemic in Belgium, COVID-19 pandemic in Belize, COVID-19 pandemic in Benin, COVID-19 pandemic in Bhutan, COVID-19 pandemic in Bolivia, COVID-19 pandemic in Bosnia and Herzegovina, COVID-19 pandemic in Botswana, COVID-19 pandemic in Brazil, COVID-19 pandemic in Brunei, COVID-19 pandemic in Bulgaria, COVID-19 pandemic in Burkina Faso, COVID-19 pandemic in Burundi, COVID-19 pandemic in Cambodia, COVID-19 pandemic in Cameroon, COVID-19 pandemic in Canada, COVID-19 pandemic in Cape Verde, COVID-19 pandemic in Chad, COVID-19 pandemic in Chile, COVID-19 pandemic in Colombia, COVID-19 pandemic in Costa Rica, COVID-19 pandemic in Crimea, COVID-19 pandemic in Croatia, COVID-19 pandemic in Cuba, COVID-19 pandemic in Cyprus, COVID-19 pandemic in Denmark, COVID-19 pandemic in Djibouti, COVID-19 pandemic in Dominica, COVID-19 pandemic in East Timor, COVID-19 pandemic in Easter Island, COVID-19 pandemic in Ecuador, COVID-19 pandemic in Egypt, COVID-19 pandemic in El Salvador, COVID-19 pandemic in England, COVID-19 pandemic in Equatorial Guinea, COVID-19 pandemic in Eritrea, COVID-19 pandemic in Estonia, COVID-19 pandemic in Eswatini, COVID-19 pandemic in Ethiopia, COVID-19 pandemic in Europe, COVID-19 pandemic in Fiji, COVID-19 pandemic in Finland, COVID-19 pandemic in France, COVID-19 pandemic in French Polynesia, COVID-19 pandemic in Gabon, COVID-19 pandemic in Gagauzia, COVID-19 pandemic in Georgia (country), COVID-19 pandemic in Germany, COVID-19 pandemic in Ghana, COVID-19 pandemic in Gibraltar, COVID-19 pandemic in Greece, COVID-19 pandemic in Greenland, COVID-19 pandemic in Grenada, COVID-19 pandemic in Guatemala, COVID-19 pandemic in Guernsey, COVID-19 pandemic in Guinea, COVID-19 pandemic in Guinea-Bissau, COVID-19 pandemic in Guyana, COVID-19 pandemic in Haiti, COVID-19 pandemic in Honduras, COVID-19 pandemic in Hungary, COVID-19 pandemic in Iceland, COVID-19 pandemic in India, COVID-19 pandemic in Indonesia, COVID-19 pandemic in Iran, COVID-19 pandemic in Iraq, COVID-19 pandemic in Israel, COVID-19 pandemic in Italy, COVID-19 pandemic in Ivory Coast, COVID-19 pandemic in Jamaica, COVID-19 pandemic in Japan, COVID-19 pandemic in Jersey, COVID-19 pandemic in Jordan, COVID-19 pandemic in Kazakhstan, COVID-19 pandemic in Kenya, COVID-

19 pandemic in Kosovo, COVID-19 pandemic in Kuwait, COVID-19 pandemic in Kyrgyzstan, COVID-19 pandemic in Laos, COVID-19 pandemic in Latvia, COVID-19 pandemic in Lebanon, COVID-19 pandemic in Libya, COVID-19 pandemic in Liechtenstein, COVID-19 pandemic in Lithuania, COVID-19 pandemic in Luxembourg, COVID-19 pandemic in mainland China, COVID-19 pandemic in Malawi, COVID-19 pandemic in Malaysia, COVID-19 pandemic in Mali, COVID-19 pandemic in Malta, COVID-19 pandemic in Moldova, COVID-19 pandemic in Monaco, COVID-19 pandemic in Montenegro, COVID-19 pandemic in New Caledonia, COVID-19 pandemic in North Asia, COVID-19 pandemic in North Macedonia, COVID-19 pandemic in Northern Cyprus, COVID-19 pandemic in Northern Ireland, COVID-19 pandemic in Norway, COVID-19 pandemic in Poland, COVID-19 pandemic in Portugal, COVID-19 pandemic in Romania, COVID-19 pandemic in Russia, COVID-19 pandemic in San Marino, COVID-19 pandemic in Scotland, COVID-19 pandemic in Serbia, COVID-19 pandemic in Sevastopol, COVID-19 pandemic in Slovakia, COVID-19 pandemic in Slovenia, COVID-19 pandemic in South Ossetia, COVID-19 pandemic in Spain, COVID-19 pandemic in Sweden, COVID-19 pandemic in Switzerland, COVID-19 pandemic in the Åland Islands, COVID-19 pandemic in the Bahamas, COVID-19 pandemic in the Central African Republic, COVID-19 pandemic in the Comoros, COVID-19 pandemic in the Czech Republic, COVID-19 pandemic in the Democratic Republic of the Congo, COVID-19 pandemic in the Dominican Republic, COVID-19 pandemic in the Donetsk People's Republic, COVID-19 pandemic in the European Union, COVID-19 pandemic in the Faroe Islands, COVID-19 pandemic in the Federated States of Micronesia, COVID-19 pandemic in the Gambia, COVID-19 pandemic in the Guantanamo Bay Naval Base, COVID-19 pandemic in the Isle of Man, COVID-19 pandemic in the Kurdistan Region, COVID-19 pandemic in the Luhansk People's Republic, COVID-19 pandemic in the Netherlands, COVID-19 pandemic in the Regional Municipality of Peel, COVID-19 pandemic in the Republic of Artsakh, COVID-19 pandemic in the Republic of Ireland, COVID-19 pandemic in the Republic of the Congo, COVID-19 pandemic in the United Kingdom, COVID-19 pandemic in Transnistria, COVID-19 pandemic in Turkey, COVID-19 pandemic in Ukraine, COVID-19 pandemic in Vatican City, COVID-19 pandemic in Wales, COVID-19 pandemic in Wallis and Futuna, COVID-19 pandemic on Charles de Gaulle, COVID-19 pandemic on Diamond Princess, COVID-19 Pandemic Unemployment Payment, Economic impact of the COVID-19 pandemic in Russia, Economic impact of the COVID-19 pandemic in the Republic of Ireland, European Union response to the COVID-19 pandemic, Evacuations by India related to the COVID-19 pandemic, Face masks during the COVID-19 pandemic, Federal aid during the COVID-19 pandemic in Canada, Food security during the COVID-19 pandemic, Ghanaian government response to the COVID-19 pandemic, Glossary of the COVID-19 pandemic, Human rights issues related to the COVID-19 pandemic, Impact of the COVID-19 pandemic on education in Ghana, Impact of the COVID-19 pandemic on education in the Republic of Ireland, Impact of the COVID-19 pandemic on education in the United Kingdom, Impact of the COVID-19 pandemic on Gaelic games, Impact of the COVID-19 pandemic on human rights in Argentina, Indian government response to the COVID-19 pandemic, Indian migrant workers during the COVID-19 pandemic, Indian state government responses to the COVID-19 pandemic, International reactions to the COVID-19 pandemic in Italy, Media coverage of the COVID-19 pandemic, Mental health during the COVID-19 pandemic, Middle East respiratory syndrome–related coronavirus, Moldovan–Romanian collaboration during the COVID-19 pandemic, Pandemic predictions and preparations prior to the COVID-19 pandemic, Political impact of the COVID-19 pandemic in Russia, Russian government responses to the COVID-19 pandemic, Severe acute respiratory syndrome coronavirus, Severe acute respiratory syndrome coronavirus 2, Social impact of the COVID-19 pandemic in Russia, Statistics of the COVID-19 pandemic in Argentina, Statistics of the COVID-19 pandemic in Australia, Statistics of the COVID-19 pandemic in Brazil, Statistics of the COVID-19 pandemic in Germany, Statistics of the COVID-19 pandemic in India, Statistics of the COVID-19 pandemic in Indonesia, Statistics of the COVID-19 pandemic in Italy, Statistics of the COVID-19 pandemic in Japan, Statistics of the COVID-19 pandemic in Malaysia, Statistics of the COVID-19 pandemic in Poland, Statistics of the COVID-19 pandemic in Portugal, Statistics of the COVID-19 pandemic in Russia, Statistics of the COVID-19 pandemic in Scotland, Statistics of the COVID-19 pandemic in Tamil Nadu, Statistics of the COVID-19 pandemic in the United Kingdom, Swedish government response to the COVID-19 pandemic, Template:COVID-19 pandemic data/Bangladesh medical cases by division, Template:COVID-19 pandemic in the Republic of Ireland, Timeline of the COVID-19 pandemic in Afghanistan, Timeline of the COVID-19 pandemic in Argentina, Timeline of the COVID-19 pandemic in Australia, Timeline of the COVID-19 pandemic in Bangladesh, Timeline of the COVID-19 pandemic in Belarus, Timeline of the COVID-19 pandemic in Brazil, Timeline of the COVID-19 pandemic in Canada, Timeline of the COVID-19 pandemic in Croatia, Timeline of the COVID-19 pandemic in Ghana, Timeline of the COVID-19 pandemic in India, Timeline of the COVID-19 pandemic in India (January–May 2020), Timeline of the COVID-19 pandemic in India (June–December 2020), Timeline of the COVID-19

pandemic in Indonesia, Timeline of the COVID-19 pandemic in Italy, Timeline of the COVID-19 pandemic in Japan, Timeline of the COVID-19 pandemic in Malaysia, Timeline of the COVID-19 pandemic in Mexico, Timeline of the COVID-19 pandemic in Nepal, Timeline of the COVID-19 pandemic in New Zealand, Timeline of the COVID-19 pandemic in Pakistan, Timeline of the COVID-19 pandemic in Romania, Timeline of the COVID-19 pandemic in Russia, Timeline of the COVID-19 pandemic in Singapore, Timeline of the COVID-19 pandemic in Spain, Timeline of the COVID-19 pandemic in Sweden, Timeline of the COVID-19 pandemic in Thailand, Timeline of the COVID-19 pandemic in the Philippines, Timeline of the COVID-19 pandemic in the Republic of Ireland, Timeline of the COVID-19 pandemic in the Republic of Ireland (2021), Timeline of the COVID-19 pandemic in the Republic of Ireland (January–June 2020), Timeline of the COVID-19 pandemic in the Republic of Ireland (July–December 2020), Timeline of the COVID-19 pandemic in the United Kingdom, Timeline of the COVID-19 pandemic in the United States, Timeline of the COVID-19 pandemic in Turkey, Timeline of the COVID-19 pandemic in Uruguay, Timeline of the COVID-19 pandemic in Vietnam, Timeline of the COVID-19 pandemic Nigeria, UK Coronavirus Cancer Monitoring Project

Additional analyses

Complementing the analysis presented in the 2022 Countdown report, the Figures below provide additional evidence on dynamics in pageviews and co-click networks (Figures 98 to 110).

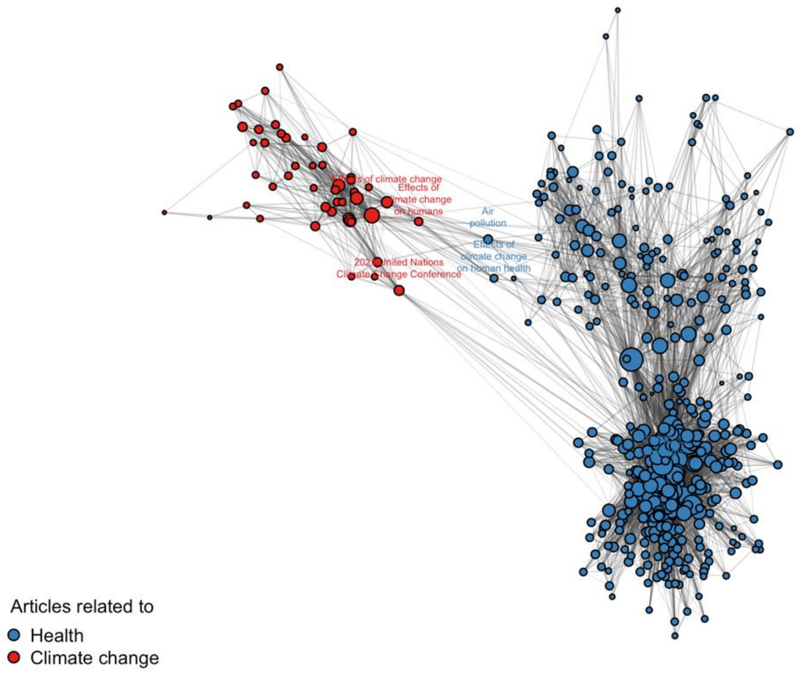

Figure 98: Connectivity graph of Wikipedia articles on climate change (red) and health (blue). Popularity of articles displayed by node size. Edges represent co-visits in the 2020 clickstream data.

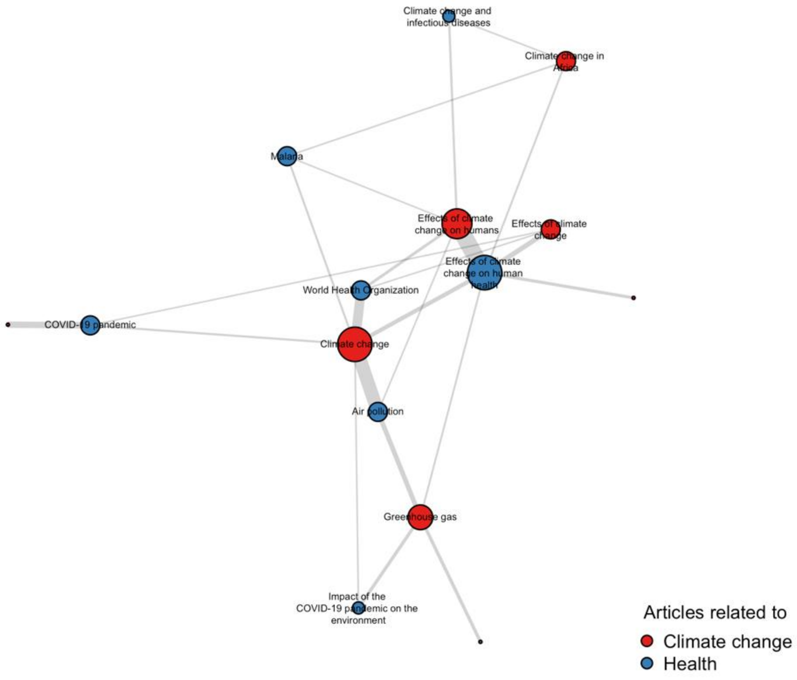

Figure 99: Connectivity graph of Wikipedia articles on climate change (red) and health (blue), filtered to co-click activity between the two domains. Popularity of articles displayed by node size. Edges represent co-visits in the 2020 clickstream data.

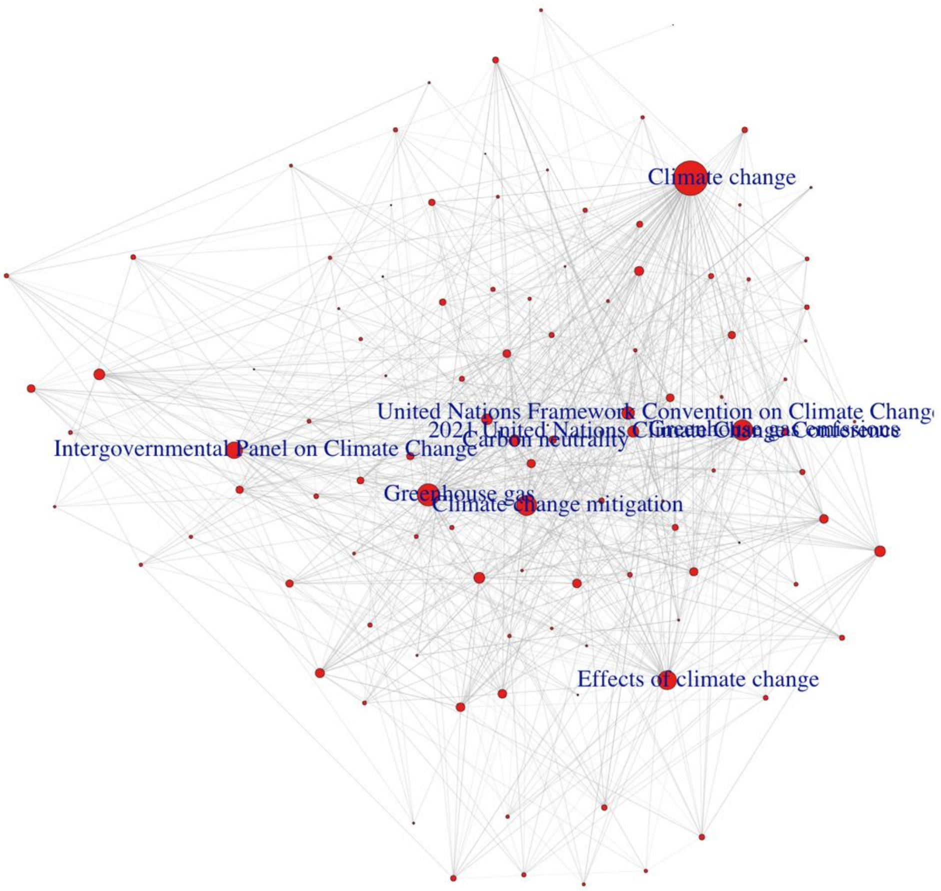

Figure 100: Connectivity graph of Wikipedia articles on climate change. Popularity of articles displayed by node size. Edges represent co-visits in the 2021 clickstream data.

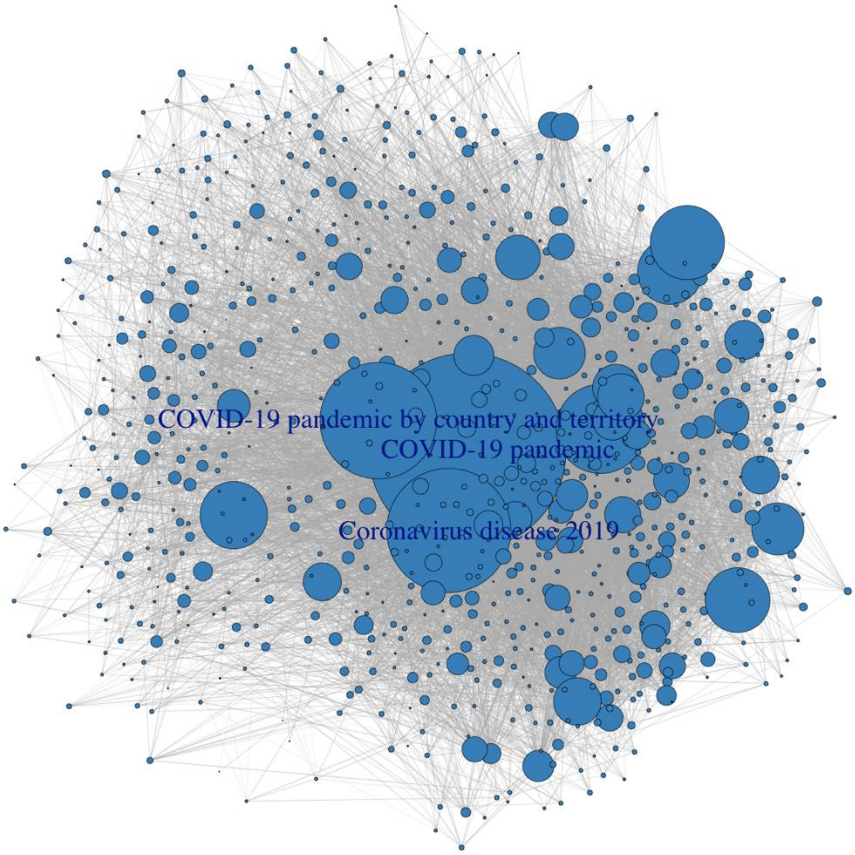

Figure 101: Connectivity graph of Wikipedia articles on health. Popularity of articles displayed by node size. Edges represent co-visits in the 2021 clickstream data.

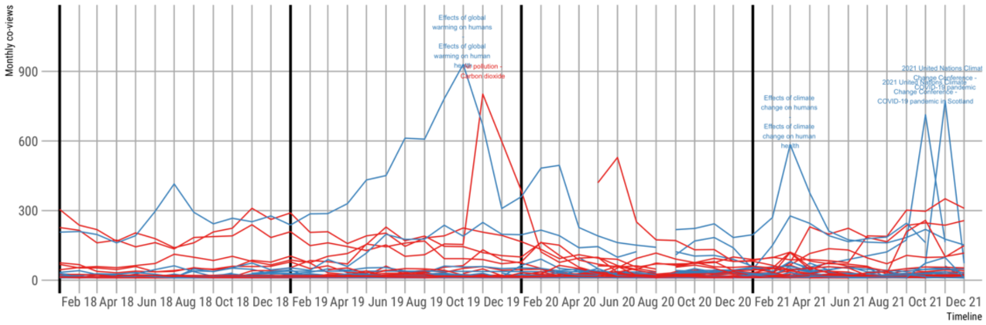

Figure 102: Co-views of climate change-health article pairs over time, 2018–2021. Dominant pairs labelled.

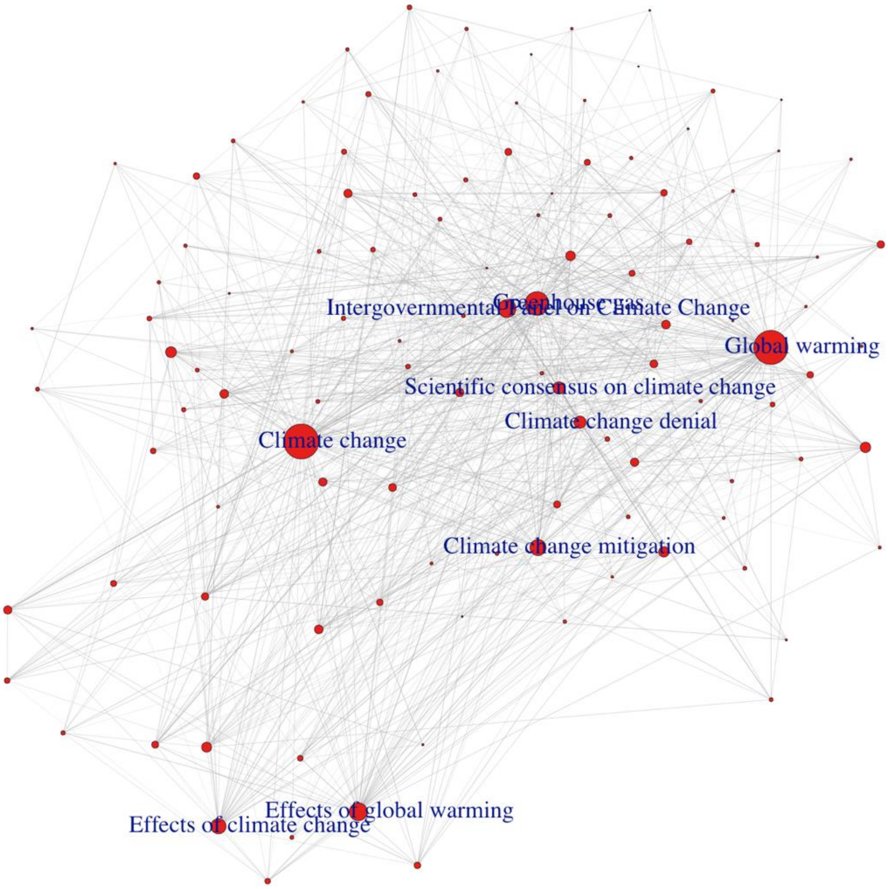

Figure 103: Connectivity graph of Wikipedia articles on climate change. Popularity of articles displayed by node size. Edges represent co-visits in the 2020 clickstream data.

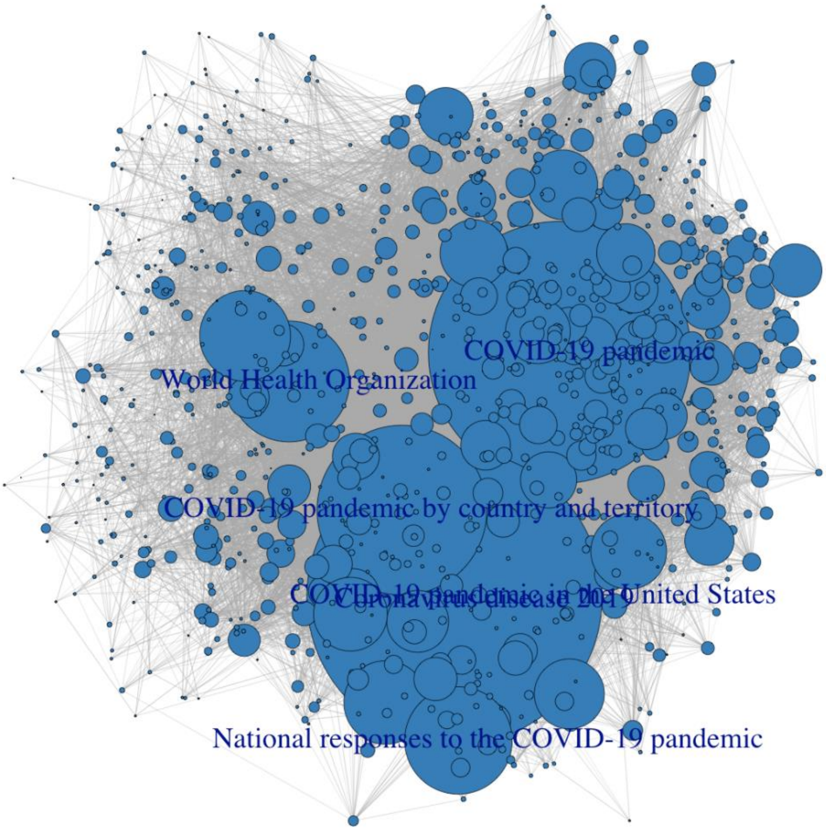

Figure 104: Connectivity graph of Wikipedia articles on health. Popularity of articles displayed by node size. Edges represent co-visits in the 2020 clickstream data.

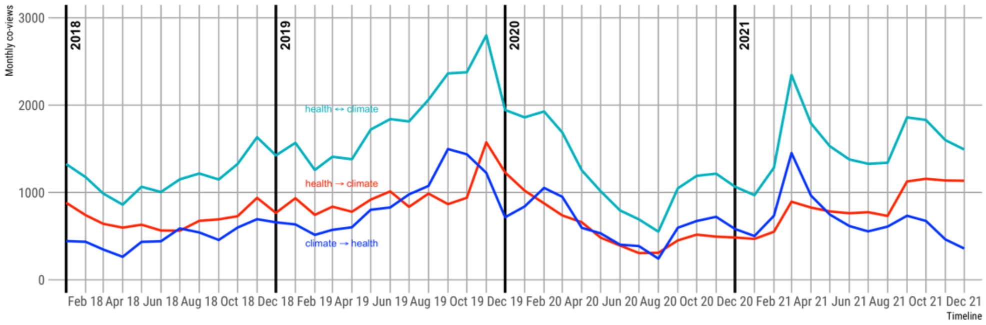

Figure 105: Aggregate monthly co-views of articles related to human health and climate change, 2018–2021 (excluding COVID-19 related articles).

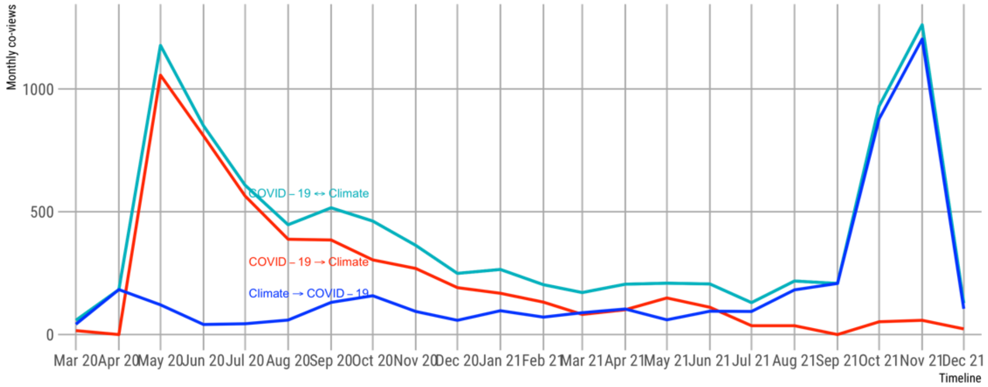

Figure 106: Aggregate monthly co-views of articles related to COVID-19 and climate change, 2021.

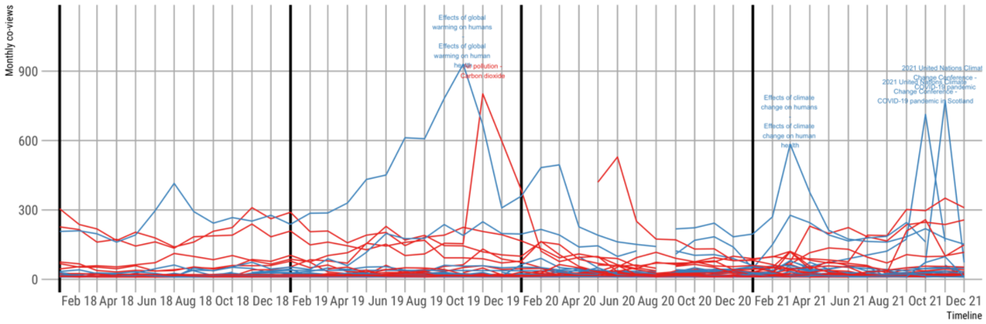

Figure 107: Co-views of climate change-health article pairs over time, 2018–2020. Dominant pairs labelled.

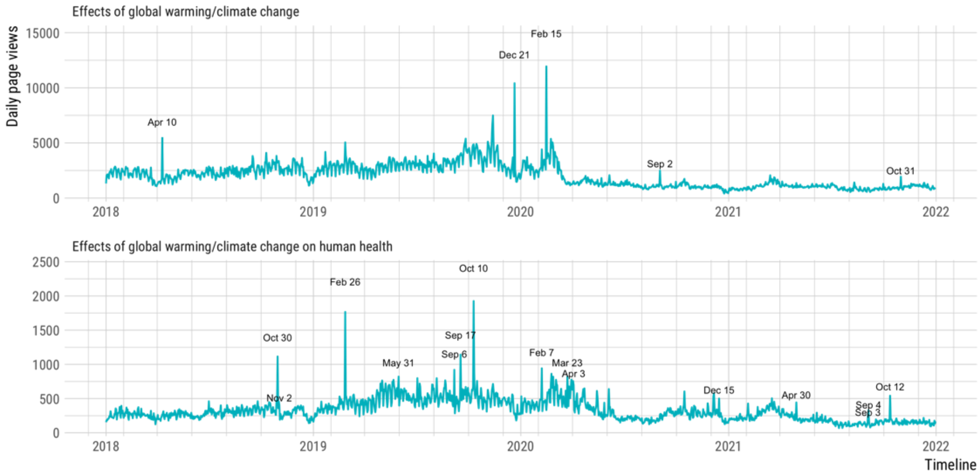

Figure 108: Daily page views 2018 to 2021 for Wikipedia articles directly related to the effects of climate change in general and on human health.

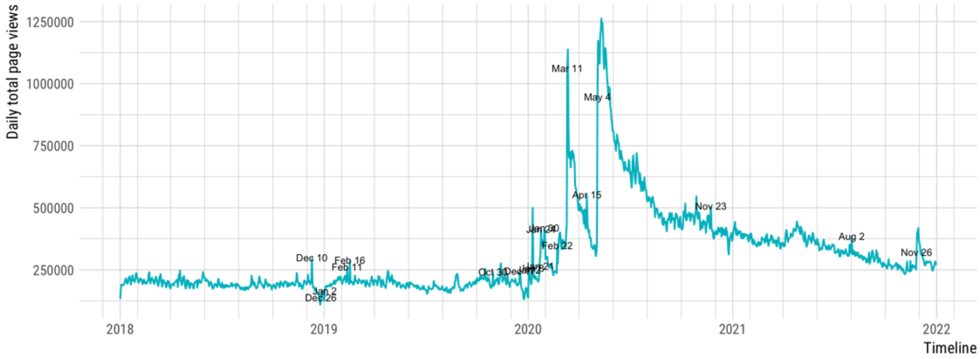

Figure 109: Aggregate daily page views 2018 to 2021 for all 1,414 selected articles on the English Wikipedia related to health.

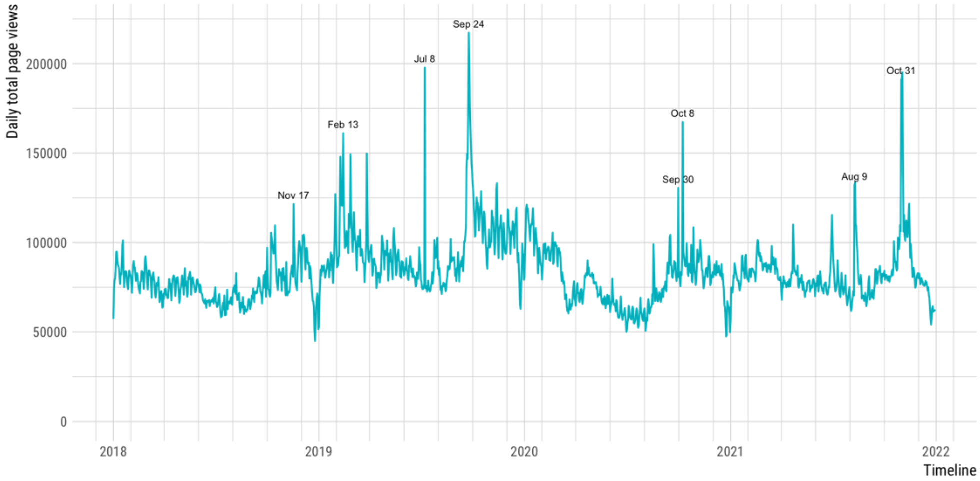

Figure 110: Aggregate daily page views 2018 to 2021 for all 610 selected articles on the English Wikipedia related to climate change.

5.3. Scientific Engagement in Health and Climate Change

Method

Scientific engagement in health and climate change is central to the *Lancet* Countdown mission: this is to facilitate, support and track progress on health and climate change. Scientific evidence is the major resource on which such progress rests; it also informs engagement in the key domains of global action, including the public, governmental and corporate domains. This indicator quantifies engagement in the topic of climate change and health by tracking the number of publications over time. A machine-learning approach allows for a more granular picture of the research landscape, including developments across major domains of research (mitigation, adaptation, impacts), the health impacts covered, locations studied, as well as patterns of authorship.

This iteration sees an update to this indicator, building on earlier methodologies, including standard scoping review methods[370-372]. The approach outlined here retains the indicator but relies on less resource-intensive and scalable methods by leveraging machine-learning approaches. The use of search strategies within scientific databases and subsequent data extraction techniques is common within the literature.[370-372] Using machine-learning-assisted review methodologies means that many potentially relevant documents can be automatically screened, greatly reducing the resources required to identify documents likely to be relevant. This means that a larger pool of potentially relevant documents can be searched. Given the exponentially increasing volume of relevant literature that is no longer amenable to regularly updated manual synthesis, this represents a cutting-edge approach, and is the best methodology available to track this indicator. The use of topic modelling means that the relative share of specific topics within these fields – and how these proportions change over time – can be analysed. This also enables the visualisation of trends over time and geographical space, given the information stored for each article.

A revised search strategy and database selection was scoped, developed and piloted, to enable tracking of scientific coverage of health and climate change. It is this revised methodology that is presented here, though methods are detailed in a peer-reviewed methods protocol in Wellcome Open Research, 'Mapping global research on climate and health using machine learning (a systematic protocol)', available at: https://doi.org/10.12688/wellcomeopenres.16415.1, and in a comprehensive study protocol.[373]

The objective for this indicator is to systematically track the evidence on the relationship between climate change, climate variability, and weather (CCVW) and human health globally. Such a broad framing is necessary to adequately represent the relevant scientific work in such a diverse field, where not all relevant work involves formal climate change attribution. The evidence map is framed using a PICoST approach: population/problem (P), interest (I), context (Co), and scope and time (S/T). The scope is global, interest is in empirical evidence on the relationships between climate change, climate variability, as well as weather (CCVW) and human health, and coverage includes any scientific article or review covered by bibliographic records in the Web of Science, Scopus or MEDLINE. Interest is in any context, i.e., any component of the nexus between climate change, climate variability, and weather (CCVW) and human health, including impacts on health, and responses to reduce health impacts from climate change (e.g. adaptation, mitigation), without prejudice to any climate-health pathway. This search as well as inclusion and exclusion criteria are framed within this PICoST framework.

The analysis searches titles, abstracts and keywords, focuses on English-only search terms, and does not restrict time coverage. Included documents must:

(a) Provide a clear link to actual, projected, or perceived impacts of climate change, responses to reduce the impacts of climate change (adaptation), or the mitigation of greenhouse gas emissions. Evidence of detection and attribution was not required.
(b) Include substantial focus on a perceived, experienced, or observed eligible health-related outcome or health system.
(c) Present empirically-driven research or review (including non-systematic reviews) of such research.

Each year, new documents will be downloaded, and those predicted to be relevant by the machine learning algorithm will be included in the indicator. The initial broad literature search yielded a total of approximately 350,000 unique records for the period between 2013 and 2020, which was too large to screen entirely by hand, and even less feasible to update regularly. A sample of 3730 documents was retrieved and hand-screened for inclusion, with each document labelled according to three major climate change research domains (climate

impacts, climate change mitigation and climate change adaptation). This was used to train a machine-learning algorithm which predicted the relevance of documents with an accuracy of 87.1% (i.e., 87% of machine-generated inclusion/exclusion decisions matched those made by hand). This classifier was used to predict the relevance of remaining unscreened documents and classify them according to climate change research domains. Based on this procedure, the headline indicator can be derived.

Once papers were downloaded from the literature databases and were included or excluded based on their predicted relevance value (see above), the number of papers published was counted. This constitutes the core of the indicator and measures the amount of literature focusing on climate and health. Each of the publications was assigned to one of three major domains of climate research using an active learning approach: climate change impacts, climate change mitigation and climate change adaptation.

In addition to this, topic modelling, an unsupervised machine learning technique, was used to discover thematic labels from the article abstracts, assigning these to the documents automatically. Topics were grouped under health risks and impacts, hazards, adaptation options, and mitigating pathways. Geographic locations were also extracted from study texts using a pretrained named entity recognition pipeline.

Data

This indicator uses data from bibliographic records in the online scientific databases Web of Science, Scopus or MEDLINE.

Caveats

Only English-language search terms are used within this protocol. Any language bias is limited, however, given that key international scientific platforms index all papers (including non-English papers) with keywords, title, and abstract in English translation. The protocol uses title, keywords, and abstract to retrieve and assess the scientific literature, meaning that non-English papers can still be retrieved as long as they are indexed in English.

The methodology provided here enables a quantitative appraisal of the research question. The use of machine learning means that there will be some uncertainty as to the number of relevant documents. Further, the quality of the individual studies and the specifics of their content will not be assessed by the indicator team. However, with the outputs all published in peer-reviewed journals, there is a de facto check on quality. For this reason, the indicator does not cover grey literature.

Focusing on peer-reviewed literature will mean some scientific work of interest is missed, particularly from the grey literature. Systematic search of grey literature is challenging, but future work could develop web-crawling approaches to find relevant reports and other publications outside of the peer reviewed literature.

Additional information

The growth of scientific publications investigating the intersection between health and climate change. Average annual growth in publications between 2000 and 2021 was 18%. In 2021, about 3,214 relevant articles were published, an increase of 22% compared to 2020 (Figure 111).

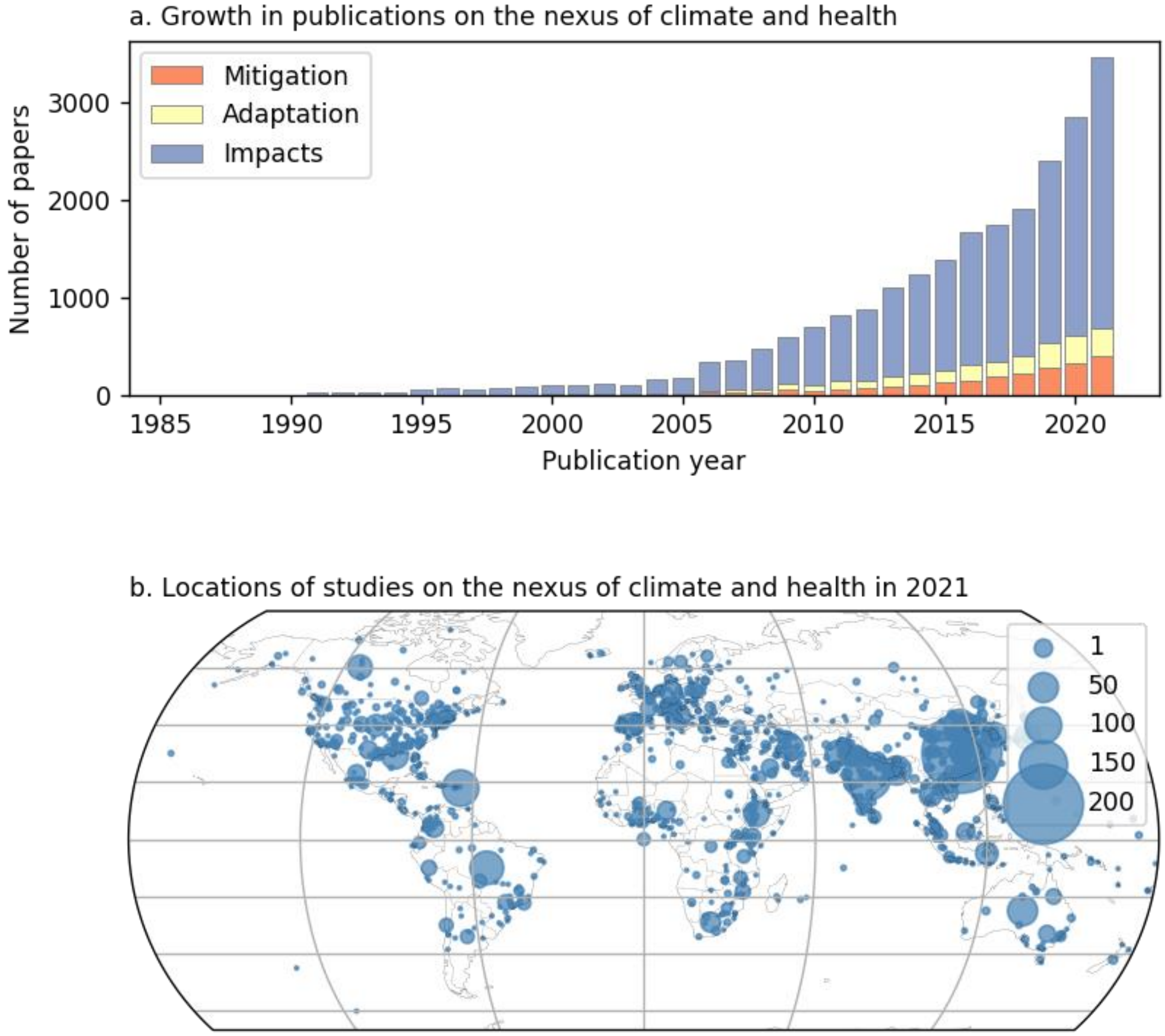

Figure 111: Growth in publications on the nexus of climate and health.

Figure 112 shows the location of studies focusing on both climate and health. The WHO region most commonly mentioned – where a geographic location could be extracted from the study title and abstract – was Western Pacific, with 866 unique studies in 2021, as depicted in Figure 113. Americas was the next most common region with 655, followed by Europe (409), South-East Asia (369), Africa (237), and Eastern Mediterranean (216).

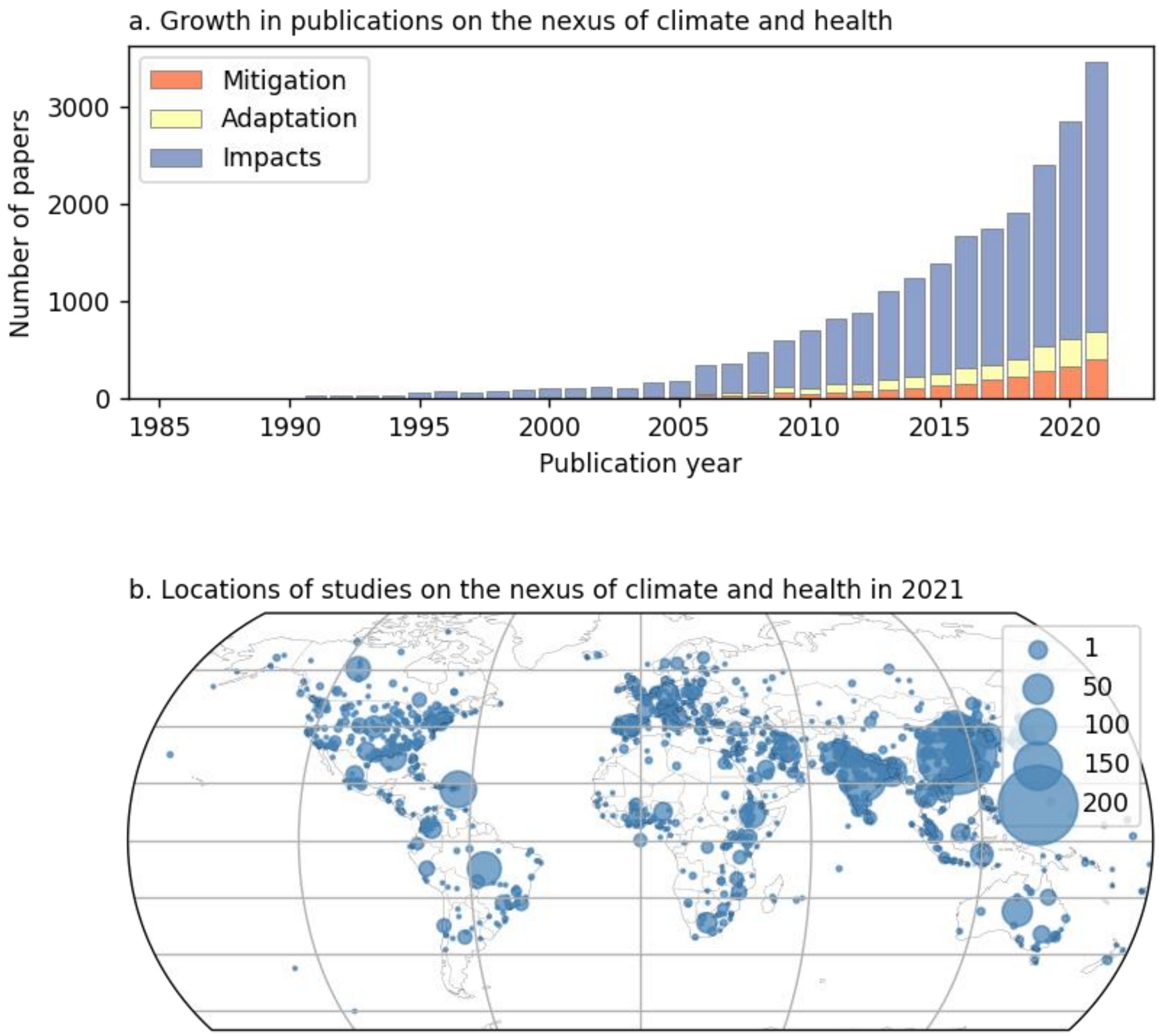

Figure 112: Locations of studies on the nexus of climate and health in 2021.

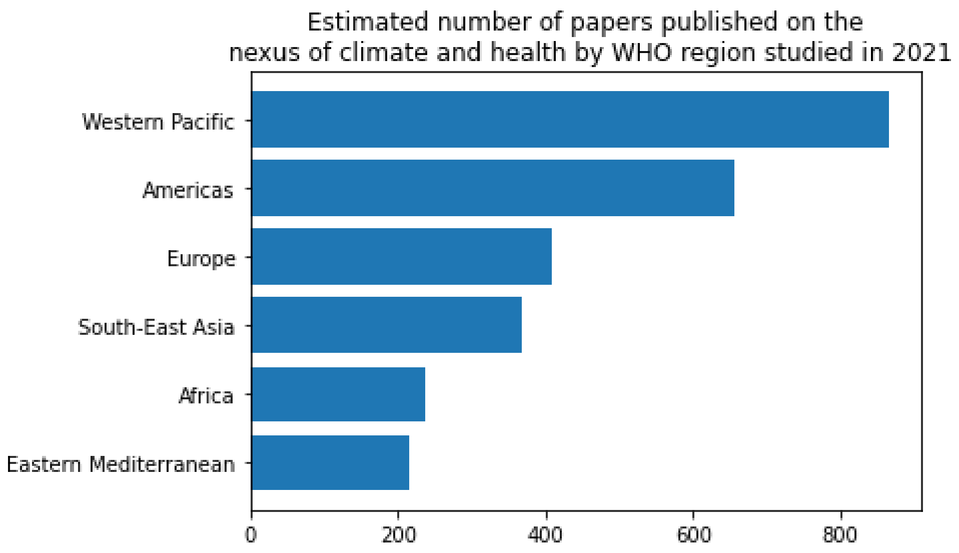

Figure 113: Estimated number of papers published on the nexus of climate and health by WHO region studied in 2021.

Counting the number of studies with an author affiliation in each region, the same order of regions prevailed, though with a larger gap between the top 3 regions of Western Pacific, Americas, and Europe (1,030, 914, and 845 studies respectively), and South-East Asia, Africa, and Eastern Mediterranean (383, 218, and 183 studies respectively) (Figure 114). The majority of new papers concerned - in line with previous years - the health implications of climate change impacts (79%) of all relevant documents. Mitigation and adaptation accounted for 12% and 10% of new relevant documents respectively. However, over the last ten years there has been a pronounced increase in attention given to such research on climate solutions.

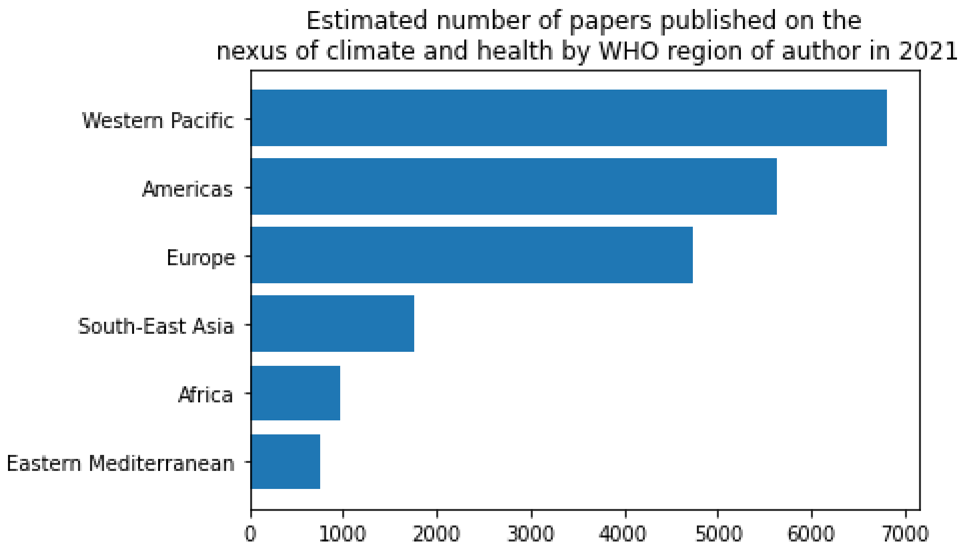

Figure 114: Estimated number of papers published on the nexus of climate and health by WHO region of author studied in 2021.

Of the 22,054 studies on climate change and health in the dataset, nearly 3,000 mentioned adaptation, while 1,800 mentioned pandemics (Figure 115). Only 108 documents mentioned the overlap between adaptation and pandemics.

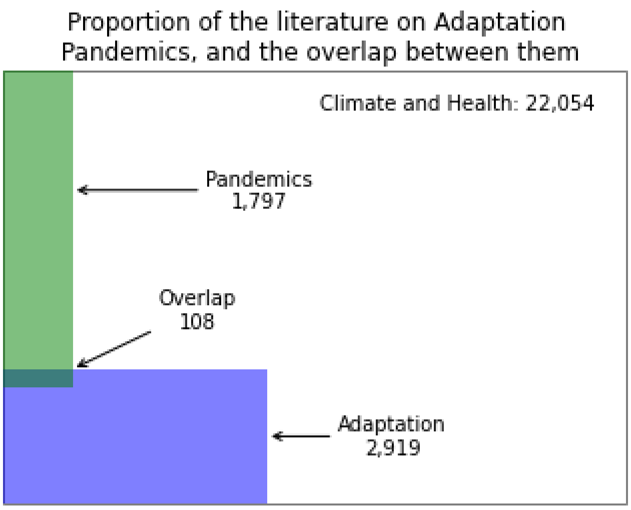

Figure 115: Proportion of the literature on adaptation, pandemics, and their overlap.

Future form of indicator

In the future, once the steps toward collecting the data and running the analysis have been automated, it is proposed to develop a 'living' evidence map, that could show the latest developments in the literature as part of the *Lancet* Countdown Data Platform. Fortnightly updates would mean that the data displayed were as up-to-date as possible.

Restriction to English-language articles will also result in missed articles. Multilingual search and classification present significant challenges, but approaches using automatic translation may be investigated. There is also scope to formulate add-ons to the indicator, for example focusing on trends in scientific coverage of particular climate-sensitive health outcomes and/or regions

5.4. Government Engagement in Health and Climate Change

5.4.1. Engagement in Health and Climate Change in the United Nations General Assembly

To produce the measure of high-level political engagement with climate change and health in the UN General Assembly, a new dataset of UN General Debate statements was used, which is discussed below. This approach to using UNGD statements to produce the indicators is based on the application of natural language processing to the corpus of UNGD statements. It identifies references to key search terms linked to (a) health, and (b) climate change (Table 69).

Health terms	Climate change terms
• malaria	• climate change
• diarrhoea	• changing climate
• infection	• climate emergency
• disease	• climate action
• diseases	• climate crisis
• sars	• climate decay
• measles	• global warming
• pneumonia	• green house

• epidemic<br>• epidemics<br>• pandemic<br>• pandemics<br>• epidemiology<br>• healthcare<br>• health<br>• mortality<br>• morbidity<br>• nutrition<br>• illness<br>• illnesses<br>• ncd<br>• ncds<br>• air pollution<br>• nutrition<br>• malnutrition<br>• malnourishment<br>• mental disorder<br>• mental disorders<br>• stunting	• temperature<br>• extreme weather<br>• global environmental change<br>• climate variability<br>• greenhouse<br>• greenhouse-gas<br>• low carbon<br>• ghge<br>• ghges<br>• renewable energy<br>• carbon emission<br>• carbon emissions<br>• carbon dioxide<br>• carbon-dioxide<br>• co2 emission<br>• co2 emissions<br>• climate pollutant<br>• climate pollutants<br>• decarbonization<br>• decarbonisation<br>• carbon neutral<br>• carbon-neutral<br>• carbon neutrality<br>• climate neutrality<br>• net-zero<br>• net zero

Table 69: UN General Debate statement reference key search terms

These key terms have been updated to reflect the changing terminology used to discuss climate change. To produce an indicator of engagement with the intersection of climate change and health, this approach focused on whether any of the climate change related terms appeared immediately before or after any health terms in the GD statements. This was based on a search of the 25 words before and after a reference to a health-related term. The choice of 25-word window context corresponds to approximately half a paragraph of text. Given that UNGD statements are highly structured and methodically developed by governments over prolonged periods of time, it is assumed that half a paragraph of text around public health terms captures a sufficiently narrow context. The number of climate change term references were counted in these contexts to produce the measure of engagement with the link between health and climate change. A robustness analysis — varying the size of the context (5, 10, and 50 words) — was also undertaken. This substantively produced the same trends over time. A sample of the references produced by the search were also examined as an additional check to ensure that the references identified reflect engagement with the health impacts of climate change.

Data

This indicator draws on a new and updated dataset of GD statements: *the United Nations General Debate corpus*, in which the annual GD statements have been pre-processed and prepared for the application of natural language processing to the official English versions of the statements.[374] The dataset contains all the country speeches made in the UN General Debate between 1970 and 2021. Table 70 presents summary of the data by year.

Year	General Debate statements	Total sentences	Total words
1970	70	11854	303791
1971	116	19901	508506
1972	125	21201	540994
1973	120	21450	536413
1974	129	22041	568739
1975	126	21365	534375
1976	134	23799	599949
1977	140	24799	606549
1978	141	25236	626163
1979	144	26462	654000
1980	149	27191	659225
1981	145	26063	633579
1982	147	23435	638691
1983	149	26803	643068
1984	150	27928	662654
1985	137	19258	592666
1986	149	19030	577525
1987	152	18336	563132
1988	154	18595	569493
1989	153	19440	574379
1990	156	17885	522197
1991	162	18552	538351
1992	167	18597	543138
1993	175	20165	587448
1994	178	19944	580530
1995	172	17870	536741
1996	181	18046	522699
1997	176	17701	514492
1998	181	18883	514836

1999	181	18529	531306
2000	178	16259	464312
2001	189	14748	414683
2002	188	13977	380481
2003	189	14716	399397
2004	192	14899	405290
2005	185	13012	353065
2006	193	14646	390476
2007	191	14586	387883
2008	192	14294	384881
2009	193	16029	423395
2010	189	14439	391954
2011	194	16293	429974
2012	195	16837	444519
2013	193	16400	440898
2014	194	15859	421947
2015	193	16129	436378
2016	194	15990	420155
2017	196	16806	439624
2018	196	16980	455205
2019	195	17526	466114
2020	193	15165	396548
2021	194	16675	442530

Table 70: Summary of data by year for all country speeches made in the UN General Debate

The data was pre-processed for analysis by removing punctuation, symbols, numbers, stopwords, and URLs. In addition, all tokens were normalised (lower-cased). All pre-processing and analysis was carried out in R using the “quanteda” package.[375]

Caveats

The search for climate change terms in the context of public health references is a proxy for the semantic linkage between the two sets of terms in GD statements. This approach produces a scalable and reproducible measure with a high degree of reliability that does not involve human judgement or subjective biases. However, there may be examples of governments referring to climate change and health but not the direct linkages between the two, which are included in the count; and there may be examples of governments discussing the health impacts of climate change in their UNGD statements, which are not included in the measure because the distance between the mention of the climate change term and the health term exceeds 25 words. Based on analysing a sample of the speeches and references, such cases are relatively rare and do not have a significant bearing on the indicator or the trends uncovered.

It is also worth noting that the analysis here is based on a narrow range of search terms, which excludes reference to many of indirect links between climate change and health. A number of GD statements in this time period refer to such indirect connections, such as the effects of climate change on water and agriculture —

however, these are not included here. Therefore, the results present a conservative estimate of high-level political engagement with the intersection of climate change and health. Future work in this area will consider engagement with these indirect links.

Future Form of Indicator

In the future, this indicator will look more closely at the references to indirect links between climate change and health. For example, this would question the main ways in which governments view climate change impacting on health and whether this changes over time based on awareness of the multiple ways in which climate change and health are connected. Some of the references to the indirect links between climate change and health made in UNGD statements are highlighted in the main report.

Additional Information

Figure 116 shows the total number of references to health, climate change, and the intersection of the two between 1970 and 2021. Figure 117 presents the total number of references to the intersection in UNGD statements between 1970 and 2021. Figure 118 shows the proportion of countries that engage with the intersection of climate change and health between 1970 and 2021. The figures show the substantial increase in engagement with the health dimensions of climate change that occurred in 2020 and 2021. In 2019 there were 109 separate references – which was significantly higher than in previous years – and in 2021 this more than tripled to 346 individual references to the intersection of climate change and health. As noted in the main report, this is primarily driven by countries discussing climate change and the COVID-19 pandemic together.

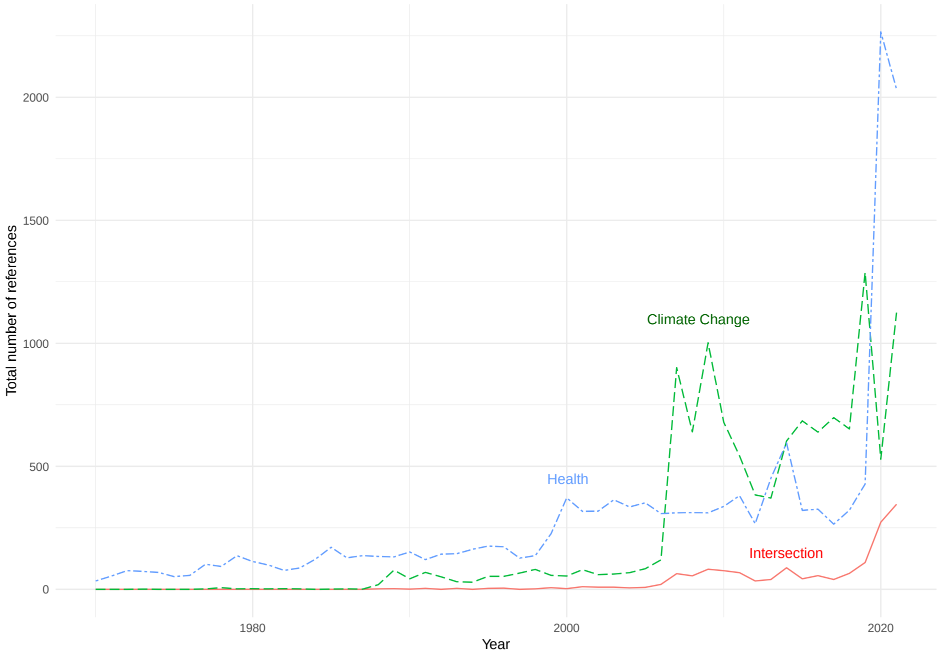

Figure 116: Total number of references to health, climate change, and intersection, 1970–2021.

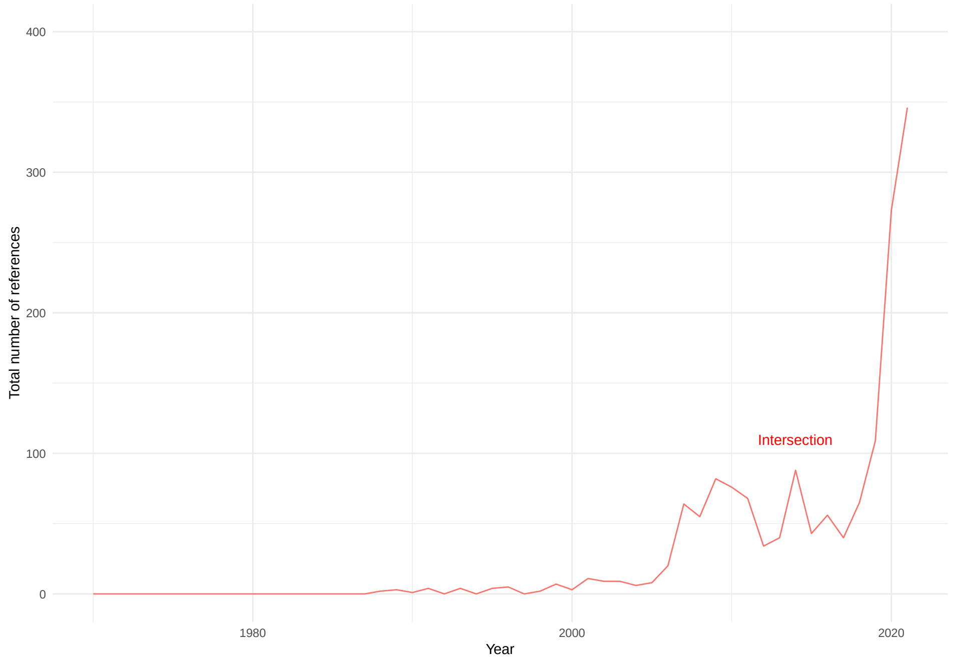

Figure 117: Total number of references to intersection, 1970–2021.

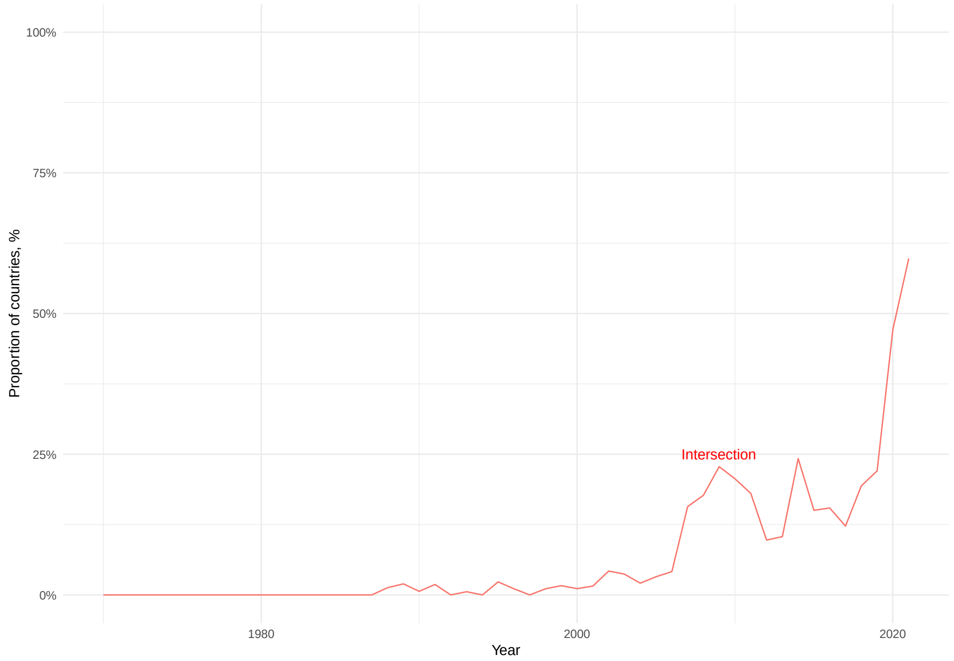

Figure 118: Proportion of countries referring to the intersection of health and climate, 1970–2021.

There is growing awareness of the gendered impacts of climate change and health. Considered here is the extent to which references to the health dimensions of climate change in countries UN General Debate statements engage with gender issues. This is done by further examining the references to the intersection of climate change and health. Once all the references to this intersection in UNGD statements for 1970–2021 were identified, the additional search terms related to gender were used to identify which of the intersection references also engaged with gender issues. The gender-related search terms were as follows: *women, women's, maternal, inequality, inequalities, gender, empowerment, sex, sexual, violence, violent, girls, reproduction, reproductive*. Hence, the analysis considers whether the 25 words of text identified in the primary search (for climate change and health terms) includes a reference to at least one of these gender-related keywords. Figure 119shows that only 3% of all references to the intersection of climate change and health also include a mention

of gender. The figure shows that this is lower than in previous years, with the 2013 seeing 26% of all climate change-health references including a gender mention.

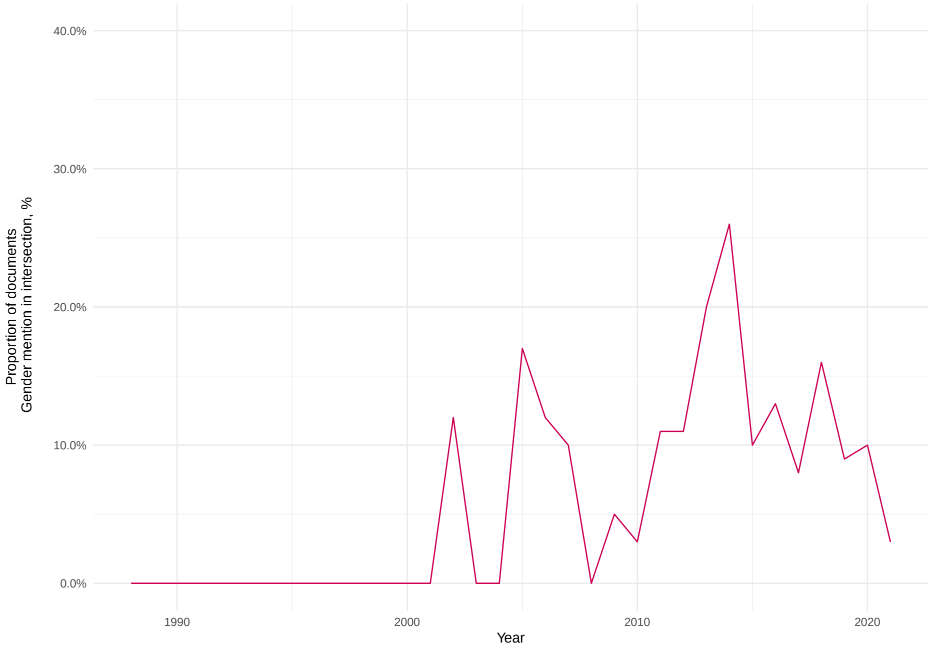

Figure 119: Proportion of references to the intersection of health and climate change that include a reference to gender, 1970–2021.

Figure 120 presents the proportion of countries that engage with the intersection of climate change and health by WHO region. The significant increase in engagement in 2021 can be seen in all of the regions – with at least 30% of countries in all of the regions referring to the health dimensions of climate change in their 2021 UNGD statements. As in previous years there is especially high engagement from countries in the Western Pacific region, with 78% of countries referring to the intersection of climate change and health. In fact, all countries in the South-East Asia and North American region refer to the intersection of health and climate change — though it is worth noting that these two regions consist of a small number of countries (9 and 2 respectively). It is worth noting that the relatively higher level of political engagement by countries in the Western Pacific is especially driven by the small island development states (SIDS) in this region. The lowest engagement is by countries in the Eastern Mediterranean regions with 30% of countries in this region referring to the intersection of climate change and health.

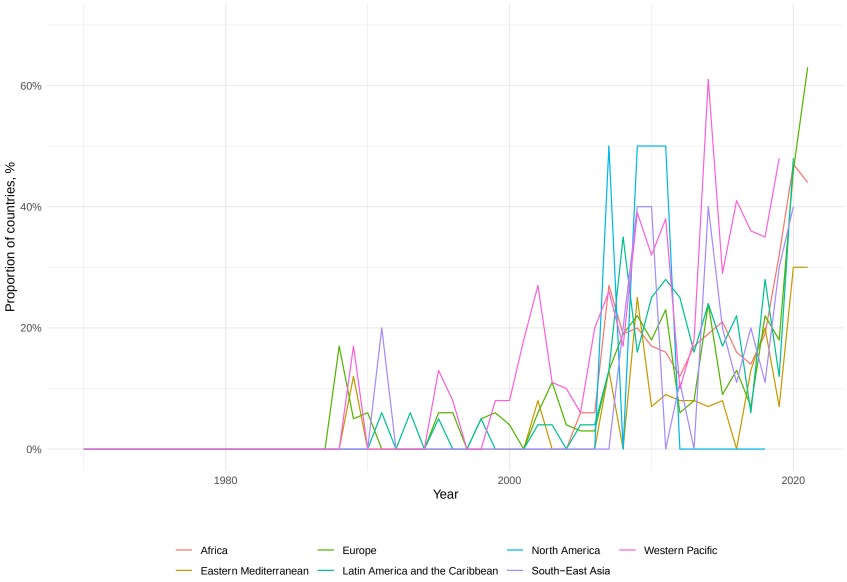

Figure 120: Proportion of countries referring to intersection of health and climate change by region, 1970–2021.

Figure 121 presents the total number of references to the climate change-health link between 1970 and 2021 by WHO region. The figure shows that the highest number of references to the intersection of climate change and health come from four regions: Africa, Europe, Latin America and the Caribbean, and the Western Pacific. In general, the figure suggests that there is lower engagement among countries in the Eastern Mediterranean, North America, and South-East Asia.

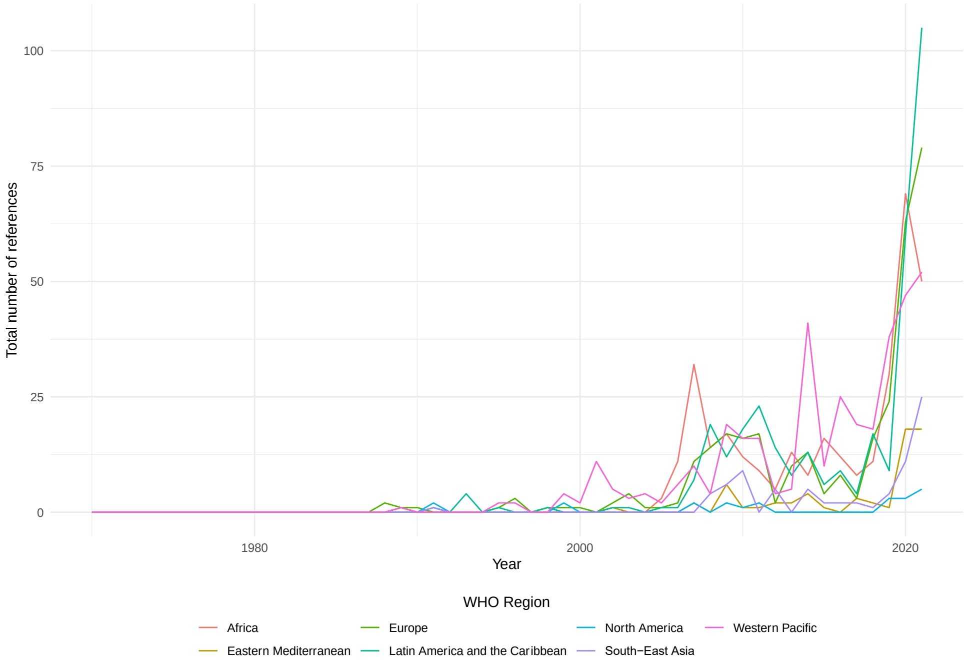

Figure 121: Total number of references to intersection by region, 1970–2021.

In addition to grouping countries by WHO region, also considered were different types of countries in terms of their potential importance and role in addressing issues related to climate change. This is provided in Figure 122 and Figure 123. As noted in previous years' reports, the SIDS have driven much of the engagement with the health impacts of climate change, as well as climate change more generally, in the UN General Assembly. As such, the analysis includes a SIDS grouping. Arguably the three most important countries/unions in addressing climate change are USA, China, and the EU. This is both in terms of their carbon dioxide emissions and their power within the international system. These are referred to as Tier 1 countries in Figure A7 and A8. Finally, an additional grouping of countries is considered that are also important in terms of their CO_2 emissions, their influence in international politics, and their potential impact on addressing climate change. This grouping — Tier 2 — includes: Poland, Australia, South Africa, Brazil, India, France, Germany, and Indonesia.

Figure 122 shows the proportion of countries that engage with the intersection of climate change and health based on these country groupings. Figure 123 shows the total number of references to the climate change-health intersection according to these groupings. Both figures demonstrate the higher level of engagement with the climate change-health linkages by SIDS than by Tier 1 or Tier 2 countries. However, it is worth noting that Figure 123 shows that a growing number of Tier 1 and Tier 2 countries are engaging with the climate change-health intersection.

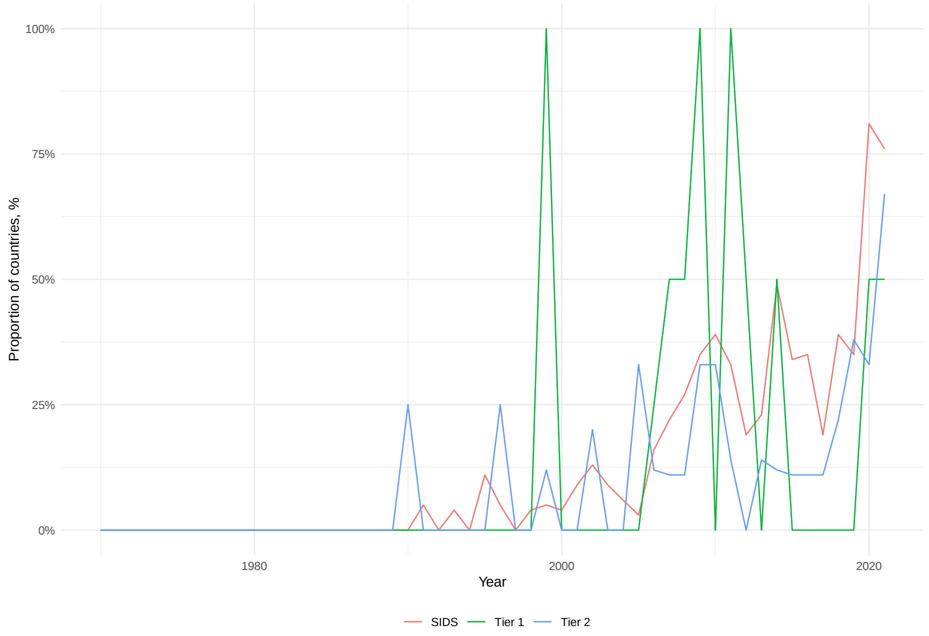

Figure 122: Proportion of countries referring to intersection of health and climate change by country grouping, 1970–2021.

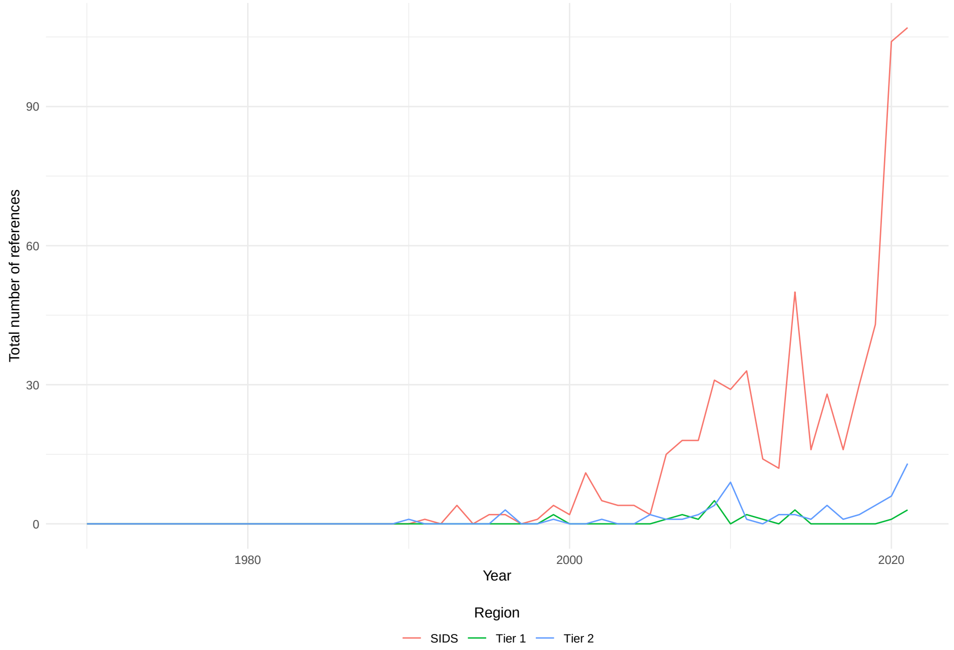

Figure 123: Total number of references to intersection by country grouping, 1970–2021.

Also considered is government engagement with the health dimensions of climate according to countries' Human Development Index (HDI) categories. Figure 124shows the proportion of countries engaging with the intersection of climate change and health by HDI category, and Figure 125 shows the total number of references by countries' HDI categories. Both figures show the significant increase in engagement across different HDI groupings. There has been a slight drop in engagement among the countries with low HDI, though this groups still sees higher engagement than others.

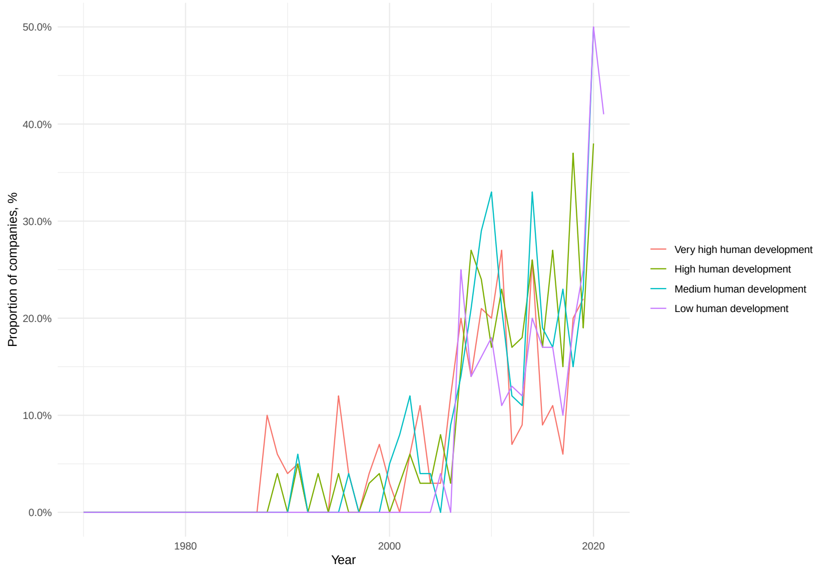

Figure 124: Proportion of countries referring to intersection of health and climate change by HDI categories, 1970–2021

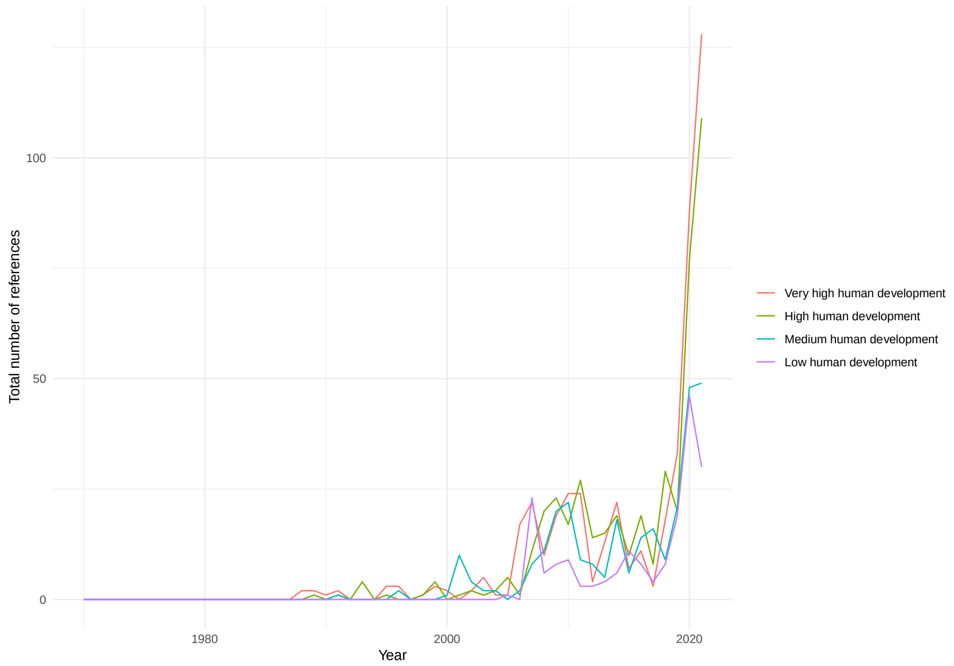

Figure 125: Total number of references to intersection by HDI categories, 1970–2021.

Figure 126 presents a world map, which shows the countries that refer to the intersection of climate change and health in their 2021 UNGD statements, and the number of individual references they make. The map shows that over half of all countries mentioned the intersection of health and climate change in their 2021 address. The map also shows that despite the higher engagement, there is still evidence of a divide between high-income countries on the one side, and low- and middle-income countries on the other side. The latter tend to engage more with climate change and health, particularly when SIDS are included. Due to their size, SIDS do not show up on the map. As noted above, SIDS tend to be highly represented among nations engaging with the health-climate change links.

Figure 127 and Figure 128 present world maps, which show the countries that refer to public health and climate change respectively in their 2021 UNGD statements, as well as indicating the number of references made by each country. The figures demonstrate that there is considerable engagement with the issues of climate change and health separately. In 2021 virtually all countries mentioned health in their UNGD statements, as can be seen

in Figure 127 and Figure 128 how that as well as a much larger share of countries around the world discussing climate change and health in their GD statements compared to those discussing the intersection, there is also much deeper engagement with these two areas individually, in that countries tend to make a number of references to climate change and health in their GD statements.

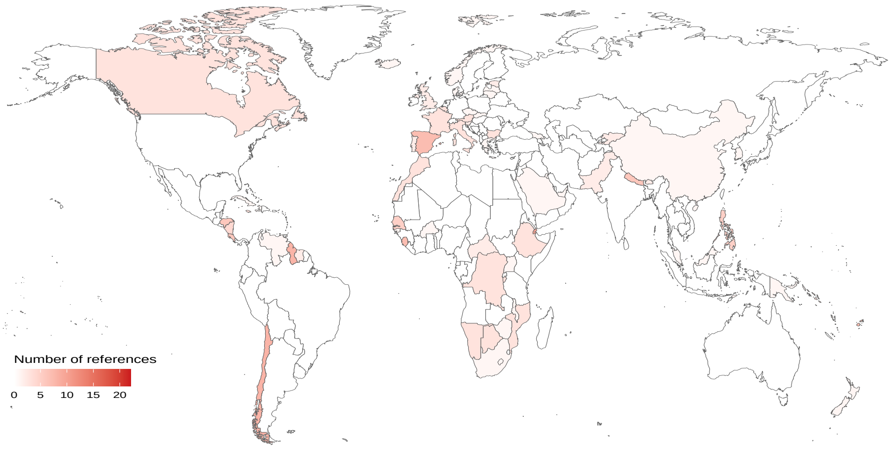

Figure 126: World map showing references to intersection of climate change and health, 2021.

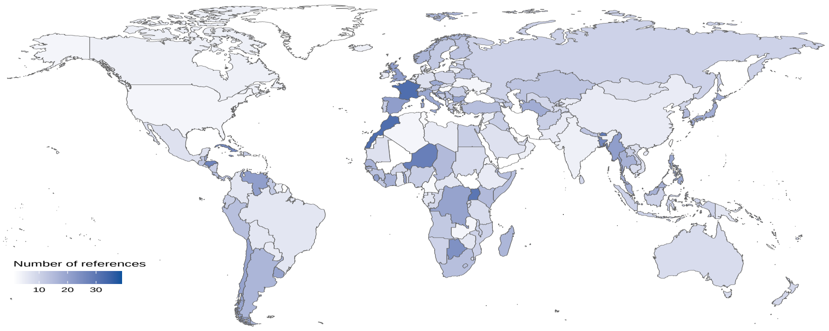

Figure 127: World map showing references to public health, 2021.

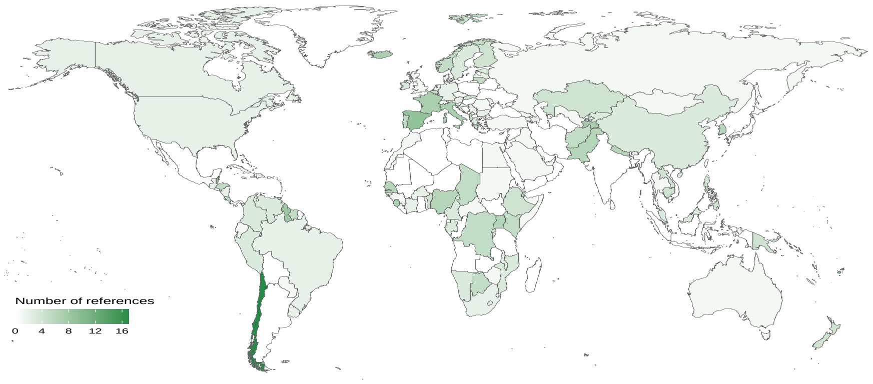

Figure 128: World map showing references to climate change, 2021.

Figure 129 to Figure 136 show engagement with climate change, health, and the intersection of climate change and health over 1970–2021 for selected countries.

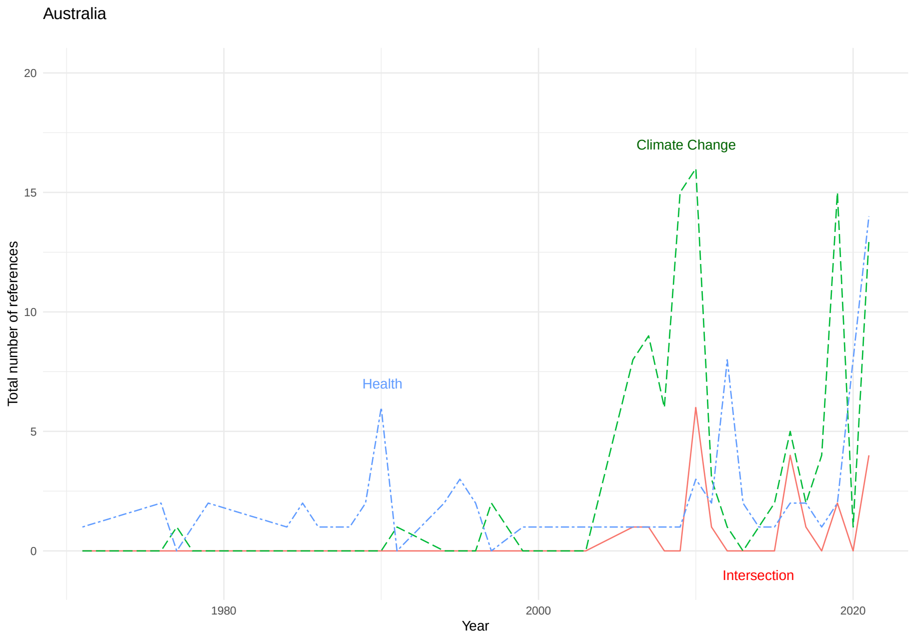

Figure 129: Engagement with climate change, health, and the intersection of climate change and health over 1970–2021 in Australia

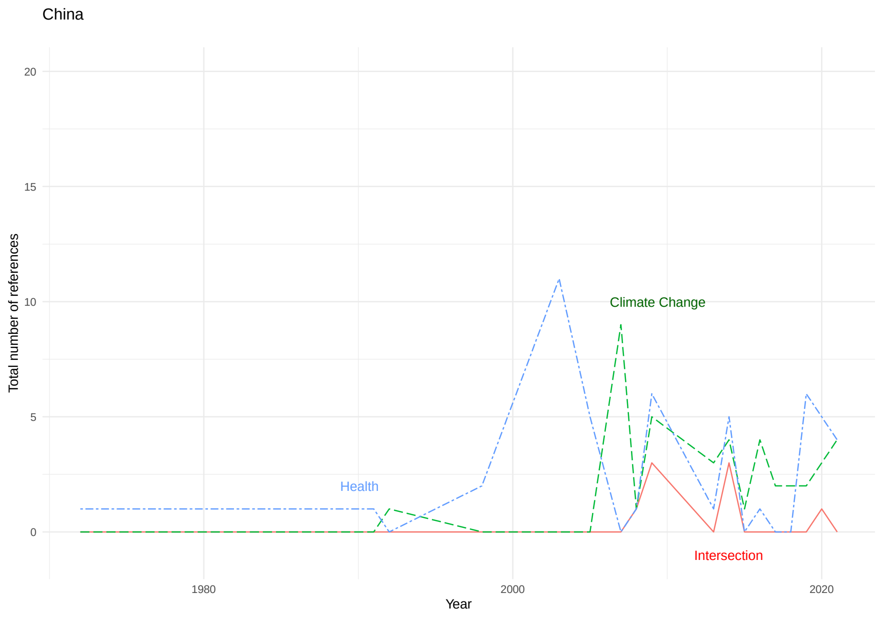

Figure 130: Engagement with climate change, health, and the intersection of climate change and health over 1970–2021 in China

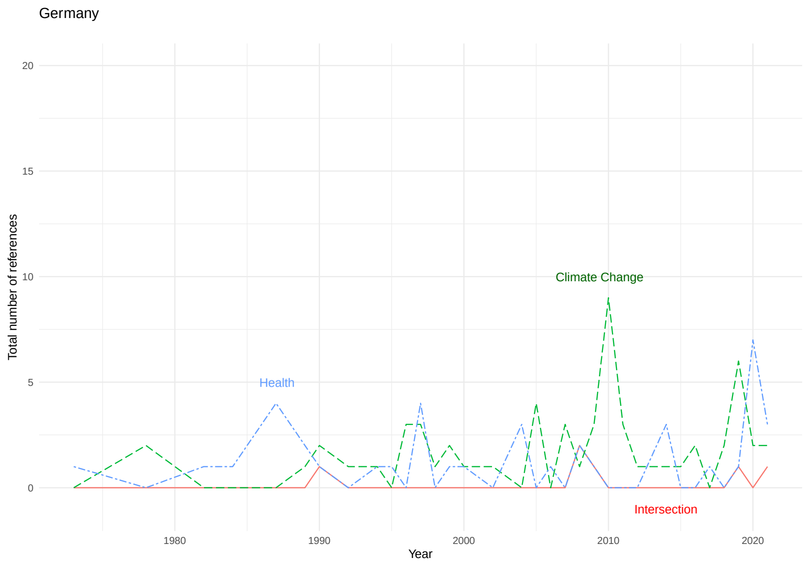

Figure 131: Engagement with climate change, health, and the intersection of climate change and health over 1970–2021 in Germany

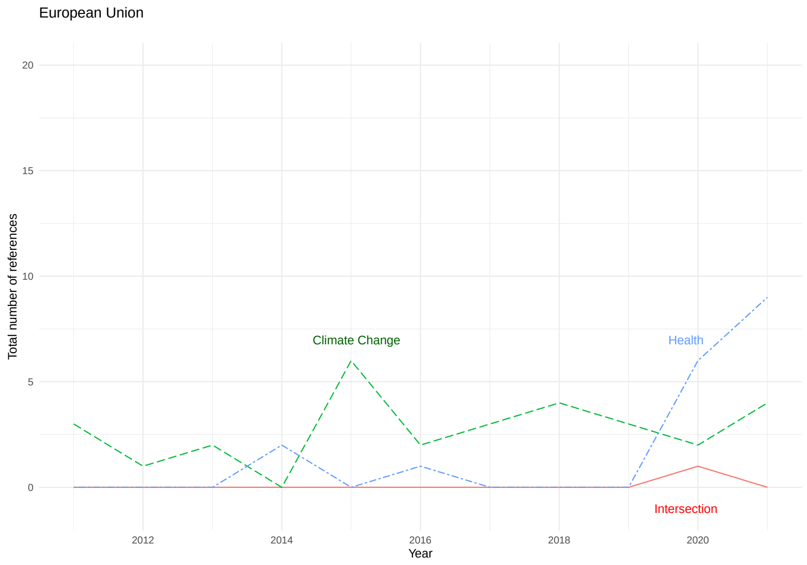

Figure 132: Engagement with climate change, health, and the intersection of climate change and health over 1970–2021 in the European Union

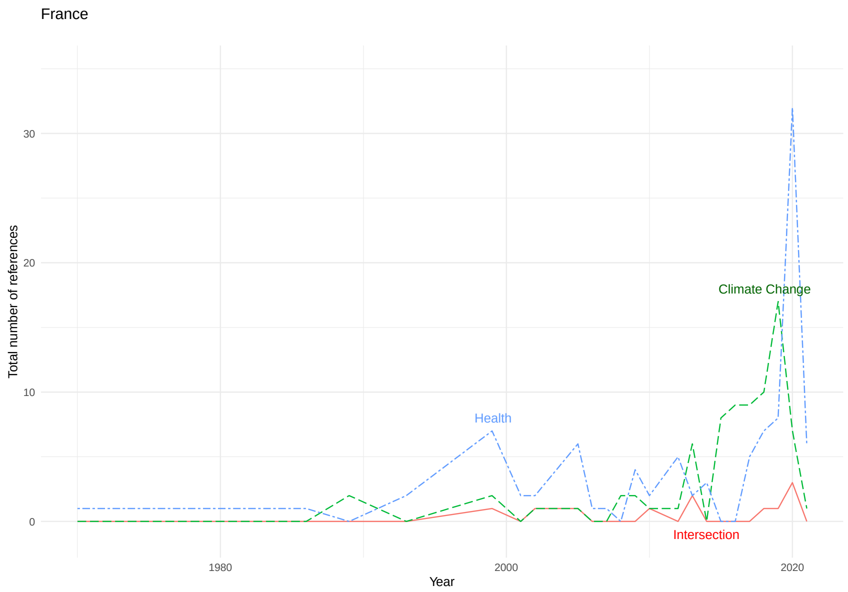

Figure 133: Engagement with climate change, health, and the intersection of climate change and health over 1970–2021 in France.

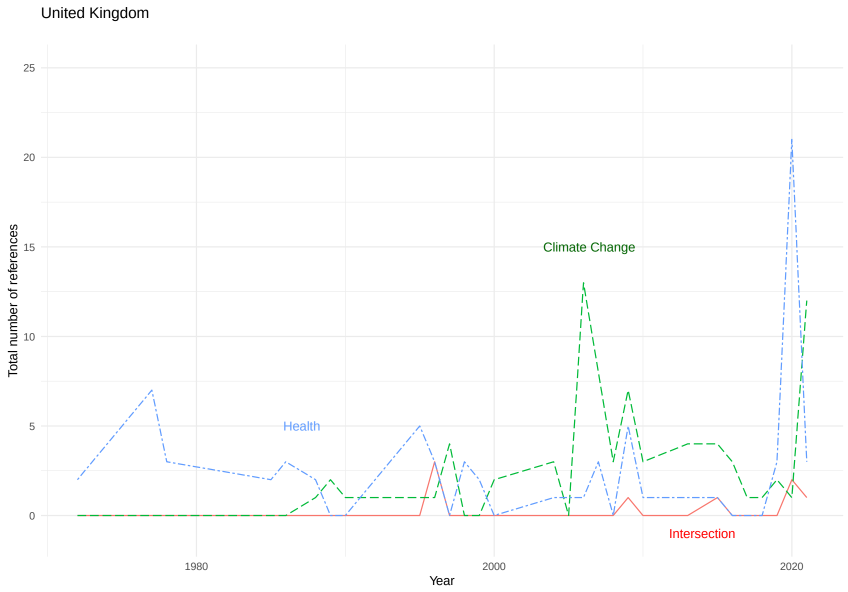

Figure 134: Engagement with climate change, health, and the intersection of climate change and health over 1970–2021 in the United Kingdom.

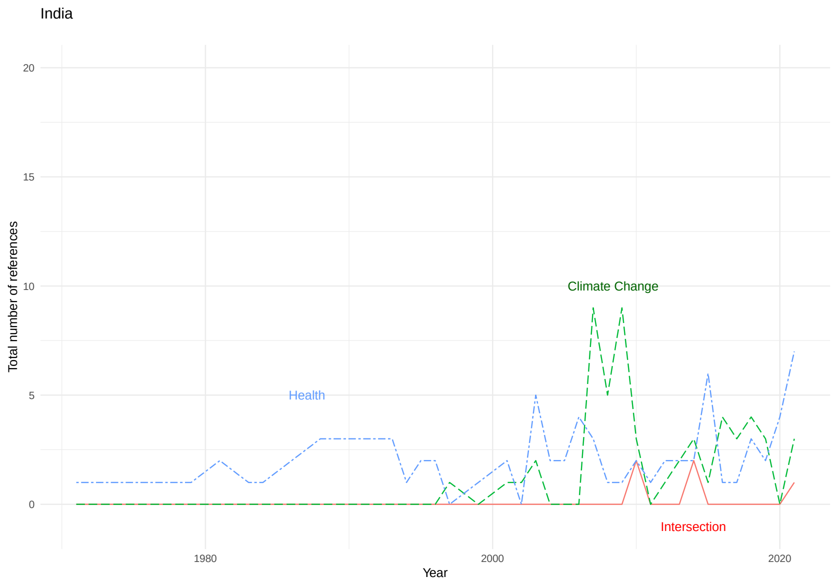

Figure 135: Engagement with climate change, health, and the intersection of climate change and health over 1970–2021 in India.

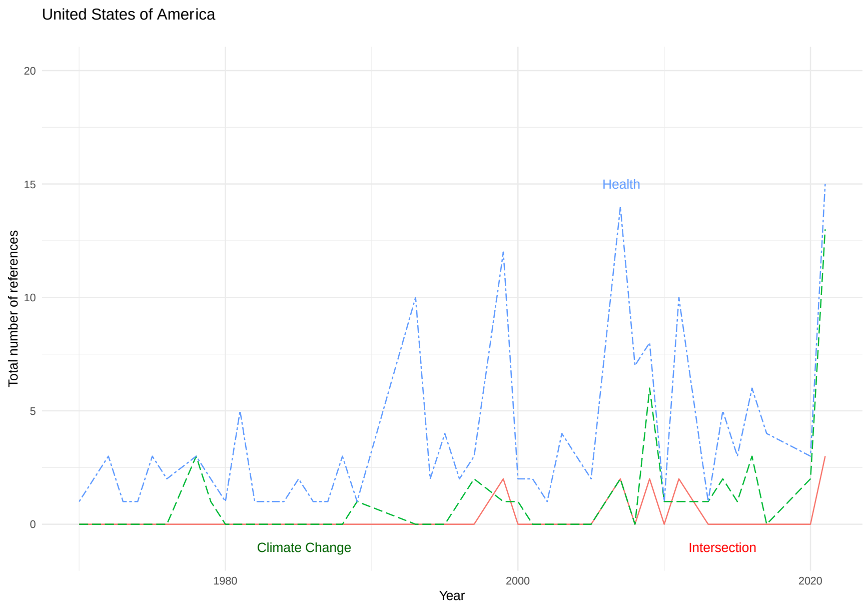

Figure 136: Engagement with climate change, health, and the intersection of climate change and health over 1970–2021 in the United States of America.

5.4.2. Engagement in Health and Climate Change in the Nationally Determined Contributions (NDCs)

Methods

Under the Paris Agreement, Nationally Determined Contributions (NDCs) for each Party to the Agreement are communicated through the NDC registry. As a measure of engagement across climate change and health, and in particular, of governments' appreciation of the health risks of climate change, all available first and second NDCs (as of March 01, 2022) were analysed with respect to their inclusion of health-related terms. Analysis of both first and second NDCs allows some indication of changes in climate-related health concerns over time.

The two categories used in analysis (health-related terms and exposure terms) were developed iteratively, with text extracted from the NDCs if it fell into one of the two categories. Health-related terms were developed iteratively. Text referring to health directly, denoting either its presence (e.g. health, well-being) or its absence (e.g. death, illness, disease), was extracted from the NDCs. Any term mentioning 'health' (e.g. health centre, health surveillance) was also included, though false positives were excluded (e.g. ecosystem health, ocean health).

All terms were agreed upon by both researchers working on the indicator. Variations of all terms occurred within the NDCs and an indicative list can be found below.

Relevant text was extracted and organised within a Microsoft Excel spreadsheet. Text was extracted in sentence form if containing a health-related term. Sentences were understood as capital letter to full-stop, or semi-colon in a list or table. In addition, the section of the NDC in which the text was extracted was also noted to establish the context of health mentions.

An iterative list of keywords was kept to later establish the proportion of countries using those key terms. This also allowed for patterns to be investigated by Human Development Index groupings and World Health Organisation regions.

Indicative list of key health-related terms

- Health
- Disease and illness (including syndrome, dengue, chikungunya, leptospirosis, typhoid, Lyme disease, malaria, diarrhoea, zika, leishmaniasis, pathogen, West Nile, fever, morbidity, epidemic)
- Death (including loss of life, fatalities, mortality, deaths)
- Malnutrition (including undernutrition, starvation)
- Heat stress
- Medical infrastructure (including medicine, medical, hospital, clinical, diagnostic, patients
- Mental health (including psychological, emotional)
- Well-being
- Injury

Data

NDCs included

For the first round of NDCs, 158 were retrieved from the UNFCCC website (www4.unfccc.int/sites/NDCStaging/Pages/All.aspx), representing 185 nations in total (the European Union NDC represents 28 nations, see Table 71). For the second round of NDCs, 126 were retrieved, representing 152 (with the European Union NDC now representing 27 nations, see Table 71). Several NDCs were in either French or Spanish and were therefore translated using Google Translate. While each document covered broadly the same material (all had mitigation strategies, most had adaptation sections, coverage of national circumstances, and an account of fairness and ambition within the NDC), most were different in presentation, making extraction of text difficult in some instances. For example, some NDCs appeared to be screenshots of pages, which meant Adobe DC was used for its Optical Character Recognition capacity, converting image into text.

**Country**	First round	Second round
Afghanistan	+	-
Albania	+	+
Algeria	+	-
Andorra	+	+
Angola	-	+
Antigua and Barbuda	+	+
Argentina	+	+
Armenia	+	+
Australia	+	+
Azerbaijan	+	-
The Bahamas	+	-
Bahrain	+	+
Bangladesh	+	+
Barbados	+	+
Belarus	+	+
Belize	+	+
Benin	+	+
Bhutan	+	+
Bolivia	+	-
Bosnia and Herzegovina	-	+
Botswana	+	-
Brazil	+	+
Brunei Darussalem	-	+
Burkina Faso	+	+
Burundi	+	+
Cambodia	+	+
Cameroon	+	-
Canada	+	+
Cape Verde	+	+
Central African Republic	+	+
Chad	+	+
Chile	+	+
China	+	+
Colombia	+	+
Comoros	+	+

Congo	+	+
Cook Islands	+	-
Costa Rica	+	+
Cote d'Ivoire	+	-
Cuba	+	+
Djibouti	+	-
Dominica	+	-
Dominion Republic	+	+
DRC	+	-
Ecuador	+	-
Egypt	+	-
El Salvador	+	+
Equatorial Guinea	+	-
Eritrea	+	-
Ethiopia	+	+
EU	+	+
Fiji	+	+
Gabon	+	-
The Gambia	+	+
Georgia	+	+
Ghana	+	+
Great Britain	-	+
Grenada	+	+
Guatemala	+	+
Guinea	+	+
Guinea-Bassau	+	+
Guyana	+	+
Haiti	+	-
Honduras	+	+
Iceland	+	+
India	+	-
Indonesia	+	+
Israel	+	+
Jamaica	+	+
Japan	+	+
Jordan	+	+

Kazakhstan	+	-
Kenya	+	+
Kiribati	+	-
Korea, Dem. Rep.	+	+
Korea, Rep.	+	+
Kuwait	+	+
Kyrgyzstan	+	+
Lao PDR	+	+
Lebanon	+	+
Lesotho	+	-
Liberia	+	+
Liechtenstein	+	-
Macedonia, FYR.	+	+
Madagascar	+	-
Malawi	+	+
Malaysia	+	+
Maldives	+	+
Mali	+	+
Marshall Islands	+	+
Mauritania	+	+
Mauritius	+	+
Mexico	+	+
Micronesia, Fed. Sts.	+	-
Moldova	+	+
Monaco	+	+
Mongolia	+	+
Montenegro	+	+
Morocco	+	+
Mozambique	+	+
Myanmar	+	+
Namibia	+	+
Nauru	+	+
Nepal	+	+
New Zealand	+	+
Nicaragua	+	+
Niger	+	+

Nigeria	+	+
Niue	+	-
Norway	+	+
Occupied Palestinian Territories	+	+
Oman	+	+
Pakistan	+	+
Palau	+	-
Panama	+	+
Papua New Guinea	+	+
Paraguay	+	+
Peru	+	+
Qatar	+	+
Rwanda	+	+
Samoa	+	+
San Marino	+	-
Sao Tome and Principe	+	+
Saudi Arabia	+	+
Serbia	+	-
Seychelles	+	+
Sierra Leone	+	+
Singapore	+	+
Solomon Islands	+	+
Somalia	+	+
South Africa	+	+
Sri Lanka	+	+
St. Kitts and Nevis	+	+
St. Lucia	+	+
St. Vincent and the Grenadines	+	-
Sudan	+	+
Suriname	+	+
Swaziland	+	+
Switzerland	+	+
Syrian Arab Republic	+	-
Tajikstan	+	+
Tanzania	+	+
Thailand	+	+

Timor-Leste	+	-
Togo	+	+
Tonga	+	+
Trinidad and Tobago	+	-
Tunisia	+	+
Turkey	-	+
Turkmenistan	+	-
Tuvalu	+	-
Uganda	+	+
Ukraine	+	+
United Arab Emirates	+	+
United States of America	+	+
Uryguay	+	-
Uzbekistan	+	+
Vanuatu	+	+
Venezuela	+	+
Vietnam	+	+
Zambia	+	+
Zimbabwe	+	+

Table 71: NDCs retrieved from UNFCCC website and included in first and second round

Other results

NDC Wordcount

Content of the NDCs is, at least to some extent, determined by their form. For example, a larger wordcount generally means more space for discussions of health and other key features of adaptation and mitigation. The second wave of NDCs (considered here as those uploaded to the UNFCCC registry from January 2020 onwards) have considerably higher wordcounts on average: first wave NDCs were 4126 words on average, compared to 11691 in the second wave, a percentage increase of 181%. Figure 137 below highlights that the largest shifts in wordcount are in the African, American, Western Pacific and European regions, while the two highest (Eastern Mediterranean and South East Asian Region) from the first wave demonstrate the smallest change in wordcount. Figure 138 demonstrates that the largest shift is in nations from the High HDI groups, with those in the very high category showing the smallest shift.

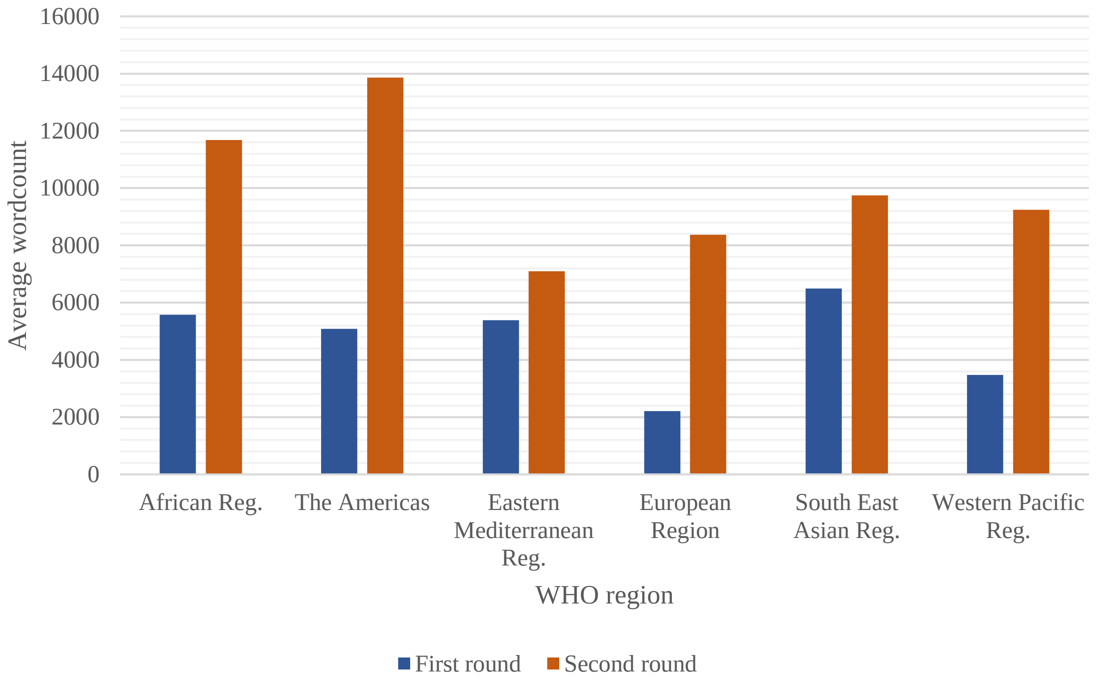

Figure 137: Average NDC wordcount for both first and second waves by WHO region.

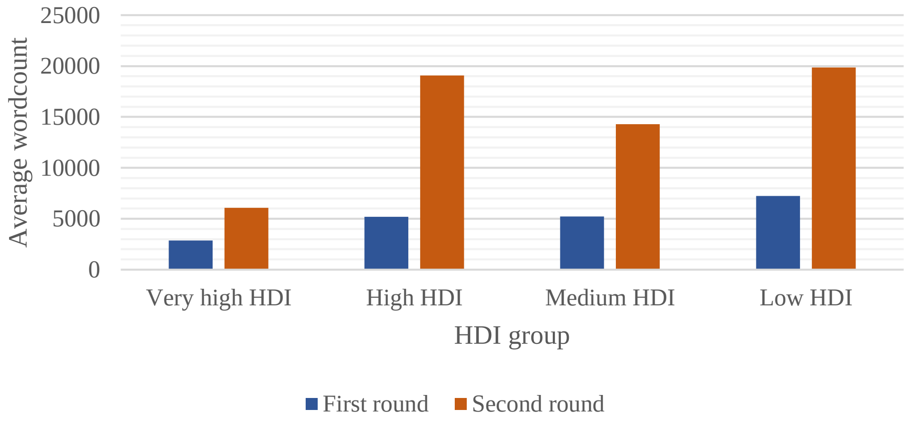

Figure 138: Average NDC wordcount for both first and second waves by HDI group.

Types of health mentions

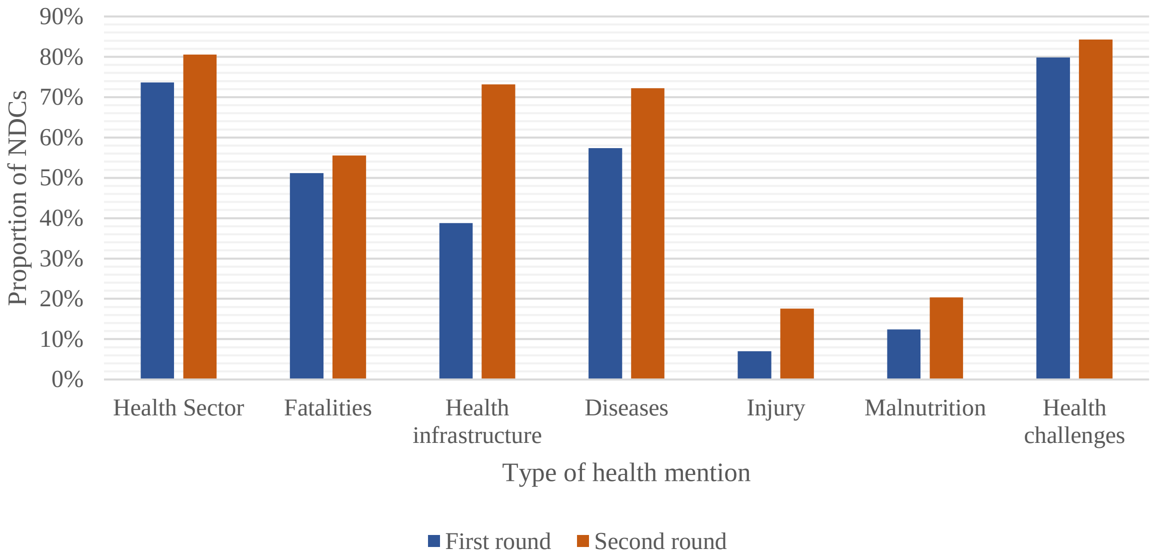

Figure 139: Proportion of NDCs mentioning different types of health keyword.

82% (129 of 157) of the first wave NDCs contained a health key word, compared to 86% (108 of 126) of the second wave NDCs (Figure 139). When aggregated into HDI groups (Figure 140; all countries in the high HDI group contain a health key word in the second round of NDCs, showing an increase from 85% in the first round. The very high HDI group also increases from 65% in the first round to 71% in the second. Both the low and medium HDI groups show small decreases from already high proportions in the first round.

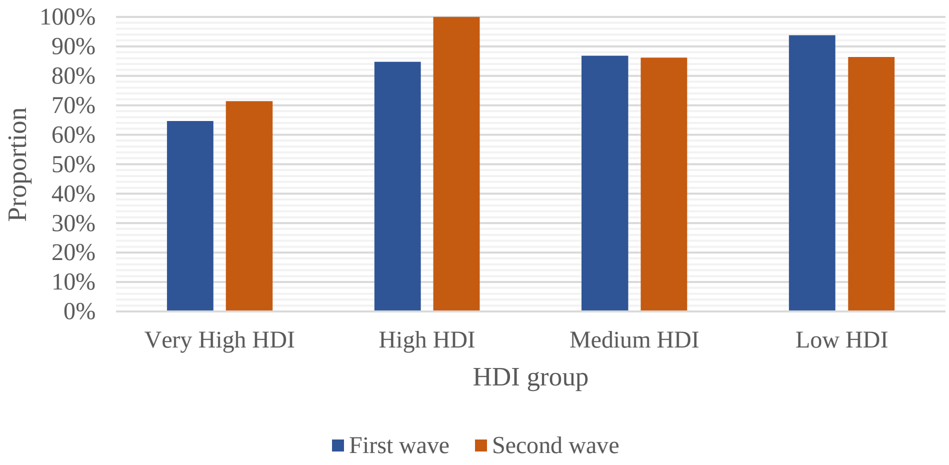

Figure 140: Proportion of NDCs by HDI group mentioning any health term.

The same broad pattern from the above figure showing any health mention is observable in direct mentions of health across HDI groups (Figure 141). These made up the majority of the health keyword mentions within the NDCs.

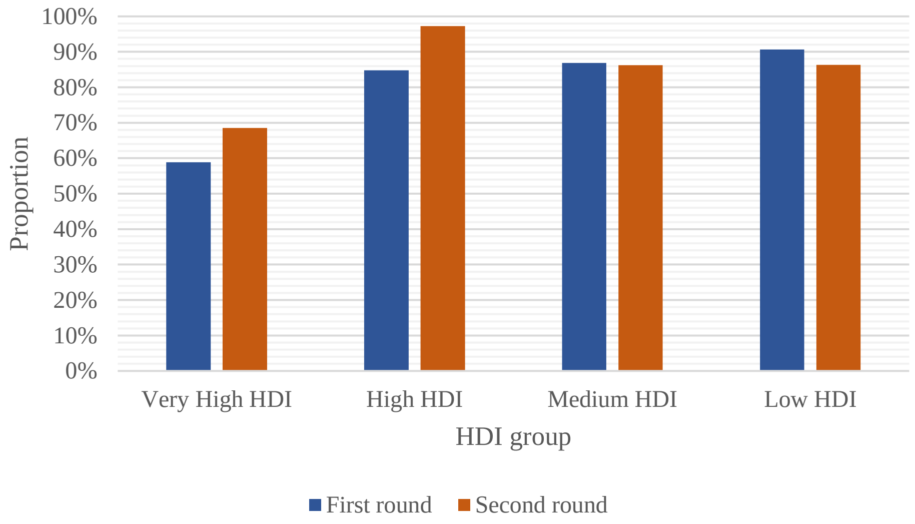

Figure 141: Proportion of NDCs by HDI group mentioning health directly.

51% of all first round NDCs and 56% of all second round NDCs mention a keyword related to mortality or fatalities. This is often related to the reporting of extreme events but is also related to background levels of mortality. Broken down by HDI group (Figure 142) the biggest increases are shown by the medium (from 50% to 66%) and very high (12% to 37%) groups that show the biggest increase in such descriptions. Countries in the medium and low HDI groups continue to use these terms the most, however.

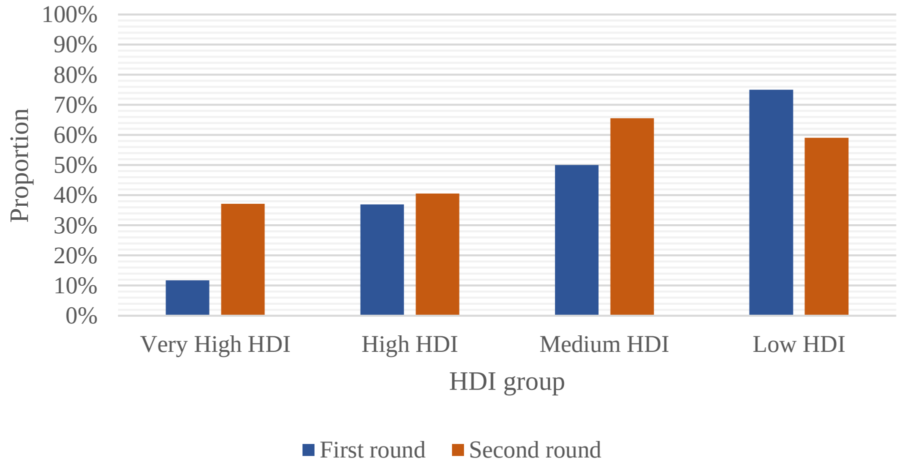

Figure 142: Proportion of NDCs by HDI group mentioning a term related to mortality, fatalities, loss of life or deaths.

57% of first round NDCs mention disease or illness, whether more generally as an umbrella term, or more specifically, such as, for example, dengue, chikungunya, malaria or Lyme disease. This increases to 72% in the second round of NDCs. Aggregating countries by HDI group (Figure 143) the large increase comes countries in the very high (24% to 40%), high (48% to 70%) and medium (45% to 69%) HDI groups. There is also a very slight increase across low HDI countries (75% to 77%), which has the highest proportion of mentions of disease or illness across both rounds of NDCs.

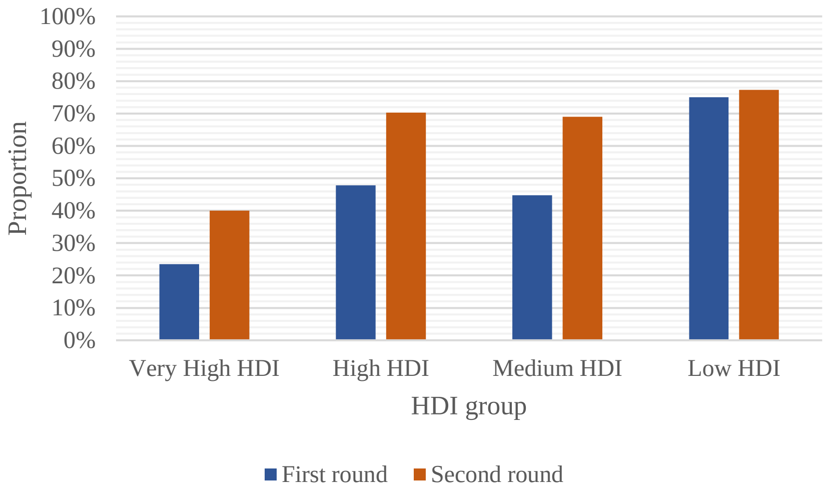

Figure 143: Proportion of NDCs by HDI group mentioning a term related to disease or illness.

In the first round of NDCs 12% mentioned either malnutrition or undernutrition. In the second round this is 20%. Countries across all HDI groups show an increase in use of these terms within their NDCs (Figure 144). In the second round of NDCs countries in all HDI groups refer to malnutrition or undernutrition.

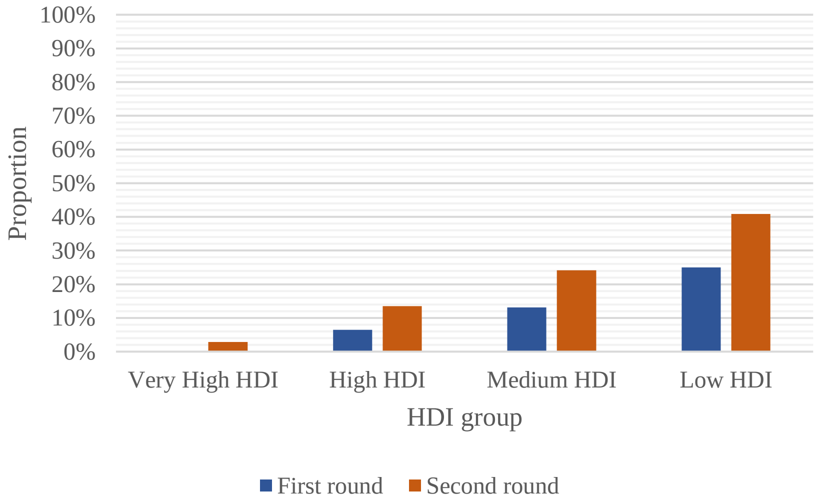

Figure 144: Proportion of NDCs by HDI group mentioning either malnutrition or undernutrition.

Where only 19% of all first round NDCs mentioning health referred also to a gender related term – such as “gender”, “women”, “reproductive” and “maternal” – this increases to 44% in second round NDCs (Figure 145). 17% of countries in the very high HDI group referred to a gender-related term in the second round, where none did so in the first round. Large increases are apparent in high (from 15% to 46%) and medium (from 16% to 55%) HDI group countries, with a small increase in countries in the low (from 34% to 41%) HDI group.

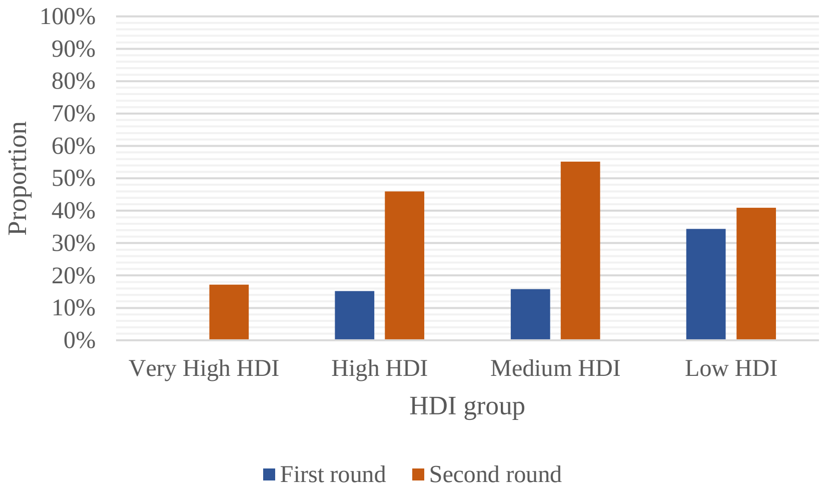

Figure 145: Proportion of NDCs by HDI group mentioning health in relation to gender.

Caveats

There may be cases within the NDCs where the discussion of health and climate change is split over two or more sentences, and where key identifiers for either the health-related category or exposure category are only implied. The researchers found that this was a rare occurrence that would not affect larger trends in the data.

Future form of indicator

This indicator uses the data from all available first NDCs held on the UNFCCC registry (https://www4.unfccc.int/sites/NDCStaging/Pages/All.aspx) as of March 1st 2022. Future reports will report on NDCs added after this date, where relevant, either as a full indicator or as a smaller section in the report.

5.5. Corporate Sector Engagement in Health And Climate Change

Methods

To produce the measure of engagement with climate change and health in companies' UN Global Compact Communication of Progress (GCCOP) reports, the publicly available GCCOP reports were used. This approach to using the GCCOP reports to produce the indicators is based on identifying references to key search terms linked to (a) health, and (b) climate change (Table 72).

Health terms	Climate change terms
• malaria<br>• diarrhoea<br>• infection<br>• disease<br>• diseases<br>• sars<br>• measles<br>• pneumonia<br>• epidemic<br>• epidemics<br>• pandemic<br>• pandemics<br>• epidemiology<br>• healthcare<br>• health<br>• mortality<br>• morbidity<br>• nutrition<br>• illness<br>• illnesses<br>• ncd<br>• ncds<br>• air pollution<br>• nutrition<br>• malnutrition<br>• malnourishment<br>• mental disorder<br>• mental disorders<br>• stunting	• climate change<br>• changing climate<br>• climate emergency<br>• climate action<br>• climate crisis<br>• climate decay<br>• global warming<br>• green house<br>• temperature<br>• extreme weather<br>• global environmental change<br>• climate variability<br>• greenhouse<br>• greenhouse-gas<br>• low carbon<br>• ghge<br>• ghges<br>• renewable energy<br>• carbon emission<br>• carbon emissions<br>• carbon dioxide<br>• carbon-dioxide<br>• co2 emission<br>• co2 emissions<br>• climate pollutant<br>• climate pollutants<br>• decarbonization<br>• decarbonisation<br>• carbon neutral<br>• carbon-neutral<br>• carbon neutrality<br>• climate neutrality<br>• net-zero<br>• net zero

Table 72: Key search terms linked to health and climate change in GCCOP reports.

These key terms have been updated from previous years to reflect the changing terminology used to discuss climate change. In order to produce an indicator of engagement with the intersection of climate change and health, the analysis focused on whether any of the climate change related terms appeared immediately before or after any public health terms in the COP reports. This was based on a search of the 25 words before and after a reference to a public health related term.

Data

To produce this indicator, the publicly available UN Global Compact COP reports are used. A total of 39,159 reports were downloaded from COP. The reports are available for companies based in 129 countries. GCCOP reports are submitted in 30 different languages. While in past years, the focus was only on the reports available in English; this year reports from all languages are included. In total, reports were submitted in 30 languages (see Table A2). These reports were translated into English using the open-source pretrained neural machine translation model Opus-MT[x] under the Huggingface[376] pipeline to implement the translation task. A number of the files were corrupt or could not be converted into plain text format for analysis. The distribution of available GCCOP reports over time is presented in Table 73.

Year	Number of reports
2011	2036
2012	2991
2013	3207
2014	3162
2015	3454
2016	3554
2017	3711
2018	3741
2019	4041
2020	3542
2021	5720

Table 73: GCCOP reports by year.

There are only single GCCOP report submissions before 2011, thus analysis is limited to the sample of GCCOP reports to the period 2011–2021. These were translated, pre-processed and prepared for the application of natural language processing by converting the reports to plain text format; removing punctuation and numbers; removing stopwords; regularising (lowercasing); and stemming. All pre-processing and analysis was carried out in R using the "quanteda" package.[375]

The different languages in which the GCCOP reports were submitted in are provided in Table 74. The table shows that 2,1205 GCCOP reports (54%) were submitted in English. In total, GCCOP were submitted in 30 different languages.

Language	Count

[x] https://github.com/Helsinki-NLP/Opus-MT.git

English	21025
Spanish	10161
French	3408
Portuguese	1179
German	947
Japanese	503
Korean	275
Italian	266
Danish	252
Turkish	224
Chinese (simplified)	161
Swedish	132
Russian	111
Polish	77
Ukrainian	69
Croatian	67
Lithuanian	66
Norwegian	57
English mix	51
Bulgarian	43
Greek	33
Catalan	20
Finnish	10
Macedonian	10
Latvian	4
Czech	3
Slovenian	2
Estonian	1
Hungarian	1
Vietnamese	1
Total	39159

Table 74: GCCOP reports by language.

Caveats

This analysis here is based on a narrow range of search terms, which excludes reference to many of indirect links between climate change and health. Reports may also discuss indirect connections, such as the effect of climate change on agriculture, however, these are not included here. Therefore, the results present a somewhat conservative estimate of high corporate engagement with the intersection of climate change and health. Future work in this area will consider engagement with these indirect links, as well as providing additional forms of analysis.

Future Form of Indicator

In the future, the indicator will look to include search terms based on indirect links between climate change and health (e.g., agriculture) to capture references to indirect links.

Additional Information

As stated in the report, and as demonstrated in Figure 146 engagement in the health and climate change nexus reached its highest level in 2021. 38% of corporations referred to the health and climate change nexus in their COP report, a far lower proportion as compared to engagement in climate change (87%) and health (72%) as separate issues.

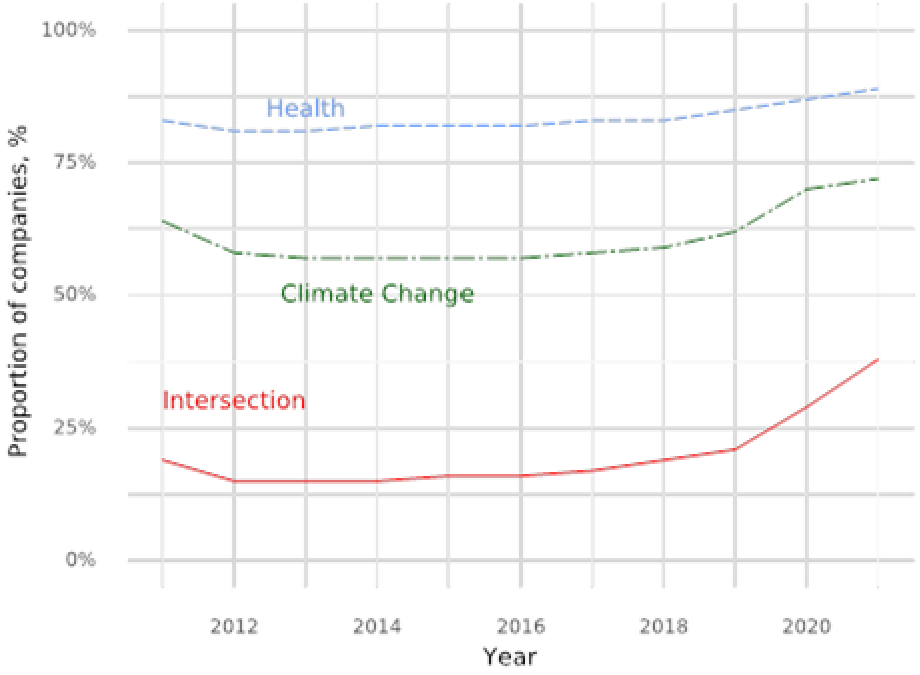

Figure 146: Proportion of companies, referring to climate change, health, and the intersection of health and climate change in their Global Compact reports, 2011–2021

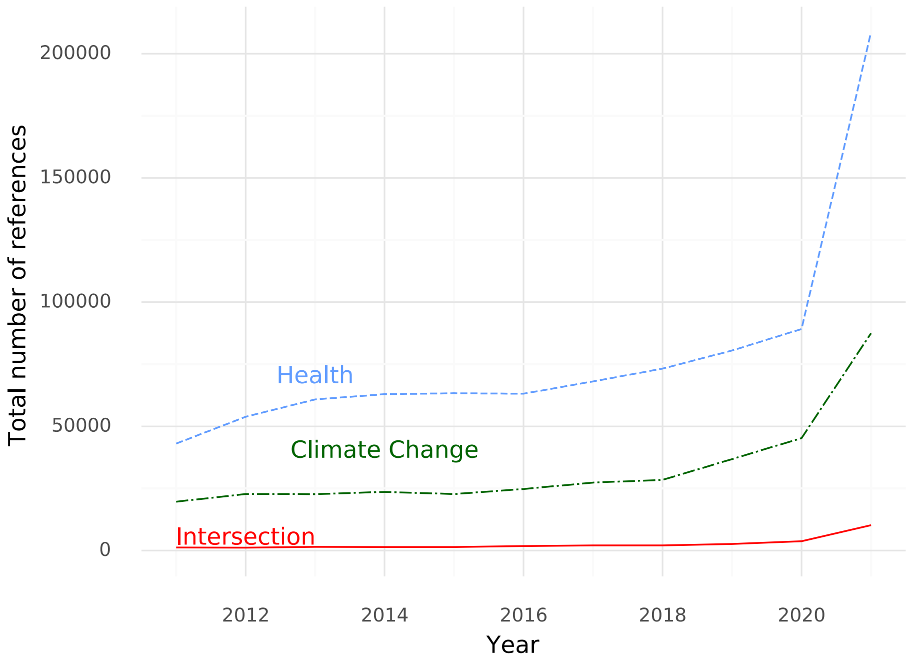

Figure 147: Total references to climate change, health, and the intersection of climate change and health, 2011–2021.

Figure 148 shows the total references with the intersection of climate change and health to better show any trends occurring in engagement. The figure shows that since 2018 there has been a sharp rise in the number of references.

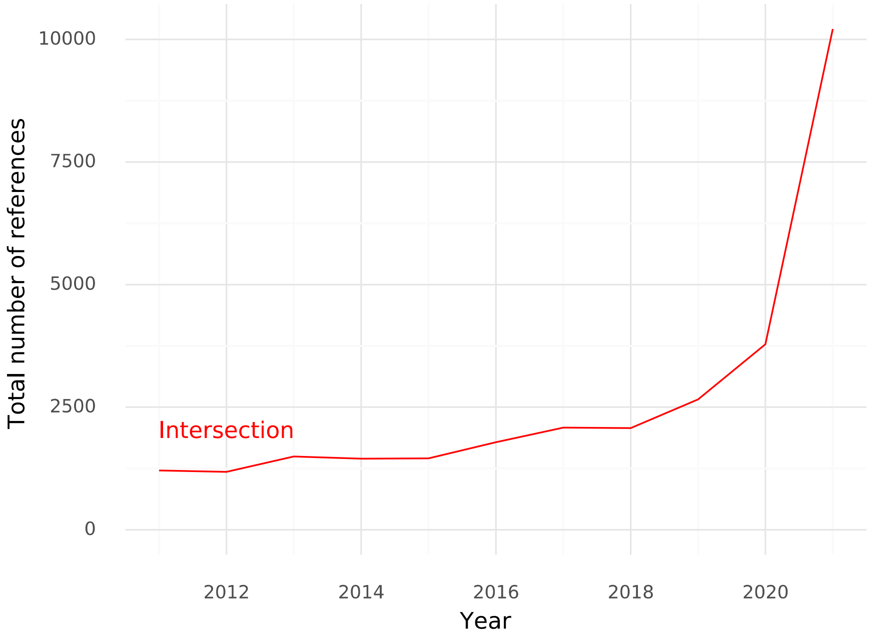

Figure 148: Total references to the intersection of climate change and health, 2011–2021.

Figure 149 shows the average number of references to climate change, health, and the intersection in GCCOP reports. The figure again demonstrates the relatively low level of engagement with the health impacts of climate change in GCCOP reports, compared to the separate references to health and climate change.

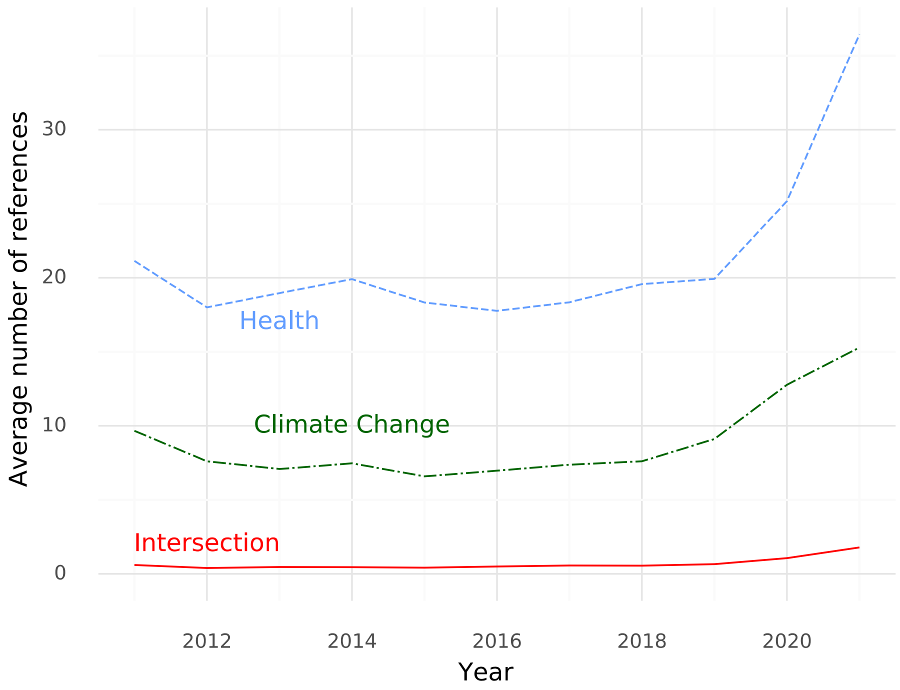

Figure 149: Average references to climate change, health, and the intersection of climate change and health in GCCOP reports, 2011–2021.

There is growing awareness of the gendered impacts of climate change and health. Consider here is the extent to which references to the health dimensions of climate change in companies' GCCOP reports engage with gender issues. This is done by further examining the references to the intersection of climate change and health. Once all the references to this intersection in GCCOP reports for 2011–2021 were identified, additional search terms related to gender were used to identify which of the intersection references also engaged with gender issues. The gender-related search terms used were as follows: *women, women's, maternal, inequality, inequalities, gender, empowerment, sex, sexual, violence, violent, girls, reproduction, reproductive*. Hence, the analysis considers whether the 25 words of text identified in the primary search (for climate change and health terms) includes a reference to at least one of these gender-related keywords.

Based on the additional search of the references to the climate change-health intersection using these gender-related keywords, references were identified to the health dimensions of climate change with a gender focus in companies' annual GCCOP reports. Figure 150 presents annual references to the gender dimensions of climate change and health in UN Global Compact COP reports between 2011 and 2021. The figure shows a steady increase in engagement between 2014 and 2018. In 2019, there was a sharp rise, with 19% of all references to the intersection of climate change and health including a mention of one of the gender keywords, followed by a fall in 2020. Engagement with gender increased in 2021 to 18%.

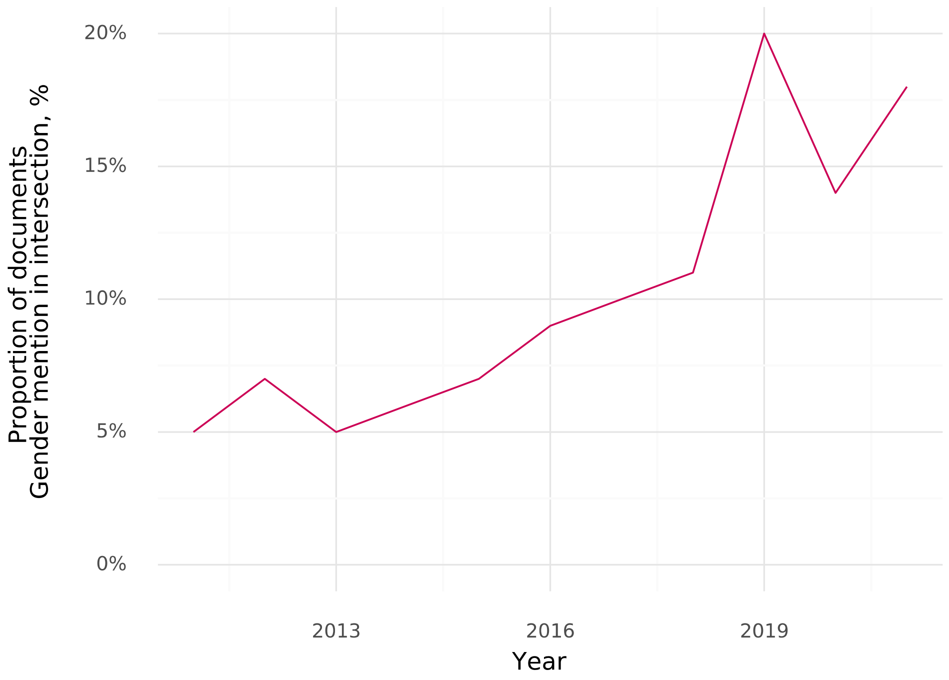

Figure 150: Proportion of references to intersection of health and climate change in GCCOP reports that include a reference to gender, 2011–2021.

Also considered here is engagement with climate change and health in the UN Global Compact COP reports by WHO region. Figure 151shows the total number of references to the climate change-health intersection based on which of the WHO regions a company is based on, and Figure 152 shows the proportion of companies based in the different WHO regions that refer to the health impacts of climate change in their annual GCCOP report.

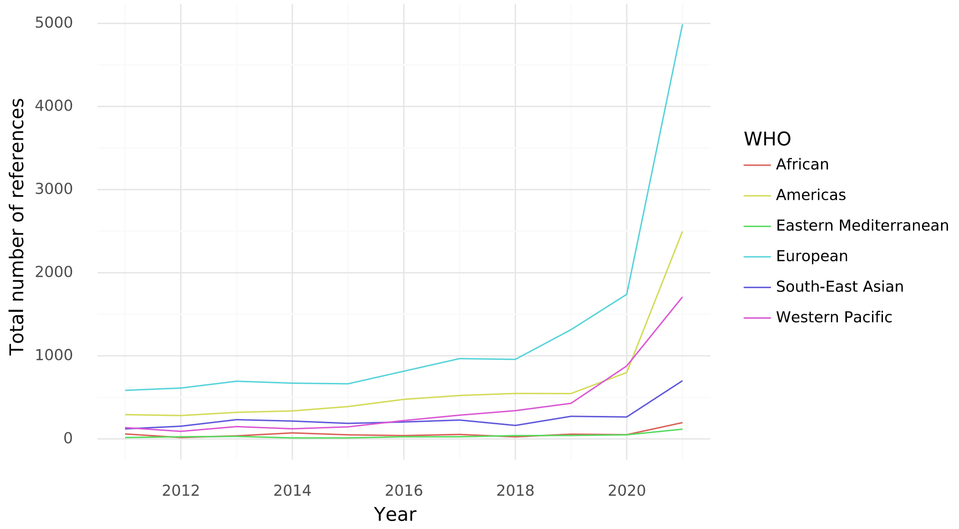

Figure 151: Total references with the intersection of climate change and health by WHO region, 2011-2021.

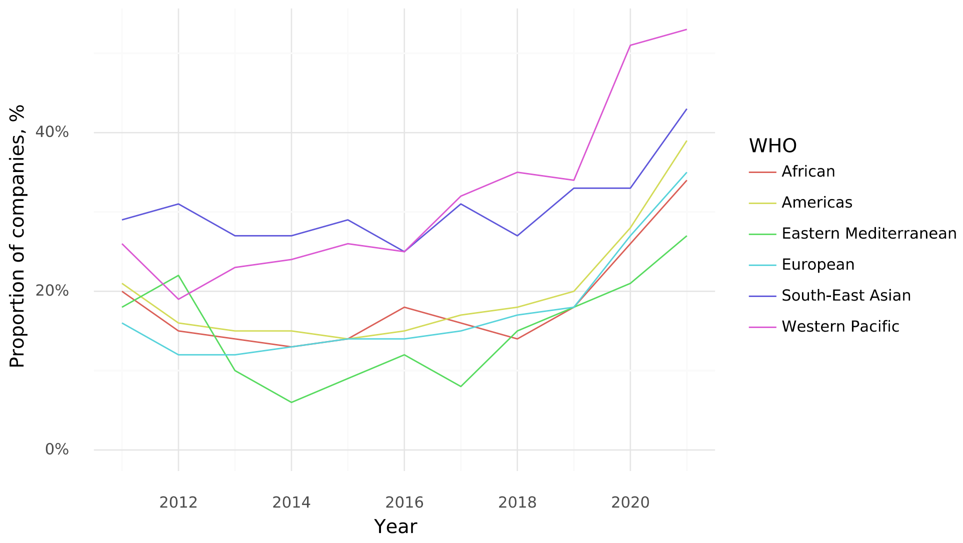

Figure 152: Proportion of companies referring to intersection of health and climate change by WHO region, 2011–2021.

Figure 151 and Figure 152 show that the highest proportion of GCCOP reports engaging with the climate change-health intersection in recent years has come from corporations based in the Western Pacific. Europe, the Americas, and South East Asia. The lowest engagement comes from corporations based in the Eastern Mediterranean region.

Different sectors are considered here too. **Table 75** shows the total number of references to climate change, health, and the intersection across the different sectors in 2020. Figure 153 presents the proportion of corporations engaging with the climate change-health relations in each sector in 2020.

	Intersection	Climate	Health
Aerospace & Defense	302	2229	5663
Alternative Energy	406	5106	5767
Automobiles & Parts	615	10133	17018
Banks	599	11899	19309
Basic Resources	0	3	12
Beverages	588	7181	16830
Chemicals	1949	13618	39019
Construction & Materials	1534	20551	52187
Diversified	1077	11572	26848
Electricity	998	18488	29181
Electronic & Electrical Equ...	482	6754	15831
Equity Investment Instruments	91	836	2037

Financial Services	1890	33341	41706
Fixed Line Telecommunications	301	3439	8692
Food & Beverage	0	16	86
Food & Drug Retailers	207	2241	6181
Food Producers	1974	13588	58982
Forestry & Paper	324	6482	7969
Gas, Water & Multiutilities	621	10435	17877
General Industrials	1872	22249	51169
General Retailers	582	11009	22639
Health Care	0	7	50
Health Care Equipment & Ser...	728	3377	40035
Household Goods & Home Cons...	348	6133	11825
Industrial Engineering	584	6746	15290
Industrial Goods & Services	0	65	762
Industrial Metals & Mining	784	9547	25286
Industrial Transportation	673	8714	17516
Leisure Goods	64	1905	3299
Life Insurance	273	1937	10419
Media	194	4414	9876
Mining	415	4114	12968
Mobile Telecommunications	465	5805	11466
Nonequity Investment Instru...	25	185	517
Nonlife Insurance	238	1936	8324
Not Applicable	189	3333	14349
Oil & Gas	2	7	36
Oil & Gas Producers	1487	18314	32126
Oil Equipment, Services & D...	232	2652	6897
Other	0	7	22
Personal Goods	378	4662	11914
Pharmaceuticals & Biotechno...	1696	7120	63263
Real Estate	17	57	118
Real Estate Investment & Se...	526	7436	12965
Real Estate Investment Trusts	313	2257	2799

Retail	3	47	397
Software & Computer Services	670	9242	19063
Support Services	1488	20429	53867
Technology	0	6	32
Technology Hardware & Equip...	692	11553	20338
Tobacco	2	48	137
Travel & Leisure	496	8421	15736

Table 75: Total number of references to the intersection of climate change and health by sector in 2021.

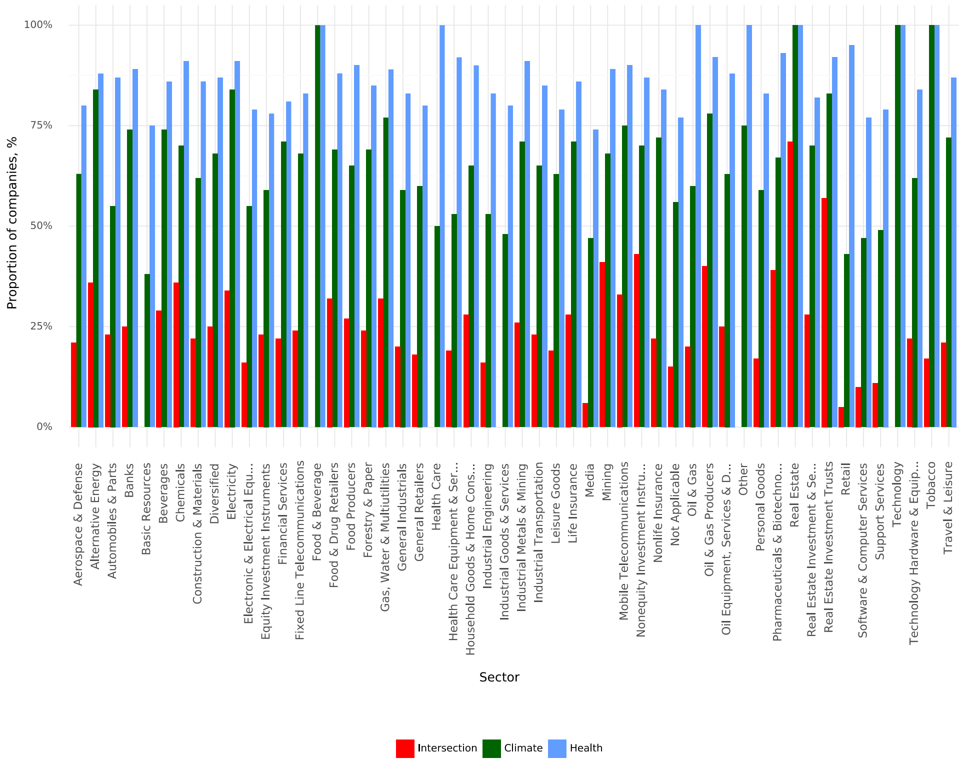

Figure 153: Proportion of corporations referring to climate change, health, and the intersection of climate change and health by sector in 2021.

The highest level of engagement with the intersection of climate change and health in 2021 can be seen in real estate, mobile telecommunications, mining, and oil and gas producers. In contrast, surprisingly, much lower levels of engagement can be seen in the healthcare sector, where only 19% of companies refer to the intersection of health and climate change in their 2021 GCCOP report.

In addition to looking at companies by WHO region, the analysis also considers companies from different types of countries in terms of their potential importance and role in addressing issues related to climate change. This is provided in Figure 156 and Figure 155. As noted in previous years' reports, the SIDS have driven much of the engagement with the health impacts of climate change, as well as climate change more generally, in the UN General Assembly. As such, a SIDS grouping is included. Arguably the three most important countries/unions in addressing climate change are USA, China, and the EU, referred to here as Tier 1 countries in Figure 154 and Figure 155. Finally, an additional grouping of countries are used here that are also important in terms of their CO_2 emissions, their influence in international politics, and their potential impact on addressing climate change. This grouping, referred to as Tier 2, countries: Poland, Australia, South Africa, Brazil, India, France, Germany, and Indonesia. Hence, the analysis looks at companies based on the type of country in which they are based in Figure 154 (total references) and Figure 155 (proportion of companies). The results in Figure 154 show that the

highest total references to the intersection of climate change and health tends to come from companies based in Tier 2 countries, and the lowest from those based in the SIDS. However, this is likely to reflect the vastly different numbers of companies that have signed up to the UN Global Compact from these regions. Figure 155 shows that in terms of the proportion of the companies that engage with health and climate change, the highest engagement is seen from companies based in SIDS, followed by those based in Tier 1 countries, with companies based in Tier 2 countries having the lowest engagement.

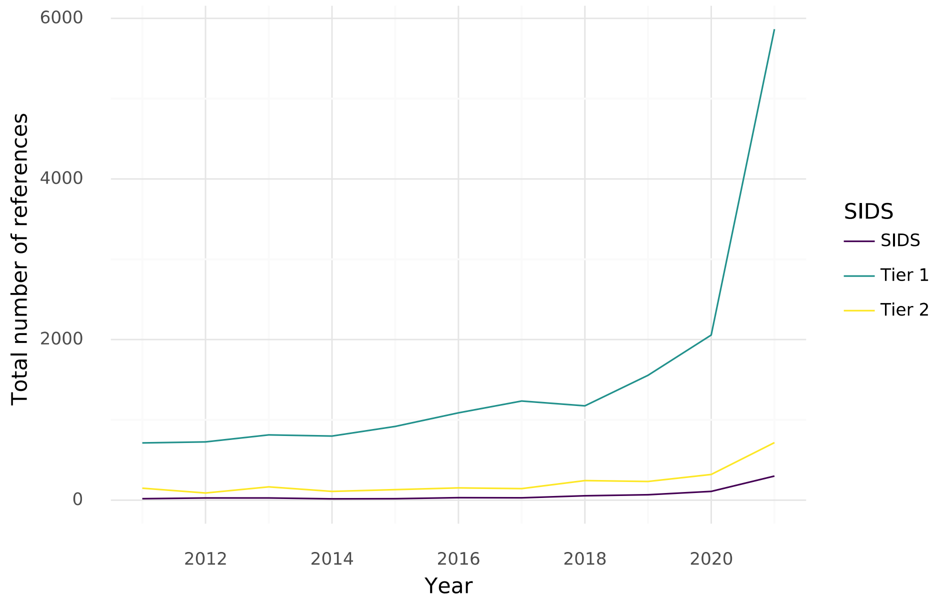

Figure 154: Total references to the climate change-health intersection by SIDS, Tier 1 countries, and Tier 2 countries, 2011–2021.

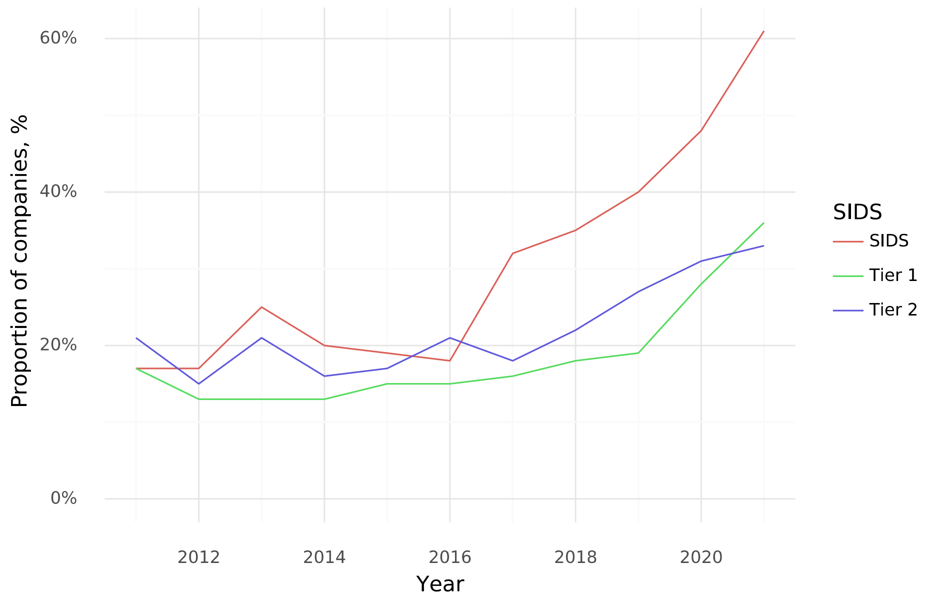

Figure 155: Proportion of corporations referring to the climate change-health intersection by SIDS, Tier 1 countries, and Tier 2 countries, 2011–2021.

The analysis also considers corporate engagement with the health dimensions of climate according to the Human Development Index (HDI) categories of the countries in which companies are based. Figure 156 shows the total references to the intersection of climate change and health in companies' GCCOP reports based on the country HDI category and Figure 157 shows the proportion of companies engaging with climate change and health in their GCCOP report by HDI category. Figure 156 shows significantly higher references to climate change and health made by countries based in countries that have very high human development compared to companies based in countries with other levels of human development. However, this reflects the fact that the majority of companies included in the analysis are based in countries with very high human development levels. It is worth noting that even when the proportion of companies that engage with climate change and health is considered (Figure 157), it is the companies based in countries with very high human development that have highest engagement, followed by those with a medium HDI; lower engagement with climate change and health is seen by companies based in countries with low human development levels.

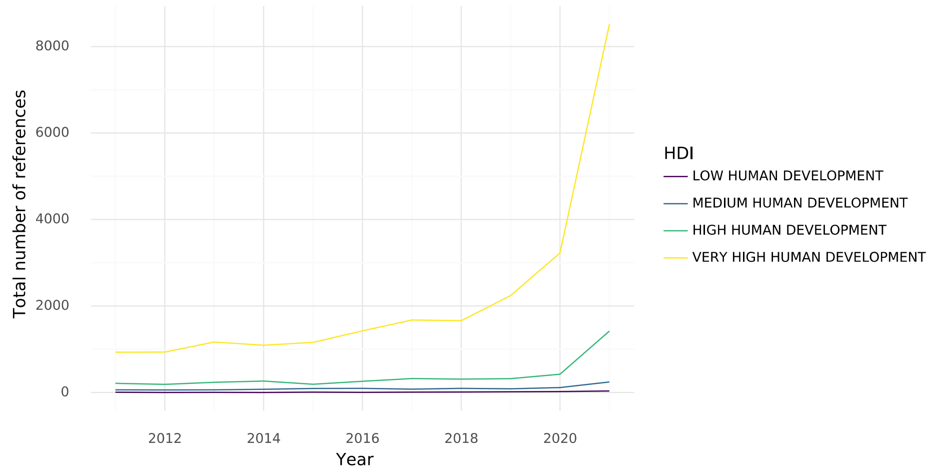

Figure 156: Total references to the climate change-health intersection by country HDI categories, 2011–2021.

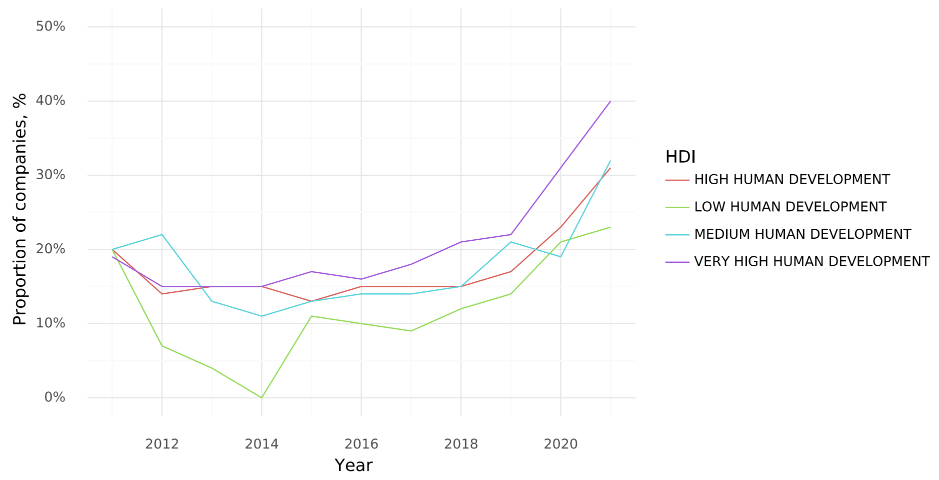

Figure 157: Figure H: Proportion of corporations referring to the climate change-health intersection by country HDI categories, 2011–2020.

References

1. Masson-Delmotte V, P. Zhai, A. Pirani, S.L. Connors, C. Péan, S. Berger, N. Caud, Y. Chen, L. Goldfarb, M.I. Gomis, M. Huang, K. Leitzell, E. Lonnoy, J.B.R. Matthews, T.K. Maycock, T. Waterfield, O. Yelekçi, R. Yu, and B. Zhou. IPCC, 2021: Climate Change 2021: The Physical Science Basis. Contribution of Working Group I to the Sixth Assessment Report of the Intergovernmental Panel on Climate Change, 2021.
2. NASA Socioeconomic Data and Applications Center (SEDAC) Gridded Population of the World (GPWv4). Available at https://beta.sedac.ciesin.columbia.edu/data/collection/gpw-v4. 2021.
3. ISIMIP. The Inter-Sectoral Impact Model Intercomparison Project (ISIMP). Input data set: Historical, gridded population. Available at https://www.isimip.org/gettingstarted/input-data-bias-correction/details/31/. 2021.
4. de Perez EC, van Aalst M, Bischiniotis K, et al. Global predictability of temperature extremes. *Environmental Research Letters* 2018; 13(5).
5. Di Napoli C, Pappenberger F, Cloke HL. Verification of Heat Stress Thresholds for a Health-Based Heat-Wave Definition. *Journal of Applied Meteorology and Climatology* 2019; 58(6): 1177-94.
6. Xu Z, Sheffield PE, Su H, Wang X, Bi Y, Tong S. The impact of heat waves on children's health: a systematic review. *Int J Biometeorol* 2014; 58(2): 239-47.
7. Chambers J. Global and cross-country analysis of exposure of vulnerable populations to heatwaves from 1980 to 2018. *Climatic Change* 2020; 163(1): 539-58.
8. Chambers J. Chambers, J. Hybrid gridded demographic data for the world, 1950-2020 0.25° resolution. Available at https://doi.org/10.5281/zenodo.6011021. 2022.
9. Hersbach H, Bell B, Berrisford P, et al. ERA5 monthly averaged data on single levels from 1979 to present. Copernicus Climate Change Service (C3S) Climate Data Store (CDS). Available at https://doi.org/10.24381/cds.f17050d7. 2019.
10. Nations U. United Nations. 2019 Revision of World Population Prospects. Available at https://population.un.org/wpp/. 2020.
11. Romanello M, McGushin A, Di Napoli C, et al. The 2021 report of the Lancet Countdown on health and climate change: code red for a healthy future. *The Lancet* 2021; 398(10311): 1619-62.
12. Jay O, Broderick C, Smallcombe J. Extreme heat policy. Available at https://sma.org.au/sma-site-content/uploads/2021/02/SMA-Extreme-Heat-Policy-2021-Final.pdf, 2021.
13. Vanos JK, Baldwin JW, Jay O, Ebi KL. Simplicity lacks robustness when projecting heat-health outcomes in a changing climate. *Nature Communications* 2020; 11(1): 1-5.
14. Kjellstrom T, Freyberg C, Lemke B, Otto M, Briggs D. Estimating population heat exposure and impacts on working people in conjunction with climate change. *International Journal of Biometeorology* 2018; 62(3): 291-306.
15. Liljegren JC, Carhart RA, Lawday P, Tschopp S, Sharp R. Modeling the wet bulb globe temperature using standard meteorological measurements. *Journal of occupational and environmental hygiene* 2008; 5(10): 645-55.
16. ILO. ILOSTAT database. Geneva, Switzerland: International Labour Organization; 2021.
17. Honda Y, Kondo M, McGregor G, et al. Heat-related mortality risk model for climate change impact projection. *Environ Health Prev Med* 2014; 19(1): 56-63.

18. Institute for Health Metrics and Evaluation (IHME) Global Health Data Exchange (GHDx). Global Burden of Disease. Available at http://ghdx.healthdata.org/gbd-results-tool. 2021.
19. United Nations. 2020 Revision of World Population Prospects. Available at https://population.un.org/wpp/. 2021.
20. Global Burden of Disease Collaborative Network. Global Burden of Disease Study 2019 (GBD 2019) Results. Seattle, United States: Institute for Health Metrics and Evaluation (IHME), 2020. Available from http://ghdx.healthdata.org/gbd-results-tool.
21. Vautard R, Aalst vM, Boucher O, et al. Human contribution to the record-breaking June and July 2019 heatwaves in Western Europe. *Environmental Research Letters* 2020; 15(9): 094077.
22. Ma F, Yuan X, Jiao Y, Ji P. Unprecedented Europe Heat in June–July 2019: Risk in the Historical and Future Context. *Geophysical Research Letters* 2020; 47(11): e2020GL087809.
23. Abram NJ, Henley BJ, Sen Gupta A, et al. Connections of climate change and variability to large and extreme forest fires in southeast Australia. *Commun Earth Environ* 2021; 2(1): 1-17.
24. Herring SC, Christidis N, Hoell A, Hoerling MP, Stott PA. Explaining Extreme Events of 2019 from a Climate Perspective. *Bulletin of the American Meteorological Society* 2021; 102(1): S1-S115.
25. Usaid, Uclouvain, Center for Research on the Epidemiology of D. Disaster* Year in Review 2019, 2020.
26. Kam J, Min S-K, Kim Y-H, Kim B-H, Kug J-S. Anthropogenic Contribution to the Record-Breaking Warm and Wet Winter 2019/20 over Northwest Russia. *Bulletin of the American Meteorological Society* 2022; 103(3): S38-S43.
27. Christidis N, Stott PA. Anthropogenic Climate Change and the Record-High Temperature of May 2020 in Western Europe. *Bulletin of the American Meteorological Society* 2022; 103(3): S33-S7.
28. Pascal M, Lagarrigue R, Laaidi K, Boulanger G, Denys S. Have health inequities, the COVID-19 pandemic and climate change led to the deadliest heatwave in France since 2003? *Public Health* 2021; 194: 143-5.
29. Green M. Heatwaves caused record deaths as Britain struggled with coronavirus: study. Reuters. 2020 2020/11/19.
30. Sousa PM, Trigo RM, Russo A, et al. Heat-related mortality amplified during the COVID-19 pandemic. *International Journal of Biometeorology* 2022; 66(3): 457-68.
31. Ciavarella A, Cotterill D, Kew S, et al. Prolonged Siberian heat of 2020. 2020: 35.
32. Min S-K, Jo S-Y, Seong M-G, et al. Human Contribution to the 2020 Summer Successive Hot-Wet Extremes in South Korea. *Bulletin of the American Meteorological Society* 2022; 103(3): S90-S7.
33. Philip SY, Kew SF, van Oldenborgh GJ, et al. Rapid attribution analysis of the extraordinary heatwave on the Pacific Coast of the US and Canada June 2021, 2021.
34. British Columbia Government News. Chief coroner's updated statement on public safety during heat wave. Friday, July 2, 2021 2021. https://news.gov.bc.ca/releases/2021PSSG0062-001295#:~:text=Lisa%20Lapointe%2C%20chief%20coroner%2C%20has,heat%20wave%20in%20British%20Columbia%3A&text=%E2%80%9CWe%20are%20releasing%20this%20information,the%20increased%20number%20of%20deaths.

35. Ministry of Public S, Solicitor General- British C. Chief coroner's statement on public safety during high temperatures. British Colombia, 2021.
36. Floodlist N. China – Floods Leave 88 Dead and 388,000 Displaced – FloodList. Floodlist. 2019 2019/06/17.
37. Whan C. Ottawa police confirm man missing since May found dead. Globalnewsca. 2019 2019/10/02.
38. Hoell A, Quan X-W, Hoerling M, et al. Record Low North American Monsoon Rainfall in 2020 Reignites Drought over the American Southwest. *Bulletin of the American Meteorological Society* 2022; 103(3): S26-S32.
39. Pei L, Yan Z, Chen D, Miao S. The Contribution of Human-Induced Atmospheric Circulation Changes to the Record-Breaking Winter Precipitation Event over Beijing in February 2020. *Bulletin of the American Meteorological Society* 2022; 103(3): S55-S60.
40. Tang H, Wang Z, Tang B, et al. Reduced Probability of 2020 June–July Persistent Heavy Mei-yu Rainfall Event in the Middle to Lower Reaches of the Yangtze River Basin under Anthropogenic Forcing. *Bulletin of the American Meteorological Society* 2022; 103(3): S83-S9.
41. Ma Y, Hu Z, Meng X, Liu F, Dong W. Was the Record-Breaking Mei-yu of 2020 Enhanced by Regional Climate Change? *Bulletin of the American Meteorological Society* 2022; 103: S76-S82.
42. Wang C, Yao Y, Wang H, Sun X, Zheng J. The 2020 Summer Floods and 2020/21 Winter Extreme Cold Surges in China and the 2020 Typhoon Season in the Western North Pacific. *Adv Atmos Sci* 2021; 38(6): 896-904.
43. Kreienkamp F, Philip SY, Tradowsky JS, et al. Rapid attribution of heavy rainfall events leading to the severe flooding in Western Europe during July 2021. *World Weather Attribution* 2021.
44. Liu Y, Zhu J, Shao X, Adusumilli NC, Wang F. Diffusion patterns in disaster-induced internet public opinion: based on a Sina Weibo online discussion about the 'Liangshan fire' in China. *Environmental Hazards* 2021; 20(2): 163-87.
45. van Oldenborgh GJ, Krikken F, Lewis S, et al. Attribution of the Australian bushfire risk to anthropogenic climate change. *Nat Hazards Earth Syst Sci Discuss* 2020; 2020: 1-46.
46. Arriagada NB, Palmer AJ, Bowman DMJS, Morgan GG, Jalaludin BB, Johnston FH. Unprecedented smoke-related health burden associated with the 2019–20 bushfires in eastern Australia. *Med J Aust* 2020; 213(6).
47. Liu Z, Eden J, Dieppois B, Drobyshev I, Gallo C, Blackett M. Were Meteorological Conditions Related to the 2020 Siberia Wildfires Made More Likely by Anthropogenic Climate Change? *Bulletin of the American Meteorological Society* 2021; 103.
48. Mercy C. The facts: Hurricane Dorian's devastating effect on The Bahamas. Mercy Corps; 2019.
49. 75yo woman dies after being thrown 30 metres as Typhoon Lingling hits Korean Peninsula. ABC News. 2019 2019/09/07.
50. Floodlist N. South Korea – Torrential Rain from Typhoon Mitag Causes Deadly Floods and Landslides. 2019 2019/10/03.
51. Otto FEL, Wolski P, Lehner F, et al. Anthropogenic influence on the drivers of the Western Cape drought 2015–2017. *Environmental Research Letters* 2018; 13(12): 124010.
52. Pascale S, Kapnick SB, Delworth TL, Cooke WF. Increasing risk of another Cape Town "Day Zero" drought in the 21st century. *Proceedings of the National Academy of Sciences* 2020; 117(47): 29495-503.

53. Wilson AM, Jetz W. Remotely sensed high-resolution global cloud dynamics for predicting ecosystem and biodiversity distributions. *PLoS biology* 2016; 14(3): e1002415.
54. Pechony O, Shindell DT. Driving forces of global wildfires over the past millennium and the forthcoming century. *Proceedings of the National Academy of Sciences* 2010; 107(45): 19167-70.
55. Sofiev M. Wildland Fires: Monitoring, Plume Modelling, Impact on Atmospheric Composition and Climate. Developments in Environmental Science: Elsevier; 2013: 451-72.
56. Burke M, Driscoll A, Heft-Neal S, Xue J, Burney J, Wara M. The changing risk and burden of wildfire in the United States. *Proceedings of the National Academy of Sciences* 2021; 118(2).
57. Khabarov N, Krasovskii A, Obersteiner M, et al. Forest fires and adaptation options in Europe. *Regional Environmental Change* 2016; 16(1): 21-30.
58. Granier C, Bessagnet B, Bond T, et al. Evolution of anthropogenic and biomass burning emissions of air pollutants at global and regional scales during the 1980–2010 period. *Climatic Change* 2011; 109(1): 163-90.
59. Liu JC, Pereira G, Uhl SA, Bravo MA, Bell ML. A systematic review of the physical health impacts from non-occupational exposure to wildfire smoke. *Environmental research* 2015; 136: 120-32.
60. Reid CE, Brauer M, Johnston FH, Jerrett M, Balmes JR, Elliott CT. Critical review of health impacts of wildfire smoke exposure. *Environmental health perspectives* 2016; 124(9): 1334-43.
61. Sofiev M, Vankevich R, Lotjonen M, et al. An operational system for the assimilation of the satellite information on wild-land fires for the needs of air quality modelling and forecasting. *Atmospheric Chemistry and Physics* 2009; 9(18): 6833-47.
62. Sofiev M, Vankevich R, Ermakova T, Hakkarainen J. Global mapping of maximum emission heights and resulting vertical profiles of wildfire emissions. *Atmospheric Chemistry and Physics* 2013; 13(14): 7039-52.
63. Sofiev M, Ermakova T, Vankevich R. Evaluation of the smoke-injection height from wild-land fires using remote-sensing data. *Atmospheric Chemistry and Physics* 2012; 12(4): 1995-2006.
64. Soares J, Sofiev M, Hakkarainen J. Uncertainties of wild-land fires emission in AQMEII phase 2 case study. *Atmospheric Environment* 2015; 115: 361-70.
65. Sofiev M, Vira J, Kouznetsov R, Prank M, Soares J, Genikhovich E. Construction of an Eulerian atmospheric dispersion model based on the advection algorithm of M. Galperin: dynamic cores v. 4 and 5 of SILAM v. 5.5. *Geoscientific Model Development Discussions* 2015; 8(3).
66. Kollanus V, Prank M, Gens A, et al. Mortality due to vegetation fire–originated PM2. 5 exposure in Europe—assessment for the years 2005 and 2008. *Environmental health perspectives* 2017; 125(1): 30-7.
67. Champeaux JL, Masson V, Chauvin F. ECOCLIMAP: a global database of land surface parameters at 1 km resolution. *Meteorological Applications: A journal of forecasting, practical applications, training techniques and modelling* 2005; 12(1): 29-32.
68. NASA. Fire Information for Resource Management System (FIRMS). Available at https://earthdata.nasa.gov/earth-observation-data/near-real-time/firms. 2022.
69. EarthEnv. Global 1-km Cloud Cover. Available at https://www.earthenv.org/cloud. 2022.

70. Copernicus Climate Change Service (C3S). Fire danger indices historical data from the Copernicus Emergency Management Service. Available at https://doi.org/10.24381/cds.0e89c522. 2021.
71. CNMR. ECOCLIMAP. Available at https://opensource.umr-cnrm.fr/projects/ecoclimap.
72. Maier SW, Russell-Smith J, Edwards AC, Yates C. Sensitivity of the MODIS fire detection algorithm (MOD14) in the savanna region of the Northern Territory, Australia. *ISPRS journal of photogrammetry and remote sensing* 2013; 76: 11-6.
73. Van Wagner C, Pickett T. Equations and FORTRAN program for the Canadian forest fire weather index system; 1985.
74. Di Giuseppe F, Pappenberger F, Wetterhall F, et al. The potential predictability of fire danger provided by numerical weather prediction. *Journal of Applied Meteorology and Climatology* 2016; 55(11): 2469-91.
75. Thonicke K, Cramer W. Long-term trends in vegetation dynamics and forest fires in Brandenburg (Germany) under a changing climate. *Natural Hazards* 2006; 38(1): 283-300.
76. Beguería S, Vicente-Serrano SM, Reig F, Latorre B. Standardized precipitation evapotranspiration index (SPEI) revisited: parameter fitting, evapotranspiration models, tools, datasets and drought monitoring. *International Journal of Climatology* 2014; 34(10): 3001-23.
77. Fan Y, Van den Dool H. A global monthly land surface air temperature analysis for 1948–present. *Journal of Geophysical Research: Atmospheres* 2008; 113(D1).
78. Schamm K, Ziese M, Becker A, et al. Global gridded precipitation over land: a description of the new GPCC First Guess Daily product. *Earth System Science Data* 2014; 6(1): 49-60.
79. Global SPEI database. Available at https://spei.csic.es/database.html. 2021.
80. MeteoSwiss. SPI and SPEI. 2018. https://www.meteoswiss.admin.ch/home/climate/swiss-climate-in-detail/climate-indicators/drought-indices/spi-and-spei.html (accessed March 2022).
81. Baylis P, Obradovich N, Kryvasheyeu Y, et al. Weather impacts expressed sentiment. *PLoS One* 2018; 13(4): e0195750.
82. Baylis P. Temperature and temperament: Evidence from Twitter. *Journal of Public Economics* 2020; 184: 104161.
83. Wang J, Obradovich N, Zheng S. A 43-Million-Person Investigation into Weather and Expressed Sentiment in a Changing Climate. *One Earth* 2020; 2(6): 568-77.
84. Noelke C, McGovern M, Corsi DJ, et al. Increasing ambient temperature reduces emotional well-being. *Environ Res* 2016; 151: 124-9.
85. Burke M, González F, Baylis P, et al. Higher temperatures increase suicide rates in the United States and Mexico. *Nature Climate Change* 2018; 8(8): 723-9.
86. Moore FC, Obradovich N, Lehner F, Baylis P. Rapidly declining remarkability of temperature anomalies may obscure public perception of climate change. *Proc Natl Acad Sci U S A* 2019; 116(11): 4905-10.
87. Carleton TA, Hsiang SM. Social and economic impacts of climate. *Science* 2016; 353(6304): aad9837.
88. Hsiang S. Climate Econometrics. *Annual Review of Resource Economics* 2016; 8(1): 43-75.

89. Diener E, Larsen RJ, Levine S, Emmons RA. Intensity and frequency: dimensions underlying positive and negative affect. *Journal of personality and social psychology* 1985; 48(5): 1253.
90. Pennebaker JW, Boyd RL, Jordan K, Blackburn K. The development and psychometric properties of LIWC2015, 2015.
91. Pennebaker JW, Chung C, Ireland M, Gonzales A, Booth R. The development and psychometric properties of LIWC2007, 2007.
92. Tausczik YR, Pennebaker JW. The Psychological Meaning of Words: LIWC and Computerized Text Analysis Methods. *Journal of Language and Social Psychology* 2009; 29(1): 24-54.
93. Coppersmith G. Quantifying Mental Health Signals in Twitter. Proceedings of the Workshop on Computational Linguistics and Clinical Psychology: From Linguistic Signal to Clinical Reality; 2014; Baltimore, Maryland, USA; 2014. p. 51-60.
94. Kahn JH, Tobin RM, Massey AE, Anderson JA. Measuring emotional expression with the Linguistic Inquiry and Word Count. *Am J Psychol* 2007; 120(2): 263-86.
95. Beasley A, Mason W. Emotional States vs. Emotional Words in Social Media. Proceedings of the ACM Web Science Conference. Oxford, United Kingdom: Association for Computing Machinery; 2015. p. Article 31.
96. Settanni M, Marengo D. Sharing feelings online: studying emotional well-being via automated text analysis of Facebook posts. *Front Psychol* 2015; 6: 1045-.
97. Hersbach H, Bell B, Berrisford P, et al. The ERA5 global reanalysis. *Quarterly Journal of the Royal Meteorological Society* 2020; 146(730): 1999-2049.
98. Myhre G, Alterskjær K, Stjern CW, et al. Frequency of extreme precipitation increases extensively with event rareness under global warming. *Scientific Reports* 2019; 9(1): 16063.
99. Dunn RJH, Alexander LV, Donat MG, et al. Development of an Updated Global Land In Situ-Based Data Set of Temperature and Precipitation Extremes: HadEX3. *Journal of Geophysical Research: Atmospheres* 2020; 125(16): e2019JD032263.
100. Contractor S. D, M.G., Alexander, L.V. Changes in Observed Daily Precipitation overGlobal Land Area since 1950. *Journal of Climate* 2021; 34: 3-19.
101. Alexander LV. Global observed long-term changes in temperature and precipitation extremes: A review of progress and limitations in IPCC assessments and beyond. *Weather and Climate Extremes* 2016; 11: 4-16.
102. Obradovich N, Tingley D, Rahwan I. Effects of environmental stressors on daily governance. *Proc Natl Acad Sci U S A* 2018; 115(35): 8710-5.
103. Obradovich N, Migliorini R, Mednick SC, Fowler JH. Nighttime temperature and human sleep loss in a changing climate. *Sci Adv* 2017; 3(5): e1601555.
104. Obradovich N, Rahwan I. Risk of a feedback loop between climatic warming and human mobility. *J R Soc Interface* 2019; 16(158): 20190058.
105. Minor K, Bjerre-Nielsen, A., Jonasdottir S.S., Lehmann, S., Obradovich, N. Ambient heat and human sleep. ArXiv201107161 Cs Econ Q-Fin, 2020.
106. Programme UND. Human Development Report 2020: The Next Frontier, Human Development and the Anthropocene. United Nations Development Programme, New York: United Nations Development Programme, 2020.
107. Copernicus Climate Change Service (C3S). ERA5 hourly data on single levels from 1979 to present. Available at https://doi.org/10.24381/cds.adbb2d47. 2021.
108. Organization WM. June ends with exceptional heat. 2021 30 June 2021.

109. Philip SY, Kew, S. F., van Oldenborgh, G. J., Anslow, F. S., Seneviratne, S. I., Vautard, R., Coumou, D., Ebi, K. L., Arrighi, J., Singh, R., van Aalst, M., Pereira Marghidan, C., Wehner, M., Yang, W., Li, S., Schumacher, D. L., Hauser, M., Bonnet, R., Luu, L. N., Lehner, F., Gillett, N., Tradowsky, J., Vecchi, G. A., Rodell, C., Stull, R. B., Howard, R., and Otto, F. E. L. Rapid attribution analysis of the extraordinary heatwave on the Pacific Coast of the US and Canada June 2021. *Earth Syst Dynam* 2021 [preprint].
110. Popovic NaC-S, W. Hidden Toll of the NorthWest Heat Wave: Hundreds of Extra Deaths. The New York Times. 2021 11th August 2021.
111. Service BCCs. Heat-related Deaths in B.C.: British Columbia Coroner's Service, 2021.
112. Philip SY, Kew SF, van Oldenborgh GJ, et al. Rapid attribution analysis of the extraordinary heatwave on the Pacific Coast of the US and Canada June 2021. *Earth System Dynamics Discussions* 2021: 1-34.
113. Kreienkamp F, Philip SY, Tradowsky JS, et al. Rapid attribution of heavy rainfall events leading to the severe flooding in Western Europe during July 2021. 2021.
114. Stanaway JD, Shepard DS, Undurraga EA, et al. The global burden of dengue: an analysis from the Global Burden of Disease Study 2013. *Lancet Infect Dis* 2016; 16(6): 712-23.
115. Hales S, de Wet N, Maindonald J, Woodward A. Potential effect of population and climate changes on global distribution of dengue fever: an empirical model. *Lancet* 2002; 360(9336): 830-4.
116. Rocklöv JT, Y; . Climate change and the rising infectiousness of dengue. *Emerging Topics in Life Science* 2019; ETLS20180123.
117. Rocklöv J, Quam MB, Sudre B, et al. Assessing Seasonal Risks for the Introduction and Mosquito-borne Spread of Zika Virus in Europe. *EBioMedicine* 2016; 9: 250-6.
118. Rocklöv J, Tozan Y, Ramadona A, et al. Using Big Data to Monitor the Introduction and Spread of Chikungunya, Europe, 2017. *Emerg Infect Dis* 2019; 25(6): 1041-9.
119. Liu-Helmersson J, Brännström, Å., Sewe, M., Semenza, J., Rocklöv, J. Estimating past, present and future trends in the global distribution and abundance of the arbovirus vector Aedes aegypti under climate change scenarios. *Frontiers in Public Health* 2019.
120. DiSera L, Sjödin H, Rocklöv J, et al. The Mosquito, the Virus, the Climate: An Unforeseen Réunion in 2018. *Geohealth* 2020; 4(8): e2020GH000253.
121. Colón-González FJ, Sewe MO, Tompkins AM, et al. Projecting the risk of mosquito-borne diseases in a warmer and more populated world: a multi-model, multi-scenario intercomparison modelling study. *Lancet Planet Health* 2021; 5(7): e404-e14.
122. Muñoz-Sabater J, Dutra E, Agustí-Panareda A, et al. ERA5-Land: A state-of-the-art global reanalysis dataset for land applications. *Earth System Science Data* 2021; 13(9): 4349-83.
123. Muñoz Sabater J. ERA5-Land monthly averaged data from 1981 to present. Copernicus Climate Change Service (C3S) Climate Data Store (CDS). Available at https://doi.org/10.24381/cds.68d2bb30. 2019.
124. Klein Goldewijk K, Beusen A, Doelman J, Stehfest E. Anthropogenic land use estimates for the Holocene; HYDE 3.2. Available at https://doi.org/10.17026/dans-25g-gez3. 2017.
125. Liu-Helmersson J, Quam M, Wilder-Smith A, et al. Climate Change and Aedes Vectors: 21st Century Projections for Dengue Transmission in Europe. *EBioMedicine* 2016; 7: 267-77.

126. Liu-Helmersson J, Stenlund H, Wilder-Smith A, Rocklöv J. Vectorial capacity of Aedes aegypti: effects of temperature and implications for global dengue epidemic potential. *PLoS One* 2014; 9(3): e89783.
127. Alduchov OA, Eskridge RE. Improved Magnus form approximation of saturation vapor pressure. *Journal of Applied Meteorology and Climatology* 1996; 35(4): 601-9.
128. University of Washington Joint Institute for the Study of the Atmosphere and Ocean (JISAO). Elevation data. Available at http://research.jisao.washington.edu/data_sets/elevation/. 2020.
129. Copernicus Global Land Monitoring Service. Land Cover. Available at https://land.copernicus.eu/global/products/lc 2022.
130. Laporta GZ, Linton Y-M, Wilkerson RC, et al. Malaria vectors in South America: current and future scenarios. *Parasites & vectors* 2015; 8(1): 1-13.
131. Sinka ME, Rubio-Palis Y, Manguin S, et al. The dominant Anopheles vectors of human malaria in the Americas: occurrence data, distribution maps and bionomic précis. *Parasites & vectors* 2010; 3(1): 1-26.
132. Buchhorn M, Smets B, Bertels L, et al. Copernicus Global Land Service: Land Cover 100m: collection 3: epoch 2019: Globe (V3.0.1). Available at https://doi.org/10.5281/zenodo.3939050. 2020.
133. Gething PW, Van Boeckel TP, Smith DL, et al. Modelling the global constraints of temperature on transmission of Plasmodium falciparum and P. vivax. *Parasites & vectors* 2011; 4(1): 92.
134. Snow RW, Sartorius B, Kyalo D, et al. The prevalence of Plasmodium falciparum in sub-Saharan Africa since 1900. *Nature* 2017; 550(7677): 515-8.
135. Martinez-Urtaza J, van Aerle R, Abanto M, et al. Genomic Variation and Evolution of Vibrio parahaemolyticus ST36 over the Course of a Transcontinental Epidemic Expansion. *MBio* 2017; 8(6).
136. Muhling BA, Gaitán CF, Stock CA, Saba VS, Tommasi D, Dixon KW. Potential Salinity and Temperature Futures for the Chesapeake Bay Using a Statistical Downscaling Spatial Disaggregation Framework. *Estuaries and Coasts* 2017; 41(2): 349-72.
137. Parveen S, Hettiarachchi KA, Bowers JC, et al. Seasonal distribution of total and pathogenic Vibrio parahaemolyticus in Chesapeake Bay oysters and waters. *International journal of food microbiology* 2008; 128(2): 354-61.
138. Copernicus Marine Environment Monitoring Service. Mercator Ocean Reanalysis. Available at http://marine.copernicus.eu/. 2021.
139. NOAA Earth System Research Laboratory. Optimum Interpolation 1/4 Degree Daily Sea Surface Temperature Analysis version 2. Available at https://www.ncdc.noaa.gov/oisst. 2021.
140. Colwell R, Huq A. Marine ecosystems and cholera. *Hydrobiologia* 2001; 460: 141-5.
141. Clemens JD, Nair GB, Ahmed T, Qadri F, Holmgren J. Cholera. *The Lancet* 2017; 390(10101): 1539-49.
142. Ali M, Nelson AR, Lopez AL, Sack DA. Updated global burden of cholera in endemic countries. *PLoS neglected tropical diseases* 2015; 9(6): e0003832.
143. Escobar LE, Morand S. Editorial: Disease Ecology and Biogeography. *Frontiers in Veterinary Science* 2021; 8.
144. Escobar LE. Ecological niche modeling: an introduction for veterinarians and epidemiologists. *Frontiers in Veterinary Science* 2020: 713.

145. Escobar LE, Qiao H, Lee C, Phelps NBD. Novel Methods in Disease Biogeography: A Case Study with Heterosporosis. *Front Vet Sci* 2017; 4: 105.
146. Cobos ME, Jiménez L, Nuñez-Penichet C, Romero-Alvarez D, Simões M. Sample data and training modules for cleaning biodiversity information. *Biodiversity Informatics* 2018; 13: 49-50.
147. Blonder B, Morrow CB, Maitner B, et al. New approaches for delineating n-dimensional hypervolumes. *Methods in Ecology and Evolution* 2018; 9(2): 305-19.
148. Castaneda-Guzman M, Mantilla-Saltos G, Murray KA, Settlage R, Escobar LE. A database of global coastal conditions. *Scientific data* 2021; 8(1): 1-8.
149. Castaneda-Guzman M, G. M-S, Murray KA, Settlage R, Escobar LE. A database of global coastal conditions. Available at https://doi.org/10.6084/m9.figshare.c.5660263.v1. 2021.
150. Rawlings TK, Ruiz GM, Colwell RR. Association of Vibrio cholerae O1 El Tor and O139 Bengal with the Copepods Acartia tonsa and Eurytemora affinis. *Applied and environmental microbiology* 2007; 73(24): 7926-33.
151. Challinor AJ, Koehler AK, Ramirez-Villegas J, Whitfield S, Das B. Current warming will reduce yields unless maize breeding and seed systems adapt immediately. *Nature Climate Change* 2016; 6(10): 954-+.
152. Portmann FT, Siebert S, Döll P. MIRCA2000—Global monthly irrigated and rainfed crop areas around the year 2000: A new high-resolution data set for agricultural and hydrological modeling. *Global biogeochemical cycles* 2010; 24(1).
153. Vos T, Lim SS, Abbafati C, et al. Global burden of 369 diseases and injuries in 204 countries and territories, 1990–2019: a systematic analysis for the Global Burden of Disease Study 2019. *The Lancet* 2020; 396(10258): 1204-22.
154. Copernicus Climate Change Service (C3S). ORAS5 global ocean reanalysis monthly data from 1958 to present. Available at https://cds.climate.copernicus.eu/cdsapp#!/dataset/reanalysis-oras5. 2022.
155. National Oceanic and Atmospheric Administration (NOAA). Coral Reef Watch Version 3.1 Daily Global 5-km Satellite Coral Bleaching Degree Heating Week Product. 2021.
156. FAO. New Food Balance Sheets. Available at http://www.fao.org/faostat/en/#data/FBS. 2021.
157. Noaa. NOAA Coral Reef Watch Version 3.1 Daily Global 5-km Satellite Coral Bleaching Degree Heating Week Product. 2018.
158. Dasgupta S, Robinson EJZ. Attributing changes in food insecurity to a changing climate. *Sci Rep* 2022; 12(1): 4709.
159. Monfreda C, Ramankutty N., Foley J.A. Farming the planet: 2. Geographic distribution of crop areas, yields, physiological types, and net production in the year 2000. *Global biogeochemical cycles* 2008; 22(1).
160. Muñoz Sabater J. ERA5-Land hourly data from 1981 to present. Copernicus Climate Change Service (C3S) Climate Data Store (CDS). 2019.
161. Beguería S. Calculation of the Standardised Precipitation-Evapotranspiration Index. http://sac.csic.es/spei; 2017.
162. Dasgupta S, Robinson EJZ. Improving Food Policies for a Climate Insecure World: Evidence from Ethiopia. *National Institute Economic Review* 2021; 258: 66-82.
163. Cafiero C, Viviani S, Nord M. Food security measurement in a global context: The food insecurity experience scale. *Measurement* 2018; 116: 146-52.

164. World Health Organization. 2021 WHO health and climate change global survey report. Geneva, Switzerland, 2021.
165. Florczyk AJ, Melchiorri, M., Corbane, C., Schiavina, M., Maffenini, M., Pesaresi, M., Politis, p., Sabo, S., Freire, S., Ehrlich, D., Kemper, T., Tommasi, P., Airaghi, D., Zanchetta, L. Descriptio of the GHS Urban Centre Database 2015: Joint Research Centre, 2019.
166. Center For International Earth Science Information Network CIESIN-Columbia University. Gridded Population of the World, Version 4 (GPWv4): Population Density, Revision 11. 2017.
167. Kriegler FJ, Malila WA, Nalepka RF, Richardson W. Preprocessing Transformations and Their Effects on Multispectral Recognition. Proceedings of the Sixth International Symposium on Remote Sensing of Environment; 1969 1969; 1969. p. 97.
168. U. S. Geological Survey. U.S. Geological Survey Landsat Data. 2021.
169. Beck HE, Zimmermann NE, McVicar TR, Vergopolan N, Berg A, Wood EF. Present and future Koppen-Geiger climate classification maps at 1-km resolution. *Sci Data* 2018; 5: 180214.
170. Watts N, Adger WN, Ayeb-Karlsson S, et al. The Lancet Countdown: tracking progress on health and climate change. *The Lancet* 2017; 389(10074): 1151-64.
171. Watts N, Amann M, Arnell N, et al. The 2019 report of The Lancet Countdown on health and climate change: ensuring that the health of a child born today is not defined by a changing climate. *The Lancet* 2019; 394(10211): 1836-78.
172. Watts N, Amann M, Ayeb-Karlsson S, et al. The Lancet Countdown on health and climate change: from 25 years of inaction to a global transformation for public health. *The Lancet* 2017.
173. Watts N, Amann M, Arnell N, et al. The 2020 report of The *Lancet* Countdown on health and climate change: responding to converging crises. *The Lancet* 2021; 397(10269): 129-70.
174. kMatrix Ltd. Adaptation and Resilience to Climate Change dataset. Rutland, UK; 2021.
175. Jaikumar R. Postindustrial Manufacturing. Harvard Business Review. 1986 1986.
176. Georgeson L, Maslin, M., Poessinouw, M. The global green economy: a review of concepts, definitions, measurement methodologies and their interactions. *Geo: Geography and Environment* 2017; 4(1).
177. International Monetary F. World Economic Outlook Database. 2018.
178. Green Climate Fund. Project portfolio dashboard. In: GCF, editor.; 2022.
179. Climate Funds Update. Climate Funds Update Data Dashboard. 2022. https://climatefundsupdate.org/data-dashboard/ (accessed 3 May 2022).
180. Global Burden of Disease Collaborative Network. Reference Life Table: Institute for Health Metrics and Evaluation (IHME), 2022.
181. United Nations Population Division. Urban population (% of total population). World Urbanization Prospects: 2018 Revision. 2019 ed. New York: World Bank; 2018.
182. Centre for Research on the Epidemiology of Disasters. EM-DAT The International Disaster Database. Available at https://emdat.be/. 2021.
183. United Nations Development Programme. Human Development Reports. Human Development Index (HDI). Available at http://hdr.undp.org/en/content/human-development-index-hdi. 2021.

184. Kopp RE, DeConto RM, Bader DA, et al. Evolving Understanding of Antarctic Ice-Sheet Physics and Ambiguity in Probabilistic Sea-Level Projections. *Earth's Future* 2017; 5(12): 1217-33.
185. Kulp SA, Strauss BH. CoastalDEM: a global coastal digital elevation model improved from SRTM using a neural network. *Remote sensing of environment* 2018; 206: 231-9.
186. Thomas MA, Lin T. Illustrative Analysis of Probabilistic Sea Level Rise Hazard. *Journal of Climate* 2020; 33(4): 1523-34.
187. McMichael C, Dasgupta S, Ayeb-Karlsson S, Kelman I. A review of estimating population exposure to sea-level rise and the relevance for migration. *Environmental Research Letters* 2020; 15(12).
188. Kulp SA, Strauss BH. New elevation data triple estimates of global vulnerability to sea-level rise and coastal flooding. *Nature communications* 2019; 10(1): 1-12.
189. Ayeb-Karlsson S, Kniveton D, Cannon T. Trapped in the prison of the mind: Notions of climate-induced (im)mobility decision-making and wellbeing from an urban informal settlement in Bangladesh. *Palgr Commun* 2020; 6(1).
190. Hauer ME, Fussell E, Mueller V, et al. Sea-level rise and human migration. *Nature Reviews Earth & Environment* 2020; 1(1): 28-39.
191. Duijndam SJ, Botzen, W.J.W., Hagedoorn, L.C., Aerts, J.C.J.H. Anticipating sea-level rise and human migration: A review of empirical evidence and avenues for future research. *Wiley Interdisciplinary Reviews: Climate Change* 2022; 13: e747.
192. Dannenberg AL, Frumkin H, Hess JJ, Ebi KL. Managed retreat as a strategy for climate change adaptation in small communities: public health implications. *Climatic Change* 2019; 153(1-2): 1-14.
193. McMichael C, Katonivualiku M, Powell T. Planned relocation and everyday agency in low-lying coastal villages in Fiji. *The Geographical Journal* 2019; 185(3): 325-37.
194. Abubakar I, Aldridge RW, Devakumar D, et al. The UCL–Lancet Commission on Migration and Health: the health of a world on the move. *The Lancet* 2018; 392(10164): 2606-54.
195. Kelman I. Difficult decisions: migration from small island developing states under climate change. *Earth's Future* 2015; 3(4): 133-42.
196. Kelman I. Islandness within climate change narratives of small island developing states (SIDS). *Island Studies Journal* 2018; 13(1): 149-66.
197. Kelman I. Imaginary Numbers of Climate Change Migrants? *Social Sciences* 2019; 8(5).
198. Ayeb-Karlsson S, Smith CD, Kniveton D. A discursive review of the textual use of ‘trapped’in environmental migration studies: The conceptual birth and troubled teenage years of trapped populations. *Ambio* 2018; 47(5): 557-73.
199. Baldwin A. Premediation and white affect: climate change and migration in critical perspective. *Transactions of the Institute of British Geographers* 2016; 41(1): 78-90.
200. IEA. CO2 Emissions from Fuel Combustion (2021 edition). UK Data Service; 2021.
201. IEA. Global Energy Review: CO2 Emissions in 2020. 2021. https://www.iea.org/articles/global-energy-review-co2-emissions-in-2020 (accessed 16/03/2022 2022).
202. IEA. Global Energy Review 2021. Paris, France: IEA, 2021.
203. IEA. CO2 Emissions From Fuel Combustion: Database Documentation (2021 Edition). 2022.

https://stats2.digitalresources.jisc.ac.uk/metadata/IEA/gge/Greenhouse_Gas_Emissions_from_Energy_documentation.pdf (accessed 16 March 2022 2022).
204. IEA. Greenhouse Gas Emissions from Energy. 2021. https://www.iea.org/data-and-statistics/data-product/greenhouse-gas-emissions-from-energy#ghg-emissions-from-fuel-combustion (accessed 16 March 2022 2022).
205. IEA. World Extended Energy Balances (2021 edition). UK Data Service; 2021.
206. IEA. World Energy Balances: Data Documentation (2021 edition). 2020. http://wds.iea.org/wds/pdf/WORLDBAL_Documentation.pdf). (accessed 31/3/2022 2022).
207. IEA. Methodology. Defining energy access. 2020. https://www.iea.org/articles/defining-energy-access-2020-methodology (accessed 31/3/2022 2022).
208. WHO. Indicator 7.1.2: Proportion of population with primary reliance on clean fuels and technology. 19 July 2016 2016. https://unstats.un.org/sdgs/metadata/files/Metadata-07-01-02.pdf (accessed 14 April 2021).
209. WHO. Global HAP database. Geneva: World Health Organization; 2022.
210. Stoner O, Lewis J, Martínez I, Gumy S, Economou T, Adair-Rohani H. What fuels the fire? Cooking fuel estimates at global, regional and country level for 1990-2030: In Review, 2021.
211. Stoner O, Shaddick G, Economou T, et al. Global household energy model: a multivariate hierarchical approach to estimating trends in the use of polluting and clean fuels for cooking. *Journal of the Royal Statistical Society: Series C (Applied Statistics)* 2020; 69(4): 815-39.
212. IEA. World Extended Energy Balances (2021 edition). UK Data Service; 2022.
213. Shupler M, Godwin W, Frostad J, Gustafson P, Arku RE, Brauer M. Global estimation of exposure to fine particulate matter (PM2. 5) from household air pollution. *Environment international* 2018; 120: 354-63.
214. Stoner O, Lewis J, Martínez IL, Gumy S, Economou T, Adair-Rohani H. Household cooking fuel estimates at global and country level for 1990 to 2030. *Nature Communications* 2021; 12(1): 5793.
215. Smith KR, Bruce N, Balakrishnan K, et al. Millions Dead: How Do We Know and What Does It Mean? Methods Used in the Comparative Risk Assessment of Household Air Pollution. *Annual Review of Public Health* 2014; 35(1): 185-206.
216. IEA. World Energy Outlook 2021. Paris, 2021.
217. World Health Organization. Household air pollution attributable death rate (per 100 000 population). Geneva; 2022.
218. Amann M, Kiesewetter G, Schöpp W, et al. Reducing global air pollution: the scope for further policy interventions. *Phil Trans R Soc A* 2020; 378: 20190331.
219. Klimont Z, Kupiainen K, Heyes C, et al. Global anthropogenic emissions of particulate matter including black carbon. *Atmospheric Chemistry and Physics* 2017; 17(14): 8681-723.
220. NASA. Earth Exchange Global Daily Downscaled Projections (NEX-GDDP). . 2022.
221. United Nations Development Programme. Human Development Reports. Human Development Index (HDI). New York: UNDP, 2021.
222. WHO. Household air pollution attributable death rate (per 100 000 population). Geneva: WHO, 2019.
223. G. B. D. Risk Factors Collaborators. Global burden of 87 risk factors in 204 countries and territories, 1990-2019: a systematic analysis for the Global Burden of Disease Study 2019. *Lancet (London, England)* 2020; 396(10258): 1223-49.

224. Global Burden of Disease Collaborative Network. Global Burden of Disease Study 2019 (GBD 2019) Particulate Matter Risk Curves. Institute for Health Metrics and Evaluation (IHME); 2021.
225. Institute for Health Metrics and Evaluation. GBD Results Tool. Seattle: IHME, 2019.
226. Du W, Wang J, Wang Z, et al. Influence of COVID-19 lockdown overlapping Chinese Spring Festival on household PM2.5 in rural Chinese homes. *Chemosphere* 2021; 278: 130406.
227. Shen H, Shen G, Chen Y, et al. Increased air pollution exposure among the Chinese population during the national quarantine in 2020. *Nature Human Behaviour* 2021; 5(2): 239-46.
228. Amann M, Bertok I, Borken-Kleefeld J, et al. Cost-effective control of air quality and greenhouse gases in Europe: Modeling and policy applications. *Environmental Modelling & Software* 2011; 26(12): 1489-501.
229. IEA. World Energy Outlook 2020. Paris: IEA, 2020.
230. Simpson D, Benedictow A, Berge H, et al. The EMEP MSC-W chemical transport model–technical description. *Atmospheric Chemistry and Physics* 2012; 12(16): 7825-65.
231. Kiesewetter G, Borken-Kleefeld J, Schöpp W, et al. Modelling street level PM 10 concentrations across Europe: source apportionment and possible futures. *Atmospheric Chemistry and Physics* 2015; 15(3): 1539-53.
232. Amann M, Kiesewetter G, Schöpp W, et al. Reducing global air pollution: the scope for further policy interventions. *Phil Trans R Soc A* 2020; 378(2183).
233. WHO. WHO Global Urban Ambient Air Pollution Database (update 2018). Geneva: World Health Organization, 2018.
234. Murray CJL, Aravkin AY, Zheng P, et al. Global burden of 87 risk factors in 204 countries and territories, 1990–2019: a systematic analysis for the Global Burden of Disease Study 2019. *The Lancet* 2020; 396(10258): 1223-49.
235. Global Burden of Disease Collaborative Network. Particulate Matter Risk Curves. Seattle: Institute for Health Metrics and Evaluation (IHME), 2021.
236. Forouzanfar MH, Alexander L, Anderson HR, et al. Global, regional, and national comparative risk assessment of 79 behavioural, environmental and occupational, and metabolic risks or clusters of risks in 188 countries, 1990–2013: a systematic analysis for the Global Burden of Disease Study 2013. *The Lancet* 2015; 386(10010): 2287-323.
237. IHME. GBD Results Tool, 2019.
238. UNDESA. World Population Prospects: The 2017 revision. New York, NY, USA: United Nations Department of Economic and Social Affairs, 2017.
239. Chen J, Hoek G. Long-term exposure to PM and all-cause and cause-specific mortality: A systematic review and meta-analysis. *Environment International* 2020; 143: 105974.
240. IEA. World Extended Energy Balances (2021 edition). Paris: IEA, 2021.
241. Alexandratos N, Bruinsma J. World agriculture towards 2030/2050. Rome: FAO, 2012.
242. IFA. International Fertiziler Association Database. Paris: IFA, 2022.
243. GBD Diet Collaborators. Health effects of dietary risks in 195 countries, 1990-2017: a systematic analysis for the Global Burden of Disease Study 2017. *Lancet (London, England)* 2019; 393(10184): 1958-72.
244. UNDESA. World Population Prospects 2019. 2019 ed: United Nations; 2019.

245. Woodcock J, Givoni M, Morgan AS. Health impact modelling of active travel visions for England and Wales using an Integrated Transport and Health Impact Modelling Tool (ITHIM). *PLoS One* 2013; 8(1): e51462.
246. Jan Kole P, Löhr AJ, Van Belleghem FGAJ, J. RAM. Wear and tear of tyres: A stealthy source of microplastics in the environment. *International Journal of Environmental Research and Public Health* 2017; 14(10).
247. Apple Inc. Mobility Trends Report. Cupertino: Apple Inc., 2022.
248. Dalin C, Tuninetti M, Carlson K, et al. Variability, drivers and interactions of key environmental stressors from food production worldwide. EGU2019; 2019; Vienna: 21st EGU General Assembly; 2019.
249. Herrero M, Havlík P, Valin H, et al. Biomass use, production, feed efficiencies, and greenhouse gas emissions from global livestock systems. *Proceedings of the National Academy of Sciences* 2013; 110(52): 20888-93.
250. Havlík P, Valin H, Herrero M, et al. Climate change mitigation through livestock system transitions. *Proceedings of the National Academy of Sciences* 2014; 111(10): 3709-14.
251. Cecile De Klein, Rafael S.A. Novoa SO, Keith A. Smith, et al. IPCC Guidelines for National Greenhouse Gas Inventories. Agriculture, Forestry and Other Land Use. Hayama: Institute for Global Environmental Strategies, 2006.
252. FAO. FAOSTAT. 2020. http://www.fao.org/faostat/ (accessed 12 Feb 2022 2022).
253. Chang J, Ciais P, Herrero M, et al. Combining livestock production information in a process-based vegetation model to reconstruct the history of grassland management. *Biogeosciences* 2016; 13(12): 3757-76.
254. Carlson KM, Gerber JS, Mueller ND, et al. Greenhouse gas emissions intensity of global croplands. *Nature Climate Change* 2017; 7(1): 63-8.
255. Dalin C, Wada Y, Kastner T, Puma MJ. Groundwater depletion embedded in international food trade. *Nature* 2017; 543: 700–4.
256. Kastner T, Kastner M, Nonhebel S. Tracing distant environmental impacts of agricultural products from a consumer perspective. *Ecological Economics* 2011; 70(6): 1032–40.
257. Mbow C, C. Rosenzweig, L.G. Barioni, et al. Food Security. In: Climate Change and Land: an IPCC special report on climate change, desertification, land degradation, sustainable land management, food security, and greenhouse gas fluxes in terrestrial ecosystems. Geneva: IPCC, 2019.
258. Chen C, Chaudhary A, Mathys A. Nutritional and environmental losses embedded in global food waste. *Resources, Conservation and Recycling* 2020; 160: 104912.
259. Poore J, Nemecek T. Reducing food's environmental impacts through producers and consumers. *Science* 2018; (360): 987–92.
260. Food, Agriculture Organization of the United N. Food balance sheets: a handbook. Rome; 2001.
261. Gustavsson J, Cederberg C, Sonesson U, Van Otterdijk R, Meybeck A. Global food losses and food waste: extent, causes and prevention: FAO Rome, 2011.
262. Miller V, Singh GM, Onopa J, et al. Global Dietary Database 2017: data availability and gaps on 54 major foods, beverages and nutrients among 5.6 million children and adults from 1220 surveys worldwide. *BMJ Global Health* 2021; 6(2): e003585.

263. Gobbo DLC, Khatibzadeh S, Imamura F, et al. Assessing global dietary habits: a comparison of national estimates from the FAO and the Global Dietary Database. *The American Journal of Clinical Nutrition* 2015; 101(5): 1038-46.
264. Micha R, Khatibzadeh S, Shi P, et al. Global, regional and national consumption of major food groups in 1990 and 2010: a systematic analysis including 266 country-specific nutrition surveys worldwide. *BMJ Open* 2015; 5(9): e008705.
265. Rennie KL, Coward A, Jebb SA. Estimating under-reporting of energy intake in dietary surveys using an individualised method. *British Journal of Nutrition* 2007; 97(6): 1169-76.
266. Freedman LS, Commins JM, Moler JE, et al. Pooled results from 5 validation studies of dietary self-report instruments using recovery biomarkers for energy and protein intake. *American journal of epidemiology* 2014; 180(2): 172-88.
267. N. C. D. Risk Factor Collaboration. Trends in adult body-mass index in 200 countries from 1975 to 2014: a pooled analysis of 1698 population-based measurement studies with 19·2 million participants. *The Lancet* 2016; 387(10026): 1377-96.
268. Murray CJL, Ezzati M, Lopez AD, Rodgers A, Vander Hoorn S. Comparative quantification of health risks: conceptual framework and methodological issues. *Population Health Metrics* 2003; 1(1): 1.
269. Lim SS, Vos T, Flaxman AD, et al. A comparative risk assessment of burden of disease and injury attributable to 67 risk factors and risk factor clusters in 21 regions, 1990–2010: a systematic analysis for the Global Burden of Disease Study 2010. *The Lancet* 2012; 380(9859): 2224-60.
270. Forouzanfar MH, Alexander L, Anderson HR, et al. Global, regional, and national comparative risk assessment of 79 behavioural, environmental and occupational, and metabolic risks or clusters of risks in 188 countries, 1990–2013: a systematic analysis for the Global Burden of Disease Study 2013. *The Lancet* 2015; 386(10010): 2287-323.
271. Murray CJL, Ezzati M, Flaxman AD, et al. GBD 2010: design, definitions, and metrics. *Lancet* 2012; 380(9859): 2063-6.
272. Wang H, Abbas KM, Abbasifard M, et al. Global age-sex-specific fertility, mortality, healthy life expectancy (HALE), and population estimates in 204 countries and territories, 1950–2019: a comprehensive demographic analysis for the Global Burden of Disease Study 2019. *The Lancet* 2020; 396(10258): 1160-203.
273. Afshin A, Micha R, Khatibzadeh S, Mozaffarian D. Consumption of nuts and legumes and risk of incident ischemic heart disease, stroke, and diabetes: a systematic review and meta-analysis. *The American Journal of Clinical Nutrition* 2014: ajcn.076901.
274. Singh GM, Danaei G, Farzadfar F, et al. The Age-Specific Quantitative Effects of Metabolic Risk Factors on Cardiovascular Diseases and Diabetes: A Pooled Analysis. *PLOS ONE* 2013; 8(7): e65174.
275. Micha R, Shulkin ML, Peñalvo JL, et al. Etiologic effects and optimal intakes of foods and nutrients for risk of cardiovascular diseases and diabetes: Systematic reviews and meta-analyses from the Nutrition and Chronic Diseases Expert Group (NutriCoDE). *PLOS ONE* 2017; 12(4): e0175149.
276. Aune D, Giovannucci E, Boffetta P, et al. Fruit and vegetable intake and the risk of cardiovascular disease, total cancer and all-cause mortality–a systematic review and dose-response meta-analysis of prospective studies. *International Journal of Epidemiology* 2016.
277. Bechthold A, Boeing H, Schwedhelm C, et al. Food groups and risk of coronary heart disease, stroke and heart failure: A systematic review and dose-response meta-analysis of prospective studies. *Critical Reviews in Food Science and Nutrition* 2019; 59(7): 1071-90.

278. GBD 2017 Diet Collaborators. Health effects of dietary risks in 195 countries, 1990–2017: a systematic analysis for the Global Burden of Disease Study 2017. *The Lancet* 2019; 393(10184): 1958-72.
279. Schwingshackl L, Knüppel S, Schwedhelm C, et al. Perspective: NutriGrade: A Scoring System to Assess and Judge the Meta-Evidence of Randomized Controlled Trials and Cohort Studies in Nutrition Research. *Advances in Nutrition: An International Review Journal* 2016; 7(6): 994-1004.
280. Schwingshackl L, Hoffmann G, Lampousi AM, et al. Food groups and risk of type 2 diabetes mellitus: a systematic review and meta-analysis of prospective studies. *European Journal of Epidemiology* 2017; 32(5): 363-75.
281. Schwingshackl L, Knüppel S, Michels N, et al. Intake of 12 food groups and disability-adjusted life years from coronary heart disease, stroke, type 2 diabetes, and colorectal cancer in 16 European countries. *European Journal of Epidemiology* 2019; 34(8): 765-75.
282. World Cancer Research Fund/American Institute for Cancer R. Diet, Nutrition, Physical Activity and Cancer: A Global Perspective. Continuous Update Project Expert Report. 2018.
283. Aune D, Lau R, Chan DSM, et al. Dairy products and colorectal cancer risk: a systematic review and meta-analysis of cohort studies. *Annals of Oncology: Official Journal of the European Society for Medical Oncology* 2012; 23(1): 37-45.
284. Aune D, Norat T, Romundstad P, Vatten LJ. Dairy products and the risk of type 2 diabetes: a systematic review and dose-response meta-analysis of cohort studies. *The American Journal of Clinical Nutrition* 2013; 98(4): 1066-83.
285. Mohan D, Mente A, Dehghan M, et al. Associations of Fish Consumption With Risk of Cardiovascular Disease and Mortality Among Individuals With or Without Vascular Disease From 58 Countries. *JAMA Internal Medicine* 2021.
286. Institute of Medicine. Dietary Reference Intakes for Energy, Carbohydrate, Fiber, Fat, Fatty Acids, Cholesterol, Protein, and Amino Acids. Washington, DC; 2005.
287. Aune D, Keum N, Giovannucci E, et al. Nut consumption and risk of cardiovascular disease, total cancer, all-cause and cause-specific mortality: a systematic review and dose-response meta-analysis of prospective studies. *BMC medicine* 2016; 14(1): 207.
288. Aune D, Keum N, Giovannucci E, et al. Whole grain consumption and risk of cardiovascular disease, cancer, and all cause and cause specific mortality: systematic review and dose-response meta-analysis of prospective studies. *BMJ (Clinical research ed)* 2016; 353: i2716.
289. Aune D, Giovannucci E, Boffetta P, et al. Fruit and vegetable intake and the risk of cardiovascular disease, total cancer and all-cause mortality—a systematic review and dose-response meta-analysis of prospective studies. *International Journal of Epidemiology* 2017; 46(3): 1029-56.
290. Murray CJL, Aravkin AY, Zheng P, et al. Global burden of 87 risk factors in 204 countries and territories, 1990–2019: a systematic analysis for the Global Burden of Disease Study 2019. *The Lancet* 2020; 396(10258): 1223-49.
291. Willett W, Rockström J, Loken B, et al. Food in the Anthropocene: the EAT–Lancet Commission on healthy diets from sustainable food systems. *The Lancet* 2019; 393(10170): 447–92.
292. Global Bmi Mortality Collaboration DE, Di Angelantonio E, Bhupathiraju S, et al. Body-mass index and all-cause mortality: individual-participant-data meta-analysis of 239 prospective studies in four continents. *Lancet (London, England)* 2016; 388(10046): 776-86.

293. Satija A, Yu E, Willett WC, Hu FB. Understanding Nutritional Epidemiology and Its Role in Policy. *Advances in Nutrition* 2015; 6(1): 5-18.
294. Collaboration PS, Whitlock G, Lewington S, et al. Body-mass index and cause-specific mortality in 900 000 adults: collaborative analyses of 57 prospective studies. *Lancet* 2009; 373(9669): 1083-96.
295. Zheng J, Huang T, Yu Y, Hu X, Yang B, Li D. Fish consumption and CHD mortality: an updated meta-analysis of seventeen cohort studies. *Public Health Nutrition* 2012; 15(4): 725-37.
296. Schwingshackl L, Hoffmann G, … KIAjo, Undefined. Food groups and intermediate disease markers: a systematic review and network meta-analysis of randomized trials. *American Journal of Clinical Nutrition* 2018; 108(3): 576-86.
297. Xun P, Qin B, Song Y, et al. Fish consumption and risk of stroke and its subtypes: accumulative evidence from a meta-analysis of prospective cohort studies. *European Journal of Clinical Nutrition* 2012; 66(11): 1199-207.
298. Jayedi A, Shab-Bidar S, Eimeri S, Djafarian K. Fish consumption and risk of all-cause and cardiovascular mortality: A dose-response meta-analysis of prospective observational studies. *Public Health Nutrition* 2018; 21(7): 1297-306.
299. Guasch-Ferré M, Satija A, Blondin SA, et al. Meta-Analysis of Randomized Controlled Trials of Red Meat Consumption in Comparison With Various Comparison Diets on Cardiovascular Risk Factors. *Circulation* 2019; 139(15): 1828-45.
300. Miller RE, Blair PD. Input-output analysis: foundations and extensions. Cambridge, UK: Cambridge University Press; 2009.
301. Dietzenbacher E, Los B, Stehrer R, Timmer M, De Vries G. The construction of world input–output tables in the WIOD project. *Economic Systems Research* 2013; 25(1): 71-98.
302. Stadler K, Wood R, Bulavskaya T, et al. EXIOBASE 3: Developing a time series of detailed environmentally extended multi-regional input-output tables. *Journal of Industrial Ecology* 2018; 22(3): 502-15.
303. WBG. Consumer price index (2010 = 100). 2020. https://data.worldbank.org/indicator/FP.CPI.TOTL?end=2017&locations=US&start=2000.
304. WHO. Global Health Expenditure Database: Indicators and data. Geneva, Switzerland: World Health Organization; 2019.
305. Pichler P-P, Jaccard IS, Weisz U, Weisz H. International comparison of health care carbon footprints. *Environmental Research Letters* 2019; 14(6): 064004.
306. Health Care Without Harm. Health Care's Climate Footprint: Health Care Without Harm, 2019.
307. Gütschow J, Jeffery L, Gieseke R, Günther A. The PRIMAP-hist national historical emissions time series (1850-2017). v2.1. 2019. https://doi.org/10.5880/pik.2019.018.
308. Lenzen M, Malik A, Li M, et al. The environmental footprint of health care: a global assessment. *The Lancet Planetary Health* 2020; 4(7): e271-e9.
309. WHO. Current health expenditure by financing schemes, in Global Health Expenditure Database. In: Organization WH, editor.; 2020.
310. UNSD. Basic Data Selection. United Nations Statistics Division; 2019.
311. Swiss R. Sigma explorer. 2022.
312. IMF. World Economic Outlook Database. 2021.
313. Aldy JE, Viscusi WK. Age Differences in the Value of Statistical Life: Revealed Preference Evidence. *Review of Environmental Economics and Policy* 2007; 1(2): 241-60

314. Robinson LA, Sullivan R, Shogren JF. Do the Benefits of COVID-19 Policies Exceed the Costs? Exploring Uncertainties in the Age–VSL Relationship. *Risk Analysis* 2020.
315. World Bank Group. Population, total,. Washington, DC, USA; 2022.
316. OECD. Gross domestic product (GDP) : GDP per head, US $, constant prices, constant PPPs, reference year 2015, millions. 2021.
317. WHO. WHO methods and data sources for global burden of disease estimates 2000-2015 2017.
318. OECD. Mortality Risk Valuation in Environment, Health and Transport Policies. OECD publishing; 2012. p. 3.
319. ILO. ILOSTAT Statistics on wages. 2021. https://ilostat.ilo.org/topics/wages/ (accessed 1st April 2021).
320. ILO. ILOSTAT: Concepts and Definitions. 2022.
321. IMF. International Finance Statistics. 2022.
322. Muriithi MK, Mutegi RG, Mwabu G. Counting unpaid work in Kenya: Gender and age profiles of hours worked and imputed wage incomes. *The Journal of the Economics of Ageing* 2020; 17: 100120-.
323. Reddy AA, Mittal S, Singha Roy N, Kanjilal-Bhaduri S. Time Allocation between Paid and Unpaid Work among Men and Women: An Empirical Study of Indian Villages. *Sustainability* 2021; 13(5): 2671-.
324. IEA. World Energy Investment 2022. Paris, 2022.
325. Buchko M. Global Coal Mining, 2021.
326. Kanda S. Global Oil & Gas Exploration & Production. 2021.
327. IRENA and ILO. Renewable Energy and Jobs - Annual Review 2021. Special Edition: Labour and Policy Perspectives. Abu Dhabi: IRENA; 2021.
328. Stand.earth, org. Global Fossil Fuel Commitments Database. 2022.
329. IEA. Energy subsidies - tracking the impact of fossil fuel subsidies. 2021. https://www.iea.org/topics/energy-subsidies (accessed 16th February 2021).
330. IEA. Energy subsidies. Methodology and assumptions - the price-gap approach. 2022.
331. OECD. OECD Inventory of support measures for fossil fuels. 2021. https://stats.oecd.org/Index.aspx?DataSetCode=FFS_AUS (accessed 6th April 2021).
332. Oecd. OECD Companion to the Inventory of Support Measures for Fossil Fuels 2018. Paris, France: OECD Publishing; 2018.
333. World B. World Bank Carbon Pricing Dashboard. 2021.
334. IEA. International Energy Agency Greenhouse Gas Emissions from Energy, 1751-2020. [data collection]. 13th ed: UK Data Service. SN 5181; 2021.
335. European C. Auctioning. 2022.
336. WHO. World Health Organization Global Health Expenditure Database. 2021. https://apps.who.int/nha/database/Select/Indicators/en (accessed 7th April 2021).
337. Mi Z, Zheng J, Meng J, et al. Economic development and converging household carbon footprints in China. *Nature Sustainability* 2020; 3(7): 529-37.
338. Stadler K, Wood R, Bulavskaya T, et al. EXIOBASE 3: Developing a Time Series of Detailed Environmentally Extended Multi-Regional Input-Output Tables. *Journal of Industrial Ecology* 2018; 22(3): 502-15.
339. Friedlingstein P, Jones MW, O'Sullivan M, et al. Global Carbon Budget 2021. *Earth System Science Data Discussions* 2021; 2021: 1-191.
340. World Bank. World Bank Open Data. 2022.
341. Wto. WTO STATS. 2022.

342. Crippa M, Oreggioni G, Guizzardi D, et al. Fossil CO2 and GHG emissions of all world countries. Luxembourg (Luxembourg): Publications Office of the European Union, 2019.
343. Intergovernmental_Panel_on_Climate_Change. Summary for Policymakers: Climate Change 2021 - The Physical Science Basis. Contribution of WG II to the 6th assessment report: IPCC, 2021.
344. Tpi. Transition Pathway Initiative: Oil & Gas Sector. 2022.
345. Sbti. The Science Based Targets initiative (SBTi): About Us. 2022.
346. Sei, Iisd, Odi, E3G, Unep. The Production Gap Report 2021, 2021.
347. Climate A. Net Zero Company Benchmark, 2022.
348. Cti. Adapt to Survive: Why oil companies must plan for net zero and avoid stranded assets, 2021.
349. Oci. Big Oil Reality Check: Assessing Oil And Gas Company Climate Plans. Washington DC, 2020.
350. Li M, Trencher G, Asuka J. The clean energy claims of BP, Chevron, ExxonMobil and Shell: A mismatch between discourse, actions and investments. *PLoS ONE* 2022; 17(2): e0263596.
351. Sbti. Sectoral Decarbonization Approach (SDA): A method for setting corporate emission reduction targets in line with climate science, 2015.
352. Krabbe O, Linthorst G, Blok K, et al. Aligning corporate greenhouse-gas emissions targets with climate goals. *Nature Climate Change* 2015; 5(12): 1057-60.
353. Sbti. Value Change in the Value Chain: Best Practices In Scope 3 Greenhouse Gas Management, 2018.
354. World Resources I, Laurent Corbier World Business Council for Sustainable D. Greenhouse Gas Protocol: a Corporate Accounting and Reporting Standard; 2004.
355. Government HM. Environmental reporting guidelines: including Streamlined Energy and Carbon Reporting requirements. London, UK, 2019.
356. Cdp. CDP S&P 500 Climate Change Report 2013, 2013.
357. Cdp. Now For Nature: The Decade of Delivery. CDP Europe Report 2021. Berlin, Germany, 2021.
358. Dietz S, Gardiner D, Hastreiter N, Jahn V, Noels J. Carbon Performance assessment of oil & gas producers: note on methodology. London, 2021.
359. Sbti. What is the SBTi's policy on fossil fuel companies? ; 2022.
360. Rogelj J, Shindell D, Jiang K, et al. Mitigation Pathways Compatible with 1.5°C in the Context of Sustainable Development. In: Masson-Delmotte V, Zhai P, Pörtner HO, et al., eds. Global Warming of 15°C An IPCC Special Report on the impacts of global warming of 15°C above pre-industrial levels and related global greenhouse gas emission pathways, in the context of strengthening the global response to the threat of climate change; 2018.
361. Sbti. Foundations of Science-based Target Setting, 2019.
362. Huppmann D, Kriegler E, Krey V, et al. IAMC 1.5°C Scenario Explorer and Data hosted by IIASA. 2019.
363. IEA. World Energy Outlook 2020. Paris, France: International Energy Agency, 2021.
364. IEA. Net Zero by 2050: A Roadmap for the Global Energy Sector. Paris, 2021.
365. Chopra P, Karagiannopoulos L. Big Oil faces major reserves challenge as new discoveries fail to replace production. Rystad Energy. 2021 2021/05/05.
366. Rystad Energy. UCube Database. 2022.
367. IEA. Key World Energy Statistics 2021. Paris, 2021.
368. IEA. World Energy Outlook 2020 Paris, France: International Energy Agency, 2021.

369. Smith DA. Situating Wikipedia as a health information resource in various contexts: A scoping review. *PloS one* 2020; 15(2): e0228786.
370. Herlihy N, Bar-Hen A, Verner G, et al. Climate change and human health: what are the research trends? A scoping review protocol. *BMJ open* 2016; 6(12): e012022.
371. Herlihy N, Bar-Hen A, Verner G, et al. Climate change and health: scoping review of scientific literature 1990-2015: Glenn Verner. *The European Journal of Public Health* 2016; 26(suppl_1): ckw174. 81.
372. Verner G, Schütte S, Knop J, Sankoh O, Sauerborn R. Health in climate change research from 1990 to 2014: positive trend, but still underperforming. *Global health action* 2016; 9(1): 30723.
373. Berrang-Ford L, Sietsma AJ, Callaghan M, et al. Systematic mapping of global research on climate and health: a machine learning review. *The Lancet Planetary Health* 2021; 5(8): e514-e25.
374. Baturo A, Dasandi N, Mikhaylov SJ. Understanding state preferences with text as data: Introducing the UN General Debate corpus. *Research & Politics* 2017; 4(2): 2053168017712821.
375. Benoit K, Watanabe K, Wang H, et al. quanteda: An R package for the quantitative analysis of textual data. *Journal of Open Source Software* 2018; 3.
376. Wolf T, Debut L, Sanh V, et al. Transformers: State-of-the-art natural language processing. Proceedings of the 2020 conference on empirical methods in natural language processing: system demonstrations; 2020; 2020. p. 38-45.
